# Supplementary material for: Conformational enantiodiscrimination for asymmetric construction of atropisomers
Source: Nat Commun. 2022 Aug 12;13:4735. doi: 10.1038/s41467-022-32432-8 (PMC9374765; doi:10.1038/s41467-022-32432-8)
Supplement: Supplementary file 1 — Supplementary Information [file 41467_2022_32432_MOESM1_ESM.pdf]

Supplementary Information

Conformational enantiodiscrimination for asymmetric construction of atropisomers

Shouyi Cen[1], Nini Huang[1], Dongsheng Lian[1], Ahui Shen[1], Mei-Xin Zhao[1]*, Zhipeng Zhang[1]*

[1]Key Laboratory for Advanced Materials and Joint International Research Laboratory of Precision Chemistry and Molecular Engineering, Feringa Nobel Prize Scientist Joint Research Center, Frontiers Science Center for Materiobiology and Dynamic Chemistry, School of Chemistry and Molecular Engineering, East China University of Science & Technology, Shanghai 200237, China

*Correspondence authors. email: mxzhao@ecust.edu.cn; zhipengzhang@ecust.edu.cn

Table of Contents

Supplementary Methods 2

1. General information 2

2. Asymmetric construction of BINAM derivatives 3

 2.1 Procedure for the synthesis of axial chiral ligands **L1**–**L11** 3

 2.2 Optimization of the reaction conditions 6

 2.3 General procedure for the asymmetric construction of BINAM derivatives 9

 2.4 Gram-scale reaction for the synthesis of chiral BINAM derivatives 9

 2.5 Synthetic transformation of **3a** to BINAM 9

3. Asymmetric construction of NOBIN derivatives 10

 3.1 Procedure for the synthesis of axial chiral ligands **L12**–**L14** 10

 3.2 Optimization of the reaction conditions 11

 3.3 General procedure for the asymmetric construction of NOBIN derivatives 14

 3.4 Gram-scale reaction for the synthesis of chiral NOBIN derivatives 14

 3.5 Synthetic transformation of **5a** to NOBIN 15

4. Characterization of intermediates, ligands, and products 15

5. NMR and HPLC spectra 56

6. X-Ray Crystallographic Data 203

Supplementary References 208

Supplementary Methods

1. General information

Chemicals were purchased from commercial suppliers and used without further purification unless otherwise stated. Analytical thin layer chromatography (TLC) was performed on precoated silica gel plates (SGF254). Flash column chromatography was performed on silica gel (90 Å, 300-400 mesh). Visualization of TLC was accomplished by irradiation with UV light at 254 nm. All new compounds were characterized by means of ^{1}H NMR, ^{13}C NMR and HR-MS. ^{1}H NMR and ^{13}C NMR spectra were recorded on a Bruker AVANCE III 400 MHz NMR spectrometer. High-resolution mass spectra (HR-MS) were recorded on a JEOC AccuTOF LC-plus 4G (ESI) or Waters GCT Premier (EI-TOF) mass spectrometer. Specific rotations were recorded on digital automatic polarimeter (WZZ-2S). Melting points were obtained for all crystalline solids on an INESA SGW X-4 Melting-Point Apparatus with microscope. Chemical shifts of ^{1}H NMR spectra were reported in ppm (δ) relative to tetramethylsilane (TMS) with the solvent resonance employed as the internal standard ($CDCl_3$, δ 7.26 ppm; DMSO-d_6, δ 2.50 ppm). Chemical shifts of ^{13}C NMR spectra are reported in ppm relative to $CDCl_3$ (77.16 ppm) or DMSO-d_6 (39.52 ppm), and were obtained with ^{1}H decoupling. ^{19}F NMR was recorded on a Bruker AVANCE III 400 NMR spectrometer ($CFCl_3$ as an external standard and low field is positive) and were obtained with ^{1}H decoupling. The following abbreviations or combinations thereof were used to explain the multiplicities: s = singlet, br = broad singlet, d = doublet, t = triplet, q = quartet, sept = septet, m = multiplet. Single crystal X-ray diffraction data was collected on a Bruker D8 Venture diffractometer. The enantiomeric ratios (e.r.) were determined via chiral HPLC (Shimadzu, Prominence LC-20A) using Daicel Chiralpak AD-H, AS-H, IC or Chiralcel OD-H column.

$Cu(MeCN)_4PF_6$ (CAS: 64443-05-6, 98% purity), *m*-xylene (CAS:108-38-3, 99% purity), 2-aminonaphthalene (CAS:91-59-8, 97% purity) benzyl chloroformate (CAS:501-53-1, 97% purity), 1,2-dichlorobenzene (CAS:95-50-1, 98% purity), pyridine (CAS:110-86-1, 99.5% purity) and 2-naphthol: (CAS:135-19-3, 98% purity) were purchased from Adamas. $Cu(acac)_2$ (CAS:13395-16-9, 99% purity) was purchased from Energy Chemical. 2-Naphthylhydrazine hydrochloride (CAS:2243-58-5, 95% purity) was purchased from Bide Pharmatech Ltd. Pyridinium chlorochromate (CAS:26299-14-9, 97% purity) was purchased from Leyan Pharmaceutical.

Abbreviations: EtOAc = ethyl acetate, DCM = dichloromethane, DCE = 1,2-dichloroethane, Et_2O = diethyl ether, MeOH = methanol, THF = tetrahydrofuran, DMF = *N*,*N*-dimethylformamide, MTBE = methyl *tert*-butyl ether, DME = 1,2-dimethoxyethane, Xylene = dimethylbenzene, Et_3N = triethylamine.

2. Asymmetric construction of BINAM derivatives

2.1 Procedure for the synthesis of axial chiral ligands L1–L11

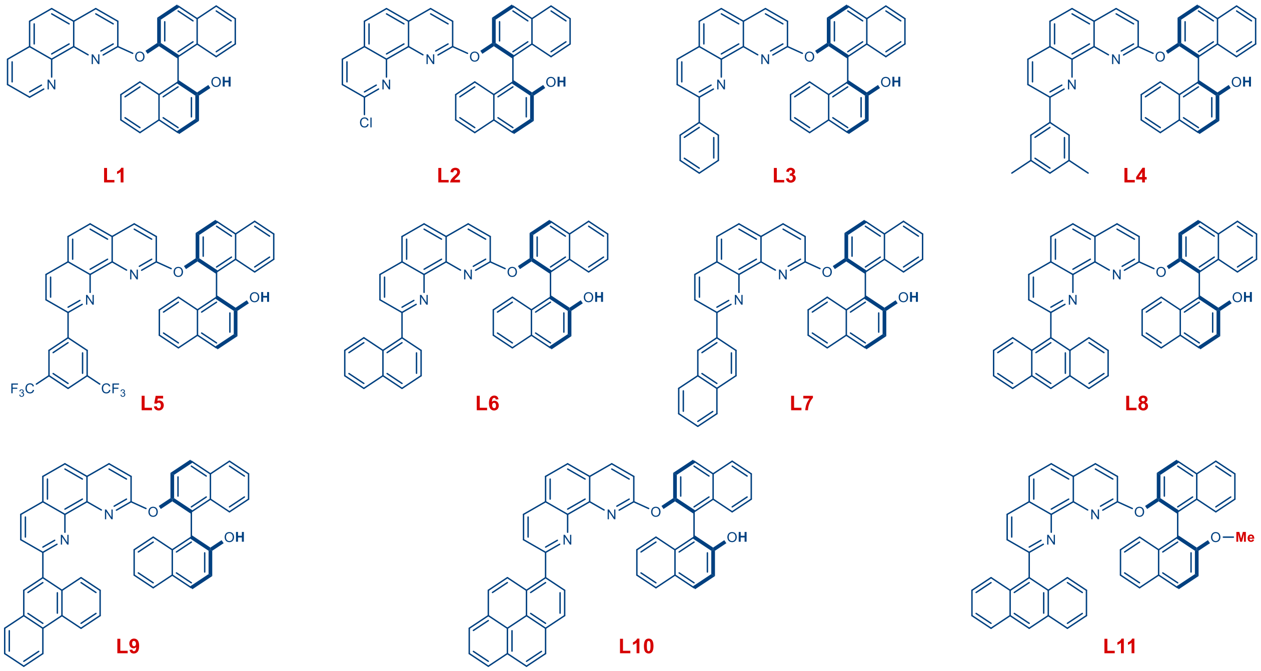

2.1.1 Ligand **L1** was synthesized according to the route shown in Supplementary Fig. 1. (*R*)-2'-methoxy-[1,1'-binaphthalen]-2-ol **6** and intermediate **7a** were synthesized according to the reported procedures[1,2].

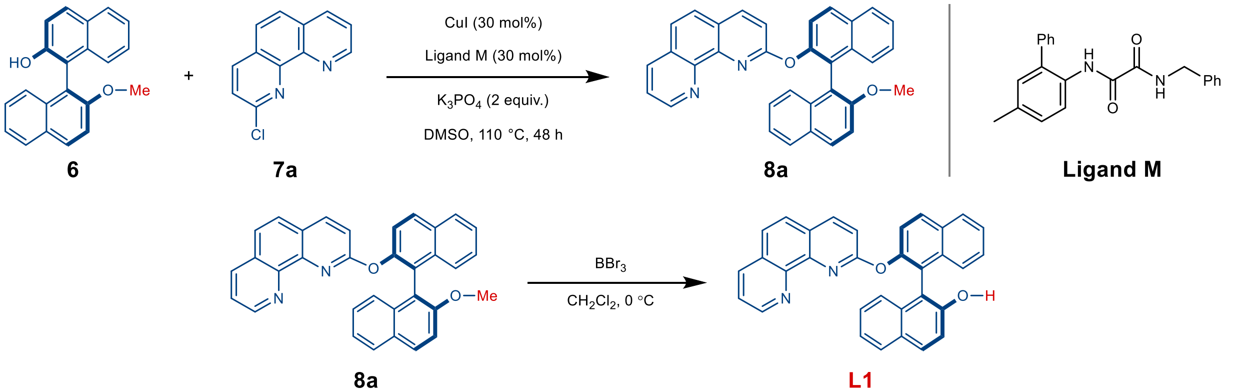

Supplementary Figure 1. Synthesis of ligand L1

To a 100 mL Schlenk flask were added (*R*)-2'-methoxy-[1,1'-binaphthalen]-2-ol **6** (0.510 g, 1.70 mmol), 2-chloro-1,10-phenanthroline **7a** (0.400 g, 1.87 mmol), CuI (97 mg, 0.51 mmol), ligand M (175 mg, 0.510 mmol), K_3PO_4 (0.720 g, 3.40 mmol) under N_2 atmosphere, followed by addition of anhydrous DMSO (10 mL) via a syringe, the reaction mixture was stirred at 110 °C for 48 h. After cooling down to room temperature, the reaction mixture was extracted with CH_2Cl_2 (3×20 mL). The combined organic layers were washed with brine, dried over Na_2SO_4. After removal of the solvent, the residue was purified by column chromatography on silica gel using petroleum ether/ethyl acetate (5:1) as the eluent giving compound **8a** (0.51 g, 62%) as white solid.

A flame-dried 100 mL Schlenk flask was charged with (*R*)-2-((2'-methoxy-[1,1'-binaphthalen]-2-yl)oxy)-1,10-phenanthroline **8a** (300 mg, 0.630 mmol), followed by addition of anhydrous CH_2Cl_2 (10 mL) via a syringe under N_2 atmosphere, then BBr_3 (17% in CH_2Cl_2, 1.9 mL, 1.90 mmol) was added dropwise to the solution at 0 °C, and the reaction mixture was stirred at room temperature for 12 h. The reaction was quenched with H_2O and extracted with CH_2Cl_2 (3×15 mL). The combined organic layers were washed with brine, dried over Na_2SO_4. After removal of the solvent under reduced pressure, the residue was purified by column chromatography on silica gel using CH_2Cl_2/MeOH (100:1) as the eluent giving compound **L1** (170 mg, 57% yield) as white solid.

2.1.2 Ligand **L2** was synthesized according to the route shown in Supplementary Fig. 2, following similar procedure for the preparation of **L1**. Intermediate **7b** was synthesized according to the reported procedures[2]. The ligand M developed by Ma and coworkers[3] is proved to be crucial to the success of this reaction.

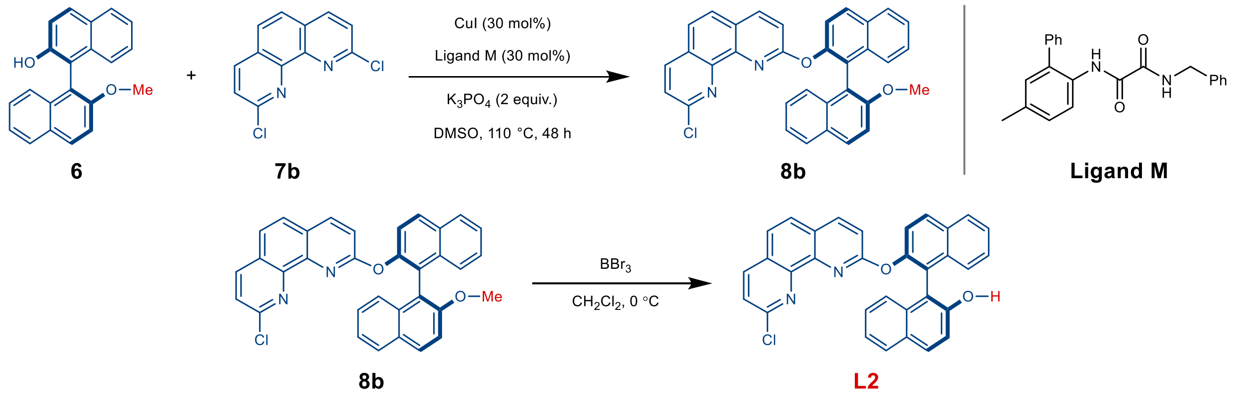

Supplementary Figure 2. Synthesis of ligand L2

2.1.3 Ligand **L3-L10** were synthesized according to the route shown in Supplementary Fig. 3 (taking **L8** for example).

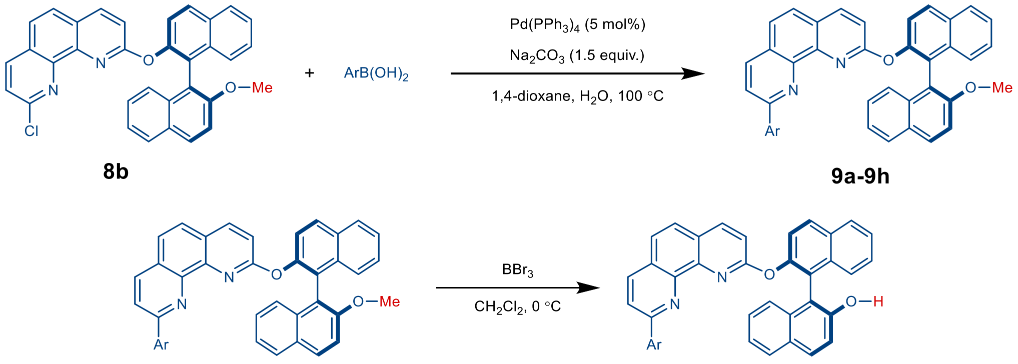

Supplementary Figure 3. Synthesis of ligands L3-L10

A 50 mL Schlenk tube equipped with a magnetic stir bar were charged with compound **8b** (150 mg, 0.290 mmol), tetrakis(triphenylphosphine)palladium (17.0 mg, 0.0150 mmol), anthracen-9-ylboronic acid (71.5 mg, 0.320 mmol) and Na_2CO_3 (46 mg, 0.44 mmol) under N_2 atmosphere, then 1,4-dioxane (4.0 mL) and H_2O (0.5 mL) were added via a syringe, and the mixture was stirred at 100 °C for 12 h. After cooling down to room temperature, the reaction mixture was filtered through a pad of celite and the filtrate was extracted with CH_2Cl_2 three times. The combined organic layers were washed with brine, dried over Na_2SO_4 and concentrated under reduced pressure. The residue was purified by column chromatography on silica gel using petroleum ether/ethyl acetate (5:1) as the eluent to afford the corresponding compound **9f** (99 mg, 52% yield) as yellow solid. **9f** was then deprotected according to the procedure shown in part **2.1.1** to afford **L8**.

2.2 Optimization of the reaction conditions

Supplementary Table 1. Screening of the copper salts[a]

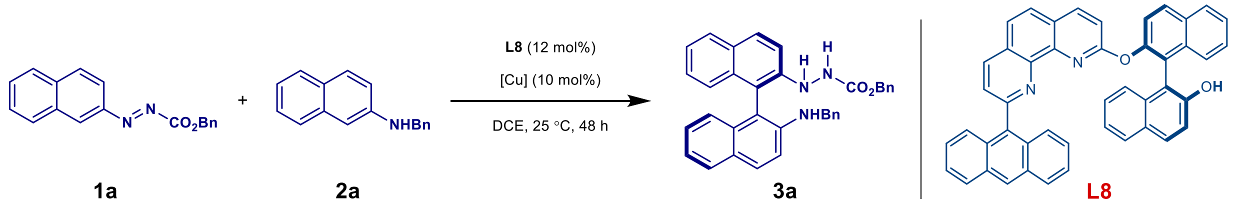

Entry	[Cu] (10 mol%)	Yield[b] (%)	E.r.[c] (*R*:*S*)
1	$CuBr{\cdot}SMe_2$	25	88:12
2	$CuBF_4{\cdot}6H_2O$	10	68:32
3	CuTc [d]	<1	N.D.
**4**	**$Cu(MeCN)_4PF_6$**	42	**95.5:4.5**
5	$Cu(TFA)_2$	<1	N.D.
6	CuSCN	<1	N.D.
7	$Cu(MeCN)_4BF_4$	25	90:10
8	$CuCl_2$	<1	N.D.
9	$CuBr_2$	<1	N.D.
10	$Cu(OTf)_2$	<1	N.D.
11	$Cu(NO_3)_2{\cdot}3H_2O$	<1	N.D.
12	CuI	<1	N.D.
13	$Cu(acac)_2$	<1	N.D.
14	CuCN	<1	N.D.
15	CuCl	<1	N.D.
16	CuBr	<1	N.D.
17	$Cu(OAc)_2$	<1	N.D.

[a]All reactions were carried out on 0.1 mmol scale in DCE (2.0 mL) at 25 °C. [b]Yields of the isolated products. [c]Enantiomeric ratios (e.r.) were determined via HPLC on a chiral stationary phase and reported as (*R*:*S*). [d]CuTc: copper(I) thiophene-2-carboxylate.

Supplementary Table 2. Screening of the solvents[a]

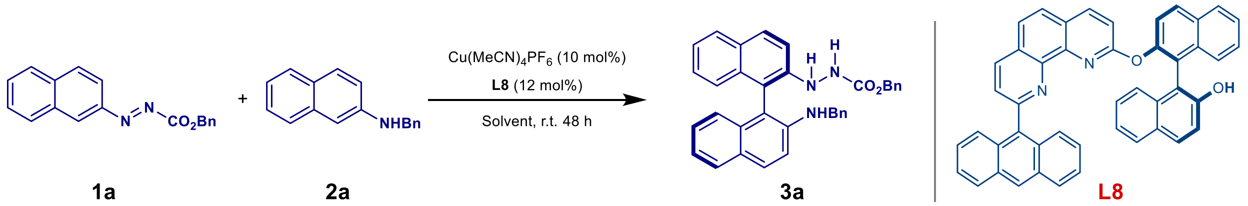

1a 2a 3a L8

Entry	Solvent (2.0 mL)	Yield[b] (%)	E.r.[c] (*R*:*S*)
1	Toluene	<1	N.D.
2	THF	<1	N.D.
3	MeCN	<1	N.D.
4	MeOH	22	61:39
5	CH_2Cl_2	37	77.5:22.5
6	$CHCl_3$	8	85:15
7	CCl_4	<1	N.D.
8	DCE	42	95.5:4.5
9	PhCl	25	93:7
10	**1,2-dichlorobenzene**	50	**96:4**

[a]All reactions were carried out on 0.1 mmol scale in solvent (2.0 mL) at 25 °C. [b]Yields of the isolated products. [c]Enantiomeric ratios (e.r.) were determined via HPLC on a chiral stationary phase and reported as (*R*:*S*).

Supplementary Table 3. Optimization of the reaction temperature[a]

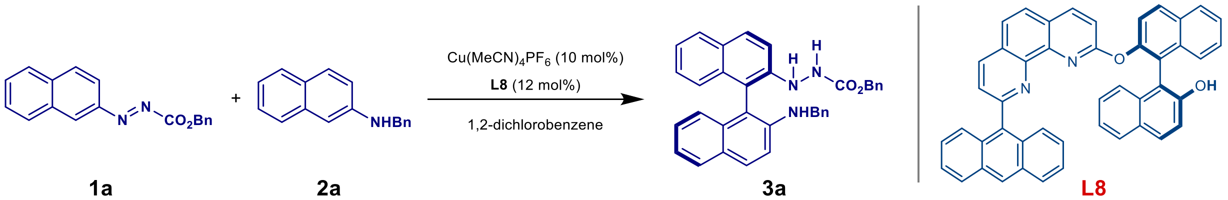

1a 2a 3a L8

Entry	T (°C)	Yield[b] (%)	E.r.[c] (*R*:*S*)
1	25	50	96:4
2	**30**	67	**97.5:2.5**
3	35	86	96:4
4	40	70	95:5

[a]All reactions were carried out on 0.1 mmol scale in 1,2-dichlorobenzene (2.0 mL) at corresponding temperature for 48 h. [b]Yields of the isolated products. [c]Enantiomeric ratios (e.r.) were determined via HPLC on a chiral stationary phase and reported as (*R*:*S*).

Supplementary Table 4. Optimization of the reaction time[a]

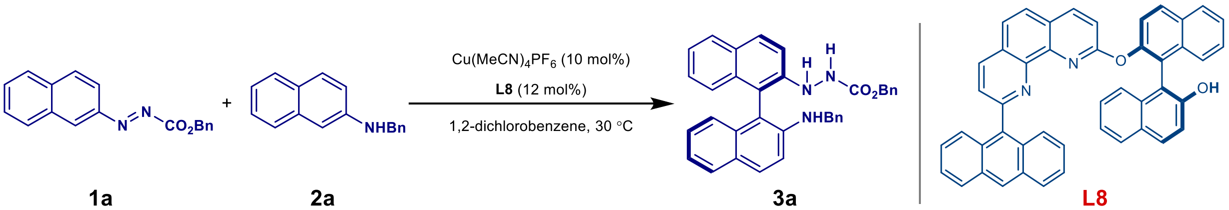

1a 2a 3a L8

Entry	Time (h)	Yield[b] (%)	E.r.[c] (*R*:*S*)
1	48	67	97.5:2.5
2	**60**	**91**	**97.5:2.5**
3	72	76	97:3

[a]All reactions were carried out on 0.1 mmol scale in 1,2-dichlorobenzene (2.0 mL) at 30 °C. [b]Yields of the isolated products. [c]Enantiomeric ratios (e.r.) were determined via HPLC on a chiral stationary phase and reported as (*R*:*S*).

2.3 General procedure for the asymmetric construction of BINAM derivatives

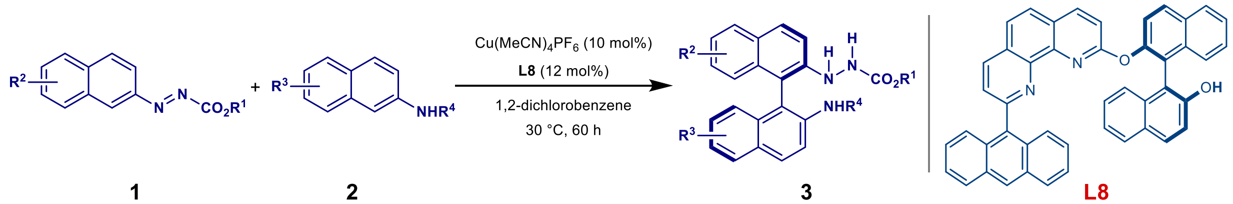

1 **2** **3** **L8**

To a solution of $Cu(MeCN)_4PF_6$ (3.7 mg, 0.010 mmol, 10 mol%) and **L8** (7.7 mg, 0.012 mmol, 12 mol%) in 1,2-dichlorobenzene (2.0 mL) were added azo compound **1** (0.10 mmol) and the 2-naphthylamine derivative **2** (0.12 mmol). The mixture was stirred under air at 30 °C for 60 h. Upon completion, the resulting mixture was directly purified by flash chromatography on silica gel using petroleum ether/ethyl acetate as the eluent to afford the desired products **3**.

2.4 Gram-scale reaction for the synthesis of chiral BINAM derivatives

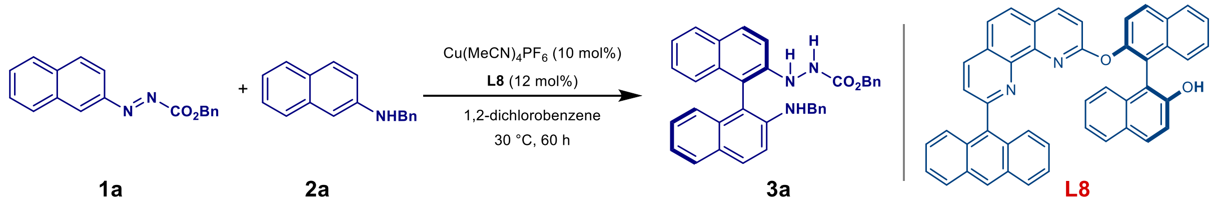

1a **2a** **3a** **L8**

1.00 g
(3.45 mmol)

79%, 97:3 e.r.

A round-bottom flask (250 mL) equipped with magnetic stir bar were charged with $Cu(MeCN)_4PF_6$ (128.5 mg, 0.3450 mmol), **L8** (265 mg, 0.410 mmol), and 1,2-dichlorobenzene (70 mL). The reaction mixture was stirred at ambient temperature for 10 minutes, then azo compound **1a** (1.00 g, 3.45 mmol) and *N*-benzyl-2-naphthylamine **2a** (0.950 g, 4.10 mmol) were added to the solution. The reaction mixture was stirred under air at 30 °C for 60 h. The solvent was removed under reduced pressure and the residue was purified by column chromatography on silica gel using petroleum ether/ethyl acetate (5:1) as the eluent to afford the products **3a** (1.42 g, 79% yield, 97:3 e.r.).

2.5 Synthetic transformation of 3a to BINAM

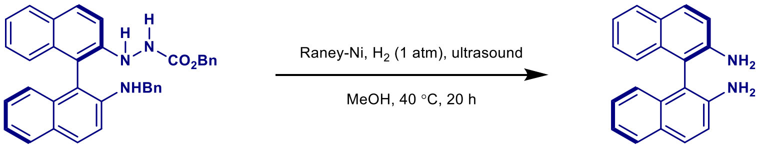

3a
97:3 e.r.

(*R*)-BINAM
85% yield, 97:3 e.r.

Product **3a** was transformed to BINAM using a method reported by Tan and coworkers[4]. To a solution of **3a** (300 mg, 0.570 mmol, 97:3 e.r.) in MeOH (10 mL) was added Raney-Ni (approximately 500 mg) which washed with MeOH for three times and dispersed in the solution. The reaction flask was evacuated under vacuum and back-filled with H_2. Then the reaction flask was immersed in an ultrasonic cleaner filled with water, and sonicated under H_2 atmosphere (1 atm) for 20 h until the starting material **3a** was fully consumed (bath temperature was measured to be 40 °C). The reaction mixture was filtered through a pad of celite and the filtrate was concentrated under reduced pressure. The residue was purified by column chromatography on silica gel using petroleum ether/ethyl acetate (6:1) as the eluent to afford (*R*)-BINAM (138 mg, 85% yield, 97:3 e.r.) as white solid. The absolute configuration of **3a** was established to be (*R*) by comparison of the specific rotation of the hydrogenated product with the literature-reported value for a sample of (*R*)-BINAM. Specific rotation of the product: $[\alpha]_D^{25}$ = +104.8 (*c* = 0.5, $CHCl_3$); Lit.[4]: $[\alpha]_D^{20}$ = +112.8 (*c* = 0.5, $CHCl_3$) for a sample with 97:3 e.r. (*R*). All other compounds **3** were assigned to be (*R*) by analogy.

3. Asymmetric construction of NOBIN derivatives

3.1 Procedure for the synthesis of axial chiral ligands L12–L14

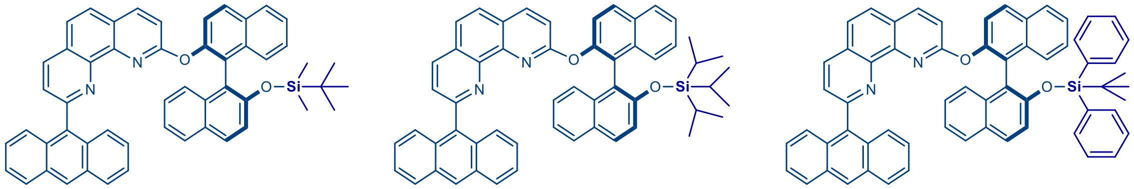

Ligand **L12-L14** were synthesized according to the route shown in Supplementary Fig. 4. The procedures are shown as follows (taking **L14** for example):

A flame-dried Schlenk flask was charged with **L8** (96.1 mg, 0.150 mmol), followed by addition of CH_2Cl_2 via a syringe, sodium hydride (12 mg, 0.30 mmol, 60 % dispersion in mineral oil) was added. Then $SiPh_2{}^tBuCl$ (78 μL, 0.30 mmol) was added dropwise to the solution at 0 °C, and the reaction mixture was stirred at room temperature for 1 h. The reaction was quenched with H_2O and extracted with CH_2Cl_2. The combined organic layers were washed with brine, dried over Na_2SO_4. After removal of the solvent under reduced pressure, the residue was purified by column

chromatography on silica gel using petroleum ether/ethyl acetate (15:1) as the eluent giving the desired product **L14** (121.3 mg, 92% yield).

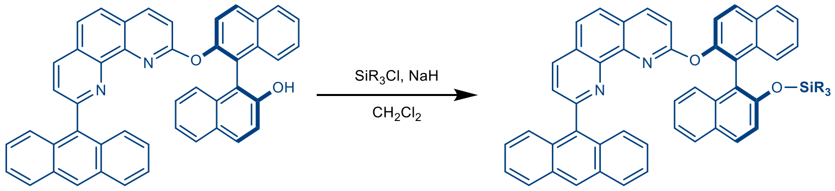

L8 L12-L14

Supplementary Figure 4. Synthesis of ligands L12-L14

3.2 Optimization of the reaction conditions

Supplementary Table 5. Screening of the solvents[a]

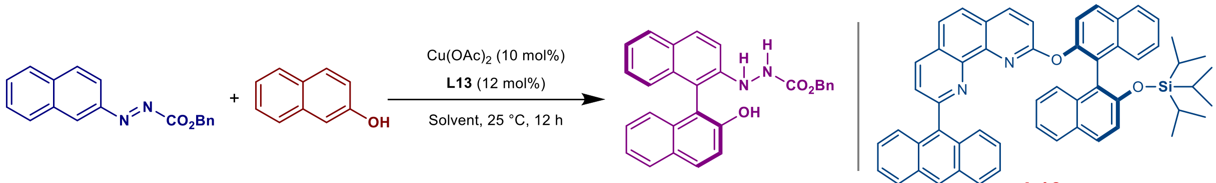

1a 4a 5a L13

Entry	Solvent (2.0 mL)	Yield[b] (%)	E.r.[c] (*R*:*S*)
1	CH_2Cl_2	31	24:76
2	DCE	28	32.5:67.5
3	$CHCl_3$	29	18.5:81.5
4	Et_2O	38	8.5:91.5
5	MTBE	53	10.5:89.5
6	THF	<1	N.D.
7	DME	<1	N.D.
8	Dioxane	<1	N.D.
9	MeOH	<1	N.D.
10	MeCN	<1	N.D.
11	Toluene	44	4:96
12	Ethylbenzene	**64**	**3.5:96.5**

[a]All reactions were carried out on 0.1 mmol scale in corresponding solvent (2.0 mL) at 25 °C for 12 h. [b]Yields of the isolated products. [c]Enantiomeric ratios (e.r.) were determined via HPLC on a chiral stationary phase and reported as (*R*:*S*).

Supplementary Table 6. Screening of the copper salts[a]

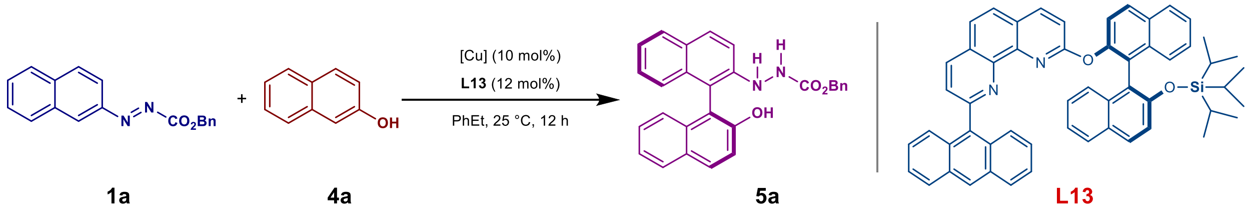

Entry	[Cu] (10 mol%)	Yield[b] (%)	E.r.[c] (*R*:*S*)
1	$Cu(OAc)_2$	64	3.5:96.5
2	**$Cu(acac)_2$**	**94**	**2.5:97.5**
3	$CuSO_4$	35	6.5:93.5
4	$Cu(NO_3)_2$	23	33.5:66.5
5	$Cu(TFA)_2$	<1	N.D.
6	$Cu(OTf)_2$	12	49:51
7	$CuCl_2$	<1	N.D.
8	$CuBr_2$	<1	N.D.
9	CuBr	16	26:74
10	CuCl	21	21.5:78.5
11	CuI	31	11.5:88.5
12	$Cu(CH_3CN)_4PF_6$	20	44.5:55.5
13	$CuBF_4{\cdot}6H_2O$	10	48:52

[a]All reactions were carried out on 0.1 mmol scale in ethylbenzene (2.0 mL) at 25 °C for 12 h. [b]Yields of the isolated products. [c]Enantiomeric ratios (e.r.) were determined via HPLC on a chiral stationary phase and reported as (*R*:*S*).

Supplementary Table 7. Screening of the solvents using Cu(acac)2 as copper salt[a]

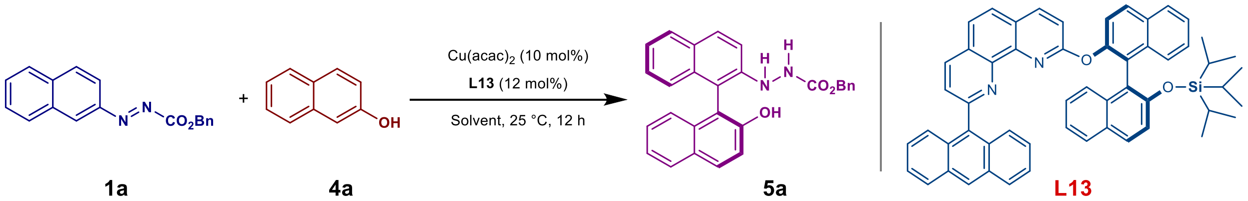

Entry	Solvent (2.0 mL)	Yield[b] (%)	E.r.[c] (*R*:*S*)
1	Ethylbenzene	94	2.5:97.5
2	Toluene	95	2.5:97.5
3	Xylene	84	3:97
**4**	***m*-Xylene**	**98**	**2.5:97.5**
5	*o*-Xylene	88	3:97

[a]All reactions were carried out on 0.1 mmol scale in corresponding solvent (2.0 mL) at 25 °C for 12 h. [b]Yields of the isolated products. [c]Enantiomeric ratios (e.r.) were determined via HPLC on a chiral stationary phase and reported as (*R*:*S*).

Supplementary Table 8. The effect of a base on the reaction using L8 as ligand[a]

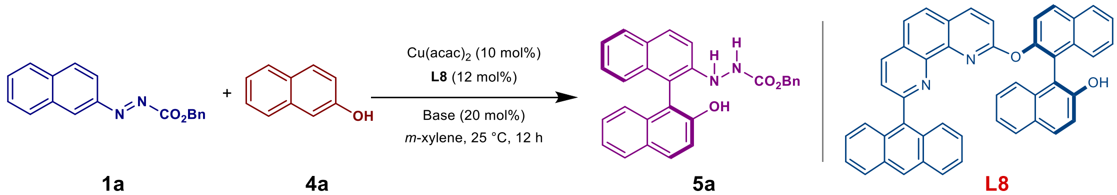

Entry	Base (20 mol%)	Yield[b] (%)	E.r.[c] (*R*:*S*)
1	/	70	66:34
2	$NaHCO_3$	**78**	**68.5:31.5**
3	Na_2CO_3	60	66:34
4	Cs_2CO_3	69	58.5:41.5
5	DMAP	78	67:33

[a]All reactions were carried out on 0.1 mmol scale in *m*-xylene (2.0 mL) at 25 °C for 12 h. [b]Yields of the isolated products. [c]Enantiomeric ratios (e.r.) were determined via HPLC on a chiral stationary phase and reported as (*R*:*S*).

3.3 General procedure for the asymmetric construction of NOBIN derivatives

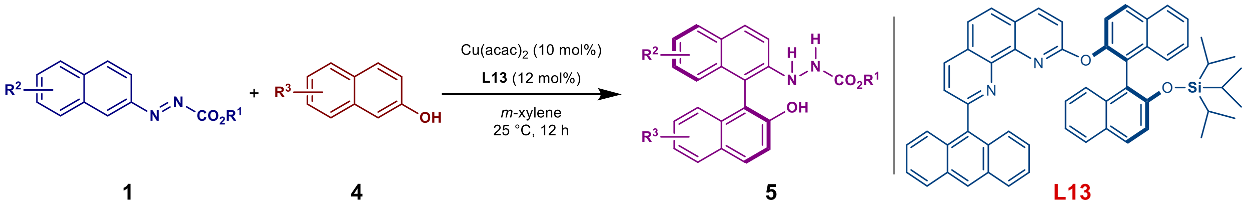

1 4 5 L13

To a solution of Cu(acac)$_2$ (2.6 mg, 0.010 mmol, 10 mol%) and **L13** (9.6 mg, 0.012 mmol, 12 mol%) in *m*-xylene (2.0 mL) were added azo compound **1** (0.10 mmol) and the 2-naphthylamine derivative **4** (0.12 mmol). The mixture was stirred under N_2 atmosphere at 25 °C for 12 h. Upon completion, the resulting mixture was directly purified by flash chromatography on silica gel using petroleum ether/ethyl acetate as the eluent to afford product **5**.

3.4 Gram-scale reaction for the synthesis of chiral NOBIN derivatives

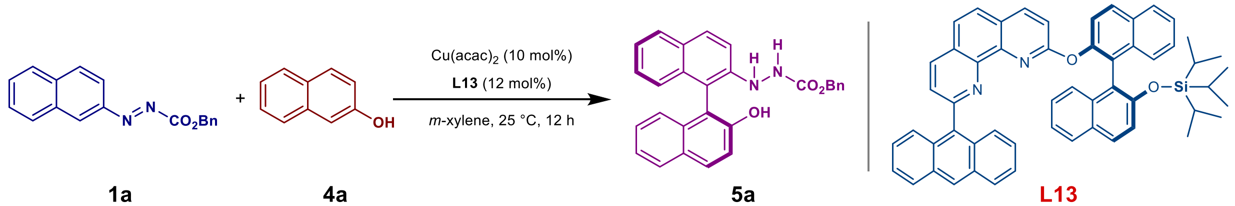

1a
1.16 g
(4.00 mol)

4a

5a
96%, 6:94 e.r.

L13

To a solution of Cu(acac)$_2$ (105 mg, 0.400 mmol) and **L13** (383 mg, 0.480 mmol) in *m*-xylene (30 mL) in a Schlenk flask, were added azo compound **1a** (1.16 g, 4.00 mmol) and 2-naphthol **4a** (692 mg, 4.80 mmol). The mixture was stirred under N_2 atmosphere at 25 °C for 12 h. The solvent was removed under reduced pressure and the residue was purified by column chromatography on silica gel using petroleum ether/ethyl acetate (6:1) as the eluent to afford the product **5a** (1.67 g, 96% yield, 6:94 e.r.) as a pale yellow solid.

3.5 Synthetic transformation of 5a to NOBIN

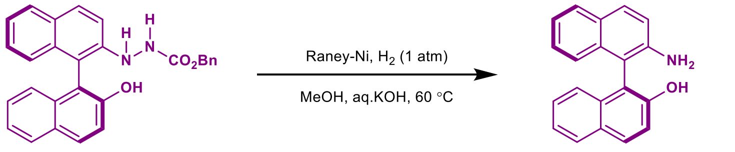

Product **5a** was transformed to NOBIN using a method reported by Tan and coworkers[4].

To a 25 mL Schlenk tube with a magnetic stir bar was added Raney-Ni catalyst (approximately 100 mg) which was washed with methanol (3×5 mL), a solution of **5a** (43.5 mg, 0.100 mmol) in methanol (5.0 mL) was added, followed by aqueous solution of KOH (1.0 mL, 2.0 M). The reaction flask was evacuated under vacuum and back-filled with H_2, then the reaction mixture was stirred and heated to 60 °C for 24 hours. After cooling to room temperature, the reaction mixture was filtered through a pad of celite. The methanol in the filtrate was removed under reduced pressure and the residue was extracted with CH_2Cl_2 (3×10 mL). The organic layers were combined, dried over Na_2SO_4, filtered and concentrated. The resulting crude product was purified by flash chromatography on silica gel using petroleum ether/ethyl acetate (5:1) as the eluent to afford (*S*)-NOBIN (27.3 mg, 96% yield, 6:94 e.r.). The absolute configuration of **5a** was established to be (*S*) by comparison of the specific rotation of the hydrogenated product with the literature-reported value for a sample of (*S*)-NOBIN. Specific rotation of the product: $[\alpha]_D^{25}$ = -42.4 (*c* = 0.5, $CHCl_3$); Lit.[4]: $[\alpha]_D^{20}$ = -54.0 (*c* = 0.5, $CHCl_3$) for a sample with 99.5:0.5 e.r. (*S*). All other compounds **6** were assigned to be (*S*) by analogy.

4. Characterization of intermediates, ligands, and products

(*R*)-2-((2'-methoxy-[1,1'-binaphthalen]-2-yl)oxy)-1,10-phenanthroline (8a)

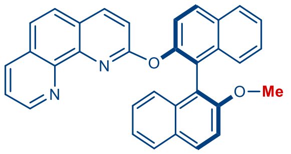

8a

White solid, 510 mg, Yield: 62%. m.p. 120–121 °C; $[\alpha]_D^{15}$ = +37.0 (*c* = 0.40, CH_2Cl_2); ^1H NMR (400 MHz, $CDCl_3$): δ 9.06 (dd, *J* = 4.4, 1.6 Hz, 1H), 8.15 (dd, *J* = 8.0, 2.0 Hz, 1H), 8.03 (d, *J* = 8.8 Hz, 1H), 8.00 (d, *J* = 8.8 Hz, 1H), 7.95 (d, *J* = 8.0 Hz, 1H), 7.86 (d, *J* = 8.8 Hz, 1H), 7.75 (d, *J*

= 8.0 Hz, 1H), 7.66 (d, *J* = 8.8 Hz, 1H), 7.62 (d, *J* = 8.8 Hz, 1H), 7.53 (dd, *J* = 8.4, 4.4 Hz, 1H), 7.49 (d, *J* = 9.2 Hz, 1H), 7.45 (ddd, *J* = 8.0, 6.0, 2.0 Hz, 1H), 7.29 (s, 1H), 7.27 (d, *J* = 2.0 Hz, 2H), 7.24 – 7.19 (m, 2H), 7.12 (ddd, *J* = 8.4, 7.2, 1.6 Hz, 1H), 7.08 (d, *J* = 8.8 Hz, 1H), 3.66 (s, 3H); ^{13}C NMR (101 MHz, $CDCl_3$): δ 163.09, 154.91, 150.77, 150.13, 145.41, 145.10, 139.25, 135.87, 134.48, 134.13, 131.42, 129.92, 129.83, 129.10, 129.04, 128.24, 127.84, 126.73, 126.45, 126.25, 125.88, 125.65, 125.33, 125.28, 125.23, 124.61, 123.55, 122.91, 121.25, 117.95, 113.22, 112.92, 56.30; HRMS (ESI-TOF) *m/z* Calcd. for $C_{33}H_{23}N_2O_2^+$ $[M+H]^+$: 479.1754; Found: 479.1743.

(*R*)-2-chloro-9-((2'-methoxy-[1,1'-binaphthalen]-2-yl)oxy)-1,10-phenanthroline (8b)

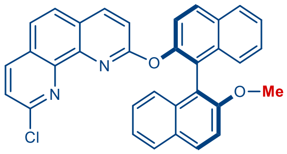

8b

White solid, 230 mg, Yield: 50%. m.p. 134–135 °C; $[\alpha]_D^{15}$ = +11.6 (*c* = 1.00, CH_2Cl_2); ^{1}H NMR (400 MHz, $CDCl_3$): δ 8.07 – 7.91 (m, 4H), 7.84 (d, *J* = 8.8 Hz, 1H), 7.73 (d, *J* = 8.0 Hz, 1H), 7.61 (d, *J* = 8.4, 1H), 7.55 (d, *J* = 8.4, 1H), 7.51 (d, *J* = 8.8 Hz, 1H), 7.49 – 7.40 (m, 2H), 7.29-7.26 (m, 4H), 7.19 (t, *J* = 7.2, Hz 1H), 7.11 (t, *J* = 7.6, Hz 1H), 7.05 (d, *J* = 8.4 Hz, 1H), 3.66 (s, 3H); ^{13}C NMR (101 MHz, $CDCl_3$): δ 163.14, 154.91, 151.02, 150.60, 145.00, 143.78, 139.23, 138.60, 134.37, 134.01, 131.42, 129.93, 129.80, 129.02, 128.21, 127.80, 127.61, 126.69, 126.42, 126.26, 126.19, 125.68, 125.64, 125.33, 125.20, 124.11, 123.81, 123.52, 121.33, 117.87, 113.49, 113.31, 56.35; HRMS (ESI-TOF) *m/z* Calcd. for $C_{33}H_{22}ClN_2O_2^+$ $[M+H]^+$: 513.1364; Found: 513.1354.

(*R*)-2-((2'-methoxy-[1,1'-binaphthalen]-2-yl)oxy)-9-phenyl-1,10-phenanthroline (9a)

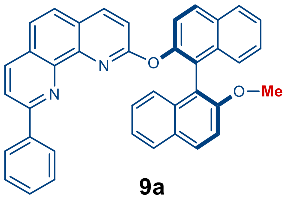

9a

White solid, 60 mg, Yield: 56%. m.p. 182–183 °C; $[\alpha]_D^{15}$ = -243.0 (*c* = 0.40, CH_2Cl_2); ^{1}H NMR (400 MHz, $CDCl_3$): δ 8.15 (d, *J* = 8.0 Hz, 3H), 8.12 (dd, *J* = 8.8 Hz, 1H), 8.03 (dd, *J* = 14.0, 8.4 Hz, 2H), 7.88 (d, *J* = 8.8 Hz, 1H), 7.82 (d, *J* = 8.8 Hz, 2H), 7.62 (d, *J* = 8.0 Hz, 1H), 7.58 – 7.48 (m, 3H), 7.46 (d, *J* = 8.4 Hz, 1H), 7.34 (d, *J* = 7.2 Hz, 1H), 7.32 – 7.25 (m, 4H), 7.24 (s, 1H), 6.98 (t, *J* = 7.2 Hz, 1H), 6.92 (d, *J* = 8.4 Hz, 1H), 6.61 (t, *J* = 7.6 Hz, 1H), 3.65 (s, 3H); ^{13}C NMR (101

MHz, $CDCl_3$): δ 162.90, 155.86, 155.04, 150.87, 145.02, 144.64, 139.10, 139.03, 136.55, 134.46, 134.02, 131.68, 129.72, 129.38, 129.22, 128.98, 128.70, 128.20, 127.79, 127.43, 127.33, 126.69, 126.41, 126.32, 125.74, 125.58, 125.53, 125.35, 125.19, 123.93, 123.37, 123.18, 119.20, 118.65, 113.74, 112.74, 56.79; HRMS (ESI-TOF) *m/z* Calcd. for $C_{39}H_{27}N_2O_2^+$ $[M+H]^+$: 555.2067; Found: 555.2057.

(*R*)-2-(3,5-dimethylphenyl)-9-((2'-methoxy-[1,1'-binaphthalen]-2-yl)oxy)-1,10-phenanthroline (9b)

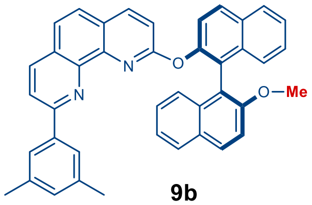

9b

White solid, 175 mg, Yield: 51%. m.p. 221–222 °C; $[\alpha]_D^{15}$ = -230.5 (*c* = 0.57, CH_2Cl_2); ^1H NMR (400 MHz, $CDCl_3$): δ 8.16 (d, *J* = 8.4 Hz, 1H), 8.11 (d, *J* = 8.8 Hz, 1H), 8.04 (d, *J* = 8.4 Hz, 1H), 7.98 (d, *J* = 8.4 Hz, 1H), 7.89 (d, *J* = 8.4 Hz, 1H), 7.87 – 7.81 (m, 4H), 7.63 (d, *J* = 8.0 Hz, 1H), 7.56 (dd, *J* = 14.8, 8.4 Hz, 2H), 7.47 (d, *J* = 8.4 Hz, 1H), 7.43 (d, *J* = 7.2 Hz, 1H), 7.30 (d, *J* = 8.8 Hz, 1H), 7.24 – 7.22 (m, 2H), 6.99 – 6.72 (m, 2H), 6.90 (d, *J* = 8.4 Hz, 1H), 6.70 (t, *J* = 7.2 Hz, 1H), 3.66 (s, 3H), 2.24 (s, 6H); ^{13}C NMR (101 MHz, $CDCl_3$): δ 162.78, 156.21, 155.06, 150.86, 144.95, 144.66, 139.06, 138.99, 138.22, 136.54, 134.44, 134.02, 131.51, 131.12, 129.72, 129.30, 129.16, 128.98, 128.28, 127.82, 127.31, 126.57, 126.39, 126.22, 125.89, 125.46, 125.37, 125.30, 125.12, 124.03, 123.39, 122.87, 119.52, 118.72, 113.79, 113.06, 56.84, 21.46; HRMS (ESI-TOF) *m/z* Calcd. for $C_{41}H_{31}N_2O_2^+$ $[M+H]^+$: 583.2380; Found: 583.2382.

(*R*)-2-(3,5-bis(trifluoromethyl)phenyl)-9-((2'-methoxy-[1,1'-binaphthalen]-2-yl)oxy)-1,10-phenanthroline (9c)

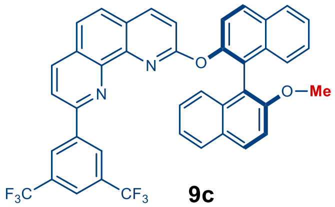

9c

White solid, 123 mg, Yield: 46%. m.p. 114–115 °C; $[\alpha]_D^{15}$ = -123.0 (*c* = 0.33, CH_2Cl_2); ^1H NMR (400 MHz, $CDCl_3$): δ 8.68 (s, 2H), 8.29 (d, *J* = 8.4 Hz, 1H), 8.13 (d, *J* = 9.2 Hz, 1H), 8.07 (d, *J* = 8.4 Hz, 1H), 8.01 (d, *J* = 8.0 Hz, 1H), 7.92 – 7.88 (m, 3H), 7.83 (d, *J* = 9.2 Hz, 1H), 7.65 (d, *J* =

8.4 Hz, 1H), 7.63 (s, 2H), 7.45 (ddd *J* = 8.0, 6.4, 1.2 Hz, 1H), 7.30 (d, *J* = 8.8 Hz, 1H), 7.28 – 7.26 (m, 1H), 7.23 – 7.21 (m, 2H), 7.02 (t, *J* = 7.6 Hz, 1H), 6.84 (d, *J* = 8.4 Hz, 1H), 6.74 (ddd, *J* = 8.0, 6.8, 0.8 Hz 1H), 3.63 (s, 3H); ^{13}C NMR (101 MHz, $CDCl_3$): δ 163.04, 155.11, 152.98, 150.69, 145.27, 144.56, 141.55, 139.13, 137.41, 134.29, 133.93, 132.13 (q, $J_{C\text{-}F}$ = 33.3 Hz), 131.63, 129.79, 129.44, 128.98, 128.55, 128.49, 127.60, 127.57, 127.51, 126.81, 126.45, 126.06, 125.89, 125.73, 125.13, 124.91, 123.87, 123.51 (q, $J_{C\text{-}F}$ = 273.7 Hz), 123.34, 122.74 (m), 122.42, 119.43, 118.60, 113.81, 113.74, 56.67; ^{19}F NMR (376 MHz, $CDCl_3$): δ -62.64; HRMS (ESI-TOF) *m/z* Calcd. for $C_{41}H_{25}F_6N_2O_2^+$ $[M+H]^+$: 691.1815; Found: 691.1809.

(*R*)-2-((2'-methoxy-[1,1'-binaphthalen]-2-yl)oxy)-9-(naphthalen-1-yl)-1,10-phenanthroline (9d)

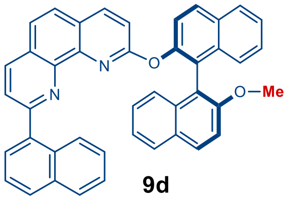

9d

White solid, 60 mg, Yield: 51%. m.p. 143–144 °C; $[\alpha]_D^{15}$ = +32.7 (*c* = 0.33, CH_2Cl_2); ^{1}H NMR (400 MHz, $CDCl_3$): δ 8.47 (d, *J* = 8.8 Hz, 1H), 8.20 (d, *J* = 8.0 Hz, 1H), 7.86 (dd, *J* = 15.2, 7.2 Hz, 4H), 7.81 (dd, *J* = 9.2, 5.2 Hz, 3H), 7.75 (dd, *J* = 7.2, 0.8 Hz, 1H), 7.71 (d, *J* = 8.8 Hz, 1H), 7.66 (d, *J* = 8.0 Hz, 1H), 7.61 (d, *J* = 8.8 Hz, 1H), 7.57 (d, *J* = 8.4 Hz, 1H), 7.50 – 7.41 (m, 2H), 7.33 – 7.26 (m, 3H), 7.26 – 7.22 (m, 2H), 7.06 (t, *J* = 7.6 Hz, 1H), 6.88 – 6.78 (m, 3H), 3.55 (s, 3H); ^{13}C NMR (101 MHz, $CDCl_3$): δ 162.99, 158.87, 155.03, 151.19, 145.17, 145.01, 138.96, 138.51, 136.08, 134.32, 134.03, 133.96, 131.55, 131.49, 129.76, 129.46, 129.25, 128.97, 128.81, 128.35, 128.06, 127.56, 127.51, 126.53, 126.43, 126.18, 126.10, 126.01, 125.87, 125.79, 125.56, 125.24, 125.04, 124.77, 124.57, 124.20, 123.40, 122.44, 118.49, 113.48, 113.34, 56.48; HRMS (ESI-TOF) *m/z* Calcd. for $C_{43}H_{29}N_2O_2^+$ $[M+H]^+$: 605.2224; Found: 605.2220.

(*R*)-2-((2'-methoxy-[1,1'-binaphthalen]-2-yl)oxy)-9-(naphthalen-2-yl)-1,10-phenanthroline (9e)

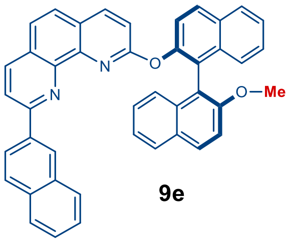

9e

White solid, 151 mg, Yield: 64%. m.p. 143–144 °C; $[\alpha]_D^{15}$ = -235.3 (*c* = 0.43, CH_2Cl_2); ^{1}H NMR (400 MHz, $CDCl_3$): δ 8.70 (s, 1H), 8.30 (d, *J* = 8.4 Hz, 1H), 8.21 (dd, *J* = 13.6, 8.8 Hz, 2H), 8.16 (d, *J* = 8.8 Hz, 1H), 8.10 (d, *J* = 8.0 Hz, 1H), 7.92 (d, *J* = 8.8 Hz, 1H), 7.87 – 7.79 (m, 3H), 7.74 (d, *J* = 8.8 Hz, 1H), 7.66 – 7.53 (m, 5H), 7.53 - 7.39 (m, 3H), 7.36 - 7.26 (m, 3H), 6.99 - 6.94 (m, 2H), 6.64 (t, *J* = 7.2 Hz, 1H), 3.68 (s, 3H); ^{13}C NMR (101 MHz, $CDCl_3$): δ 162.90, 155.61, 155.10, 150.89, 145.15, 144.68, 139.09, 136.59, 136.40, 134.54, 134.07, 134.01, 133.61, 131.71, 129.73, 129.34, 129.25, 129.00, 128.36, 128.30, 127.89, 127.64, 127.28, 127.11, 126.80, 126.60, 126.47, 126.40, 126.14, 125.80, 125.69, 125.62, 125.46, 125.20, 124.84, 123.94, 123.40, 123.23, 119.33, 118.75, 113.83, 112.95, 56.88; HRMS (ESI-TOF) *m/z* Calcd. for $C_{43}H_{29}N_2O_2^+$ $[M+H]^+$: 605.2224; Found: 605.2215.

For data of **9f**, see **L11** in the following part.

(*R*)-2-((2'-methoxy-[1,1'-binaphthalen]-2-yl)oxy)-9-(phenanthren-9-yl)-1,10-phenanthroline (9g)

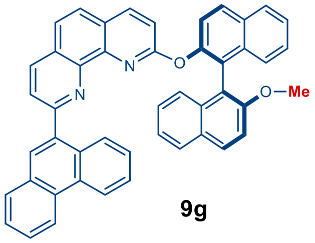

9g

Yellow solid, 158 mg, Yield: 62%. m.p. 128–129 °C; $[\alpha]_D^{15}$ = +42.4 (*c* = 0.57, CH_2Cl_2); ^{1}H NMR (400 MHz, $CDCl_3$): δ 8.72 (t, *J* = 9.2 Hz, 2H), 8.43 (d, *J* = 8.4 Hz, 1H), 8.27 (d, *J* = 8.4 Hz, 1H), 8.07 (s, 1H), 7.93 (dd, *J* = 8.4, 2.4 Hz, 2H), 7.89 (d, *J* = 8.4 Hz, 1H), 7.85 (d, *J* = 2.0 Hz, 1H), 7.83 – 7.79 (m, 2H), 7.73 (d, *J* = 8.8 Hz, 1H), 7.70 – 7.65 (m, 3H), 7.64 (s, 1H), 7.62 – 7.57 (m, 1H), 7.54 (t, *J* = 7.6 Hz, 1H), 7.48 (ddd, *J* = 8.0, 6.4, 1.2 Hz, 1H), 7.36 – 7.27 (m, 4H), 7.12 – 7.06 (m, 2H), 6.89 (d, *J* = 8.4 Hz 1H), 6.86 (t, *J* = 7.6 Hz 1H), 3.61 (s, 3H); ^{13}C NMR (101 MHz, $CDCl_3$): δ 163.07, 158.79, 155.02, 151.19, 145.31, 144.98, 139.04, 137.30, 136.01, 134.33, 133.94, 131.57, 131.52, 130.81, 130.70, 130.48 , 130.01, 129.79, 129.52, 129.42, 129.18, 128.96, 128.37, 127.70,

127.56, 127.11, 126.86, 126.75, 126.67, 126.47, 126.19, 126.00, 125.96, 125.57, 125.08, 124.87, 124.73, 124.24, 123.39, 122.75, 122.59, 122.40, 118.42, 113.46, 113.33, 56.51; HRMS (ESI-TOF) *m/z* Calcd. for $C_{47}H_{31}N_2O_2^+$ $[M+H]^+$: 655.2380; Found: 655.2397.

(*R*)-2-((2'-methoxy-[1,1'-binaphthalen]-2-yl)oxy)-9-(pyren-1-yl)-1,10-phenanthroline (9h)

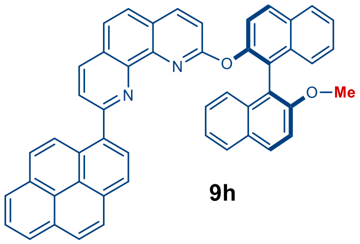

9h

Yellow solid, 120 mg, Yield: 45%. m.p. 113–114 °C; $[\alpha]_D^{15}$ = +81.3 (*c* = 0.32, CH_2Cl_2); ^{1}H NMR (400 MHz, $CDCl_3$): δ 8.87 (d, *J* = 9.2 Hz, 1H), 8.31 (d, *J* = 8.0 Hz, 1H), 8.26 (d, *J* = 8.0 Hz, 1H), 8.19 – 8.14 (m, 2H), 8.07 (dd, *J* = 12.8, 8.8 Hz, 2H), 8.00 – 7.97 (m, 3H), 7.87 (d, *J* = 8.4 Hz, 1H), 7.85 – 7.74 (m, 4H), 7.66 (d, *J* = 8.0 Hz, 1H), 7.64 (d, *J* = 8.8 Hz, 1H) 7.59 (d, *J* = 8.8 Hz, 1H), 7.48 (d, *J* = 9.6 Hz, 1H), 7.45 (ddd, *J* = 8.0, 6.4, 1.6 Hz, 1H), 7.33 (d, *J* = 8.4 Hz, 1H), 7.30 – 7.26 (m, 2H), 7.24 – 7.21 (m, 1H), 7.05 (t, *J* = 6.8 Hz, 1H), 6.85 (t, *J* = 7.6 Hz, 1H), 6.80 (d, *J* = 8.4 Hz, 1H), 3.56 (s, 3H); ^{13}C NMR (101 MHz, $CDCl_3$): δ 162.98, 159.13, 155.05, 152.32, 151.34, 145.30, 144.98, 138.96, 136.13, 135.61, 134.33, 133.94, 131.69, 131.50, 131.47, 131.01, 129.78, 129.48, 129.24, 128.97, 128.81, 128.38, 128.02, 127.56, 127.35, 126.45, 126.21, 126.02, 125.94, 125.86, 125.61, 125.42, 125.32, 125.24, 125.22, 125.20, 125.15, 125.07, 124.92, 124.80, 124.68, 124.20, 123.40, 122.47, 118.54, 113.48, 56.49; HRMS (ESI-TOF) *m/z* Calcd. for $C_{49}H_{31}N_2O_2^+$ $[M+H]^+$: 679.2380; Found: 679.2368.

(*R*)-2'-((1,10-phenanthrolin-2-yl)oxy)-[1,1'-binaphthalen]-2-ol (L1)

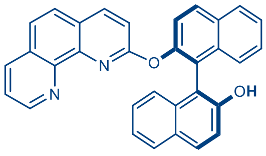

L1

White solid, 170 mg, Yield: 57%. m.p. 141–142 °C; $[\alpha]_D^{15}$ = +962.0 (*c* = 0.20, CH_2Cl_2); ^{1}H NMR (400 MHz, $CDCl_3$): δ 8.84 (dd, *J* = 4.4, 2.0 Hz, 1H), 8.26 (br, 1H), 8.09 (dd, *J* = 8.0, 1.6 Hz, 1H), 8.05 (d, *J* = 8.8 Hz, 1H), 7.97 (t, *J* = 8.4 Hz, 2H), 7.79 – 7.72 (m, 1H), 7.65 (d, *J* = 8.8 Hz, 1H), 7.61 (d, *J* = 8.8 Hz, 1H), 7.57 (d, *J* = 8.8 Hz, 1H), 7.50 – 7.41 (m, 3H), 7.37 – 7.31 (m, 2H), 7.31

– 7.25 (m, 3H), 7.01 (d, *J* = 8.4 Hz, 1H), 6.97 (d, *J* = 8.8 Hz, 1H); ^{13}C NMR (101 MHz, $CDCl_3$): δ 163.04, 153.21, 151.04, 149.72, 144.63, 143.83, 139.92, 136.04, 134.34, 134.12, 131.99, 130.79, 129.56, 129.13, 129.07, 128.23, 127.92, 127.13, 126.40, 125.25, 126.19, 125.94, 125.80, 125.30, 124.99, 124.52, 123.27, 122.99, 122.13, 120.59, 116.78, 113.77; HRMS (ESI-TOF) *m/z* Calcd. for $C_{32}H_{21}N_2O_2^+$ [M+H]$^+$: 465.1598; Found: 465.1597.

(*R*)-2'-((9-chloro-1,10-phenanthrolin-2-yl)oxy)-[1,1'-binaphthalen]-2-ol (L2)

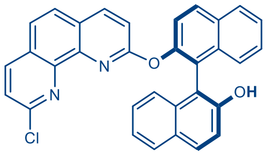

L2

White solid, 119 mg, Yield: 62%. m.p. 237–238 °C; $[\alpha]_D^{15}$ = +916.0 (*c* = 0.20, CH_2Cl_2); ^{1}H NMR (400 MHz, $CDCl_3$): δ 8.17 (dd, *J* = 8.4, 1.2 Hz, 1H), 8.10 – 7.94 (m, 3H), 7.79 – 7.76 (m, 2H), 7.75 (d, *J* = 4.0 Hz, 1H), 7.70 – 7.64 (m, 1H), 7.63 (d, *J* = 2.0 Hz, 1H), 7.59 (d, *J* = 8.8 Hz, 1H), 7.57 – 7.53 (m, 1H), 7.49 (ddd, *J* = 8.0, 5.6, 2.4 Hz, 1H), 7.43 (dd, *J* = 8.8, 6.4 Hz, 1H), 7.36 – 7.32 (m, 1H), 7.31 – 7.26 (m, 2H), 7.01 (dd, *J* = 8.8, 6.0 Hz, 2H); ^{13}C NMR (101 MHz, $CDCl_3$): δ 163.30, 153.05, 151.05, 150.95, 144.52, 142.83, 139.94, 138.62, 134.17, 134.14, 132.07, 131.05, 129.64, 129.00, 128.24, 127.94, 127.70, 127.18, 126.45, 126.32, 126.16, 125.87, 125.73, 125.55, 124.95, 124.26, 123.82, 123.25, 121.94, 120.07, 115.93, 114.37; HRMS (ESI-TOF) *m/z* Calcd. for $C_{32}H_{20}ClN_2O_2^+$ [M+H]$^+$: 499.1208; Found: 499.1216.

(*R*)-2'-((9-phenyl-1,10-phenanthrolin-2-yl)oxy)-[1,1'-binaphthalen]-2-ol (L3)

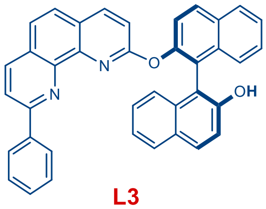

L3

White solid, 65 mg, Yield: 67%. m.p. 300–301 °C; $[\alpha]_D^{15}$ = +941.0 (*c* = 0.20, CH_2Cl_2); ^{1}H NMR (400 MHz, $CDCl_3$): δ 8.13 (d, *J* = 8.8 Hz, 1H), 8.09 (s, 1H), 8.06 (d, *J* = 4.0 Hz, 2H), 8.00 (d, *J* = 8.4 Hz, 1H), 7.93 (d, *J* = 8.4 Hz, 1H), 7.80 – 7.73 (m, 1H), 7.71 – 7.60 (m, 4H), 7.55 (dd, *J* = 10.8, 8.8 Hz, 2H), 7.46 (d, *J* = 8.8 Hz, 1H), 7.42 (d, *J* = 6.8 Hz, 1H), 7.36 (d, *J* = 8.4 Hz, 1H), 7.30 – 7.23 (m, 3H), 7.17 (t, *J* = 7.2 Hz, 1H), 7.04 (d, *J* = 8.8 Hz, 1H), 6.90 (d, *J* = 9.2 Hz, 1H), 6.85 (t, *J* = 7.6 Hz, 2H); ^{13}C NMR (101 MHz, $CDCl_3$): δ 163.35, 155.61, 153.26, 151.50, 144.17, 143.69,

140.11, 138.16, 136.62, 134.37, 134.31, 132.25, 131.11, 129.59, 129.35, 128.98, 128.57, 128.36, 127.95, 127.09, 126.88, 126.61, 126.49, 126.36, 125.88, 125.31, 124.84, 124.22, 123.26, 122.49, 120.23, 119.45, 115.90, 113.05; HRMS (ESI-TOF) *m/z* Calcd. for $C_{38}H_{25}N_2O_2^+$ $[M+H]^+$: 541.1911; Found: 541.1915.

(*R*)-2'-((9-(3,5-dimethylphenyl)-1,10-phenanthrolin-2-yl)oxy)-[1,1'-binaphthalen]-2-ol (L4)

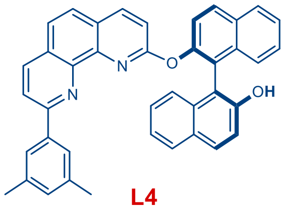

L4

White solid, 110 mg, Yield: 56%. m.p. 266–267 °C; $[\alpha]_D^{15}$ = +778.0 (*c* = 0.20, CH_2Cl_2); ^{1}H NMR (400 MHz, $CDCl_3$): δ 8.14 (d, *J* = 8.8 Hz, 1H), 8.11 (d, *J* = 9.2 Hz, 1H), 8.05 (s, 1H), 8.04 (d, *J* = 8.4 Hz, 1H), 7.98 (d, *J* = 8.4 Hz, 2H), 7.76 (dd, *J* = 7.6, 1.2 Hz, 1H), 7.65 (d, *J* = 9.2 Hz, 1H), 7.61 (s, 2H), 7.55 – 7.45 (m, 4H), 7.33 – 7.25 (m, 3H), 7.20 (d, *J* = 7.6 Hz, 1H), 7.04 (d, *J* = 8.4 Hz, 1H), 6.94 (d, *J* = 8.8 Hz, 1H), 6.84 (s, 1H), 1.92 (s, 6H); ^{13}C NMR (101 MHz, $CDCl_3$): δ 163.18, 156.54, 153.31, 151.05, 144.41, 144.03, 140.29, 138.61, 138.16, 136.58, 134.36, 134.23, 132.07, 131.01, 130.92, 129.48, 129.03, 128.38, 128.06, 127.92, 127.12, 126.41, 126.30, 125.96, 125.85, 125.62, 125.35, 125.14, 125.02, 124.47, 123.24, 122.38, 120.59, 120.13, 116.41, 113.45, 21.25; HRMS (ESI-TOF) *m/z* Calcd. for $C_{40}H_{29}N_2O_2^+$ $[M+H]^+$: 569.2224; Found: 569.2222.

(*R*)-2'-((9-(3,5-bis(trifluoromethyl)phenyl)-1,10-phenanthrolin-2-yl)oxy)-[1,1'-binaphthalen]-2-ol (L5)

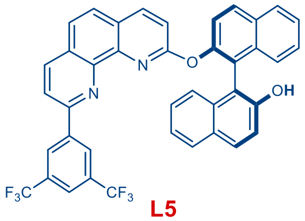

L5

White solid, 126 mg, Yield: 64%. m.p. 116–117 °C; $[\alpha]_D^{15}$ = +222.0 (*c* = 0.50, CH_2Cl_2); ^{1}H NMR (400 MHz, $CDCl_3$): δ 8.43 (s, 1H), 8.41 (s, 2H), 8.09 (d, *J* = 8.4 Hz, 1H), 8.00 (d, *J* = 8.8 Hz, 2H), 7.89 (d, *J* = 8.0 Hz, 1H), 7.84 (d, *J* = 8.4 Hz, 1H), 7.81 (d, *J* = 7.6 Hz, 1H), 7.74 (d, *J* = 8.8 Hz, 1H), 7.66 (d, *J* = 8.8 Hz, 1H), 7.62 (s, 1H), 7.55 (d, *J* = 8.8 Hz, 1H), 7.47 – 7.44 (m, 1H), 7.38 (d, *J* = 8.8 Hz, 1H), 7.35 – 7.30 (m, 2H), 7.27 (dd, *J* = 7.6, 0.8 Hz, 1H), 7.21 (d, *J* = 8.8 Hz, 1H), 7.15 (d, *J* = 8.0 Hz, 1H), 7.14 (d, *J* = 8.8 Hz, 1H), 7.07 (d, *J* = 9.2 Hz, 1H); ^{13}C NMR (101 MHz, $CDCl_3$):

δ 163.74, 153.87, 153.28, 151.24, 144.61, 143.98, 141.30, 140.56, 137.44, 134.37, 134.28, 131.98, 131.73 (q, J_{C-F} = 33.3 Hz), 131.02, 129.66, 129.11, 128.54, 128.17, 128.00, 127.83 (m), 127.19, 126.59, 126.50, 126.33, 126.05, 125.85, 125.61, 125.14, 124.48, 123.33, 123.21 (q, J_{C-F} = 274.0 Hz), 122.44 (m), 121.67, 120.57, 120.34, 116.22, 114.59; ^{19}F NMR (376 MHz, $CDCl_3$): δ -62.90; HRMS (ESI-TOF) *m/z* Calcd. for $C_{40}H_{23}F_6N_2O_2^+$ [M+H]$^+$: 677.1658; Found: 677.1652.

(*R*)-2'-((9-(naphthalen-1-yl)-1,10-phenanthrolin-2-yl)oxy)-[1,1'-binaphthalen]-2-ol (L6)

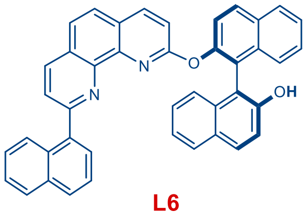

L6

White solid, 63 mg, Yield: 44%. m.p. 313–314 °C; $[\alpha]_D^{15}$ = +928.0 (*c* = 0.20, CH_2Cl_2); ^{1}H NMR (400 MHz, $CDCl_3$): δ 8.17 (d, *J* = 8.4 Hz, 1H), 8.06 (d, *J* = 8.4 Hz, 1H), 7.91 (d, *J* = 8.8 Hz, 1H), 7.87 (d, *J* = 8.4 Hz, 1H), 7.83 (d, *J* = 8.0 Hz, 1H), 7.76 (d, *J* = 8.4 Hz, 1H), 7.73 – 7.66 (m, 4H), 7.62 (d, *J* = 9.2 Hz, 1H), 7.55 – 7.48 (m, 3H), 7.45 (td, *J* = 7.6, 0.8 Hz, 1H), 7.38 (d, *J* = 8.8 Hz, 1H), 7.28 – 7.24 (m, 1H), 7.24 – 7.22 (m, 2H), 7.22 – 7.19 (m, 3H), 7.05 (t, *J* = 7.6 Hz, 1H), 6.99 (t, *J* = 7.6 Hz, 1H), 6.89 (dd, *J* = 8.4, 6.0 Hz, 2H); ^{13}C NMR (101 MHz, $CDCl_3$): δ 163.23, 158.20, 153.11, 151.31, 144.65, 143.97, 140.02, 137.83, 135.84, 134.23, 134.13, 133.83, 132.03, 130.87, 130.82, 129.55, 129.04, 128.90, 128.21, 128.14, 127.91, 127.67, 126.84, 126.40, 126.23, 126.04, 125.70, 125.61, 125.59, 125.45, 125.38, 125.35, 124.99, 124.89, 124.32, 123.15, 122.24, 119.91, 115.68, 113.34; HRMS (ESI-TOF) *m/z* Calcd. for $C_{42}H_{27}N_2O_2^+$ [M+H]$^+$: 591.2067; Found: 591.2070.

(*R*)-2'-((9-(naphthalen-2-yl)-1,10-phenanthrolin-2-yl)oxy)-[1,1'-binaphthalen]-2-ol (L7)

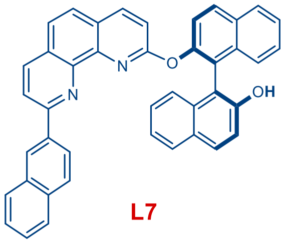

L7

White solid, 181 mg, Yield: 51%. m.p. 266–267 °C; $[\alpha]_D^{15}$ = +661.0 (*c* = 0.20, CH_2Cl_2); ^{1}H NMR (400 MHz, $CDCl_3$): δ 8.30 (s, 1H), 8.25 (s, 1H), 8.11 (dd, *J* = 10.4, 9.2 Hz, 2H), 7.97 (s, 2H), 7.94 (d, *J* = 8.4 Hz, 1H), 7.77 – 7.67 (m, 2H), 7.71 – 7.67 (m, 1H), 7.65 (d, *J* = 8.8 Hz, 2H), 7.48 – 7.43

(m, 4H), 7.40 – 7.36 (m, 2H), 7.30 – 7.28 (m, 3H), 7.21 (s, 1H), 7.15 (d, *J* = 8.8 Hz, 1H), 7.11 (d, *J* = 8.0 Hz, 1H), 7.01 (d, *J* = 8.8 Hz, 1H), 6.94 (d, *J* = 9.2 Hz, 1H); ^{13}C NMR (101 MHz, $CDCl_3$): δ 163.31, 155.55, 153.32, 151.44, 144.21, 143.73, 140.23, 136.61, 135.71, 134.43, 134.38, 133.90, 133.35, 132.26, 131.15, 129.63, 129.17, 129.04, 128.54, 128.19, 127.99, 127.98, 127.46, 127.21, 126.82, 126.55, 126.52, 125.90, 125.43, 125.31, 124.93, 124.38, 124.27, 123.31, 122.52, 120.41, 119.72, 116.21, 113.19; HRMS (ESI-TOF) *m/z* Calcd. for $C_{42}H_{27}N_2O_2^+$ [M+H]$^+$: 591.2067; Found: 591.2069.

(*R*)-2'-((9-(anthracen-9-yl)-1,10-phenanthrolin-2-yl)oxy)-[1,1'-binaphthalen]-2-ol (L8)

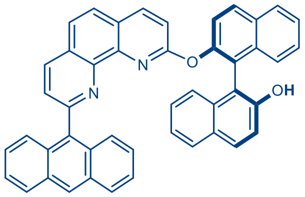

L8

White solid, 110 mg, Yield: 55%. m.p. 265–266 °C; $[\alpha]_D^{15}$ = +848.0 (*c* = 0.20, CH_2Cl_2); ^{1}H NMR (400 MHz, $CDCl_3$): δ 8.27 (s, 1H), 8.21 (d, *J* = 8.0 Hz, 1H), 7.88 (d, *J* = 8.8 Hz, 1H), 7.81 (d, *J* = 8.4 Hz, 2H), 7.77 – 7.65 (m, 4H), 7.64 – 7.55 (m, 4H), 7.52 (d, *J* = 8.4 Hz, 1H), 7.35 (d, *J* = 8.8 Hz, 1H), 7.31 – 7.23 (m, 2H), 7.22 – 7.10 (m, 4H), 7.08 (d, *J* = 7.2 Hz, 1H), 7.04 (d, *J* = 6.8 Hz, 1H), 6.98 (t, *J* = 7.2 Hz, 1H), 6.91 (d, *J* = 8.4 Hz, 1H), 6.84 (dd, *J* = 8.4, 5.2 Hz, 2H); ^{13}C NMR (101 MHz, $CDCl_3$): δ 163.11, 157.67, 153.09, 151.45, 144.85, 144.11, 139.80, 135.71, 134.80, 134.15, 133.86, 131.51, 131.30, 131.23, 130.40, 130.02, 129.95, 129.45, 128.73, 128.29, 128.21, 127.93, 127.87, 127.75, 127.17, 126.49, 126.42, 126.24, 126.21, 126.11, 125.72, 125.64, 125.53, 125.22, 125.14, 124.90, 124.85, 124.35, 124.26, 122.95, 122.04, 119.62, 115.17, 113.70; HRMS (ESI-TOF) *m/z* Calcd. for $C_{46}H_{29}N_2O_2^+$ [M+H]$^+$: 641.2224; Found: 641.2215.

(*R*)-2'-((9-(phenanthren-9-yl)-1,10-phenanthrolin-2-yl)oxy)-[1,1'-binaphthalen]-2-ol (L9)

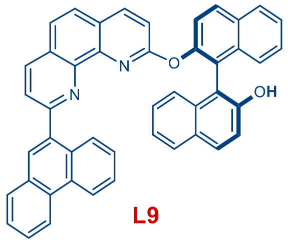

L9

Yellow solid, 65 mg, Yield: 47%. m.p. 175–176 °C; $[\alpha]_D^{15}$ = +725.0 (*c* = 1.00, CH_2Cl_2); ^{1}H NMR (400 MHz, $CDCl_3$): δ 8.57 (t, *J* = 8.8 Hz, 2H), 8.19 (d, *J* = 8.0 Hz, 1H), 8.15 (d, *J* = 8.0 Hz, 1H),

7.97 (d, *J* = 8.8 Hz, 1H), 7.93 (s, 1H), 7.92 (d, *J* = 8.4 Hz, 1H), 7.86 (d, *J* = 8.4 Hz, 1H), 7.80 (br, 1H), 7.75 (t, *J* = 8.0 Hz, 2H), 7.65 (d, *J* = 8.8 Hz, 1H), 7.62 – 7.55 (m, 3H), 7.50 – 7.36 (m, 4H), 7.32 (ddd, *J* = 8.0, 6.0, 2.4 Hz, 1H), 7.26 (td, *J* = 6.4, 1.2 Hz, 1H), 7.23 (s, 1H), 7.21 - 7.14 (m, 4H), 6.97 (d, *J* = 8.8 Hz, 1H), 6.92 (d, *J* = 9.2 Hz, 1H); ^{13}C NMR (101 MHz, $CDCl_3$): δ 163.16, 158.15, 153.21, 151.15, 144.63, 143.91, 140.07, 136.48, 135.86, 134.23, 134.12, 131.84, 131.43, 130.76, 130.64, 130.45, 130.42, 129.75, 129.56, 128.91, 128.14, 127.92, 127.79, 127.04, 126.94, 126.51, 126.39, 126.30, 126.14, 125.90, 125.77, 125.60, 125.54, 125.40, 125.02, 124.93, 124.30, 123.15, 122.70, 122.24, 122.15, 120.02, 115.83, 113.50; HRMS (ESI-TOF) *m/z* Calcd. for $C_{46}H_{29}N_2O_2^+$ $[M+H]^+$: 641.2224; Found: 641.2224.

(*R*)-2'-((9-(pyren-1-yl)-1,10-phenanthrolin-2-yl)oxy)-[1,1'-binaphthalen]-2-ol (L10)

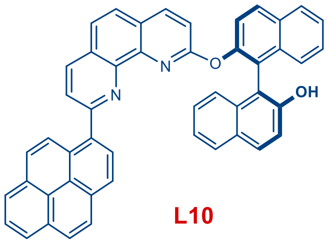

L10

White solid, 59 mg, Yield: 61%. m.p. 253–254 °C; $[\alpha]_D^{15}$ = +794.0 (*c* = 0.20, CH_2Cl_2); ^{1}H NMR (400 MHz, $CDCl_3$): δ 8.46 (d, *J* = 9.2 Hz, 1H), 8.29 (d, *J* = 8.4 Hz, 1H), 8.19 (d, *J* = 7.2 Hz, 1H), 8.13 – 8.06 (m, 3H), 8.05 – 7.96 (m, 5H), 7.79 - 7.70 (m, 7H), 7.64 (d, *J* = 8.8 Hz, 1H), 7.47 (d, *J* = 8.8 Hz, 1H), 7.30 – 7.23 (m, 2H), 7.18 – 7.16 (m, 2H), 7.20 – 7.15 (m, 2H), 7.06 (d, *J* = 8.4 Hz, 1H), 6.88 (d, *J* = 8.8 Hz, 1H); ^{13}C NMR (101 MHz, $CDCl_3$): δ 163.30, 158.52, 153.14, 151.36, 144.84, 143.98, 140.02, 135.93, 135.35, 134.23, 134.07, 131.92, 131.49, 131.46, 130.89, 130.84, 129.55, 128.88, 128.68, 128.47, 128.08, 127.90, 127.85, 127.56, 126.79, 126.39, 125.93, 125.83, 125.81, 125.67, 125.63, 125.50, 125.47, 125.31, 125.21, 124.97, 124.94, 124.87, 124.80, 124.32, 123.12, 122.24, 119.89, 115.57, 113.42; HRMS (ESI-TOF) *m/z* Calcd. for $C_{48}H_{29}N_2O_2^+$ $[M+H]^+$: 665.2224; Found: 665.2208.

(*R*)-2-(anthracen-9-yl)-9-((2'-methoxy-[1,1'-binaphthalen]-2-yl)oxy)-1,10-phenanthroline (L11)

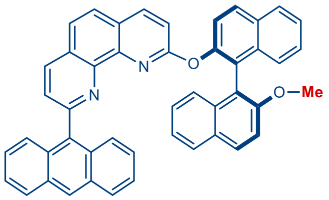

L11

Yellow solid, 99 mg, Yield: 52%. m.p. 160–161 ℃; $[\alpha]_D^{15}$ = +82.7 (*c* = 0.66, CH_2Cl_2); ^{1}H NMR (400 MHz, $CDCl_3$): δ 8.51 (s, 1H), 8.32 (dd, *J* = 8.0, 3.2 Hz, 1H), 8.02 (t, *J* = 6.8 Hz, 2H), 7.94 (dd, *J* = 8.8, 1.6 Hz, 1H), 7.85 (d, *J* = 8.4 Hz, 1H), 7.82 – 7.65 (m, 8H), 7.60 (dd, *J* = 8.8, 3.2 Hz, 1H), 7.44 – 7.35 (m, 3H), 7.25 – 7.24 (m, 3H), 7.23 – 7.18 (m, 2H), 7.13 – 7.07 (m, 2H), 6.94 (d, *J* = 8.4 Hz, 2H), 3.60 (s, 3H); ^{13}C NMR (101 MHz, $CDCl_3$): δ 163.10, 158.18, 154.94, 151.34, 145.62, 145.14, 139.02, 135.74, 134.24, 133.88, 131.45, 131.29, 130.46, 129.77, 129.55, 128.89, 128.37, 128.28, 128.23, 127.76, 127.68, 127.66, 126.72, 126.63, 126.52, 126.46, 126.17, 126.09, 125.72, 125.68, 125.65, 125.61, 125.07, 125.00, 124.61, 124.42, 123.36, 121.82, 118.08, 113.47, 113.26, 56.29; HRMS (ESI-TOF) *m/z* Calcd. for $C_{47}H_{31}N_2O_2^+$ $[M+H]^+$: 655.2380; Found: 655.2369.

(*R*)-2-(anthracen-9-yl)-9-((2'-((tert-butyldimethylsilyl)oxy)-[1,1'-binaphthalen]-2-yl)oxy)-1,10-phenanthroline (L12)

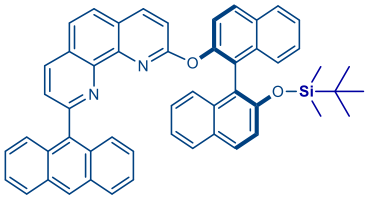

L12

Yellow solid, 108.2 mg, Yield: 86%; m.p. 138–140 °C; $[\alpha]_D^{20}$ = -80.8 (*c* = 0.25, $CHCl_3$); ^{1}H NMR (400 MHz, $CDCl_3$): δ 8.53 (s, 1H), 8.34 (d, *J* = 8.4 Hz, 1H), 8.05 (d, *J* = 8.4 Hz, 1H), 8.01 (d, *J* = 8.8 Hz, 1H), 7.91 (d, *J* = 8.8 Hz, 1H), 7.81 – 7.72 (m, 6H), 7.68 – 7.62 (m, 4H), 7.47 – 7.38 (m, 2H), 7.35 – 7.27 (m, 2H), 7.24 – 7.22 (m, 3H), 7.14 (d, *J* = 8.8 Hz, 1H), 7.01 (t, *J* = 7.4 Hz, 1H), 6.93 (d, *J* = 8.8 Hz, 1H), 6.89 (d, *J* = 8.0 Hz, 1H), 6.73 (t, *J* = 7.6 Hz, 1H), 0.47 (s, 9H), 0.04 (s, 3H), -0.29 (s, 3H); ^{13}C NMR (101 MHz, $CDCl_3$): δ 163.12, 157.94, 151.64, 151.35, 145.65, 145.03, 138.90, 135.70, 135.64, 134.25, 134.16, 131.52, 131.46, 131.33, 130.47, 130.38, 129.26, 129.22, 129.01, 128.52, 128.16, 128.12, 127.78, 127.68, 127.36, 126.80, 126.68, 126.54, 126.35, 126.23, 126.19, 126.11, 125.70, 125.67, 125.61, 125.56, 125.19, 125.00, 124.80, 124.79, 124.30, 123.38,

122.27, 121.01, 120.60, 114.02, 25.24, 17.75, -3.96, -4.58; HRMS (ESI-TOF) *m/z* Calcd. for $C_{52}H_{43}N_2O_2Si^+$ [M+H]$^+$: 755.3088; Found: 755.3089.

(*R*)-2-(anthracen-9-yl)-9-((2'-((triisopropylsilyl)oxy)-[1,1'-binaphthalen]-2-yl)oxy)-1,10-phenanthroline (L13)

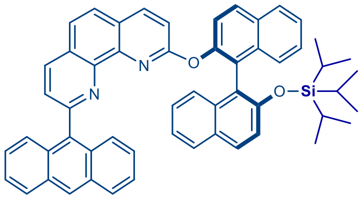

L13

Yellow solid, 109 mg, Yield: 82%; m.p. 230–231 °C; $[\alpha]_D^{20}$ = +10.2 (*c* = 1.00, $CHCl_3$); ^1H NMR (400 MHz, $CDCl_3$): δ 8.53 (s, 1H), 8.33 (d, *J* = 8.0 Hz, 1H), 8.06 (d, *J* = 8.4 Hz, 1H), 8.01 (d, *J* = 8.4 Hz, 1H), 7.89 - 7.70 (m, 8H), 7.63 (dd, *J* = 8.8, 2.0 Hz, 2H), 7.59 (d, *J* = 8.0 Hz, 1H), 7.46 (t, *J* = 7.4 Hz, 1H), 7.40 (ddd, *J* = 8.0, 6.0, 1.6 Hz, 1H), 7.31 (t, *J* = 7.4 Hz, 2H), 7.27 - 7.21 (m, 3H), 7.16 (d, *J* = 8.8 Hz, 1H), 6.95 (t, *J* = 7.2 Hz, 1H), 6.84 (d, *J* = 8.4 Hz, 1H), 6.80 (t, *J* = 7.6 Hz, 1H), 6.63 (t, *J* = 7.2 Hz, 1H), 1.02 (sept, *J* = 7.6 Hz, 3H), 0.77 (d, *J* = 7.6 Hz, 9H), 0.73 (d, *J* = 7.6 Hz, 9H); ^{13}C NMR (101 MHz, $CDCl_3$): δ 163.09, 157.78, 151.67, 151.46, 145.60, 144.96, 138.84, 135.63, 135.57, 134.21, 134.19, 131.56, 131.49, 131.41, 130.46, 130.36, 129.14, 129.07, 128.64, 128.55, 128.11, 128.06, 127.84, 127.61, 127.21, 126.83, 126.69, 126.53, 126.40, 126.26, 126.17, 125.91, 125.65, 125.60, 125.57, 125.38, 125.22, 124.98, 124.96, 124.68, 124.16, 123.05, 122.39, 120.12, 119.98, 113.97, 17.86, 17.83, 12.90; HRMS (ESI-TOF) *m/z* Calcd. for $C_{55}H_{49}N_2O_2Si^+$ [M+H]$^+$: 797.3558; Found: 797.3560.

(*R*)-2-(anthracen-9-yl)-9-((2'-((tert-butyldiphenylsilyl)oxy)-[1,1'-binaphthalen]-2-yl)oxy)-1,10-phenanthroline (L14)

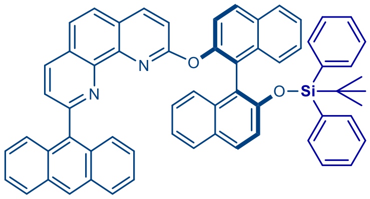

L14

Yellow solid, 121 mg, Yield: 92%; m.p. 149–150 °C; $[\alpha]_D^{20}$ = -73.4 (*c* = 1.00, $CHCl_3$); ^1H NMR (400 MHz, $CDCl_3$): δ 8.56 (s, 1H), 8.34 (d, *J* = 8.4 Hz, 1H), 8.09 (d, *J* = 8.4 Hz, 1H), 8.03 (d, *J* = 8.4 Hz, 1H), 7.89 (d, *J* = 8.4 Hz, 1H), 7.85 – 7.78 (m, 5H), 7.73 (d, *J* = 7.2 Hz, 1H), 7.71 (d, *J* =

7.6 Hz, 1H), 7.66 – 7.60 (m, 5H), 7.55 (d, *J* = 8.0 Hz, 1H), 7.49 – 7.42 (m, 4H), 7.38 – 7.33 (m, 4H), 7.30 – 7.25 (m, 5H), 7.11 (t, *J* = 7.4 Hz, 1H), 7.03 – 6.93 (m, 3H), 6.81 – 6.77 (m, 2H), 0.50 (s, 9H); ^{13}C NMR (101 MHz, $CDCl_3$): δ 163.20, 158.06, 151.43, 150.80, 145.70, 145.22, 139.18, 135.78, 135.70, 135.55, 135.51, 134.38, 134.07, 132.78, 132.73, 131.52, 131.48, 130.46, 130.40, 129.92, 129.84, 129.41, 128.91, 128.80, 128.49, 128.19, 128.16, 127.81, 127.77, 127.40, 126.81, 126.69, 126.61, 126.35, 126.16, 126.07, 125.74, 125.66, 125.60, 125.33, 125.22, 125.07, 125.02, 124.34, 123.35, 122.25, 120.21, 120.17, 113.52, 25.86, 18.97; HRMS (ESI-TOF) *m/z* Calcd. for $C_{62}H_{47}N_2O_2Si^+$ $[M+H]^+$: 879.3401; Found: 879.3401.

Compounds 3

(*R*)-benzyl 2-(2'-(benzylamino)-[1,1'-binaphthalen]-2-yl)hydrazine-1-carboxylate (3a)

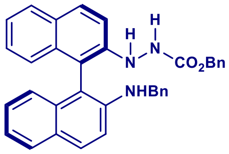

3a

Pale yellow solid, 47 mg. Yield: 91%; e.r. 97.5:2.5; m.p. 78–79 °C; $[\alpha]_D^{15}$ = +56.2 (*c* = 0.80, CH_2Cl_2); ^{1}H NMR (400 MHz, $CDCl_3$): δ 7.88 (d, *J* = 8.8 Hz, 1H), 7.82 (d, *J* = 7.6 Hz, 1H), 7.75 (d, *J* = 9.2 Hz, 1H), 7.69 (d, *J* = 9.2 Hz, 1H), 7.38 (d, *J* = 8.8 Hz, 1H), 7.35 – 7.18 (m, 7H), 7.17 – 7.07 (m, 8H), 6.98 – 6.80 (m, 2H), 6.53 (br, 1H), 5.70 (s, 1H), 5.04 (s, 2H), 4.49 (br, 1H), 4.36 (s, 1H), 4.08 (br, 1H); ^{13}C NMR (101 MHz, $CDCl_3$): δ 156.71, 144.28, 144.20, 139.99, 135.89, 133.91, 133.51, 130.05, 129.96, 129.81, 128.62, 128.49, 128.31, 128.17, 127.62, 127.03, 126.88, 124.64, 123.82, 123.63, 122.10, 114.31, 114.24, 110.52, 67.51, 47.62; HRMS (ESI-TOF) *m/z* Calcd. for $C_{35}H_{30}N_3O_2^+$ $[M+H]^+$: 524.2333; Found: 524.2323.

(*R*)-phenyl 2-(2'-(benzylamino)-[1,1'-binaphthalen]-2-yl)hydrazine-1-carboxylate (3b)

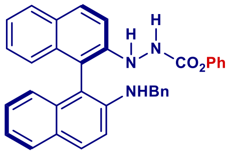

3b

White solid, 48 mg. Yield: 94%; e.r. 88.5:11.5; m.p. 88–89 °C; $[\alpha]_D^{15}$ = +36.5 (*c* = 0.42, CH_2Cl_2); ^{1}H NMR (400 MHz, $CDCl_3$): δ 7.94 (d, *J* = 7.6 Hz, 1H), 7.84 (d, *J* = 7.2 Hz, 1H), 7.78 (d, *J* = 8.4 Hz, 1H), 7.72 (d, *J* = 7.6 Hz, 1H), 7.50 – 7.48 (m, 1H), 7.31 – 7.25 (m, 4H), 7.20 – 7.04 (m, 11H), 6.97 (s, 1H), 6.81 (s, 1H), 6.56 (br, 1H), 5.80 (s, 1H), 4.44 (br, 1H), 4.34 (s, 1H), 4.17 (br, 1H);

^{13}C NMR (101 MHz, $CDCl_3$): δ 154.88, 150.67, 144.29, 143.94, 140.00, 133.95, 133.50, 130.11, 129.95, 129.45, 128.50, 128.37, 128.19, 127.63, 127.10, 127.01, 126.95, 126.92, 125.77, 124.70, 123.80, 122.15, 121.42, 114.33, 110.39, 47.67; HRMS (ESI-TOF) *m/z* Calcd. for $C_{34}H_{28}N_3O_2^+$ $[M+H]^+$: 510.2176; Found: 510.2172.

(*R*)-propyl 2-(2'-(benzylamino)-[1,1'-binaphthalen]-2-yl)hydrazine-1-carboxylate (3c)

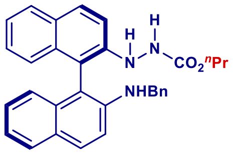

3c

Pale yellow solid, 36 mg. Yield: 76%; e.r. 97:3; m.p. 78–79 °C; $[\alpha]_D^{15}$ = +56.8 (*c* = 0.50, CH_2Cl_2); ^1H NMR (400 MHz, $CDCl_3$): δ 7.92 (d, *J* = 8.8 Hz, 1H), 7.83 (d, *J* = 8.0 Hz, 1H), 7.78 (d, *J* = 9.2 Hz, 1H), 7.75 – 7.69 (m, 1H), 7.42 (d, *J* = 8.8 Hz, 1H), 7.33 – 7.27 (m, 1H), 7.25 – 7.22 (m, 1H), 7.21 – 7.18 (m, 4H), 7.18 – 7.08 (m, 5H), 7.01 – 6.94 (m, 1H), 6.43 (br, 1H), 5.69 (s, 1H), 4.49 (br, 2H), 4.40 (s, 1H), 3.99 (m, 2H), 1.60 (m, 2H), 0.88 (m, 3H); ^{13}C NMR (101 MHz, $CDCl_3$): δ 157.06, 144.39, 144.27, 140.03, 133.93, 133.53, 130.04, 129.97, 129.80, 128.54, 128.33, 128.19, 127.67, 127.01, 126.95, 126.90, 124.64, 123.87, 123.59, 122.13, 114.37, 114.17, 110.68, 67.49, 47.76, 22.35, 10.30; HRMS (ESI-TOF) *m/z* Calcd. for $C_{31}H_{30}N_3O_2^+$ $[M+H]^+$: 476.2333; Found: 476.2340.

(*R*)-benzyl 2-(2'-(benzylamino)-6-bromo-[1,1'-binaphthalen]-2-yl)hydrazine-1-carboxylate (3d)

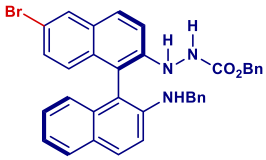

3d

Pale yellow solid, 48 mg. Yield: 80%; e.r. 96.5:3.5; m.p. 80–81 °C; $[\alpha]_D^{15}$ = +38.5 (*c* = 0.52, CH_2Cl_2); ^1H NMR (400 MHz, $CDCl_3$): δ 7.99 (s, 1H), 7.82 (d, *J* = 8.8 Hz, 1H), 7.78 (d, *J* = 9.2 Hz, 1H), 7.72 (d, *J* = 9.2 Hz, 1H), 7.43 (d, *J* = 9.2 Hz, 1H), 7.32 (br, 3H), 7.28 (dd, *J* = 8.8, 2.0 Hz, 2H), 7.24 – 7.13 (m, 7H), 7.10 (d, *J* = 8.8 Hz, 2H), 6.96 (d, *J* = 8.8 Hz, 1H), 6.90 (br, 1H), 6.51 (br, 1H), 5.73 (s, 1H), 5.08 (s, 2H), 4.40 (br, 2H), 4.11 (br, 1H); ^{13}C NMR (101 MHz, $CDCl_3$): δ 156.66, 144.65, 144.21, 139.86, 135.79, 134.30, 133.81, 132.10, 130.90, 130.33, 130.24, 129.05, 128.68, 128.58, 128.27, 127.64, 127.08, 127.01, 126.89, 126.54, 123.57, 122.26, 117.34, 115.23,

114.36, 109.77, 67.66, 47.71; HRMS (ESI-TOF) *m/z* Calcd. for $C_{35}H_{29}BrN_3O_2^+$ $[M+H]^+$: 602.1438; Found: 602.1446.

(*R*)-benzyl 2-(2'-(benzylamino)-6-methyl-[1,1'-binaphthalen]-2-yl)hydrazine-1-carboxylate (3e)

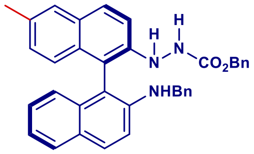

3e

Pale yellow solid, 40 mg. Yield: 75%; e.r. 97:3; m.p. 75–76 °C; $[\alpha]_D^{15}$ = +40.9 (*c* = 0.43, CH_2Cl_2); ^1H NMR (400 MHz, $CDCl_3$): δ 7.84 (d, *J* = 8.8 Hz, 1H), 7.77 (dd, *J* = 8.8, 2.0 Hz, 1H), 7.72 (d, *J* = 6.8 Hz, 1H), 7.62 (s, 1H), 7.38 (d, *J* = 8.8 Hz, 1H), 7.39 – 7.25 (m, 4H), 7.23 – 7.12 (m, 7H), 7.10 – 7.07 (m, 3H), 7.02 (dd, *J* = 8.0, 3.2 Hz, 1H), 6.96 (s, 1H), 6.51 (br, 1H), 5.67 (s, 1H), 5.07 (s, 2H), 4.51 (br, 1H), 4.40 (s, 1H), 4.11 (br, 1H), 2.46 (s, 3H); ^{13}C NMR (101 MHz, $CDCl_3$): δ 156.78, 144.17, 143.56, 140.03, 135.94, 133.94, 133.14, 131.68, 130.11, 130.00, 129.32, 128.66, 128.52, 128.17, 127.63, 127.40, 126.90, 124.59, 123.86, 122.12, 114.35, 110.78, 67.53, 47.74, 21.52; HRMS (ESI-TOF) *m/z* Calcd. for $C_{36}H_{32}N_3O_2^+$ $[M+H]^+$: 538.2489; Found: 538.2493.

(*R*)-benzyl 2-(2'-(benzylamino)-6-(methoxycarbonyl)-[1,1'-binaphthalen]-2-yl)hydrazine-1-carboxylate (3f)

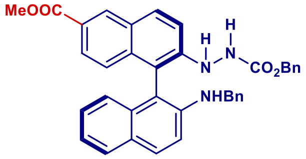

3f

Pale yellow solid, 57 mg. Yield: 98%; e.r. 97.5:2.5; m.p. 93–94 °C; $[\alpha]_D^{15}$ = +49.2 (*c* = 0.50, CH_2Cl_2); ^1H NMR (400 MHz, $CDCl_3$): δ 8.60 (s, 1H), 8.00 (d, *J* = 8.8 Hz, 1H), 7.79 (dd, *J* = 8.0, 0.8 Hz, 1H), 7.78 (d, *J* = 8.8 Hz, 1H), 7.71 (d, *J* = 8.8 Hz, 1H), 7.44 (d, *J* = 9.2 Hz, 1H), 7.38 – 7.24 (m, 3H), 7.23 (s, 1H), 7.22 – 7.01 (m, 10H), 6.90 (s, 1H), 6.59 (br, 1H), 5.82 (s, 1H), 5.07 (s, 2H), 4.44 (br, 1H), 4.39 (s, 1H), 4.11 (br, 1H), 3.93 (s, 3H); ^{13}C NMR (101 MHz, $CDCl_3$): δ 167.55, 156.62, 146.48, 144.20, 139.83, 136.05, 135.74, 133.75, 131.65, 131.54, 130.36, 128.66, 128.56, 128.28, 127.66, 127.06, 126.99, 126.86, 126.50, 125.04, 124.69, 123.53, 122.25, 114.67, 114.36, 109.71, 67.70, 52.12, 47.63; HRMS (ESI-TOF) *m/z* Calcd. for $C_{37}H_{32}N_3O_4^+$ $[M+H]^+$: 582.2387; Found: 582.2389.

(*R*)-benzyl 2-(2'-(benzylamino)-7-bromo-[1,1'-binaphthalen]-2-yl)hydrazine-1-carboxylate (3g)

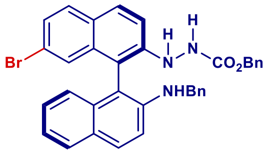

3g

Pale yellow solid, 57 mg. Yield: 94%; e.r. 97:3; m.p. 85–86 °C; $[\alpha]_D^{15}$ = +71.6 (*c* = 0.50, CH_2Cl_2); ^1H NMR (400 MHz, $CDCl_3$): δ 7.86 (d, *J* = 8.8 Hz, 1H), 7.77 (d, *J* = 8.8 Hz, 1H), 7.73 – 7.68 (m, 2H), 7.39 (dd, *J* = 8.4, 5.6 Hz, 2H), 7.35 – 7.28 (m, 3H), 7.25 – 7.14 (m, 8H), 7.08 (d, *J* = 8.8 Hz, 2H), 6.94 (s, 2H), 6.51 (br, 1H), 5.71 (s, 1H), 5.06 (s, 2H), 4.40 (s, 1H), 4.03 (br, 2H); ^{13}C NMR (101 MHz, $CDCl_3$): δ 156.64, 145.23, 144.11, 139.80, 135.78, 134.76, 133.67, 130.44, 130.04, 129.90, 128.70, 128.52, 128.45, 128.32, 128.17, 127.65, 127.06, 126.98, 126.82, 126.57, 123.48, 122.22, 121.86, 114.50, 114.29, 109.40, 67.62, 47.38; HRMS (ESI-TOF) *m/z* Calcd. for $C_{35}H_{29}BrN_3O_2^+$ $[M+H]^+$: 602.1438; Found: 602.1433.

(*R*)-benzyl 2-(2'-(benzylamino)-7-methyl-[1,1'-binaphthalen]-2-yl)hydrazine-1-carboxylate (3h)

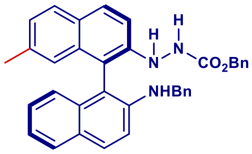

3h

Pale yellow solid, 46 mg. Yield: 86%; e.r. 97:3; m.p. 84–86 °C; $[\alpha]_D^{15}$ = +65.9 (*c* = 0.44, CH_2Cl_2); ^1H NMR (400 MHz, $CDCl_3$): δ 7.86 (d, *J* = 8.4 Hz, 1H), 7.78 – 7.71 (m, 3H), 7.43 – 7.25 (m, 4H), 7.24 – 7.04 (m, 10H), 6.99 (s, 2H), 6.90 (s, 1H), 6.49 (br, 1H), 5.65 (s, 1H), 5.06 (s, 2H), 4.47 (br, 1H), 4.39 (s, 1H), 4.07 (br, 1H), 2.27 (s, 3H); ^{13}C NMR (101 MHz, $CDCl_3$): δ 156.72, 144.36, 144.11, 140.07, 136.82, 135.93, 133.88, 133.62, 129.96, 129.72, 128.65, 128.52, 128.22, 128.17, 127.63, 126.89, 126.01, 123.87, 123.53, 122.09, 114.29, 113.31, 110.70, 67.51, 47.52, 22.08; HRMS (ESI-TOF) *m/z* Calcd. for $C_{36}H_{32}N_3O_2^+$ $[M+H]^+$: 538.2489; Found: 538.2485.

(*R*)-benzyl 2-(2'-(benzylamino)-7-phenyl-[1,1'-binaphthalen]-2-yl)hydrazine-1-carboxylate (3i)

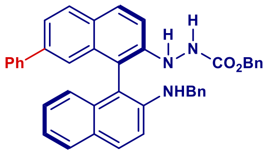

3i

White solid, 59 mg. Yield: 98%; e.r. 97:3; m.p. 91–92 °C; $[\alpha]_D^{15}$ = +113.5 (*c* = 0.95, CH_2Cl_2); ^1H NMR (400 MHz, $CDCl_3$): δ 8.02 – 7.84 (m, 2H), 7.76 (dd, *J* = 9.2, 4.0 Hz, 1H), 7.74 – 7.66 (m, 1H), 7.59 (d, *J* = 6.8 Hz, 1H), 7.41 – 7.39 (m, 3H), 7.37 – 7.23 (m, 7H), 7.22 (s, 1H), 7.21 – 6.87 (m, 10H), 6.52 (br, 1H), 5.71 (s, 1H), 5.07 (s, 2H), 4.52 (br, 1H), 4.40 (s, 1H), 4.11 (br, 1H); ^{13}C NMR (101 MHz, $CDCl_3$): δ 156.75, 144.74, 144.14, 141.42, 140.02, 139.70, 135.90, 133.88, 133.71, 130.22, 129.70, 129.06, 128.95, 128.74, 128.66, 128.51, 128.23, 127.64, 127.54, 127.23, 126.93, 126.87, 126.76, 123.77, 123.46, 122.52, 122.10, 114.38, 114.18, 110.17, 67.56, 47.48; HRMS (ESI-TOF) *m/z* Calcd. for $C_{41}H_{34}N_3O_2^+$ $[M+H]^+$: 600.2645; Found: 600.2652.

(*R*)-benzyl 2-(2'-(benzylamino)-7-methoxy-[1,1'-binaphthalen]-2-yl)hydrazine-1-carboxylate (3j)

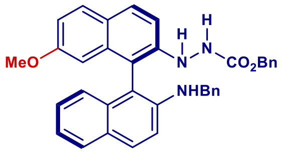

3j

Pale yellow solid, 40 mg. Yield: 72%; e.r. 97:3; m.p. 82–84 °C; $[\alpha]_D^{15}$ = +79.0 (*c* = 0.40, CH_2Cl_2); ^1H NMR (400 MHz, $CDCl_3$): δ 7.83 (d, *J* = 8.4 Hz, 1H), 7.79 – 7.64 (m, 3H), 7.42 – 6.91 (m, 16H), 6.51 (br, 1H), 6.39 (s, 1H), 5.67 (s, 1H), 5.07 (s, 2H), 4.55 (br, 1H), 4.40 (s, 1H), 4.11 (br, 1H), 3.47 (s, 3H); ^{13}C NMR (101 MHz, $CDCl_3$): δ 158.81, 156.76, 144.87, 144.07, 140.14, 135.92, 134.92, 133.76, 130.08, 129.88, 129.70, 128.65, 128.51, 128.16, 127.66, 126.92, 126.89, 125.26, 123.79, 122.08, 115.95, 114.21, 111.72, 110.58, 103.23, 67.53, 55.09, 47.57; HRMS (ESI-TOF) *m/z* Calcd. for $C_{36}H_{32}N_3O_3^+$ $[M+H]^+$: 554.2438; Found: 554.2436.

(*R*)-benzyl 2-(2'-(benzylamino)-6'-methyl-[1,1'-binaphthalen]-2-yl)hydrazine-1-carboxylate (3k)

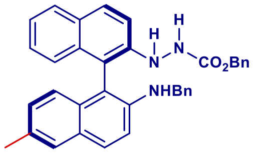

3k

Pale yellow solid, 46 mg. Yield: 86%; e.r. 97.5:2.5; m.p. 78–79 °C; $[\alpha]_D^{15}$ = +38.8 (*c* = 0.50, CH_2Cl_2); ^{1}H NMR (400 MHz, $CDCl_3$): δ 7.88 (d, *J* = 8.8 Hz, 1H), 7.82 (d, *J* = 6.8 Hz, 1H), 7.68 (d, *J* = 8.8 Hz, 1H), 7.49 (s, 1H), 7.37 (d, *J* = 8.8 Hz, 1H), 7.35 – 7.02 (m, 13H), 7.00 - 6.75 (m, 3H), 6.53 (br, 1H), 5.69 (s, 1H), 5.05 (s, 2H), 4.35 (s, 2H), 4.07 (br, 1H), 2.36 (s, 3H); ^{13}C NMR (101 MHz, $CDCl_3$): δ 156.72, 144.23, 143.57, 140.10, 135.79, 133.53, 132.05, 131.45, 129.89, 129.78, 129.36, 129.12, 128.61, 128.46, 128.28, 127.88, 127.22, 127.01, 126.93, 126.84, 124.67, 123.79, 123.60, 114.47, 114.19, 110.68, 67.50, 47.77, 21.35; HRMS (ESI-TOF) *m/z* Calcd. for $C_{36}H_{32}N_3O_2^+$ $[M+H]^+$: 538.2489; Found: 538.2496.

(*R*)-benzyl 2-(2'-(benzylamino)-6'-phenyl-[1,1'-binaphthalen]-2-yl)hydrazine-1-carboxylate (3l)

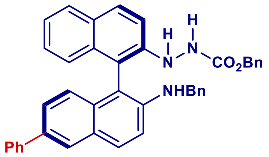

3l

Pale yellow solid, 33 mg. Yield: 55%; e.r. 96:4; m.p. 84–85 °C; $[\alpha]_D^{15}$ = +39.5 (*c* = 0.44, CH_2Cl_2); ^{1}H NMR (400 MHz, $CDCl_3$): δ 7.93 – 7.80 (m, 4H), 7.59 (d, *J* = 6.4 Hz, 2H), 7.45 – 7.35 (m, 4H), 7.34 – 7.24 (m, 6H), 7.20 – 6.90 (m, 10H), 6.54 (br, 1H), 5.74 (s, 1H), 5.06 (s, 2H), 4.53 (br, 1H), 4.39 (s, 1H), 4.08 (br, 1H); ^{13}C NMR (101 MHz, $CDCl_3$): δ 156.67, 144.37, 144.33, 141.42, 139.96, 135.88, 134.77, 133.52, , 133.19, 130.46, 130.09, 129.88, 128.84, 128.67, 128.55, 128.38, 127.85, 127.14, 126.95, 126.91, 126.86, 126.54, 126.16, 124.64, 124.39, 123.73, 114.75, 114,27, 110.41, 67.62, 47.65; HRMS (ESI-TOF) *m/z* Calcd. for $C_{41}H_{34}N_3O_2^+$ $[M+H]^+$: 600.2646; Found: 600.2655.

(*R*)-benzyl 2-(2'-(benzylamino)-6'-methoxy-[1,1'-binaphthalen]-2-yl)hydrazine-1-carboxylate (3m)

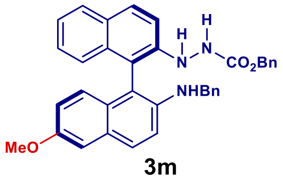

3m

Pale yellow solid, 43 mg. Yield: 78%; e.r. 98:2; m.p. 81–83 °C; $[\alpha]_D^{15}$ = +57.2 (*c* = 0.50, CH_2Cl_2); ^{1}H NMR (400 MHz, $CDCl_3$): δ 7.90 (d, *J* = 8.8 Hz, 1H), 7.83 (d, *J* = 7.6 Hz, 1H), 7.68 (d, *J* = 8.8 Hz, 1H), 7.39 (d, *J* = 8.8 Hz, 1H), 7.37 – 7.27 (m, 4H), 7.22 (s, 1H), 7.22 – 7.12 (m, 5H), 7.12 – 7.02 (m, 4H), 6.98 – 6.67 (m, 3H), 6.53 (br, 1H), 5.70 (s, 1H), 5.06 (s, 2H), 4.36 (br, 2H), 4.08 (br,

1H), 3.83 (s, 3H); ^{13}C NMR (101 MHz, $CDCl_3$): δ 156.70, 155.23, 144.22, 142.65, 140.16, 135.89, 133.53, 129.97, 129.80, 129.30, 128.74, 128.65, 128.48, 128.42, 128.31, 127.04, 126.98, 126.86, 125.62, 124.66, 123.65, 119.36, 115.08, 114.21, 111.39, 106.60, 67.56, 55.41, 48.00; HRMS (ESI-TOF) *m/z* Calcd. for $C_{36}H_{32}N_3O_3^+$ $[M+H]^+$: 554.2438; Found: 554.2443.

(*R*)-benzyl 2-(2'-(benzylamino)-7'-bromo-[1,1'-binaphthalen]-2-yl)hydrazine-1-carboxylate (3n)

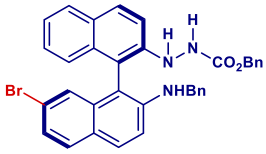

3n

Orange solid, 35 mg. Yield: 58%; e.r. 96:4; m.p. 105–106 °C; $[\alpha]_D^{15}$ = +54.6 (*c* = 0.23, CH_2Cl_2); ^{1}H NMR (400 MHz, $CDCl_3$): δ 7.91 (d, *J* = 8.8 Hz, 1H), 7.85 (d, *J* = 7.6 Hz, 1H), 7.71 (d, *J* = 9.2 Hz, 1H), 7.57 (d, *J* = 8.8 Hz, 1H), 7.39 (d, *J* = 9.2 Hz, 1H), 7.38 – 7.25 (m, 6H), 7.23 (s, 1H), 7.21 – 7.10 (m, 6H), 7.07 (d, *J* = 8.4 Hz, 3H), 6.56 (br, 1H), 5.67 (s, 1H), 5.10 (s, 2H), 4.57 (br, 1H), 4.38 (s, 1H), 4.07 (br, 1H); ^{13}C NMR (101 MHz, $CDCl_3$): δ 156.73, 145.01, 144.36, 139.70, 135.93, 135.31, 133.30, 130.36, 130.05, 129.91, 128.65, 128.57, 128.46, 127.23, 126.99, 126.82, 125.94, 125.71, 125.39, 124.34, 123.78, 121.62, 114.50, 114.27, 109.57, 67.62, 47.52; HRMS (ESI-TOF) *m/z* Calcd. for $C_{35}H_{29}BrN_3O_2^+$ $[M+H]^+$: 602.1438; Found: 602.1439.

(*R*)-benzyl 2-(2'-(benzylamino)-7'-methyl-[1,1'-binaphthalen]-2-yl)hydrazine-1-carboxylate (3o)

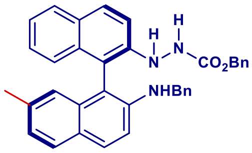

3o

White solid, 50 mg. Yield: 93%; e.r. 98:2; m.p. 82–84 °C; $[\alpha]_D^{15}$ = +78.1 (*c* = 0.54, CH_2Cl_2); ^{1}H NMR (400 MHz, $CDCl_3$): δ 7.91 (d, *J* = 8.8 Hz, 1H), 7.84 (d, *J* = 7.6 Hz, 1H), 7.72 (d, *J* = 8.8 Hz, 1H), 7.62 (d, *J* = 8.0 Hz, 1H), 7.40 (d, *J* = 8.8 Hz, 1H), 7.34 – 7.09 (m, 12H), 7.03 – 6.98 (m, 3H), 6.75 (s, 1H), 6.50 (br, 1H), 5.71 (s, 1H), 5.06 (s, 2H), 4.37 (br, 2H), 4.03 (br, 1H), 2.17 (s, 3H); ^{13}C NMR (101 MHz, $CDCl_3$): δ 156.68, 144.27, 140.10, 136.63, 135.95, 134.12, 133.53, 129.89, 129.85, 129.81, 128.64, 128.47, 128.30, 128.08, 126.99, 126.90, 126.85, 125.92, 124.75, 124.48,

123.62, 122.75, 114.28, 113.40, 109.99, 67.49, 47.67, 22.07; HRMS (ESI-TOF) *m/z* Calcd. for $C_{36}H_{32}N_3O_2^+$ [M+H]$^+$: 538.2489; Found: 538.2497.

(*R*)-benzyl 2-(2'-(benzylamino)-7'-phenyl-[1,1'-binaphthalen]-2-yl)hydrazine-1-carboxylate (3p)

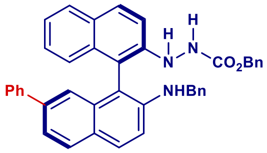

3p

Pale yellow solid, 48 mg. Yield: 80%; e.r. 98:2; m.p. 83–84 °C; $[\alpha]_D^{15}$ = +56.2 (*c* = 1.00, CH_2Cl_2); ^{1}H NMR (400 MHz, $CDCl_3$): δ 7.90 (d, *J* = 9.2 Hz, 1H), 7.83 (d, *J* = 7.6 Hz, 1H), 7.79 (d, *J* = 9.2 Hz, 2H), 7.43 (dd, *J* = 8.4, 1.2 Hz, 2H), 7.39 (d, *J* = 8.8 Hz, 2H), 7.34 – 7.28 (m, 4H), 7.25 – 7.23 (m, 3H), 7.21 (s, 1H), 7.21 – 7.12 (m, 8H), 7.09 (d, *J* = 8.8 Hz, 2H), 6.50 (br, 1H), 5.75 (s, 1H), 5.06 (s, 2H), 4.46 (br, 1H), 4.38 (s, 1H), 4.07 (br, 1H); ^{13}C NMR (101 MHz, $CDCl_3$): δ 156.71, 144.67, 144.33, 141.75, 139.97, 139.55, 135.97, 134.17, 133.51, 130.09, 129.85, 129.80, 128.75, 128.61, 128.52, 128.35, 127.59, 127.05, 126.91, 126.86, 124.65, 123.65, 121.99, 114.39, 114.22, 113.76, 110.92, 67.48, 47.64; HRMS (ESI-TOF) *m/z* Calcd. for $C_{41}H_{34}N_3O_2^+$ [M+H]$^+$: 600.2646; Found: 600.2664.

(*R*)-benzyl 2-(2'-(benzylamino)-7'-methoxy-[1,1'-binaphthalen]-2-yl)hydrazine-1-carboxylate (3q)

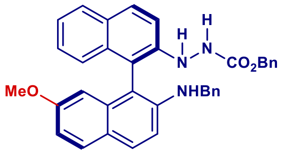

3q

Pale yellow solid, 52 mg. Yield: 94%; e.r. 97:3; m.p. 78–79 °C; $[\alpha]_D^{15}$ = +75.6 (*c* = 1.00, CH_2Cl_2); ^{1}H NMR (400 MHz, $CDCl_3$): δ 7.90 (d, *J* = 9.2 Hz, 1H), 7.83 (d, *J* = 8.0 Hz, 1H), 7.68 (d, *J* = 8.8 Hz, 1H), 7.62 (d, *J* = 9.2 Hz, 1H), 7.39 (d, *J* = 8.8 Hz, 1H), 7.36 – 7.20 (m, 6H), 7.16 – 7.13 (m, 7H), 6.93 (d, *J* = 8.8 Hz, 1H), 6.84 (dd, *J* = 9.2, 2.8 Hz, 1H), 6.51 (br, 1H), 6.33 (s, 1H), 5.75 (s, 1H), 5.07 (s, 2H), 4.35 (br, 2H), 4.06 (br, 1H), 3.49 (s, 3H); ^{13}C NMR (101 MHz, $CDCl_3$): δ 158.70, 156.63, 144.72, 144.21, 140.04, 135.94, 135.20, 133.36, 129.97, 129.86, 129.79, 129.73, 128.66, 128.48, 128.35, 128.30, 128.23, 127.02, 126.87, 124.64, 123.62, 123.09, 114.32, 114.17, 111.79,

109.76, 102.80, 67.51, 55.07, 47.64; HRMS (ESI-TOF) *m/z* Calcd. for $C_{36}H_{32}N_3O_3^+$ [M+H]$^+$: 554.2438; Found: 554.2439.

(*R*)-benzyl 2-(2'-((4-fluorobenzyl)amino)-[1,1'-binaphthalen]-2-yl)hydrazine-1-carboxylate (3r)

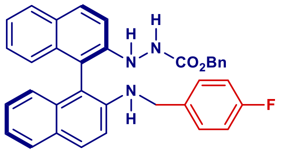

3r

Pale yellow solid, 44 mg. Yield : 81%; e.r. 96.5:3.5; m.p. 79–80 °C; $[\alpha]_D^{15}$ = +66.8 (*c* = 0.50, CH_2Cl_2); ^{1}H NMR (400 MHz, $CDCl_3$): δ 7.92 (d, *J* = 8.8 Hz, 1H), 7.85 (d, *J* = 7.6 Hz, 1H), 7.78 (d, *J* = 8.8 Hz, 1H), 7.72 (d, *J* = 9.2 Hz, 1H), 7.41 (d, *J* = 8.8 Hz, 1H), 7.38 – 7.26 (m, 4H), 7.25 – 7.20 (m, 2H), 7.19 – 7.11 (m, 3H), 7.07 (d, *J* = 8.4 Hz, 3H), 6.95 – 6.85 (m, 4H), 6.54 (br, 1H), 5.71 (s, 1H), 5.08 (s, 2H), 4.48 (br, 1H) , 4.36 (s, 1H), 4.04 (br, 1H); ^{13}C NMR (101 MHz, $CDCl_3$): δ 161.91 (d, $J_{C\text{-}F}$ = 245.3 Hz), 156.74, 144.27, 144.00, 135.87, 135.60, 133.92, 133.48, 130.10, 130.05, 129.85, 128.69, 128.45 (d, $J_{C\text{-}F}$ = 7.9 Hz), 128.36, 128.22, 127.69, 127.06, 126.98, 124.57, 123.82, 123.69, 122.24, 115.3 (d, $J_{C\text{-}F}$ = 21.5 Hz), 114.21, 110.69, 67.60, 47.05; ^{19}F NMR (376 MHz, $CDCl_3$): δ -116.31; HRMS (ESI-TOF) *m/z* Calcd. for $C_{35}H_{29}FN_3O_2^+$ [M+H]$^+$: 542.2238; Found: 542.2234.

(*R*)-benzyl 2-(2'-((4-chlorobenzyl)amino)-[1,1'-binaphthalen]-2-yl)hydrazine-1-carboxylate (3s)

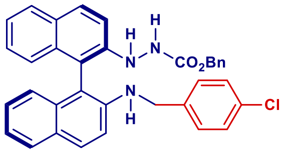

3s

Orange solid, 38 mg. Yield: 68%; e.r. 97.5:2.5; m.p. 75–77 °C; $[\alpha]_D^{15}$ = +33.8 (*c* = 1.00, CH_2Cl_2); ^{1}H NMR (400 MHz, $CDCl_3$): δ 7.92 (d, *J* = 8.4 Hz, 1H), 7.84 (d, *J* = 6.8 Hz, 1H), 7.77 (d, *J* = 9.2 Hz, 1H), 7.72 (d, *J* = 8.8 Hz, 1H), 7.46 – 7.30 (m, 5H), 7.25 – 7.20 (m, 1H), 7.18 – 7.12 (m, 6H), 7.10 – 6.90 (m, 5H), 6.55 (br, 1H), 5.71 (s, 1H), 5.08 (s, 2H), 4.50 (br, 1H), 4.35 (s, 1H), 4.00 (br, 1H); ^{13}C NMR (101 MHz, $CDCl_3$): δ 156.75, 144.27, 143.86, 138.56, 135.86, 133.92, 133.46, 132.54, 130.12, 130.07, 129.85, 128.65, 128.54, 128.37, 128.32, 128.23, 127.71, 127.08, 127.00,

124.55, 123.82, 123.71, 122.28, 114.15, 110.73, 67.61, 47.08; HRMS (ESI-TOF) *m/z* Calcd. for $C_{35}H_{29}ClN_3O_2^+$ [M+H]$^+$: 558.1943; Found: 558.1955.

(*R*)-benzyl 2-(2'-((4-methoxybenzyl)amino)-[1,1'-binaphthalen]-2-yl)hydrazine-1-carboxylate (3t)

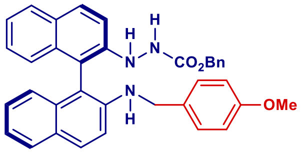

3t

Pink solid, 54 mg. Yield: 98%; e.r. 97:3; m.p. 80–81 °C; $[\alpha]_D^{15}$ = +55.3 (*c* = 0.60, CH_2Cl_2); ^{1}H NMR (400 MHz, $CDCl_3$): δ 7.90 (d, *J* = 8.8 Hz, 1H), 7.83 (d, *J* = 7.6 Hz, 1H), 7.77 (d, *J* = 9.2 Hz, 1H), 7.71 (d, *J* = 9.2 Hz, 1H), 7.39 (d, *J* = 9.2 Hz, 1H), 7.35 – 7.26 (m, 4H), 7.25 – 7.19 (m, 2H), 7.17 – 6.85 (m, 8H), 6.72 (d, *J* = 8.4 Hz, 2H), 6.52 (br, 1H), 5.70 (s, 1H), 5.06 (s, 2H), 4.41 (br, 1H), 4.31 (s, 1H), 4.06 (br, 1H), 3.71 (s, 3H); ^{13}C NMR (101 MHz, $CDCl_3$): δ 158.60, 156.73, 144.29, 135.91, 133.90, 133.50, 131.95, 130.03, 129.96, 129.82, 128.65, 128.44, 128.31, 128.18, 128.13, 127.63, 127.01, 126.88, 124.65, 123.82, 123.63, 122.04, 114.45, 114.22, 113.91, 110.58, 67.53, 55.31, 47.15; HRMS (ESI-TOF) *m/z* Calcd. for $C_{36}H_{32}N_3O_3^+$ [M+H]$^+$: 554.2438; Found: 554.2446.

(*R*)-benzyl 2-(2'-(([1,1'-biphenyl]-4-ylmethyl)amino)-[1,1'-binaphthalen]-2-yl)hydrazine-1-carboxylate (3u)

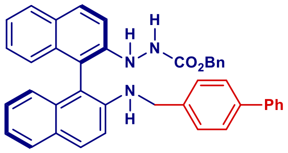

3u

Pale yellow solid, 31 mg. Yield: 52%; e.r. 97:3; m.p. 75–77 °C; $[\alpha]_D^{15}$ = +15.4 (*c* = 1.00, CH_2Cl_2); ^{1}H NMR (400 MHz, $CDCl_3$): δ 7.92 (d, *J* = 8.8 Hz, 1H), 7.84 (d, *J* = 7.6 Hz, 1H), 7.79 (d, *J* = 8.8 Hz, 1H), 7.72 (d, *J* = 9.2 Hz, 1H), 7.51 (d, *J* = 7.6 Hz, 2H), 7.43 – 7.37 (m, 6H), 7.35 – 7.20 (m, 8H), 7.19 – 6.90 (m, 6H), 6.52 (br, 1H), 5.73 (s, 1H), 5.07 (s, 2H), 4.52 (br, 1H), 4.43 (s, 1H), 4.10 (br, 1H); ^{13}C NMR (101 MHz, $CDCl_3$): δ 156.76, 144.30, 144.21, 141.00, 139.86, 139.14, 135.90, 133.95, 133.53, 130.12, 130.03, 129.86, 128.83, 128.66, 128.46, 128.35, 128.22, 127.68, 127.36, 127.27, 127.12, 127.08, 126.95, 124.66, 123.84, 123.69, 122.17, 114.36, 114.24, 110.60, 67.58, 47.44; HRMS (ESI-TOF) *m/z* Calcd. for $C_{41}H_{34}N_3O_2^+$ [M+H]$^+$: 600.2646; Found: 600.2641.

(*R*)-benzyl 2-(2'-((naphthalen-2-ylmethyl)amino)-[1,1'-binaphthalen]-2-yl)hydrazine-1-carboxylate (3v)

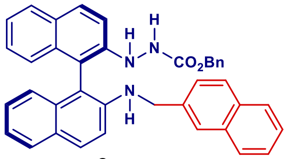

3v

Off-white solid, 55 mg. Yield: 96%; e.r. 97:3; m.p. 91–92 °C; $[\alpha]_D^{15}$ = +23.9 (*c* = 0.36, CH_2Cl_2); ^{1}H NMR (400 MHz, $CDCl_3$): δ 7.93 (d, *J* = 8.4 Hz, 1H), 7.86 (d, *J* = 6.8 Hz, 1H), 7.79 – 7.60 (m, 6H), 7.47 – 7.26 (m, 9H), 7.16 – 6.90 (m, 7H), 6.54 (br, 1H), 5.77 (s, 1H), 5.08 (s, 2H), 4.58 (br, 1H), 4.55 (s, 1H), 4.21 (br, 1H); ^{13}C NMR (101 MHz, $CDCl_3$): δ 156.83, 144.35, 144.12, 137.53, 135.88, 133.95, 133.55, 132.70, 130.01, 130.03, 129.88, 128.66, 128.48, 128.38, 128.21, 127.79, 127.72, 127.65, 127.12, 126.92, 126.07, 125.58, 125.35, 125.29, 124.67, 123.82, 123.70, 122.14, 114.34, 114.26, 110.56, 67.57, 47.67; HRMS (ESI-TOF) *m/z* Calcd. for $C_{39}H_{32}N_3O_2^+$ $[M+H]^+$: 574.2489; Found: 574.2489.

(*R*)-benzyl 2-(2'-(benzylamino)-6,6'-dimethyl-[1,1'-binaphthalen]-2-yl)hydrazine-1-carboxylate (3w)

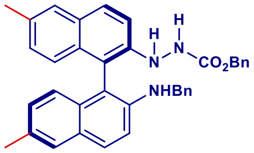

3w

Orange solid, 44 mg. Yield: 80%; e.r. 96.5:3.5; m.p. 81–82 °C; $[\alpha]_D^{15}$ = +33.6 (*c* = 0.53, CH_2Cl_2); ^{1}H NMR (400 MHz, $CDCl_3$): δ 7.82 (d, *J* = 8.8 Hz, 1H), 7.68 (d, *J* = 9.2 Hz, 1H), 7.61 (s, 1H), 7.49 (s, 1H), 7.42 – 7.26 (m, 4H), 7.21 (s, 1H), 7.20 – 7.10 (m, 5H), 7.10 – 6.80 (m, 6H), 6.49 (br, 1H), 5.65 (s, 1H), 5.06 (s, 2H), 4.37 (br, 2H), 4.07 (br, 1H), 2.44 (s, 3H), 2.36 (s, 3H); ^{13}C NMR (101 MHz, $CDCl_3$): δ 156.78, 143.56, 143.52, 140.18, 135.95, 133.08, 132.09, 131.71, 131.44, 130.06, 129.29, 129.26, 129.23, 129.12, 128.64, 128.47, 127.86, 127.35, 127.20, 126.93, 126.84, 124.62, 123.82, 114.46, 114.38, 110.87, 67.49, 47.80, 21.51, 21.36; HRMS (ESI-TOF) *m/z* Calcd. for $C_{37}H_{34}N_3O_2^+$ $[M+H]^+$: 552.2646; Found: 552.2640.

(*R*)-benzyl 2-(2'-(benzylamino)-6'-methyl-6-phenyl-[1,1'-binaphthalen]-2-yl)hydrazine-1-carboxylate (3x)

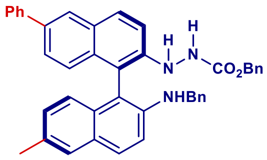

3x

Orange solid, 51 mg. Yield: 82%; e.r. 96.5:3.5; m.p. 91–92 °C; $[\alpha]_D^{15}$ = +24.6 (*c* = 1.0, CH_2Cl_2); ^1H NMR (400 MHz, $CDCl_3$): δ 8.04 (s, 1H), 7.95 (d, *J* = 8.8 Hz, 1H), 7.70 (d, *J* = 9.2 Hz, 1H), 7.67 (d, *J* = 7.2 Hz, 2H), 7.55 – 7.38 (m, 5H), 7.37 – 7.26 (m, 4H), 7.21 – 7.09 (m, 8H), 7.00 – 6.82 (m, 3H), 6.52 (br, 1H), 5.72 (s, 1H), 5.07 (s, 2H), 4.41 (br, 1H), 4.38 (s, 1H), 4.10 (br, 1H), 2.38 (s, 3H); ^{13}C NMR (101 MHz, $CDCl_3$): δ 156.72, 144.39, 143.59, 141.29, 140.09, 136.22, 135.90, 132.80, 132.06, 131.56, 130.25, 130.04, 129.45, 129.21, 128.93, 128.66, 128.50, 127.93, 127.29, 127.14, 126.97, 126.90, 126.65, 126.25, 125.27, 123.78, 114.64, 114.56, 110.61, 67.58, 47.86, 21.38; HRMS (ESI-TOF) *m/z* Calcd. for $C_{42}H_{36}N_3O_2^+$ $[M+H]^+$: 614.2802; Found: 614.2805.

(*R*)-benzyl 2-(2'-(benzylamino)-6-bromo-6'-methoxy-[1,1'-binaphthalen]-2-yl)hydrazine-1-carboxylate (3y)

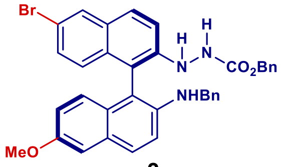

3y

Pale yellow solid, 56 mg. Yield: 88%; e.r. 97.5:2.5; m.p. 87–88 °C; $[\alpha]_D^{15}$ = +38.8 (*c* = 0.50, CH_2Cl_2); ^1H NMR (400 MHz, $CDCl_3$): δ 7.97 (s, 1H), 7.78 (d, *J* = 8.8 Hz, 1H), 7.68 (d, *J* = 8.8 Hz, 1H), 7.39 (d, *J* = 9.2 Hz, 1H), 7.35 – 7.29 (m, 2H), 7.26 (dd, *J* = 9.2, 2.0 Hz, 2H), 7.22 (s, 1H), 7.21 – 7.14 (m, 5H), 7.10 – 7.07 (m, 3H), 6.93 (d, *J* = 8.8 Hz, 1H), 6.91 – 6.74 (m, 2H), 6.55 (br, 1H), 5.70 (s, 1H), 5.06 (s, 2H), 4.35 (s, 1H), 4.24 (br, 1H), 4.10 (br, 1H), 3.82 (s, 3H); ^{13}C NMR (101 MHz, $CDCl_3$): δ 156.68, 155.29, 144.59, 142.61, 140.03, 135.78, 132.09, 130.83, 130.21, 129.14, 129.01, 128.67, 128.53, 128.44, 126.96, 126.53, 125.34, 119.50, 117.32, 115.11, 110.65, 106.67, 67.65, 55.42, 47.98; HRMS (ESI-TOF) *m/z* Calcd. for $C_{36}H_{31}BrN_3O_3^+$ $[M+H]^+$: 632.1543; Found: 632.1537.

(*R*)-benzyl 2-(2'-(benzylamino)-6-bromo-6'-methyl-[1,1'-binaphthalen]-2-yl)hydrazine-1-carboxylate (3z)

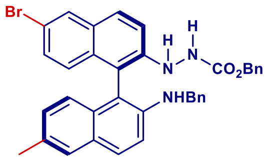

3z

White solid, 60 mg. Yield: 97%; e.r. 97.5:2.5; m.p. 84–85 °C; $[\alpha]_D^{15}$ = +31.5 (*c* = 0.90, CH_2Cl_2); ^{1}H NMR (400 MHz, $CDCl_3$): δ 7.97 (s, 1H), 7.78 (d, *J* = 9.2 Hz, 1H), 7.69 (dd, *J* = 8.8, 2.8 Hz, 1H), 7.50 (s, 1H), 7.39 (d, *J* = 9.2 Hz, 1H), 7.36 – 7.01 (m, 12H), 7.00 – 6.70 (m, 3H), 6.51 (br, 1H), 5.70 (s, 1H), 5.06 (s, 2H), 4.36 (br, 2H), 4.07 (br, 1H), 2.37 (s, 3H); ^{13}C NMR (101 MHz, $CDCl_3$): δ 156.66, 144.61, 143.55, 139.99, 135.80, 132.11, 131.93, 131.65, 130.86, 130.20, 129.63, 129.29, 128.97, 128.67, 128.54, 127.90, 127.31, 126.97, 126.57, 123.53, 117.30, 115.20, 114.52, 109.94, 67.64, 47.83, 21.36; HRMS (ESI-TOF) *m/z* Calcd. for $C_{36}H_{31}BrN_3O_2^+$ $[M+H]^+$: 616.1594; Found: 616.1590.

(*R*)-benzyl 2-(2'-(benzylamino)-6-(methoxycarbonyl)-6'-methyl-[1,1'-binaphthalen]-2-yl)hydrazine-1-carboxylate (3aa)

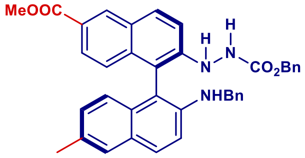

3aa

Off-white solid, 58 mg. Yield: 97%; e.r. 96:4; m.p. 95–96 °C; $[\alpha]_D^{15}$ = +26.4 (*c* = 0.50, CH_2Cl_2); ^{1}H NMR (400 MHz, $CDCl_3$): δ 8.59 (s, 1H), 7.98 (d, *J* = 8.8 Hz, 1H), 7.79 (dd, *J* = 8.8, 1.2 Hz, 1H), 7.70 (d, *J* = 8.8 Hz, 1H), 7.50 (s, 1H), 7.43 (d, *J* = 8.8 Hz, 1H), 7.36 – 7.24 (m, 4H), 7.21 – 7.03 (m, 7H), 6.98 – 6.82 (m, 3H), 6.60 (br, 1H), 5.81 (s, 1H), 5.06 (s, 2H), 4.36 (s, 2H), 4.10 (br, 1H), 3.92 (s, 3H), 2.37 (s, 3H); ^{13}C NMR (101 MHz, $CDCl_3$): δ 167.55, 156.61, 146.44, 143.54, 139.88, 136.06, 135.74, 131.89, 131.63, 131.46, 129.66, 129.27, 128.64, 128.52, 127.91, 127.32, 126.94, 126.90, 126.47, 124.98, 124.71, 123.49, 114.64, 114.51, 113.80, 109.87, 67.67, 52.16, 47.76, 21.33; HRMS (ESI-TOF) *m/z* Calcd. for $C_{38}H_{34}N_3O_4^+$ $[M+H]^+$: 596.2544; Found: 596.2544.

(*R*)-benzyl 2-(2'-(benzylamino)-7,7'-dimethoxy-[1,1'-binaphthalen]-2-yl)hydrazine-1-carboxylate (3ab)

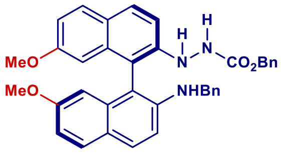

3ab

Pale yellow solid, 57 mg. Yield: 98%; e.r. 96.5:3.5; m.p. 80–82 °C; $[\alpha]_D^{15}$ = +65.9 (*c* = 0.44, CH_2Cl_2); ^1H NMR (400 MHz, $CDCl_3$): δ 7.81 (d, *J* = 8.8 Hz, 1H), 7.72 (d, *J* = 8.8 Hz, 1H), 7.67 (d, *J* = 8.8 Hz, 1H), 7.60 (d, *J* = 8.8 Hz, 1H), 7.40 – 7.05 (m, 11H), 6.98 (dd, *J* = 8.8, 2.0 Hz, 1H), 6.92 (d, *J* = 8.8 Hz, 1H), 6.84 (dd, *J* = 8.8, 2.4 Hz, 1H), 6.52 (br, 1H), 6.43 (d, *J* = 2.4 Hz, 1H), 6.38 (s, 1H), 5.71 (s, 1H), 5.07 (s, 2H), 4.39 (br, 1H), 4.36 (s, 1H), 4.08 (br, 1H), 3.49 (s, 6H); ^{13}C NMR (101 MHz, $CDCl_3$): δ 158.79, 158.69, 156.59, 144.81, 144.55, 140.15, 135.96, 135.05, 134.78, 129.85, 129.79, 129.70, 129.67, 128.65, 128.47, 126.88, 126.85, 125.26, 123.12, 115.92, 114.33, 113.10, 111.67, 109.86, 103.13, 102.67, 67.47, 55.09, 47.50; HRMS (ESI-TOF) *m/z* Calcd. for $C_{37}H_{34}N_3O_4^+$ $[M+H]^+$: 584.2544; Found: 584.2549.

(*R*)-benzyl 2-(2'-(benzylamino)-7-methoxy-7'-phenyl-[1,1'-binaphthalen]-2-yl)hydrazine-1-carboxylate (3ac)

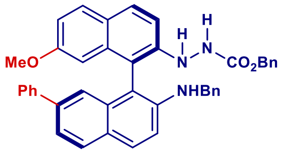

3ac

Orange solid, 50 mg. Yield: 80%; e.r. 98:2; m.p. 92–93 °C; $[\alpha]_D^{15}$ = +14.6 (*c* = 0.58, CH_2Cl_2); ^1H NMR (400 MHz, $CDCl_3$): δ 7.83 (d, *J* = 9.2 Hz, 1H), 7.78 (dd, *J* = 8.4, 4.0 Hz, 2H), 7.73 (d, *J* = 8.4 Hz, 1H), 7.50 – 7.38 (m, 3H), 7.35 – 7.10 (m, 11H) 7.09 (d, *J* = 8.4 Hz, 1H), 6.98 (d, *J* = 8.0 Hz, 1H), 6.50 (br, 1H), 6.44 (d, *J* = 2.4 Hz, 1H), 5.71 (s, 1H), 5.07 (s, 2H), 4.51 (br, 1H), 4.40 (s, 1H), 4.10 (br, 1H), 3.49 (s, 3H); ^{13}C NMR (101 MHz, $CDCl_3$): δ 158.83, 156.70, 144.92, 144.43, 141.73, 140.07, 139.46, 135.99, 134.91, 134.02, 129.91, 129.80, 128.74, 128.64, 128.52, 127.57, 127.06, 126.93, 126.90, 126.86, 125.26, 121.92, 115.89, 114.28, 112.82, 111.71, 111.04, 103.28, 67.47, 55.12, 47.56; HRMS (ESI-TOF) *m/z* Calcd. for $C_{42}H_{36}N_3O_3^+$ $[M+H]^+$: 630.2751; Found: 630.2743.

(*R*)-benzyl 2-(2'-(benzylamino)-7-bromo-6'-methyl-[1,1'-binaphthalen]-2-yl)hydrazine-1-carboxylate (3ad)

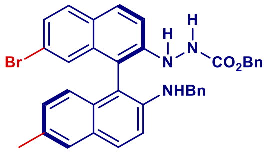

3ad

Pale yellow solid, 53 mg. Yield: 86%; e.r. 96.5:3.5; m.p. 93–94 °C; $[\alpha]_D^{15}$ = +65.2 (*c* = 0.50, CH_2Cl_2); ^{1}H NMR (400 MHz, $CDCl_3$): δ 7.85 (d, *J* = 9.2 Hz, 1H), 7.71 – 7.68 (m, 2H), 7.51 (s, 1H), 7.40 – 7.37 (m, 2H), 7.34 – 7.27 (m, 4H), 7.23 – 7.21 (m, 4H), 7.17 – 7.15 (m, 2H), 7.06 (d, *J* = 8.4 Hz, 2H), 6.91 – 6.74 (m, 2H), 6.50 (br, 1H), 5.70 (s, 1H), 5.07 (s, 2H), 4.38 (s, 1H), 4.32 (br, 1H), 4.04 (br, 1H), 2.38 (s, 3H); ^{13}C NMR (101 MHz, $CDCl_3$): δ 156.63, 145.19, 143.47, 139.94, 135.80, 134.79, 131.82, 131.61, 130.01, 129.85, 129.75, 129.31, 128.68, 128.53, 128.42, 128.17, 127.91, 127.37, 127.06, 126.96, 126.87, 126.62, 123.45, 121.83, 114.44, 109.54, 67.64, 47.59, 21.36; HRMS (ESI-TOF) *m/z* Calcd. for $C_{36}H_{31}BrN_3O_2^+$ $[M+H]^+$: 616.1594; Found: 616.1590.

(*R*)-[1,1'-binaphthalene]-2,2'-diamine

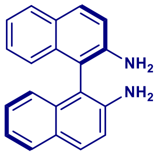

(*R*)-BINAM

White solid, 138 mg. Yield: 85%; e.r. 97:3; $[\alpha]_D^{25}$ = +104.8 (*c* = 0.5, $CHCl_3$); {Lit.[4]: $[\alpha]_D^{20}$ = +112.8 (*c* = 0.5, $CHCl_3$) for 94% ee (*R*)}; ^{1}H NMR (400 MHz, $CDCl_3$): δ 7.83 – 7.72 (m, 4H), 7.24 – 7.18 (m, 4H), 7.14 (d, *J* = 8.8 Hz, 2H), 7.11 – 7.02 (m, 2H), 3.69 (br, 4H); ^{13}C NMR (101 MHz, $CDCl_3$): δ 142.83, 133.81, 129.59, 128.57, 128.27, 126.95, 124.06, 122.54, 118.43, 112.68.

Compounds 5

(*S*)-benzyl 2-(2'-hydroxy-[1,1'-binaphthalen]-2-yl)hydrazine-1-carboxylate (5a)

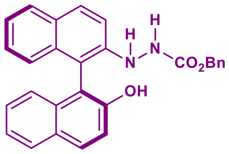

5a

Light yellow solid, 42.6 mg, Yield: 98%; e.r. 2.5:97.5; m.p. 56–58 °C; $[\alpha]_D^{15}$ = -96.0 (*c* = 0.10, $CHCl_3$) {Lit.[4]: $[\alpha]_D^{20}$ = -88.0 (c = 0.5, $CHCl_3$) for 89% ee (*S*)}; ^{1}H NMR (400 MHz, $CDCl_3$): δ 7.95 (d, *J* = 8.8 Hz, 1H), 7.91 – 7.89 (m, 2H), 7.85 (d, *J* = 8.0 Hz, 1H), 7.45 – 7.15 (m, 11H), 7.11

(d, J = 7.6 Hz, 1H), 7.05 (d, J = 7.6 Hz, 1H) , 6.87 (s, 1H), 6.37 (s, 1H), 5.68 (s, 1H), 5.07 (s, 2H); ^{13}C NMR (101 MHz, $CDCl_3$): δ 157.09, 151.86, 144.61, 135.52, 134.05, 133.67, 130.71, 130.63, 129.74, 129.49, 128.63, 128.54, 128.37, 128.26, 127.30, 127.02, 124.67, 124.56, 123.92, 123.81, 119.16, 114.68, 113.68, 112.36, 67.78; HRMS (ESI-TOF) m/z Calcd. for $C_{28}H_{22}N_2O_3Na^+$ $[M+Na]^+$: 457.1523; Found: 457.1525.

(*S*)-propyl 2-(2'-hydroxy-[1,1'-binaphthalen]-2-yl)hydrazine-1-carboxylate (5b)

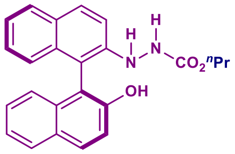

5b

Light yellow solid, 36.3 mg, Yield: 94%; e.r. 3.5:96.5; m.p. 61–62 °C; $[\alpha]_D^{15}$ = -74.0 (c = 0.10, $CHCl_3$); ^{1}H NMR (400 MHz, $CDCl_3$): δ 7.94 (dd, J = 8.8, 5.6 Hz, 2H), 7.88 (d, J = 8.0 Hz, 1H), 7.84 (d, J = 8.4 Hz, 1H), 7.44 – 7.30 (m, 4H), 7.24 – 7.21 (m, 2H), 7.09 (d, J = 8.4 Hz, 1H), 7.01 (d, J = 7.2 Hz, 1H), 6.74 (s, 1H), 6.40 (s, 1H), 5.67 (s, 1H), 4.01 (s, 2H), 1.60 (s, 2H), 0.91 (s, 3H); ^{13}C NMR (101 MHz, $CDCl_3$): δ 157.42, 151.91, 144.77, 134.14, 133.71, 130.73, 130.68, 129.79, 129.55, 128.29, 128.26, 127.31, 127.04, 124.69, 124.62, 123.94, 123.83, 119.31, 114.70, 113.74, 112.53, 67.82, 22.23, 10.23; HRMS (ESI-TOF) m/z Calcd. for $C_{24}H_{22}N_2O_3Na^+$ $[M+Na]^+$: 409.1523; found 409.1526.

(*S*)-isopropyl 2-(2'-hydroxy-[1,1'-binaphthalen]-2-yl)hydrazine-1-carboxylate (5c)

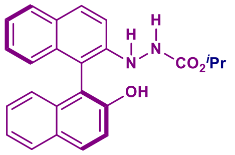

5c

Light yellow solid, 36.3 mg, Yield: 94%; e.r. 8:92; m.p. 60–62 °C; $[\alpha]_D^{15}$ = -68.0 (c = 0.10, $CHCl_3$) {Lit.[4]: $[\alpha]_D^{20}$ = -49.7 (c = 0.5, $CHCl_3$) for 92% ee (*S*)}; ^{1}H NMR (400 MHz, $CDCl_3$): δ 7.95 (dd, J = 8.8, 4.0 Hz, 2H), 7.88 (d, J = 8.0 Hz, 1H), 7.84 (d, J = 8.4 Hz, 1H), 7.44-7.40 (m, 2H), 7.36-7.30 (m, 2H), 7.27-7.20 (m, 2H), 7.09 (d, J = 8.4 Hz, 1H), 6.98 (d, J = 7.6 Hz, 1H), 6.66 (s, 1H), 6.42 (s, 1H), 5.69 (s, 1H), 4.92 (sept, J = 6.0 Hz, 1H), 1.22 (s, 6H); ^{13}C NMR (101 MHz, $CDCl_3$): δ 156.94, 151.91, 144.83, 134.18, 133.73, 130.72, 130.68, 129.80, 129.56, 128.30, 128.25, 127.30, 127.07, 124.71, 124.65, 123.93, 123.84, 119.39, 114.71, 113.83, 112.54, 70.13, 22.14, 22.09; HRMS (ESI-TOF) m/z Calcd. for $C_{24}H_{22}N_2O_3Na^+$ $[M+Na]^+$: 409.1523; found 409.1527.

(*S*)-benzyl 2-(7-bromo-2'-hydroxy-[1,1'-binaphthalen]-2-yl)hydrazine-1-carboxylate (5d)

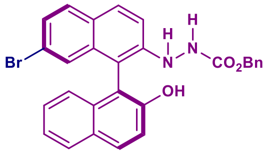

5d

Light yellow solid, 43.6 mg, Yield: 85%; e.r. 3.5:96.5; m.p. 75–76 °C; $[\alpha]_D^{15}$ = -80.0 (*c* = 0.10, $CHCl_3$); ^1H NMR (400 MHz, $CDCl_3$): δ 7.95 (d, *J* = 8.8 Hz, 1H), 7.89-7.86 (m, 2H), 7.69 (d, *J* = 8.8 Hz, 1H), 7.41-7.30 (m, 10H), 7.14 (s, 1H), 7.03 (d, *J* = 7.6 Hz, 1H), 6.76 (s, 1H), 6.19 (s, 1H), 5.67 (s, 1H), 5.07 (s, 2H); ^{13}C NMR (101 MHz, $CDCl_3$): δ 157.05, 151.90, 145.55, 135.43, 135.05, 133.83, 131.18, 130.64, 129.93, 129.64, 128.73, 128.46, 128.22, 127.48, 127.30, 126.59, 124.37, 124.05, 122.08, 119.29, 115.03, 112.91, 111.70, 68.02; HRMS (ESI-TOF) *m*/*z* Calcd. for $C_{28}H_{21}N_2O_3NaBr^+$ $[M+Na]^+$: 535.0628; found 535.0637.

(*S*)-benzyl 2-(2'-hydroxy-7-methyl-[1,1'-binaphthalen]-2-yl)hydrazine-1-carboxylate (5e)

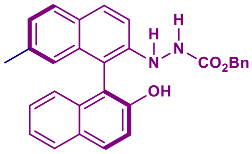

5e

Light yellow solid, 42.6 mg, Yield: 95%; e.r. 4:96; m.p. 66–67 °C; $[\alpha]_D^{20}$ = -98.0 (*c* = 0.10, $CHCl_3$); ^1H NMR (400 MHz, $CDCl_3$): δ 7.95 (d, *J* = 8.8 Hz, 1H), 7.88 (d, *J* = 8.4 Hz, 2H), 7.75 (d, *J* = 8.0 Hz, 1H), 7.44 (d, *J* = 8.4 Hz, 1H), 7.41 – 7.06 (m, 8H), 7.17 (d, *J* = 8.0 Hz, 1H), 7.10 (d, *J* = 7.6 Hz, 1H), 6.78 (s, 1H), 6.75 (s, 1H), 6.19 (s, 1H), 5.64 (s, 1H), 5.16 – 4.98 (m, 2H), 2.23 (s, 3H); ^{13}C NMR (101 MHz, $CDCl_3$): δ 157.05, 151.86, 144.74, 137.22, 135.58, 134.07, 133.88, 130.68, 130.45, 129.55, 128.67, 128.56, 128.39, 128.28, 128.15, 128.08, 127.03, 126.31, 124.71, 123.82, 123.48, 119.15, 113.86, 113.74, 111.77, 67.81, 22.03; HRMS (ESI-TOF) *m*/*z* Calcd. for $C_{29}H_{24}N_2O_3Na^+$ $[M+Na]^+$: 471.1679; found 471.1669.

(*S*)-benzyl 2-(2'-hydroxy-7-phenyl-[1,1'-binaphthalen]-2-yl)hydrazine-1-carboxylate (5f)

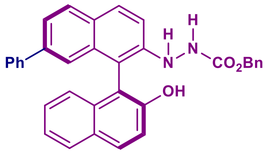

5f

Light yellow solid, 42.4 mg, Yield: 83%; e.r. 4:96; m.p. 67–68 °C; $[\alpha]_D^{15}$ = -36.0 (c = 0.10, $CHCl_3$); ^1H NMR (400 MHz, $CDCl_3$): δ 7.88-7.80 (m, 4H), 7.54 (d, J = 8.0 Hz, 1H), 7.38-7.07 (m, 16H), 6.74 (s, 1H), 6.27 (s, 1H), 5.61 (s, 1H), 4.99 (s, 2H); ^{13}C NMR (101 MHz, $CDCl_3$): δ 157.07, 151.92, 145.06, 141.24, 140.05, 135.53, 133.96, 130.88, 130.39, 129.55, 128.84, 128.68, 128.57, 128.39, 128.33, 127.51, 127.27, 127.08, 124.66, 123.85, 123.81, 122.49, 119.13, 114.74, 113.50, 112.63, 67.84; HRMS (ESI-TOF) m/z Calcd. for $C_{34}H_{26}N_2O_3Na^+$ $[M+Na]^+$: 533.1836; found 533.1830.

(*S*)-benzyl 2-(6-bromo-2'-hydroxy-[1,1'-binaphthalen]-2-yl)hydrazine-1-carboxylate (5g)

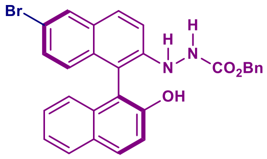

5g

Light yellow solid, 47.2 mg, Yield: 92%; e.r. 4.5:95.5; m.p. 71–72 °C; $[\alpha]_D^{15}$ = -76.0 (c = 0.10, $CHCl_3$); ^1H NMR (400 MHz, $CDCl_3$): δ 7.99 (s, 1H), 7.93 (d, J = 9.2 Hz, 1H), 7.87 (d, J = 8.0 Hz, 1H), 7.81 (d, J = 9.2 Hz, 1H), 7.41-7.24 (m, 10H), 7.01 (d, J = 8.0 Hz, 1H), 6.86 (d, J = 9.2 Hz, 1H), 6.80 (s, 1H), 6.31 (s, 1H), 5.68 (s, 1H), 5.07 (s, 2H); ^{13}C NMR (101 MHz, $CDCl_3$): δ 157.12, 151.87, 144.93, 135.41, 133.93, 132.27, 131.02, 130.88, 130.54, 130.19, 129.71, 129.54, 128.71, 128.38, 127.22, 126.49, 124.44, 123.99, 119.30, 117.76, 115.78, 113.07, 112.73, 68.01; HRMS (ESI-TOF) m/z Calcd. for $C_{28}H_{21}N_2O_3NaBr^+$ $[M+Na]^+$: 535.0628; found 535.0640.

(*S*)-benzyl 2-(2'-hydroxy-6-methyl-[1,1'-binaphthalen]-2-yl)hydrazine-1-carboxylate (5h)

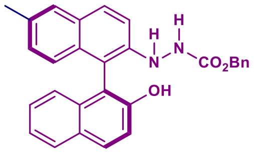

5h

Light yellow solid, 43.5 mg, Yield: 97%; e.r.4:96; m.p. 69–70 °C; $[\alpha]_D^{15}$ = -102.0 (c = 0.10, $CHCl_3$); ^1H NMR (400 MHz, $CDCl_3$): δ 7.94 (d, J = 8.8 Hz, 1H), 7.87 (d, J = 8.0 Hz, 1H), 7.85 (d, J = 10.0 Hz, 1H), 7.63 (s, 1H), 7.43 – 7.23 (m, 9H), 7.08 (d, J = 8.0 Hz, 2H), 6.91 (d, J = 8.4 Hz, 1H), 6.77 (s, 1H), 6.29 (s, 1H), 5.66 (s, 1H), 5.08 (s, 2H), 2.46 (s, 3H); ^{13}C NMR (101 MHz, $CDCl_3$): δ 157.11, 151.87, 143.90, 135.58, 134.14, 133.56, 131.85, 130.71, 130.08, 129.58, 129.52, 128.70, 128.61, 128.43, 128.29, 127.35, 127.06, 124.69, 124.52, 123.82, 119.19, 114.82, 113.81, 112.52,

67.88, 21.45; HRMS (ESI-TOF) *m/z* Calcd. for $C_{29}H_{24}N_2O_3Na^+$ $[M+Na]^+$: 471.1679; found 471.1678.

(*S*)-benzyl 2-(2'-hydroxy-6-phenyl-[1,1'-binaphthalen]-2-yl)hydrazine-1-carboxylate (5i)

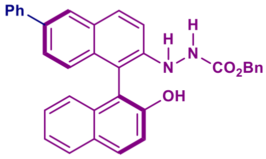

5i

Light yellow solid, 46.5 mg, Yield: 91%; e.r. 6:94; m.p. 86–87 °C; $[\alpha]_D^{20}$ = -44.0 (*c* = 0.10, $CHCl_3$); ^1H NMR (400 MHz, $CDCl_3$): δ 8.06 (s, 1H), 8.02 – 7.93 (m, 2H), 7.90 (d, *J* = 8.4 Hz, 1H), 7.67 (d, *J* = 7.2 Hz, 2H), 7.52 – 7.16 (m, 13H), 7.14 (d, *J* = 8.0 Hz, 1H), 7.09 (d, *J* = 8.4 Hz, 1H) , 6.80 (s, 1H), 6.36 (s, 1H), 5.72 (s, 1H), 5.16 – 4.94 (m, 2H); ^{13}C NMR (101 MHz, $CDCl_3$): δ 157.10, 151.93, 144.75, 141.06, 136.75, 135.53, 134.10, 132.93, 131.03, 130.86, 130.09, 129.57, 128.94, 128.72, 128.65, 128.45, 128.36, 127.30, 127.26, 127.16, 127.01, 126.21, 125.21, 124.67, 123.92, 119.29, 115.20, 113.63, 112.46, 67.96; HRMS (ESI-TOF) *m/z* Calcd. for $C_{34}H_{26}N_2O_3Na^+$ $[M+Na]^+$: 533.1836; found 533.1837.

(*S*)-benzyl 2-(2'-hydroxy-6-(methoxycarbonyl)-[1,1'-binaphthalen]-2-yl)hydrazine-1-carboxylate (5j)

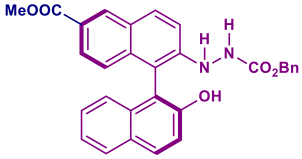

5j

Light yellow solid, 47.8 mg, Yield: 97%; e.r. 3:97; m.p. 85–86 °C; $[\alpha]_D^{20}$ = -28.0 (*c* = 0.10, $CHCl_3$); ^1H NMR (400 MHz, $CDCl_3$): δ 8.58 (s, 1H), 8.00 (d, *J* = 8.8 Hz, 1H), 7.94 (d, *J* = 8.8 Hz, 1H), 7.87 (d, *J* = 8.0 Hz, 1H), 7.78 (dd, *J* = 8.8, 0.8 Hz, 1H), 7.42-7.22 (m, 9H), 7.00 (d, *J* = 8.8 Hz, 2H), 6.87 (s, 1H), 6.25 (s, 1H), 5.79 (s, 1H), 5.07 (s, 2H), 3.92 (s, 3H); ^{13}C NMR (101 MHz, $CDCl_3$): δ 167.39, 157.08, 151.93, 146.78, 136.22, 135.40, 133.83, 132.10, 131.48, 131.04, 129.55, 128.68, 128.39, 127.19, 126.76, 125.28, 124.69, 124.41, 123.97, 119.20, 115.19, 113.00, 112.13, 68.00, 52.21; HRMS (ESI-TOF) *m/z* Calcd. for $C_{30}H_{24}N_2O_5Na^+$ $[M+Na]^+$: 515.1577; found 515.1581.

(*S*)-benzyl 2-(7'-bromo-2'-hydroxy-[1,1'-binaphthalen]-2-yl)hydrazine-1-carboxylate (5k)

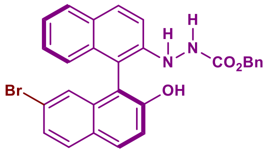

5k

Light yellow solid, 46.7 mg, Yield: 91%; e.r. 7:93; m.p. 66–67 °C; $[\alpha]_D^{15}$ = -92.0 (*c* = 0.10, $CHCl_3$); ^1H NMR (400 MHz, $CDCl_3$): δ 7.87 (t, *J* = 9.2 Hz, 2H), 7.81 (d, *J* = 7.6 Hz, 1H), 7.69 (d, *J* = 8.4 Hz, 1H), 7.39-7.21 (m, 11H), 6.93 (d, *J* = 7.6 Hz, 1H), 6.81 (s, 1H), 6.36 (s, 1H), 5.60 (s, 1H), 5.03 (s, 2H); ^{13}C NMR (101 MHz, $CDCl_3$): δ 157.13, 152.80, 144.76, 135.50, 133.51, 131.08, 130.72, 129.98, 129.87, 128.70, 128.40, 127.98, 127.56, 127.33, 126.69, 124.30, 124.13, 121.76, 119.72, 114.75, 113.13, 111.51, 67.95; HRMS (ESI-TOF) *m/z* Calcd. for $C_{28}H_{21}N_2O_3NaBr^+$ $[M+Na]^+$: 535.0628; found 535.0626.

(*S*)-benzyl 2-(2'-hydroxy-7'-methyl-[1,1'-binaphthalen]-2-yl)hydrazine-1-carboxylate (5l)

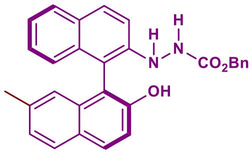

5l

Light yellow solid, 44.0 mg, Yield: 98%; e.r. 4.5:95.5; m.p. 65–66 °C; $[\alpha]_D^{15}$ = -128.0 (*c* = 0.10, $CHCl_3$); ^1H NMR (400 MHz, $CDCl_3$): δ 7.94 (d, *J* = 8.8 Hz, 1H), 7.89 (d, *J* = 8.8 Hz, 1H), 7.86 (d, *J* = 8.0 Hz, 1H), 7.78 (d, *J* = 8.4 Hz, 1H), 7.43 – 7.17 (m, 9H), 7.18 (dd, *J* = 8.0, 1.6 Hz, 1H), 7.04 (d, *J* = 8.4 Hz, 1H), 6.87 (s, 1H), 6.78 (s, 1H), 6.17 (s, 1H), 5.71 (s, 1H), 5.18 – 4.98 (m, 2H), 2.24 (s, 3H); ^{13}C NMR (101 MHz, $CDCl_3$): δ 157.06, 151.95, 144.63, 136.95, 135.56, 134.29, 133.70, 130.60, 130.48, 129.81, 128.66, 128.57, 128.44, 128.23, 128.17, 127.75, 127.31, 126.13, 124.65, 123.95, 123.59, 118.16, 114.72, 113.13, 112.68, 67.83, 21.92; HRMS (ESI-TOF) *m/z* Calcd. for $C_{29}H_{24}N_2O_3Na^+$ $[M+Na]^+$: 471.1679; found 471.1690.

(*S*)-benzyl 2-(2'-hydroxy-7'-phenyl-[1,1'-binaphthalen]-2-yl)hydrazine-1-carboxylate (5m)

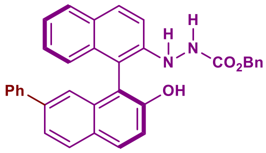

5m

Light yellow solid, 44.9 mg, Yield: 88%; e.r. 5:95; m.p. 89–91 °C; $[\alpha]_D^{20}$ = -70.0 (c = 0.10, $CHCl_3$); ^{1}H NMR (400 MHz, $CDCl_3$): δ 7.98 (d, J = 3.6 Hz, 1H), 7.96 (d, J = 2.8 Hz, 1H), 7.93 (d, J = 9.2 Hz, 1H), 7.85 (d, J = 7.6 Hz, 1H), 7.62 (dd, J = 8.4, 1.2 Hz, 1H), 7.44 – 7.24 (m, 15H), 7.07 (d, J = 7.6 Hz, 1H), 6.76 (s, 1H), 6.26 (s, 1H), 5.73 (s, 1H), 5.09 (s, 1H), 4.90 (br, 1H); ^{13}C NMR (101 MHz, $CDCl_3$): δ 157.05, 152.33, 144.73, 141.28, 139.83, 135.58, 134.39, 133.68, 130.81, 130.50, 129.83, 128.87, 128.74, 128.69, 128.60, 128.45, 128.29, 127.95, 127.58, 127.39, 127.26, 124.55, 124.00, 123.71, 122.74, 119.20, 114.67, 114.05, 112.17, 67.85; HRMS (ESI-TOF) m/z Calcd. for $C_{34}H_{26}N_2O_3Na^+$ $[M+Na]^+$: 533.1836; found 533.1830.

(*S*)-benzyl 2-(7'-cyano-2'-hydroxy-[1,1'-binaphthalen]-2-yl)hydrazine-1-carboxylate (5n)

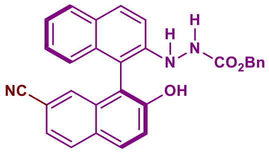

5n

Light yellow solid, 38.6 mg, Yield: 84%; e.r. 7:93; m.p. 74–75 °C; $[\alpha]_D^{20}$ = -92.0 (c = 0.10, $CHCl_3$); ^{1}H NMR (400 MHz, $CDCl_3$): δ 7.98 – 7.88 (m, 3H), 7.86 (d, J = 8.4 Hz, 1H), 7.54 (d, J = 8.0 Hz, 1H), 7.46 – 7.44 (m, 2H), 7.38 – 7.23 (m, 8H), 6.93 (s, 1H), 6.87 (d, J = 8.0 Hz, 1H), 6.66 (s, 1H), 5.60 (s, 1H), 5.16 – 4.90 (m, 2H); ^{13}C NMR (101 MHz, $CDCl_3$): δ 157.22, 153.40, 144.86, 135.42, 133.42, 131.45, 130.94, 130.90, 130.73, 129.89, 129.46, 128.70, 128.67, 128.56, 128.42, 127.71, 124.55, 124.28, 123.98, 122.57, 119.38, 114.76, 114.43, 110.66, 110.38, 68.01; HRMS (ESI-TOF) m/z Calcd. for $C_{29}H_{21}N_3O_3Na^+$ $[M+Na]^+$: 482.1475; found 482.1481.

(*S*)-benzyl 2-(2'-hydroxy-7'-methoxy-[1,1'-binaphthalen]-2-yl)hydrazine-1-carboxylate (5o)

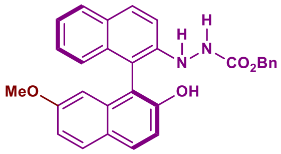

5o

Light yellow solid, 45.5 mg, Yield: 98%; e.r. 5:95; m.p. 65–66 °C; $[\alpha]_D^{20}$ = -78.0 (c = 0.10, $CHCl_3$); ^{1}H NMR (400 MHz, $CDCl_3$): δ 7.91 (d, J = 8.8 Hz, 1H), 7.86 – 7.83 (m, 2H), 7.77 (d, J = 8.8 Hz, 1H), 7.38 – 7.23 (m, 9H), 7.06 (d, J = 8.4 Hz, 1H), 7.01 (dd, J = 8.8, 2.4 Hz, 1H), 6.76 (s, 1H), 6.42 (d, J = 2.4 Hz, 1H), 6.08 (s, 1H), 5.71 (s, 1H), 5.15 – 5.01 (m, 2H), 3.51 (s, 3H); ^{13}C NMR (101 MHz, $CDCl_3$): δ 158.81, 156.98, 152.49, 144.65, 135.61, 135.42, 133.52, 130.75, 130.50,

129.86, 128.71, 128.61, 128.44, 128.27, 127.39, 124.86, 124.53, 123.98, 116.37, 115.86, 114.59, 112.77, 112.27, 103.84, 67.86, 55.16; HRMS (ESI-TOF) *m/z* Calcd. for $C_{29}H_{24}N_2O_4Na^+$ $[M+Na]^+$: 487.1628; found 487.1628.

(*S*)-benzyl 2-(6'-bromo-2'-hydroxy-[1,1'-binaphthalen]-2-yl)hydrazine-1-carboxylate (5p)

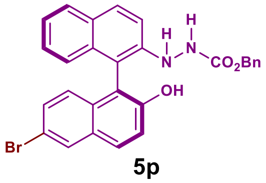

5p

Light yellow solid, 44.7 mg, Yield: 87%; e.r. 5:95; m.p. 71–81 °C; $[\alpha]_D^{20}$ = -72.0 (*c* = 0.10, $CHCl_3$); ^1H NMR (400 MHz, $CDCl_3$): δ 8.03 (d, *J* = 1.6 Hz, 1H), 7.92 (d, *J* = 8.8 Hz, 1H), 7.85 – 7.82 (m, 2H), 7.43 – 7.23 (m, 10H), 6.95 (d, *J* = 8.0 Hz, 2H), 6.83 (s, 1H), 6.39 (s, 1H), 5.64 (s, 1H), 5.07 (s, 2H), ^{13}C NMR (101 MHz, $CDCl_3$): δ 157.12, 152.21, 144.65, 135.44, 133.56, 132.67, 130.97, 130.67, 130.28, 130.24, 129.82, 128.71, 128.65, 128.43, 128.36, 127.51, 126.60, 124.36, 124.12, 120.38, 117.68, 114.68, 113.96, 111.71, 67.98; HRMS (ESI-TOF) *m/z* Calcd. for $C_{28}H_{21}N_2O_3NaBr^+$ $[M+Na]^+$: 535.0628; found 535.0626.

(*S*)-benzyl 2-(2'-hydroxy-6'-methyl-[1,1'-binaphthalen]-2-yl)hydrazine-1-carboxylate (5q)

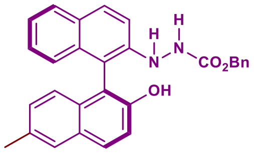

5q

Light yellow solid, 41.3 mg, Yield: 92%; e.r. 5:95; m.p. 63–64 °C; $[\alpha]_D^{15}$ = -112.0 (*c* = 0.10, $CHCl_3$); ^1H NMR (400 MHz, $CDCl_3$): δ 7.94 (d, *J* = 9.2 Hz, 1H), 7.85 (d, *J* = 8.8 Hz, 2H), 7.65 (s, 1H), 7.40-7.32 (m, 9H), 7.07 (s, 1H), 7.01 (d, *J* = 8.8 Hz, 1H), 6.98 (d, *J* = 8.4 Hz, 1H), 6.75 (s, 1H), 6.15 (s, 1H), 5.71 (s, 1H), 5.19 – 4.95 (m, 2H), 2.45 (s, 3H); ^{13}C NMR (101 MHz, $CDCl_3$): δ 157.08, 151.20, 144.57, 135.55, 133.72, 133.33, 132.20, 130.63, 130.09, 129.75, 129.27, 128.69, 128.59, 128.42, 128.24, 127.42, 127.33, 124.63, 124.56, 123.96, 119.20, 114.68, 113.59, 112.66, 67.87, 21.43; HRMS (ESI-TOF) *m/z* Calcd. for $C_{29}H_{24}N_2O_3Na^+$ $[M+Na]^+$: 471.1679; found 471.1675.

(*S*)-benzyl 2-(2'-hydroxy-6'-phenyl-[1,1'-binaphthalen]-2-yl)hydrazine-1-carboxylate (5r)

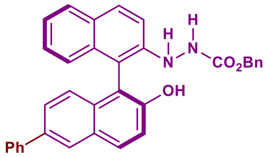

5r

Light yellow solid, 49.5 mg, Yield: 97%; e.r. 4:96; m.p. 78–80 °C; $[\alpha]_D^{15}$ = -66.0 (*c* = 0.10, $CHCl_3$); ^1H NMR (400 MHz, $CDCl_3$): δ 8.10 (d, *J* = 1.6 Hz, 1H), 8.00 (d, *J* = 8.8 Hz, 1H), 7.95 (d, *J* = 8.8 Hz, 1H), 7.87 (d, *J* = 8.0 Hz, 1H), 7.67 (d, *J* = 7.6 Hz, 2H), 7.49 – 7.25 (m, 13H), 7.17 (d, *J* = 8.0 Hz, 1H), 7.08 (d, *J* = 8.4 Hz, 1H), 6.81 (s, 1H), 6.37 (br, 1H), 5.74 (s, 1H), 5.20 – 4.90 (m, 2H); ^{13}C NMR (101 MHz, $CDCl_3$): δ 157.11, 152.02, 144.65, 141.04, 136.63, 135.52, 133.70, 133.32, 131.08, 130.80, 129.81, 128.92, 128.70, 128.62, 128.43, 128.31, 127.43, 127.30, 127.25, 126.71, 126.25, 125.26, 124.62, 124.05, 119.69, 114.75, 113.65, 112.38, 67.92; HRMS (ESI-TOF) *m/z* Calcd. for $C_{34}H_{26}N_2O_3Na^+$ $[M+Na]^+$: 533.1836; found 533.1829.

(*S*)-benzyl 2-(6'-cyano-2'-hydroxy-[1,1'-binaphthalen]-2-yl)hydrazine-1-carboxylate (5s)

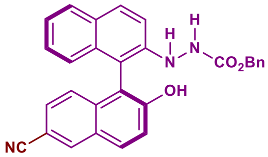

5s

Light yellow solid, 43.7 mg, Yield: 95%; e.r. 5:95; m.p. 74–75 °C; $[\alpha]_D^{20}$ = -58.0 (*c* = 0.10, $CHCl_3$); ^1H NMR (400 MHz, $CDCl_3$): δ 8.23 (s, 1H), 7.98-7.95 (m, 2H), 7.86 (d, *J* = 7.6 Hz, 1H), 7.51 (d, *J* = 8.4 Hz, 1H), 7.43 – 7.18 (m, 9H), 7.13 (d, *J* = 8.4 Hz, 1H), 6.88 (s, 1H), 6.86 (s, 1H), 6.68 (s, 1H), 5.62 (s, 1H), 5.16 – 4.92 (m, 2H); ^{13}C NMR (101 MHz, $CDCl_3$): δ 157.18, 154.69, 144.84, 136.02, 135.37, 134.33, 133.42, 131.32, 129.87, 128.75, 128.50, 128.42, 127.72, 125.97, 124.28, 124.08, 121.13, 119.56, 114.75, 114.21, 110.85, 107.13, 77.48, 77.16, 76.84, 68.07; HRMS (ESI-TOF) *m/z* Calcd. for $C_{29}H_{21}N_3O_3Na^+$ $[M+Na]^+$: 482.1475; found 482.1479.

(*S*)-benzyl 2-(2'-hydroxy-6'-methoxy-[1,1'-binaphthalen]-2-yl)hydrazine-1-carboxylate (5t)

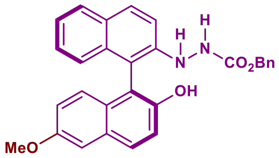

5t

Light yellow solid, 42.6 mg, Yield: 92%; e.r. 3.5:96.5; m.p. 65–66 °C; $[\alpha]_D^{20}$ = -116.0 (c = 0.10, $CHCl_3$); ^{1}H NMR (400 MHz, $CDCl_3$): δ 7.92 (d, J = 8.8 Hz, 1H), 7.84 – 7.82 (m, 2H), 7.39 – 7.31 (m, 7H), 7.24 – 7.21 (m, 3H), 7.02 – 6.94 (m, 3H), 6.80 (s, 1H), 6.15 (s, 1H), 5.70 (s, 1H), 5.16 – 5.01 (m, 2H), 3.89 (s, 3H); ^{13}C NMR (101 MHz, $CDCl_3$): δ 157.10, 156.33, 150.21, 144.53, 135.54, 133.71, 130.70, 130.50, 129.85, 129.46, 129.31, 128.72, 128.63, 128.45, 128.27, 127.37, 126.27, 124.67, 124.03, 119.72, 119.44, 114.72, 114.10, 112.76, 106.82, 67.95, 55.45; HRMS (ESI-TOF) m/z Calcd. for $C_{29}H_{24}N_2O_4Na^+$ $[M+Na]^+$: 487.1628; found 487.1628.

(*S*)-benzyl 2-(2'-hydroxy-6'-(methoxycarbonyl)-[1,1'-binaphthalen]-2-yl)hydrazine-1-carboxylate (5u)

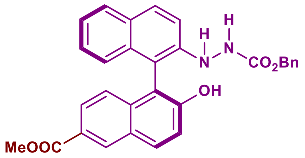

5u

Light yellow solid, 48.3 mg, Yield: 98%; e.r. 3.5:96.5; m.p. 80–81 °C; $[\alpha]_D^{20}$ = -56.0 (c = 0.10, $CHCl_3$); ^{1}H NMR (400 MHz, $CDCl_3$): δ 8.62 (s, 1H), 8.03 (d, J = 8.8 Hz, 1H), 7.94 (d, J = 8.8 Hz, 1H), 7.85 (d, J = 8.0 Hz, 1H), 7.80 (d, J = 7.6 Hz, 1H), 7.46 (d, J = 8.0 Hz, 1H), 7.40 – 7.17 (m, 8H), 7.09 (d, J = 8.4 Hz, 1H), 6.93 (d, J = 8.4 Hz, 1H), 6.86 (s, 1H), 6.52 (s, 1H), 5.67 (s, 1H), 5.08 (s, 2H), 3.93 (s, 3H); ^{13}C NMR (101 MHz, $CDCl_3$): δ 167.38, 157.17, 154.07, 144.73, 136.71, 135.46, 133.55, 132.22, 131.46, 131.00, 129.82, 128.68, 128.61, 128.52, 128.37, 127.49, 126.54, 125.42, 124.90, 124.31, 124.09, 120.09, 114.74, 113.89, 111.62, 77.48, 77.16, 76.84, 67.93, 52.21; HRMS (ESI-TOF) m/z Calcd. for $C_{30}H_{24}N_2O_5Na^+$ $[M+Na]^+$: 515.1577; found 515.1571.

(*S*)-benzyl 2-(6'-formyl-2'-hydroxy-[1,1'-binaphthalen]-2-yl)hydrazine-1-carboxylate (5v)

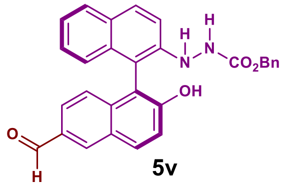

5v

Light yellow solid, 34.7 mg, Yield: 75%; e.r. 4:96; m.p. 73–74 °C; $[\alpha]_D^{20}$ = -44.0 (c = 0.10, $CHCl_3$); ^{1}H NMR (400 MHz, $CDCl_3$): δ 10.04 (s, 1H), 8.33 (s, 1H), 8.07 (d, J = 9.2 Hz, 1H), 7.94 (d, J = 8.8 Hz, 1H), 7.94 (d, J = 8.0 Hz, 1H), 7.68 (d, J = 7.2 Hz, 1H), 7.49 (d, J = 8.4 Hz, 1H), 7.40 – 7.18 (m, 8H), 7.15 (d, J = 8.0 Hz, 1H), 6.93 (s, 1H), 6.91 (s, 1H), 6.70 (br, 1H), 5.66 (s, 1H), 5.07

(s, 2H); ^{13}C NMR (101 MHz, $CDCl_3$): δ 192.08, 157.19, 154.93, 144.76, 137.82, 135.43, 134.61, 133.49, 132.48, 132.37, 131.18, 129.84, 128.71, 128.66, 128.60, 128.45, 127.60, 125.77, 124.40, 124.18, 120.46, 114.75, 114.40, 111.29, 67.99; HRMS (ESI-TOF) *m/z* Calcd. for $C_{29}H_{22}N_2O_4Na^+$ $[M+Na]^+$: 485.1472; found 485.1473.

(*S*)-benzyl 2-(6'-cyclohexyl-2'-hydroxy-[1,1'-binaphthalen]-2-yl)hydrazine-1-carboxylate (5w)

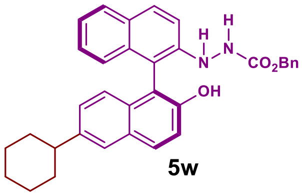

5w

Light yellow solid, 44.9 mg, Yield: 87%; e.r. 3.5:96.5; m.p. 64–65 °C; $[\alpha]_D^{20}$ = -90.0 (*c* = 0.10, $CHCl_3$); ^{1}H NMR (400 MHz, $CDCl_3$): δ 7.93 – 7.88 (m, 2H), 7.84 (d, *J* = 7.6 Hz, 1H), 7.69 (s, 1H), 7.38 – 7.12 (m, 10H), 7.05 (d, *J* = 8.0 Hz, 1H), 7.01 (d, *J* = 8.0 Hz, 1H), 6.78 (s, 1H), 6.23 (s, 1H), 5.69 (s, 1H), 5.20 – 4.90 (s, 2H), 2.65 – 2.59 (m, 1H), 2.01 – 1.71 (m, 5H) , 1.55 – 1.39 (m, 4H), 1.34 – 1.25 (m, 1H); ^{13}C NMR (101 MHz, $CDCl_3$): δ 157.07, 151.23, 144.57, 143.49, 135.56, 133.71, 132.62, 130.60, 130.44, 129.74, 128.68, 128.58, 128.42, 128.22, 127.38, 127.29, 125.01, 124.70, 124.53, 123.95, 119.08, 114.72, 113.53, 112.76, 67.86, 44.36, 34.54, 34.45, 27.04, 26.30; HRMS (ESI-TOF) *m/z* Calcd. for $C_{34}H_{32}N_2O_3Na^+$ $[M+Na]^+$: 539.2305; found 539.2315.

(*S*)-benzyl 2-(3'-bromo-2'-hydroxy-[1,1'-binaphthalen]-2-yl)hydrazine-1-carboxylate (5x)

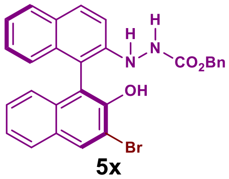

5x

Light yellow solid, 44.2 mg, Yield: 86%; e.r. 5:95; m.p. 71–73 °C; $[\alpha]_D^{20}$ = -98.0 (c = 0.10, $CHCl_3$); ^{1}H NMR (400 MHz, $CDCl_3$): δ 8.26 (s, 1H), 7.91 (d, *J* = 9.0 Hz, 1H), 7.84 (d, *J* = 8.0 Hz, 1H), 7.79 (d, *J* = 8.2 Hz, 1H), 7.40 – 7.10 (m, 10H), 7.06 (d, *J* = 7.6 Hz, 1H), 6.96 (d, *J* = 8.0 Hz, 1H), 6.80 (s, 1H), 6.76 (s, 1H), 5.61 (s, 1H), 5.06 (s, 2H); ^{13}C NMR (101 MHz, $CDCl_3$): δ 157.03, 148.44, 144.39, 135.50, 133.36, 133.11, 130.90, 129.98, 129.72, 128.66, 128.59, 128.40, 128.31, 127.45, 127.37, 127.33, 124.97, 124.83, 124.37, 124.02, 115.61, 114.69, 113.41, 112.32, 67.90; HRMS (ESI-TOF) *m/z* Calcd. for $C_{28}H_{21}N_2O_3NaBr^+$ $[M+Na]^+$: 535.0628; found 535.0624.

(*S*)-benzyl 2-(2'-hydroxy-3'-methyl-[1,1'-binaphthalen]-2-yl)hydrazine-1-carboxylate (5y)

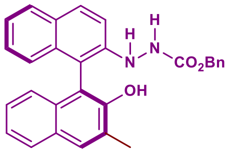

5y

Light yellow solid, 42.2 mg, Yield: 94%; e.r. 3.5:96.5; m.p. 61–63 °C; $[\alpha]_D^{20}$ = -100.0 (*c* = 0.10, $CHCl_3$); ^{1}H NMR (400 MHz, $CDCl_3$): δ 7.88 (d, *J* = 8.8 Hz, 1H), 7.80 (d, *J* = 8.0 Hz, 1H), 7.77 – 7.75 (m, 2H), 7.31 – 7.13 (m, 10H), 6.98 (d, *J* = 7.2 Hz, 2H), 6.71 (s, 1H), 6.14 (s, 1H), 5.64 (s, 1H), 5.04 (s, 2H), 2.51 (s, 3H); ^{13}C NMR (101 MHz, $CDCl_3$): δ 156.98, 151.22, 144.67, 135.60, 133.76, 132.75, 130.66, 130.18, 129.78, 129.49, 128.67, 128.58, 128.40, 128.26, 127.47, 127.35, 126.03, 124.61, 124.43, 123.93, 123.76, 114.59, 112.98, 112.59, 67.82, 17.39; HRMS (ESI-TOF) *m/z* Calcd. for $C_{29}H_{24}N_2O_3Na^+$ $[M+Na]^+$: 471.1679; found 471.1679.

(*S*)-benzyl 2-(2'-hydroxy-3'-phenyl-[1,1'-binaphthalen]-2-yl)hydrazine-1-carboxylate (5z)

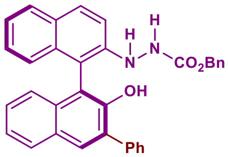

5z

Light yellow solid, 48.5 mg, Yield: 95%; e.r. 6.5:93.5; m.p. 74–75 °C; $[\alpha]_D^{15}$ = -162.0 (*c* = 0.10, $CHCl_3$); ^{1}H NMR (400 MHz, $CDCl_3$): δ 8.04 (s, 1H), 7.94 (d, *J* = 8.8 Hz, 1H), 7.92 (d, *J* = 7.6 Hz, 1H), 7.87 (d, *J* = 8.0 Hz, 1H), 7.80 (d, *J* = 6.8 Hz, 2H), 7.48 (t, *J* = 7.6 Hz, 2H), 7.40 – 7.12 (m, 13H), 6.74 (s, 1H), 6.18 (s, 1H), 5.71 (s, 1H), 5.05 (s, 2H); ^{13}C NMR (101 MHz, $CDCl_3$): δ 156.89, 149.62, 144.82, 138.13, 135.59, 133.70, 133.34, 131.62, 131.02, 130.74, 129.84, 129.53, 128.62, 128.52, 128.35, 128.31, 127.51, 127.41, 127.02, 124.58, 124.19, 123.93, 114.70, 114.50, 112.28, 67.74; HRMS (ESI-TOF) *m/z* Calcd. for $C_{34}H_{26}N_2O_3Na^+$ $[M+Na]^+$: 533.1836; found 533.1844.

(*S*)-benzyl 2-(2'-hydroxy-6,6'-dimethyl-[1,1'-binaphthalen]-2-yl)hydrazine-1-carboxylate (5aa)

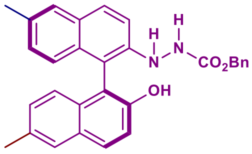

5aa

Light yellow solid, 31.0 mg, Yield: 67%; e.r. 6:94; m.p. 70–71 °C; $[\alpha]_D^{15}$ = -70.0 (c = 0.10, $CHCl_3$); ^1H NMR (400 MHz, $CDCl_3$): δ 7.86 – 7.84 (m, 2H), 7.65 (s, 1H) , 7.62 (s, 1H), 7.37 – 7.32 (m, 6H), 7.14 – 6.84 (m, 1H), 7.08 (d, J = 8.0 Hz, 2H), 6.98 (d, J = 8.4 Hz, 1H), 6.92 (d, J = 8.4 Hz, 1H), 6.74 (s, 1H), 6.18 (s, 1H), 5.67 (s, 1H), 5.09 (s, 2H), 2.45 (s, 6H); ^{13}C NMR (101 MHz, $CDCl_3$): δ 157.09, 151.19, 143.84, 135.60, 133.52, 133.30, 132.25, 131.87, 130.02, 129.73, 129.55, 129.26, 128.70, 128.60, 128.42, 127.40, 127.32, 124.58, 119.19, 114.79, 113.73, 112.74, 67.86, 21.44; HRMS (ESI-TOF) m/z Calcd. for $C_{30}H_{26}N_2O_3Na^+$ $[M+Na]^+$: 485.1836; found 485.1839.

(*S*)-benzyl 2-(2'-hydroxy-7,7'-dimethyl-[1,1'-binaphthalen]-2-yl)hydrazine-1-carboxylate (5ab)

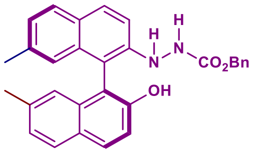

5ab

Light yellow solid, 42.1 mg, Yield: 91%; e.r. 4.5:95.5; m.p. 77–78 °C; $[\alpha]_D^{20}$ = -96.0 (c = 0.10, $CHCl_3$); ^1H NMR (400 MHz, $CDCl_3$): δ 7.90 (d, J = 8.8 Hz, 2H), 7.80 – 7.75 (m, 2H), 7.40 – 7.10 (m, 7H), 7.19 (d, J = 8.4 Hz, 2H), 6.89 (s, 1H), 6.81 (s, 1H), 6.73 (s, 1H), 6.07 (s, 1H), 5.65 (s, 1H), 5.16 – 4.98 (m, 2H), 2.25 (s, 6H); ^{13}C NMR (101 MHz, $CDCl_3$): δ 157.00, 151.95, 144.78, 137.23, 136.95, 135.63, 134.26, 133.92, 130.45, 128.71, 128.61, 128.46, 128.18, 128.14, 127.80, 126.36, 126.16, 123.62, 118.12, 113.78, 113.31, 112.02, 77.48, 77.16, 76.84, 67.87, 22.08, 22.01; HRMS (ESI-TOF) m/z Calcd. for $C_{30}H_{26}N_2O_3Na^+$ $[M+Na]^+$: 485.1836; found 485.1832.

(*S*)-benzyl 2-(6,6'-dibromo-2'-hydroxy-[1,1'-binaphthalen]-2-yl)hydrazine-1-carboxylate (5ac)

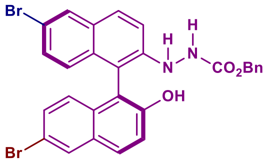

5ac

Light yellow solid, 47.4 mg, Yield: 80%; e.r. 6:94; m.p. 76–77 °C; $[\alpha]_D^{15}$ = -24.0 (c = 0.10, $CHCl_3$); ^1H NMR (400 MHz, $CDCl_3$): δ 8.02 (d, J = 1.6 Hz, 1H), 7.99 (s, 1H), 7.84 – 7.82 (m, 2H), 7.45 – 7.15 (m, 9H), 6.88 (d, J = 8.0 Hz, 1H), 6.80 (s, 1H), 6.78 (s, 1H), 6.39 (s, 1H), 5.65 (s, 1H), 5.15 – 4.95 (m, 2H); ^{13}C NMR (101 MHz, $CDCl_3$): δ 157.13, 152.22, 144.97, 135.34, 132.51, 132.14, 130.90, 130.71, 130.46, 130.35, 130.30, 130.10, 130.01, 128.76, 128.47, 126.35, 126.24, 120.51,

117.92, 117.84, 115.79, 113.35, 112.03, 68.15; HRMS (ESI-TOF) *m*/*z* Calcd. for $C_{28}H_{20}N_2O_3NaBr_2^+$ $[M+Na]^+$: 612.9733; found 612.9725.

(*S*)-dimethyl 2-(2-((benzyloxy)carbonyl)hydrazinyl)-2'-hydroxy-[1,1'-binaphthalene]-6,6'-dicarboxylate (5ad)

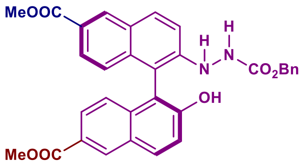

5ad

Light yellow solid, 52.9 mg, Yield: 96%; e.r. 6:94; m.p. 109–110 °C; $[\alpha]_D^{20}$ = +30.0 (*c* = 0.10, $CHCl_3$); ^1H NMR (400 MHz, $CDCl_3$): δ 8.57 (d, *J* = 1.2 Hz, 1H), 8.54 (d, *J* = 0.8 Hz, 1H), 7.97 (t, *J* = 8.2 Hz, 2H), 7.74 (dd, *J* = 8.8, 1.6 Hz, 2H), 7.43 – 7.12 (m, 7H), 7.07 (s, 1H), 7.02 (d, *J* = 7.6 Hz, 1H), 6.92 (d, *J* = 8.8 Hz, 1H), 6.66 (s, 1H), 5.80 (s, 1H), 5.04 (s, 2H), 3.89 (s, 6H); ^{13}C NMR (101 MHz, $CDCl_3$): δ 167.28, 157.14, 154.18, 146.90, 136.42, 136.07, 135.36, 132.44, 132.32, 131.51, 131.48, 128.66, 128.62, 128.49, 128.33, 126.86, 126.64, 125.44, 125.29, 124.61, 124.36, 120.09, 115.23, 113.23, 111.27, 67.99, 52.22; HRMS (ESI-TOF) *m*/*z* Calcd. for $C_{32}H_{26}N_2O_7Na^+$ $[M+Na]^+$: 573.1632; found 573.1642.

(*S*)-2-Amino-2'-hydroxy-1,1'-binaphthyl

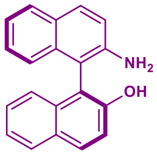

(*S*)-NOBIN

White solid, 27.3 mg. Yield: 96%; e.r.6:94; $[\alpha]_D^{25}$ = -42.4 (*c* = 0.5, $CHCl_3$) {Lit.[4] $[\alpha]_D^{20}$ = -54.0 (c = 0.5, $CHCl_3$) for 99% ee (*S*)}; ^1H NMR (400 MHz, DMSO-d_6): δ 9.31 (s, 1H), 7.88 (t, *J* = 9.2 Hz, 2H), 7.74-7.72 (m, 2H), 7.38 (d, *J* = 8.8 Hz, 1H), 7.27-7.23 (m, 1H), 7.20-7.17 (m, 2H), 7.11-7.05 (m, 2H), 6.96 (d, *J* = 8.4 Hz, 1H), 6.78-6.76 (m, 1H), 4.55 (s, 2H); ^{13}C NMR (101 MHz, DMSO-d_6): δ 153.33, 143.96, 134.05, 133.67, 129.13, 128.48, 128.14, 128.07, 127.81, 127.03, 126.15, 125.71, 124.16, 123.48, 122.56, 120.78, 118.82, 118.47, 114.95, 111.26.

5. NMR and HPLC spectra

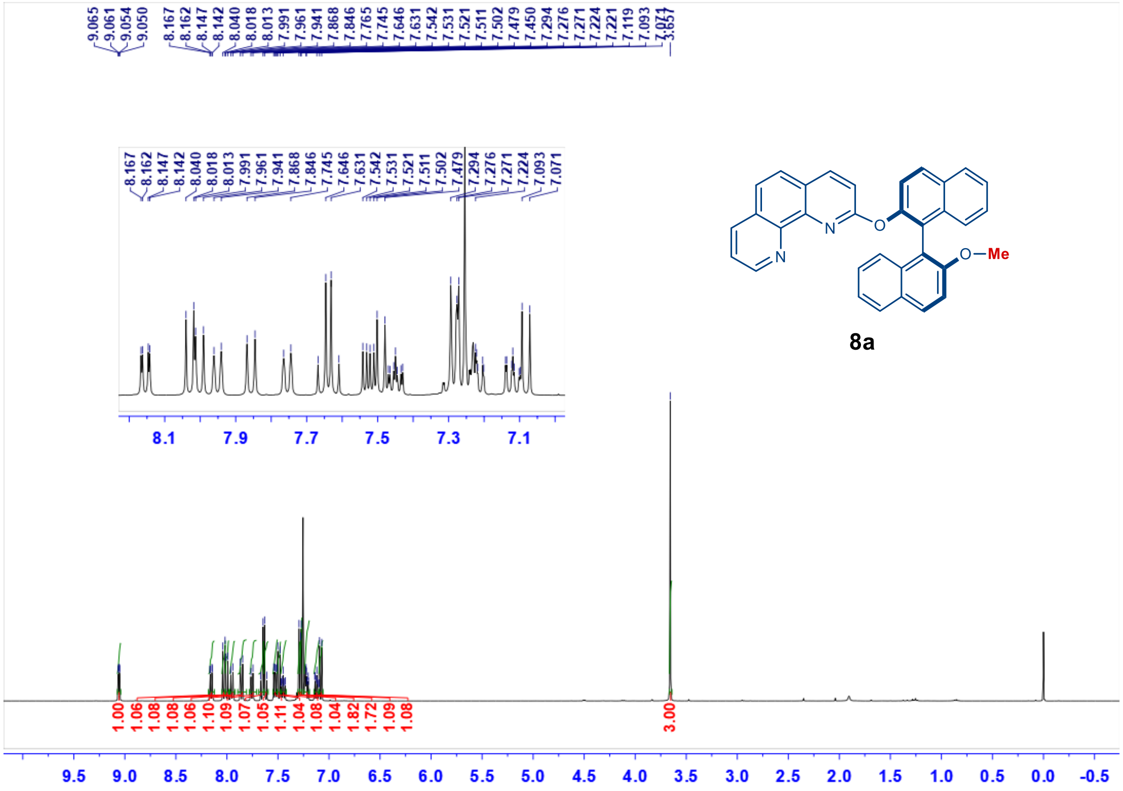

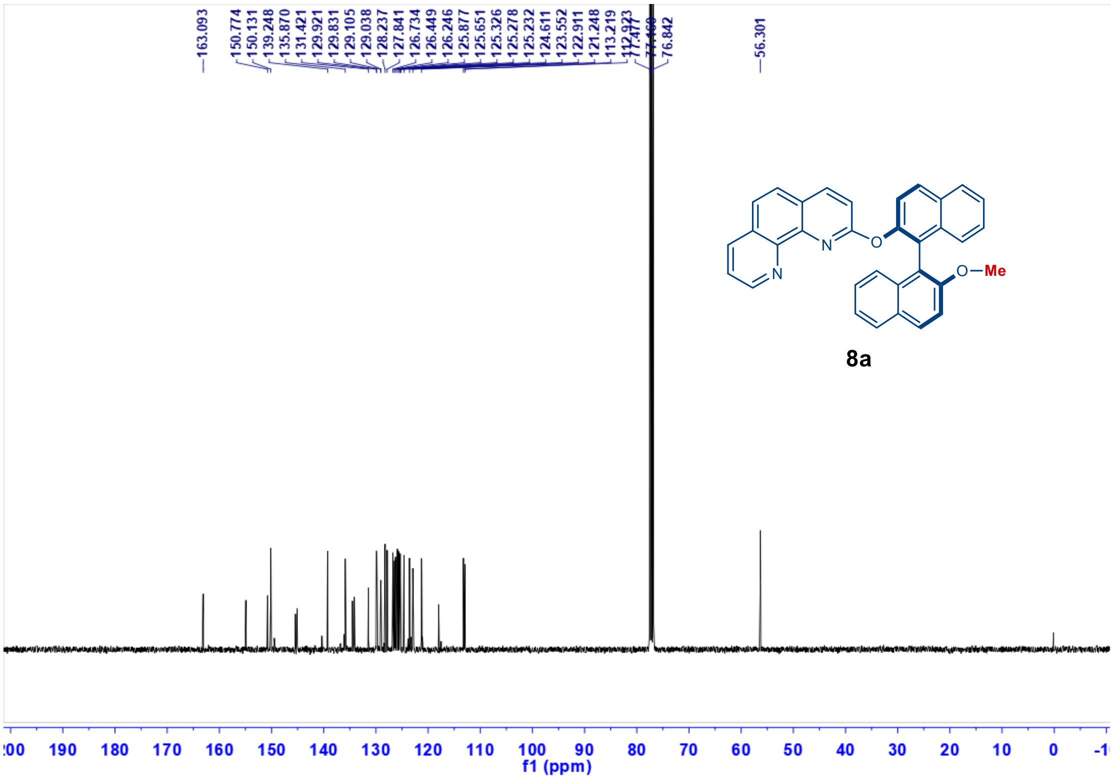

Supplementary Figure 5. ^{1}H and ^{13}C NMR spectra of 8a

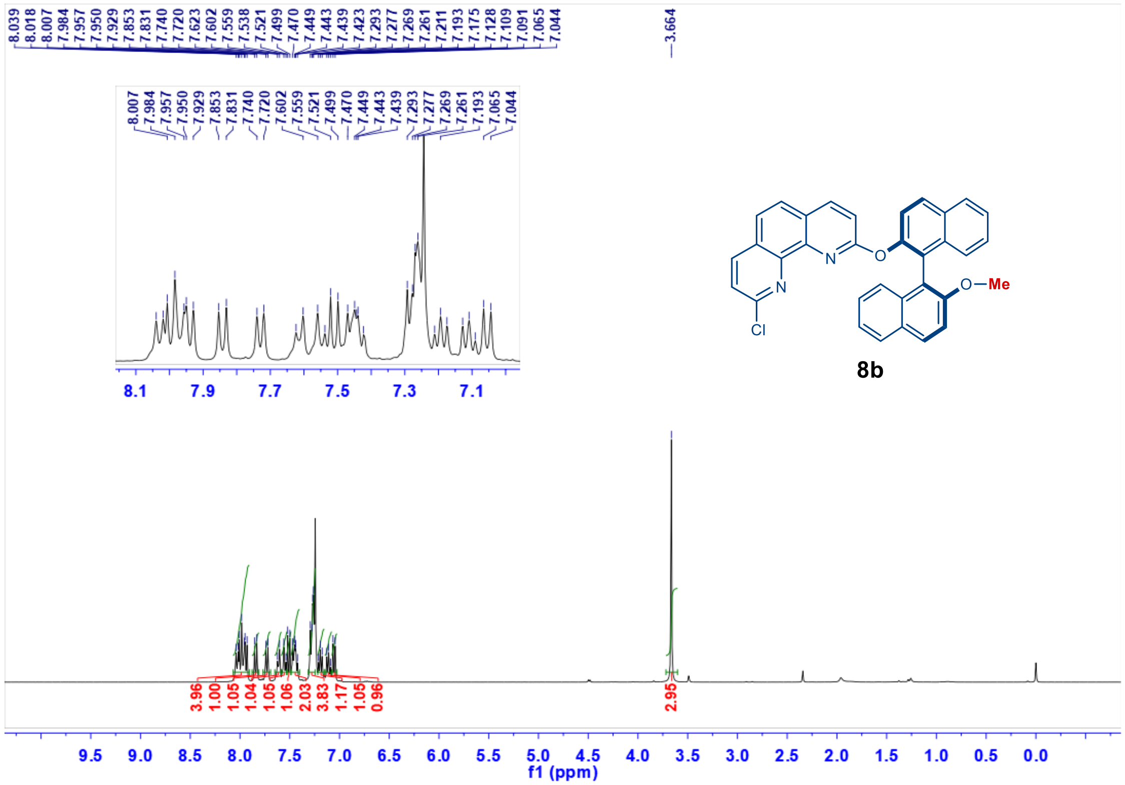

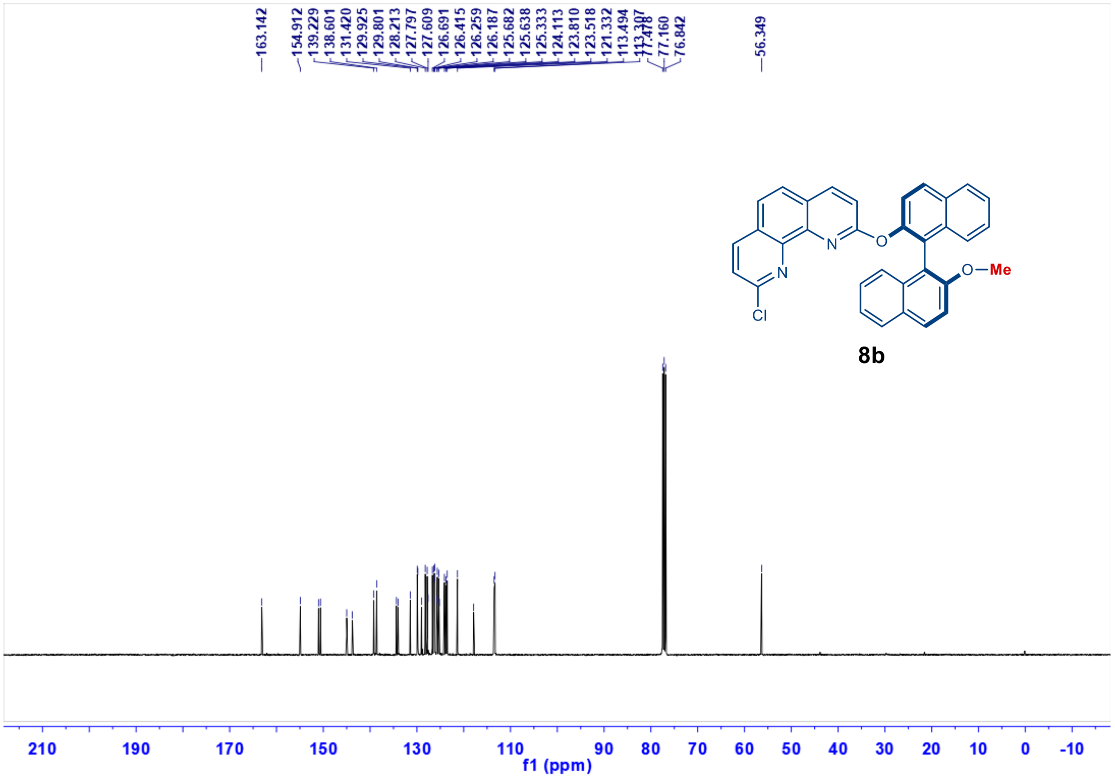

Supplementary Figure 6. ^{1}H and ^{13}C NMR spectra of 8b

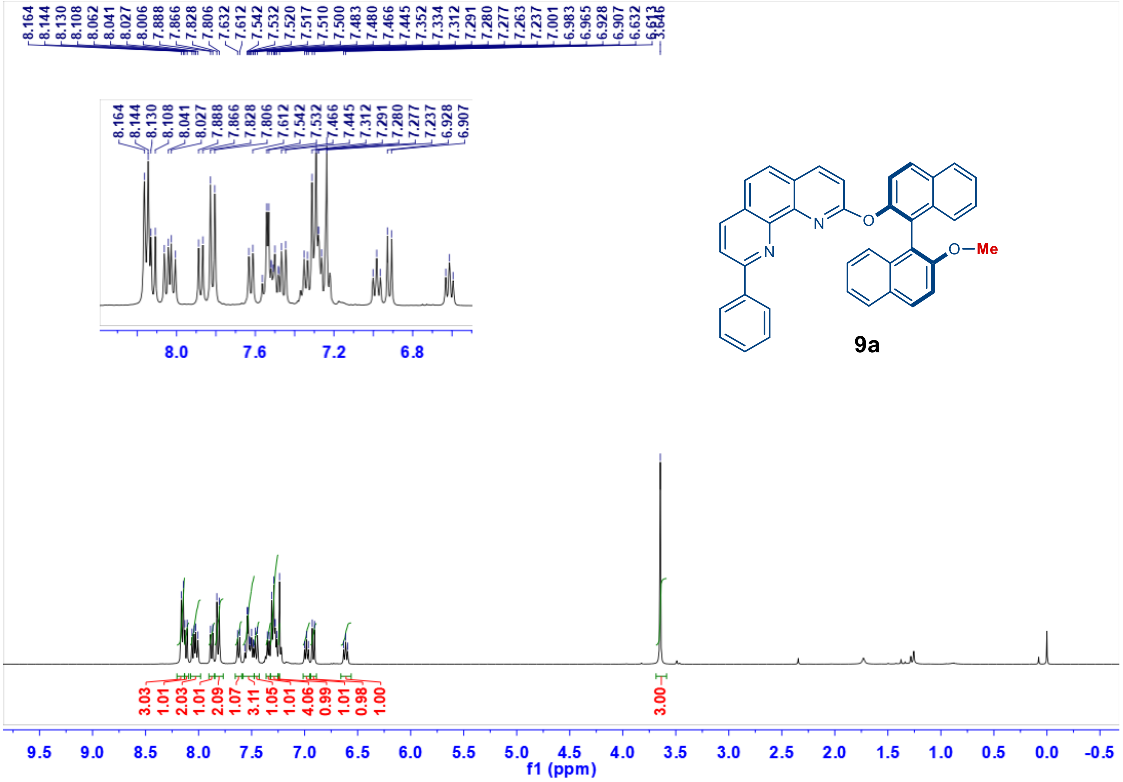

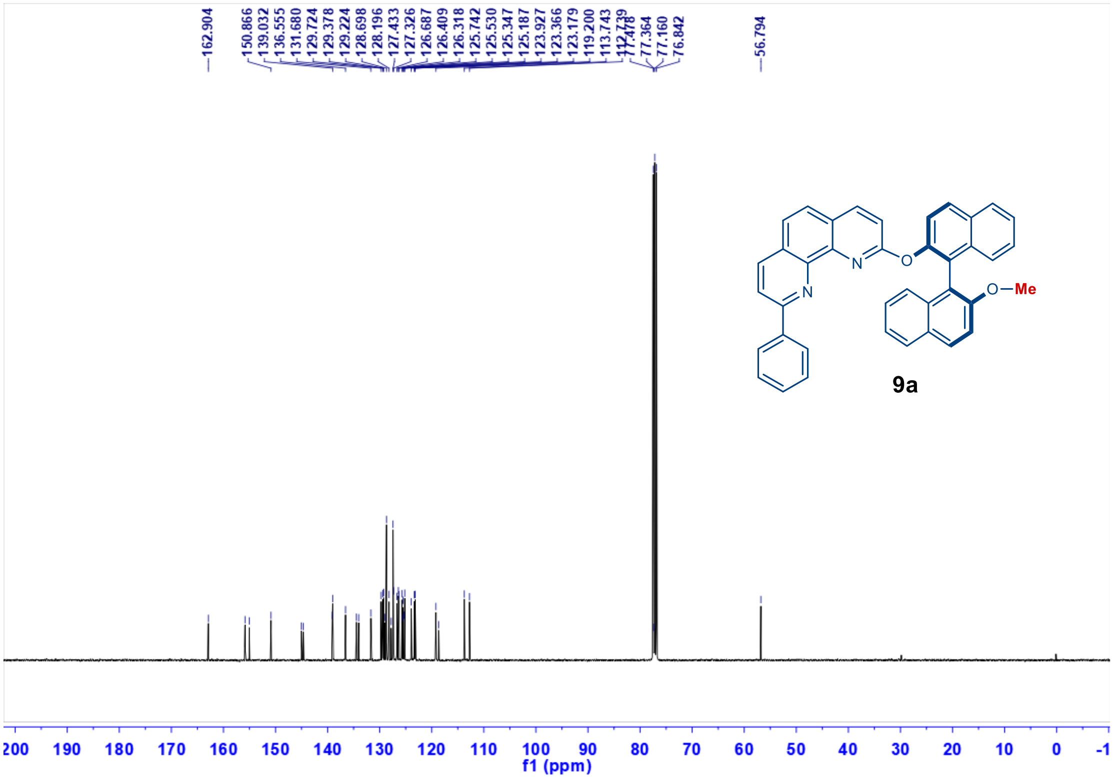

Supplementary Figure 7. ^{1}H and ^{13}C NMR spectra of 9a

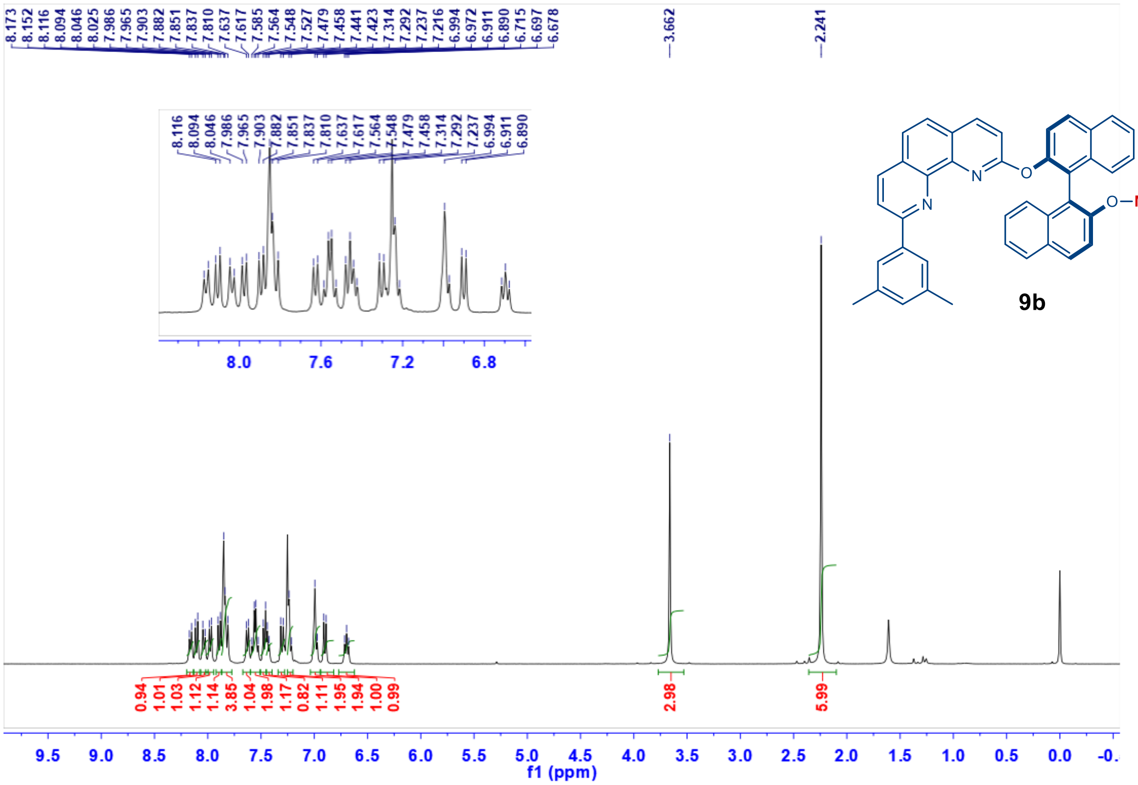

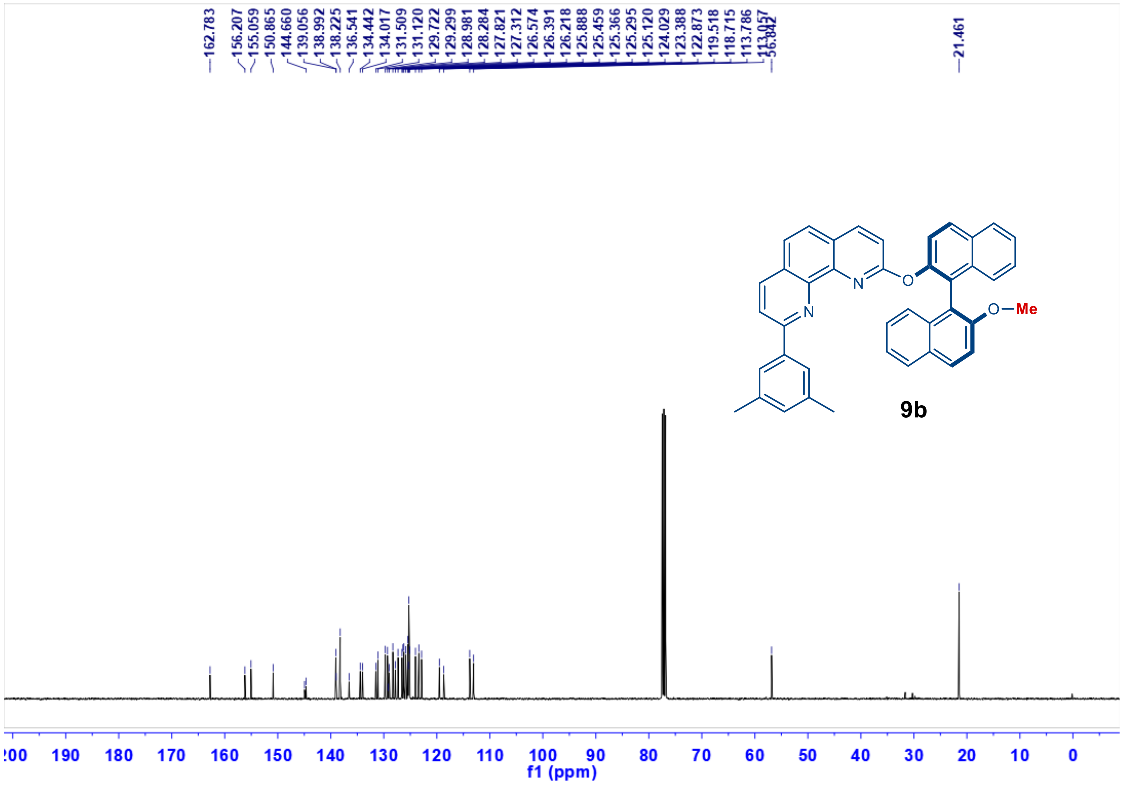

Supplementary Figure 8. ^{1}H and ^{13}C NMR spectra of 9b

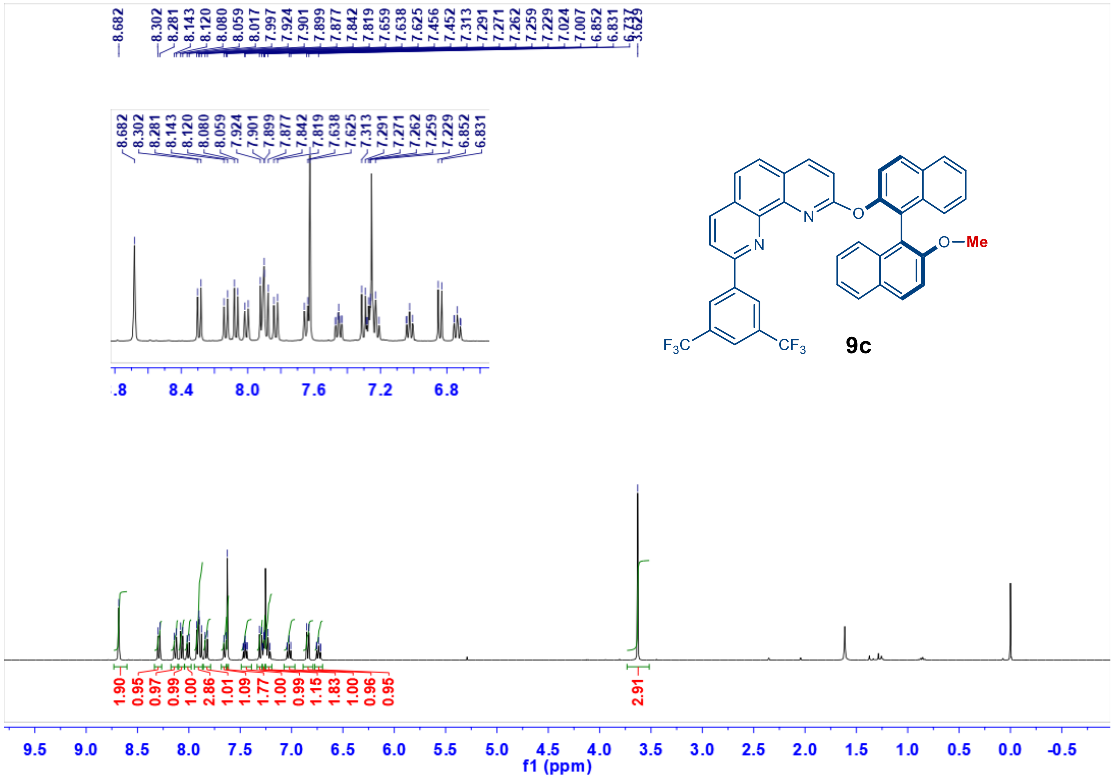

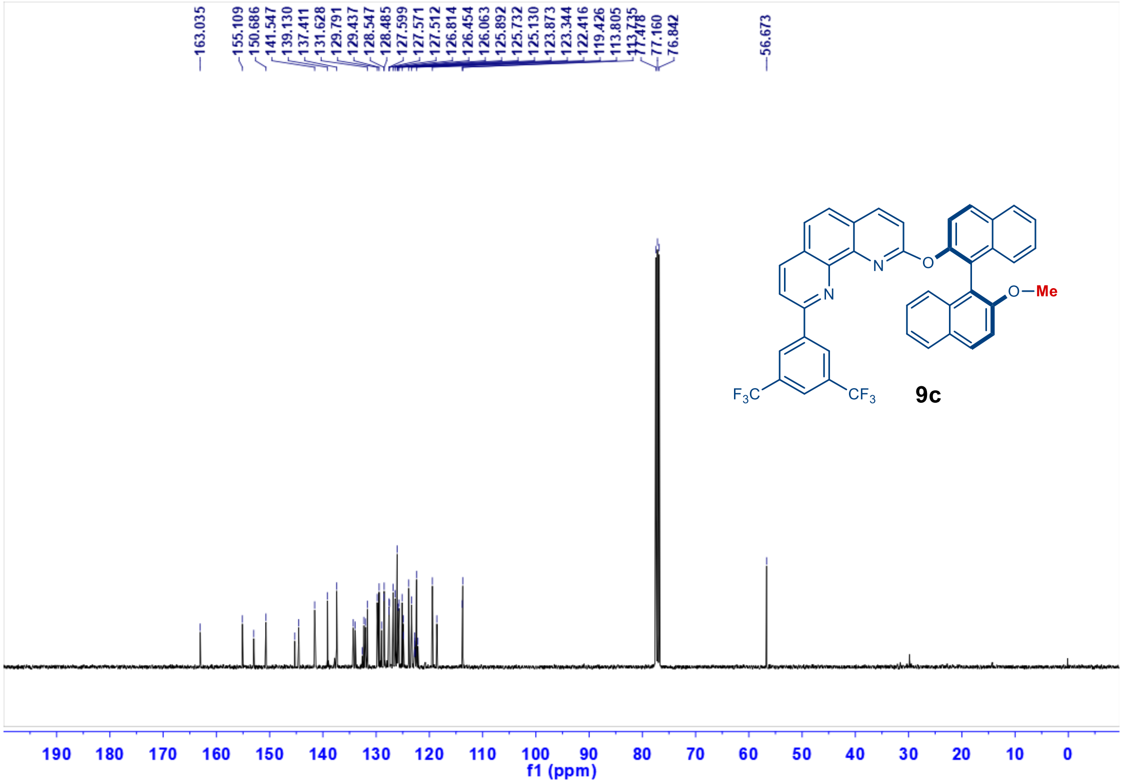

Supplementary Figure 9. ^{1}H and ^{13}C NMR spectra of 9c

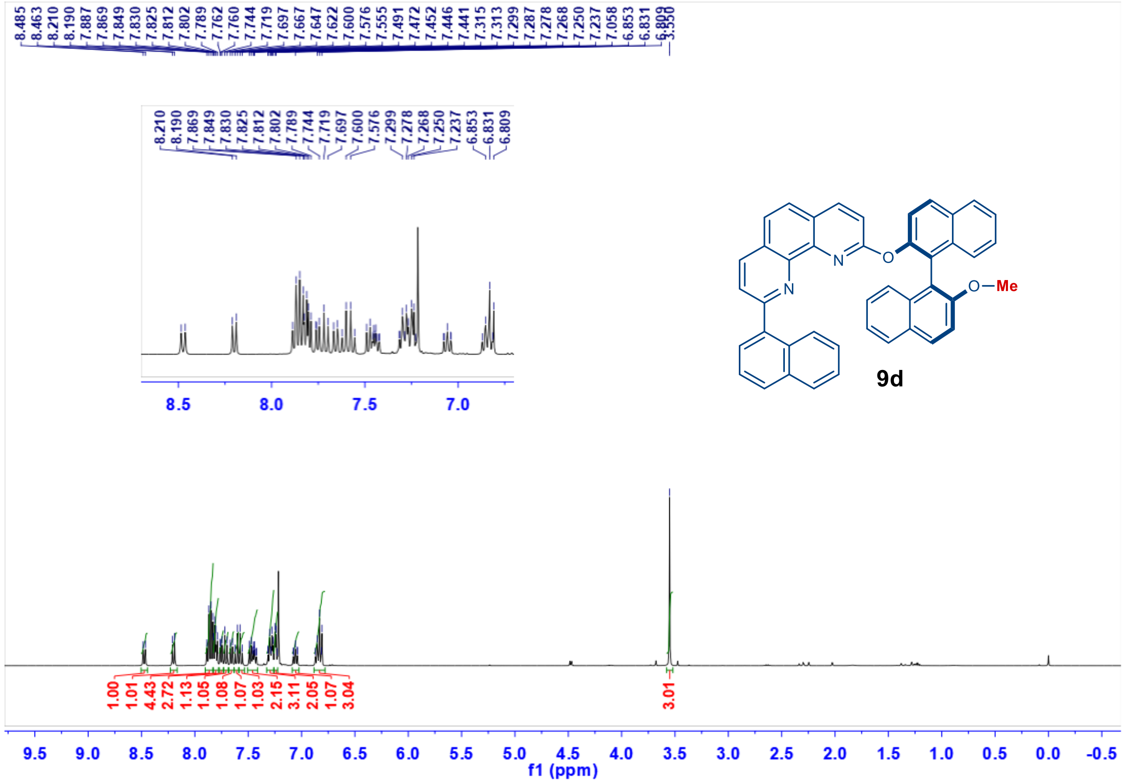

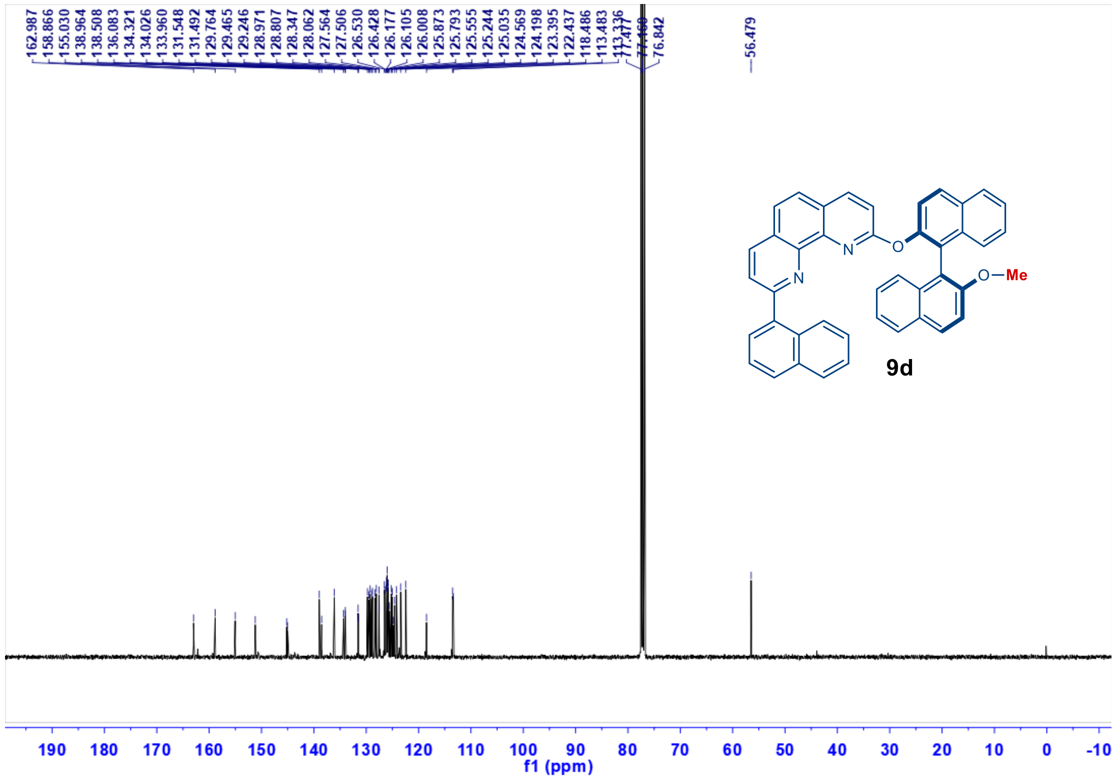

Supplementary Figure 10. ^{1}H and ^{13}C NMR spectra of 9d

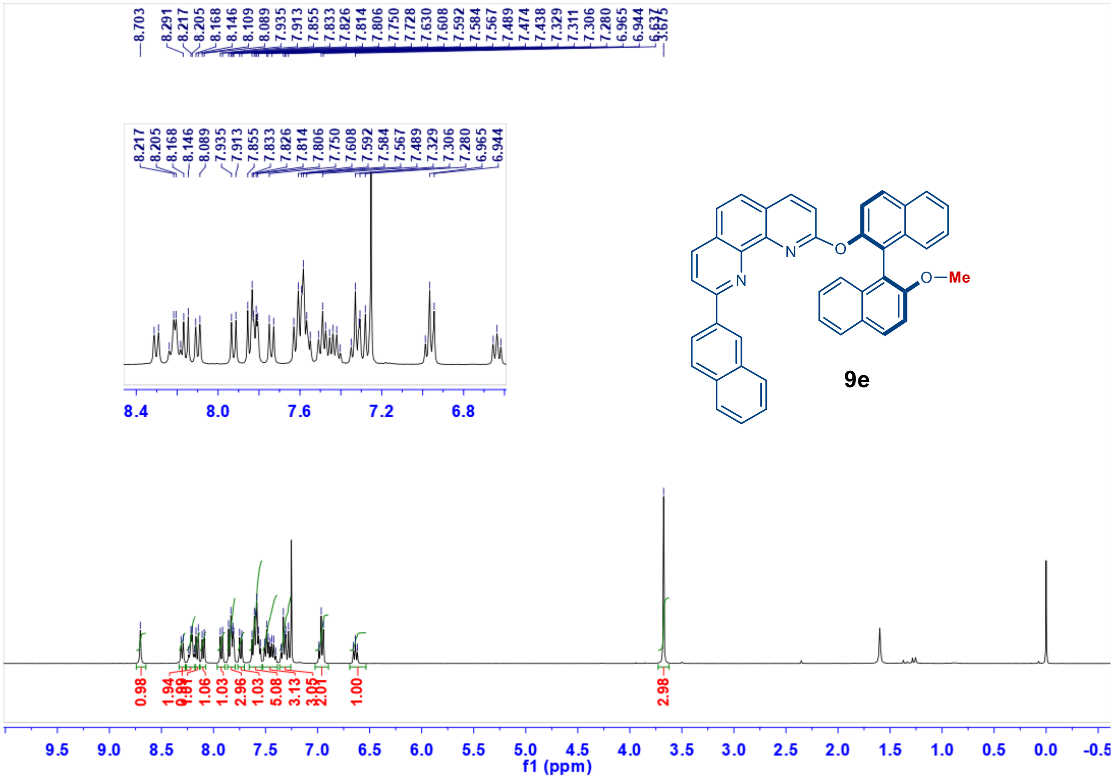

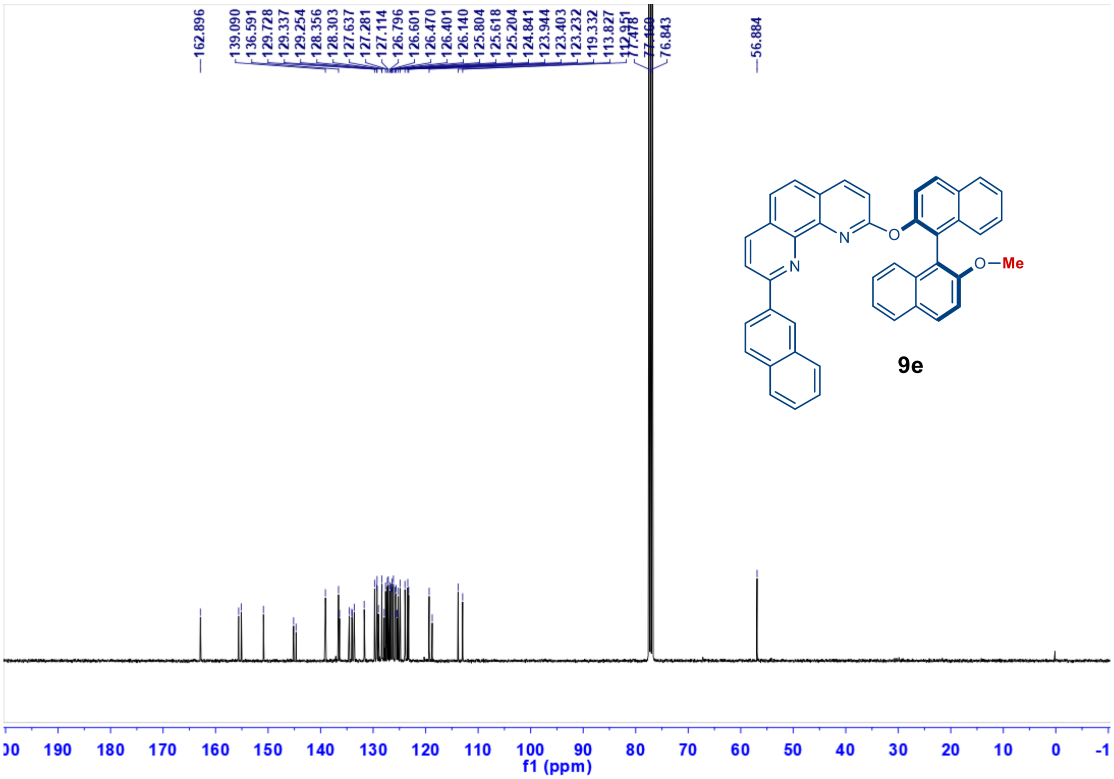

Supplementary Figure 11. ^{1}H and ^{13}C NMR spectra of 9e

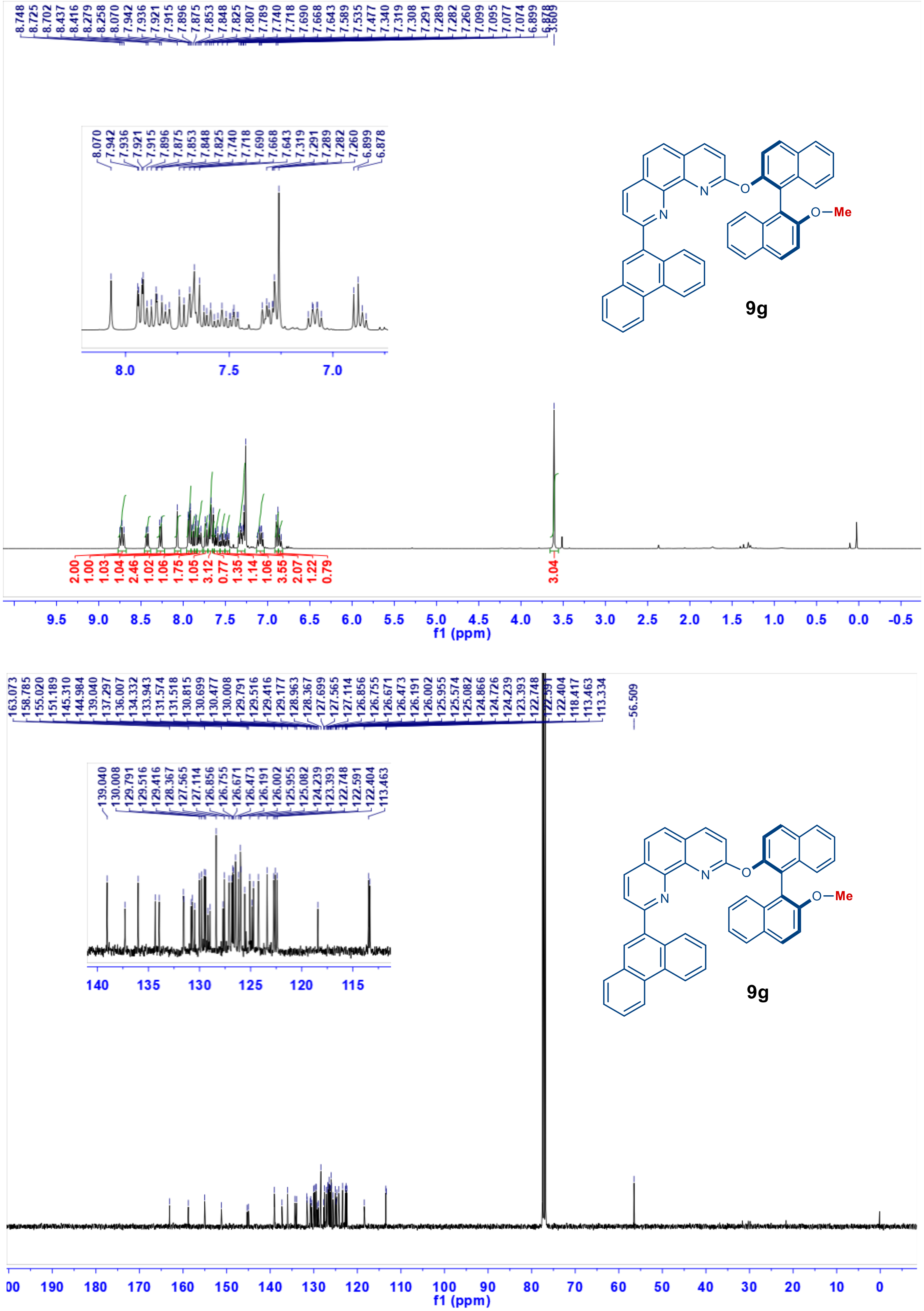

Supplementary Figure 12. ^{1}H **and** ^{13}C **NMR spectra of 9g**

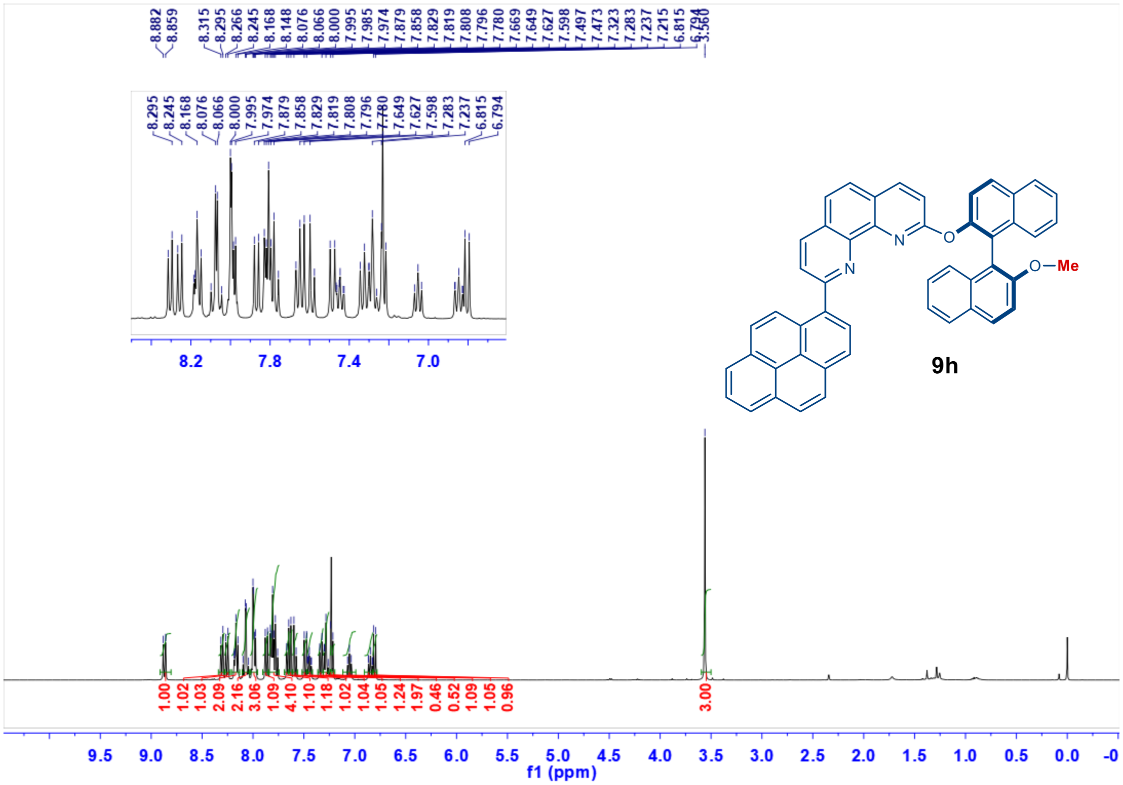

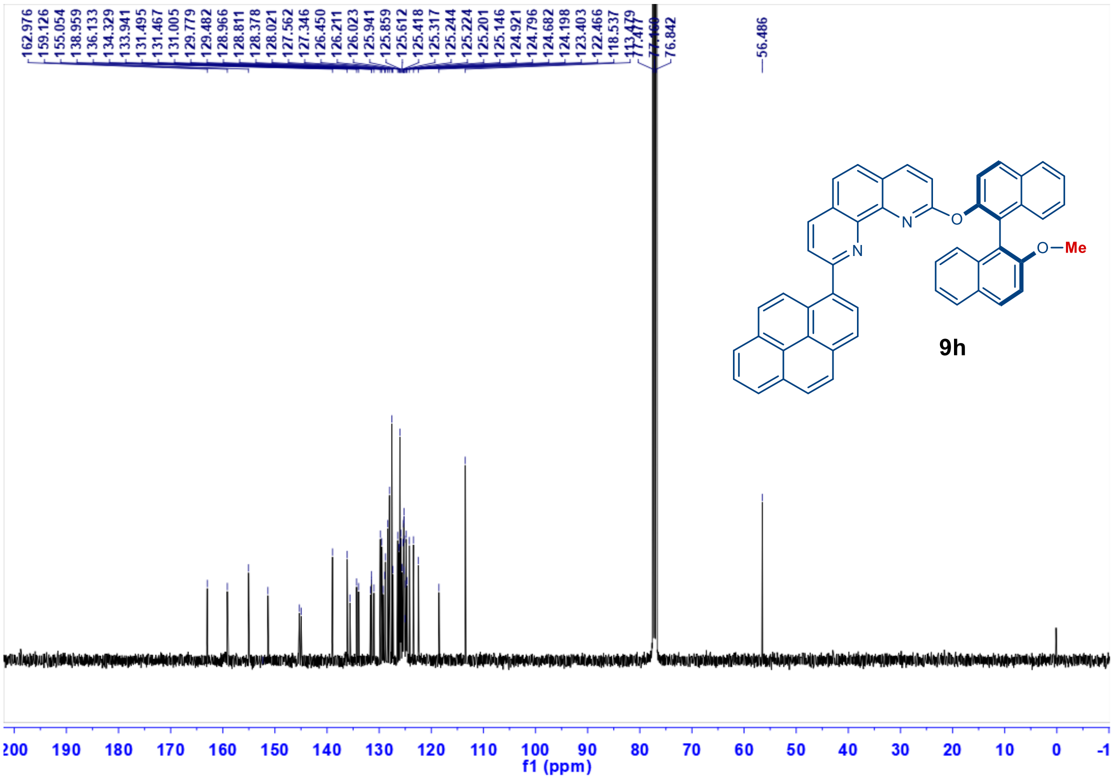

Supplementary Figure 13. ^{1}H and ^{13}C NMR spectra of 9h

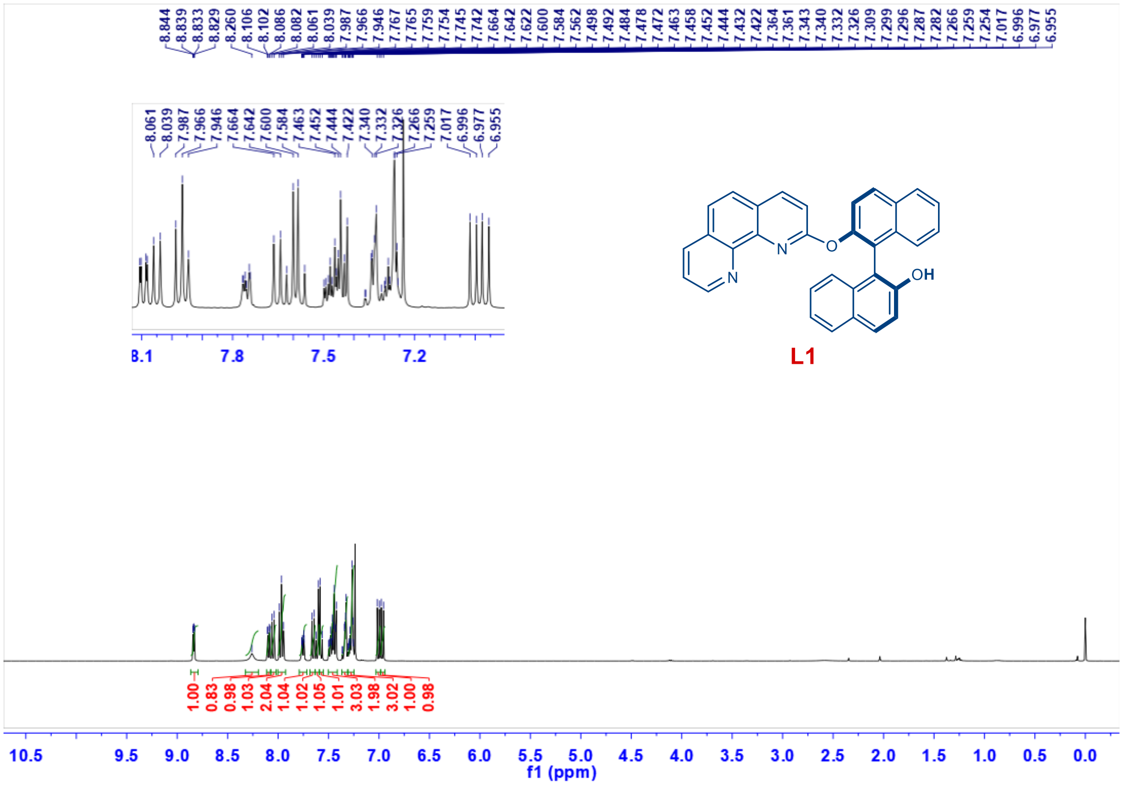

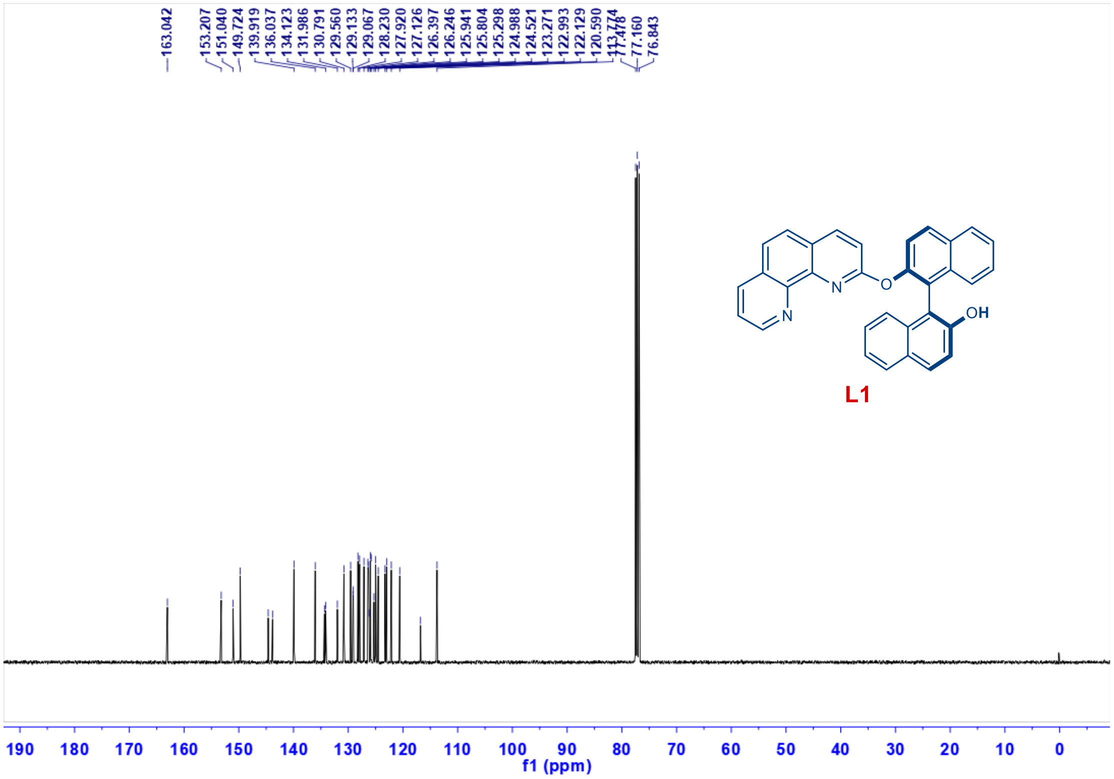

Supplementary Figure 14. ^{1}H and ^{13}C NMR spectra of L1

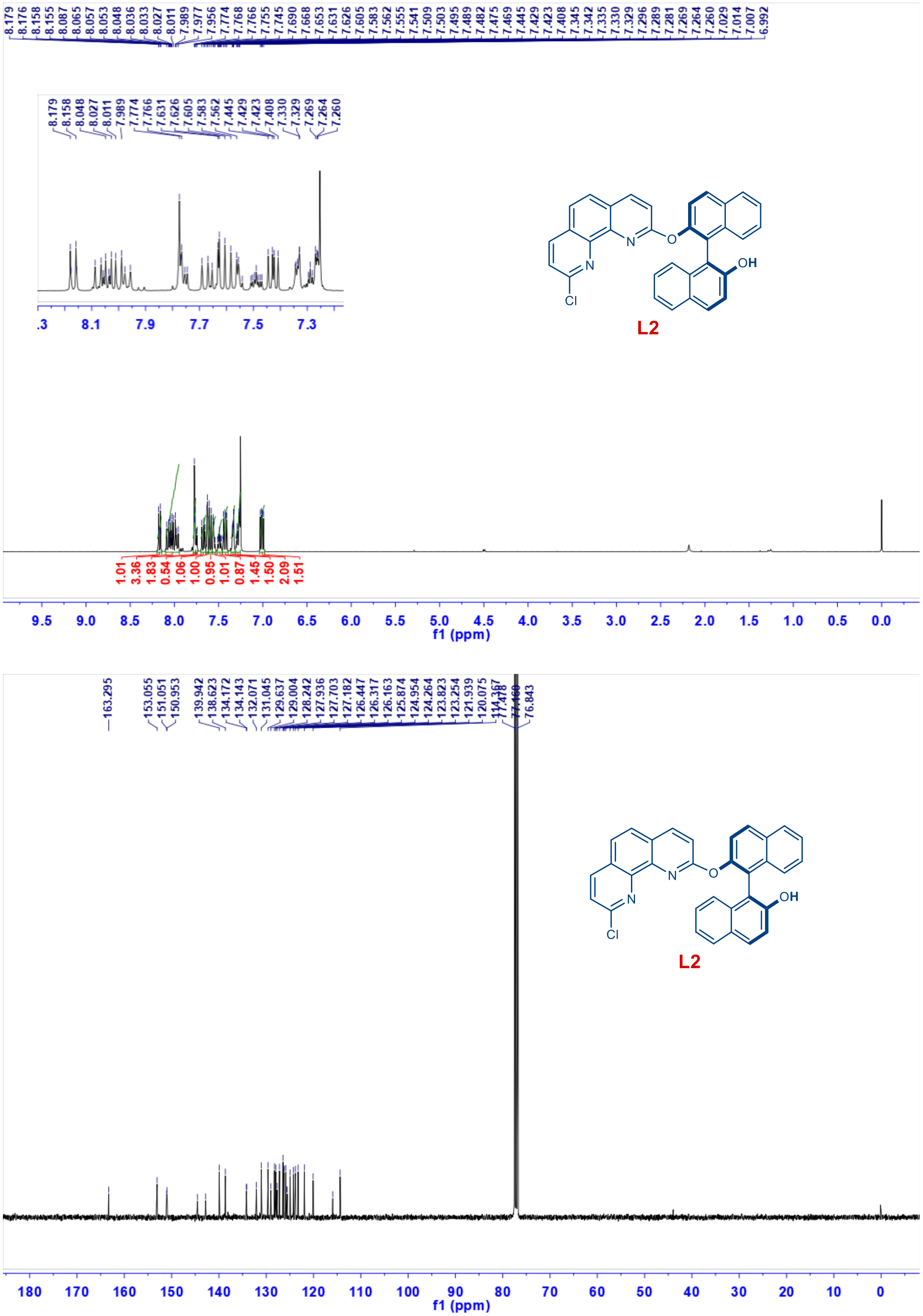

Supplementary Figure 15. ^{1}H and ^{13}C NMR spectra of L2

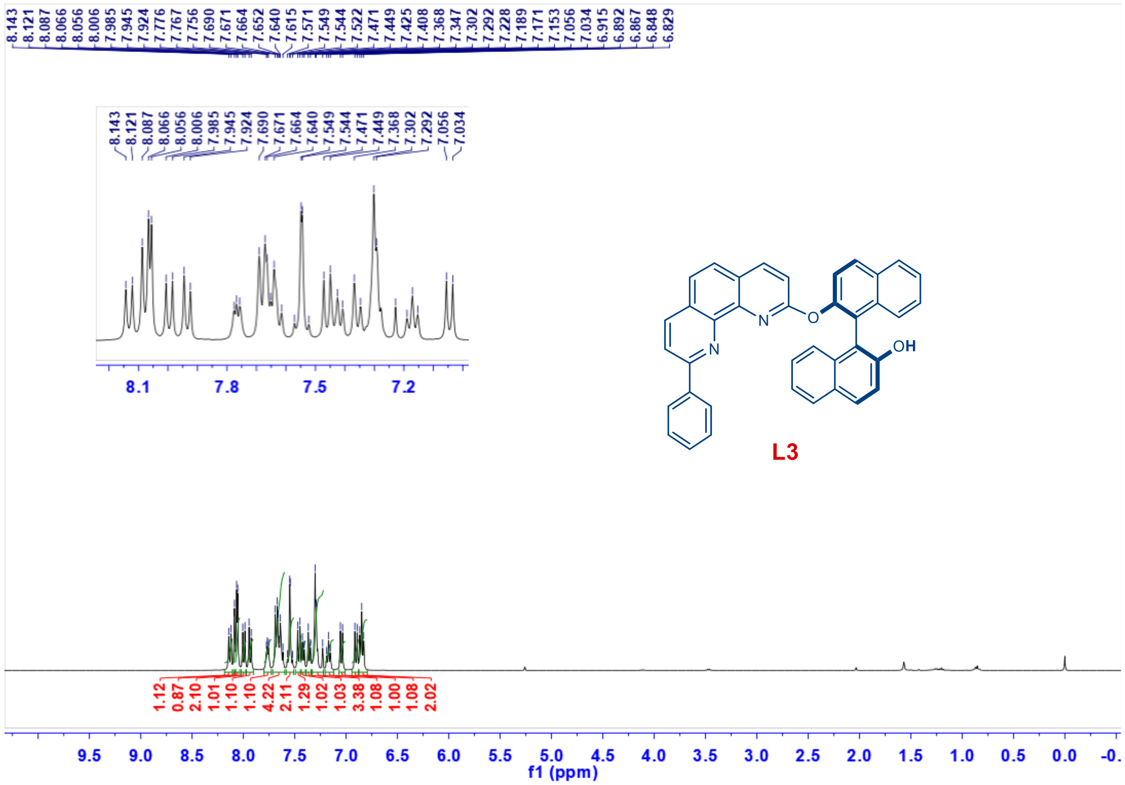

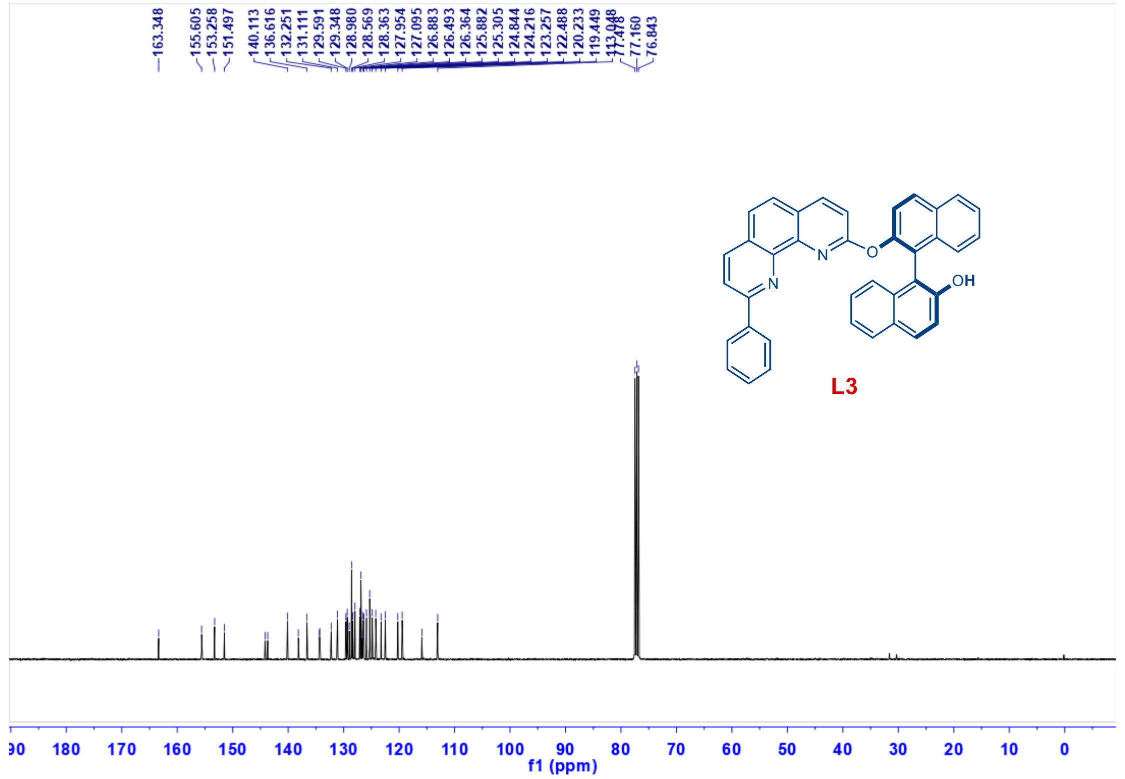

Supplementary Figure 16. ^{1}H and ^{13}C NMR spectra of L3

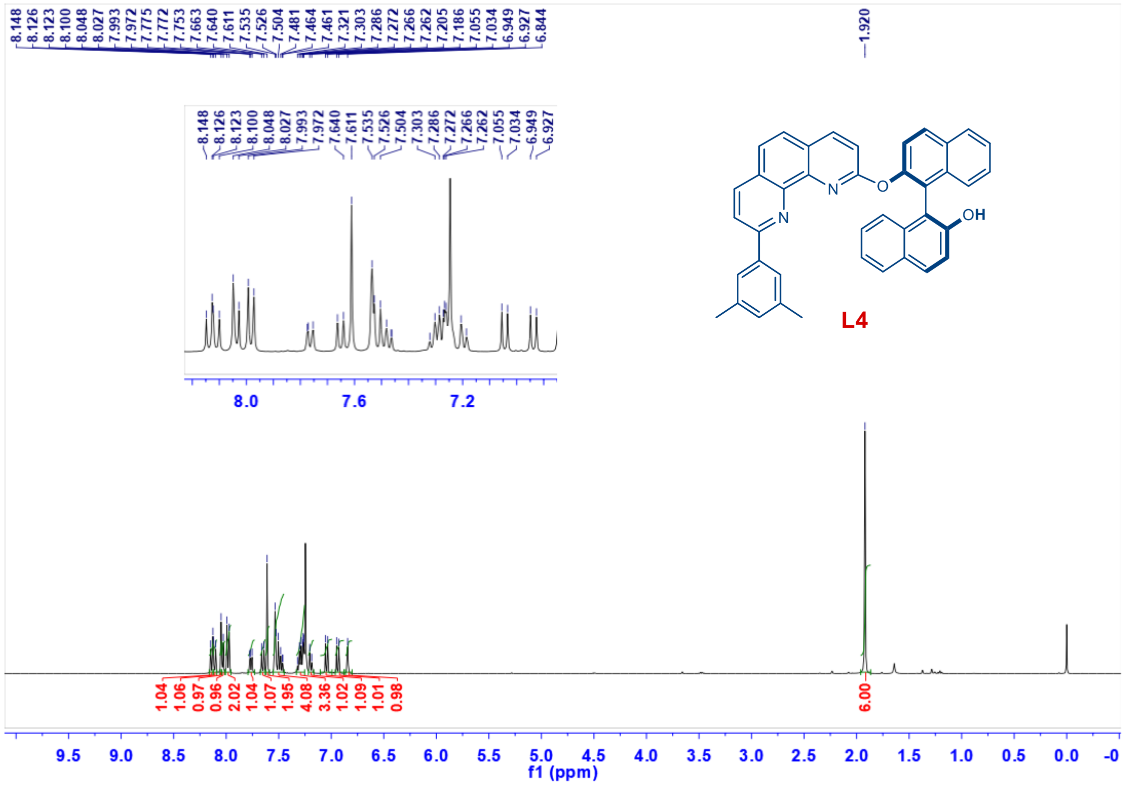

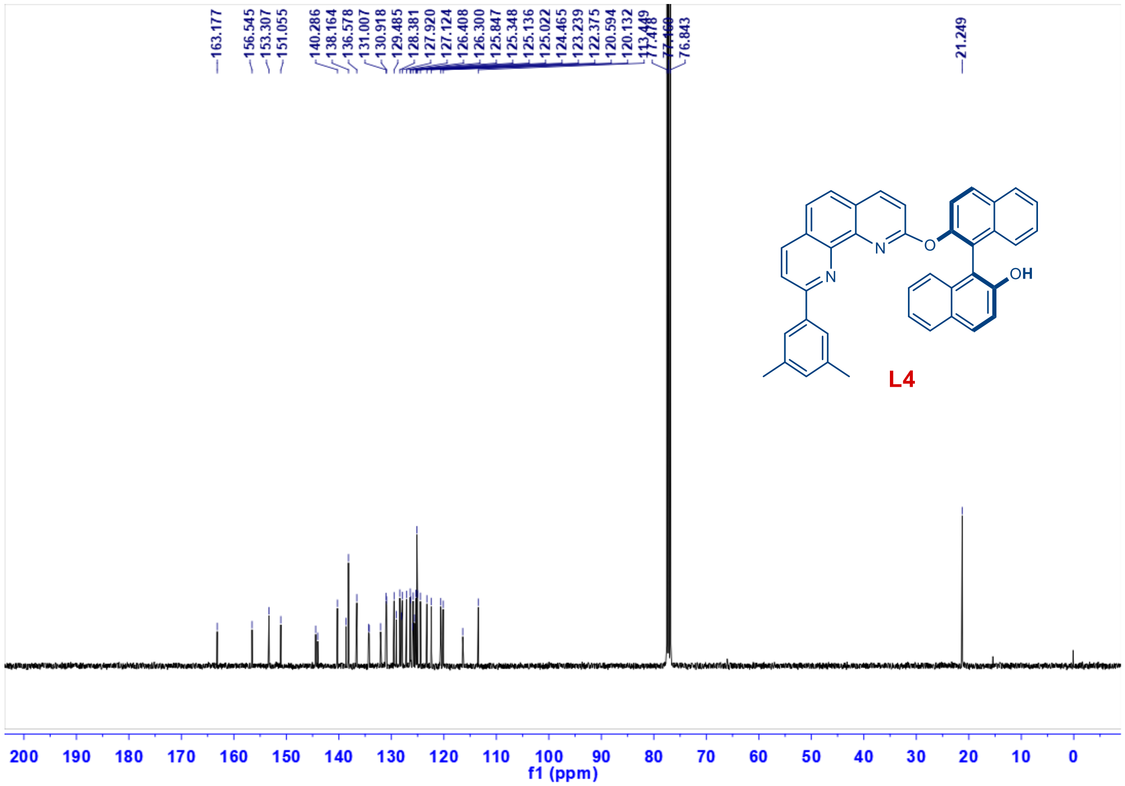

Supplementary Figure 17. ^{1}H and ^{13}C NMR spectra of L4

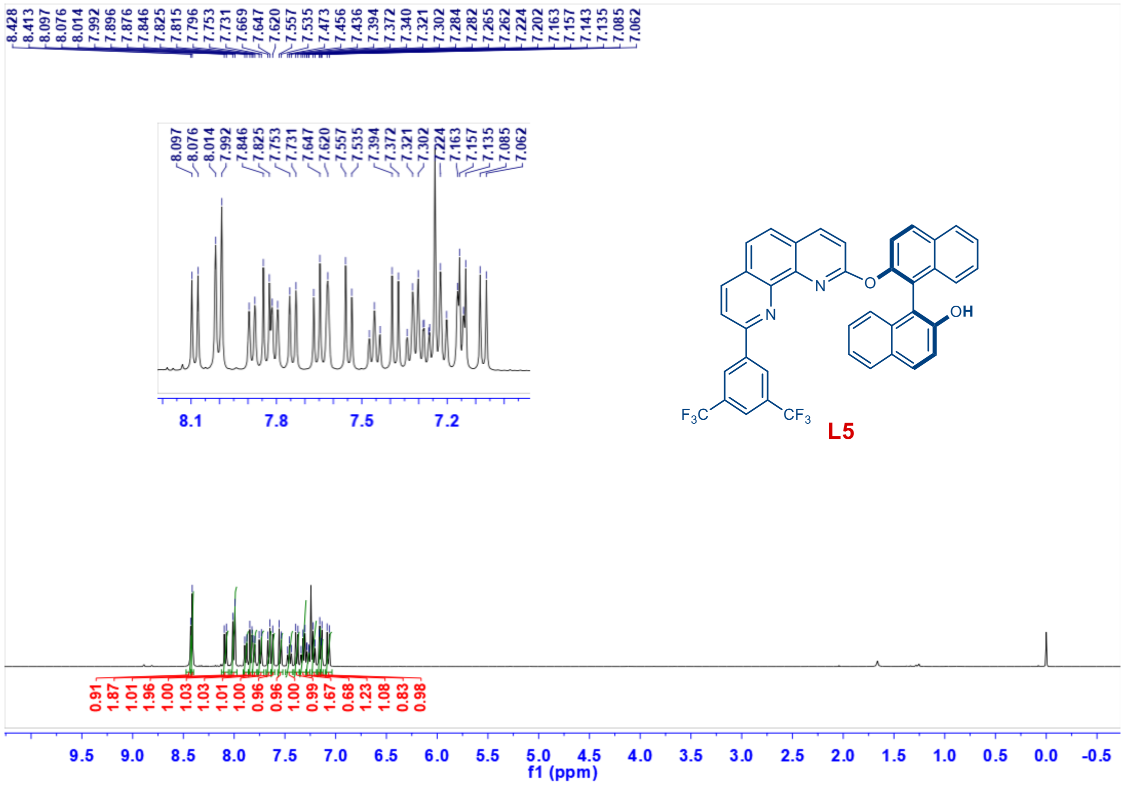

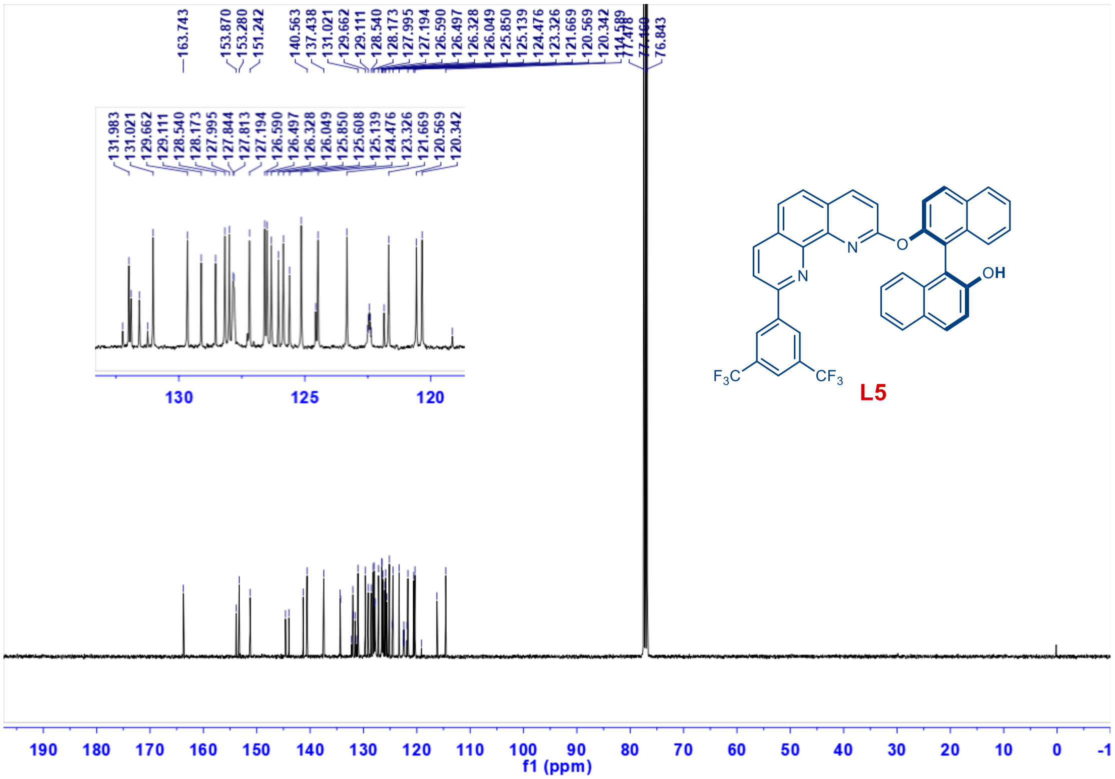

Supplementary Figure 18. ^{1}H **and** ^{13}C **NMR spectra of L5**

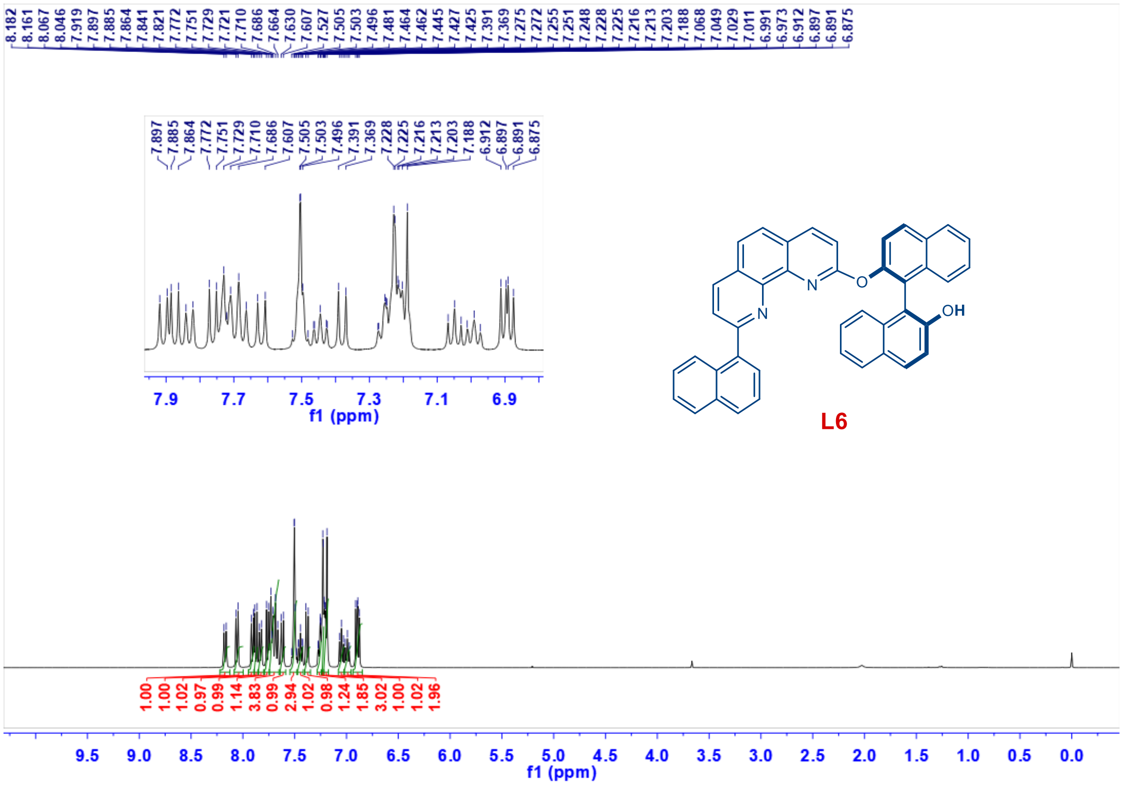

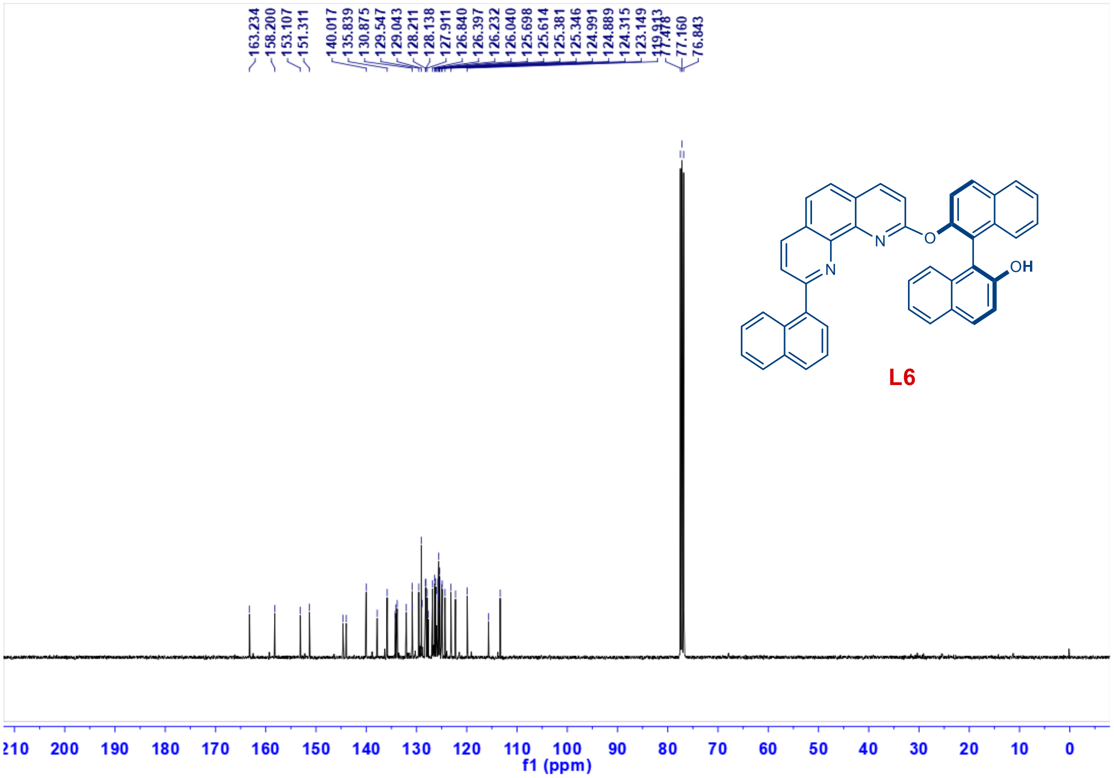

Supplementary Figure 19. ^{1}H and ^{13}C NMR spectra of L6

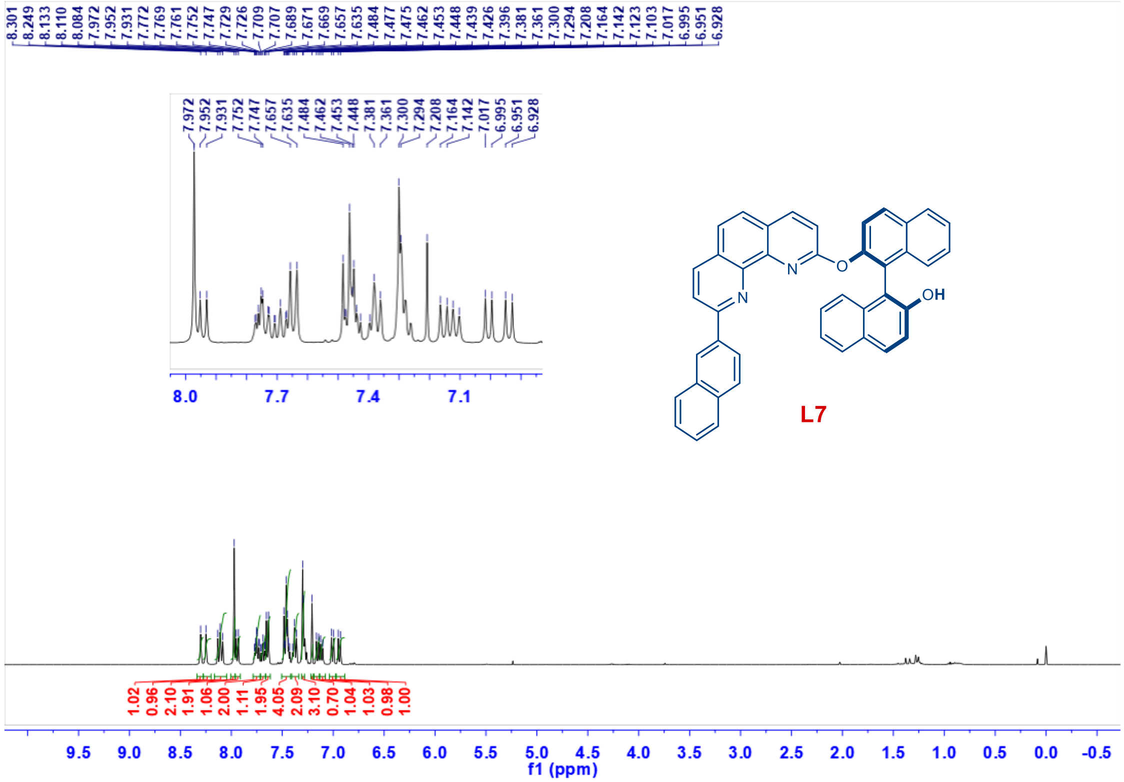

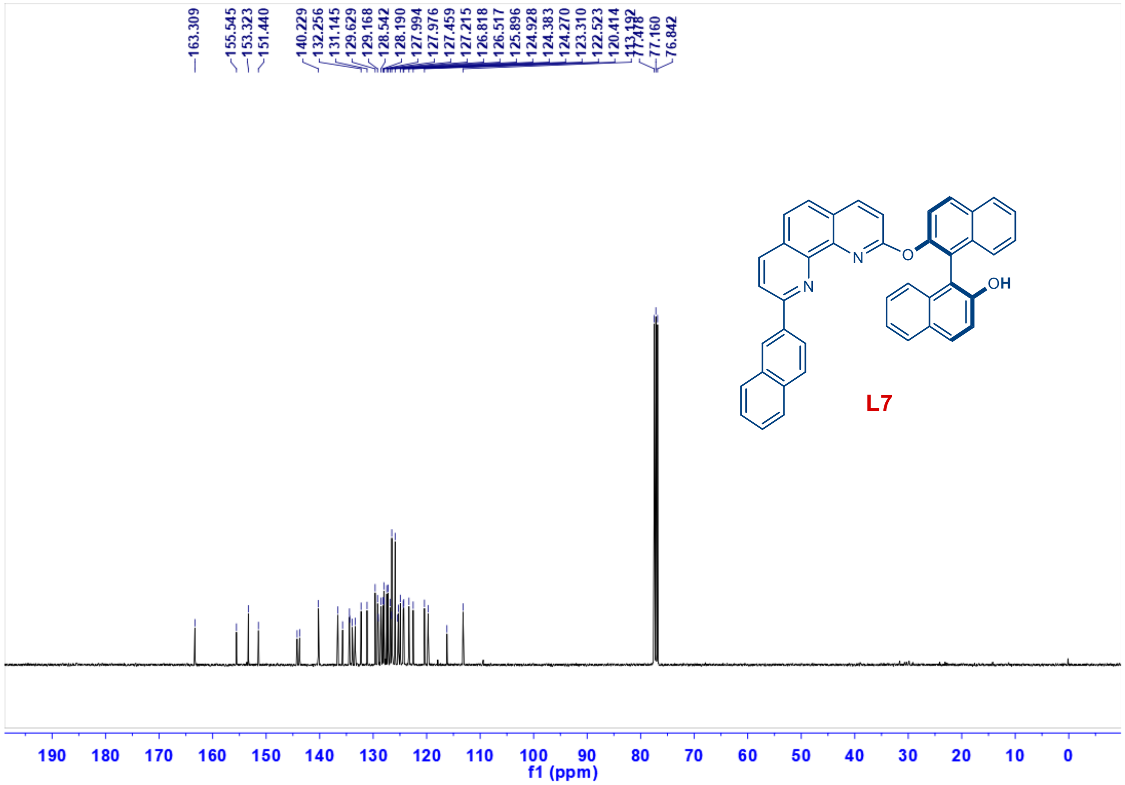

Supplementary Figure 20. ^{1}H and ^{13}C NMR spectra of L7

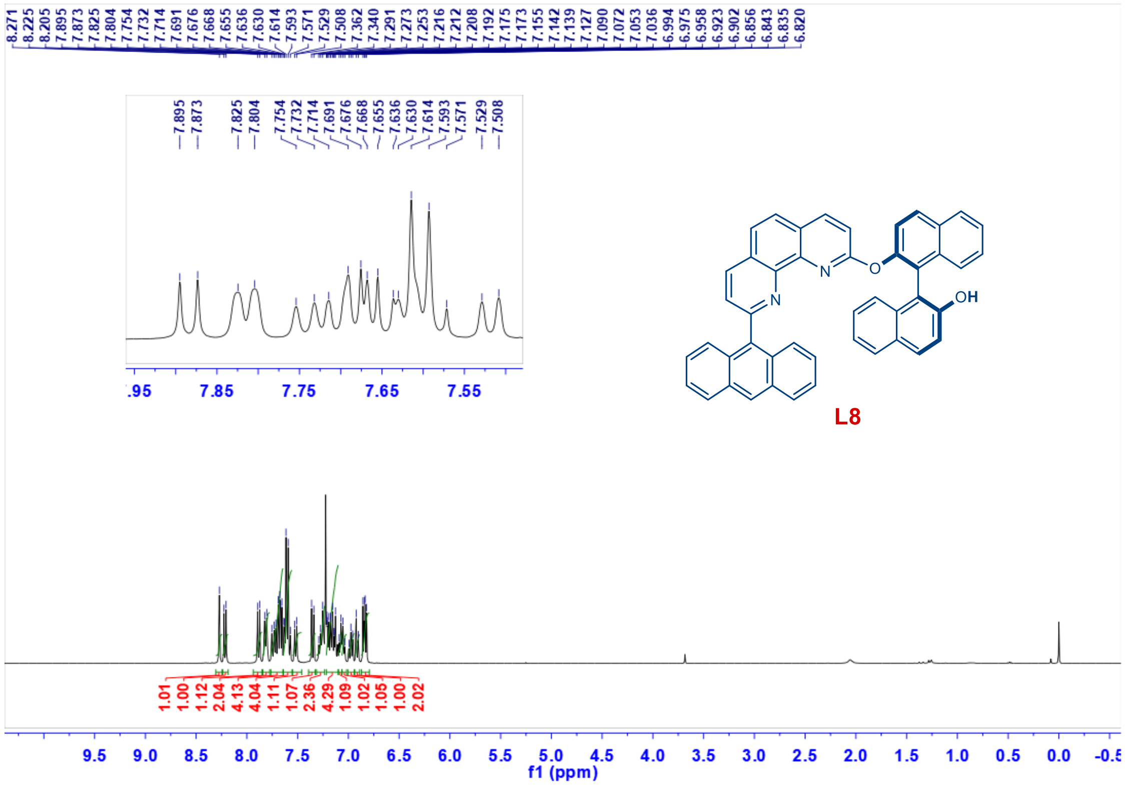

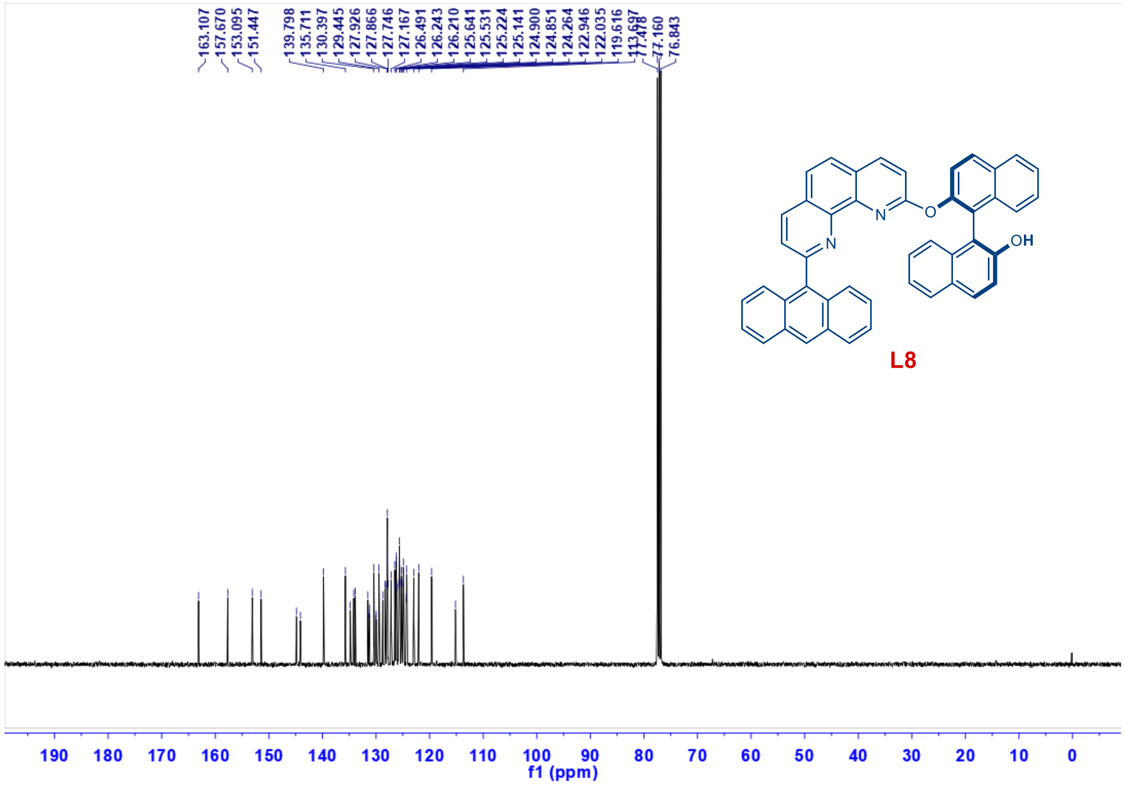

Supplementary Figure 21. ^{1}H and ^{13}C NMR spectra of L8

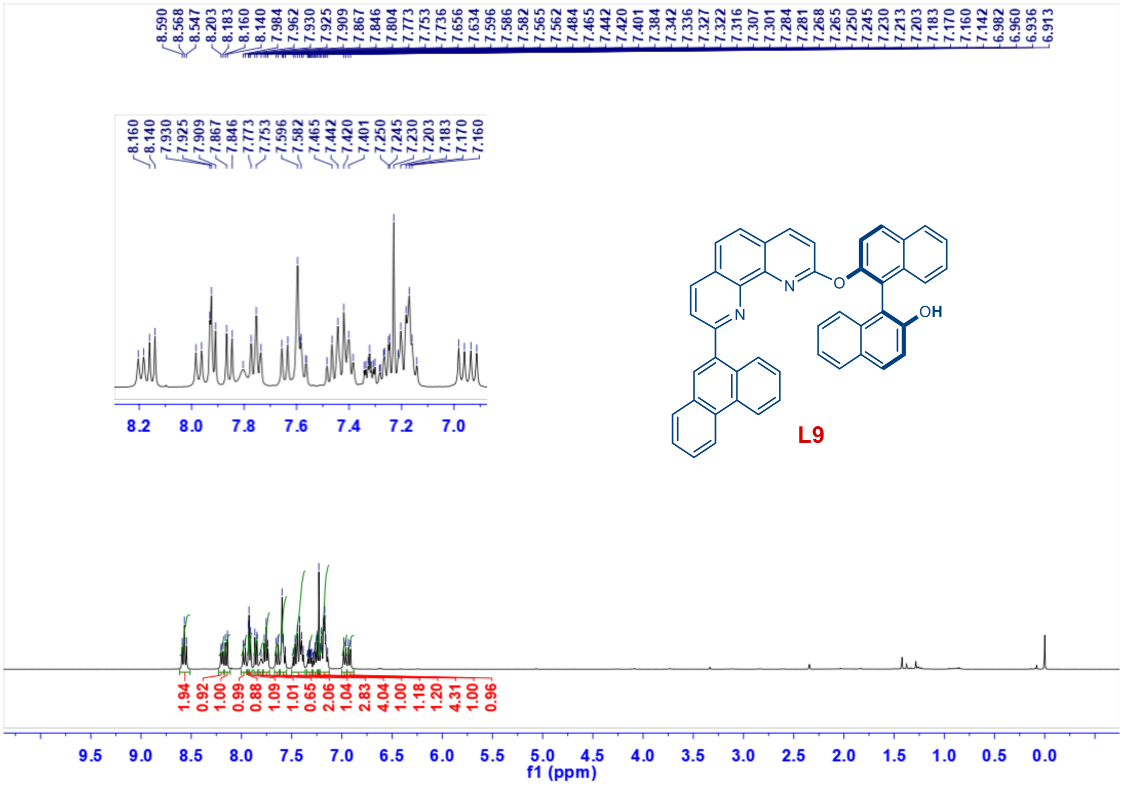

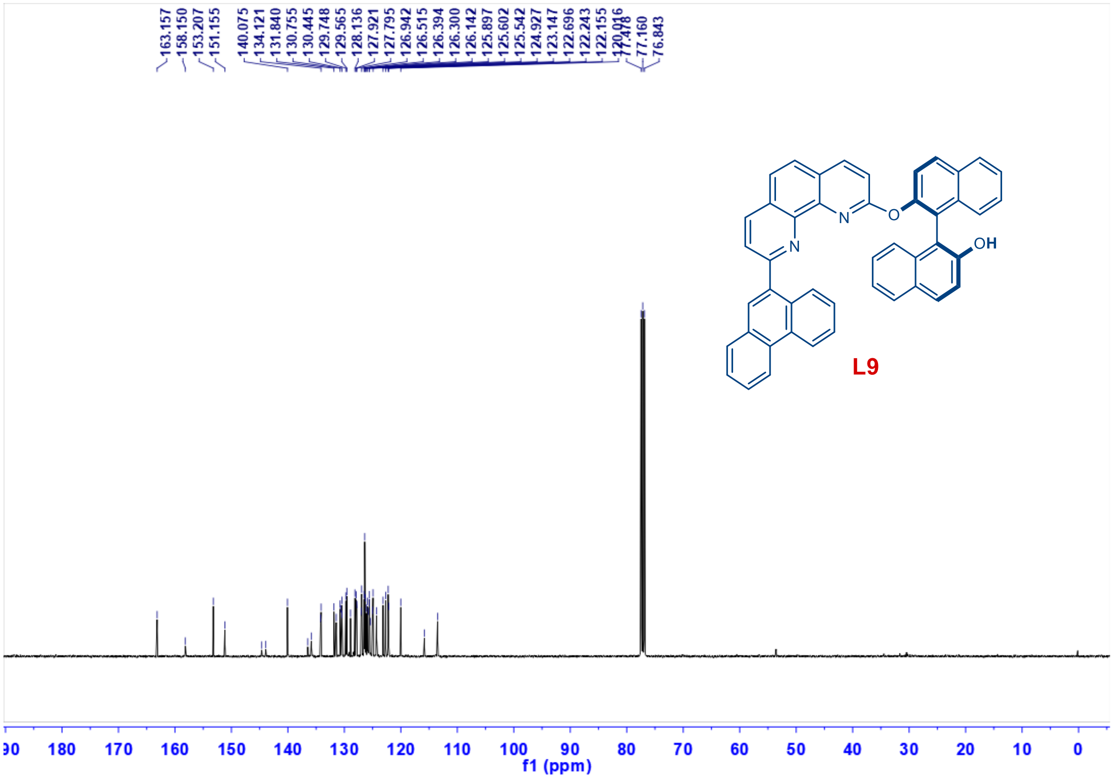

Supplementary Figure 22. ^{1}H and ^{13}C NMR spectra of L9

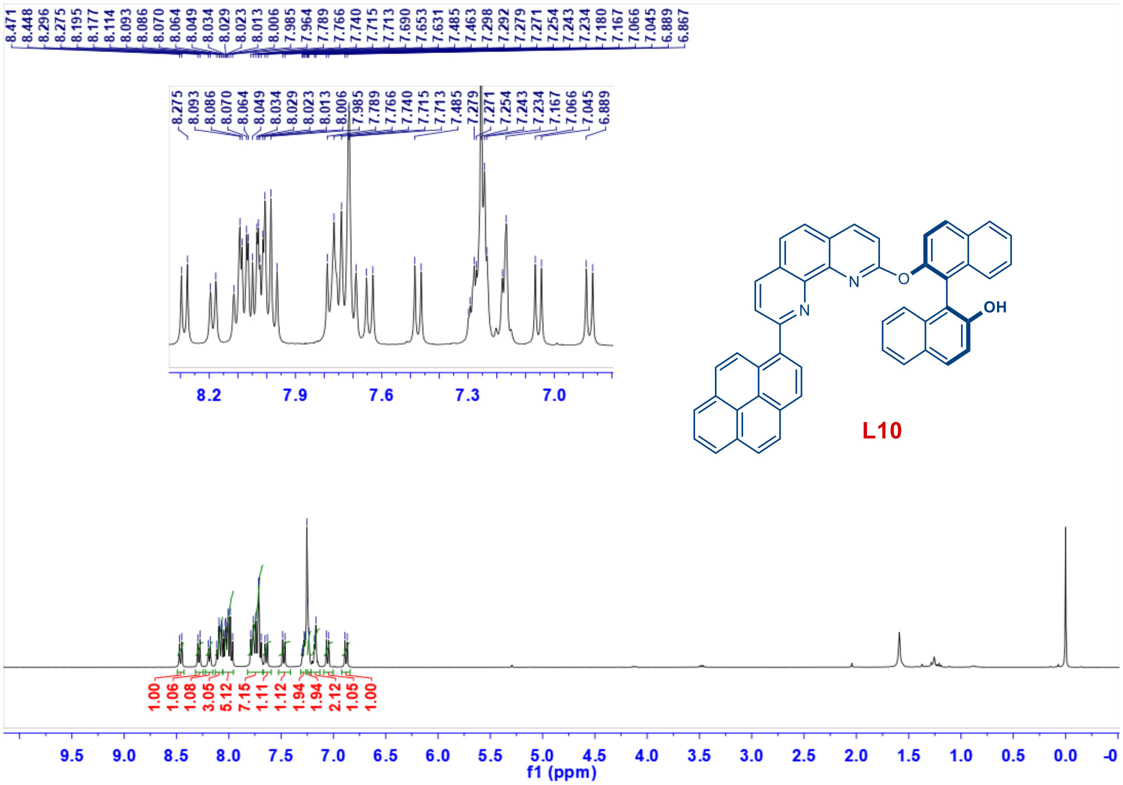

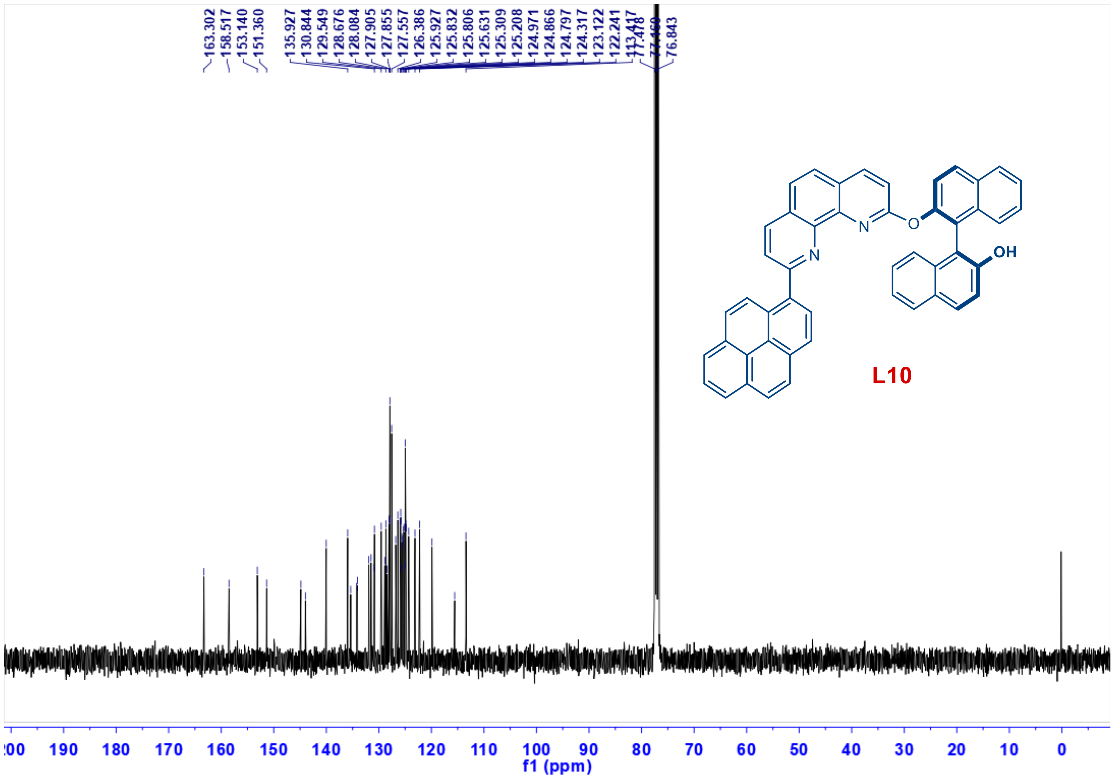

Supplementary Figure 23. ^{1}H and ^{13}C NMR spectra of L10

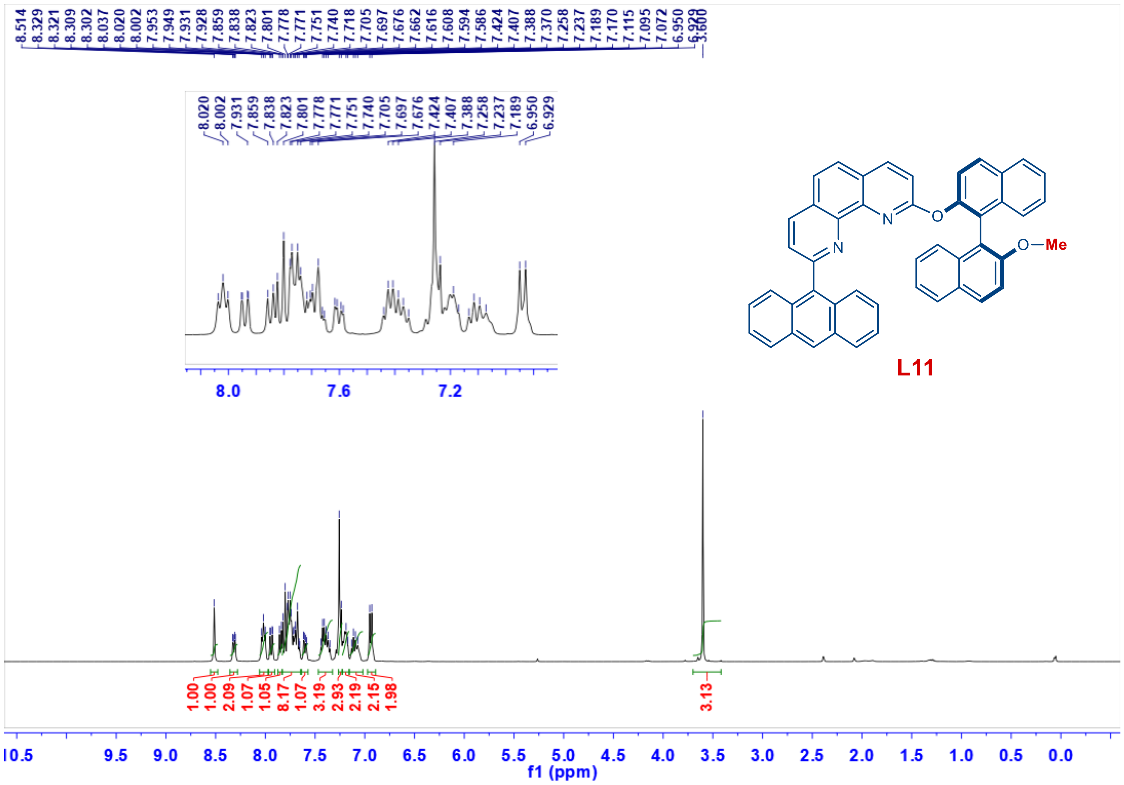

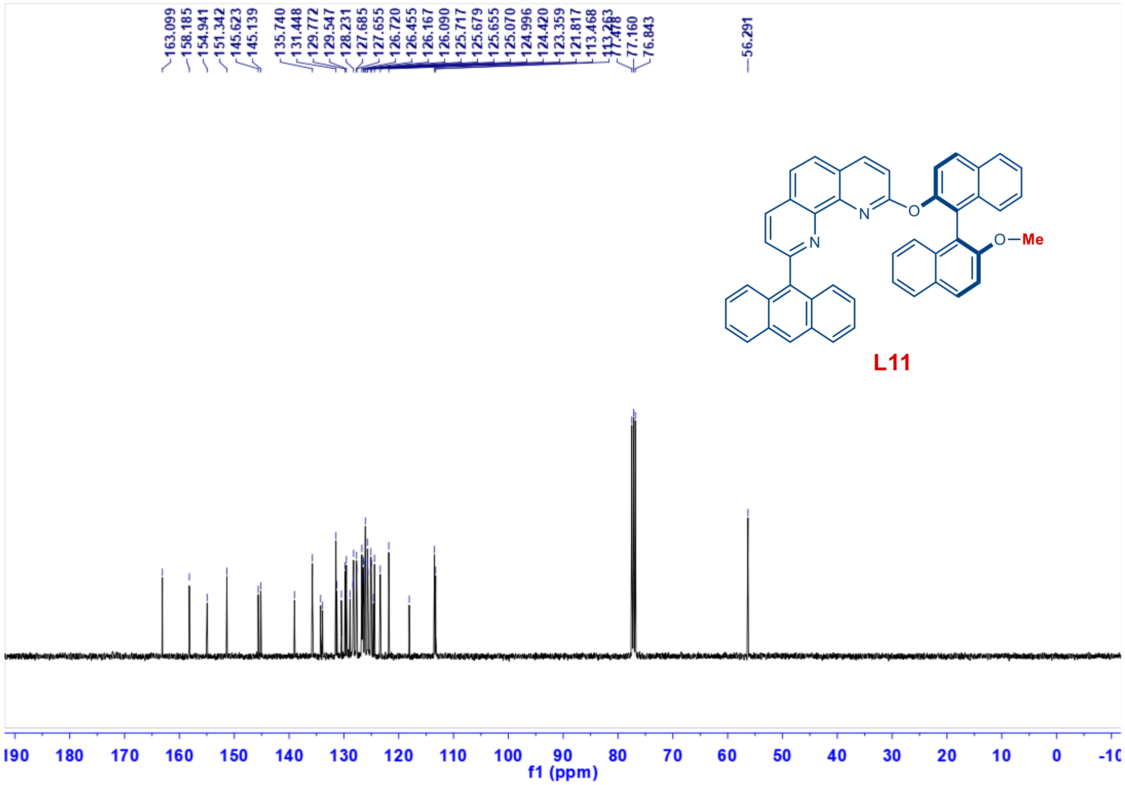

Supplementary Figure 24. ^{1}H and ^{13}C NMR spectra of L11

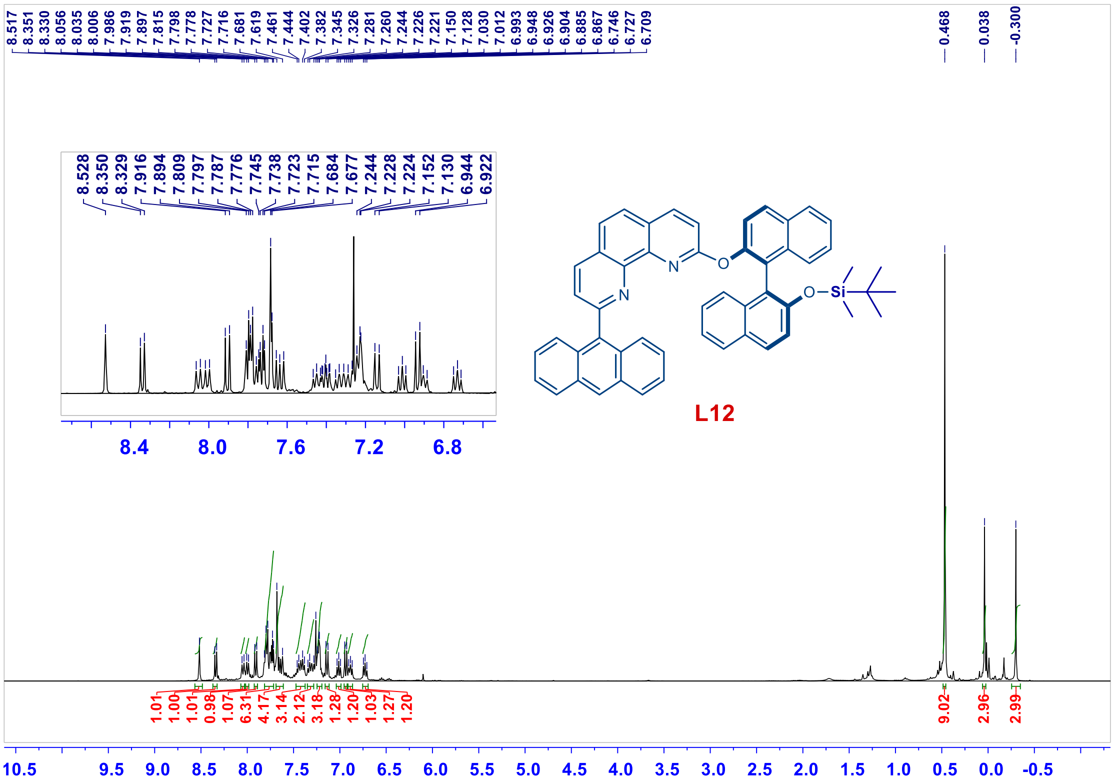

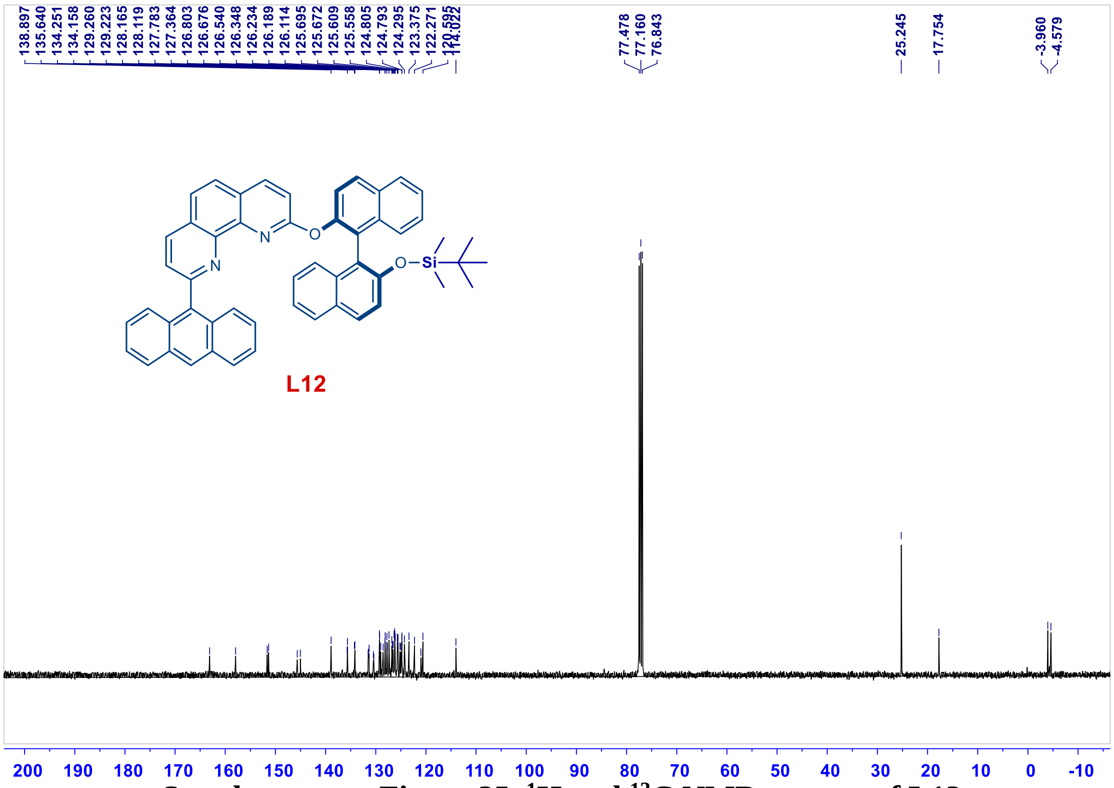

Supplementary Figure 25. ^{1}H and ^{13}C NMR spectra of L12

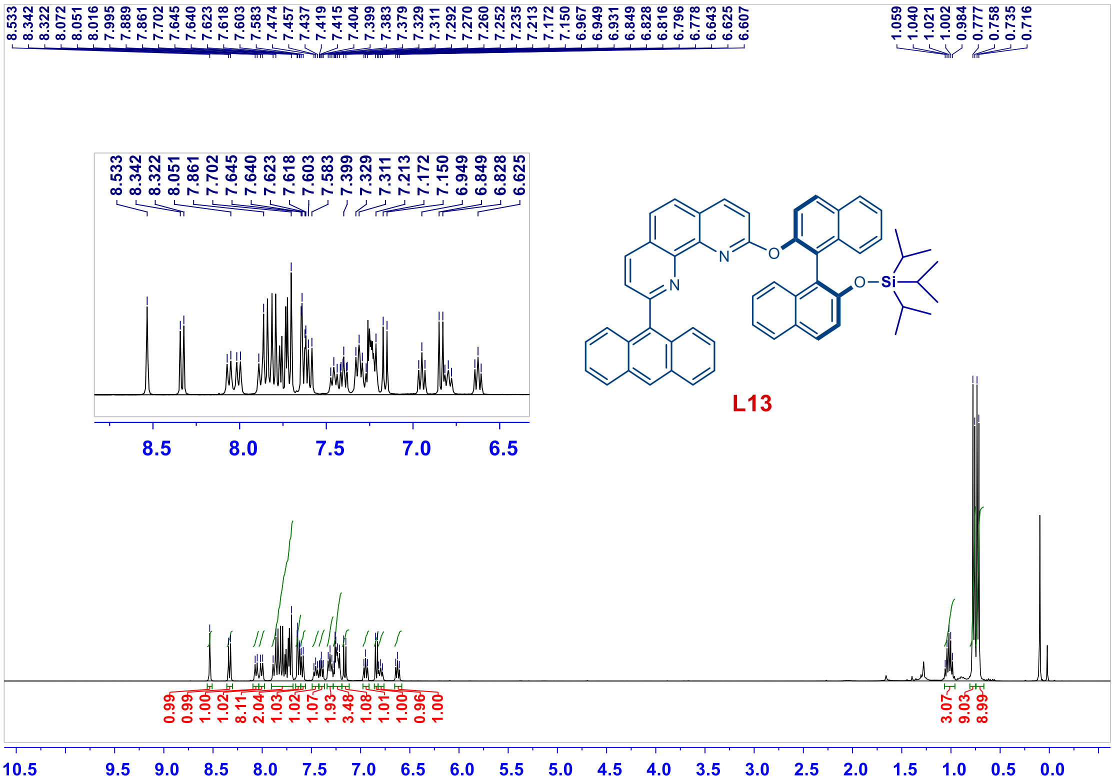

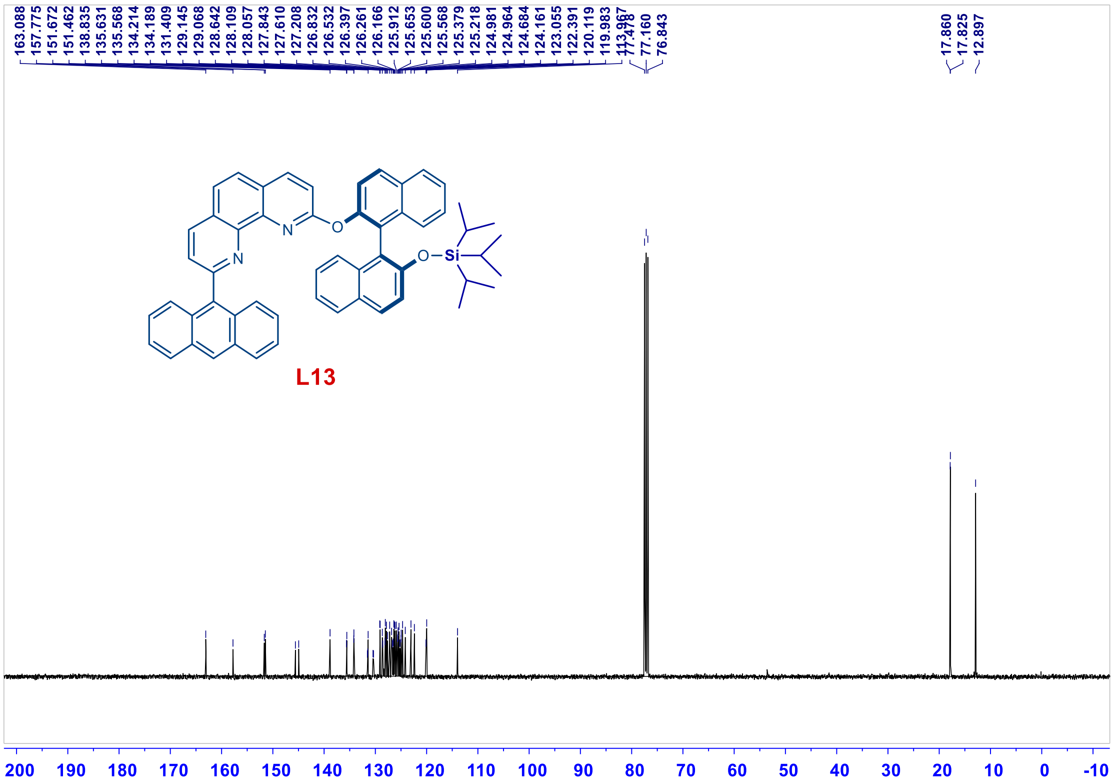

Supplementary Figure 26. ^{1}H and ^{13}C NMR spectra of L13

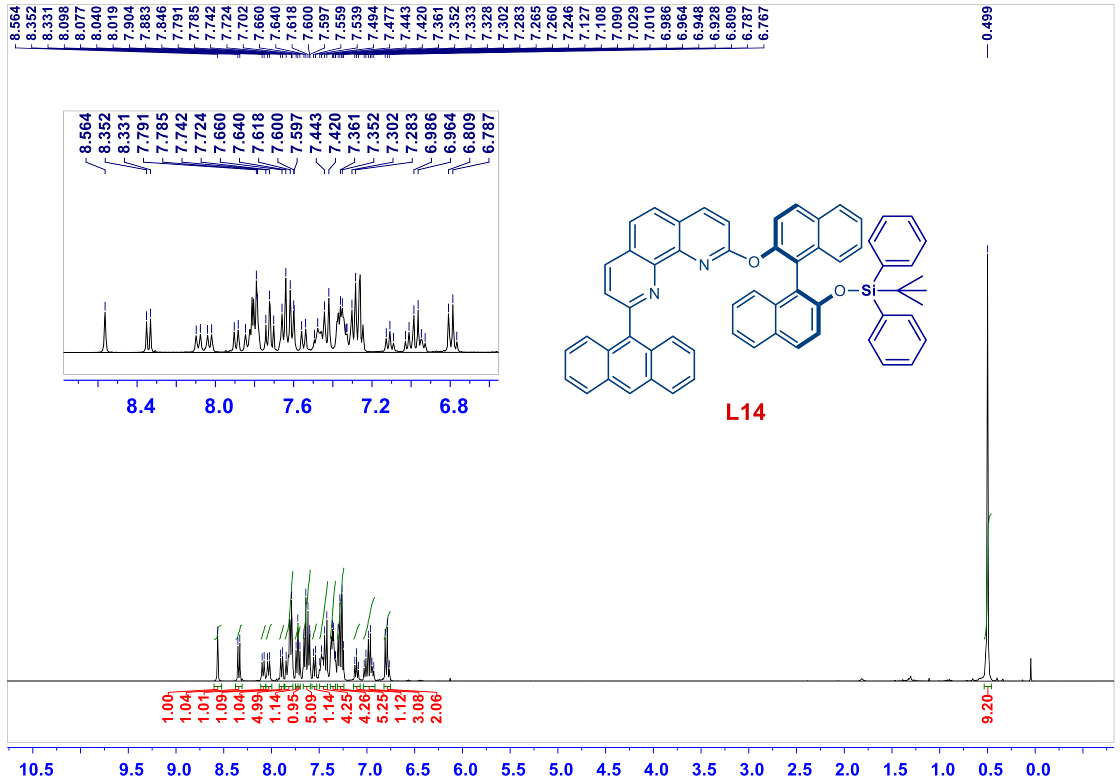

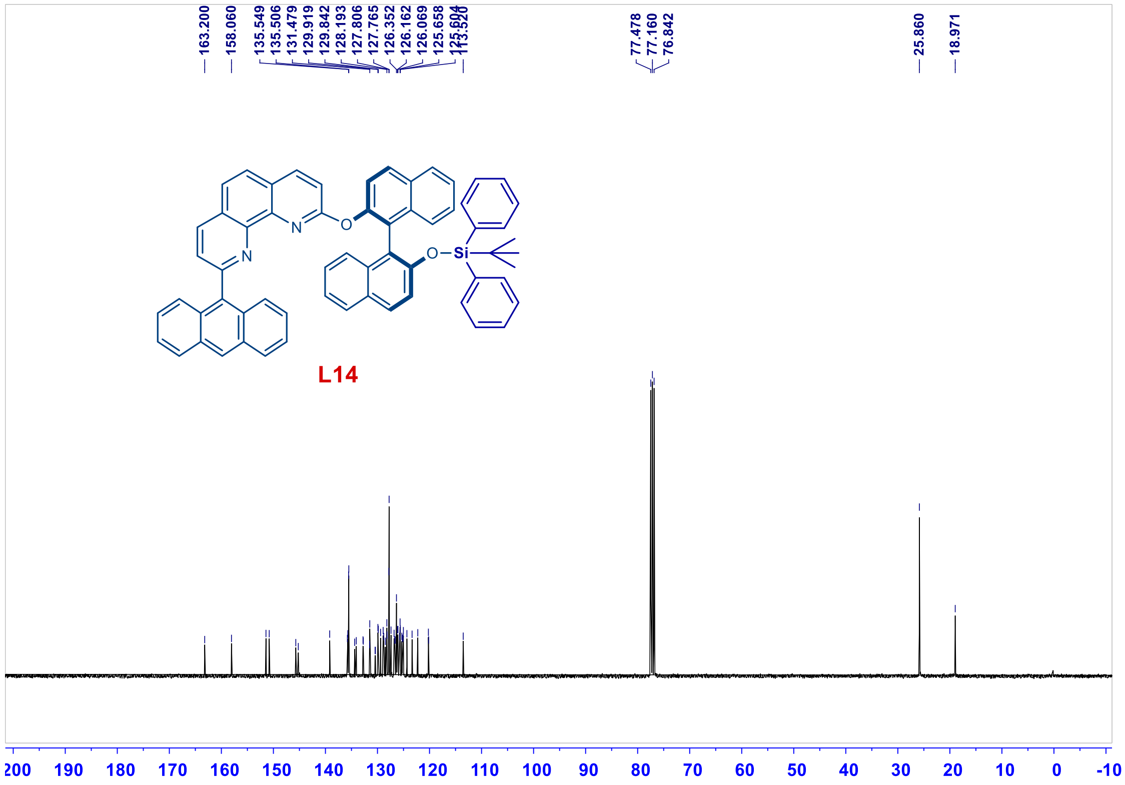

Supplementary Figure 27. ^{1}H and ^{13}C NMR spectra of L14

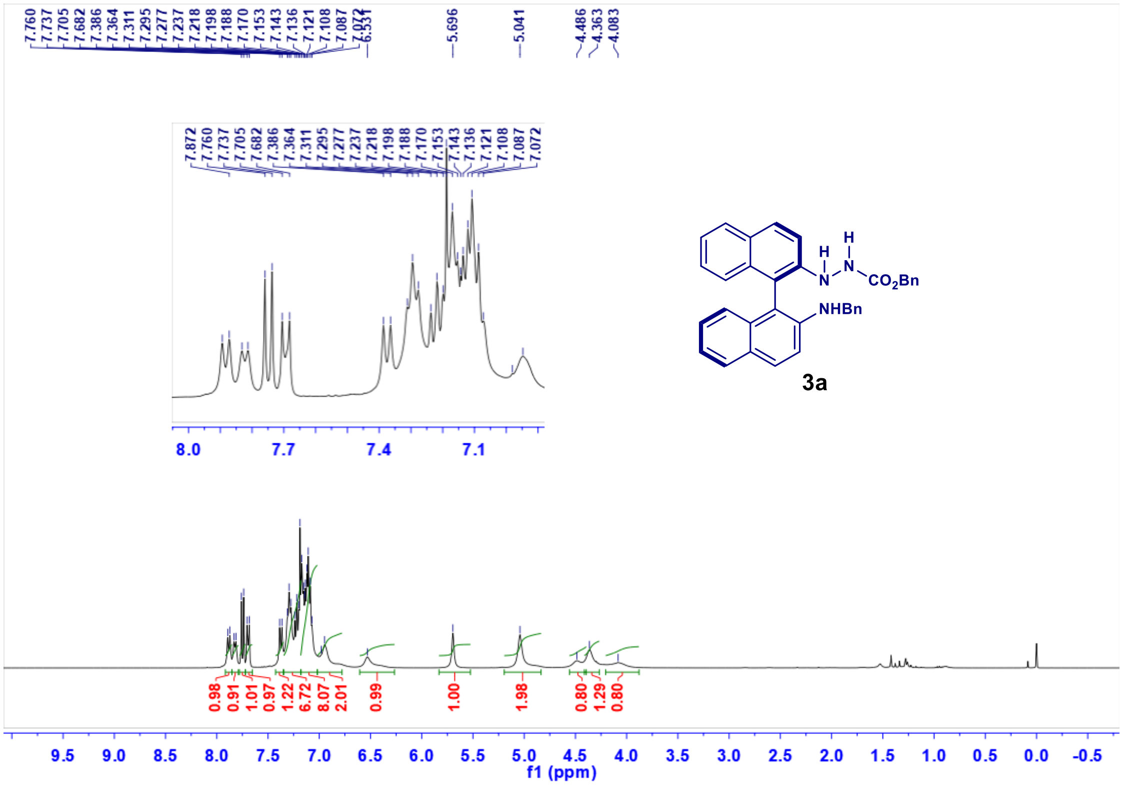

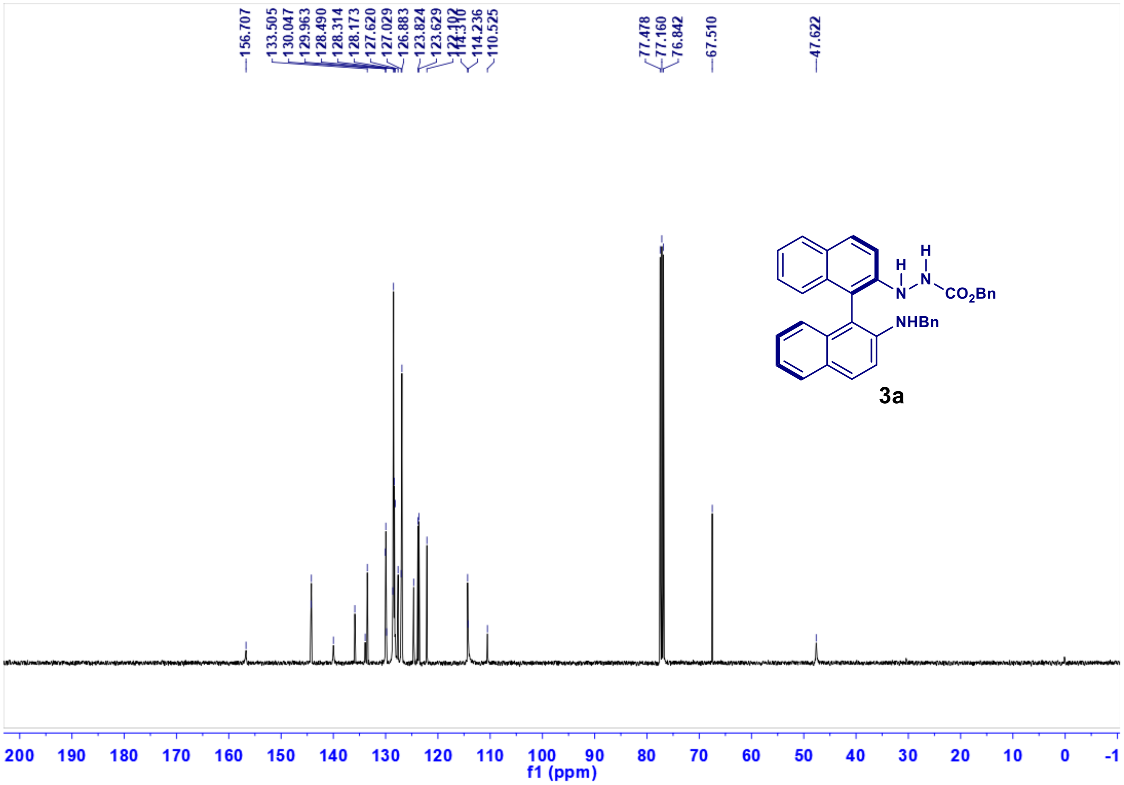

Supplementary Figure 28. ^{1}H and ^{13}C NMR spectra of 3a

Supplementary Figure 29. HPLC spectra of (*R*)-benzyl 2-(2'-(benzylamino)-[1,1'-binaphthalen]-2-yl)hydrazine-1-carboxylate (3a). Diacel Chiralpak AD-H, *n*-Hexane:*i*-PrOH = 85:15, flow = 1.0 mL/min, 25 °C, λ = 254 nm, t_R(major) = 13.9 min, t_R(minor) = 12.8 min, e.r. = 97.5:2.5

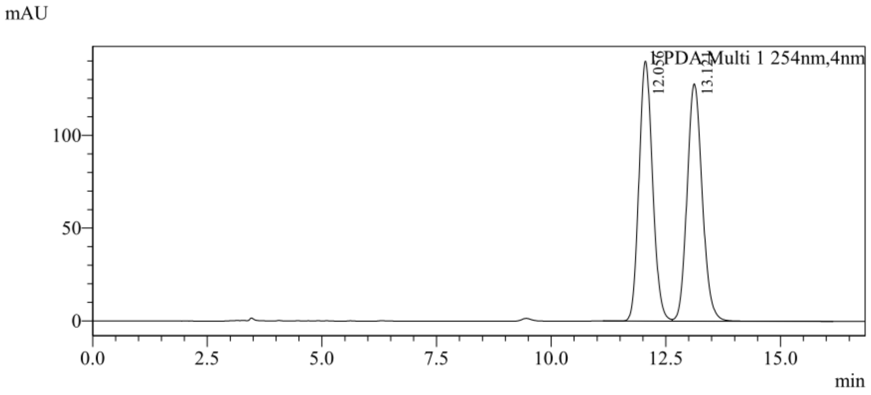

Peak Table

PDA Ch1 254nm

Peak#	Ret. Time	Peak End	Height	Area	Area%
1	12.056	12.640	140104	2890583	49.538
2	13.121	16.139	127884	2944533	50.462
Total			267988	5835116	100.000

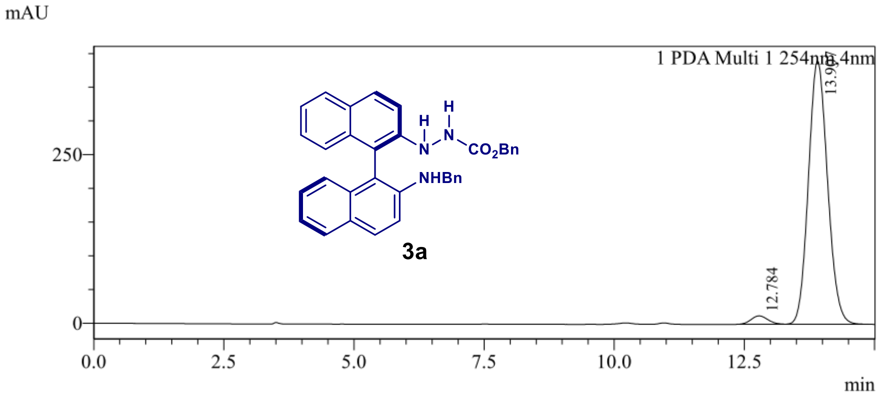

Peak Table

PDA Ch1 254nm

Peak#	Ret. Time	Peak End	Height	Area	Area%
1	12.784	13.259	12371	275530	2.688
2	13.907	14.869	390074	9975505	97.312
Total			402445	10251035	100.000

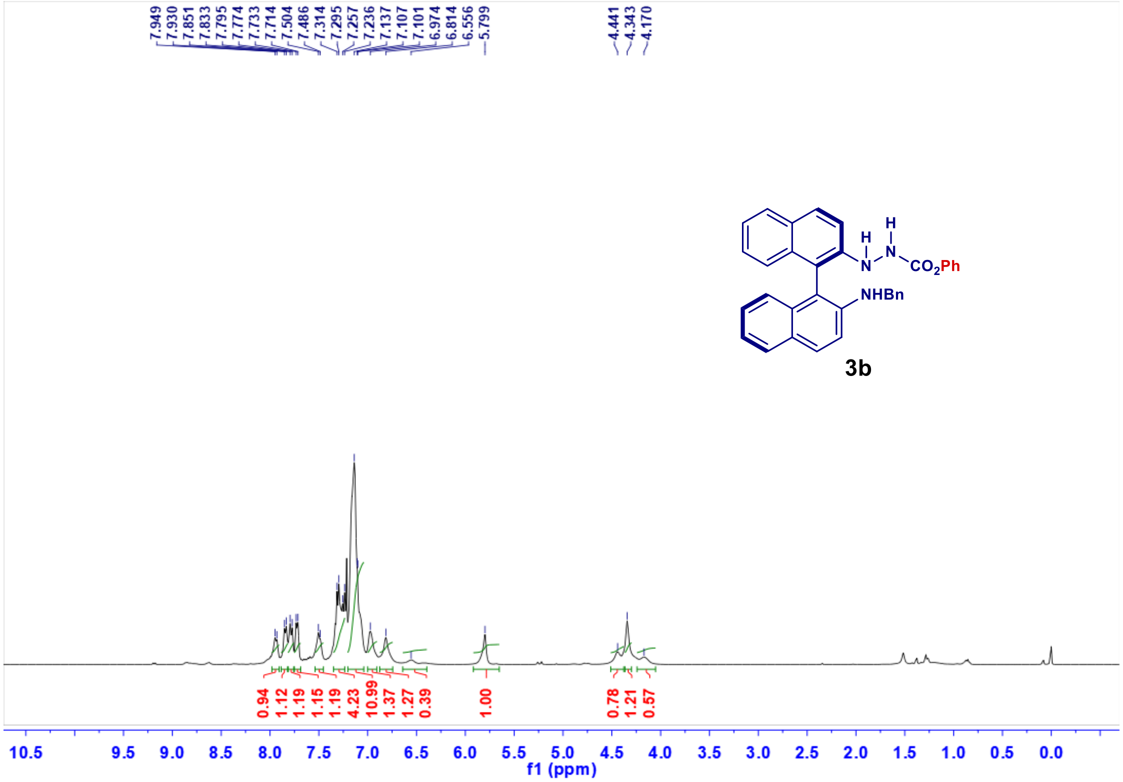

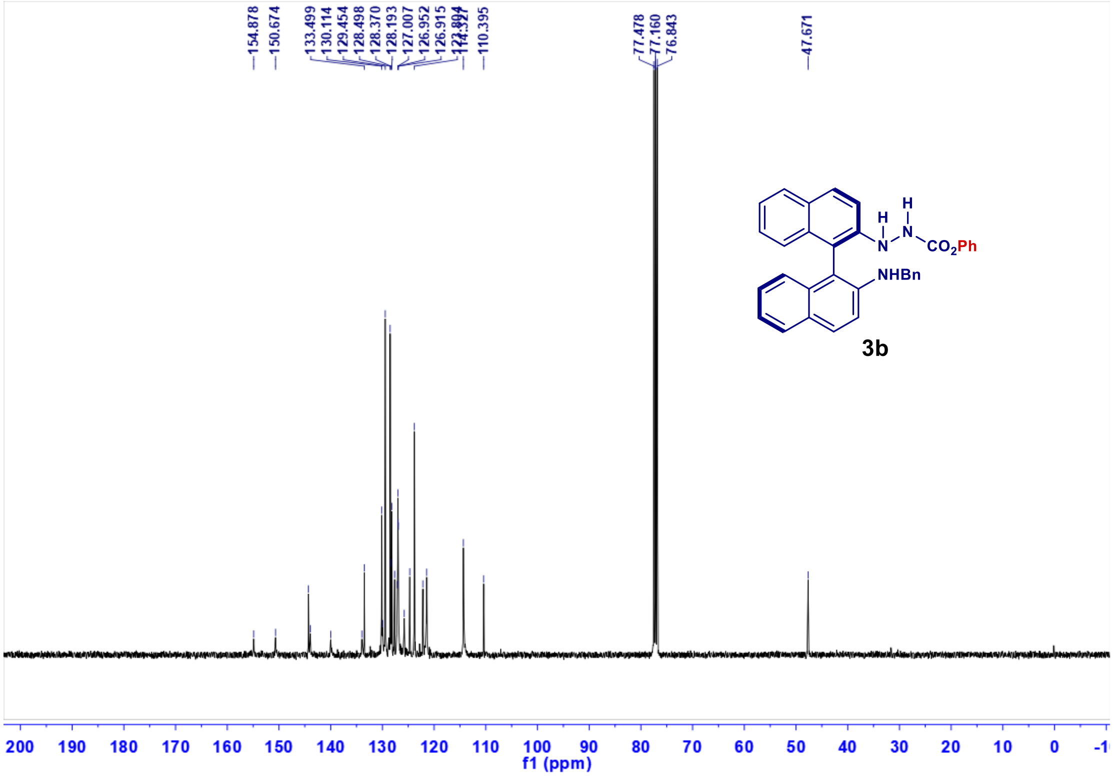

Supplementary Figure 30. ^{1}H and ^{13}C NMR spectra of 3b

Supplementary Figure 31. HPLC spectra of (*R*)-phenyl 2-(2'-(benzylamino)-[1,1'-binaphthalen]-2-yl)hydrazine-1-carboxylate (3b). Diacel Chiralcel OD-H, *n*-Hexane:*i*-PrOH = 80:20, flow = 1.0 mL/min, 25 °C, λ = 254 nm, t_R(major) = 11.8 min, t_R(minor) = 19.8 min, e.r. = 88.5:11.5

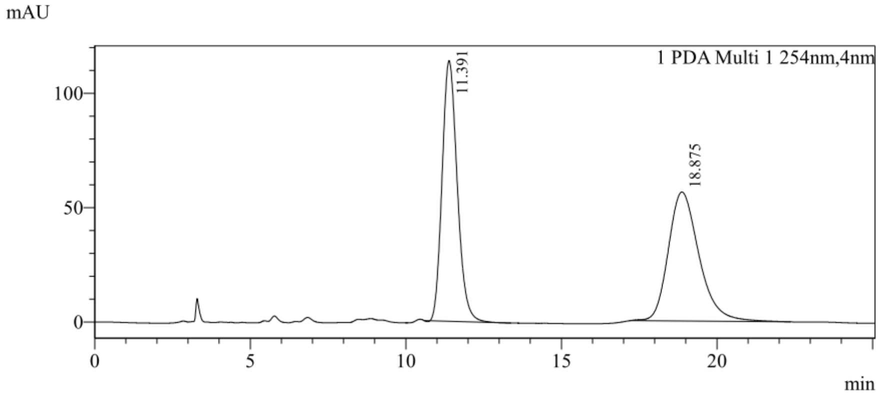

Peak Table

PDA Ch1 254nm

Peak#	Ret. Time	Peak End	Height	Area	Area%
1	11.391	13.365	113955	3850949	49.789
2	18.875	22.357	56417	3883532	50.211
Total			170372	7734481	100.000

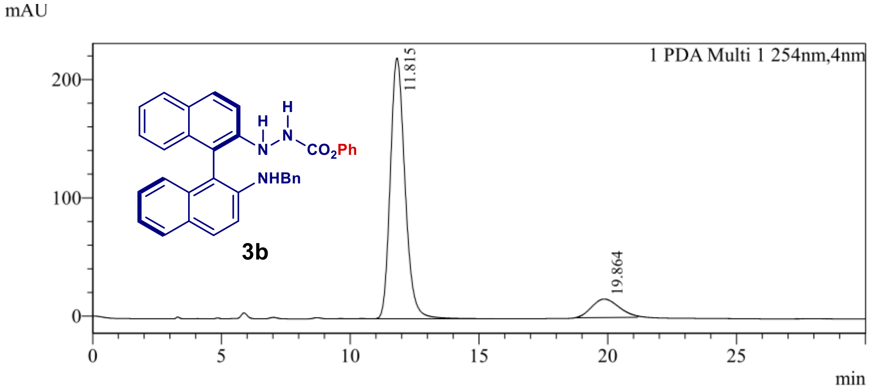

Peak Table

PDA Ch1 254nm

Peak#	Ret. Time	Peak End	Height	Area	Area%
1	11.815	15.552	220261	8726647	88.431
2	19.864	21.152	15587	1141660	11.569
Total			235848	9868308	100.000

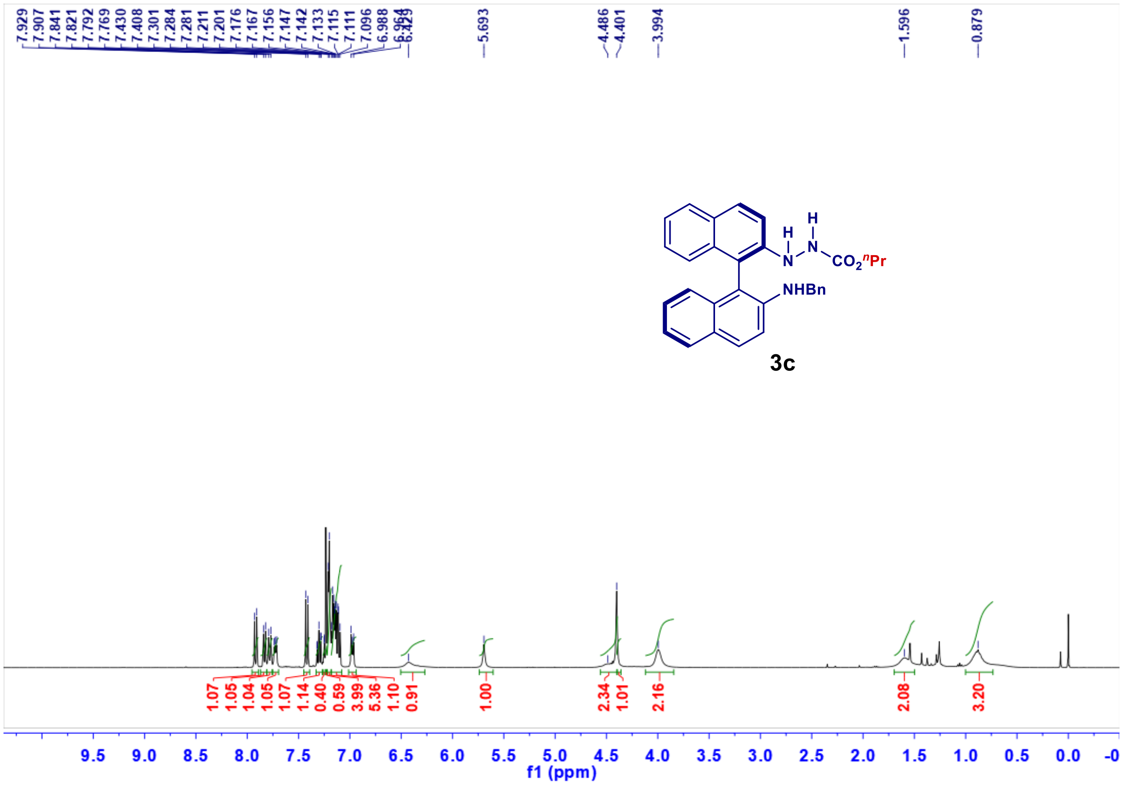

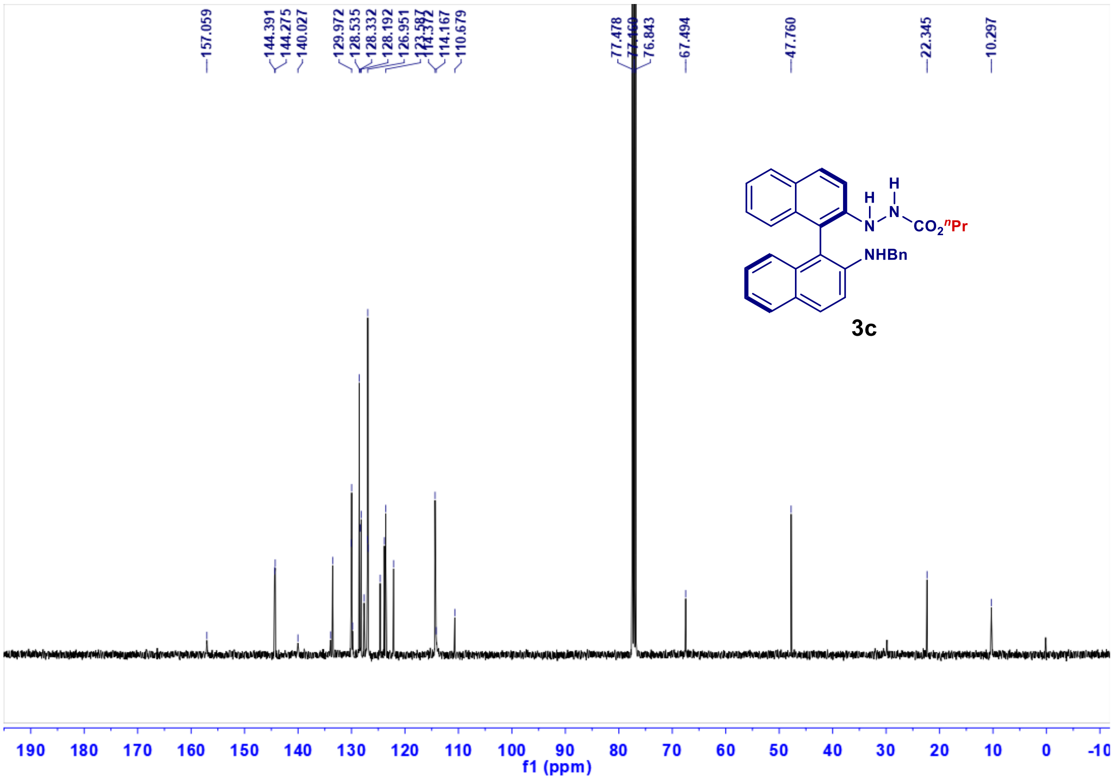

Supplementary Figure 32. ^{1}H and ^{13}C NMR spectra of 3c

Supplementary Figure 33. HPLC spectra of (*R*)-propyl 2-(2'-(benzylamino)-[1,1'-binaphthalen]-2-yl)hydrazine-1-carboxylate (3c). Diacel Chiralpak AD-H, *n*-Hexane:*i*-PrOH = 85:15, flow = 1.0 mL/min, 25 °C, λ = 254 nm, t_R(major) = 10.4 min, t_R(minor) = 7.8 min, e.r. = 97:3

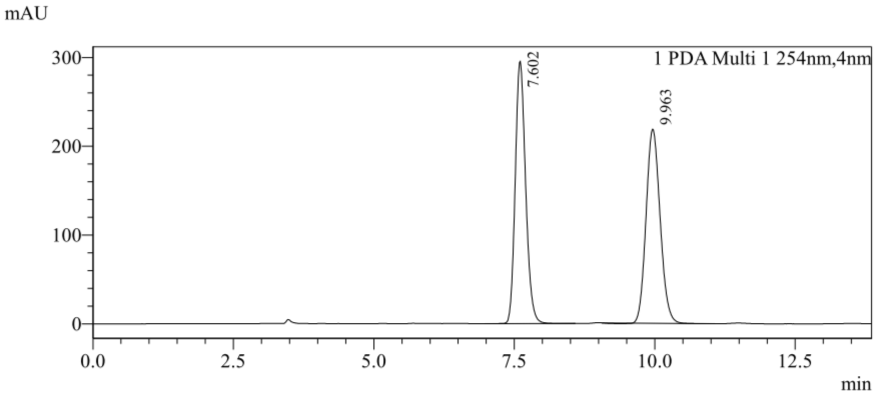

Peak Table

PDA Ch1 254nm

Peak#	Ret. Time	Peak End	Height	Area	Area%
1	7.602	8.576	295145	3788094	49.976
2	9.963	11.061	218790	3791726	50.024
Total			513934	7579820	100.000

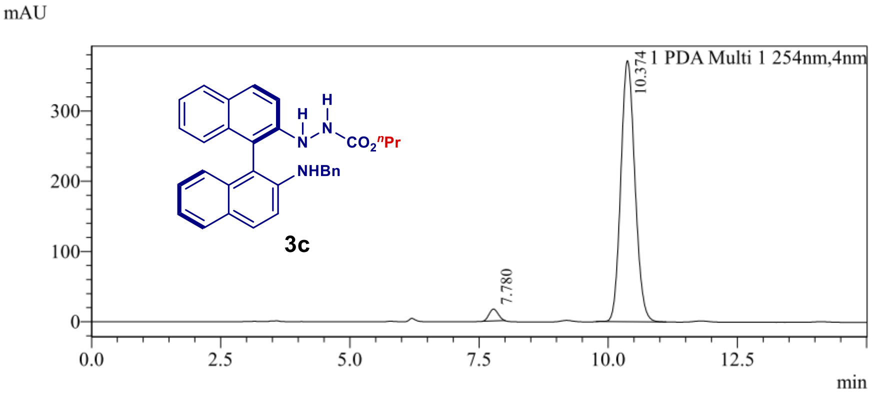

Peak Table

PDA Ch1 254nm

Peak#	Ret. Time	Peak End	Height	Area	Area%
1	7.780	8.000	16877	207303	2.848
2	10.374	11.115	371476	7072797	97.152
Total			388353	7280100	100.000

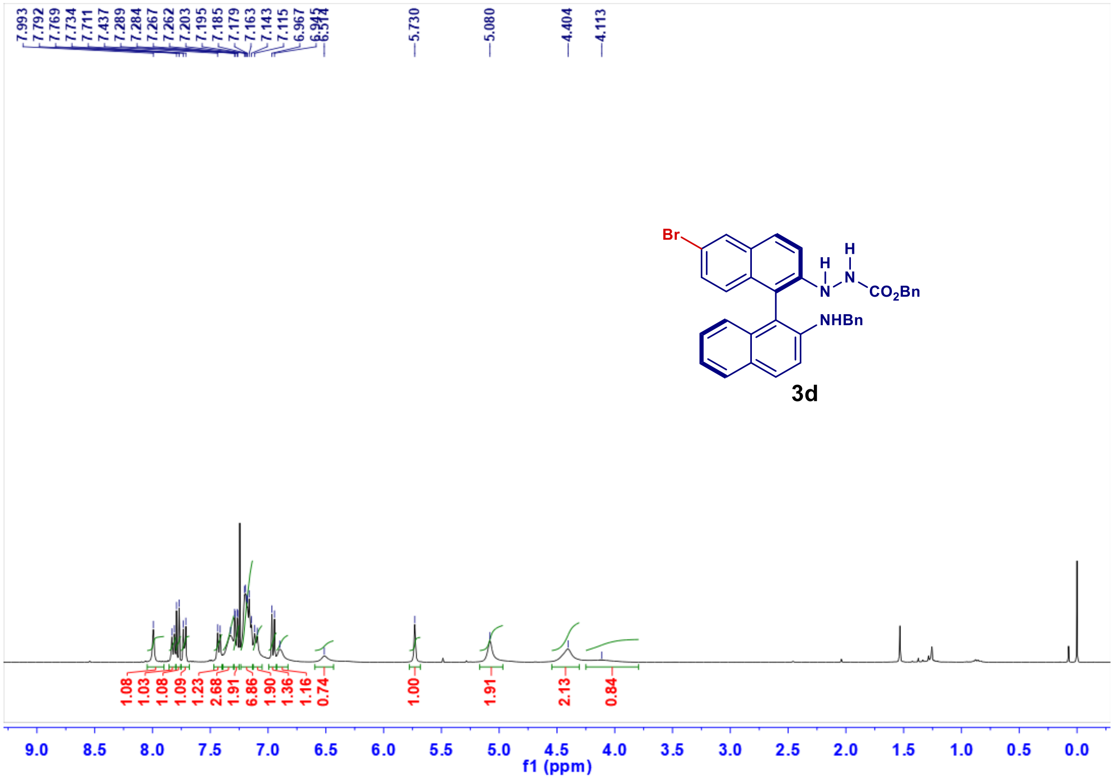

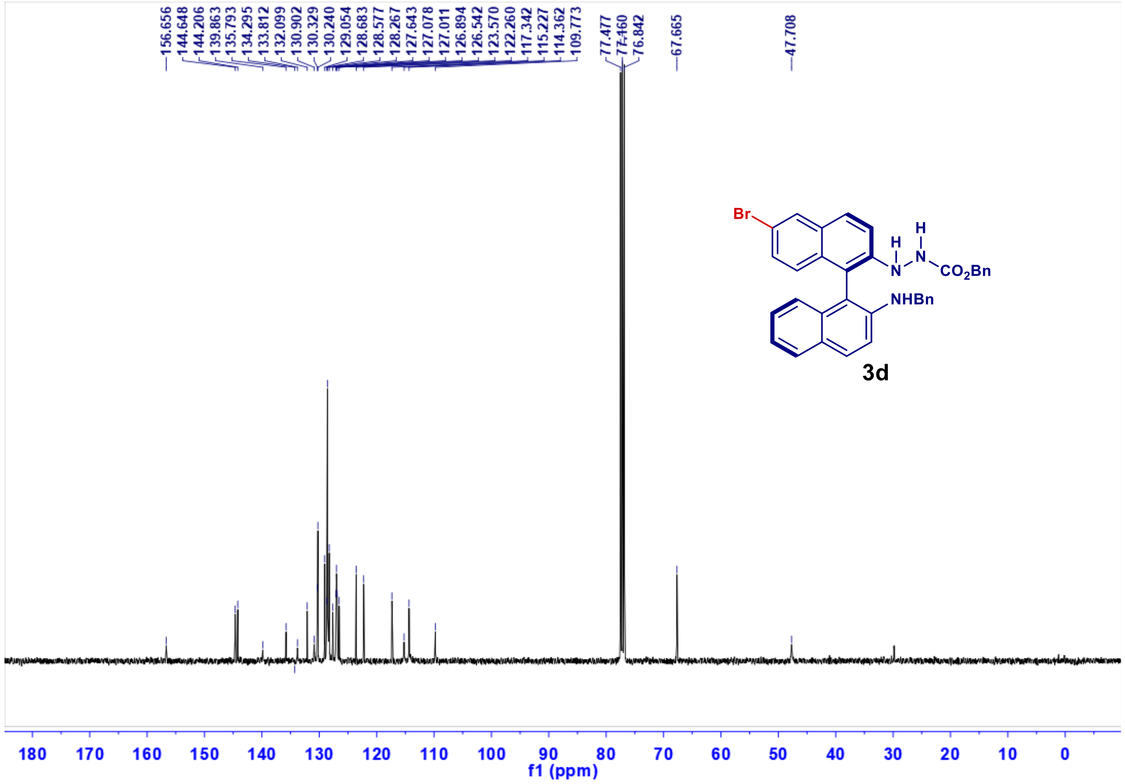

Supplementary Figure 34. ^{1}H and ^{13}C NMR spectra of 3d

Supplementary Figure 35. HPLC spectra of (*R*)-Benzyl 2-(2'-(benzylamino)-6-bromo-[1,1'-binaphthalen]-2-yl)hydrazine-1-carboxylate (3d). Diacel Chiralpak AD-H, *n*-Hexane:*i*-PrOH = 85:15, flow = 1.0 mL/min, 25 °C, λ = 254 nm, t_R(major) = 12.4 min, t_R(minor) = 10.3 min, e.r. = 96.5:3.5

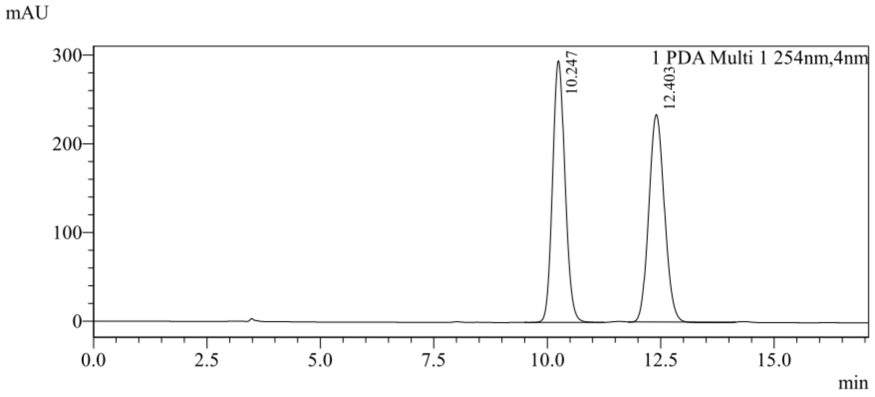

Peak Table

PDA Ch1 254nm

Peak#	Ret. Time	Peak End	Height	Area	Area%
1	10.247	11.243	294775	5650751	50.375
2	12.403	14.144	233887	5566678	49.625
Total			528662	11217429	100.000

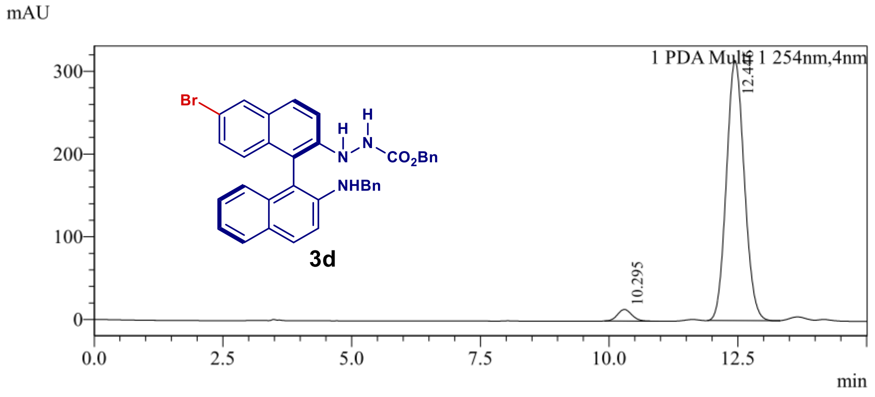

Peak Table

PDA Ch1 254nm

Peak#	Ret. Time	Peak End	Height	Area	Area%
1	10.295	10.955	14004	269951	3.431
2	12.446	13.312	314220	7599050	96.569
Total			328225	7869001	100.000

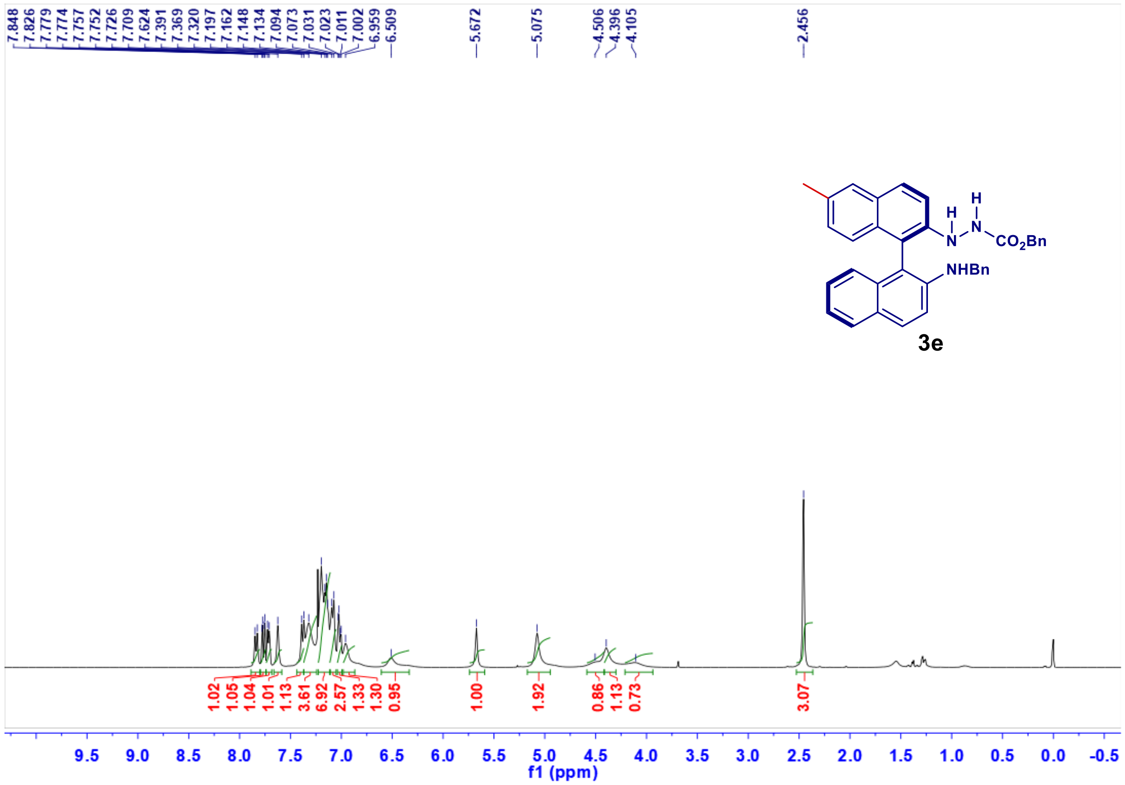

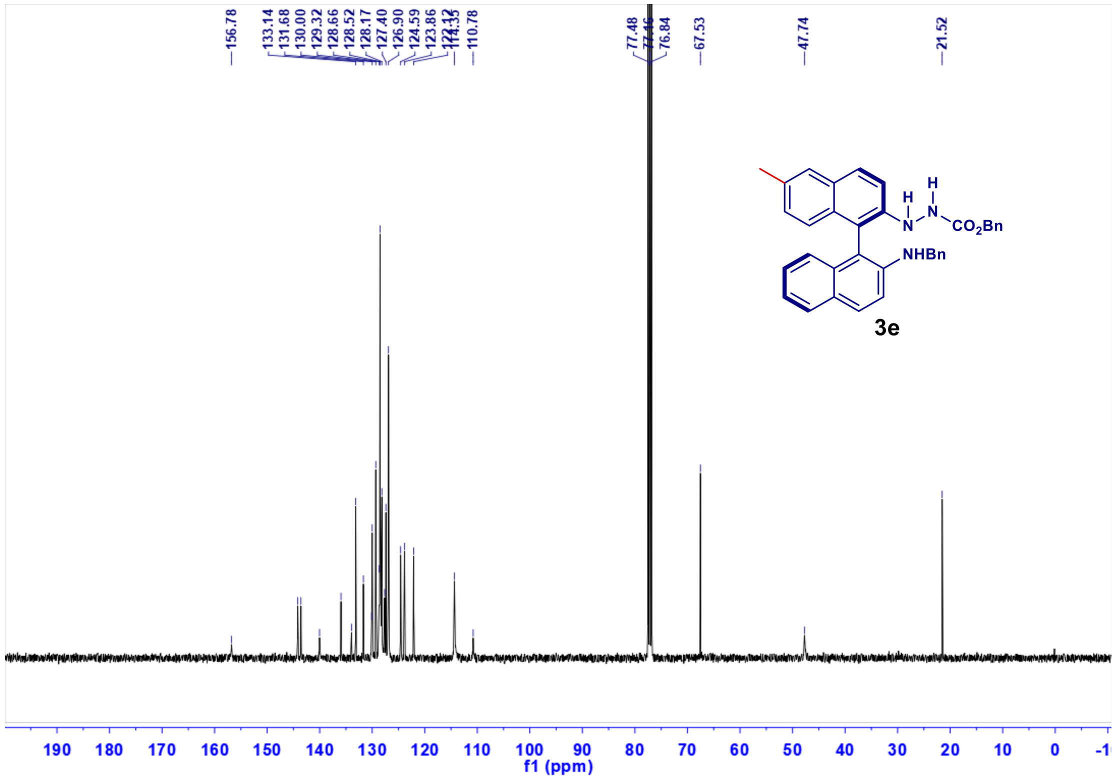

Supplementary Figure 36. ^{1}H and ^{13}C NMR spectra of 3e

Supplementary Figure 37. HPLC spectra of (*R*)-benzyl 2-(2'-(benzylamino)-6-methyl-[1,1'-binaphthalen]-2-yl)hydrazine-1-carboxylate (3e). Diacel Chiralpak AD-H, *n*-Hexane:*i*-PrOH = 85:15, flow = 1.0 mL/min, 25 °C, λ = 254 nm, t_R(major) = 11.2 min, t_R(minor) = 9.6 min, e.r. = 97:3

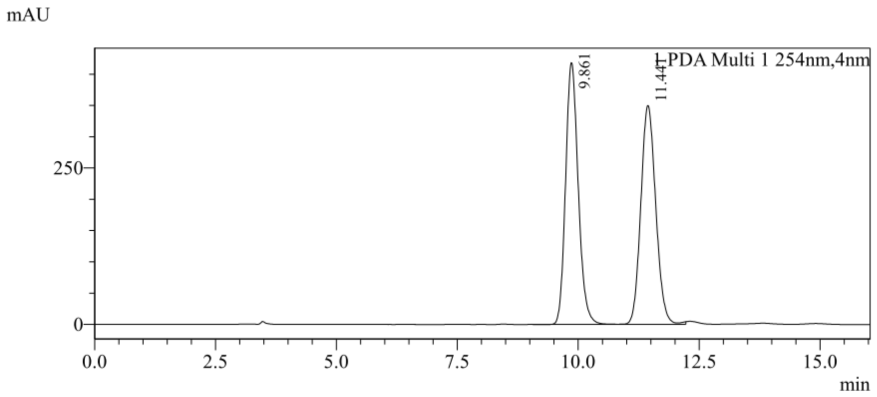

Peak Table

PDA Ch1 254nm

Peak#	Ret. Time	Peak End	Height	Area	Area%
1	9.861	10.795	418216	7607820	50.251
2	11.441	12.224	349724	7531852	49.749
Total			767940	15139672	100.000

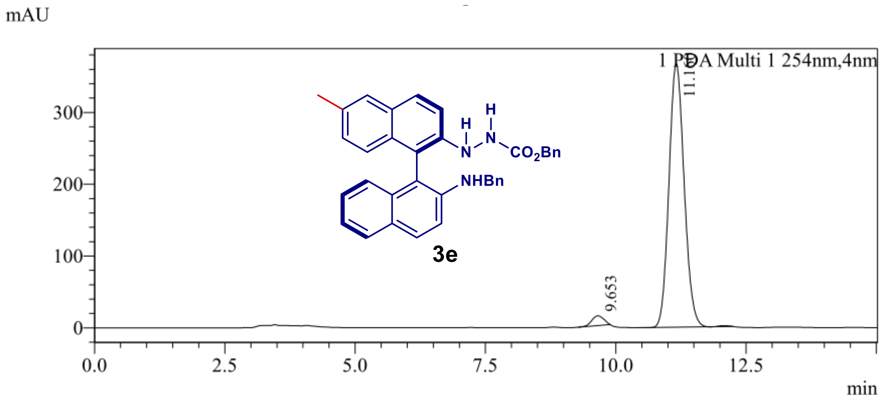

Peak Table

PDA Ch1 254nm

Peak#	Ret. Time	Peak End	Height	Area	Area%
1	9.653	9.888	13568	215569	2.811
2	11.160	12.235	367242	7453096	97.189
Total			380810	7668665	100.000

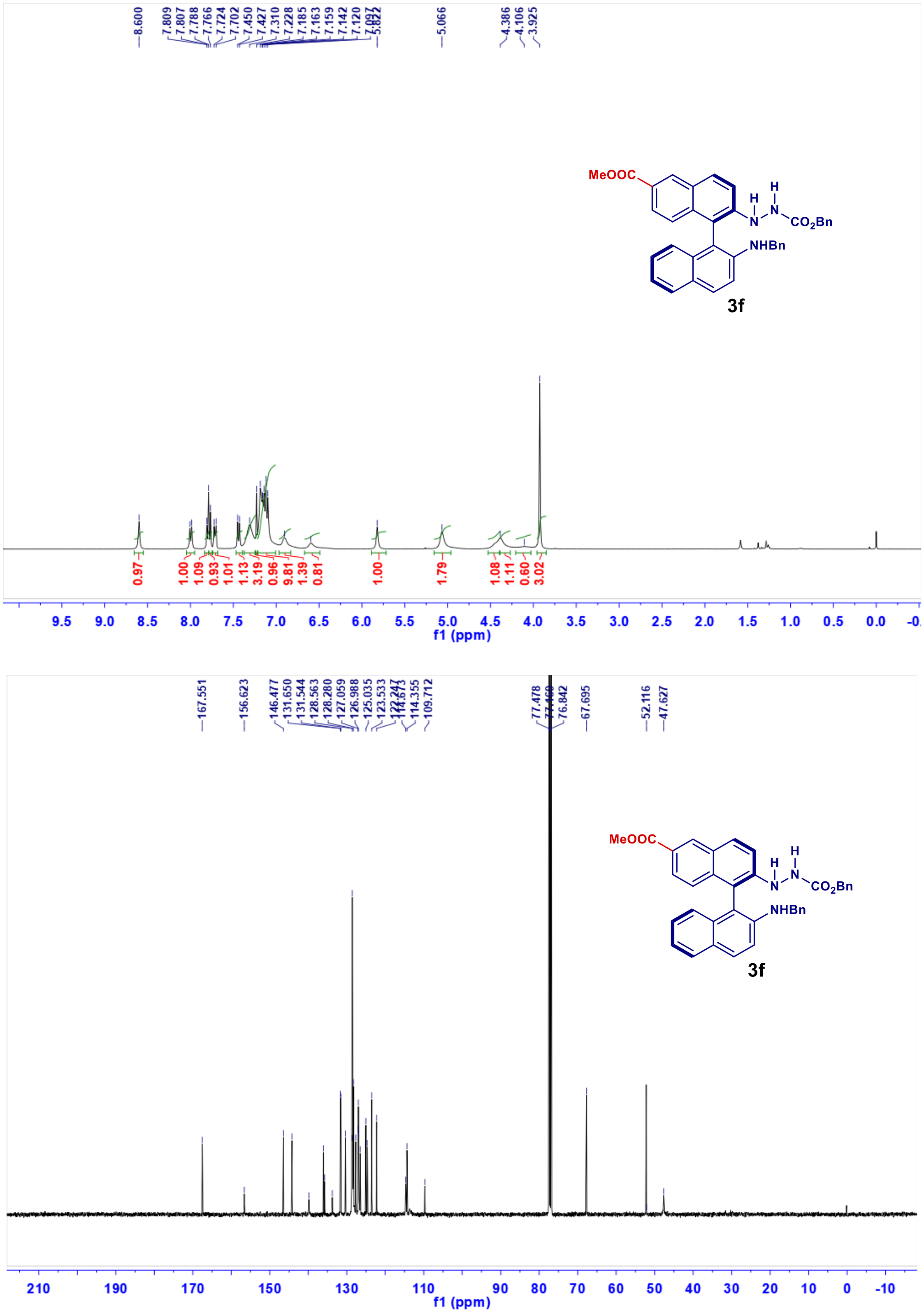

Supplementary Figure 38. ^{1}H **and** ^{13}C **NMR spectra of 3f**

Supplementary Figure 39. HPLC spectra of (*R*)-benzyl 2-(2'-(benzylamino)-6-(methoxycarbonyl)-[1,1'-binaphthalen]-2-yl)hydrazine-1-carboxylate (3f). Diacel Chiralcel OD-H, *n*-Hexane:*i*-PrOH = 80:20, flow = 1.0 mL/min, 25 °C, λ = 254 nm, t_R(major) = 18.9 min, t_R(minor) = 24.5 min, e.r. = 97.5:2.5

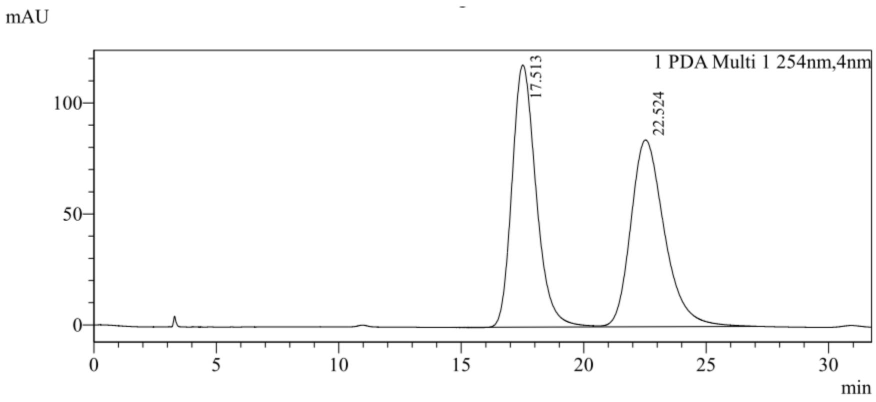

Peak Table

PDA Ch1 254nm

Peak#	Ret. Time	Peak End	Height	Area	Area%
1	17.513	20.384	118116	8082027	50.083
2	22.524	27.093	84169	8055373	49.917
Total			202285	16137399	100.000

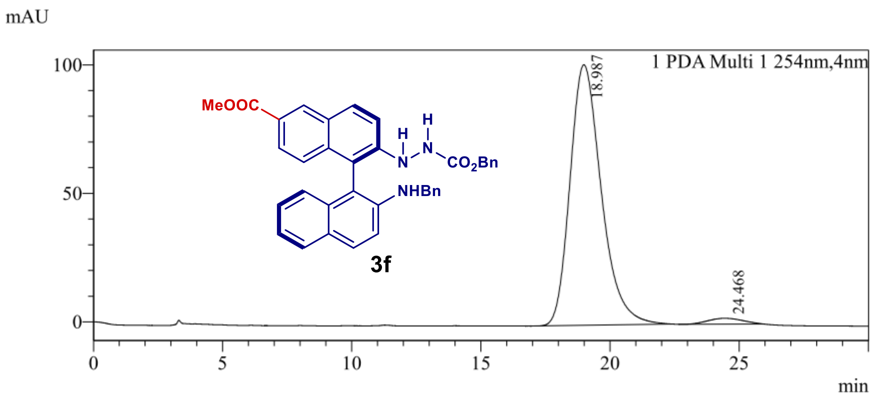

Peak Table

PDA Ch1 254nm

Peak#	Ret. Time	Peak End	Height	Area	Area%
1	18.987	22.432	101356	8557590	97.503
2	24.468	25.995	2306	219164	2.497
Total			103662	8776754	100.000

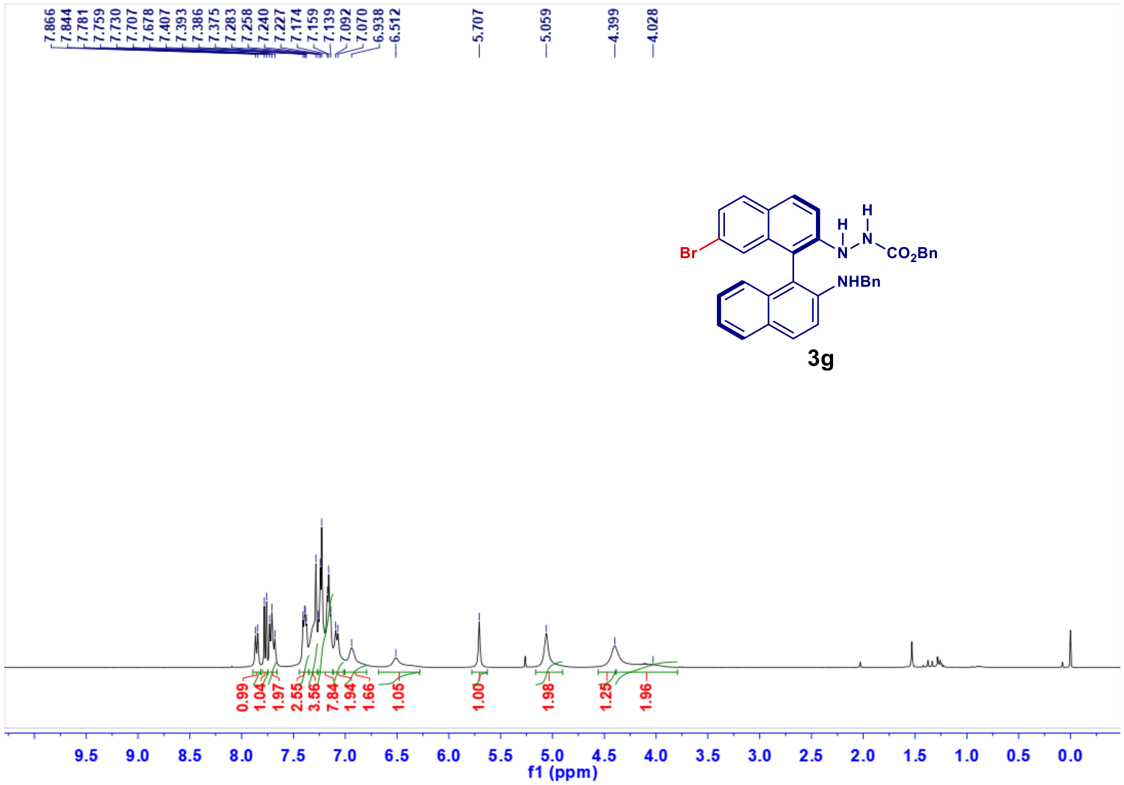

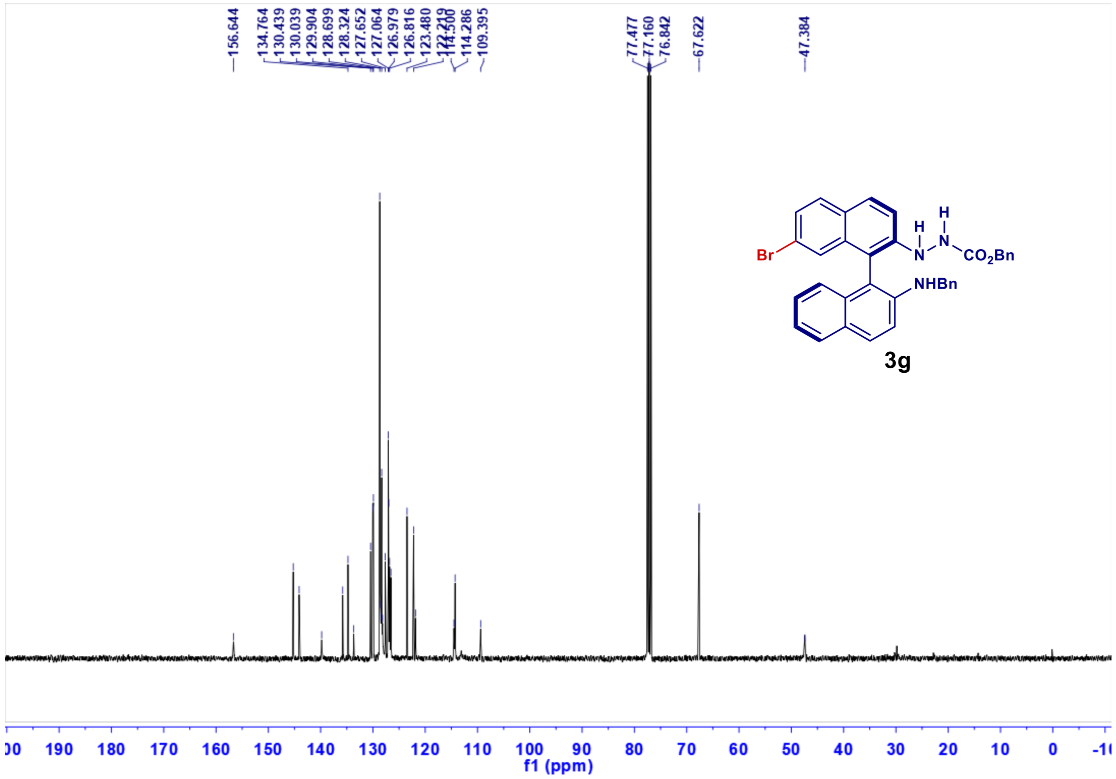

Supplementary Figure 40. ^{1}H and ^{13}C NMR spectra of 3g

Supplementary Figure 41. HPLC spectra of (*R*)-benzyl 2-(2'-(benzylamino)-7-bromo-[1,1'-binaphthalen]-2-yl)hydrazine-1-carboxylate (3g). Diacel Chiralcel OD-H, *n*-Hexane:*i*-PrOH = 80:20, flow = 1.0 mL/min, 25 °C, λ = 254 nm, t_R(major) = 12.4 min, t_R(minor) = 19.3 min, e.r. = 97:3

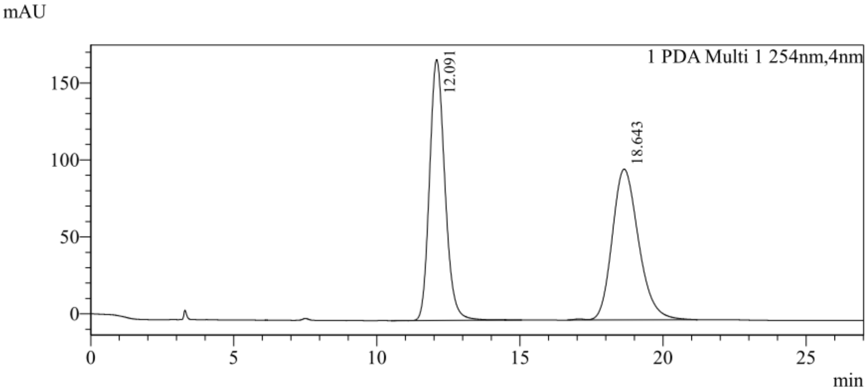

Peak Table

PDA Ch1 254nm

Peak#	Ret. Time	Peak End	Height	Area	Area%
1	12.091	15.051	169333	6321437	50.204
2	18.643	21.195	97923	6269947	49.796
Total			267256	12591384	100.000

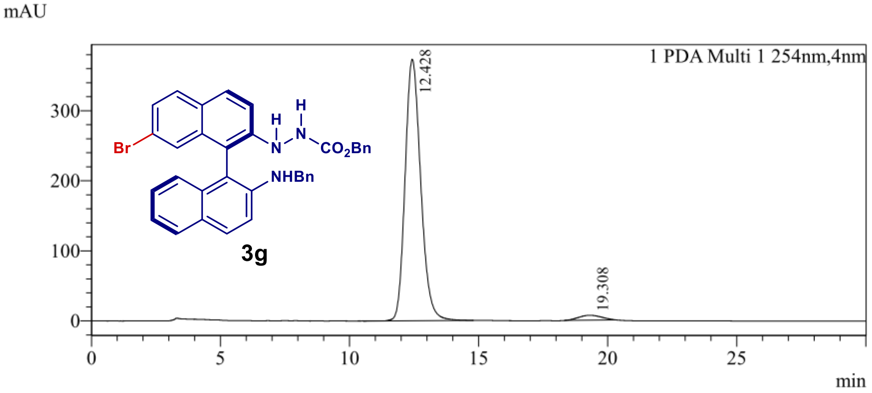

Peak Table

PDA Ch1 254nm

Peak#	Ret. Time	Peak End	Height	Area	Area%
1	12.428	14.784	372994	15714081	97.284
2	19.308	20.352	7045	438669	2.716
Total			380039	16152750	100.000

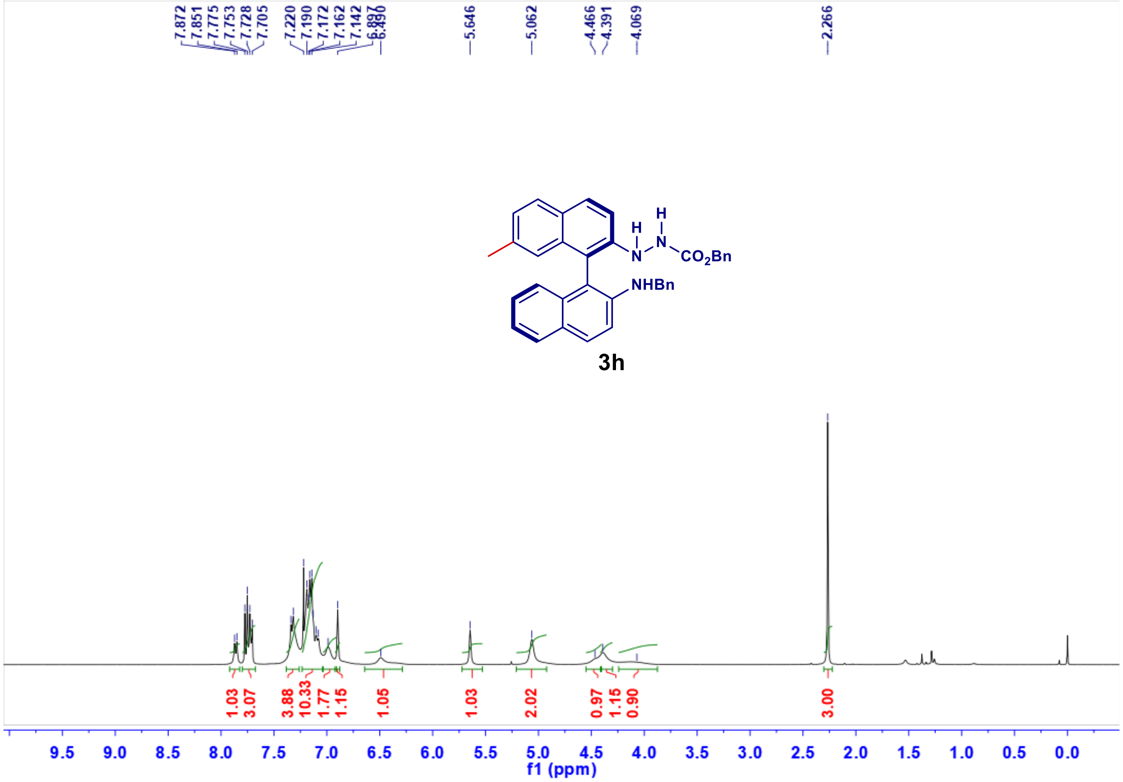

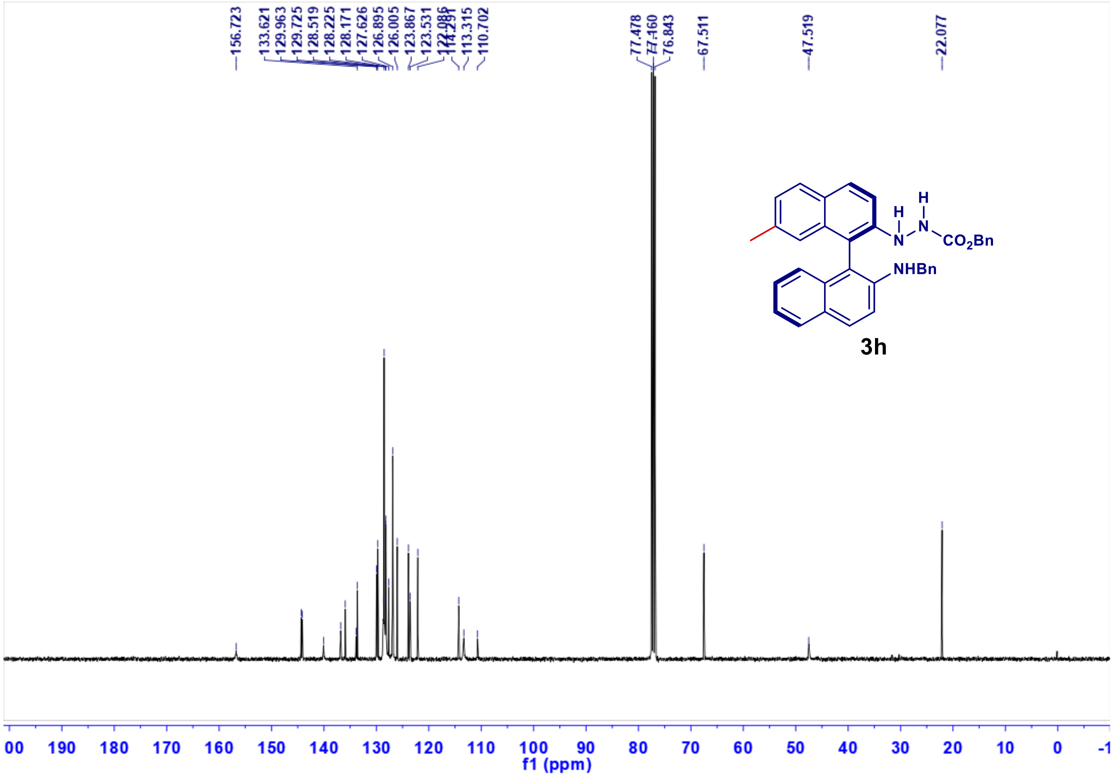

Supplementary Figure 42. ^{1}H and ^{13}C NMR spectra of 3h

Supplementary Figure 43. HPLC spectra of (*R*)-benzyl 2-(2'-(benzylamino)-7-methyl-[1,1'-binaphthalen]-2-yl)hydrazine-1-carboxylate (3h). Diacel Chiralpak AD-H, *n*-Hexane:*i*-PrOH = 85:15, flow = 1.0 mL/min, 25 °C, λ = 254 nm, t_R(major) = 11.8 min, t_R(minor) = 10. min, e.r. = 97:3

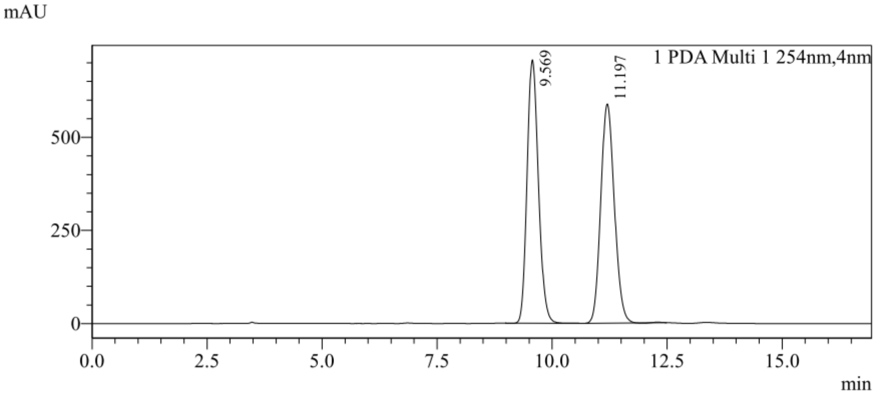

Peak Table

PDA Ch1 254nm

Peak#	Ret. Time	Peak End	Height	Area	Area%
1	9.569	10.677	706354	12168674	50.044
2	11.197	12.480	588564	12147156	49.956
Total			1294918	24315830	100.000

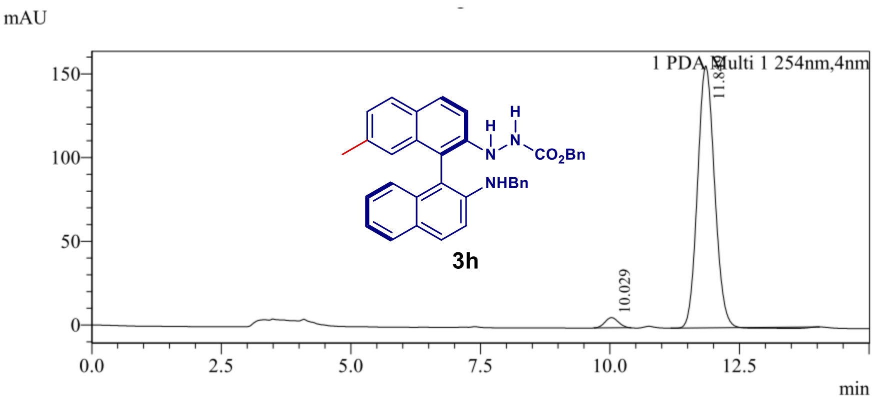

Peak Table

PDA Ch1 254nm

Peak#	Ret. Time	Peak End	Height	Area	Area%
1	10.029	10.400	6119	110483	3.030
2	11.849	14.037	156384	3536212	96.970
Total			162503	3646695	100.000

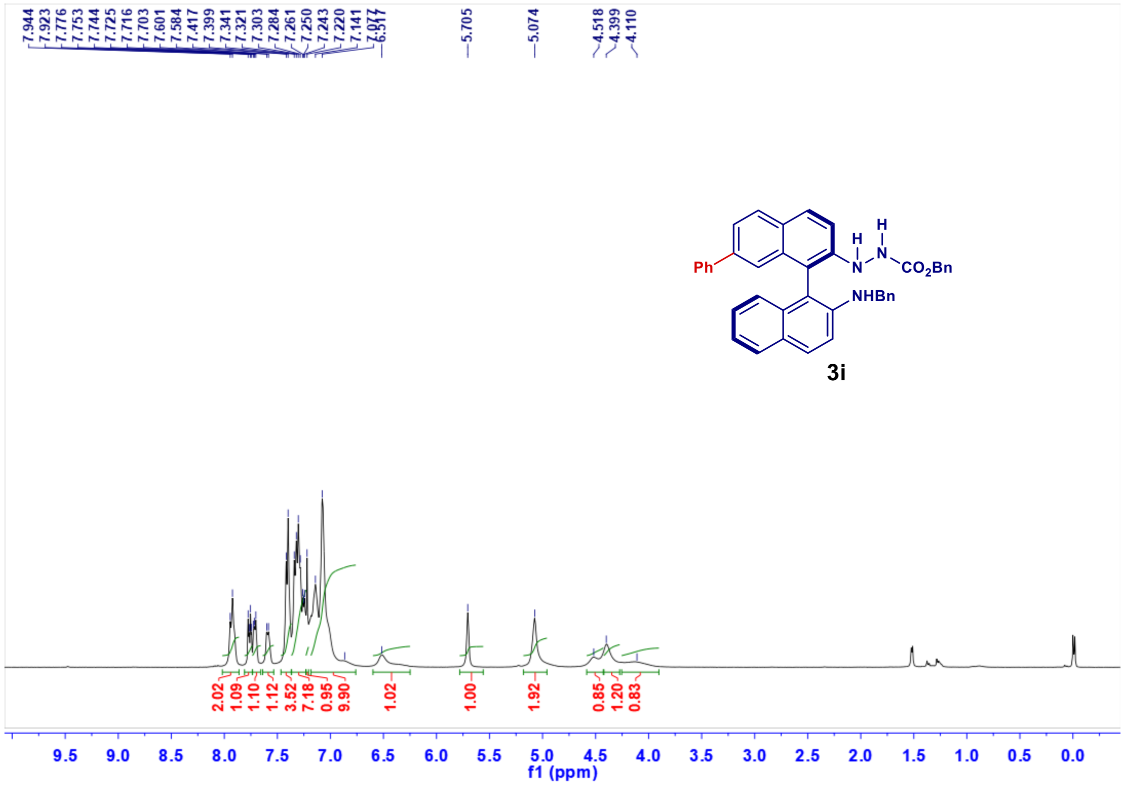

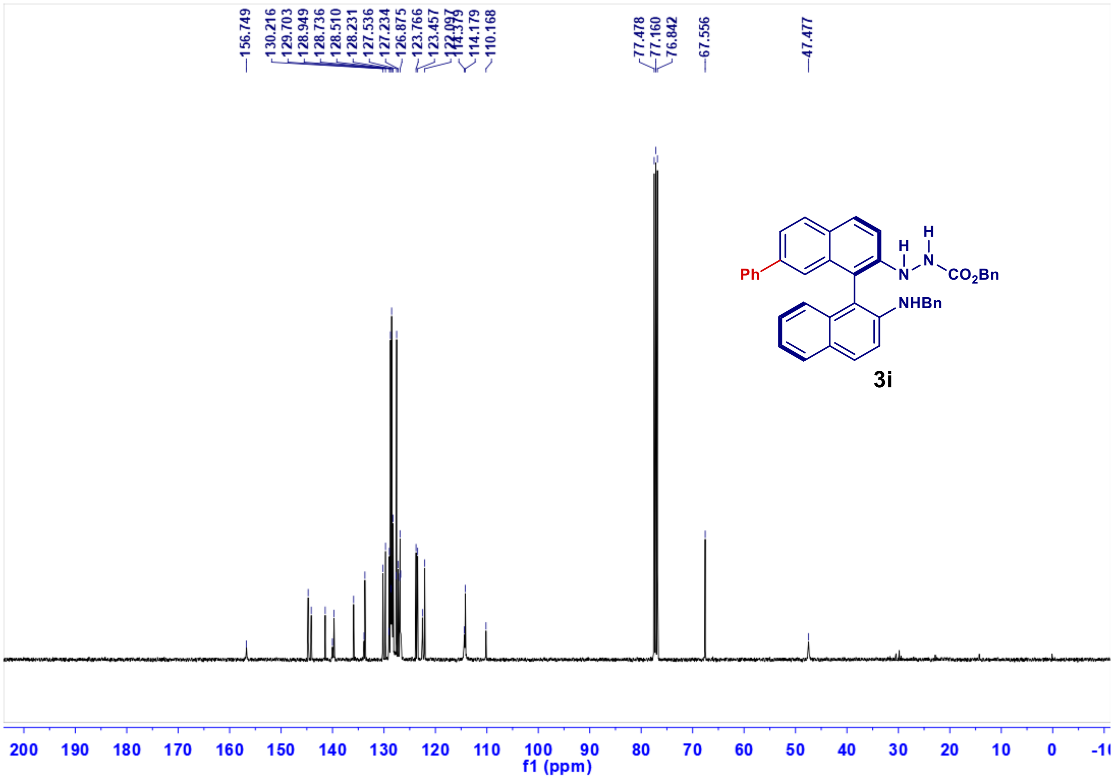

Supplementary Figure 44. ^{1}H and ^{13}C NMR spectra of 3i

Supplementary Figure 45. HPLC spectra of (*R*)-benzyl 2-(2'-(benzylamino)-7-phenyl-[1,1'-binaphthalen]-2-yl)hydrazine-1-carboxylate (3i). Diacel Chiralpak AD-H, *n*-Hexane:*i*-PrOH = 85:15, flow = 1.0 mL/min, 25 °C, λ = 254 nm, t_R(major) = 11.3 min, t_R(minor) = 18.6 min, e.r. = 97:3

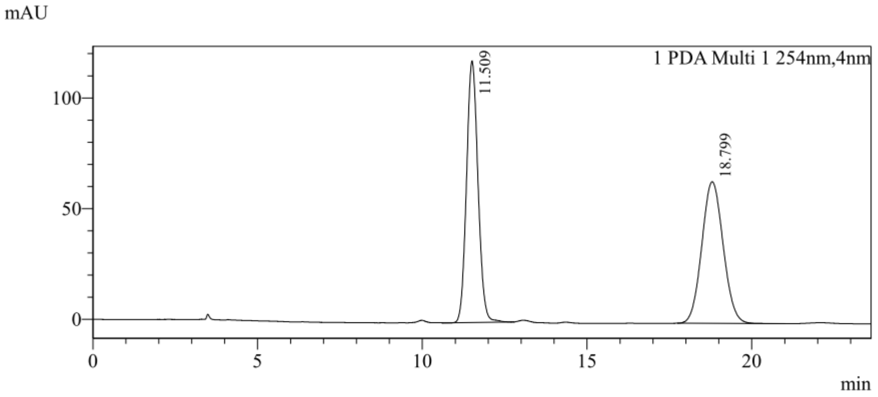

Peak Table

PDA Ch1 254nm

Peak#	Ret. Time	Peak End	Height	Area	Area%
1	11.509	12.789	118042	2857570	49.753
2	18.799	20.992	63985	2885977	50.247
Total			182027	5743547	100.000

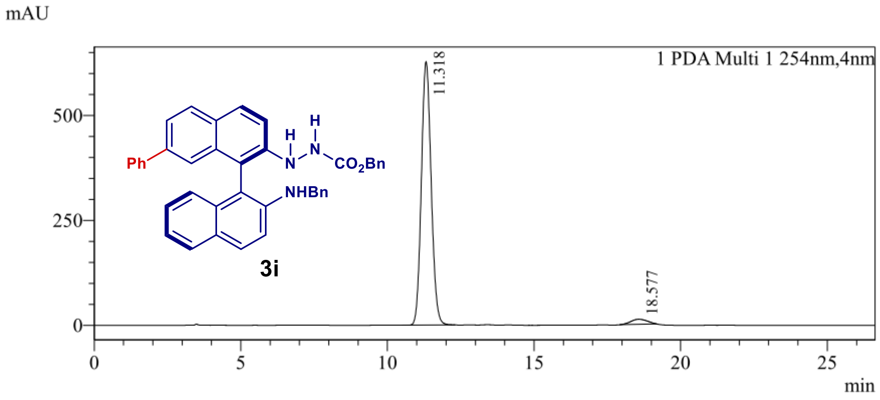

Peak Table

PDA Ch1 254nm

Peak#	Ret. Time	Peak End	Height	Area	Area%
1	11.318	12.299	627749	14598421	96.880
2	18.577	19.168	12082	470206	3.120
Total			639831	15068628	100.000

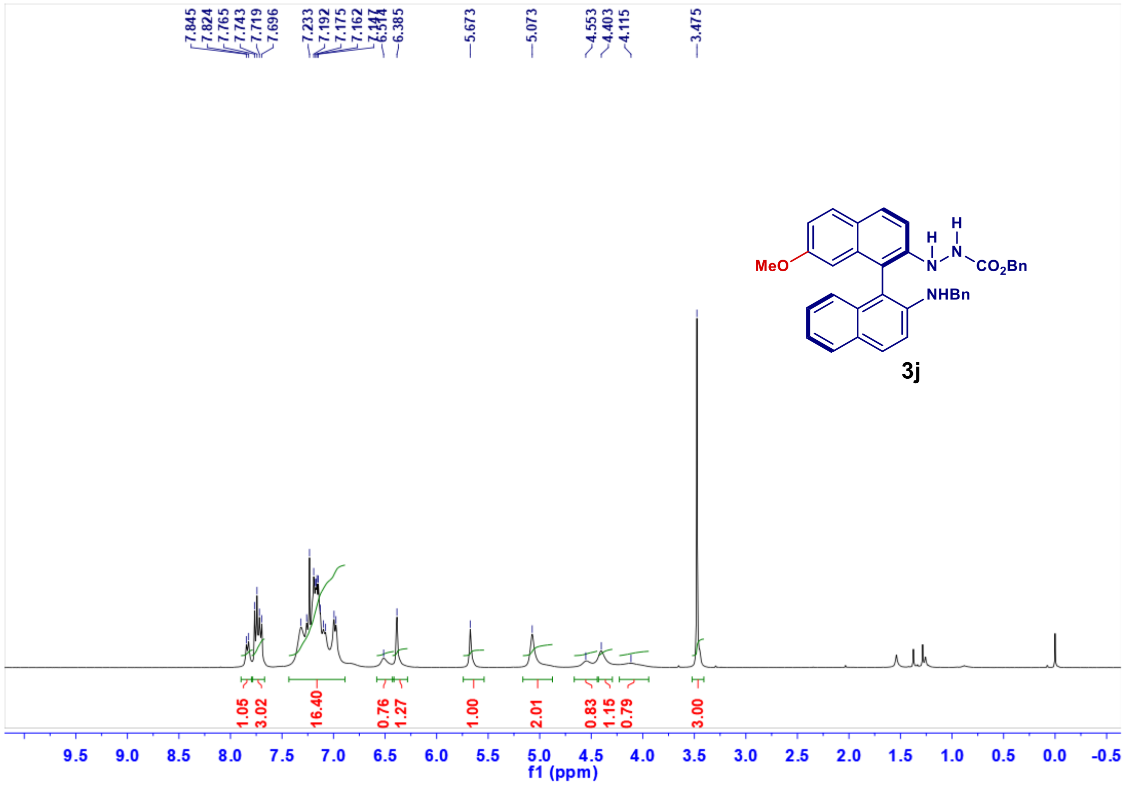

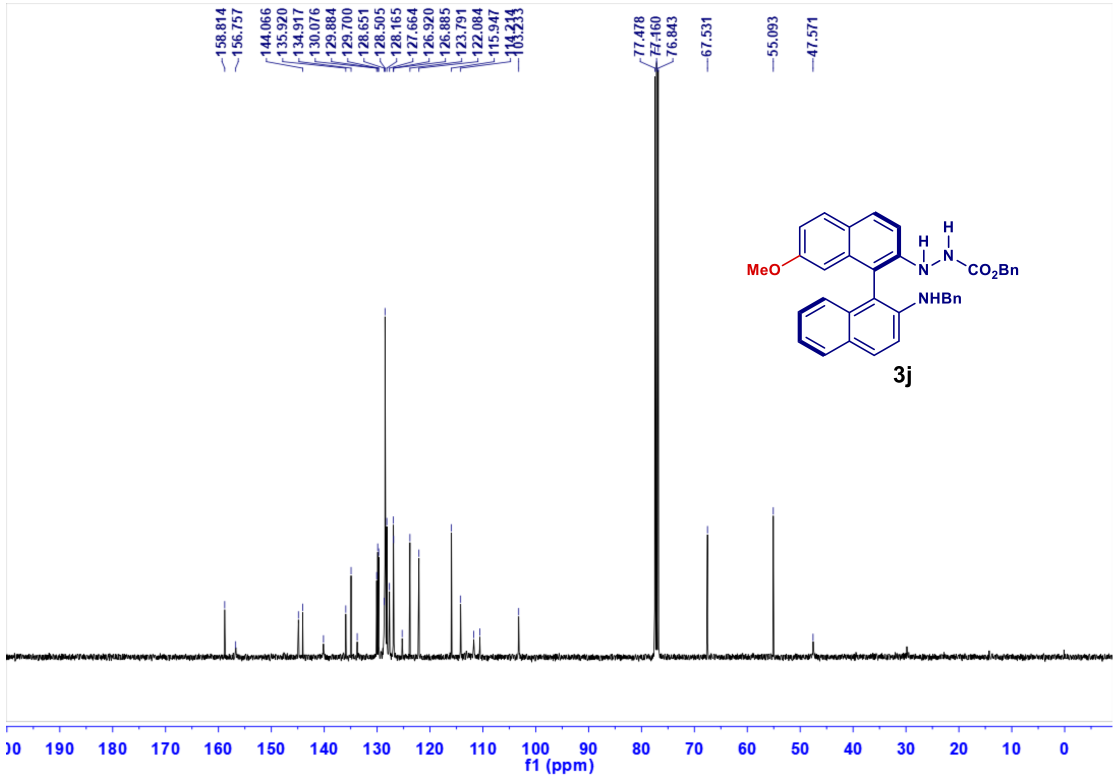

Supplementary Figure 46. ^{1}H and ^{13}C NMR spectra of 3j

Supplementary Figure 47. HPLC spectra of (*R*)-benzyl 2-(2'-(benzylamino)-7-methoxy-[1,1'-binaphthalen]-2-yl)hydrazine-1-carboxylate (3j). Diacel Chiralpak AD-H, *n*-Hexane:*i*-PrOH = 85:15, flow = 1.0 mL/min, 25 °C, λ = 254 nm, t_R(major) = 12.9 min, t_R(minor) = 18.2 min, e.r. = 97:3

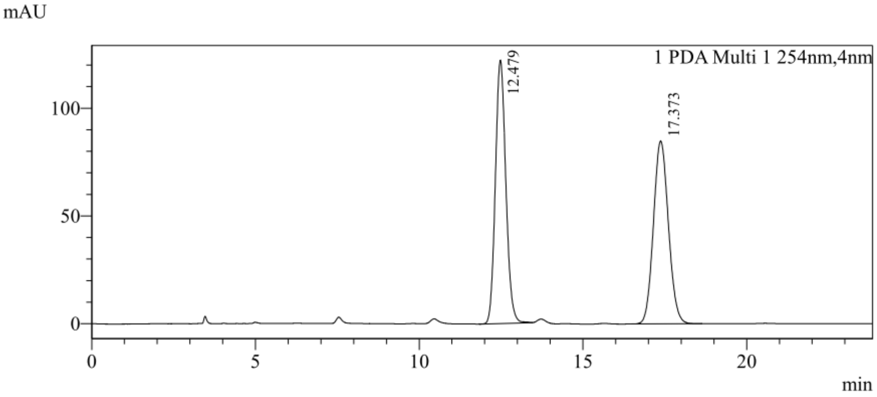

Peak Table

PDA Ch1 254nm

Peak#	Ret. Time	Peak End	Height	Area	Area%
1	12.479	13.451	122104	2710224	50.115
2	17.373	18.624	84867	2697823	49.885
Total			206972	5408047	100.000

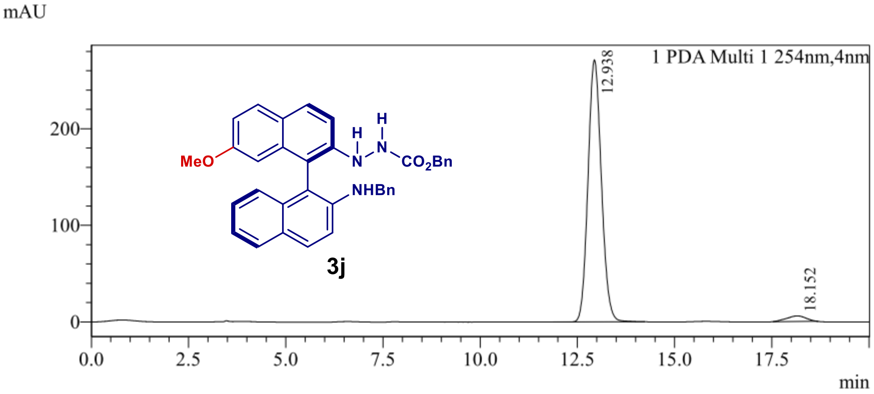

Peak Table

PDA Ch1 254nm

Peak#	Ret. Time	Peak End	Height	Area	Area%
1	12.938	14.219	271185	6483923	96.992
2	18.152	18.677	5771	201063	3.008
Total			276956	6684986	100.000

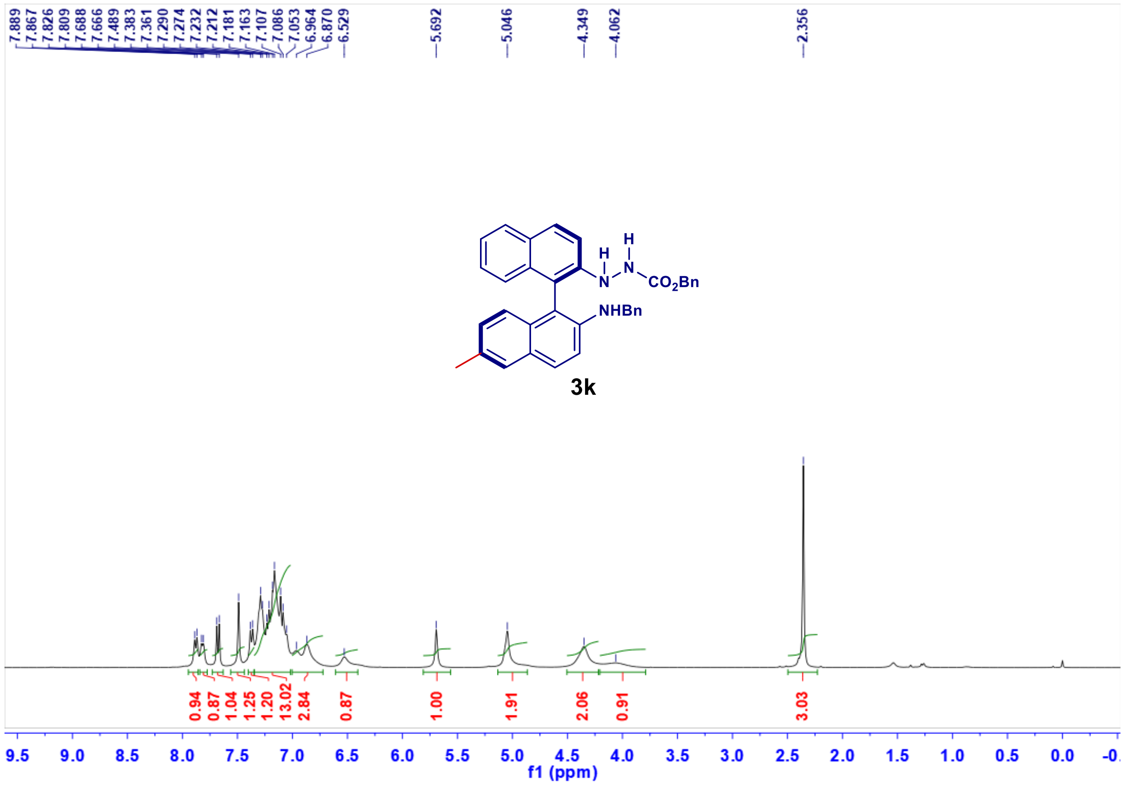

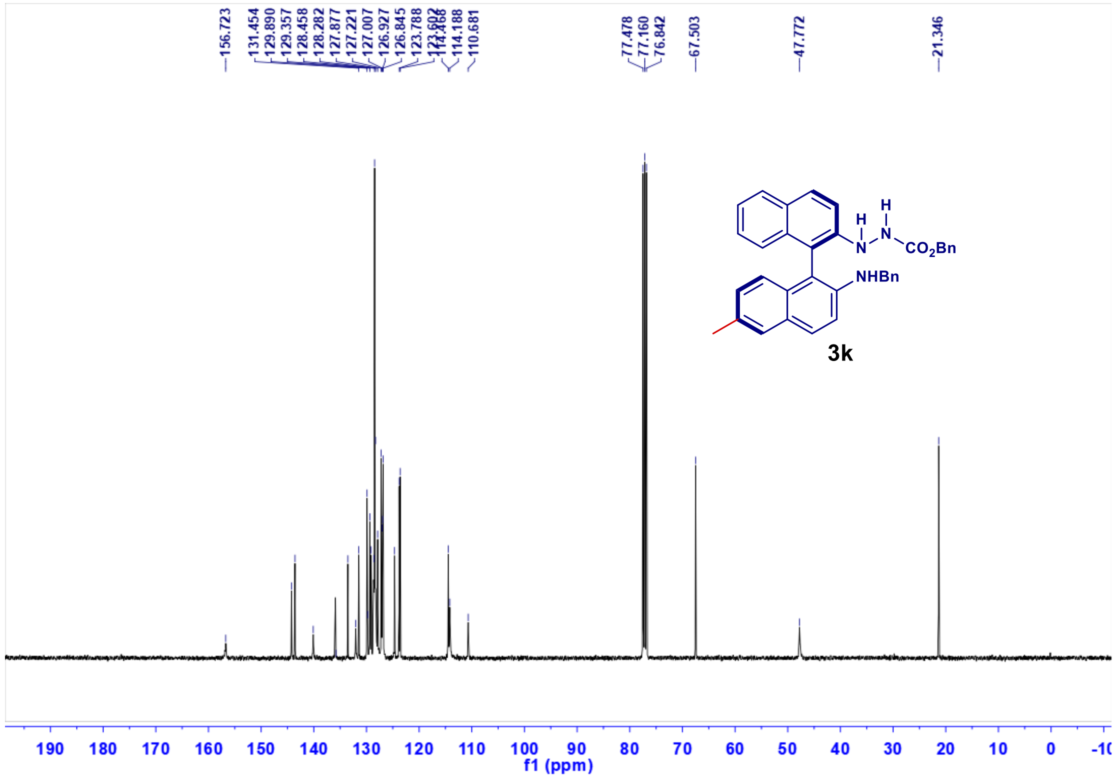

Supplementary Figure 48. ^{1}H and ^{13}C NMR spectra of 3k

Supplementary Figure 49. HPLC spectra of (*R*)-benzyl 2-(2'-(benzylamino)-6'-methyl-[1,1'-binaphthalen]-2-yl)hydrazine-1-carboxylate (3k). Diacel Chiralpak AD-H, *n*-Hexane:*i*-PrOH = 85:15, flow = 1.0 mL/min, 25 °C, λ = 254 nm, t_R(major) = 10.7 min, t_R(minor) = 14.2 min, e.r. = 97.5:2.5

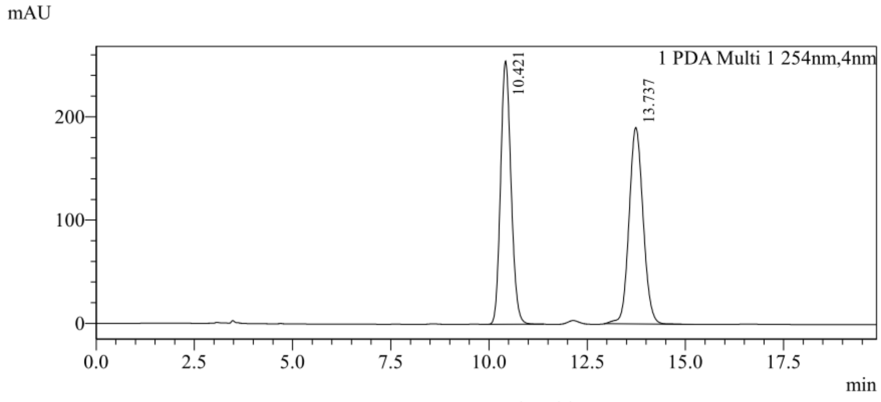

Peak Table

PDA Ch1 254nm

Peak#	Ret. Time	Peak End	Height	Area	Area%
1	10.421	11.381	254758	4715514	49.850
2	13.737	15.200	190224	4743858	50.150
Total			444982	9459372	100.000

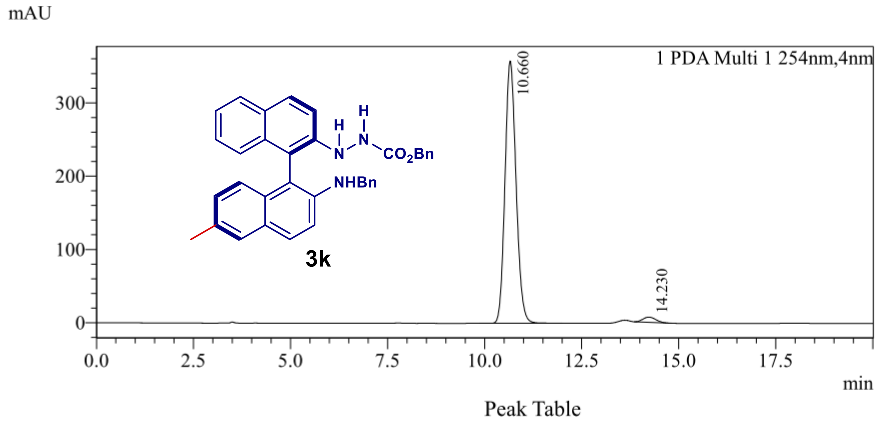

Peak Table

PDA Ch1 254nm

Peak#	Ret. Time	Peak End	Height	Area	Area%
1	10.660	11.989	357539	7086872	97.716
2	14.230	14.827	7149	165643	2.284
Total			364689	7252515	100.000

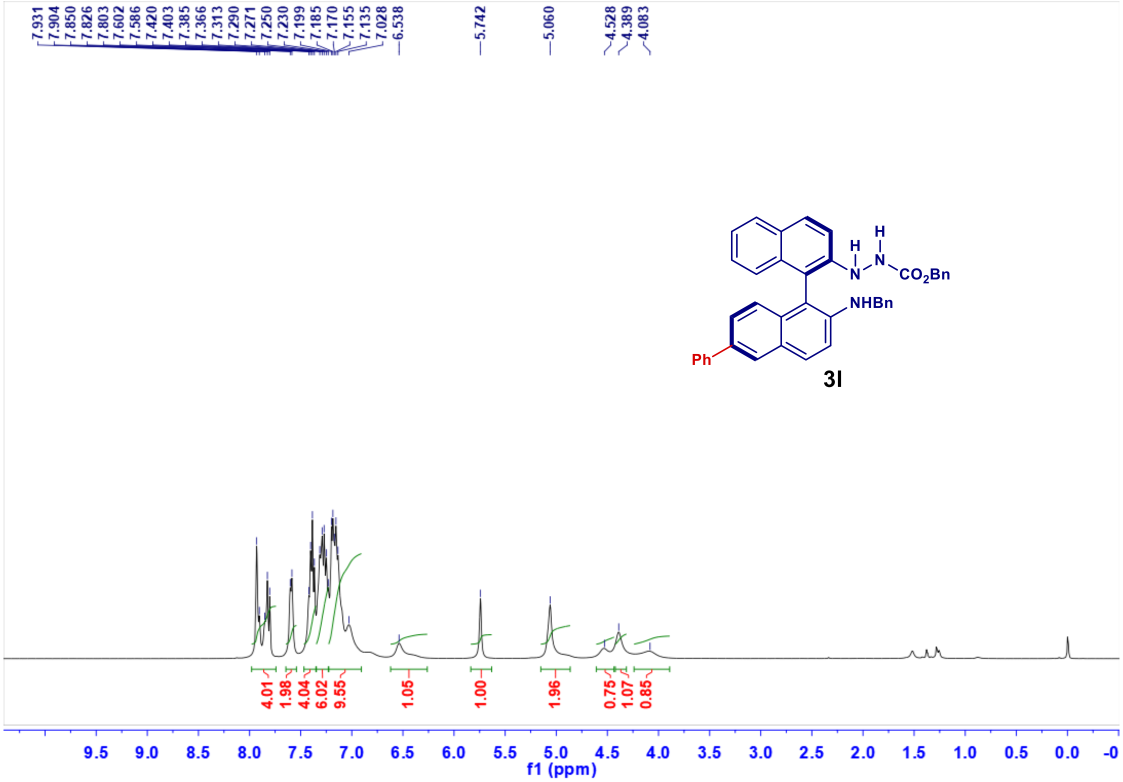

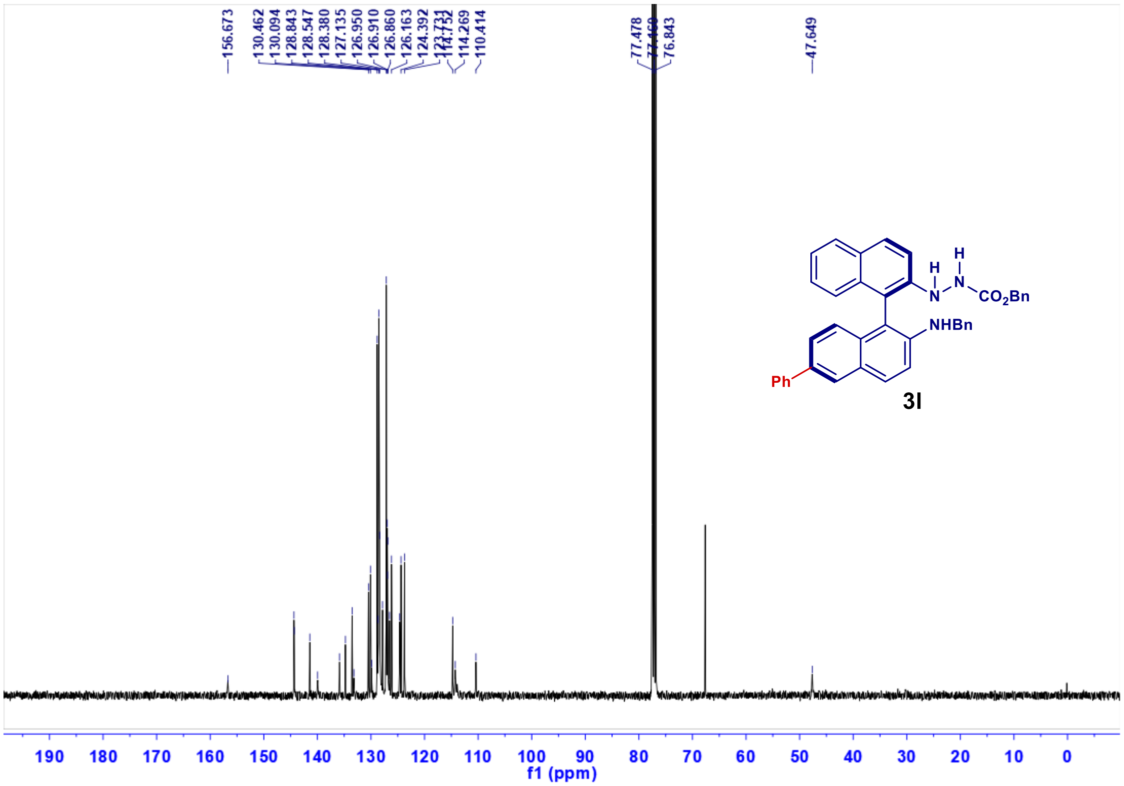

Supplementary Figure 50. ^{1}H **and** ^{13}C **NMR spectra of 3l**

Supplementary Figure 51. HPLC spectra of (*R*)-benzyl 2-(2'-(benzylamino)-6'-phenyl-[1,1'-binaphthalen]-2-yl)hydrazine-1-carboxylate (3l). Diacel Chiralpak AD-H, *n*-Hexane:*i*-PrOH = 70:30, flow = 1.0 mL/min, 25 °C, λ = 254 nm, t_R(major) = 8.8 min, t_R(minor) = 15.1 min, e.r. = 96:4

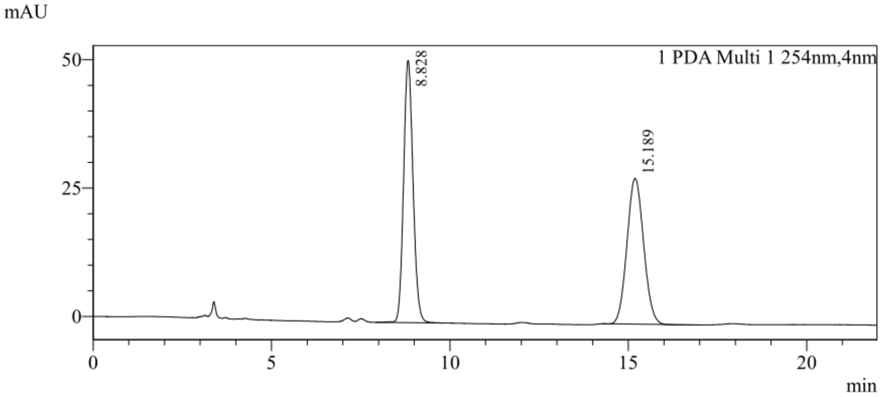

Peak Table

PDA Ch1 254nm

Peak#	Ret. Time	Peak End	Height	Area	Area%
1	8.828	9.813	51085	941264	50.540
2	15.189	17.077	28411	921153	49.460
Total			79496	1862417	100.000

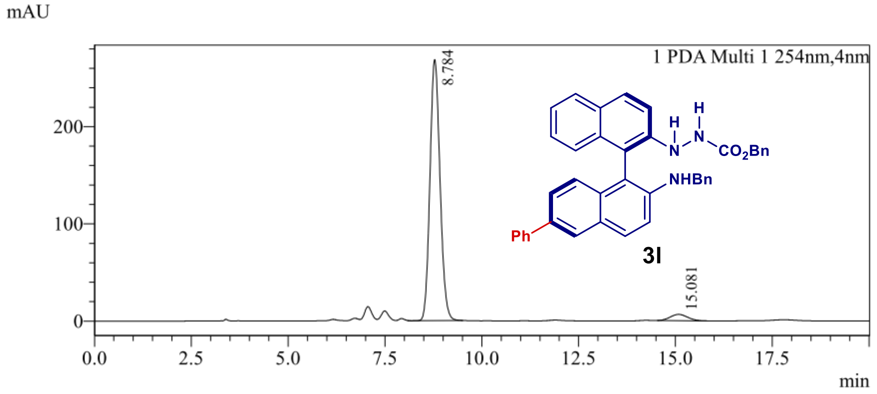

Peak Table

PDA Ch1 254nm

Peak#	Ret. Time	Peak End	Height	Area	Area%
1	8.784	9.504	268139	4843028	96.013
2	15.081	15.829	6457	201097	3.987
Total			274596	5044125	100.000

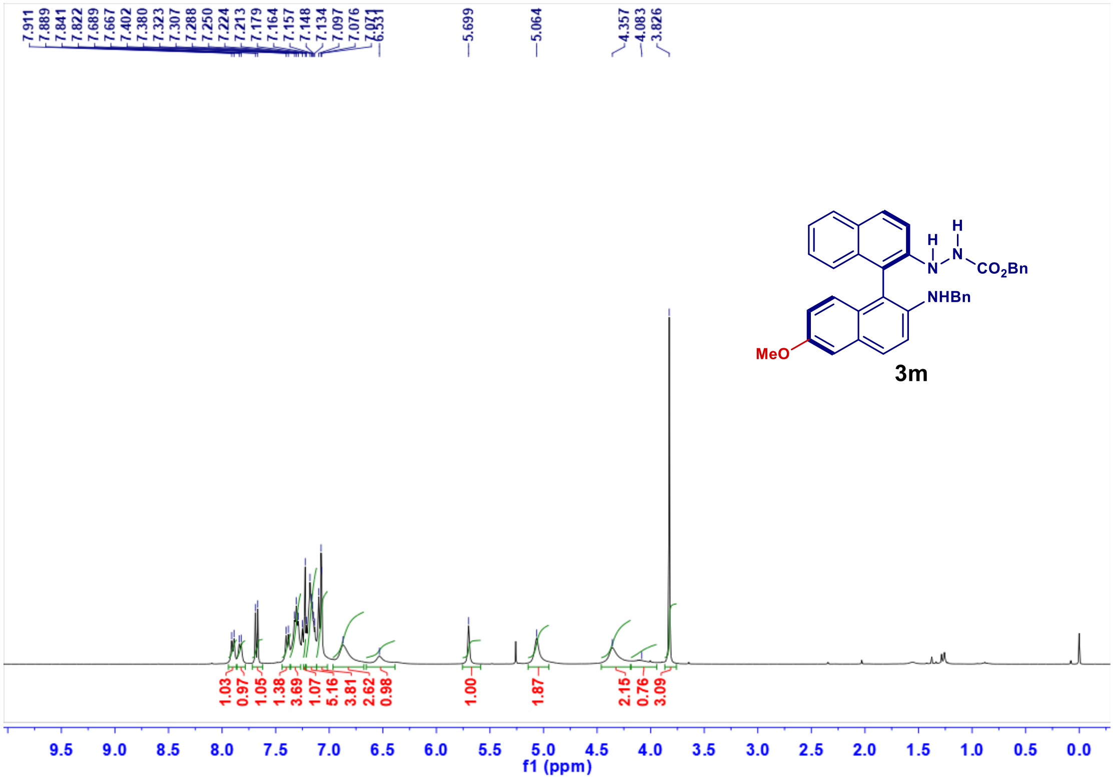

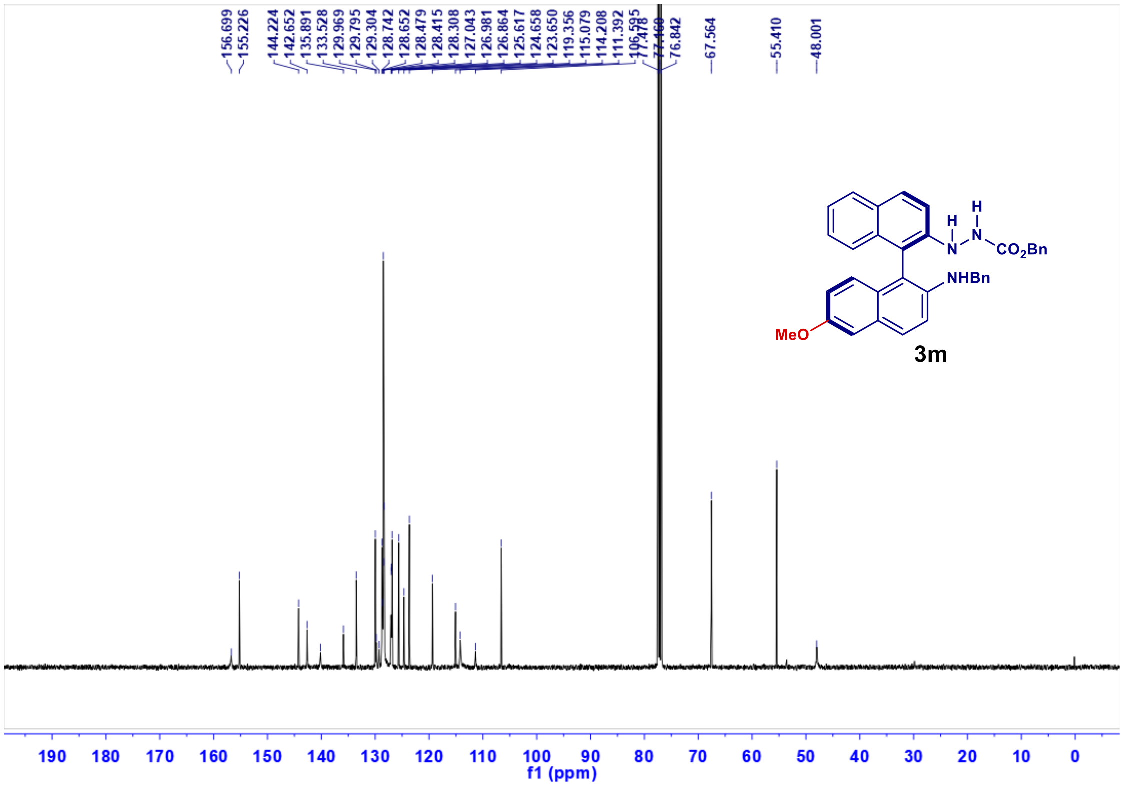

Supplementary Figure 52. ^{1}H and ^{13}C NMR spectra of 3m

Supplementary Figure 53. HPLC spectra of (*R*)-benzyl 2-(2'-(benzylamino)-6'-methoxy-[1,1'-binaphthalen]-2-yl)hydrazine-1-carboxylate (3m). Diacel Chiralcel OD-H, *n*-Hexane:*i*-PrOH = 80:20, flow = 1.0 mL/min, 25 °C, λ = 254 nm, t_R(major) = 12.8 min, t_R(minor) = 18.8 min, e.r. = 98:2

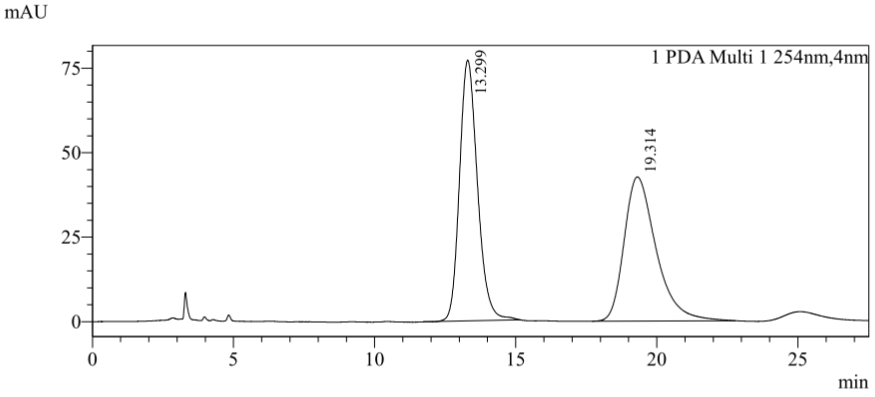

Peak Table

PDA Ch1 254nm

Peak#	Ret. Time	Peak End	Height	Area	Area%
1	13.299	15.168	77187	3419458	50.407
2	19.314	22.784	42631	3364202	49.593
Total			119817	6783659	100.000

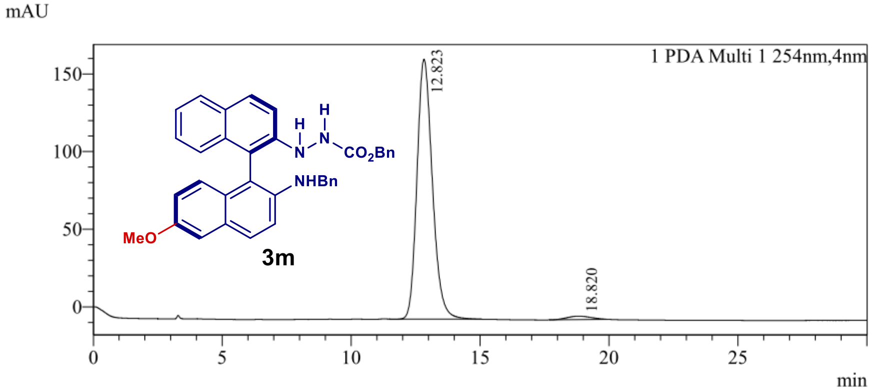

Peak Table

PDA Ch1 254nm

Peak#	Ret. Time	Peak End	Height	Area	Area%
1	12.823	15.029	167401	6962276	97.999
2	18.820	19.915	2190	142178	2.001
Total			169592	7104454	100.000

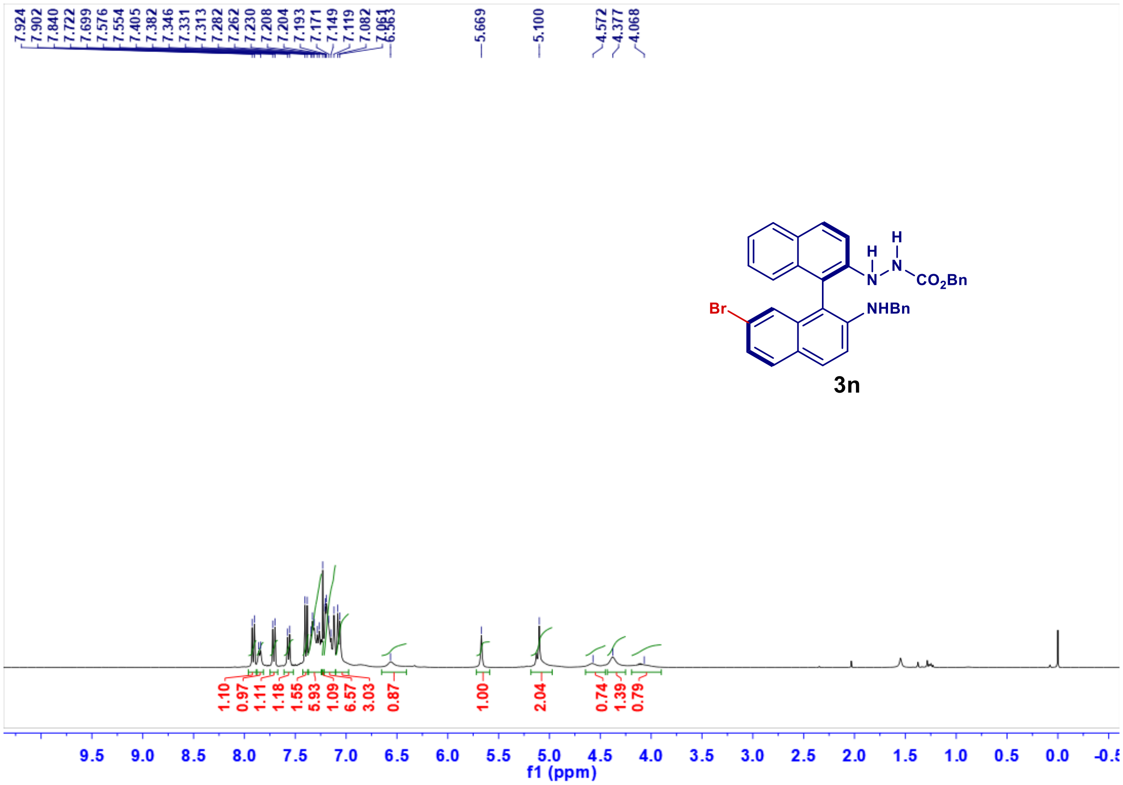

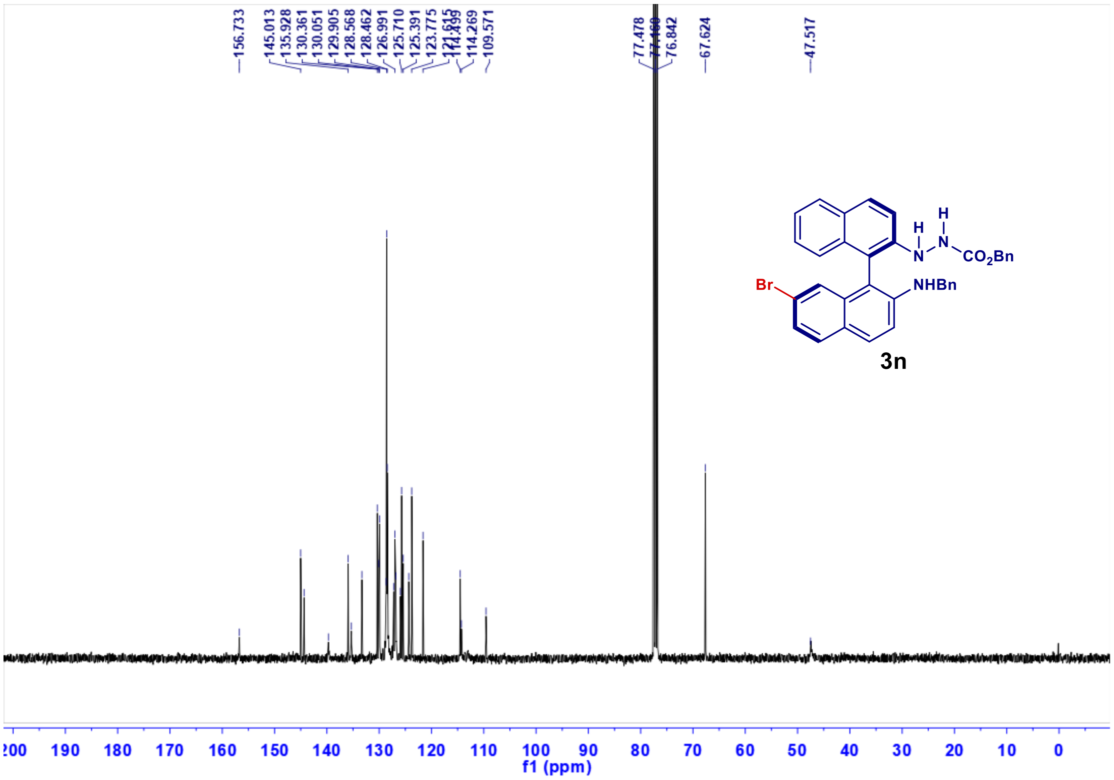

Supplementary Figure 54. ^{1}H and ^{13}C NMR spectra of 3n

Supplementary Figure 55. HPLC spectra of (*R*)-benzyl 2-(2'-(benzylamino)-7'-bromo-[1,1'-binaphthalen]-2-yl)hydrazine-1-carboxylate (3n). Diacel Chiralpak AD-H, *n*-Hexane:*i*-PrOH = 85:15, flow = 1.0 mL/min, 25 °C, λ = 254 nm, t_R(major) = 10.1 min, t_R(minor) = 8.6 min, e.r. = 96:4

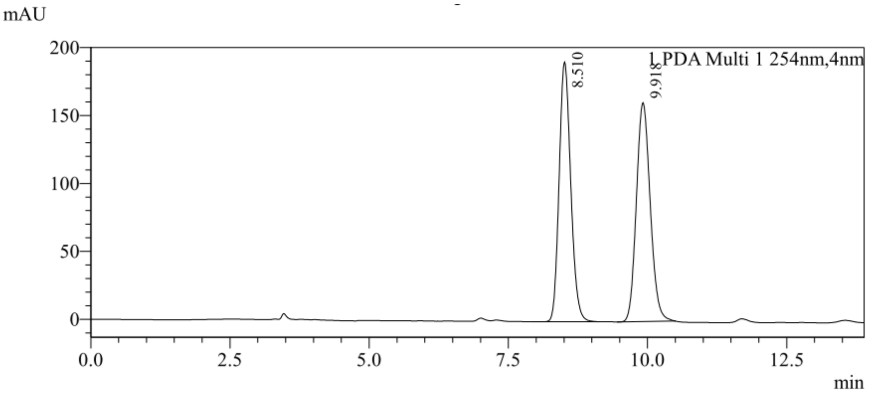

Peak Table

PDA Ch1 254nm

Peak#	Ret. Time	Peak End	Height	Area	Area%
1	8.510	9.131	191108	2767202	49.841
2	9.918	10.496	161116	2784859	50.159
Total			352223	5552061	100.000

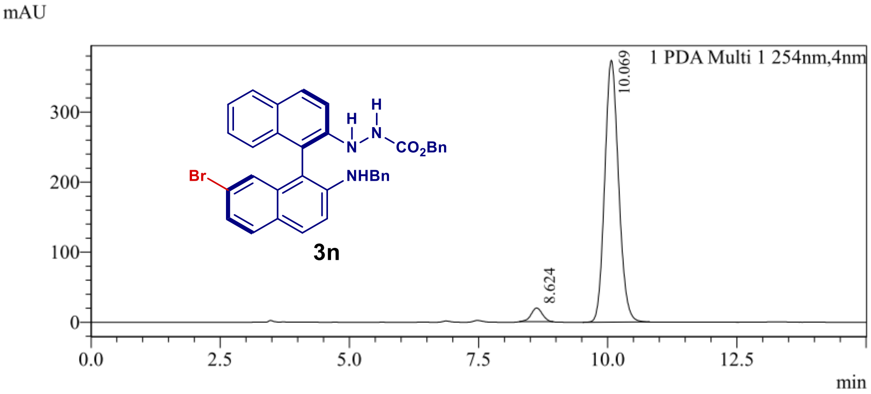

Peak Table

PDA Ch1 254nm

Peak#	Ret. Time	Peak End	Height	Area	Area%
1	8.624	8.939	19464	296005	4.070
2	10.069	10.805	373483	6975990	95.930
Total			392947	7271994	100.000

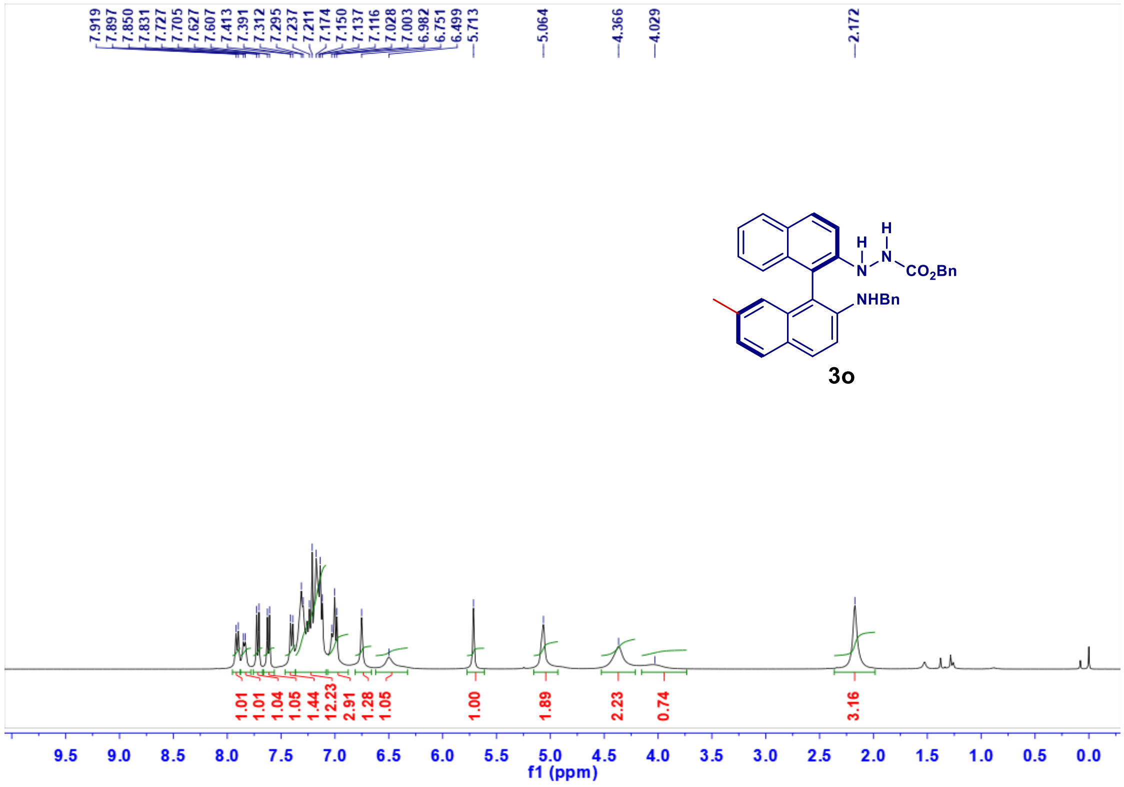

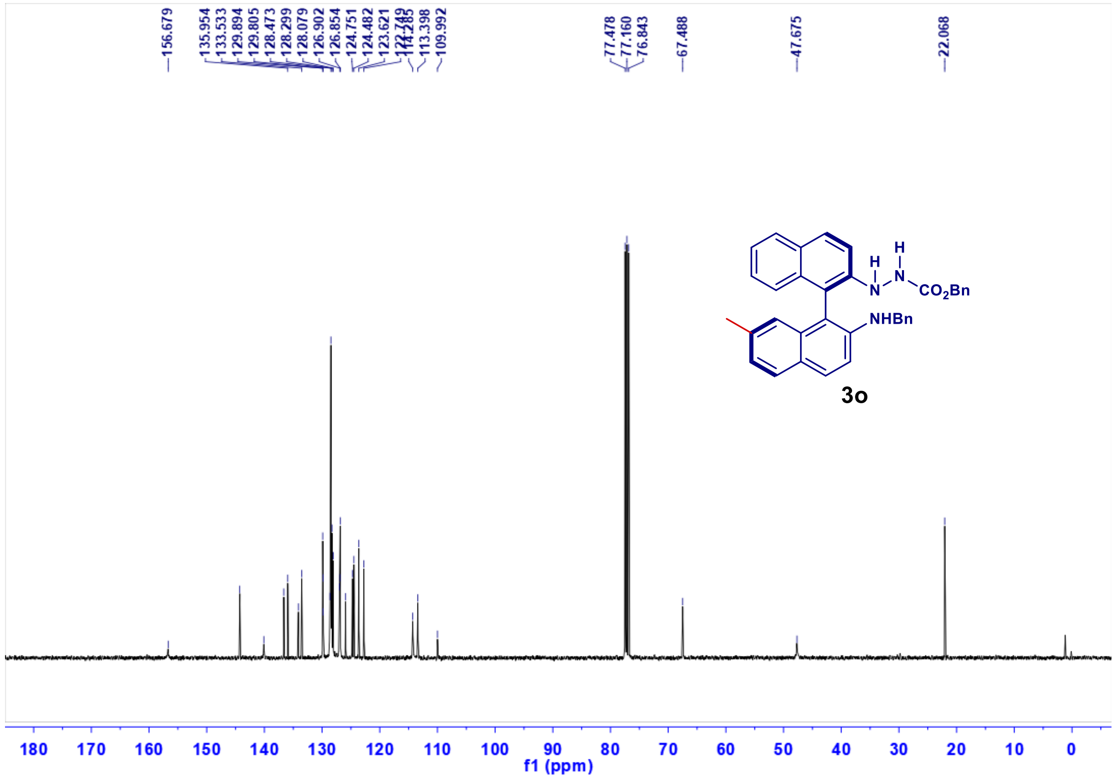

Supplementary Figure 56. ^{1}H and ^{13}C NMR spectra of 3o

Supplementary Figure 57. HPLC spectra of (*R*)-benzyl 2-(2'-(benzylamino)-7'-methyl-[1,1'-binaphthalen]-2-yl)hydrazine-1-carboxylate (3o). Diacel Chiralpak AD-H, *n*-Hexane:*i*-PrOH = 85:15, flow = 1.0 mL/min, 25 °C, λ = 254 nm, t_R(major) = 9.9 min, t_R(minor) = 8.9 min, e.r. = 98:2

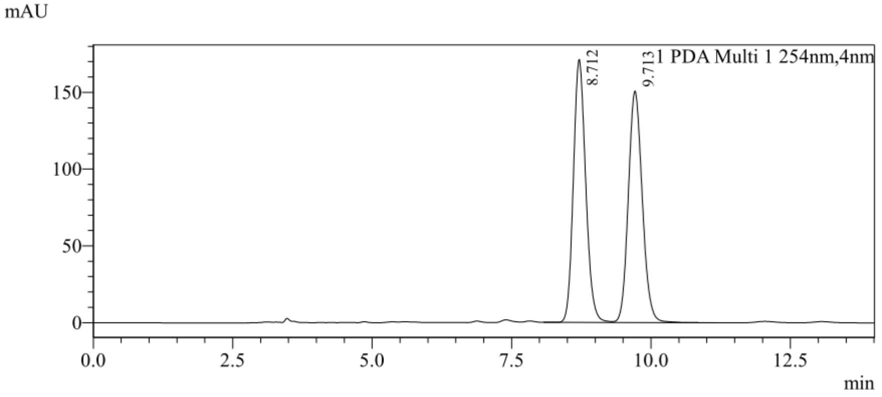

Peak Table

PDA Ch1 254nm

Peak#	Ret. Time	Peak End	Height	Area	Area%
1	8.712	9.301	171131	2637570	50.122
2	9.713	11.168	150726	2624709	49.878
Total			321856	5262278	100.000

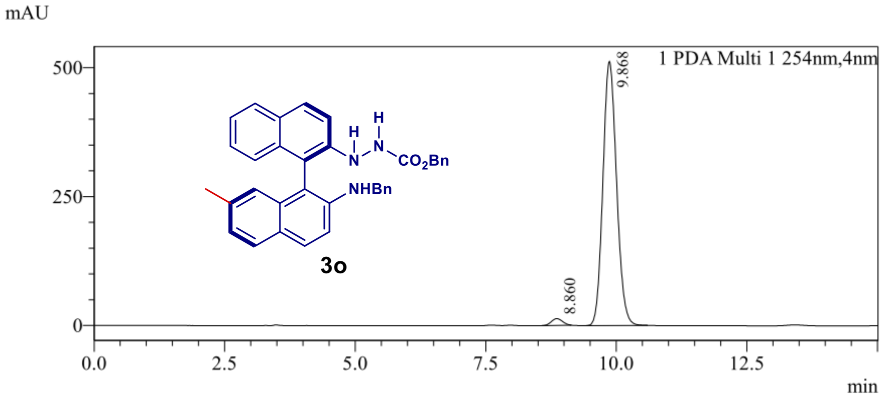

Peak Table

PDA Ch1 254nm

Peak#	Ret. Time	Peak End	Height	Area	Area%
1	8.860	9.131	13111	192887	2.029
2	9.868	10.592	512189	9314313	97.971
Total			525301	9507200	100.000

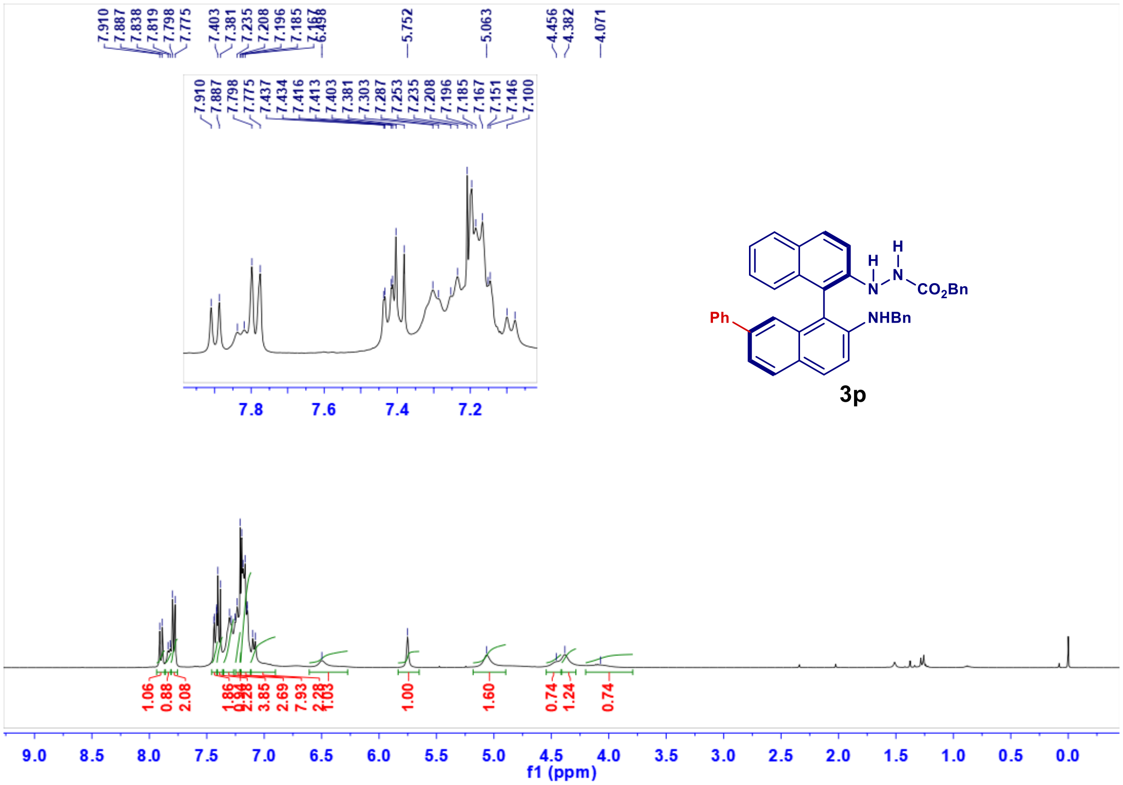

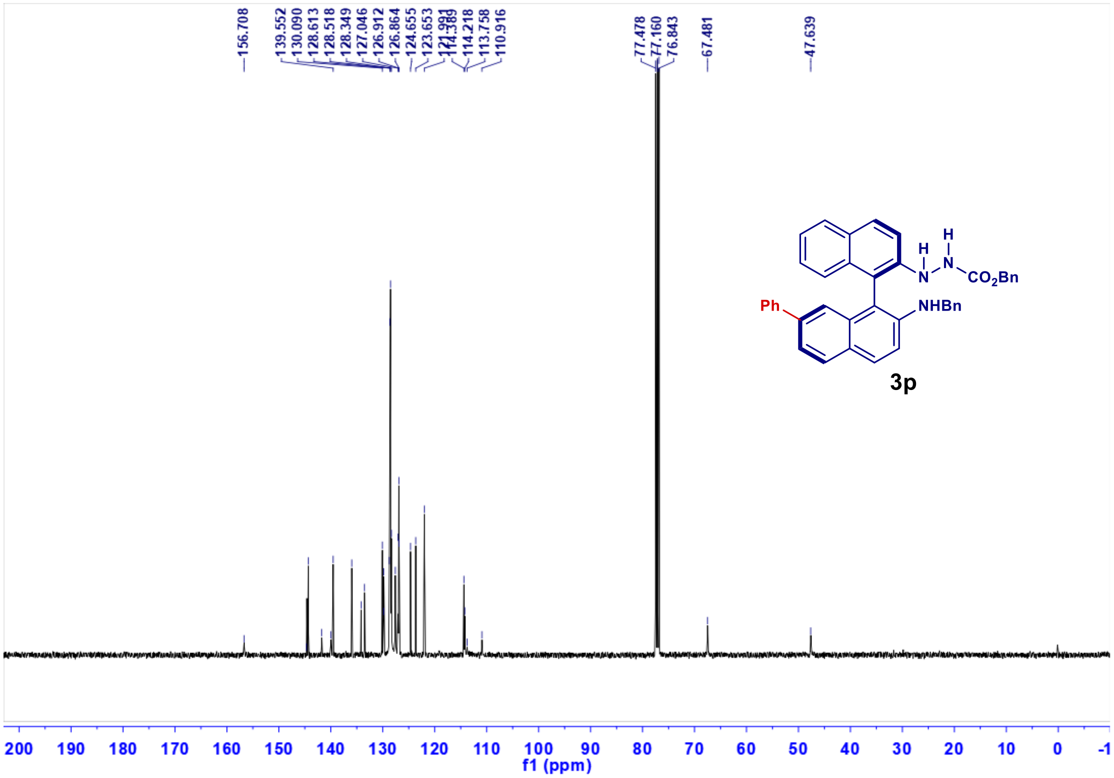

Supplementary Figure 58. ^{1}H **and** ^{13}C **NMR spectra of 3p**

Supplementary Figure 59. HPLC spectra of (*R*)-benzyl 2-(2'-(benzylamino)-7'-phenyl-[1,1'-binaphthalen]-2-yl)hydrazine-1-carboxylate (3p). Diacel Chiralpak AD-H, *n*-Hexane:*i*-PrOH = 85:15, flow = 1.0 mL/min, 25 °C, λ = 254 nm, t_R(major) = 11.1min, t_R(minor) = 10.0 min, e.r. = 98:2

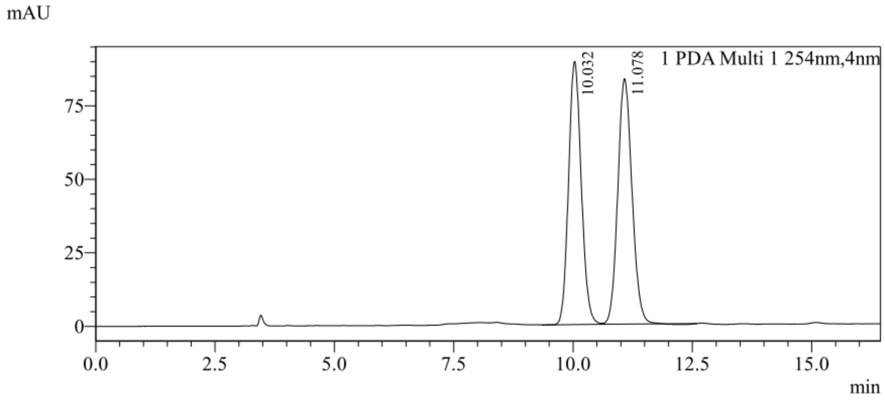

Peak Table

PDA Ch1 254nm

Peak#	Ret. Time	Peak End	Height	Area	Area%
1	10.032	10.603	89448	1659677	49.028
2	11.078	12.587	83397	1725488	50.972
Total			172845	3385165	100.000

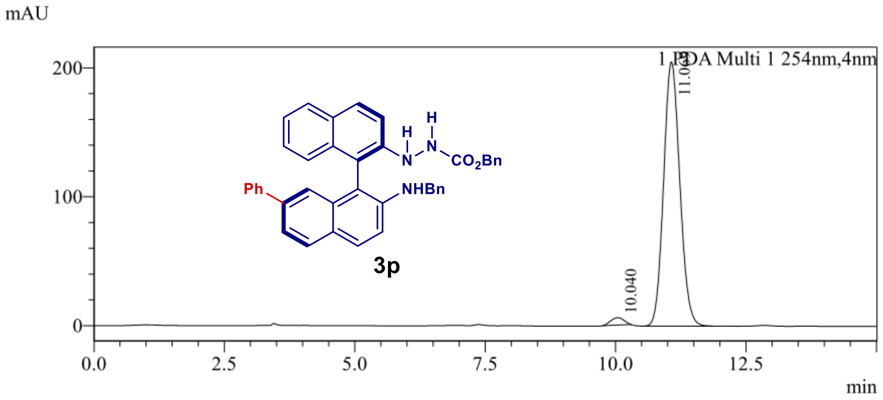

Peak Table

PDA Ch1 254nm

Peak#	Ret. Time	Peak End	Height	Area	Area%
1	10.040	10.293	5723	99796	2.204
2	11.069	11.872	204961	4427934	97.796
Total			210683	4527730	100.000

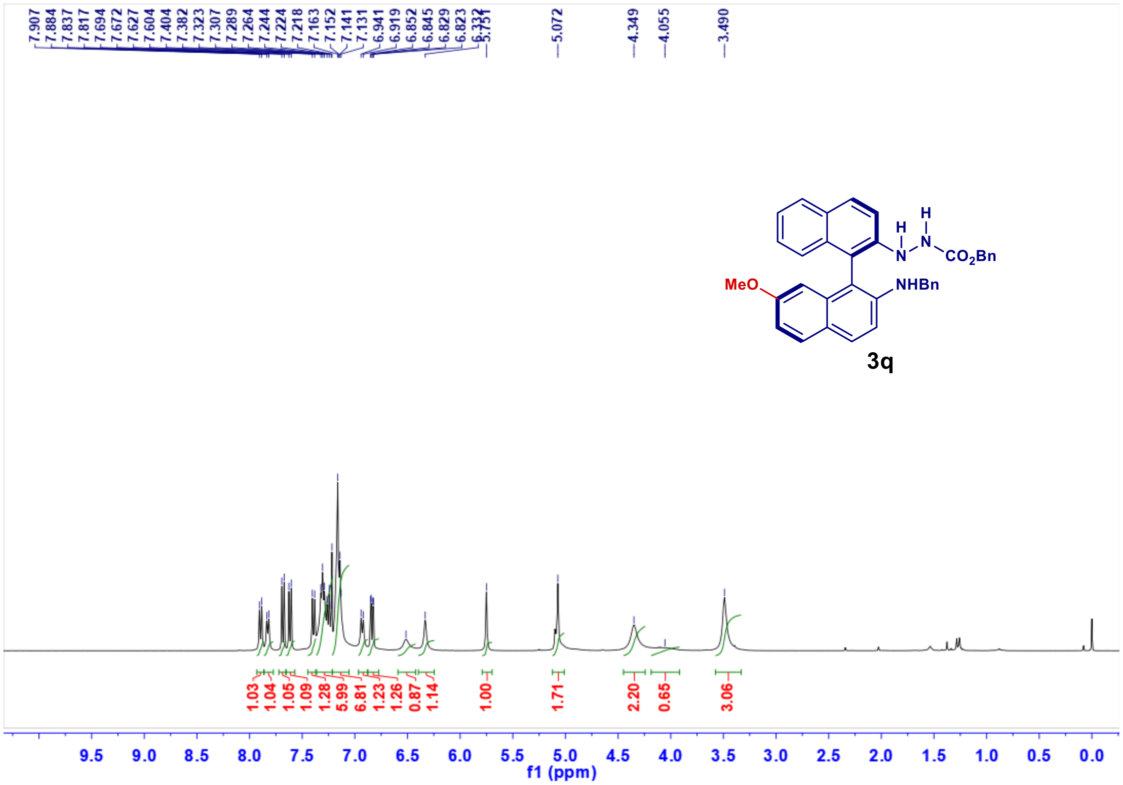

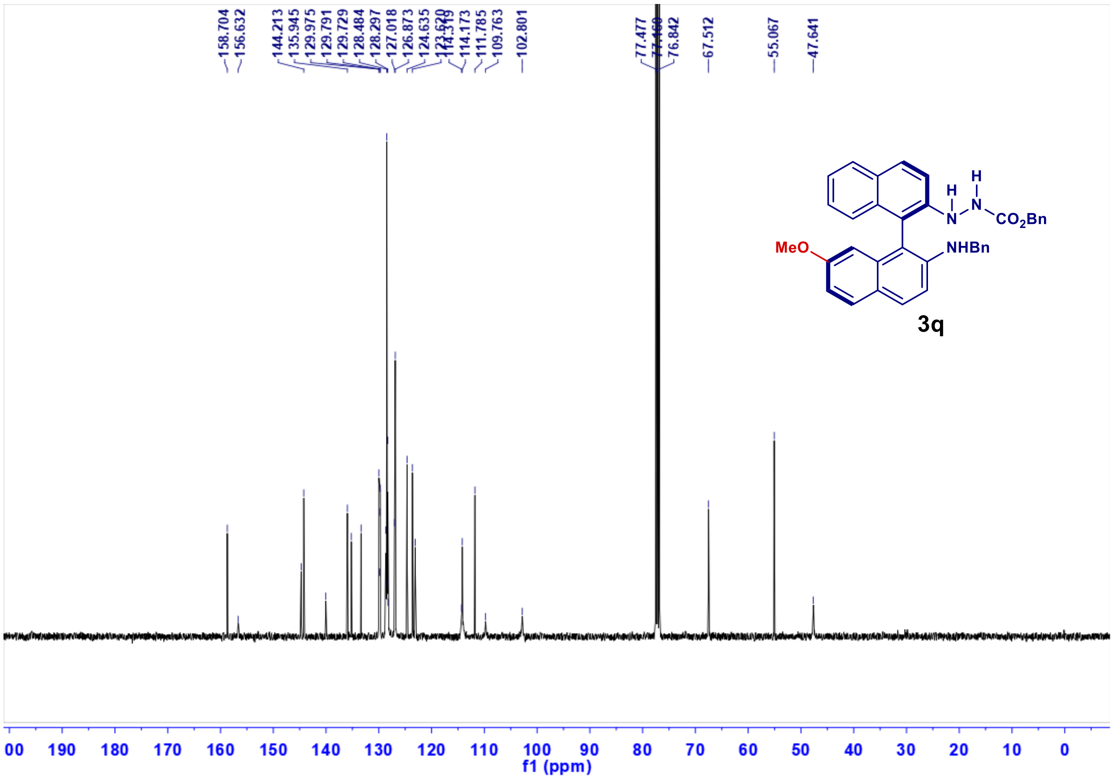

Supplementary Figure 60. ^{1}H and ^{13}C NMR spectra of 3q

Supplementary Figure 61. HPLC spectra of (*R*)-benzyl 2-(2'-(benzylamino)-7'-methoxy-[1,1'-binaphthalen]-2-yl)hydrazine-1-carboxylate (3q). Diacel Chiralcel OD-H, *n*-Hexane:*i*-PrOH = 80:20, flow = 1.0 mL/min, 25 °C, λ = 254 nm, t_R(major) = 10.1 min, t_R(minor) = 11.6 min, e.r. = 97:3

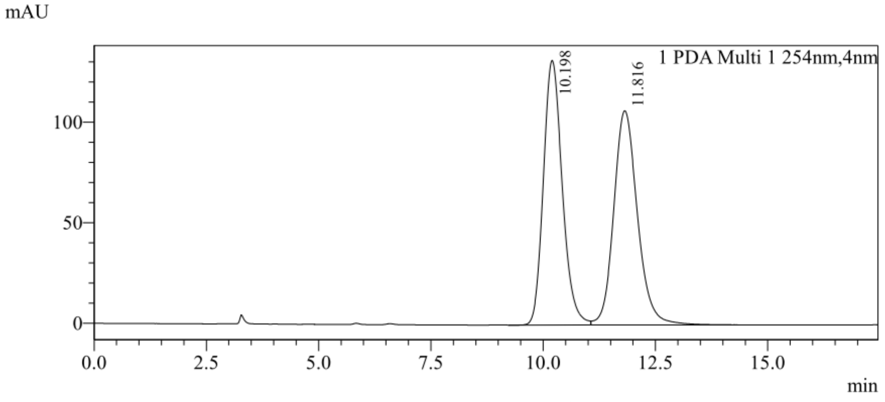

Peak Table

PDA Ch1 254nm

Peak#	Ret. Time	Peak End	Height	Area	Area%
1	10.198	11.061	131534	3844916	49.603
2	11.816	14.336	106498	3906464	50.397
Total			238032	7751380	100.000

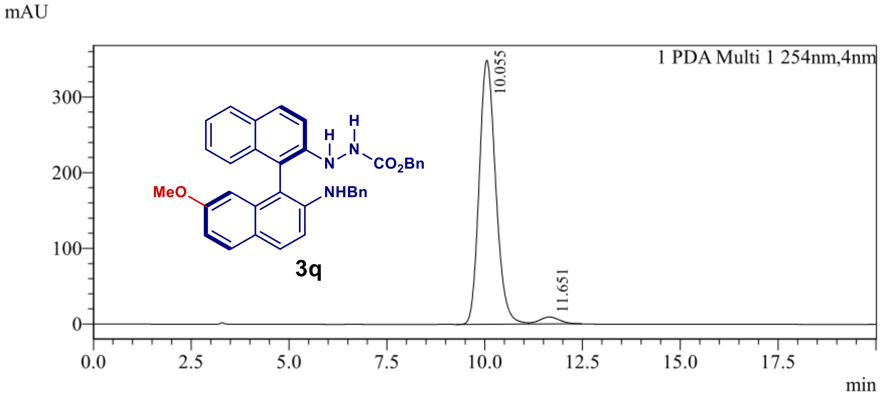

Peak Table

PDA Ch1 254nm

Peak#	Ret. Time	Peak End	Height	Area	Area%
1	10.055	11.125	348786	10255973	96.803
2	11.651	12.469	9210	338714	3.197
Total			357996	10594687	100.000

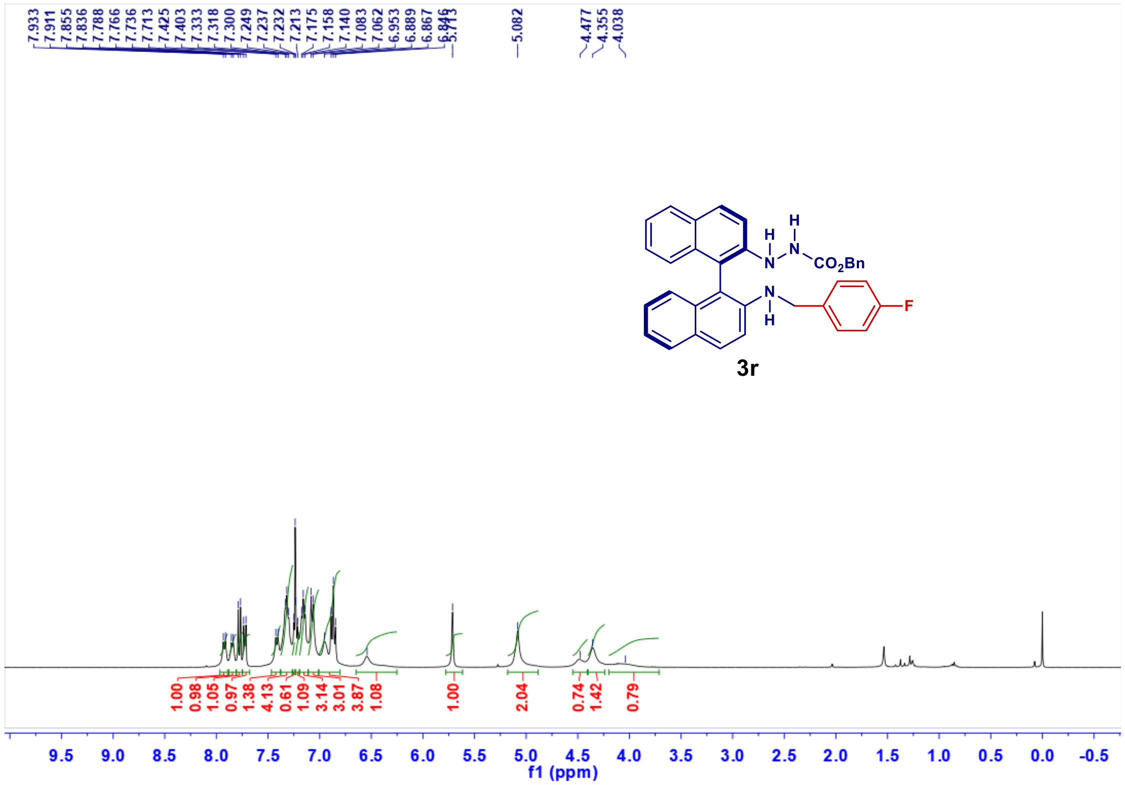

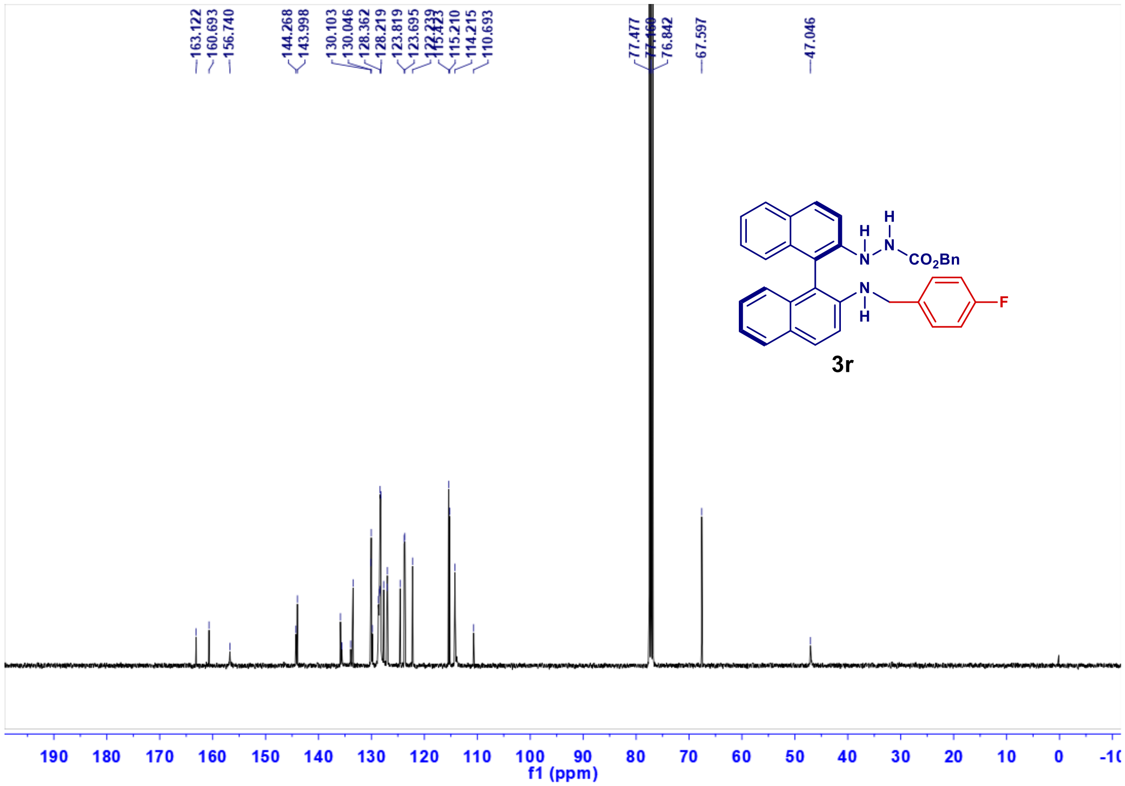

Supplementary Figure 62. ^{1}H and ^{13}C NMR spectra of 3r

Supplementary Figure 63. HPLC spectra of (*R*)-benzyl 2-(2'-((4-fluorobenzyl)amino)-[1,1'-binaphthalen]-2-yl)hydrazine-1-carboxylate (3r). Diacel Chiralpak AD-H, *n*-Hexane:*i*-PrOH = 85:15, flow = 1.0 mL/min, 25 °C, λ = 254 nm, t_R(major) = 13.6 min, t_R(minor) = 12.5 min, e.r. = 96.5:3.5

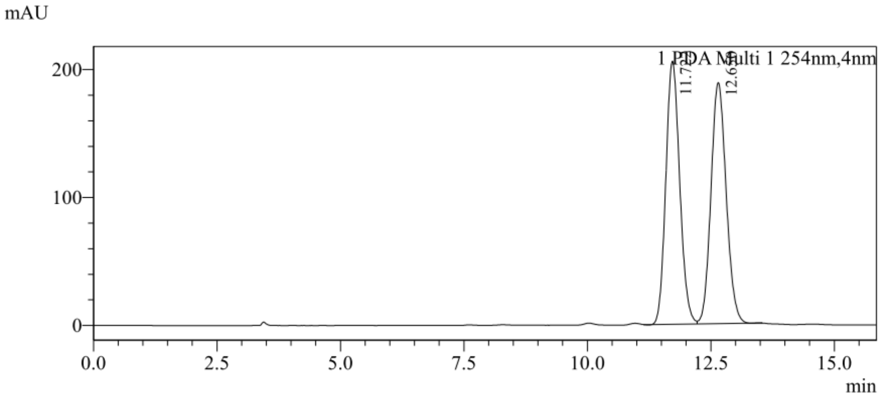

Peak Table

PDA Ch1 254nm

Peak#	Ret. Time	Peak End	Height	Area	Area%
1	11.722	12.224	205488	4021879	49.870
2	12.650	13.536	188390	4042901	50.130
Total			393878	8064780	100.000

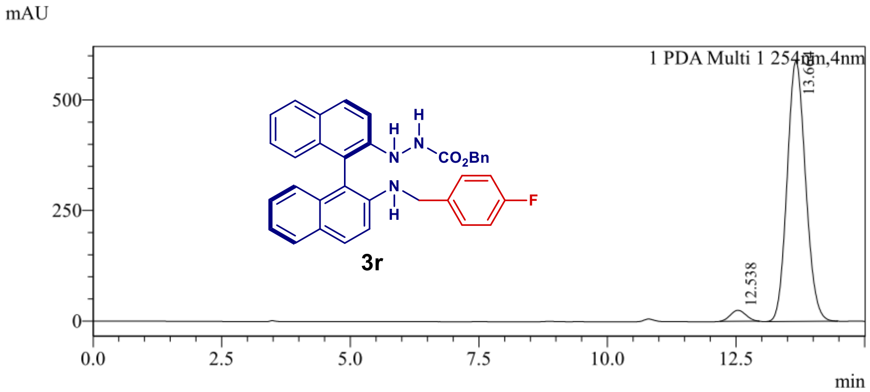

Peak Table

PDA Ch1 254nm

Peak#	Ret. Time	Peak End	Height	Area	Area%
1	12.538	12.949	24731	519233	3.412
2	13.664	14.475	588708	14699481	96.588
Total			613439	15218713	100.000

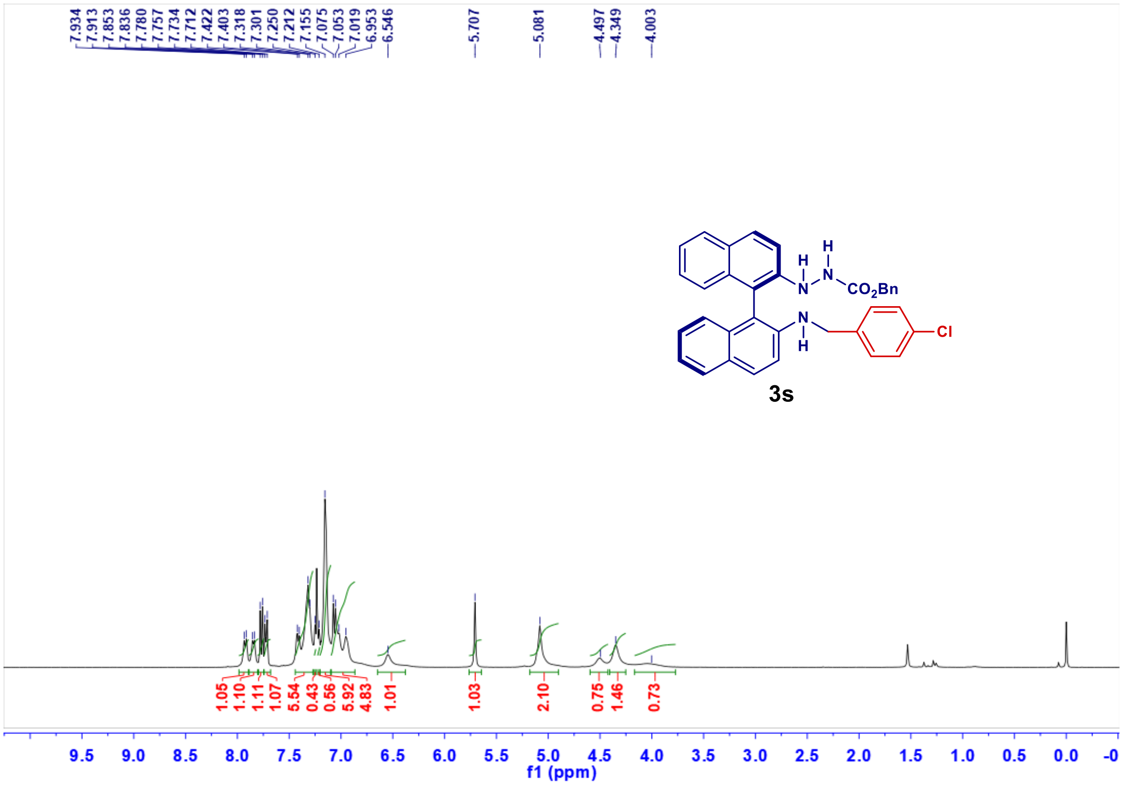

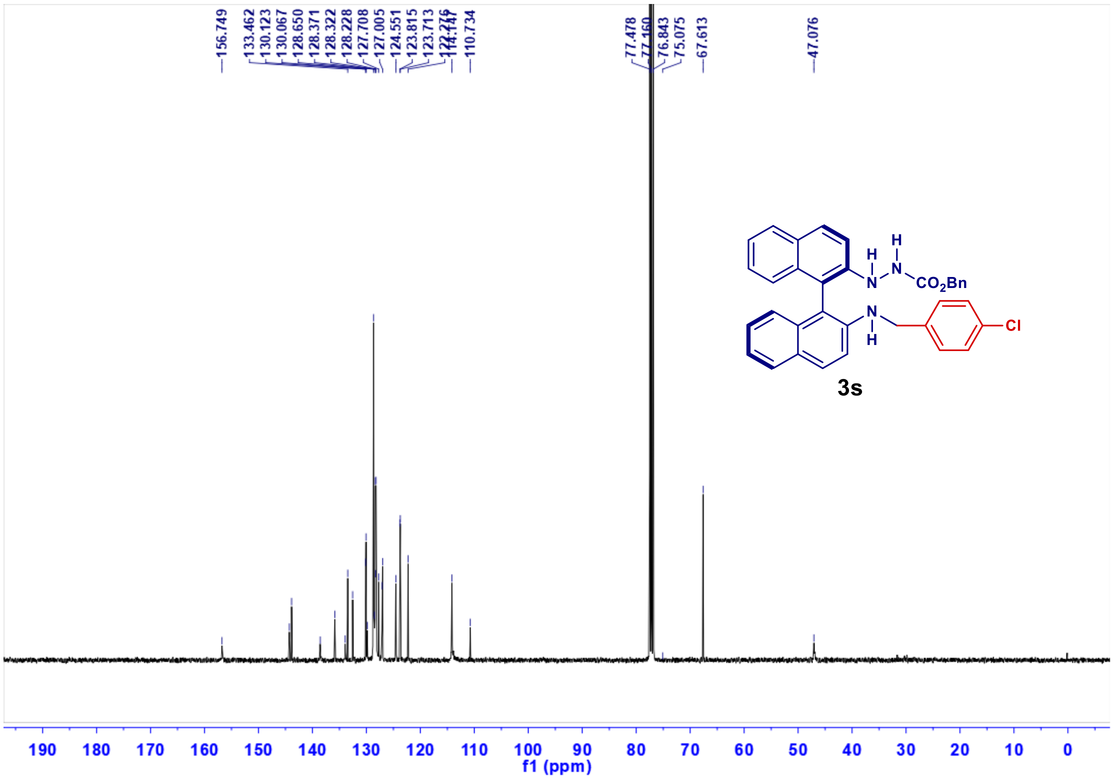

Supplementary Figure 64. ^{1}H and ^{13}C NMR spectra of 3s

Supplementary Figure 65. HPLC spectra of (*R*)-benzyl 2-(2’-((4-chlorobenzyl)amino)-[1,1’-binaphthalen]-2-yl)hydrazine-1-carboxylate (3s). Diacel Chiralcel OD-H, *n*-Hexane:*i*-PrOH = 80:20, flow = 1.0 mL/min, 25 °C, λ = 254 nm, t_R(major) = 11.1 min, t_R(minor) = 17.0 min, e.r. = 97.5:2.5

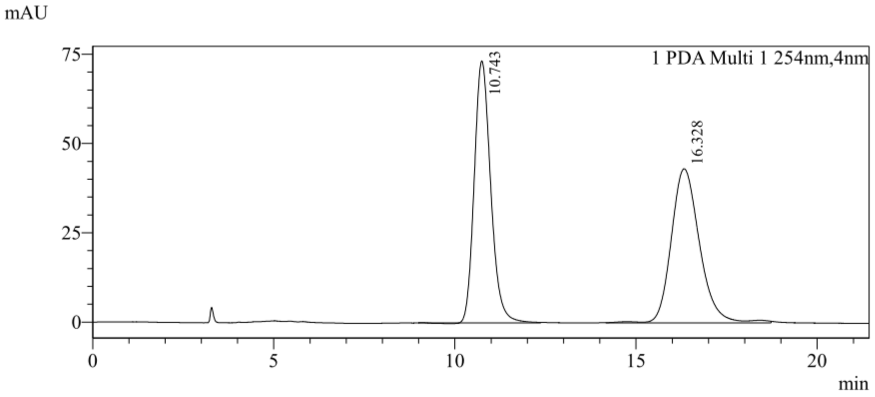

Peak Table

PDA Ch1 254nm

Peak#	Ret. Time	Peak End	Height	Area	Area%
1	10.743	12.373	73323	2314666	49.520
2	16.328	18.731	43190	2359491	50.480
Total			116513	4674157	100.000

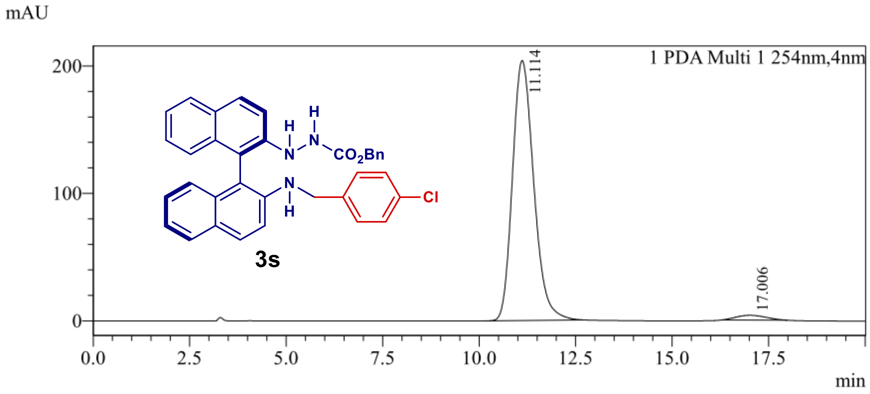

Peak Table

PDA Ch1 254nm

Peak#	Ret. Time	Peak End	Height	Area	Area%
1	11.114	12.693	203896	7826551	97.540
2	17.006	17.973	3708	197409	2.460
Total			207604	8023959	100.000

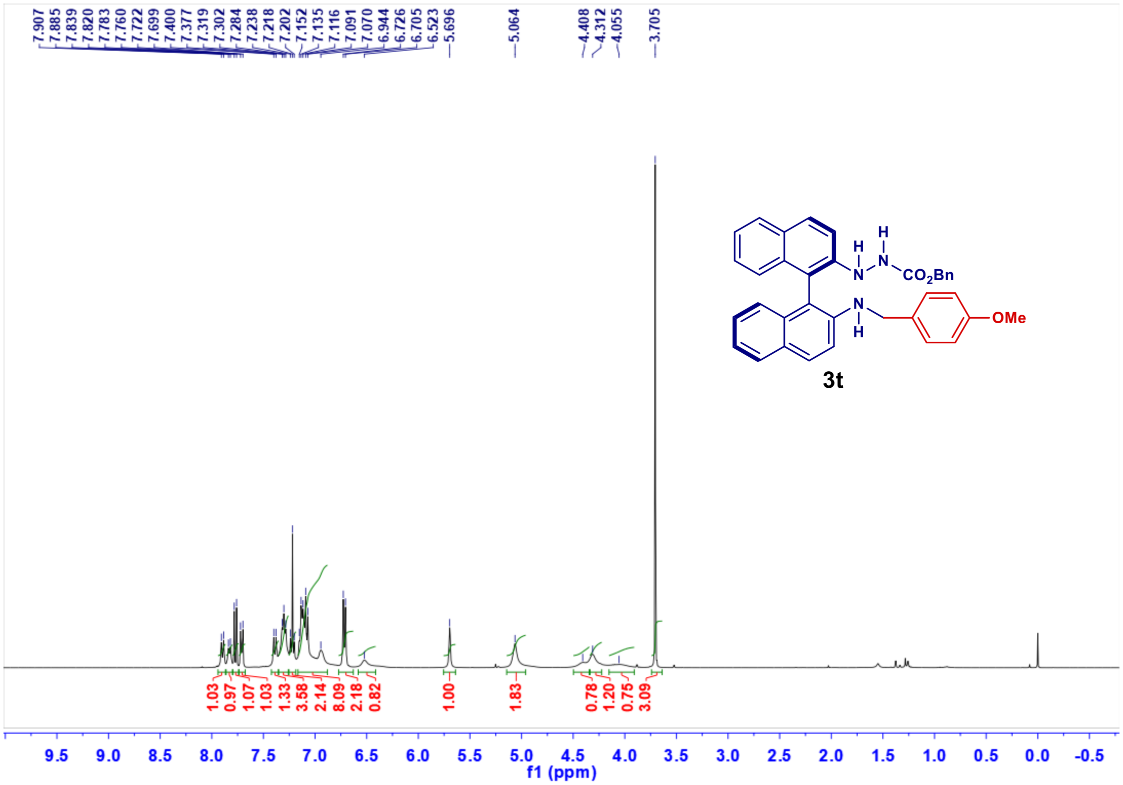

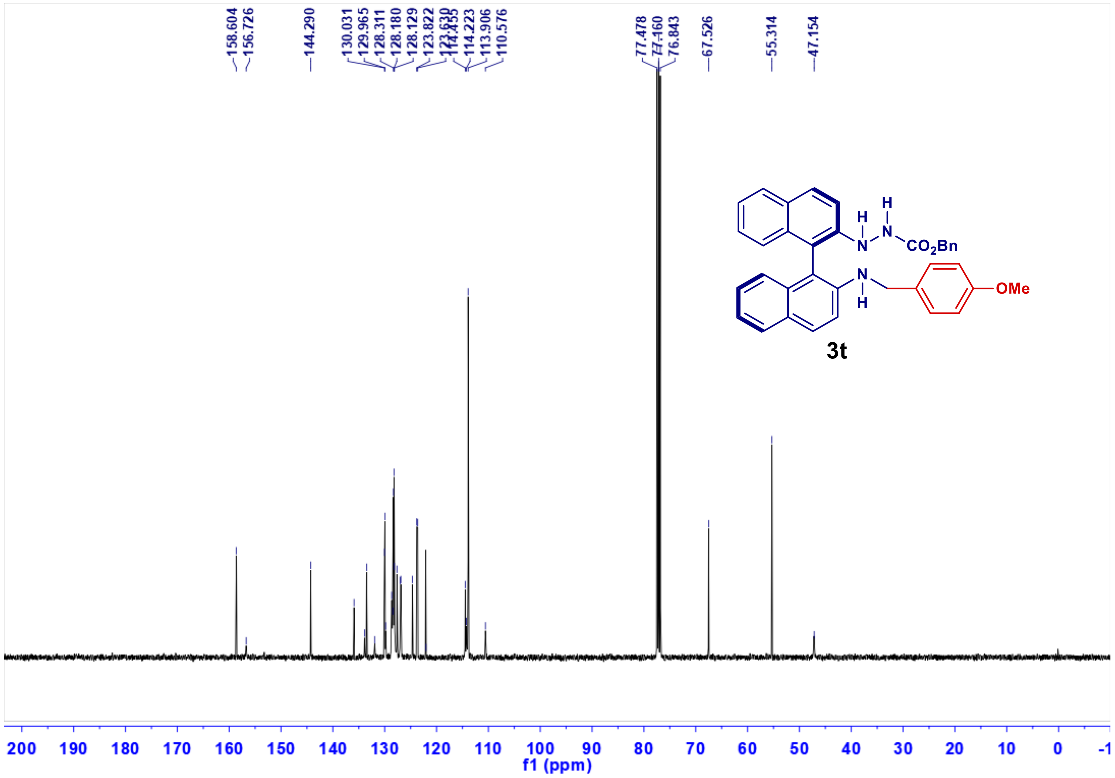

Supplementary Figure 66. ^{1}H and ^{13}C NMR spectra of 3t

Supplementary Figure 67. HPLC spectra of (*R*)-benzyl 2-(2'-((4-methoxybenzyl)amino)-[1,1'-binaphthalen]-2-yl)hydrazine-1-carboxylate (3t). Diacel Chiralpak AD-H, *n*-Hexane:*i*-PrOH = 85:15, flow = 1.0 mL/min, 25 °C, λ = 254 nm, t_R(major) = 18.9 min, t_R(minor) = 16.6 min, e.r. = 97:3

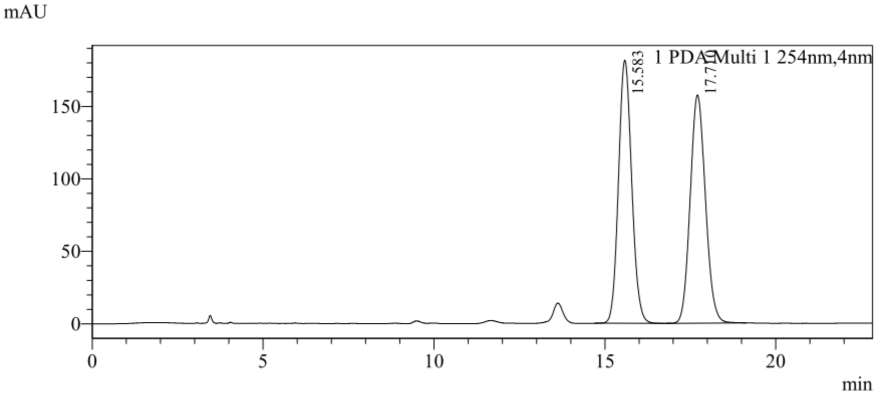

Peak Table

PDA Ch1 254nm

Peak#	Ret. Time	Peak End	Height	Area	Area%
1	15.583	16.832	181471	4879189	49.923
2	17.710	19.125	157382	4894306	50.077
Total			338853	9773495	100.000

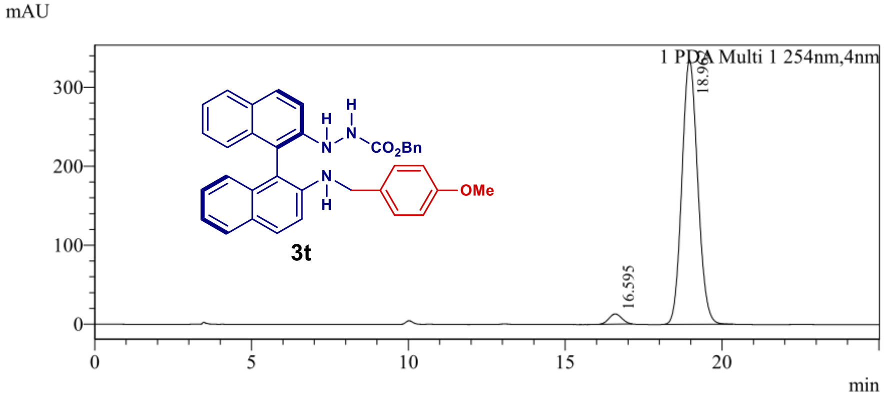

Peak Table

PDA Ch1 254nm

Peak#	Ret. Time	Peak End	Height	Area	Area%
1	16.595	17.120	12972	370398	3.051
2	18.962	20.331	334915	11768914	96.949
Total			347888	12139312	100.000

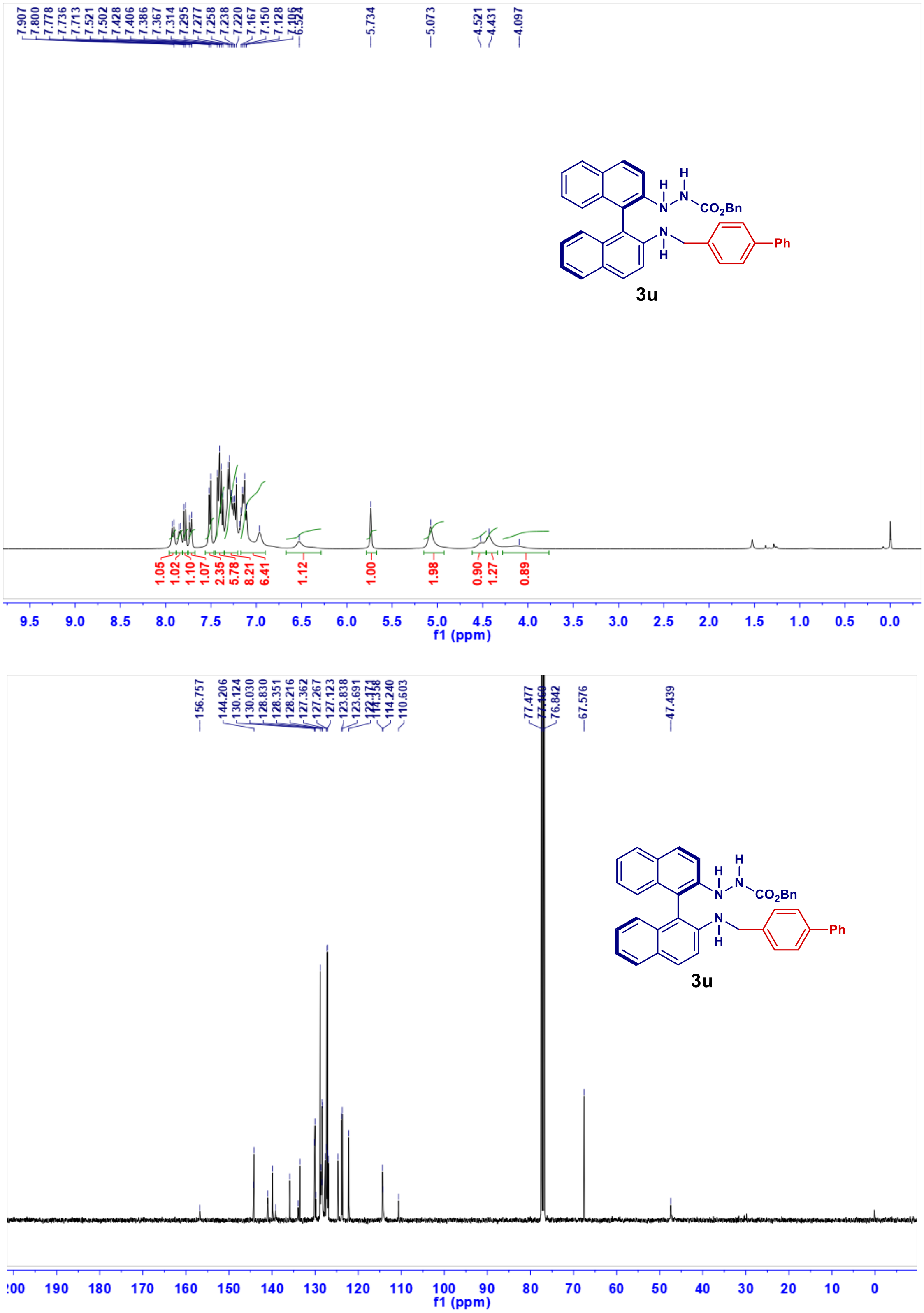

Supplementary Figure 68. ^{1}H and ^{13}C NMR spectra of 3u

Supplementary Figure 69. HPLC spectra of (*R*)-benzyl 2-(2'-(([1,1'-biphenyl]-4-ylmethyl)amino)-[1,1'-binaphthalen]-2-yl)hydrazine-1-carboxylate (3u). Diacel Chiralpak AD-H, *n*-Hexane:*i*-PrOH = 85:15, flow = 1.0 mL/min, 25 °C, λ = 254 nm, t_R(major) = 17.5 min, t_R(minor) = 16.1 min, e.r. = 97:3

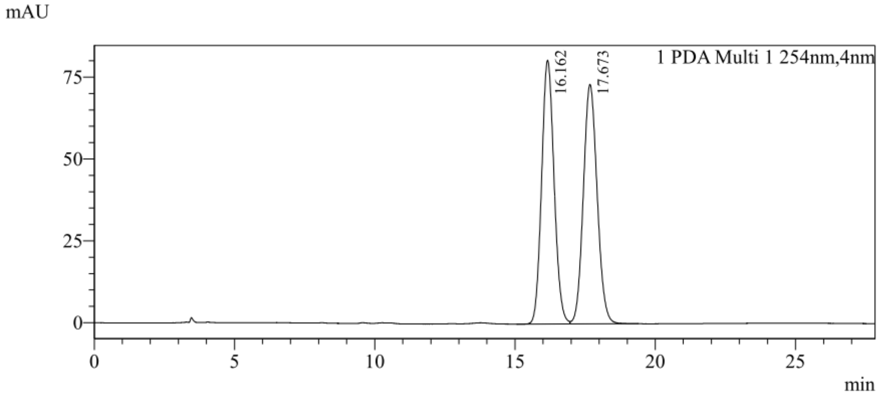

Peak Table

PDA Ch1 254nm

Peak#	Ret. Time	Peak End	Height	Area	Area%
1	16.162	16.960	80551	2490926	49.810
2	17.673	19.392	73184	2509977	50.190
Total			153735	5000903	100.000

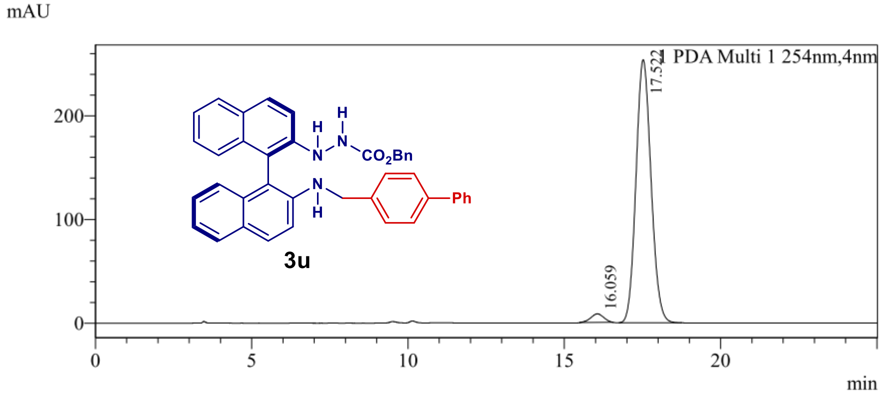

Peak Table

PDA Ch1 254nm

Peak#	Ret. Time	Peak End	Height	Area	Area%
1	16.059	16.565	8331	242942	2.775
2	17.522	18.752	253804	8512091	97.225
Total			262135	8755033	100.000

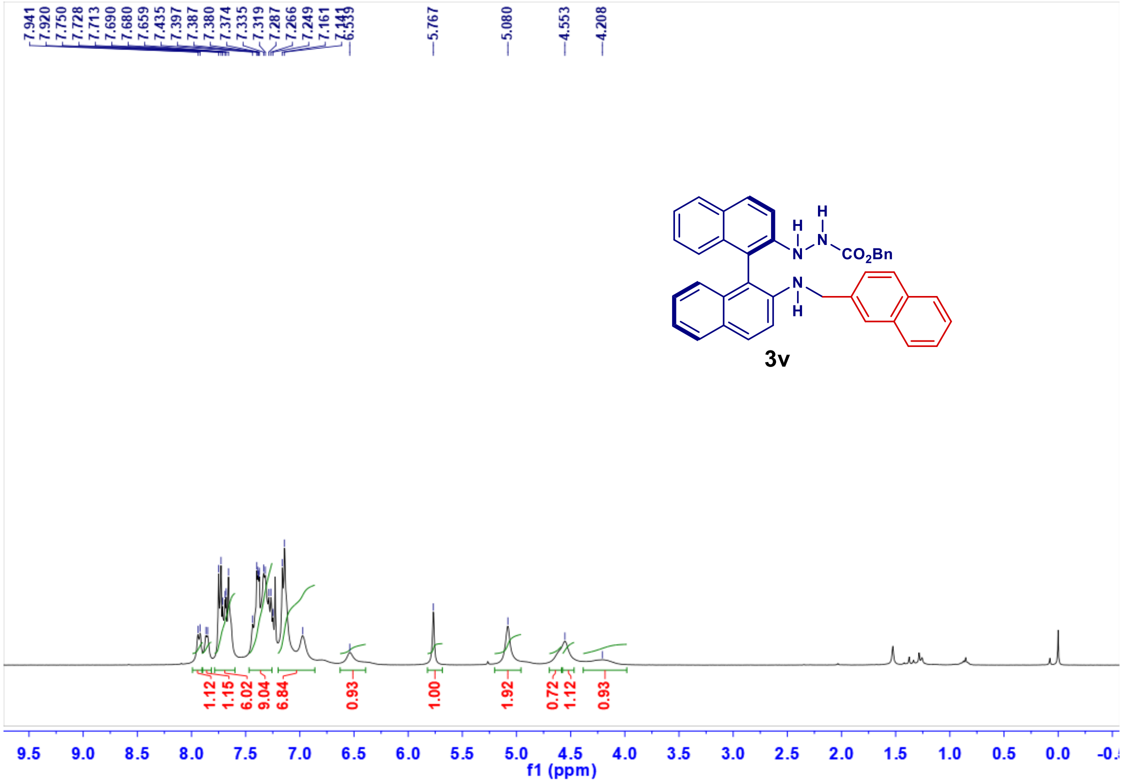

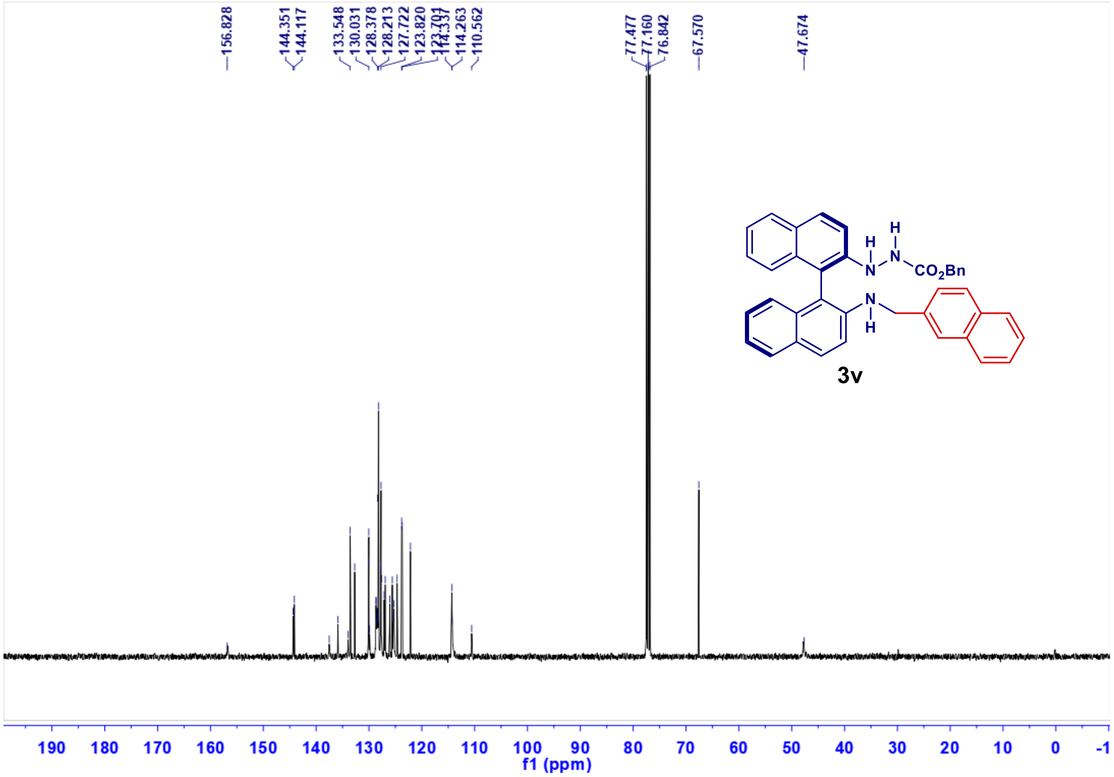

Supplementary Figure 70. ^{1}H and ^{13}C NMR spectra of 3v

Supplementary Figure 71. HPLC spectra of (*R*)-benzyl 2-(2’-((naphthalen-2-ylmethyl)amino)-[1,1’-binaphthalen]-2-yl)hydrazine-1-carboxylate (3v). Diacel Chiralpak AD-H, *n*-Hexane:*i*-PrOH = 85:15, flow = 1.0 mL/min, 25 °C, λ = 254 nm, t_R(major) = 17.2 min, t_R(minor) = 14.7 min, e.r. = 97:3

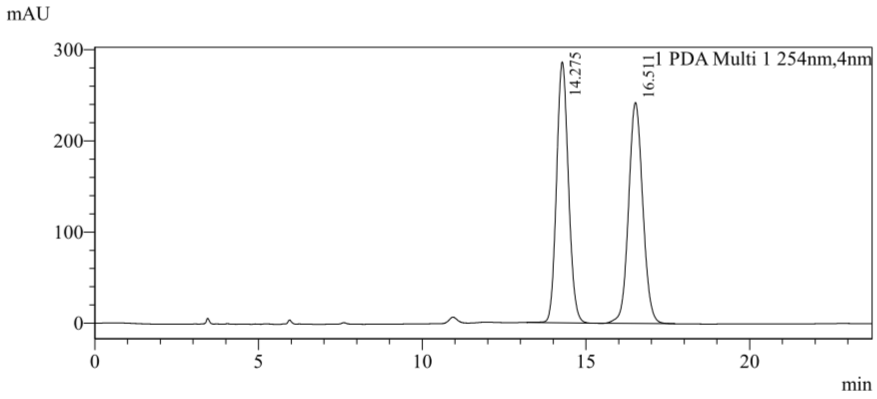

Peak Table

PDA Ch1 254nm

Peak#	Ret. Time	Peak End	Height	Area	Area%
1	14.275	15.285	286080	7210771	49.635
2	16.511	17.696	242480	7316787	50.365
Total			528560	14527557	100.000

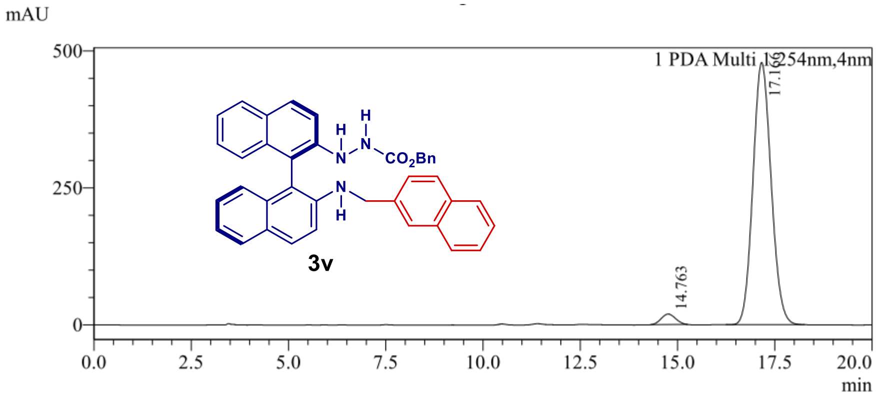

Peak Table

PDA Ch1 254nm

Peak#	Ret. Time	Peak End	Height	Area	Area%
1	14.763	15.253	19110	488454	3.042
2	17.166	18.261	478461	15569082	96.958
Total			497571	16057537	100.000

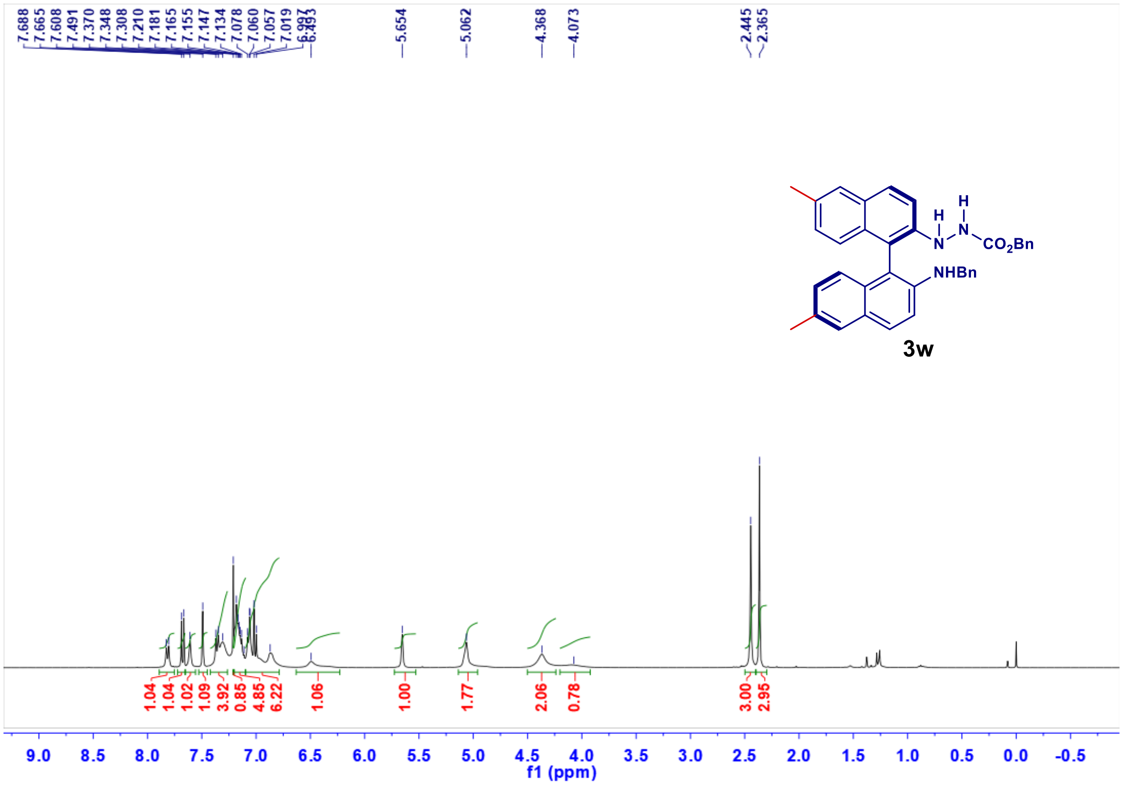

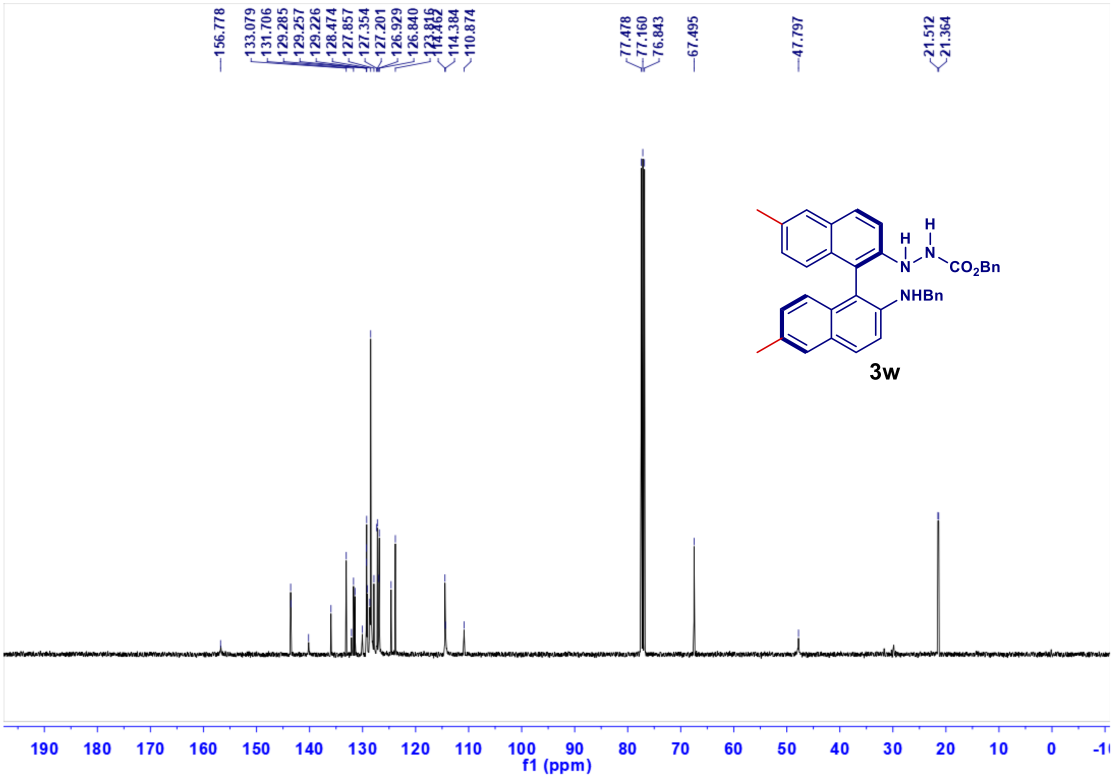

Supplementary Figure 72. ^{1}H and ^{13}C NMR spectra of 3w

Supplementary Figure 73. HPLC spectra of (*R*)-benzyl 2-(2'-(benzylamino)-6,6'-dimethyl-[1,1'-binaphthalen]-2-yl)hydrazine-1-carboxylate (3w). Diacel Chiralpak AD-H, *n*-Hexane:*i*-PrOH = 85:15, flow = 1.0 mL/min, 25 °C, λ = 254 nm, t_R(major) = 9.7 min, t_R(minor) = 10.9 min, e.r. = 96.5:3.5

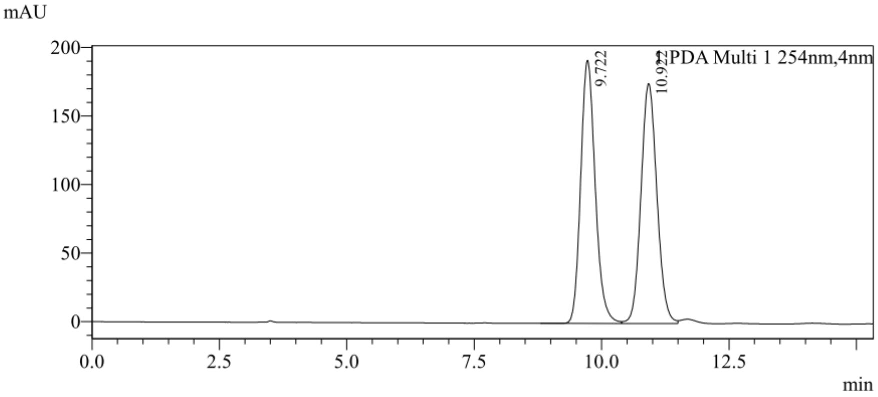

Peak Table

PDA Ch1 254nm

Peak#	Ret. Time	Peak End	Height	Area	Area%
1	9.722	10.389	191797	3806232	49.965
2	10.922	11.499	175063	3811537	50.035
Total			366860	7617769	100.000

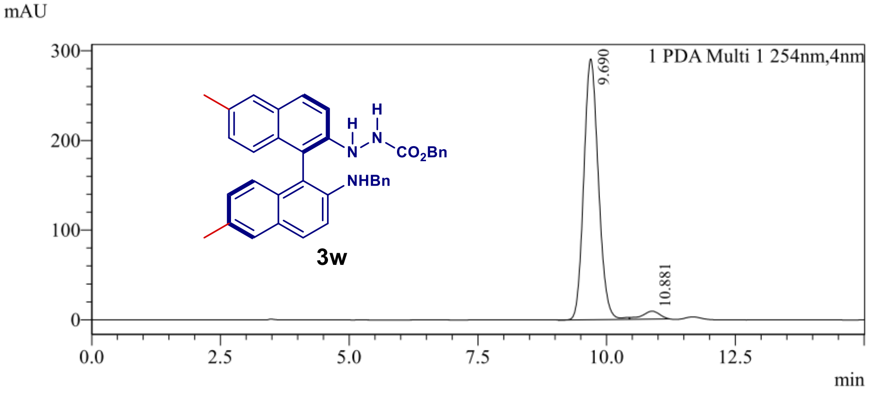

Peak Table

PDA Ch1 254nm

Peak#	Ret. Time	Peak End	Height	Area	Area%
1	9.690	10.443	290461	5714723	96.559
2	10.881	11.221	8743	203667	3.441
Total			299204	5918391	100.000

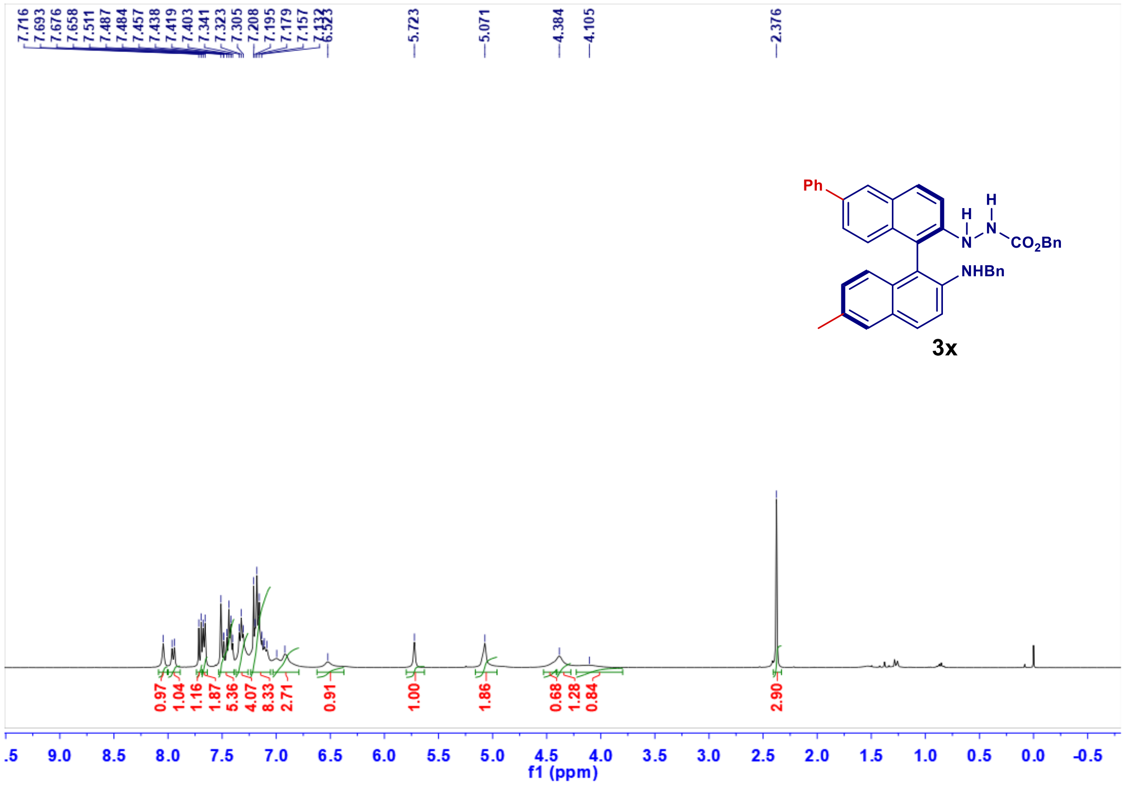

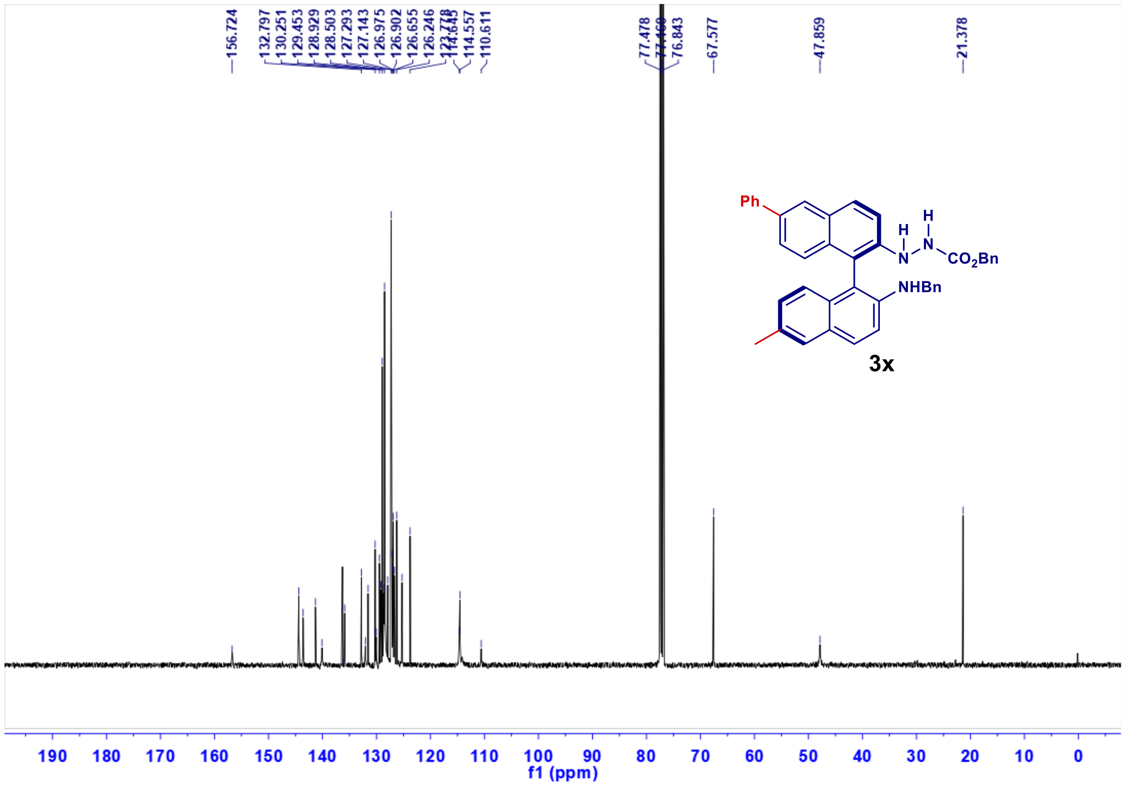

Supplementary Figure 74. ¹H and ¹³C NMR spectra of 3x

Supplementary Figure 75. HPLC spectra of (*R*)-benzyl 2-(2'-(benzylamino)-6'-methyl-6-phenyl-[1,1'-binaphthalen]-2-yl)hydrazine-1-carboxylate (3x). Diacel Chiralpak AD-H, *n*-Hexane:*i*-PrOH = 85:15, flow = 1.0 mL/min, 25 °C, λ = 254 nm, t_R(major) = 12.7 min, t_R(minor) = 15.6 min, e.r. = 96.5:3.5

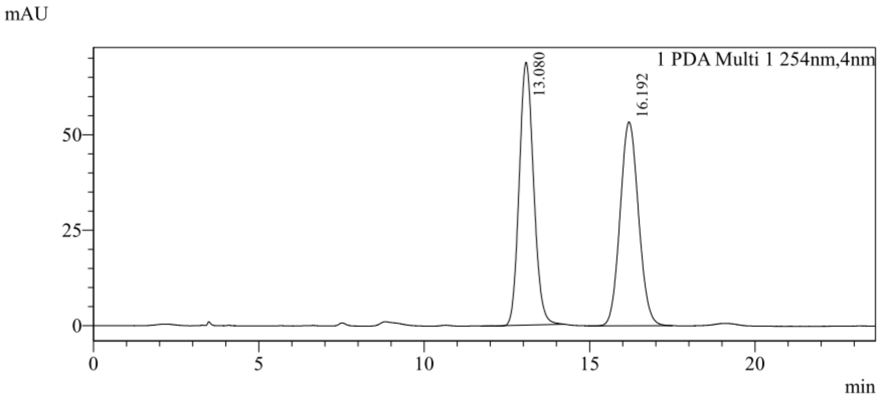

Peak Table

PDA Ch1 254nm

Peak#	Ret. Time	Peak End	Height	Area	Area%
1	13.080	14.251	68807	2080998	50.150
2	16.192	17.493	53386	2068547	49.850
Total			122192	4149545	100.000

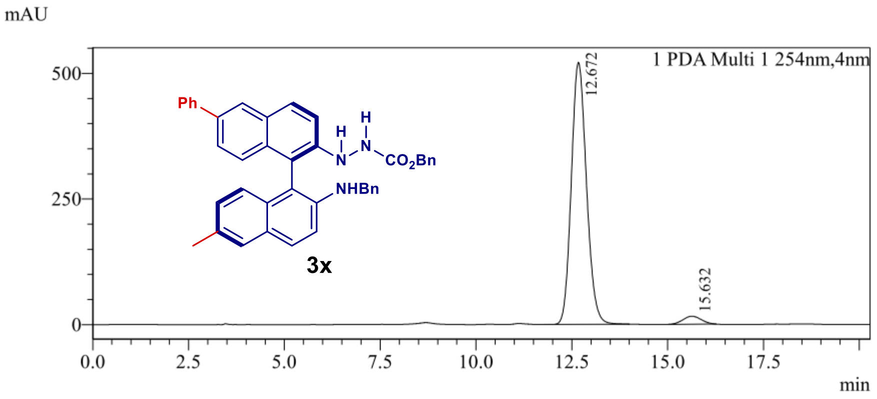

Peak Table

PDA Ch1 254nm

Peak#	Ret. Time	Peak End	Height	Area	Area%
1	12.672	13.995	521482	14364279	96.426
2	15.632	16.256	15968	532452	3.574
Total			537450	14896731	100.000

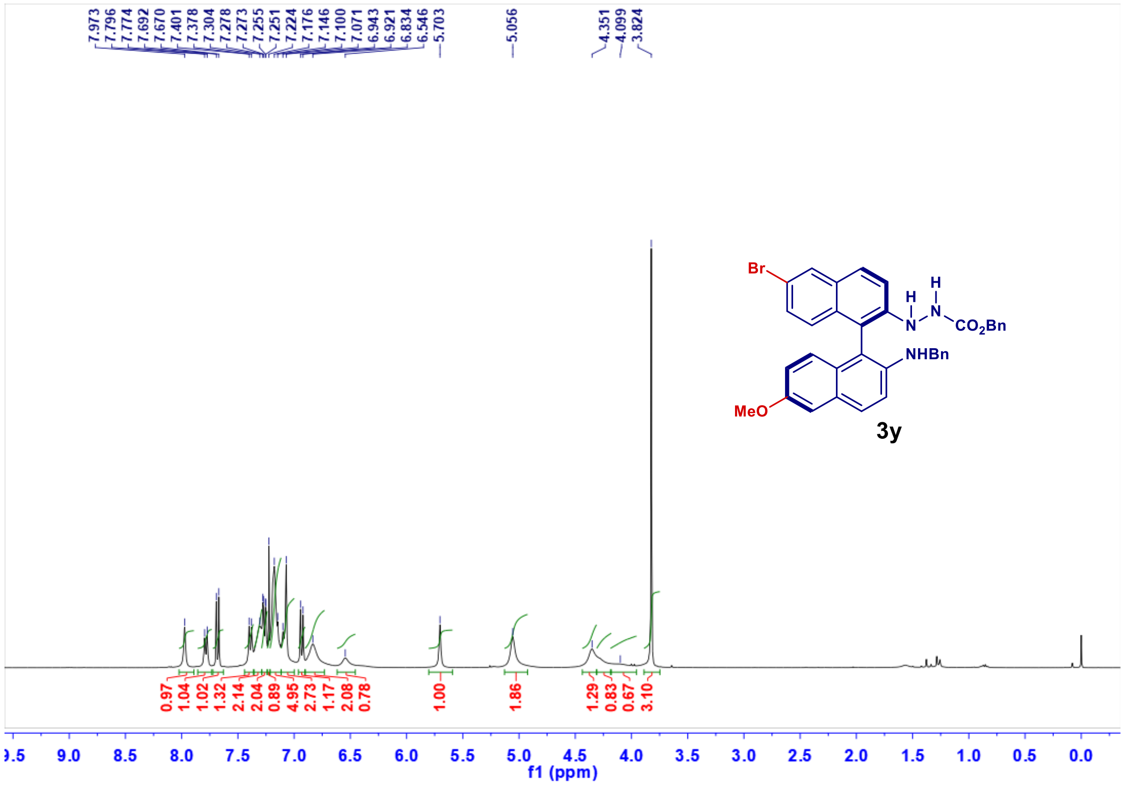

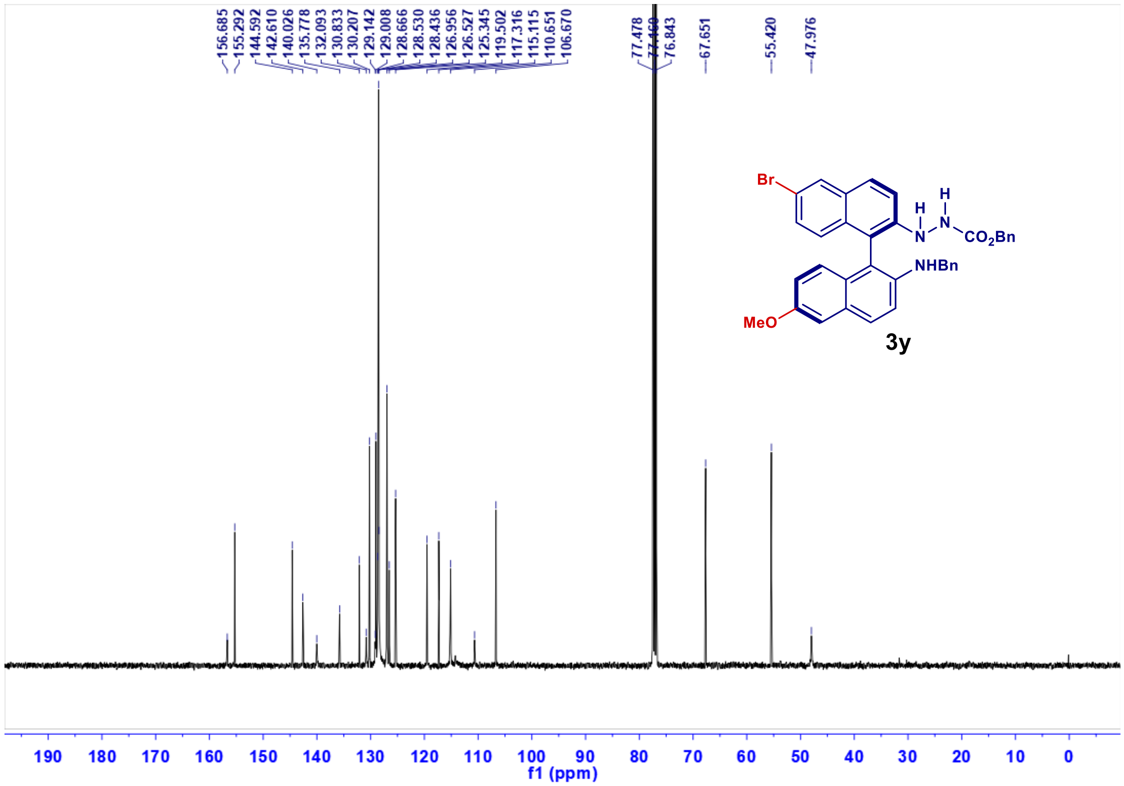

Supplementary Figure 76. ^{1}H and ^{13}C NMR spectra of 3y

Supplementary Figure 77. HPLC spectra of (*R*)-benzyl 2-(2'-(benzylamino)-6-bromo-6'-methoxy-[1,1'-binaphthalen]-2-yl)hydrazine-1-carboxylate (3y). Diacel Chiralcel OD-H, *n*-Hexane:*i*-PrOH = 80:20, flow = 1.0 mL/min, 25 °C, λ = 254 nm, t_R(major) = 15.8 min, t_R(minor) = 26.9 min, e.r. = 97.5:2.5

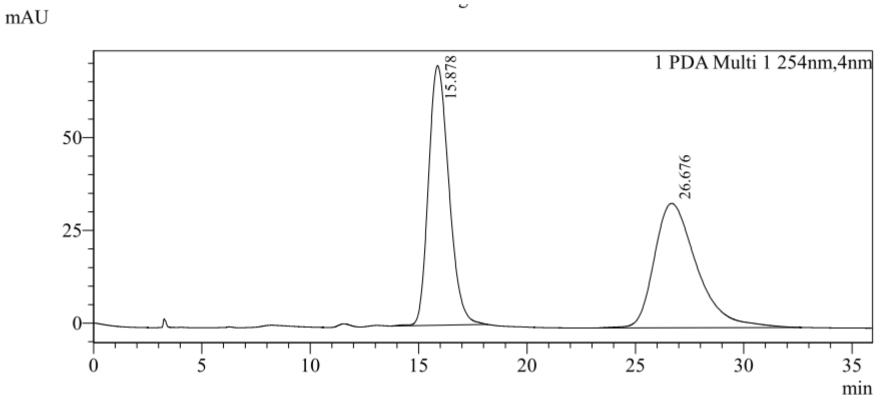

Peak Table

PDA Ch1 254nm

Peak#	Ret. Time	Peak End	Height	Area	Area%
1	15.878	18.197	69977	4589467	50.077
2	26.676	32.672	33517	4575345	49.923
Total			103494	9164812	100.000

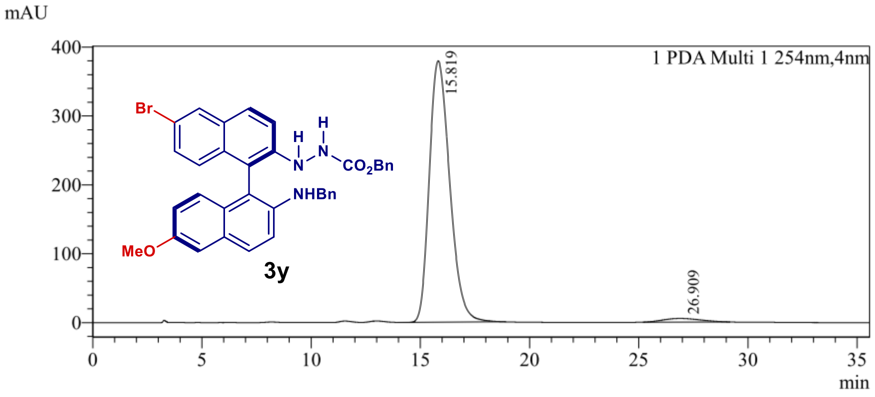

Peak Table

PDA Ch1 254nm

Peak#	Ret. Time	Peak End	Height	Area	Area%
1	15.819	18.869	379421	24715163	97.529
2	26.909	29.163	5263	626293	2.471
Total			384684	25341457	100.000

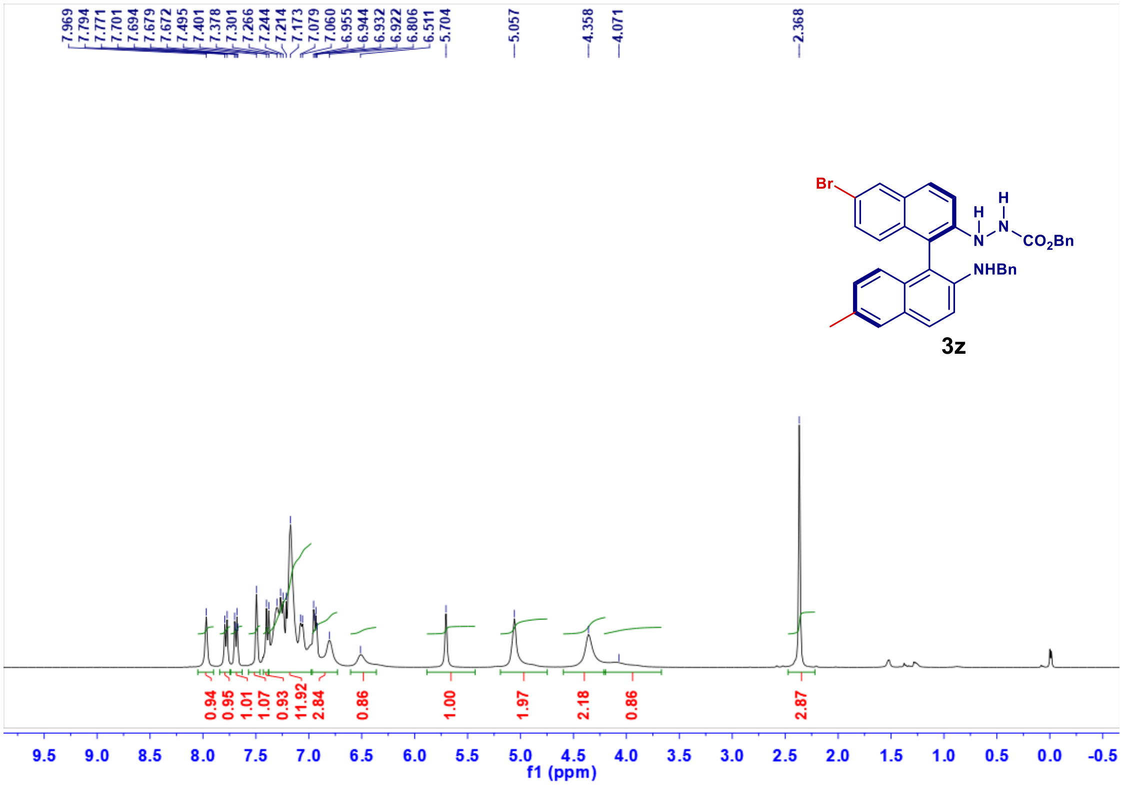

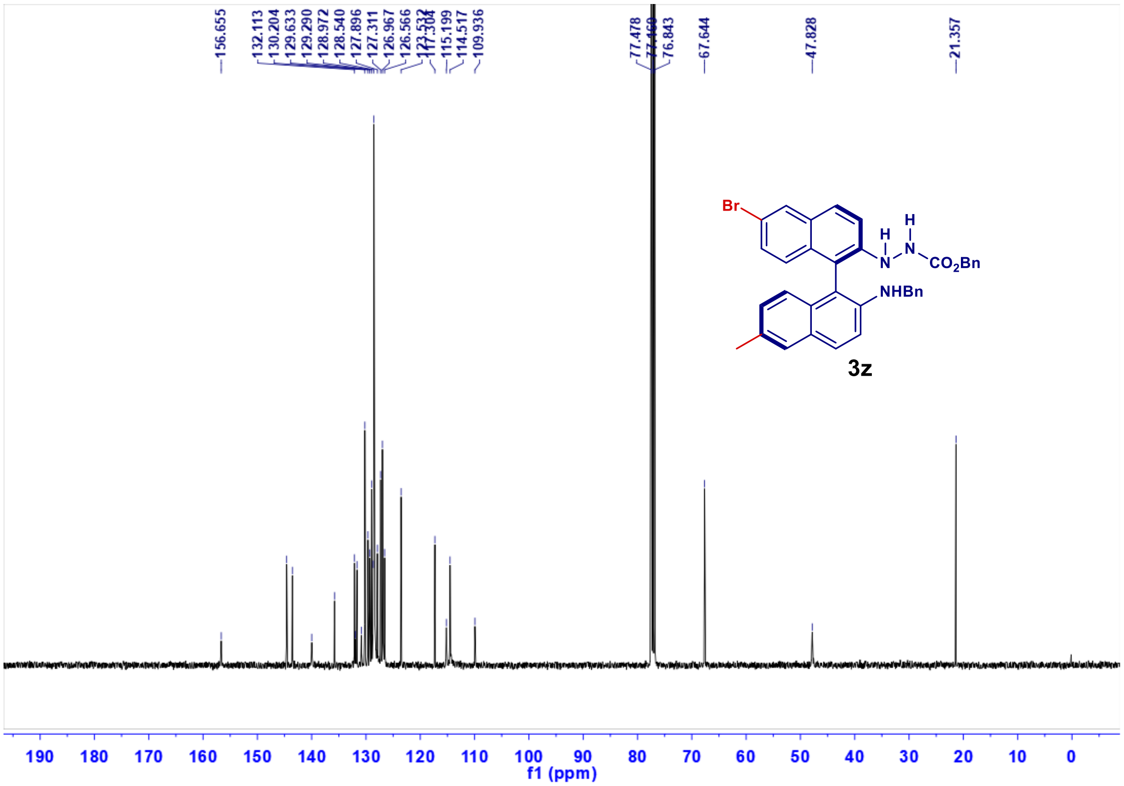

Supplementary Figure 78. ^{1}H and ^{13}C NMR spectra of 3z

Supplementary Figure 79. HPLC spectra of (*R*)-benzyl 2-(2'-(benzylamino)-6-bromo-6'-methyl-[1,1'-binaphthalen]-2-yl)hydrazine-1-carboxylate (3z). Diacel Chiralcel OD-H, *n*-Hexane:*i*-PrOH = 80:20, flow = 1.0 mL/min, 25 °C, λ = 254 nm, t_R(major) = 11.4 min, t_R(minor) = 16.2 min, e.r. = 97.5:2.5

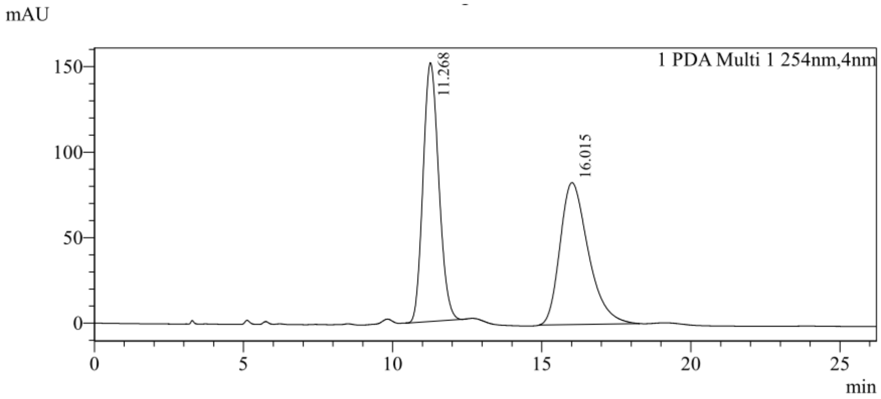

Peak Table

PDA Ch1 254nm

Peak#	Ret. Time	Peak End	Height	Area	Area%
1	11.268	12.459	151238	5546264	50.319
2	16.015	18.272	82982	5476039	49.681
Total			234221	11022304	100.000

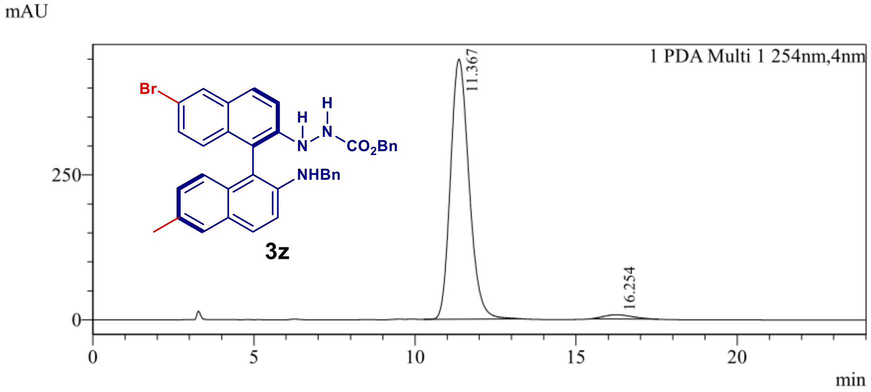

Peak Table

PDA Ch1 254nm

Peak#	Ret. Time	Peak End	Height	Area	Area%
1	11.367	13.355	448522	17963683	97.449
2	16.254	17.547	7401	470219	2.551
Total			455924	18433902	100.000

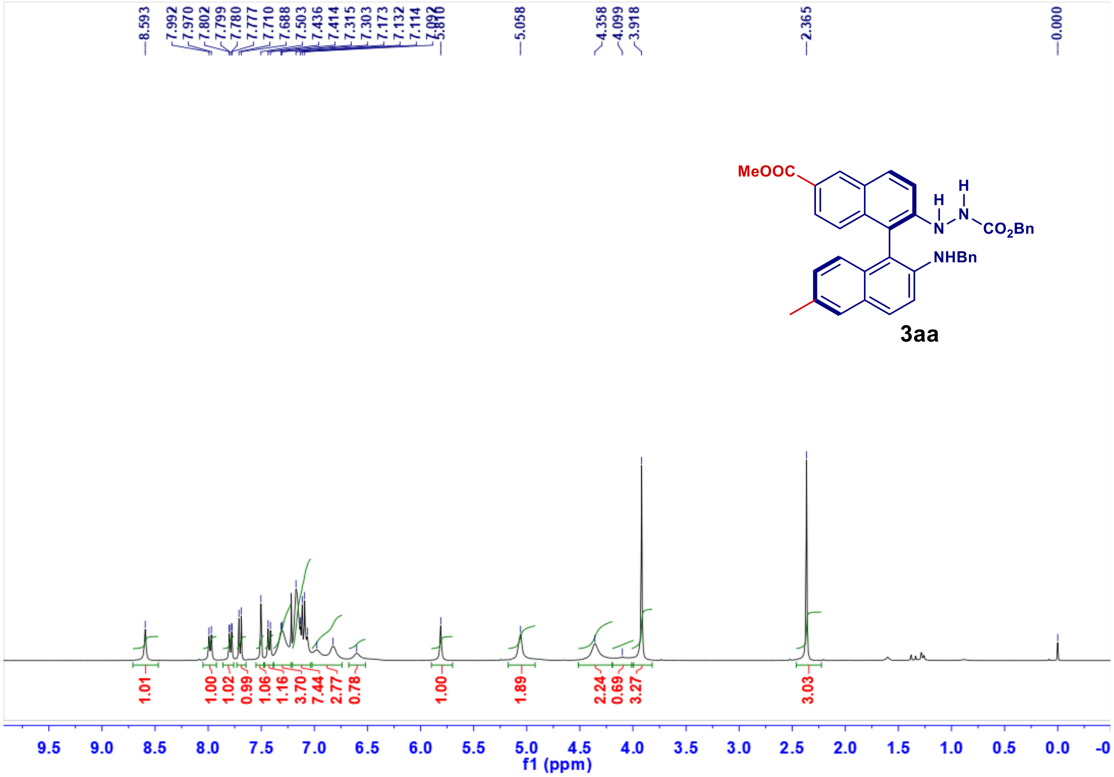

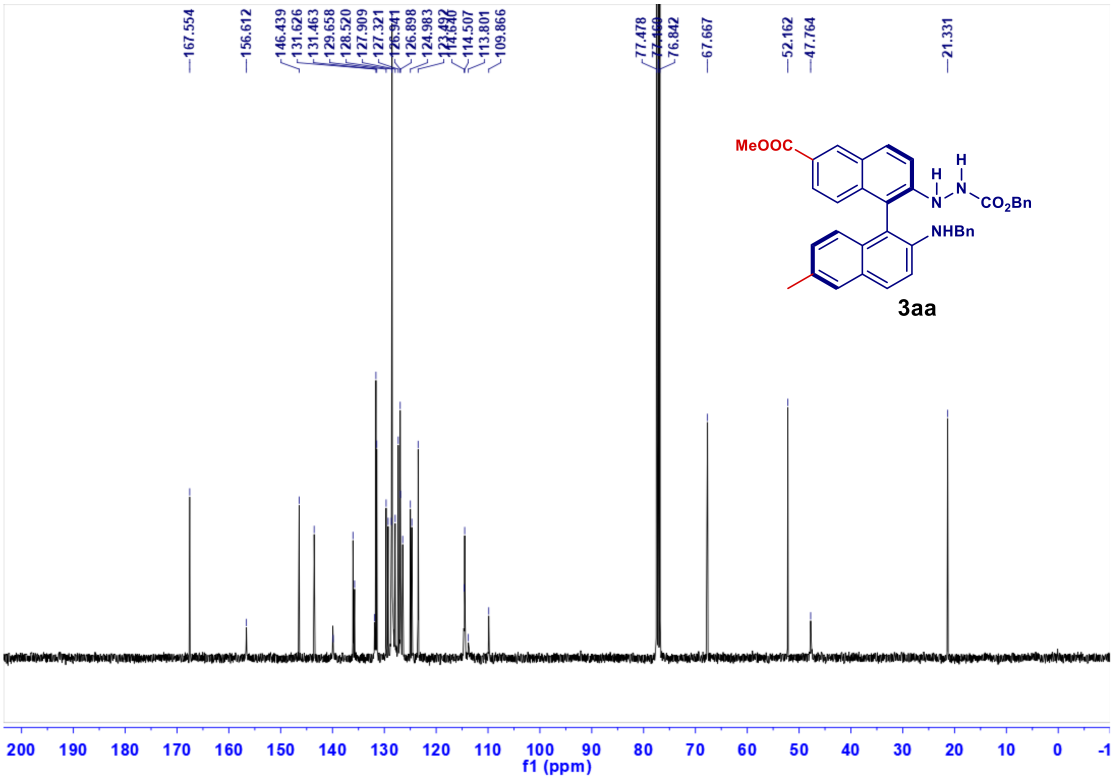

Supplementary Figure 80. ^{1}H and ^{13}C NMR spectra of 3aa

Supplementary Figure 81. HPLC spectra of (*R*)-benzyl 2-(2'-(benzylamino)-6-(methoxycarbonyl)-6'-methyl-[1,1'-binaphthalen]-2-yl)hydrazine-1-carboxylate (3aa). Diacel Chiralpak AD-H, *n*-Hexane:*i*-PrOH = 75:25, flow = 1.0 mL/min, 25 °C, λ = 254 nm, t_R(major) = 9.5 min, t_R(minor) = 14.7 min, e.r. = 96:4

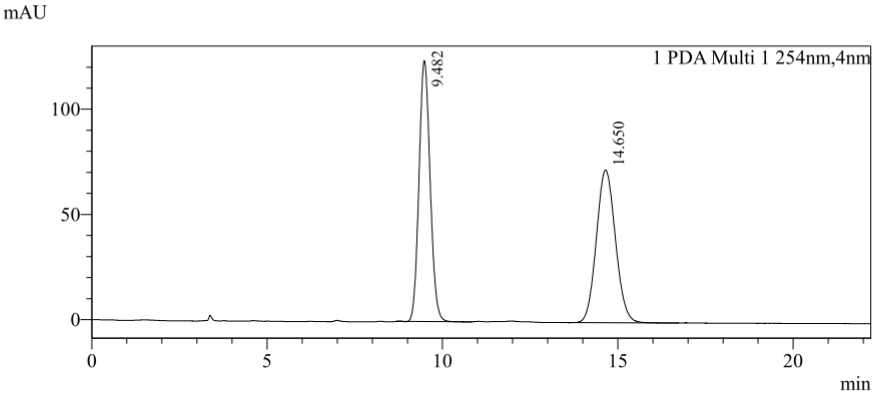

Peak Table

PDA Ch1 254nm

Peak#	Ret. Time	Peak End	Height	Area	Area%
1	9.482	10.837	123926	2730094	49.863
2	14.650	16.725	72586	2745088	50.137
Total			196512	5475182	100.000

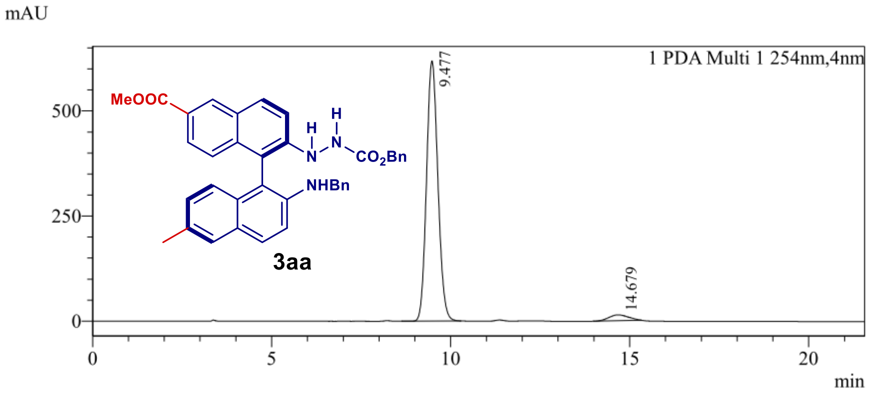

Peak Table

PDA Ch1 254nm

Peak#	Ret. Time	Peak End	Height	Area	Area%
1	9.477	10.283	618462	13624804	96.258
2	14.679	15.317	13916	529713	3.742
Total			632379	14154517	100.000

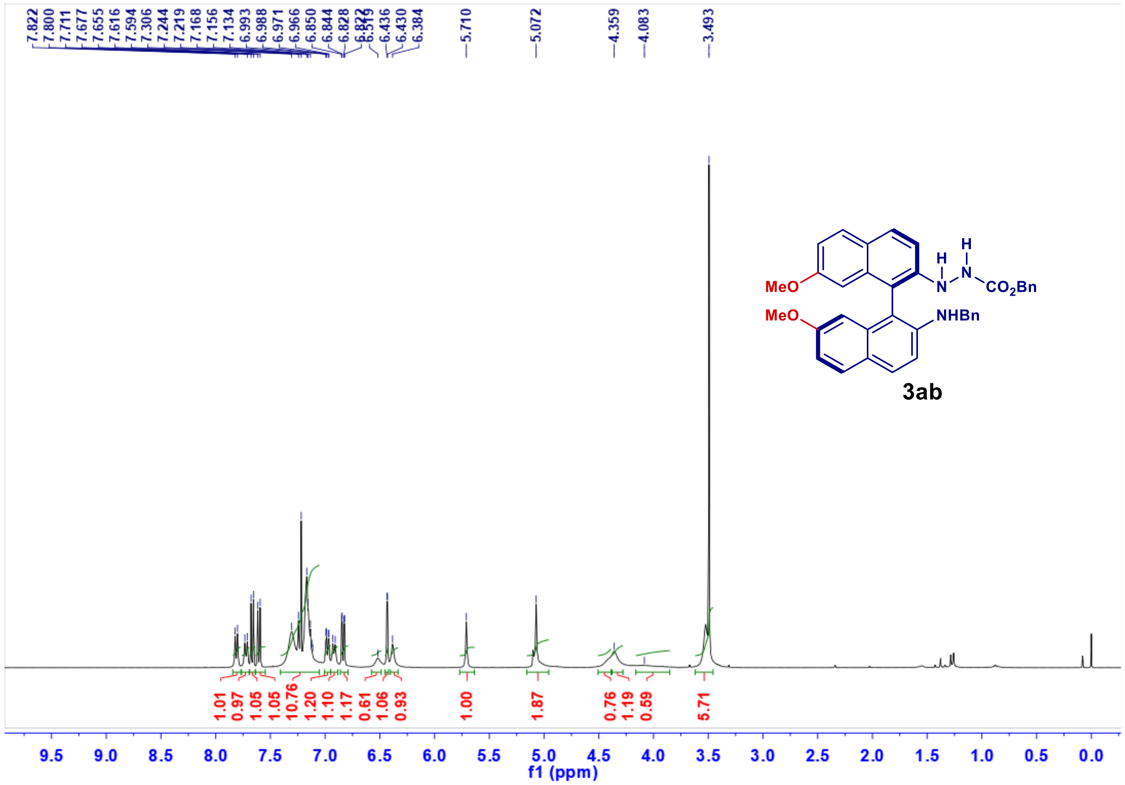

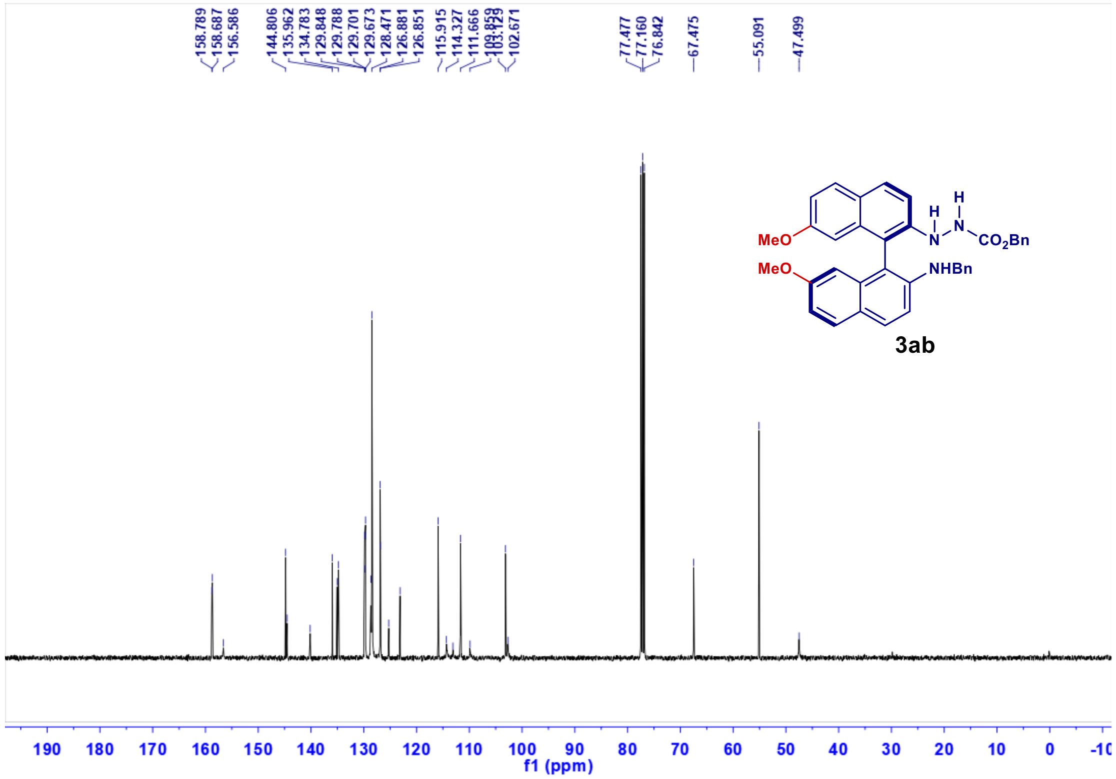

Supplementary Figure 82. ^{1}H and ^{13}C NMR spectra of 3ab

Supplementary Figure 83. HPLC spectra of (*R*)-benzyl 2-(2’-(benzylamino)-7,7’-dimethoxy-[1,1’-binaphthalen]-2-yl)hydrazine-1-carboxylate (3ab). Diacel Chiralpak AD-H, *n*-Hexane:*i*-PrOH = 85:15, flow = 1.0 mL/min, 25 °C, λ = 254 nm, t_R(major) = 13.0 min, t_R(minor) = 16.2 min, e.r. = 96.5:3.5

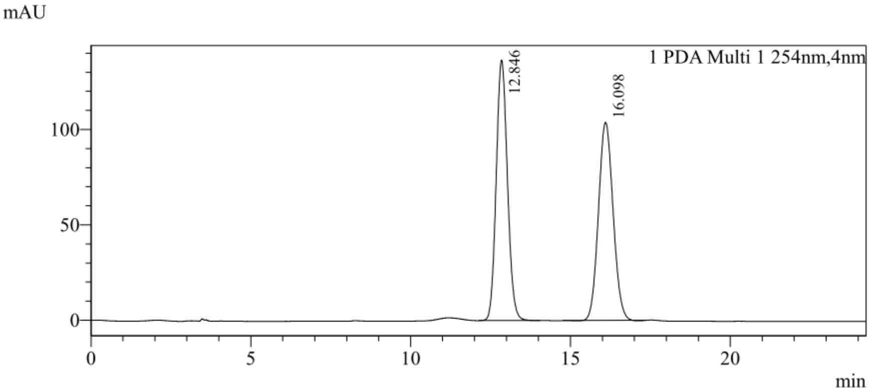

Peak Table

PDA Ch1 254nm

Peak#	Ret. Time	Peak End	Height	Area	Area%
1	12.846	14.037	136477	3333613	50.184
2	16.098	17.355	103865	3309206	49.816
Total			240342	6642819	100.000

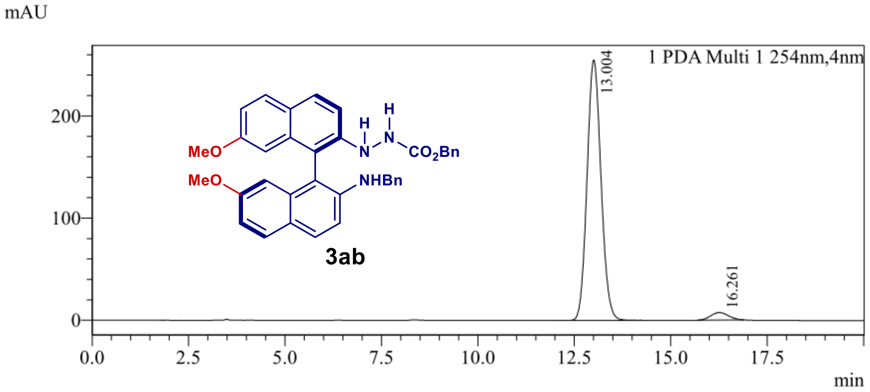

Peak Table

PDA Ch1 254nm

Peak#	Ret. Time	Peak End	Height	Area	Area%
1	13.004	14.261	254924	6419323	96.437
2	16.261	16.885	7345	237177	3.563
Total			262269	6656500	100.000

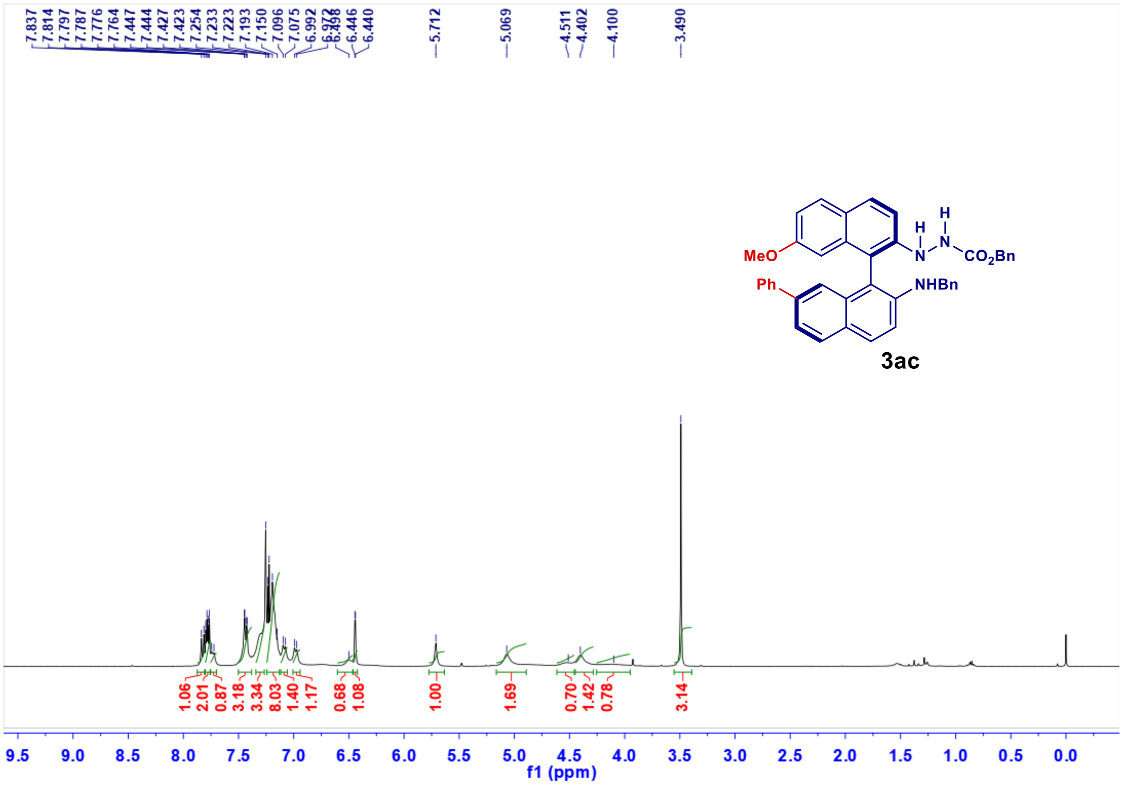

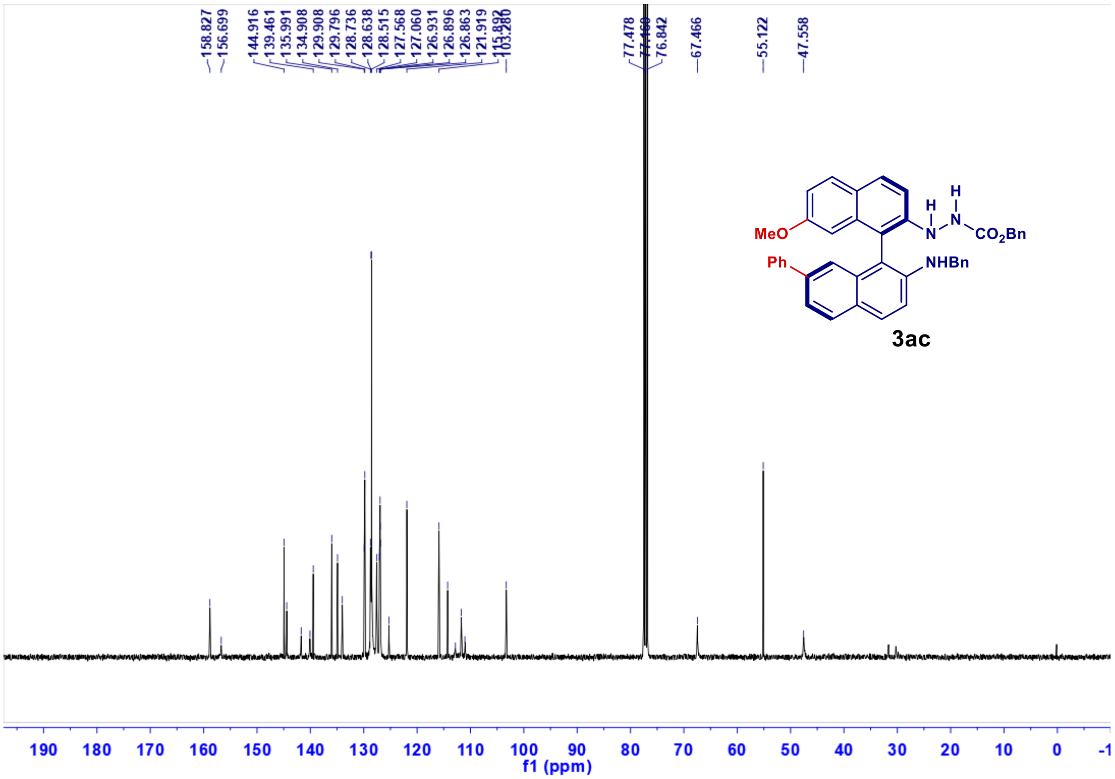

Supplementary Figure 84. ^{1}H and ^{13}C NMR spectra of 3ac

Supplementary Figure 85. HPLC spectra of (*R*)-benzyl 2-(2'-(benzylamino)-7-methoxy-7'-phenyl-[1,1'-binaphthalen]-2-yl)hydrazine-1-carboxylate (3ac). Diacel Chiralpak IC, *n*-Hexane:*i*-PrOH = 80:20, flow = 1.0 mL/min, 25 °C, λ = 254 nm, t_R(major) = 7.3 min, t_R(minor) = 8.7 min, e.r. = 98:2

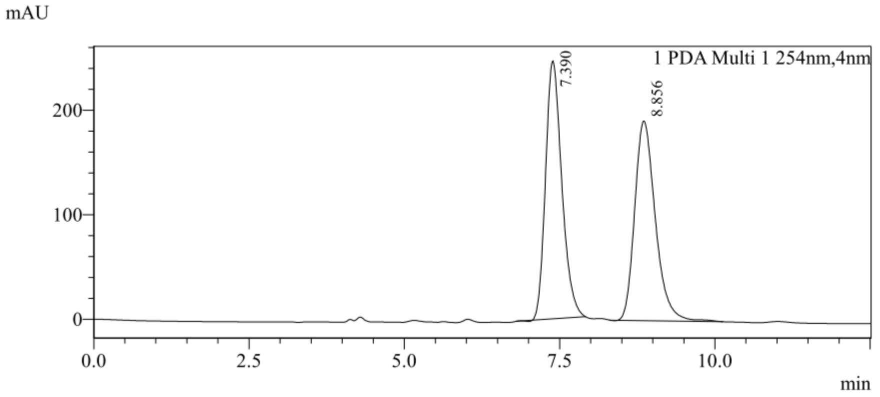

Peak Table

PDA Ch1 254nm

Peak#	Ret. Time	Peak End	Height	Area	Area%
1	7.390	7.915	246645	4495450	50.405
2	8.856	10.123	191018	4423295	49.595
Total			437663	8918745	100.000

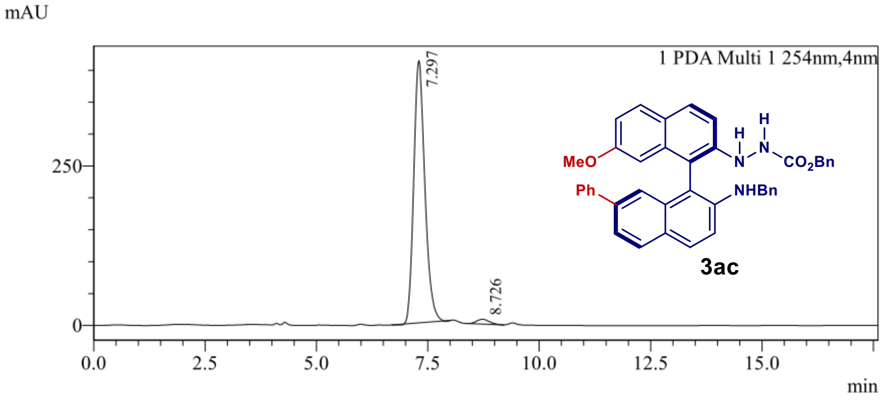

Peak Table

PDA Ch1 254nm

Peak#	Ret. Time	Peak End	Height	Area	Area%
1	7.297	8.032	410442	7136456	97.959
2	8.726	9.205	7518	148672	2.041
Total			417959	7285128	100.000

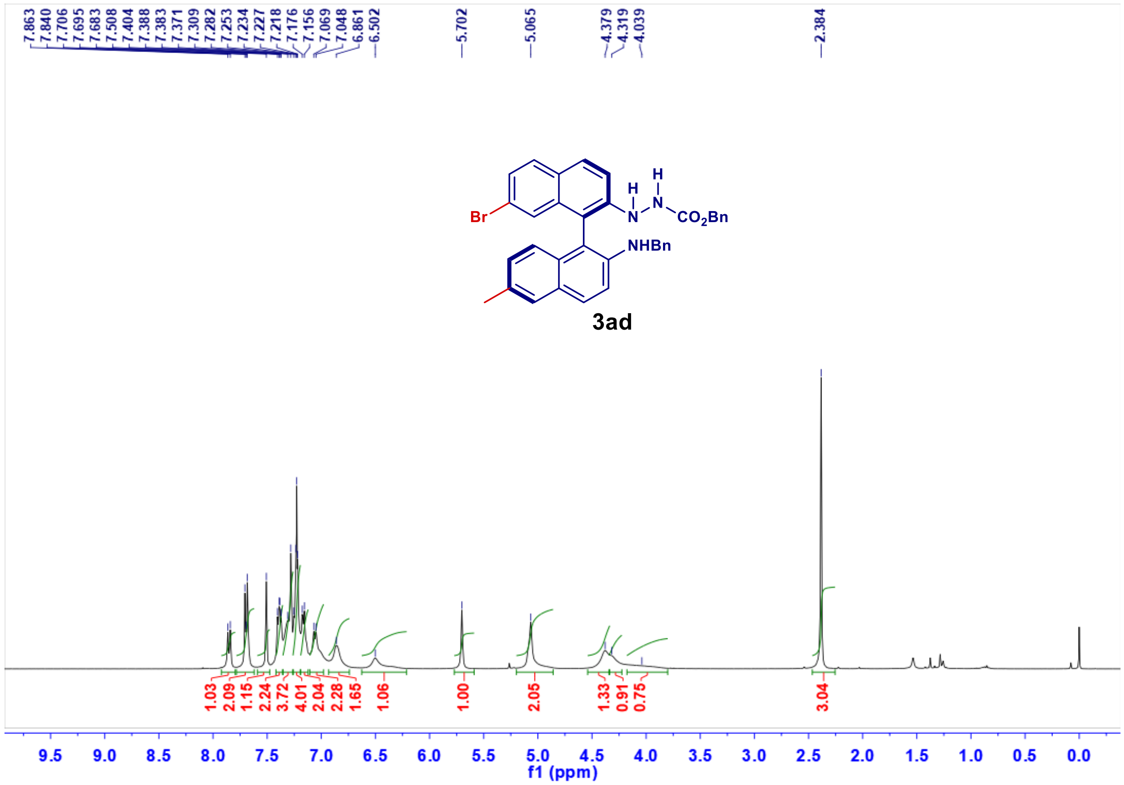

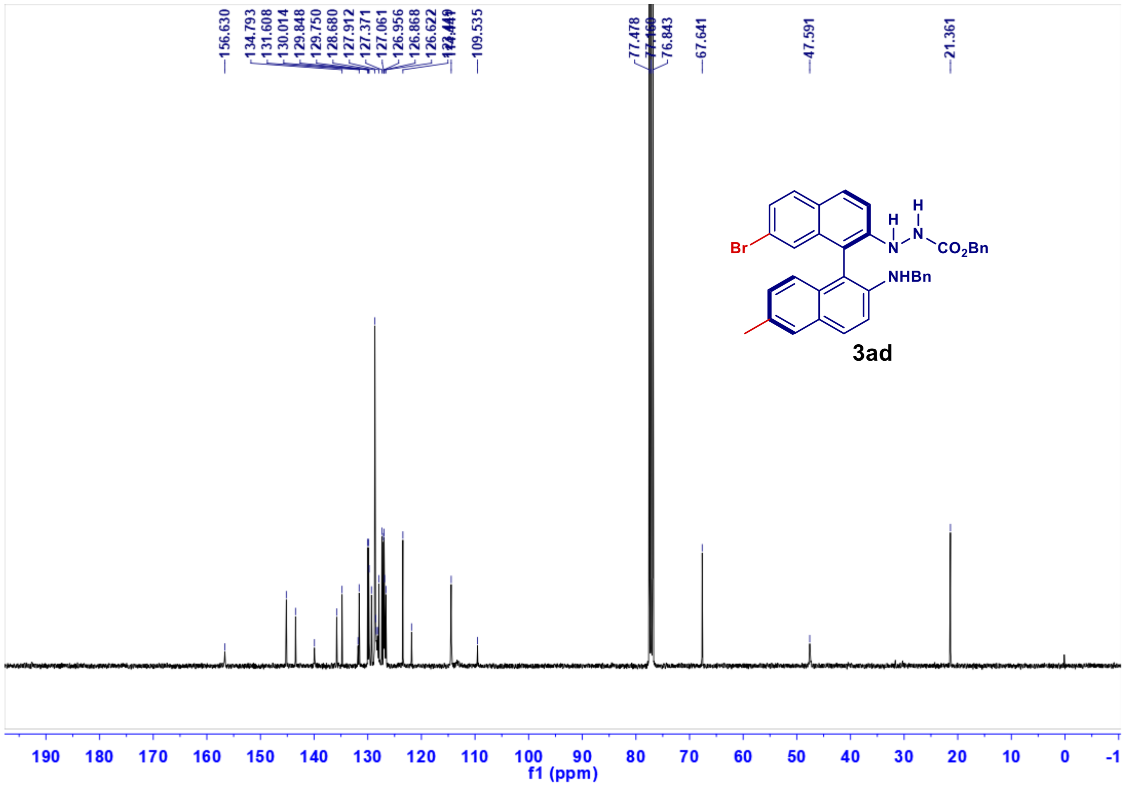

Supplementary Figure 86. ^{1}H and ^{13}C NMR spectra of 3ad

Supplementary Figure 87. HPLC spectra of (*R*)-benzyl 2-(2'-(benzylamino)-7,7'-dimethoxy-[1,1'-binaphthalen]-2-yl)hydrazine-1-carboxylate (3ad). Diacel Chiralpak AD-H, *n*-Hexane:*i*-PrOH = 85:15, flow = 1.0 mL/min, 25 °C, λ = 254 nm, t_R(major) = 8.7 min, t_R(minor) = 12.8 min, e.r. = 96.5:3.5

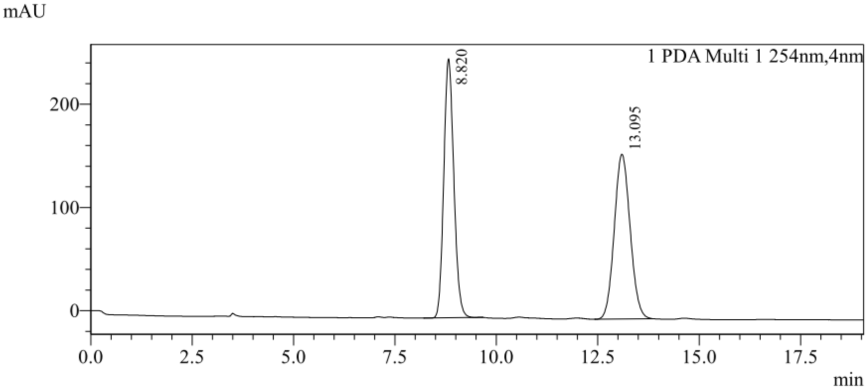

Peak Table

PDA Ch1 254nm

Peak#	Ret. Time	Peak End	Height	Area	Area%
1	8.820	9.664	250661	4330573	49.920
2	13.095	13.845	159702	4344449	50.080
Total			410364	8675022	100.000

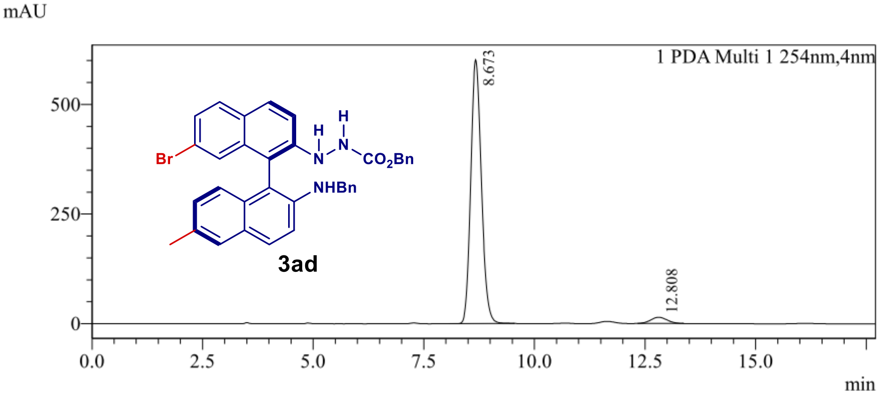

Peak Table

PDA Ch1 254nm

Peak#	Ret. Time	Peak End	Height	Area	Area%
1	8.673	9.547	601566	10066850	96.497
2	12.808	13.365	14159	365469	3.503
Total			615725	10432319	100.000

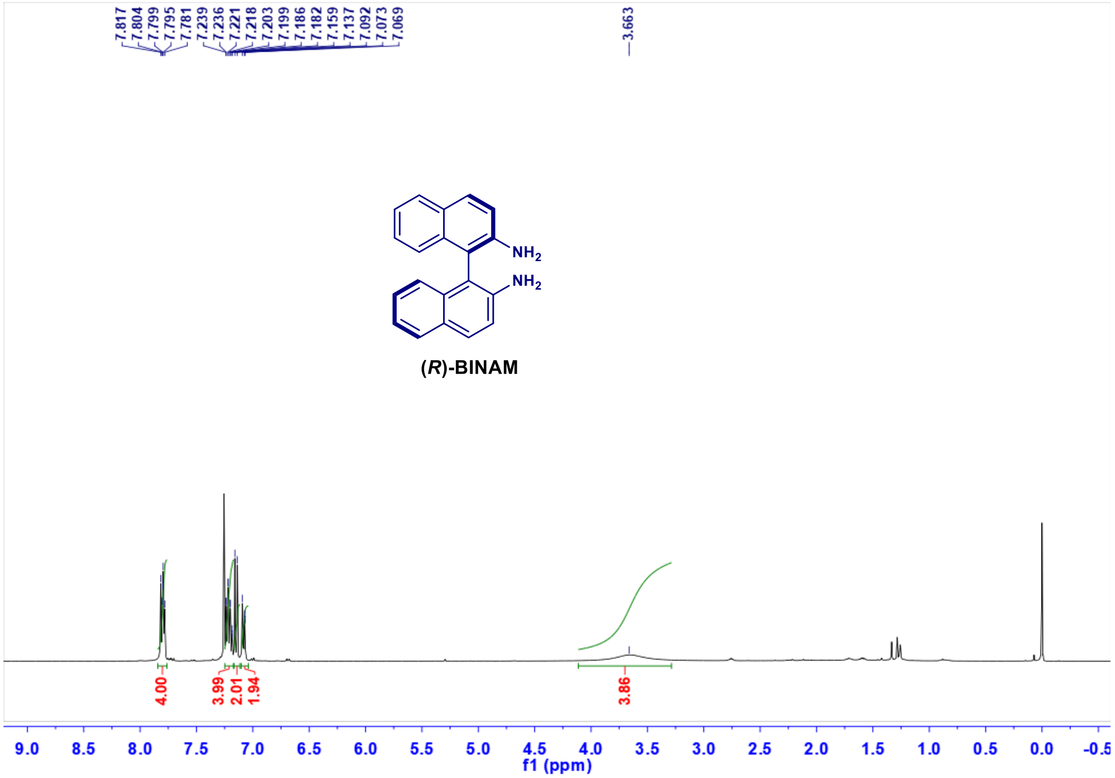

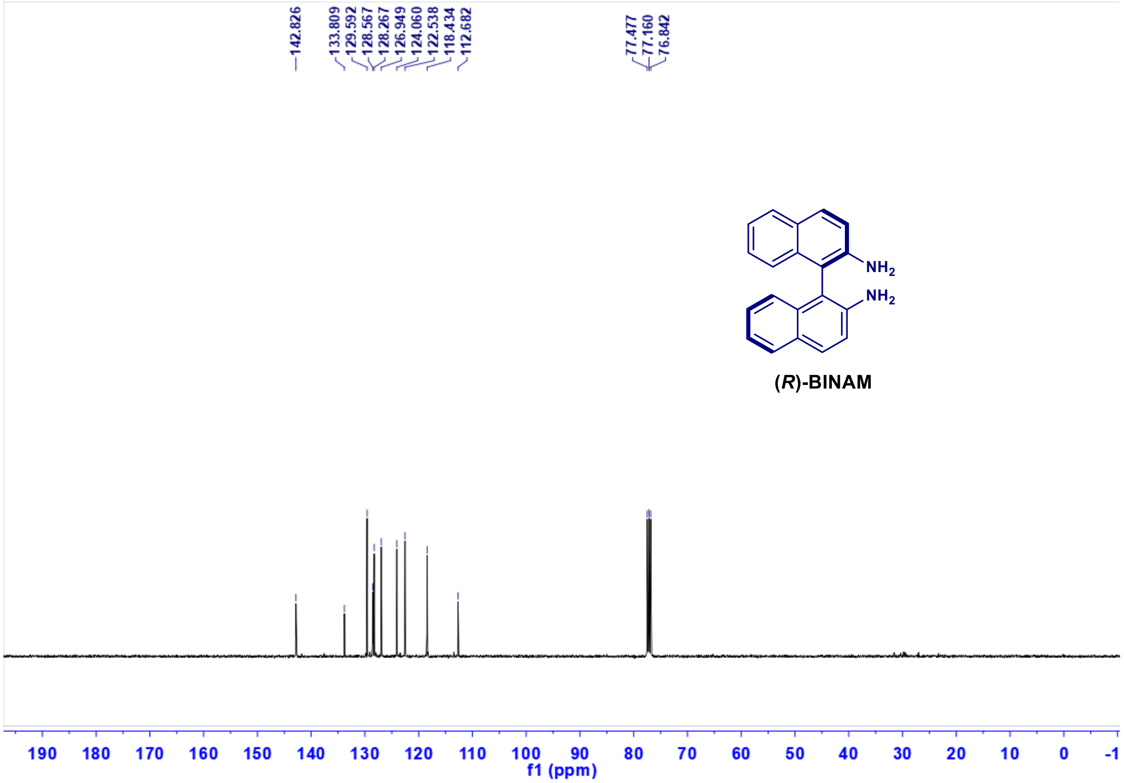

Supplementary Figure 88. ^{1}H and ^{13}C NMR spectra of (*R*)-BINAM

Supplementary Figure 89. HPLC spectra of (*R*)-BINAM. Diacel Chiralpak AD-H, *n*-Hexane:*i*-PrOH = 70:30, flow = 1.0 mL/min, 25 °C, λ = 254 nm, t_R(major) = 9.1 min, t_R(minor) = 28.4 min, e.r. = 97:3

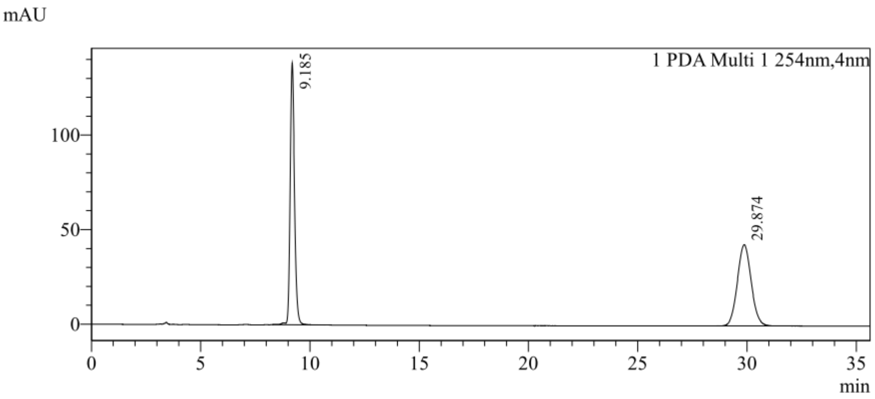

Peak Table

PDA Ch1 254nm

Peak#	Ret. Time	Peak End	Height	Area	Area%
1	9.185	10.677	138454	1908489	50.149
2	29.874	32.320	43030	1897181	49.851
Total			181484	3805669	100.000

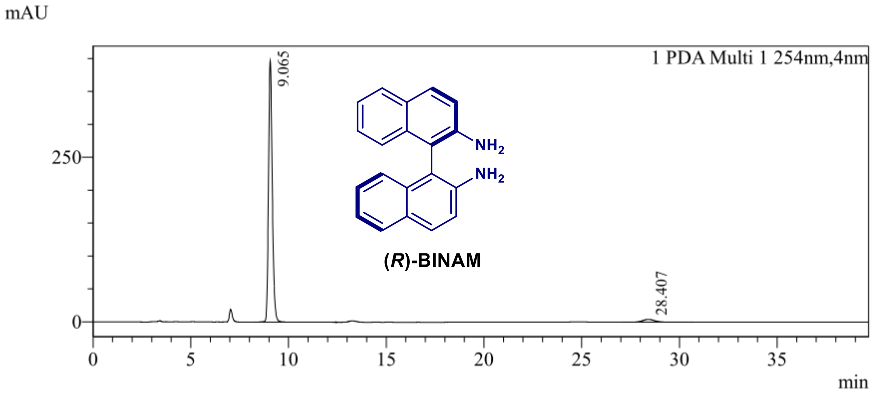

Peak Table

PDA Ch1 254nm

Peak#	Ret. Time	Peak End	Height	Area	Area%
1	9.065	9.835	396721	5322817	96.823
2	28.407	30.709	4189	174663	3.177
Total			400910	5497481	100.000

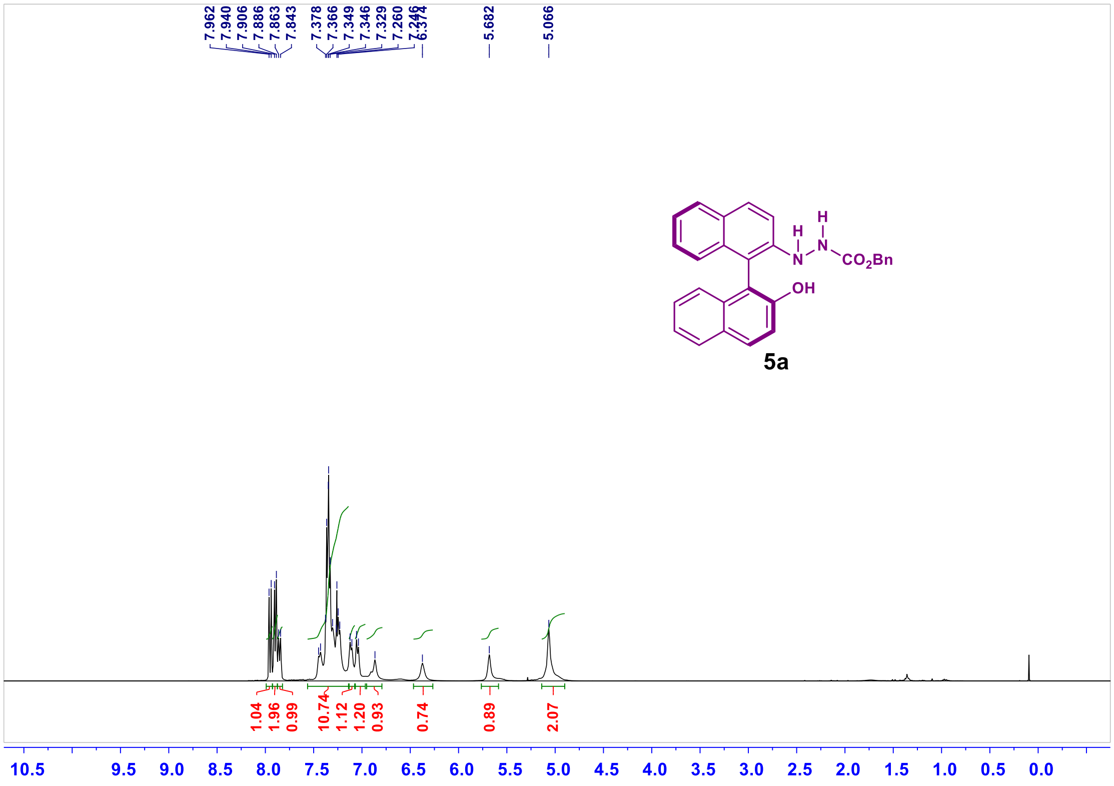

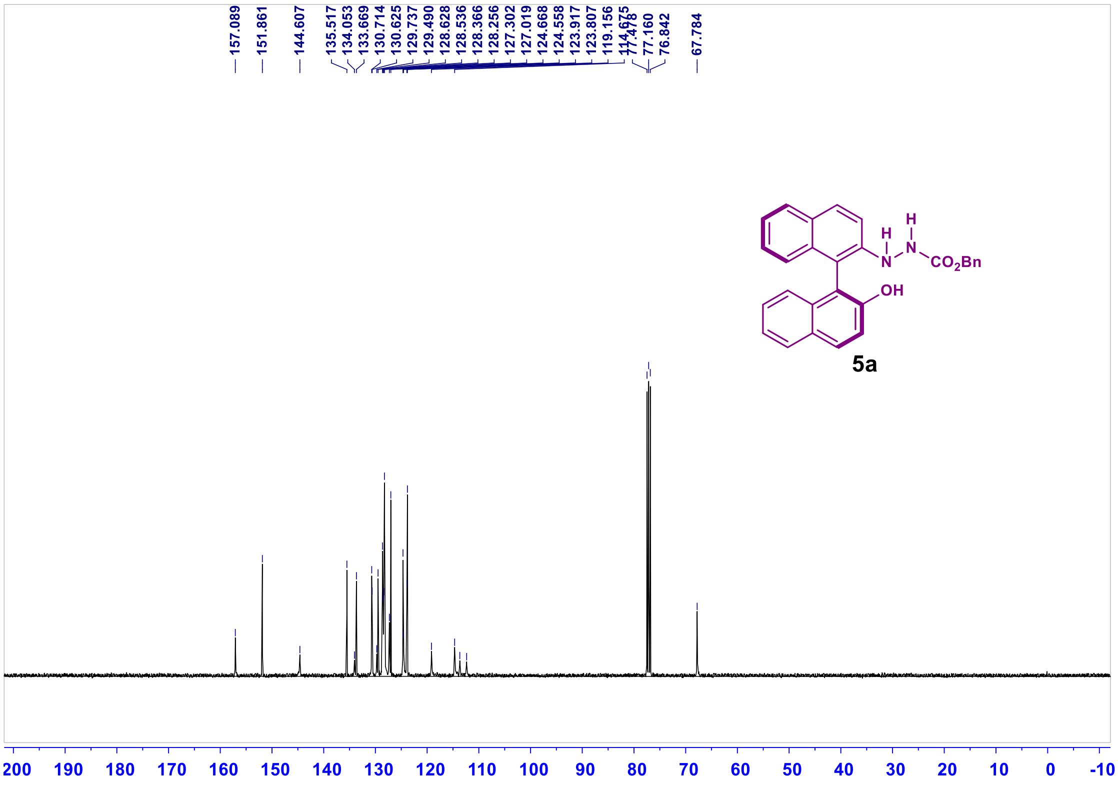

Supplementary Figure 90. ^{1}H and ^{13}C NMR spectra of 5a

Supplementary Figure 91. HPLC spectra of (*S*)-benzyl 2-(2'-hydroxy-[1,1'-binaphthalen]-2-yl)hydrazine-1-carboxylate (5a). Diacel Chiralpak AD-H, *n*-Hexane:*i*-PrOH = 80:20, flow = 1.0 mL/min, 25 °C, λ = 231 nm, t_R(major) = 14.4 min, t_R(minor) = 13.4 min, e.r. = 2.5:97.5

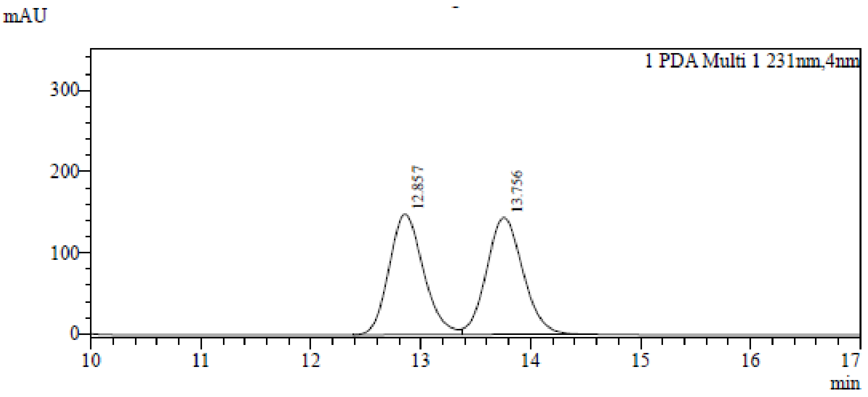

Peak Table

PDA Ch1 231nm

Peak#	Ret. Time	Peak End	Height	Area	Area%
1	12.857	13.376	148057	3250452	49.531
2	13.756	14.464	143640	3312065	50.469
Total			291697	6562517	100.000

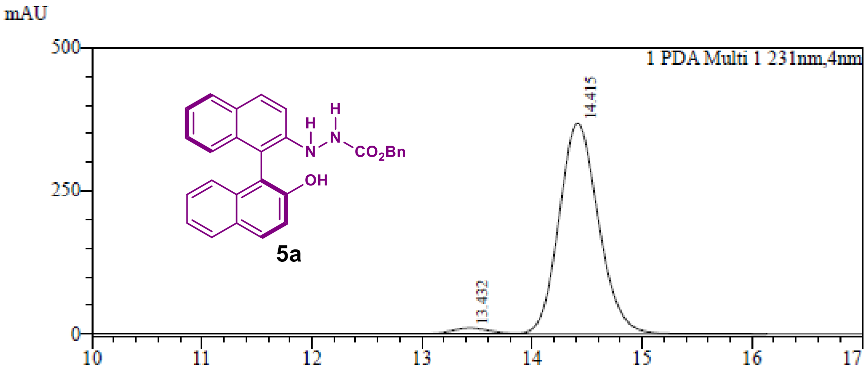

Peak Table

PDA Ch1 231nm

Peak#	Ret. Time	Peak End	Height	Area	Area%
1	13.432	13.856	10014	237191	2.519
2	14.415	16.128	367694	9178690	97.481
Total			377707	9415880	100.000

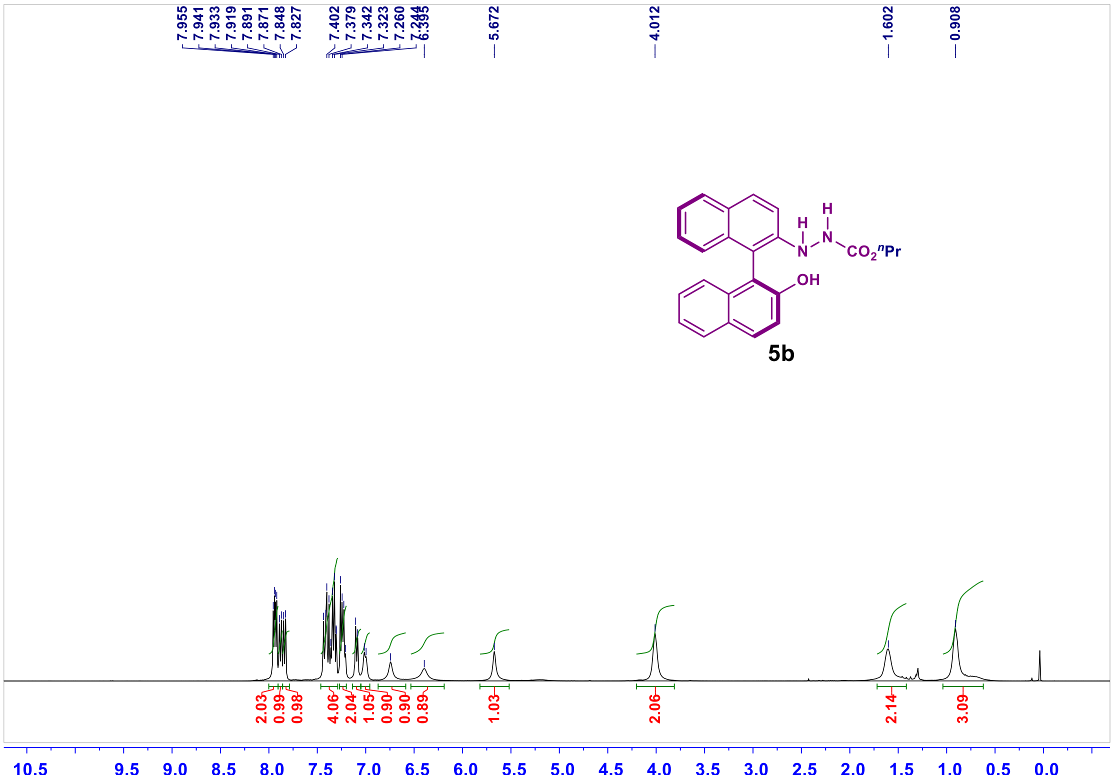

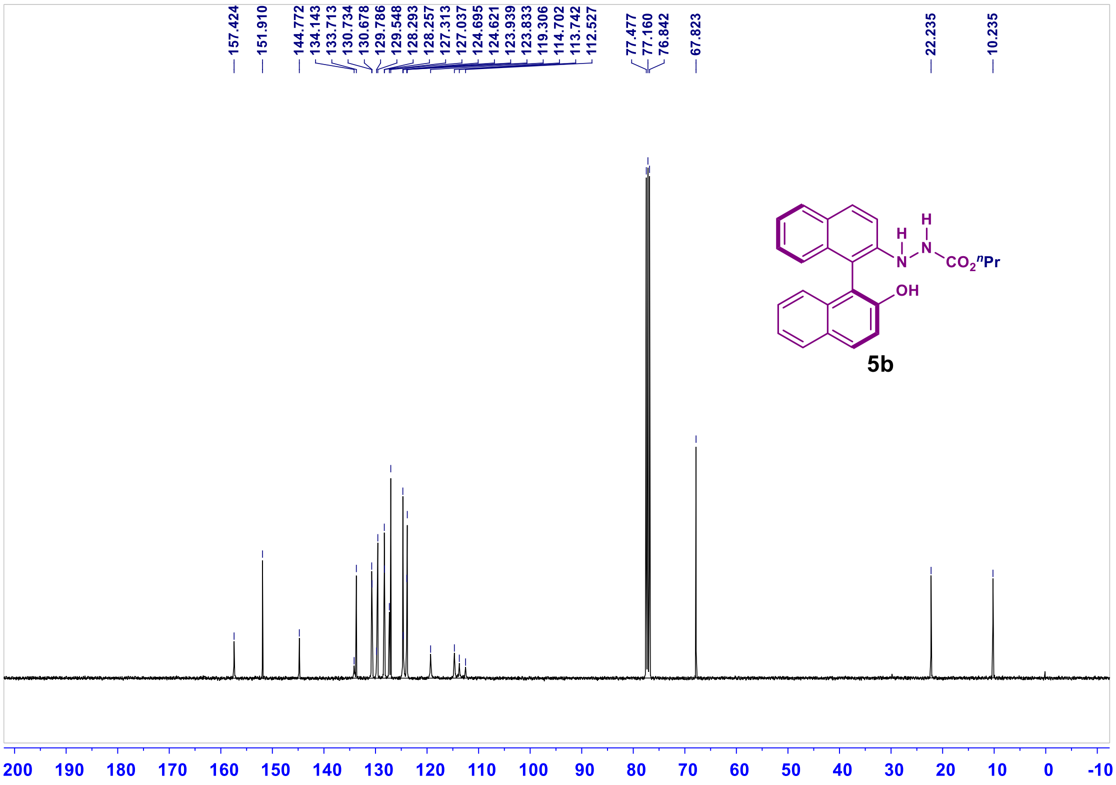

Supplementary Figure 92. ^{1}H and ^{13}C NMR spectra of 5b

Supplementary Figure 93. HPLC spectra of (*S*)-propyl 2-(2'-hydroxy-[1,1'-binaphthalen]-2-yl)hydrazine-1-carboxylate (5b). Diacel Chiralpak AD-H, *n*-Hexane:*i*-PrOH = 80:20, flow = 1.0 mL/min, 25 °C, λ = 230 nm, t_R(major) = 9.8 min, t_R(minor) = 8.9 min, e.r. = 3.5:96.5

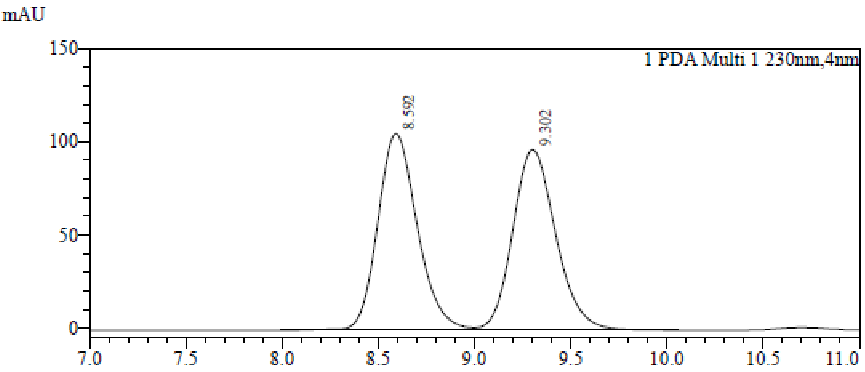

Peak Table

PDA Ch1 230nm

Peak#	Ret. Time	Peak End	Height	Area	Area%
1	8.592	8.992	105494	1512577	50.093
2	9.302	10.059	96999	1506936	49.907
Total			202493	3019512	100.000

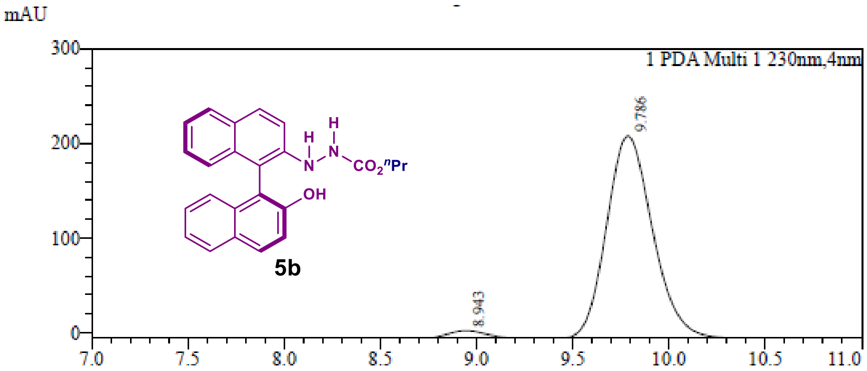

Peak Table

PDA Ch1 230nm

Peak#	Ret. Time	Peak End	Height	Area	Area%
1	8.943	9.376	8710	130855	3.513
2	9.786	10.763	214083	3594537	96.487
Total			222793	3725393	100.000

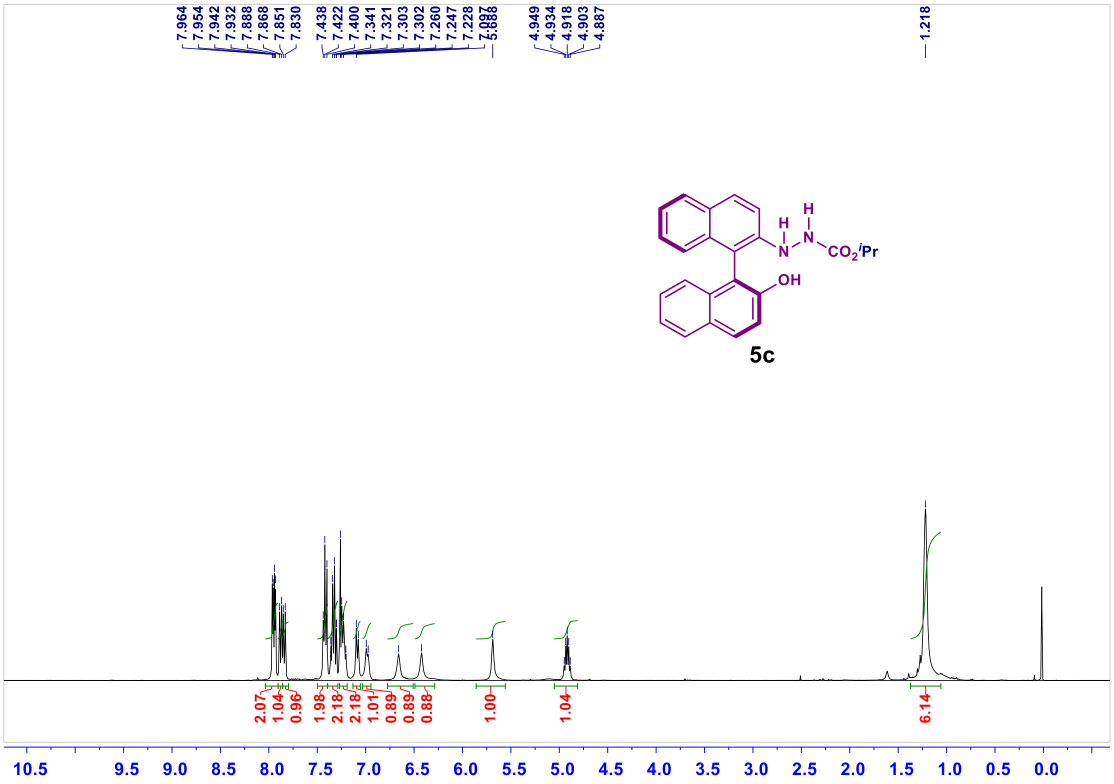

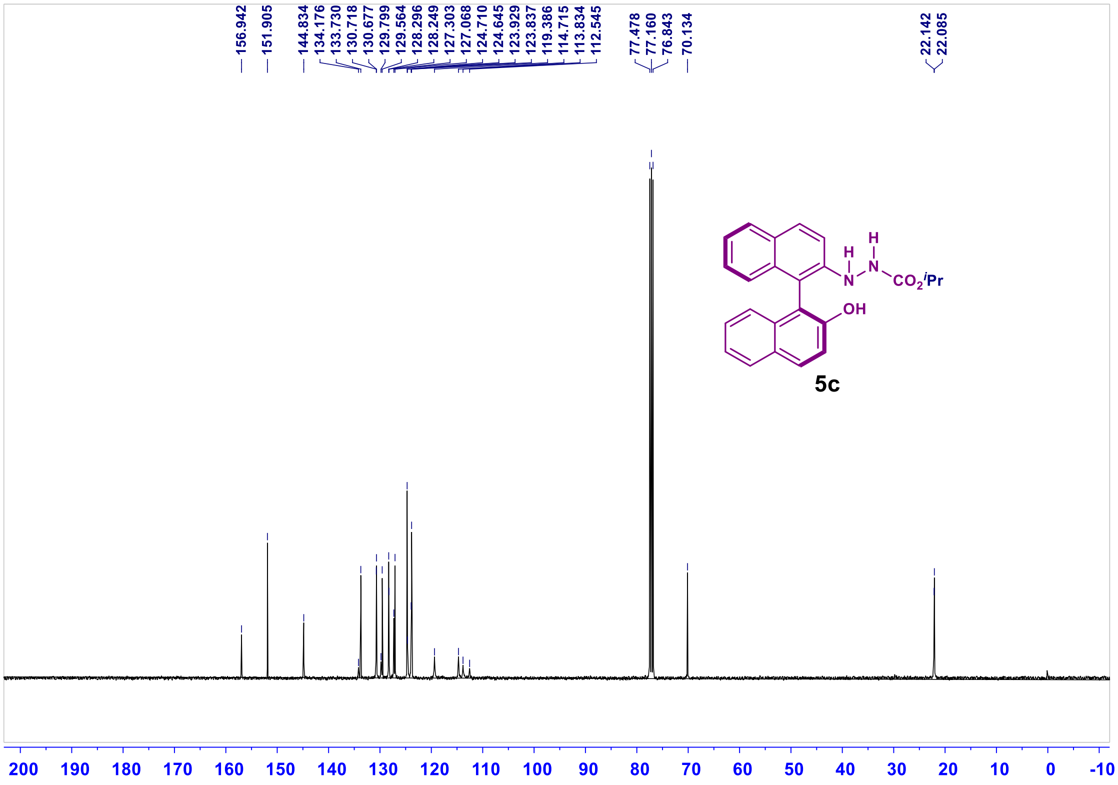

Supplementary Figure 94. ^{1}H and ^{13}C NMR spectra of 5c

Supplementary Figure 95. HPLC spectra of (*S*)-isopropyl 2-(2'-hydroxy-[1,1'-binaphthalen]-2-yl)hydrazine-1-carboxylate (5c). Diacel Chiralcel OD-H, *n*-Hexane:*i*-PrOH = 80:20, flow = 1.0 mL/min, 25 °C, λ = 230 nm, t_R(major) = 6.8 min, t_R(minor) = 6.1 min, e.r. = 8:92

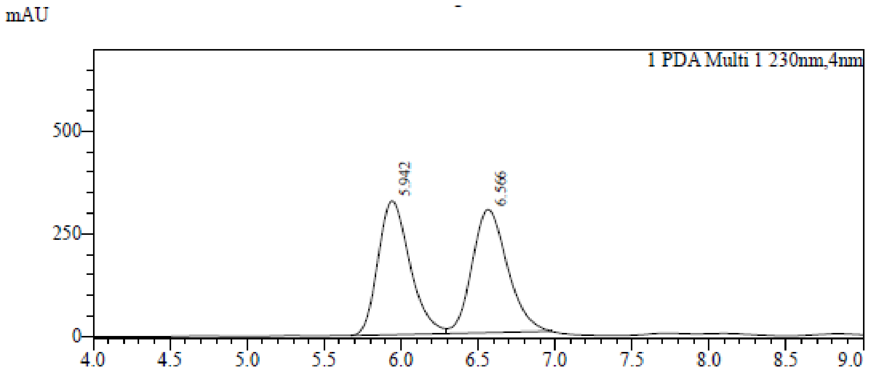

Peak Table

PDA Ch1 230nm

Peak#	Ret. Time	Peak End	Height	Area	Area%
1	5.942	6.293	326704	4722037	49.496
2	6.566	6.976	299846	4818163	50.504
Total			626550	9540199	100.000

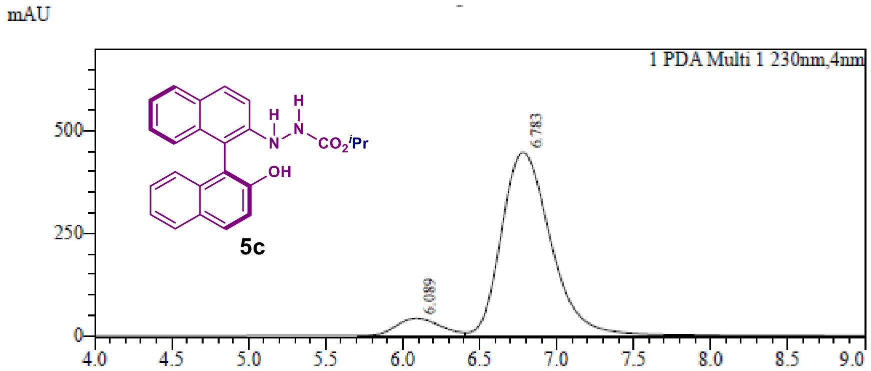

Peak Table

PDA Ch1 230nm

Peak#	Ret. Time	Peak End	Height	Area	Area%
1	6.089	6.411	42782	855745	7.832
2	6.783	9.600	447359	10070943	92.168
Total			490141	10926688	100.000

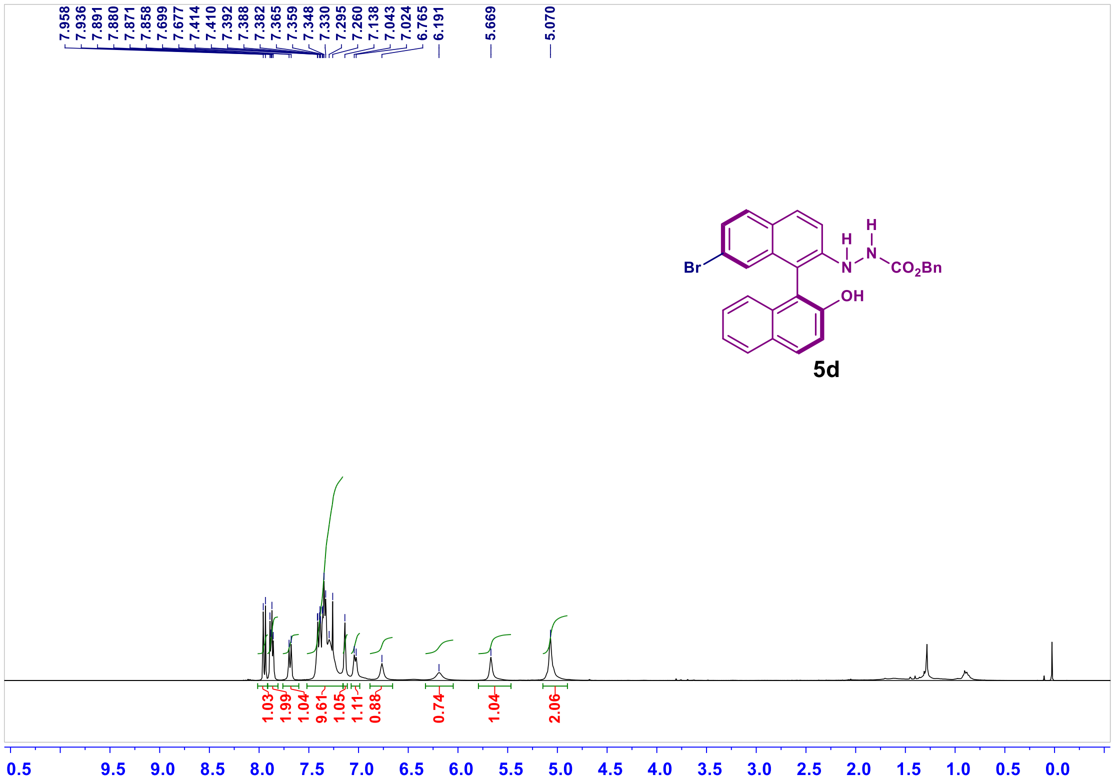

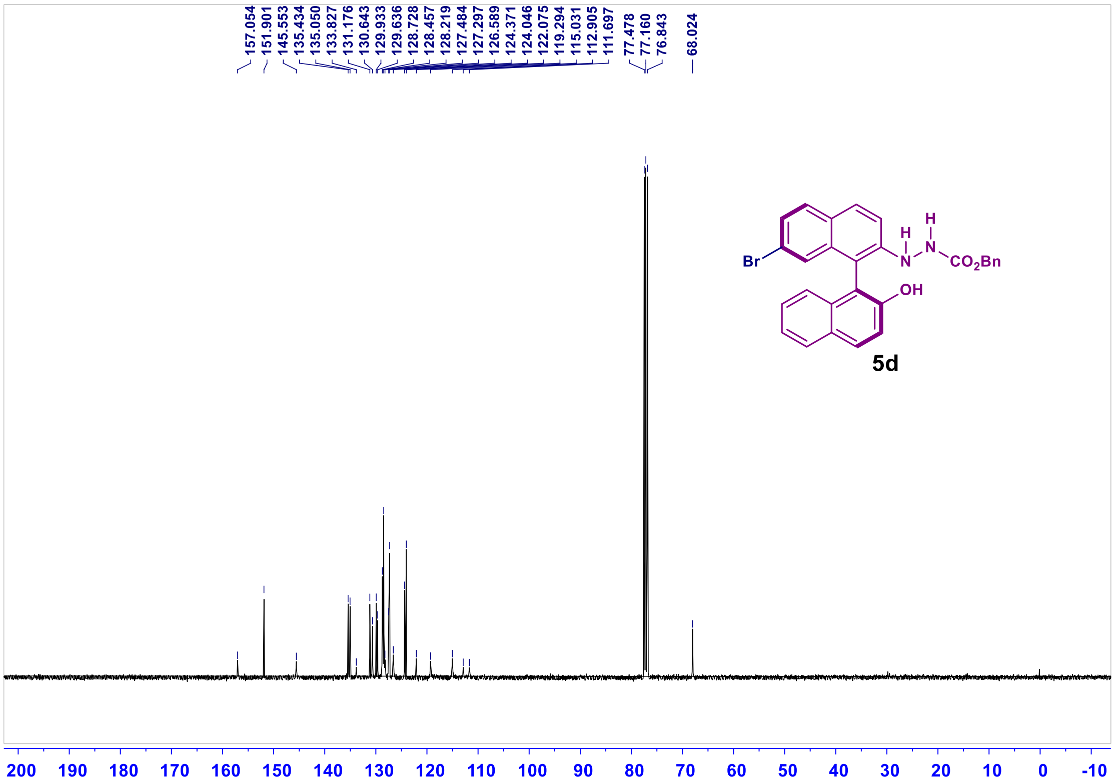

Supplementary Figure 96. ^{1}H and ^{13}C NMR spectra of 5d

Supplementary Figure 97. HPLC spectra of (*S*)-benzyl 2-(7-bromo-2'-hydroxy-[1,1'-binaphthalen]-2-yl)hydrazine-1-carboxylate (5d). Diacel Chiralpak AD-H, *n*-Hexane:*i*-PrOH = 80:20, flow = 1.0 mL/min, 25 °C, λ = 229 nm, t_R(major) = 11.4 min, t_R(minor) = 10.7 min, e.r. = 3.5:96.5

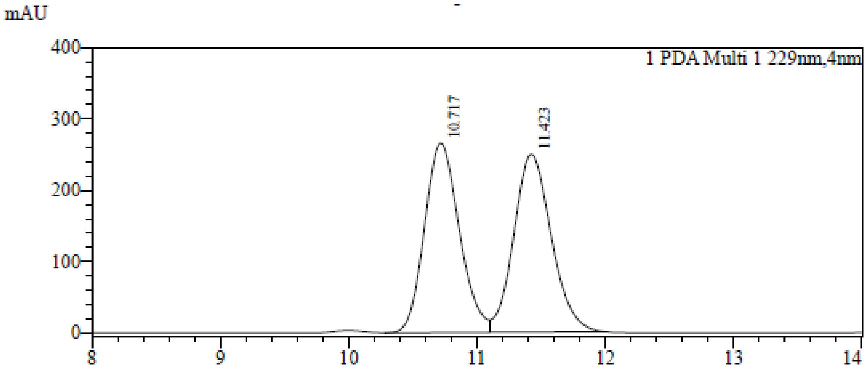

Peak Table

PDA Ch1 229nm

Peak#	Ret. Time	Peak End	Height	Area	Area%
1	10.717	11.093	265737	5008216	49.616
2	11.423	11.989	249540	5085839	50.384
Total			515277	10094055	100.000

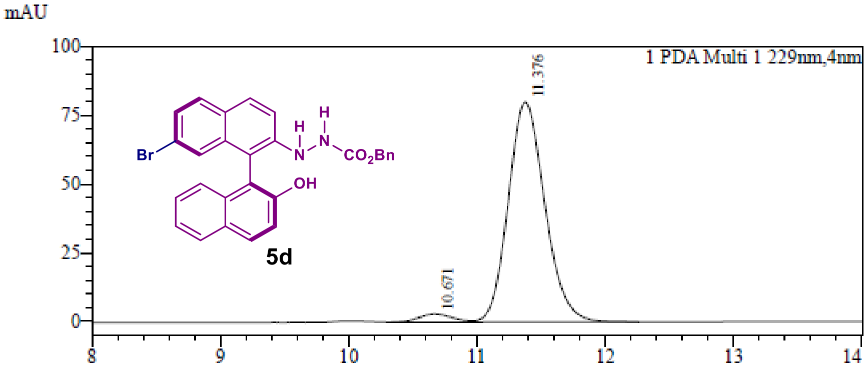

Peak Table

PDA Ch1 229nm

Peak#	Ret. Time	Peak End	Height	Area	Area%
1	10.671	10.955	2959	54656	3.314
2	11.376	12.256	80036	1594442	96.686
Total			82995	1649098	100.000

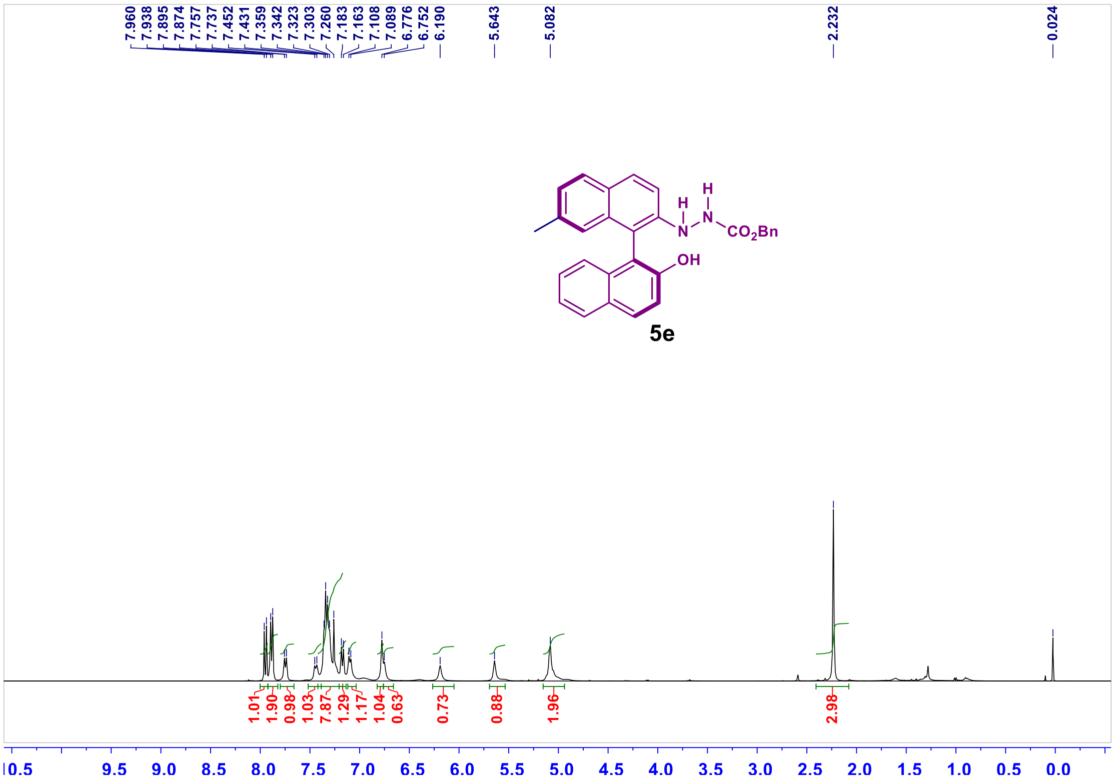

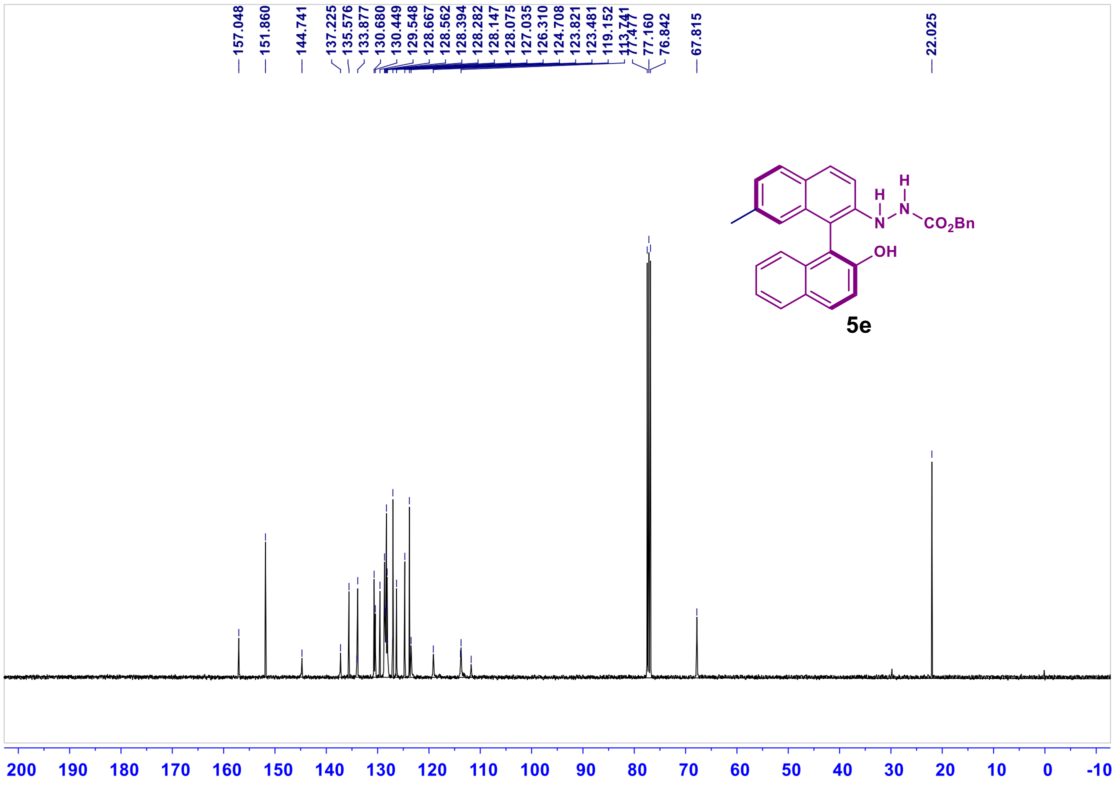

Supplementary Figure 98. ^{1}H and ^{13}C NMR spectra of 5e

Supplementary Figure 99. HPLC spectra of (*S*)-benzyl 2-(2'-hydroxy-7-methyl-[1,1'-binaphthalen]-2-yl)hydrazine-1-carboxylate (5e). Diacel Chiralpak AD-H, *n*-Hexane:*i*-PrOH = 80:20, flow = 1.0 mL/min, 25 °C, λ = 229 nm, t_R(major) = 11.5 min, t_R(minor) = 9.3 min, e.r. = 4:96

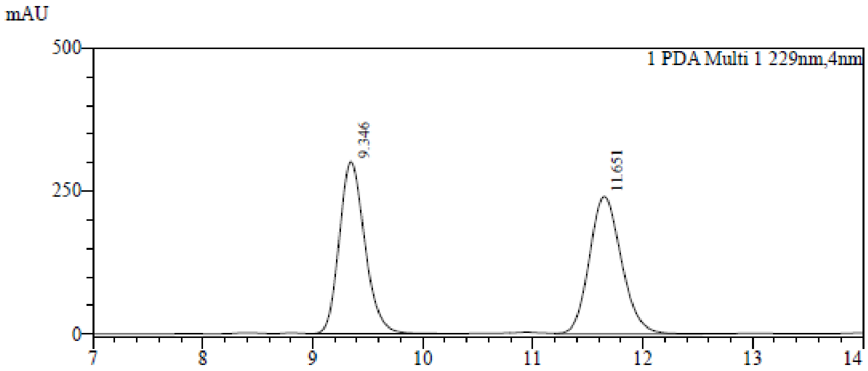

Peak Table

PDA Ch1 229nm

Peak#	Ret. Time	Peak End	Height	Area	Area%
1	9.346	10.261	301932	4935579	50.094
2	11.651	12.619	240485	4917061	49.906
Total			542417	9852640	100.000

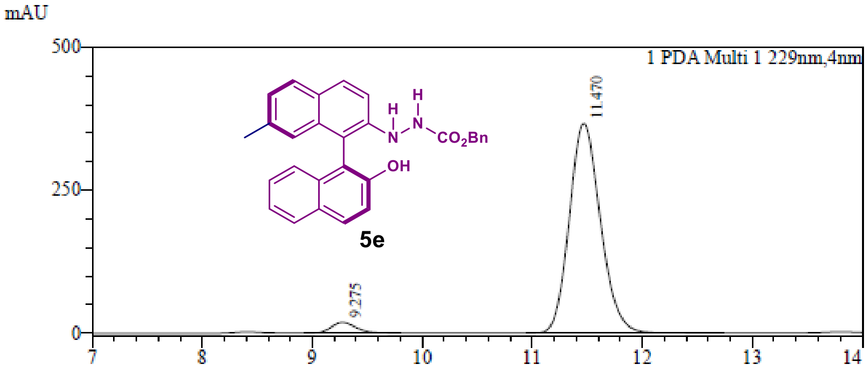

Peak Table

PDA Ch1 229nm

Peak#	Ret. Time	Peak End	Height	Area	Area%
1	9.275	9.803	19129	302983	4.013
2	11.470	12.747	367047	7247893	95.987
Total			386176	7550876	100.000

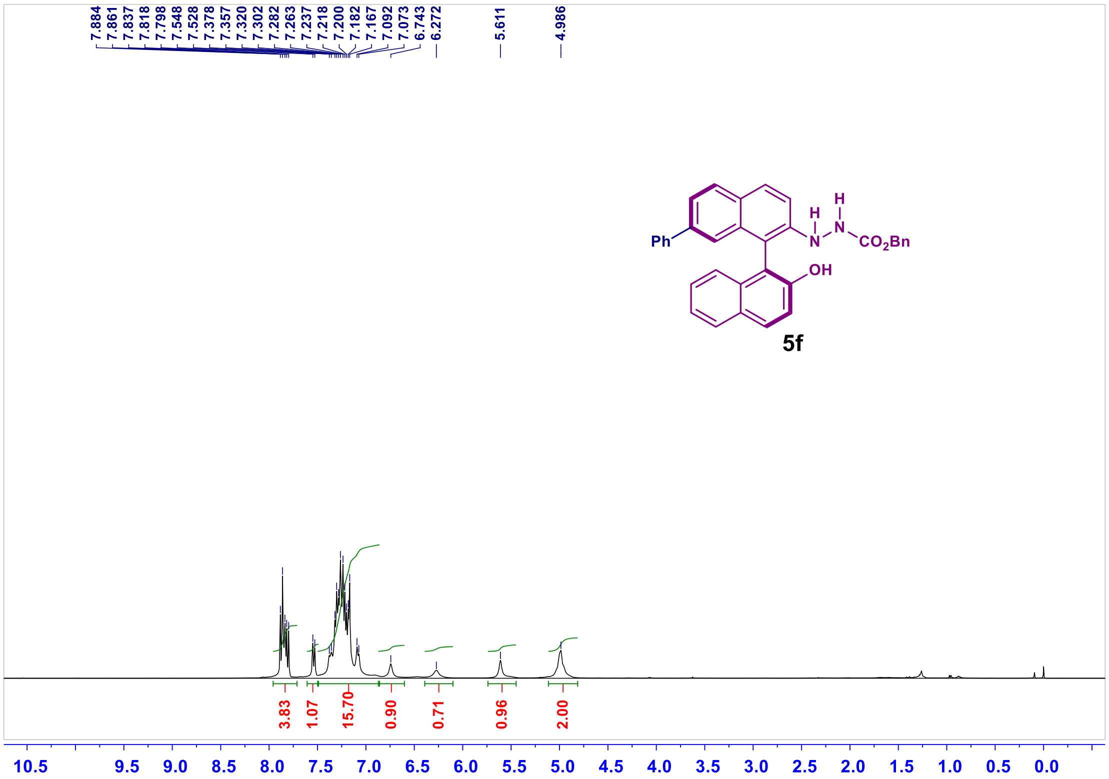

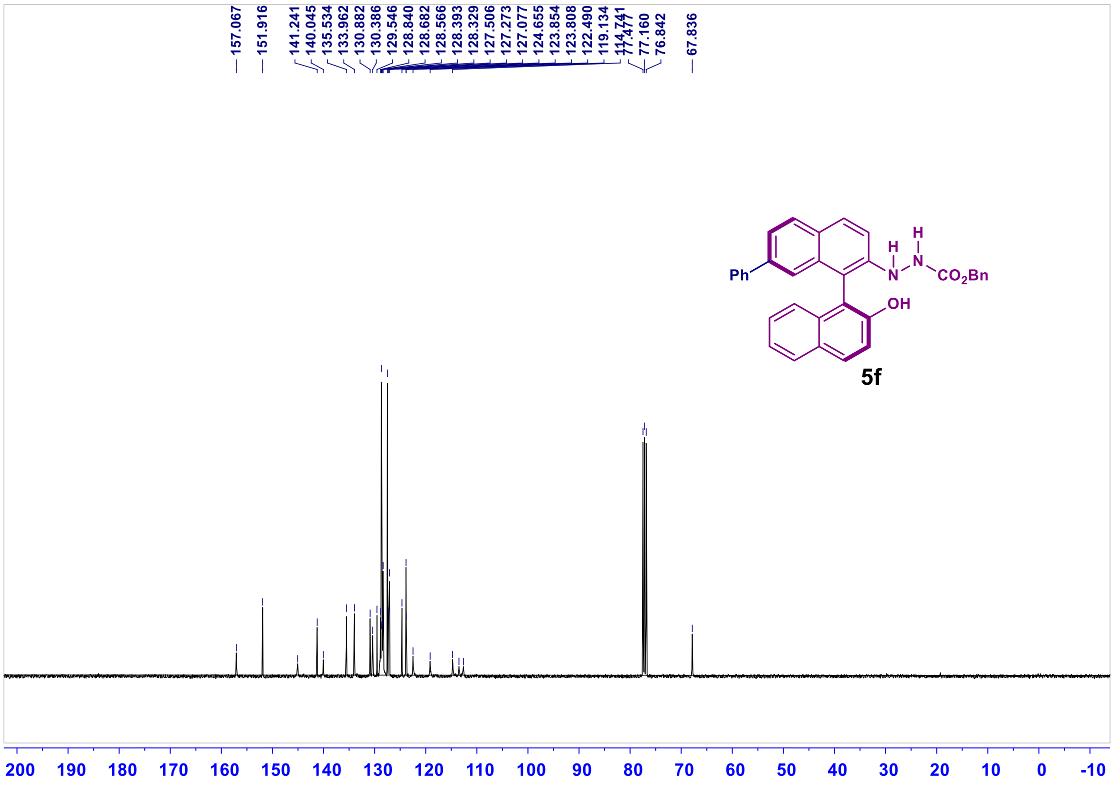

Supplementary Figure 100. ^{1}H and ^{13}C NMR spectra of 5f

Supplementary Figure 101. HPLC spectra of (*S*)-benzyl 2-(2'-hydroxy-7-phenyl-[1,1'-binaphthalen]-2-yl)hydrazine-1-carboxylate (5f). Diacel Chiralpak AD-H, *n*-Hexane:*i*-PrOH = 80:20, flow = 1.0 mL/min, 25 °C, λ = 228 nm, t_R(major) = 13.8 min, t_R(minor) = 14.9 min, e.r. = 4:96

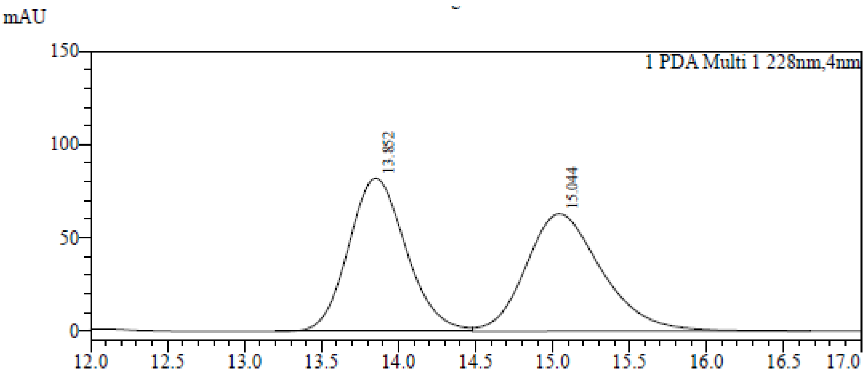

Peak Table

PDA Ch1 228nm

Peak#	Ret. Time	Peak End	Height	Area	Area%
1	13.852	14.475	82170	2132227	49.692
2	15.044	16.672	62959	2158693	50.308
Total			145129	4290920	100.000

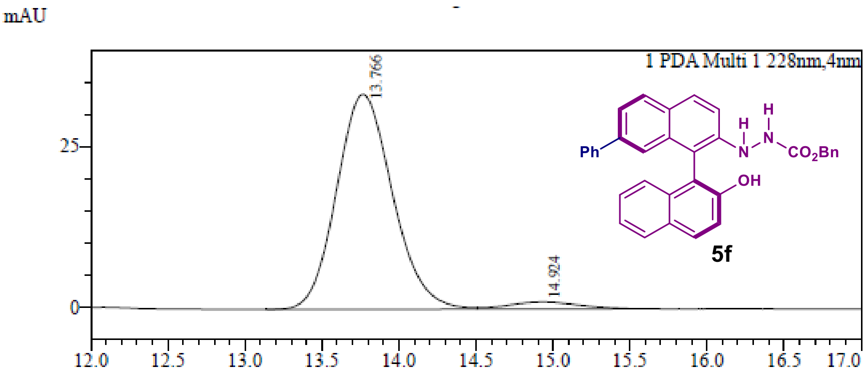

Peak Table

PDA Ch1 228nm

Peak#	Ret. Time	Peak End	Height	Area	Area%
1	13.766	14.507	33468	849115	96.011
2	14.924	15.669	1110	35274	3.989
Total			34578	884389	100.000

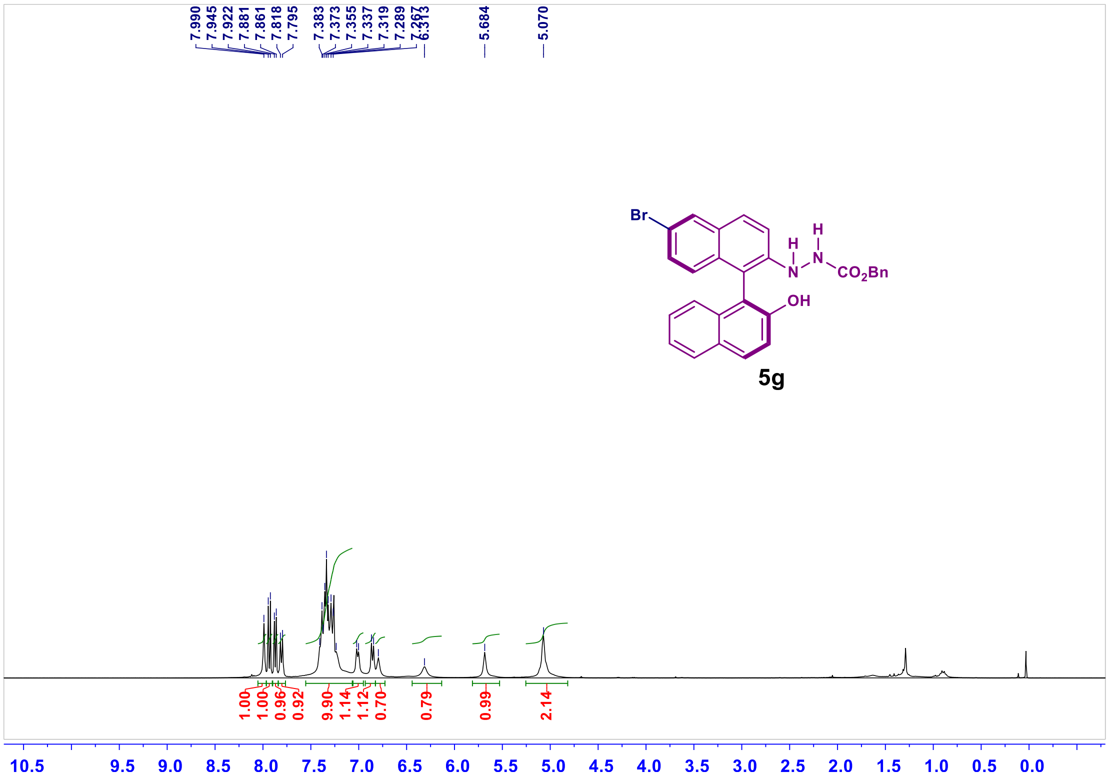

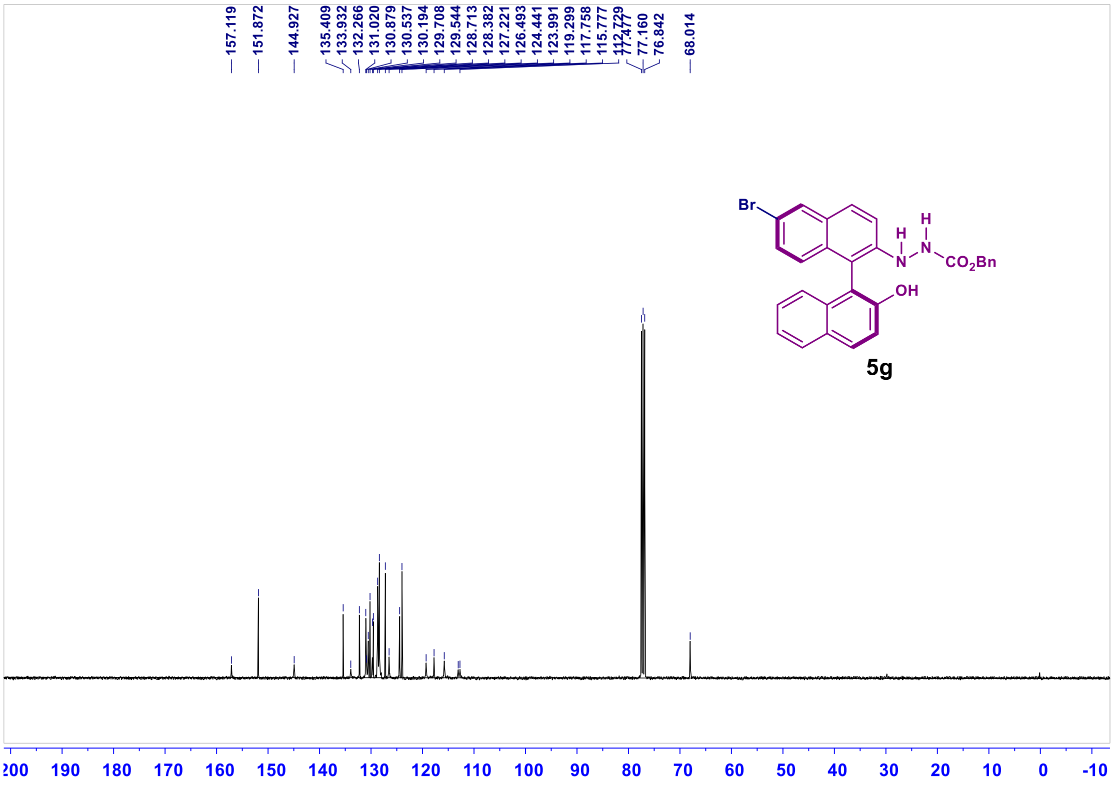

Supplementary Figure 102. ^{1}H and ^{13}C NMR spectra of 5g

Supplementary Figure 103. HPLC spectra of (*S*)-benzyl 2-(6-bromo-2'-hydroxy-[1,1'-binaphthalen]-2-yl)hydrazine-1-carboxylate (5g). Diacel Chiralpak AD-H, *n*-Hexane:*i*-PrOH = 80:20, flow = 1.0 mL/min, 25 °C, λ = 229 nm, t_R(major) = 13.5 min, t_R(minor) = 11.1 min, e.r. = 4.5:95.5

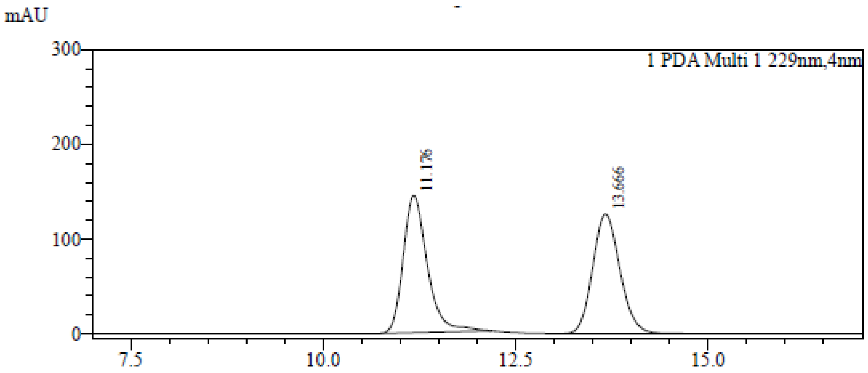

Peak Table

PDA Ch1 229nm

Peak#	Ret. Time	Peak End	Height	Area	Area%
1	11.176	12.181	144936	3169299	50.285
2	13.666	14.944	126514	3133319	49.715
Total			271450	6302618	100.000

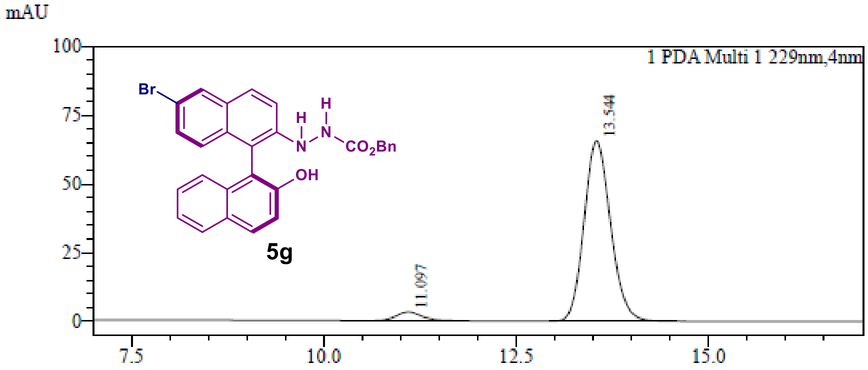

Peak Table

PDA Ch1 229nm

Peak#	Ret. Time	Peak End	Height	Area	Area%
1	11.097	11.861	3206	74209	4.492
2	13.544	14.581	65656	1577880	95.508
Total			68862	1652089	100.000

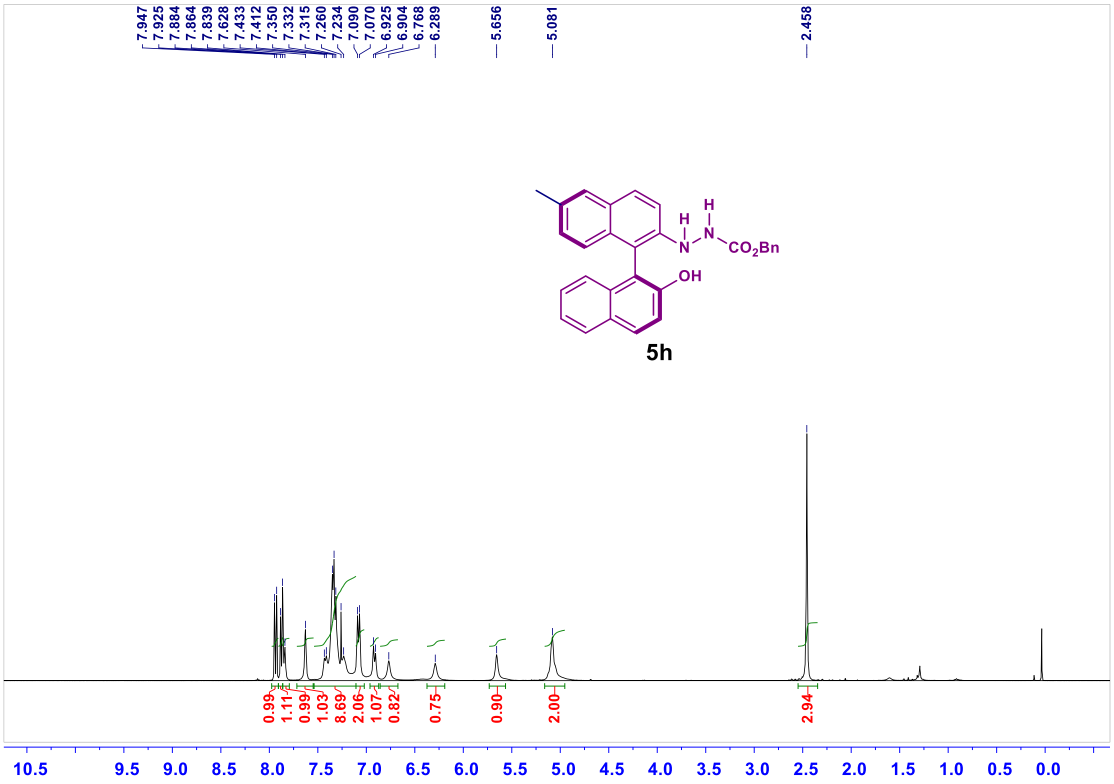

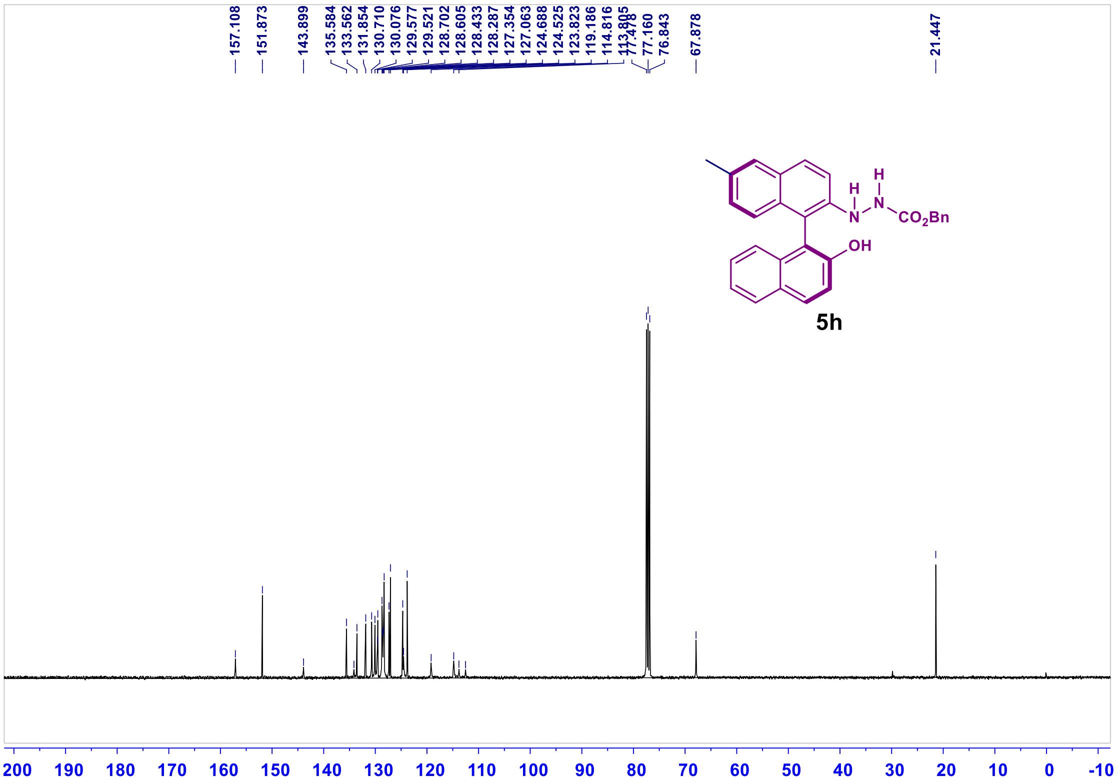

Supplementary Figure 104. ^{1}H and ^{13}C NMR spectra of 5h

Supplementary Figure 105. HPLC spectra of (*S*)-benzyl 2-(2'-hydroxy-6-methyl-[1,1'-binaphthalen]-2-yl)hydrazine-1-carboxylate (5h). Diacel Chiralpak AD-H, *n*-Hexane:*i*-PrOH = 80:20, flow = 1.0 mL/min, 25 °C, λ = 230 nm, t_R(major) = 13.3 min, t_R(minor) = 11.3 min, e.r. = 4:96

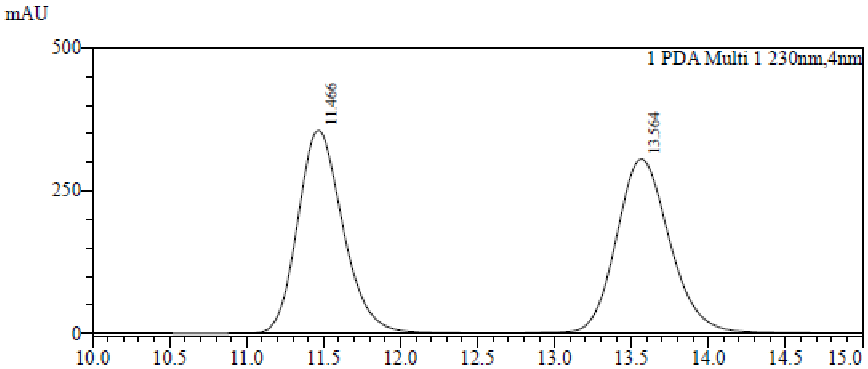

Peak Table

PDA Ch1 230nm

Peak#	Ret. Time	Peak End	Height	Area	Area%
1	11.466	12.661	355819	7313680	49.535
2	13.564	14.923	305779	7451091	50.465
Total			661597	14764771	100.000

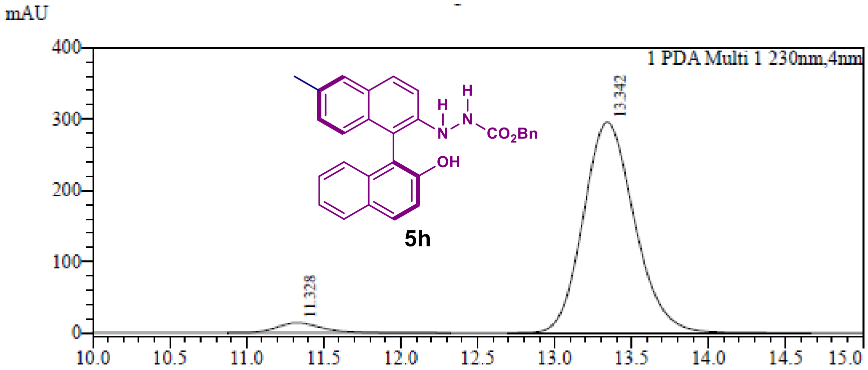

Peak Table

PDA Ch1 230nm

Peak#	Ret. Time	Peak End	Height	Area	Area%
1	11.328	12.320	13785	279642	3.926
2	13.342	14.667	295605	6842894	96.074
Total			309390	7122536	100.000

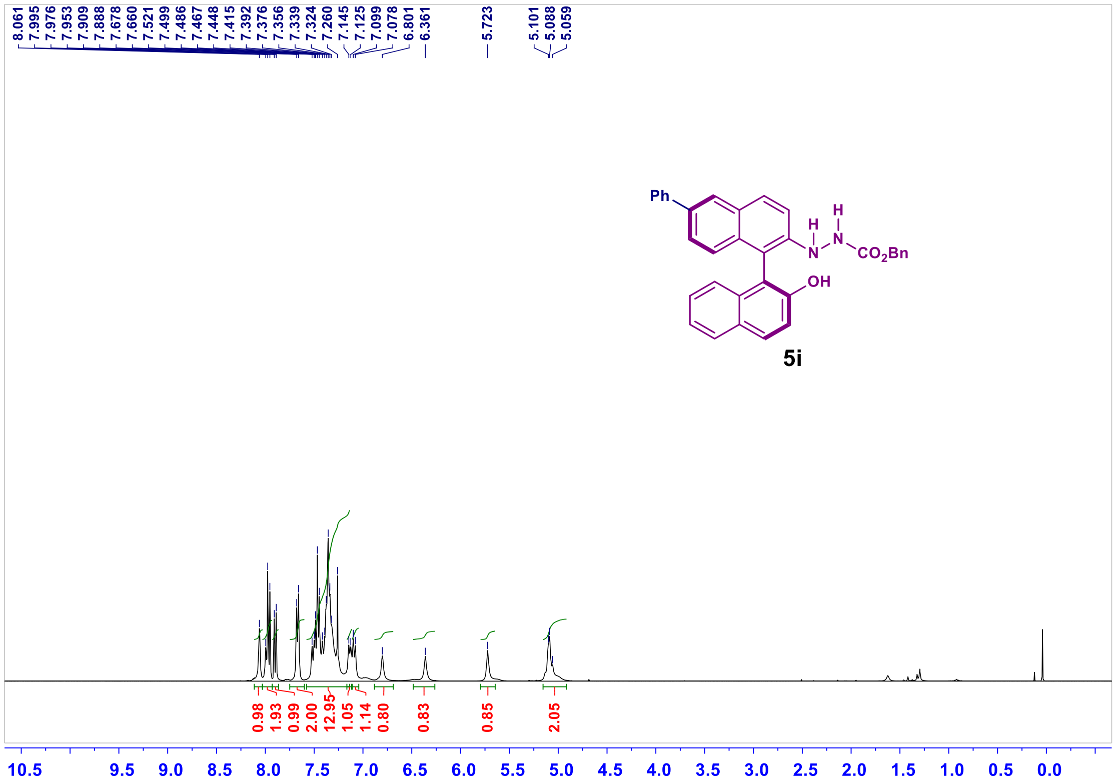

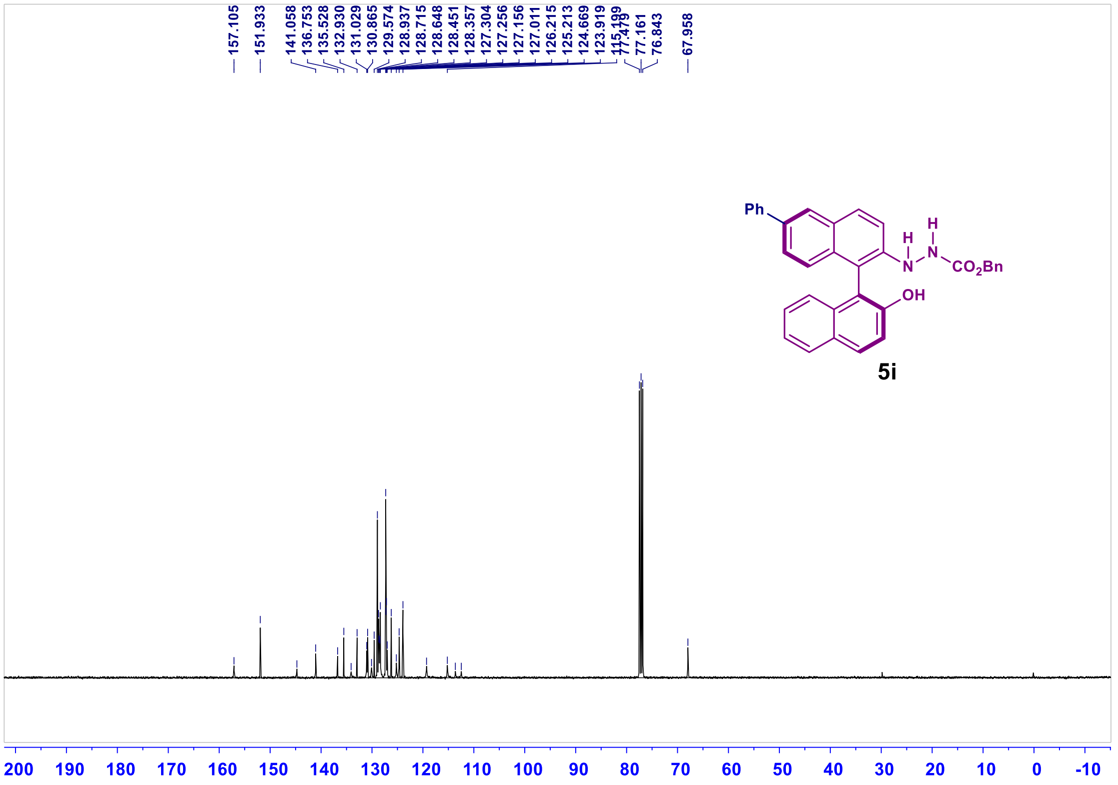

Supplementary Figure 106. ^{1}H and ^{13}C NMR spectra of 5i

Supplementary Figure 107. HPLC spectra of (*S*)-benzyl 2-(2'-hydroxy-6-phenyl-[1,1'-binaphthalen]-2-yl)hydrazine-1-carboxylate (5i). Diacel Chiralpak AS-H, *n*-Hexane:*i*-PrOH = 80:20, flow = 1.0 mL/min, 25 °C, λ = 228 nm, t_R(major) = 26.5 min, t_R(minor) = 18.3 min, e.r. = 6:94

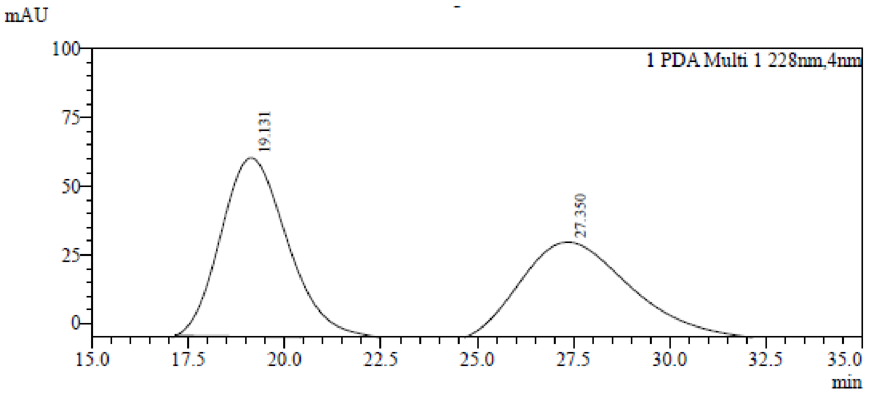

Peak Table

PDA Ch1 228nm

Peak#	Ret. Time	Peak End	Height	Area	Area%
1	19.131	22.613	65094	7876807	49.950
2	27.350	34.891	36664	7892711	50.050
Total			101758	15769518	100.000

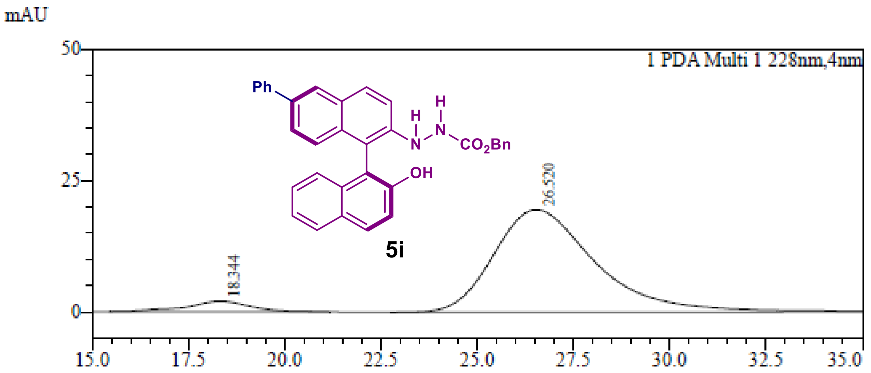

Peak Table

PDA Ch1 228nm

Peak#	Ret. Time	Peak End	Height	Area	Area%
1	18.344	21.152	1996	226807	5.881
2	26.520	38.123	19540	3629575	94.119
Total			21536	3856383	100.000

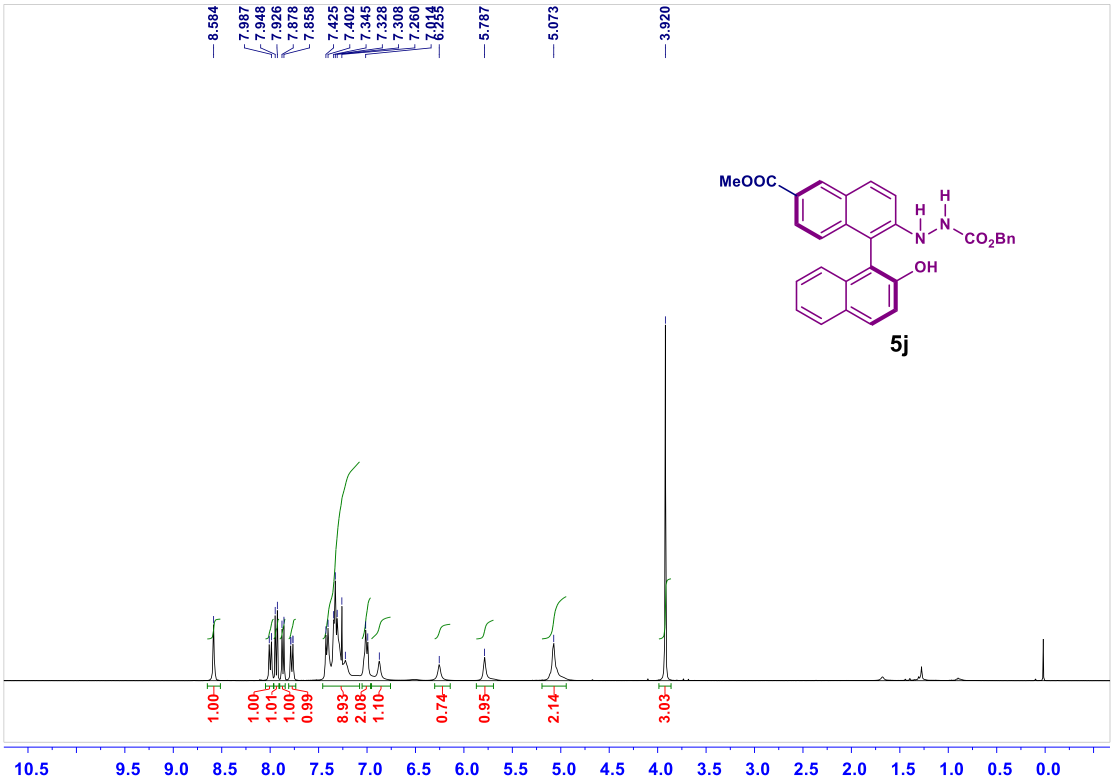

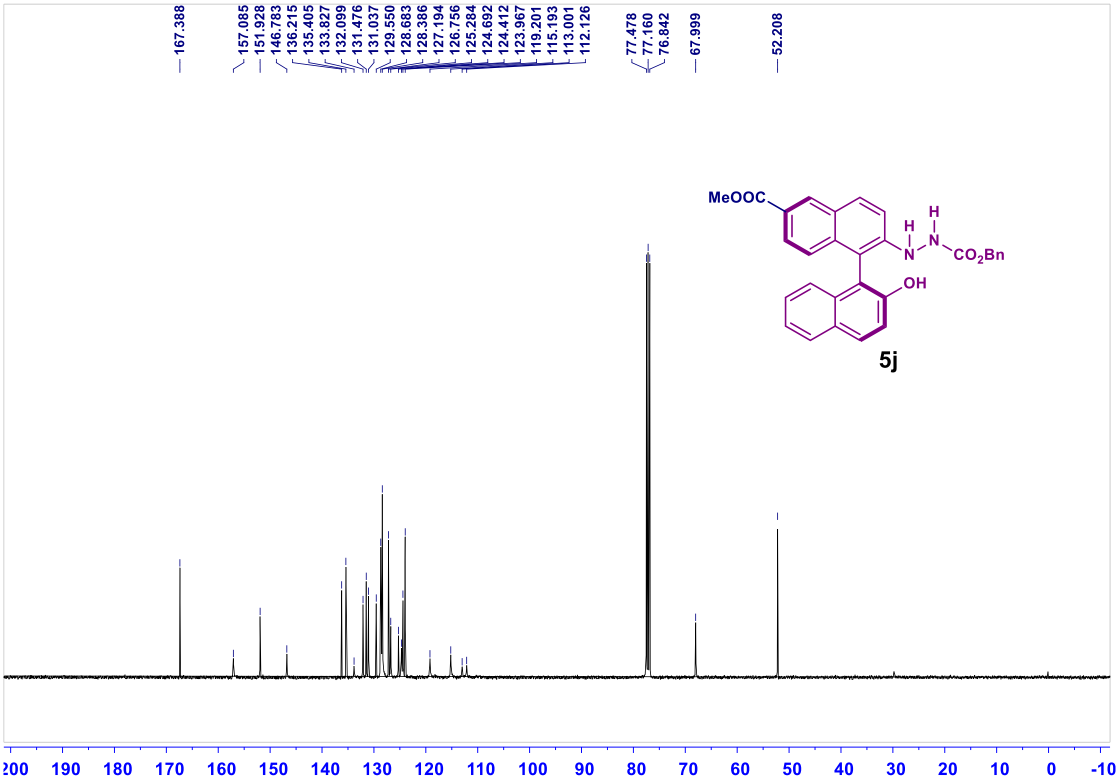

Supplementary Figure 108. ^{1}H and ^{13}C NMR spectra of 5j

Supplementary Figure 109. HPLC spectra of (*S*)-benzyl 2-(2'-hydroxy-6-(methoxycarbonyl)-[1,1'-binaphthalen]-2-yl)hydrazine-1-carbox ylate (5j). Diacel Chiralcel OD-H, *n*-Hexane:*i*-PrOH = 80:20, flow = 1.0 mL/min, 25 °C, λ = 229 nm, t_R(major) = 16.9 min, t_R(minor) = 21.1 min, e.r. = 3:97

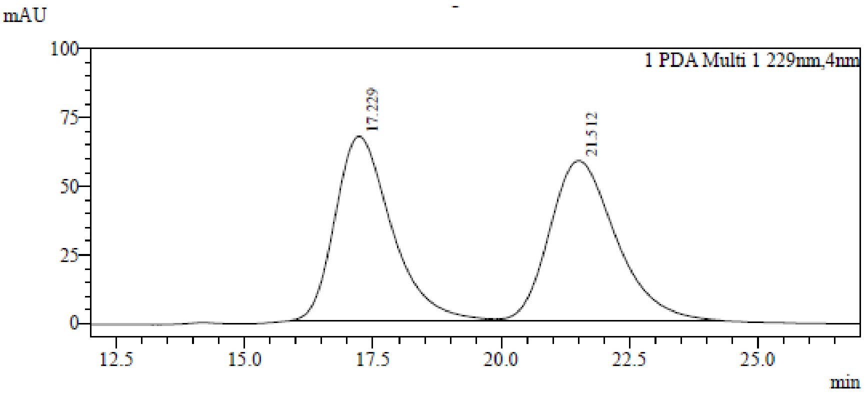

Peak Table

PDA Ch1 229nm

Peak#	Ret. Time	Peak End	Height	Area	Area%
1	17.229	19.915	67054	5149897	49.554
2	21.512	24.309	58180	5242628	50.446
Total			125234	10392526	100.000

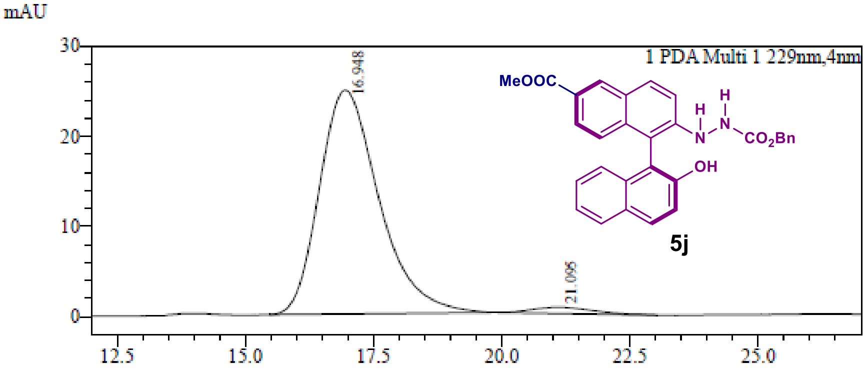

Peak Table

PDA Ch1 229nm

Peak#	Ret. Time	Peak End	Height	Area	Area%
1	16.948	19.872	24801	2007883	97.203
2	21.095	23.019	712	57767	2.797
Total			25513	2065650	100.000

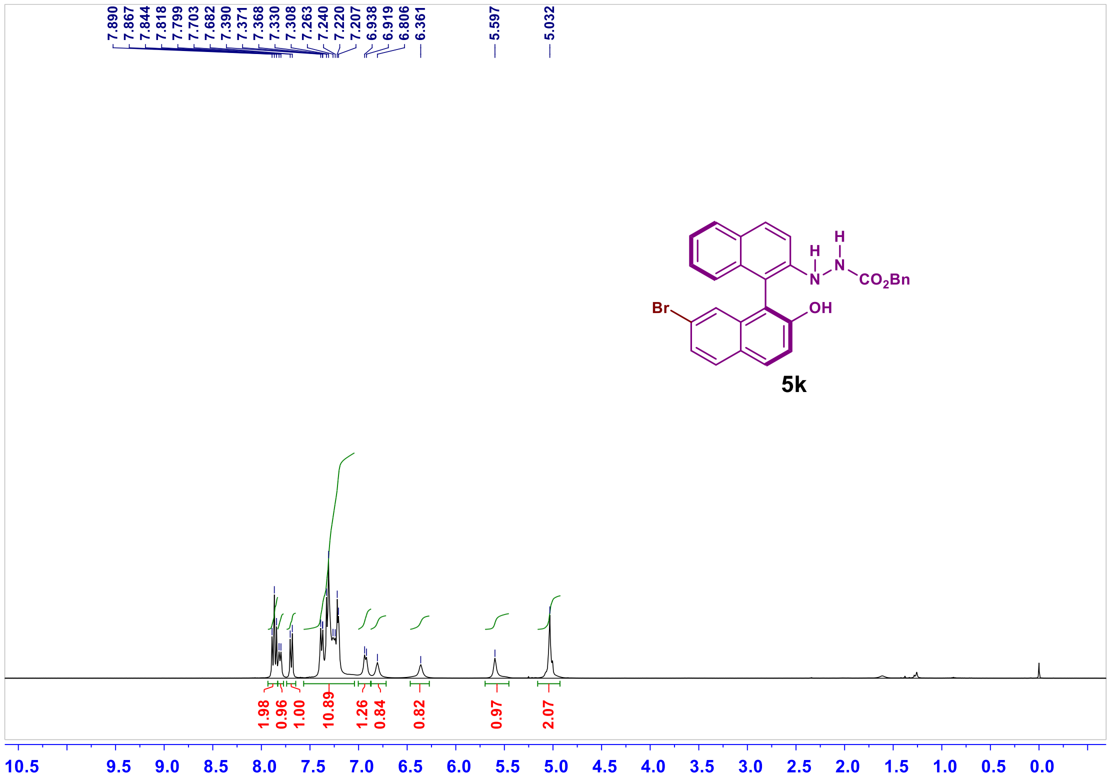

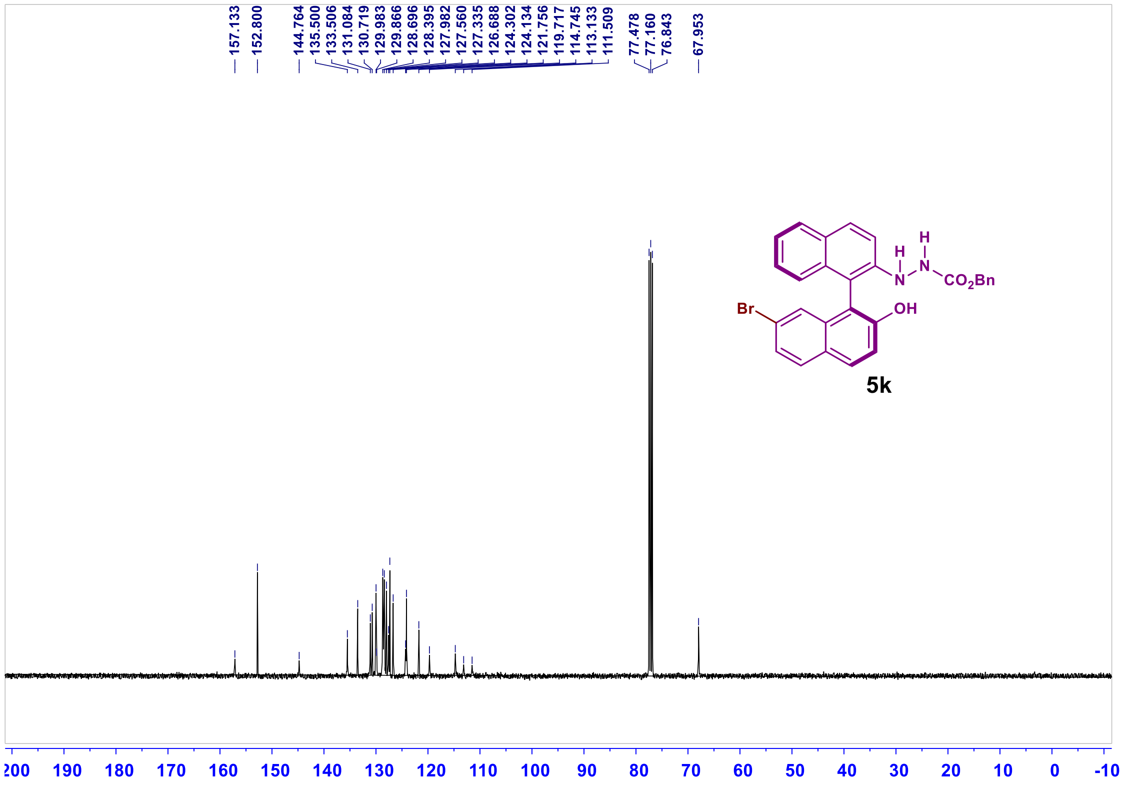

Supplementary Figure 110. ^{1}**H and** ^{13}**C NMR spectra of 5k**

Supplementary Figure 111. HPLC spectra of (*S*)-benzyl 2-(7'-bromo-2'-hydroxy-[1,1'-binaphthalen]-2-yl)hydrazine-1-carboxylate (5k). Diacel Chiralcel OD-H, *n*-Hexane:*i*-PrOH = 80:20, flow = 1.0 mL/min, 25 °C, λ = 238 nm, t_R(major) = 10.1 min, t_R(minor) = 12.3 min, e.r. = 7:93

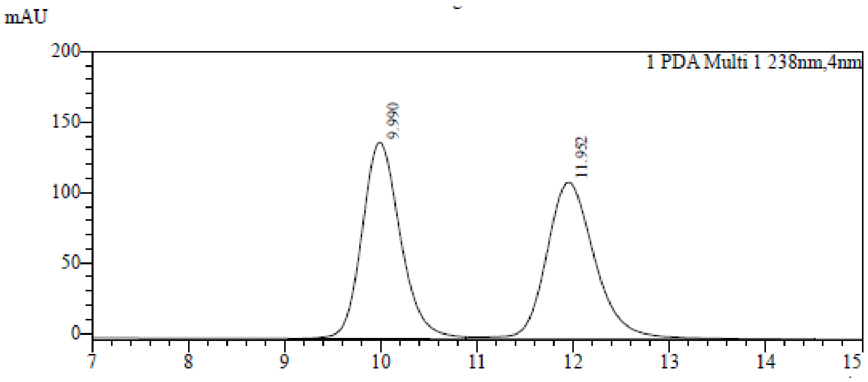

Peak Table

PDA Ch1 238nm

Peak#	Ret. Time	Peak End	Height	Area	Area%
1	9.990	10.891	139237	3875974	49.708
2	11.952	14.507	111131	3921458	50.292
Total			250368	7797431	100.000

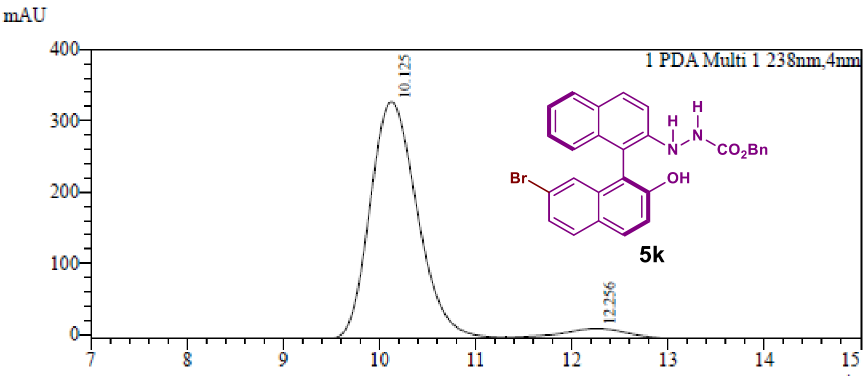

Peak Table

PDA Ch1 238nm

Peak#	Ret. Time	Peak End	Height	Area	Area%
1	10.125	11.307	333727	11501377	92.864
2	12.256	14.165	15784	883761	7.136
Total			349511	12385137	100.000

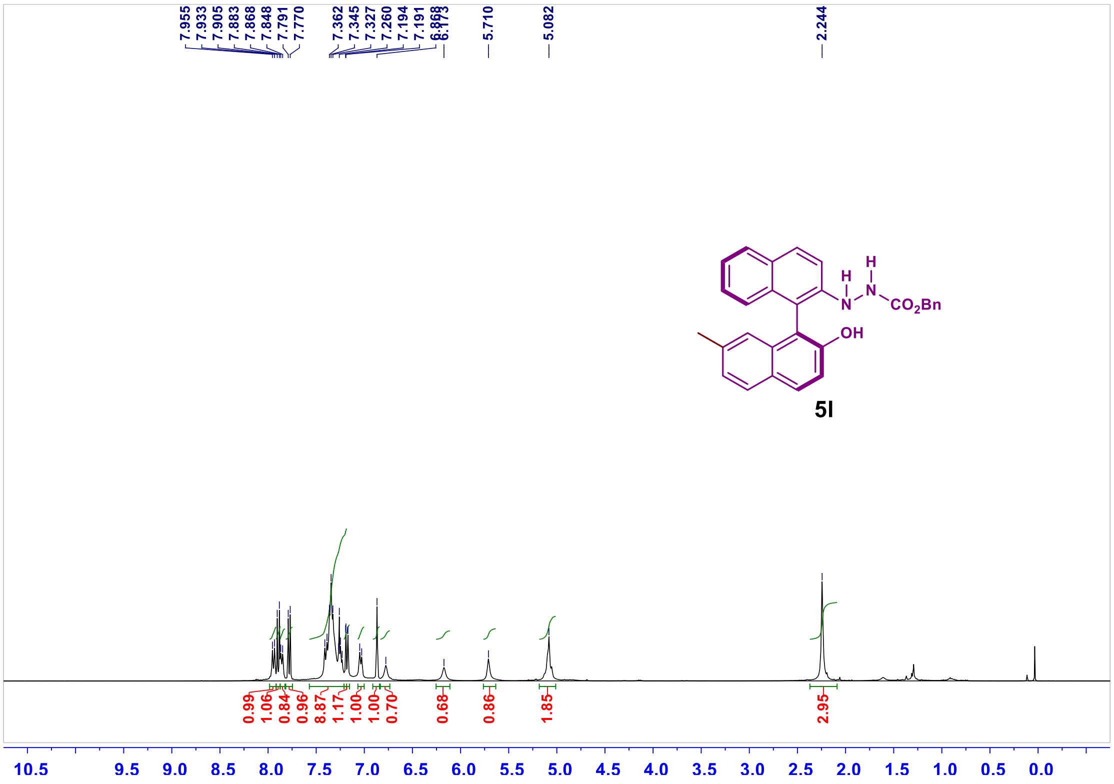

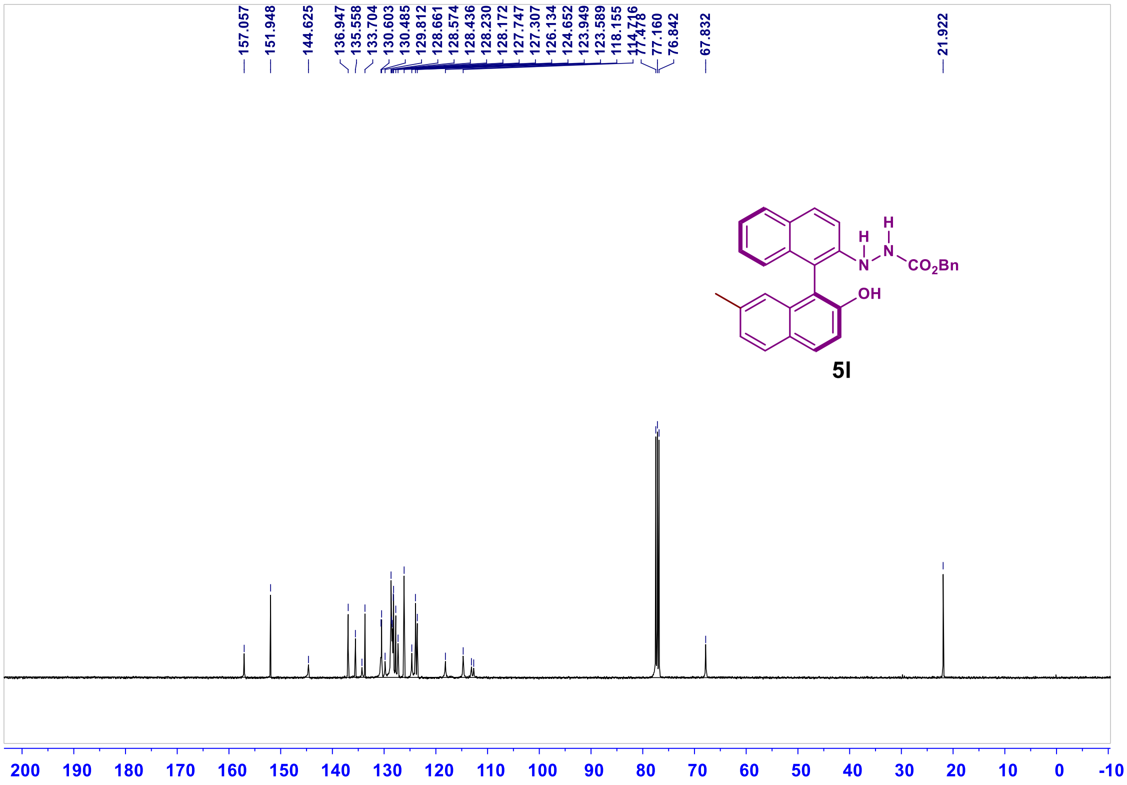

Supplementary Figure 112. ^{1}H and ^{13}C NMR spectra of 5l

Supplementary Figure 113. HPLC spectra of (*S*)-benzyl 2-(2'-hydroxy-7'-methyl-[1,1'-binaphthalen]-2-yl)hydrazine-1-carboxylate (5l). Diacel Chiralcel OD-H, *n*-Hexane:*i*-PrOH = 80:20, flow = 1.0 mL/min, 25 °C, λ = 234 nm, t_R(major) = 10.0 min, t_R(minor) = 13.6 min, e.r. = 4.5:95.5

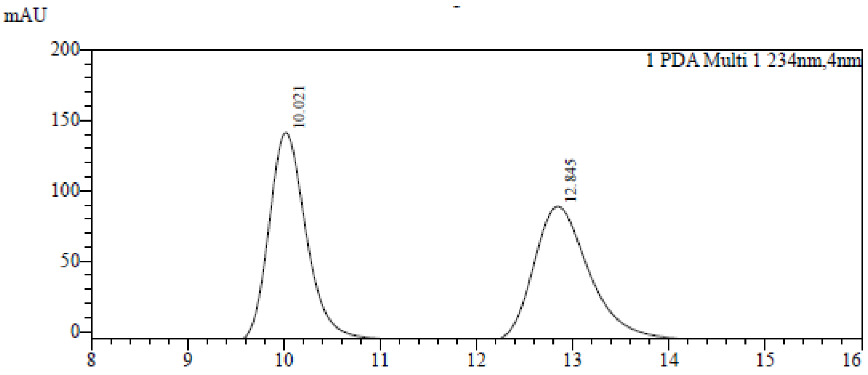

Peak Table

PDA Ch1 234nm

Peak#	Ret. Time	Peak End	Height	Area	Area%
1	10.021	11.573	148347	4113414	50.329
2	12.845	16.192	96124	4059662	49.671
Total			244471	8173076	100.000

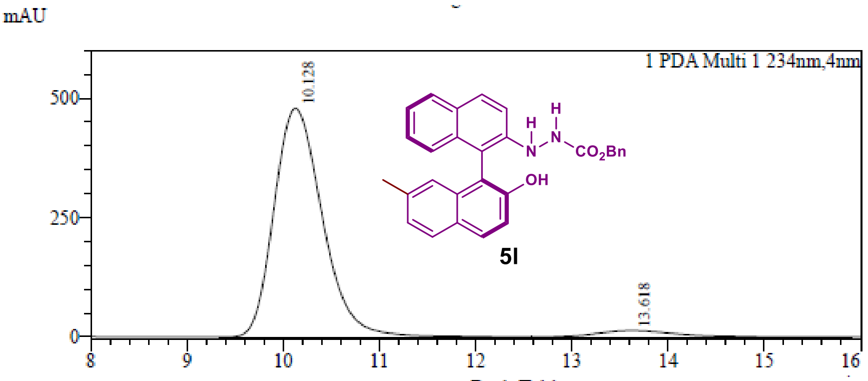

Peak Table

PDA Ch1 234nm

Peak#	Ret. Time	Peak End	Height	Area	Area%
1	10.128	12.576	478228	16748271	95.610
2	13.618	15.904	13333	768973	4.390
Total			491561	17517244	100.000

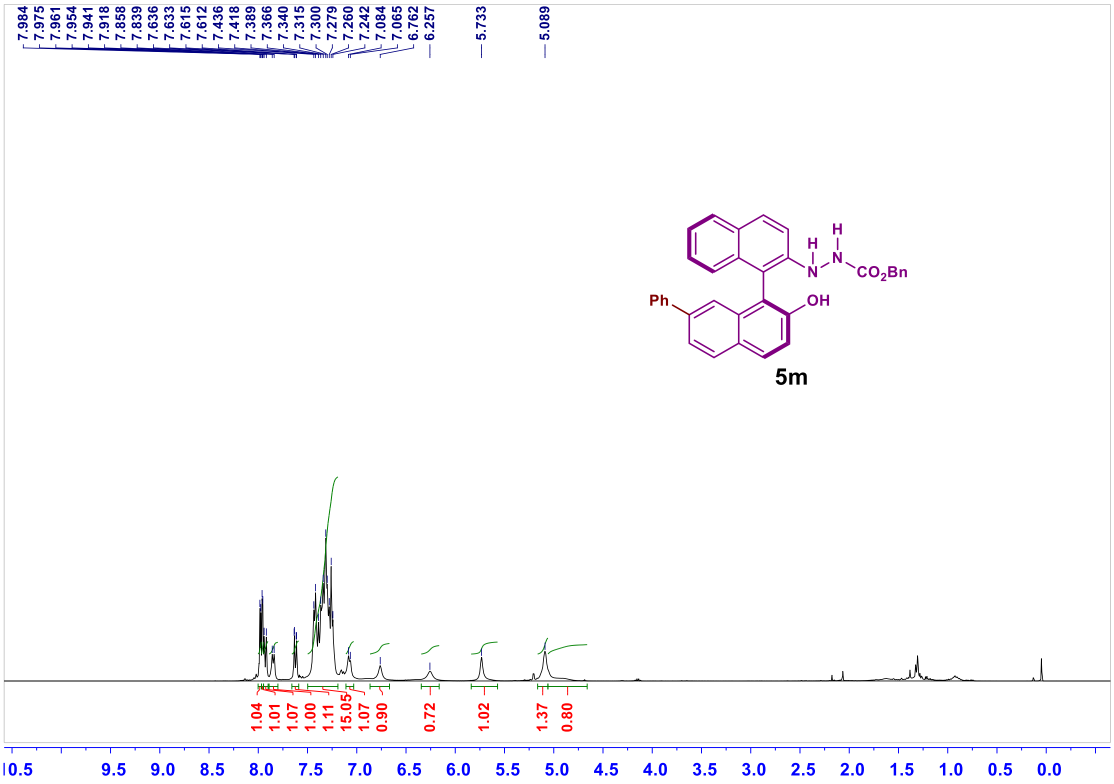

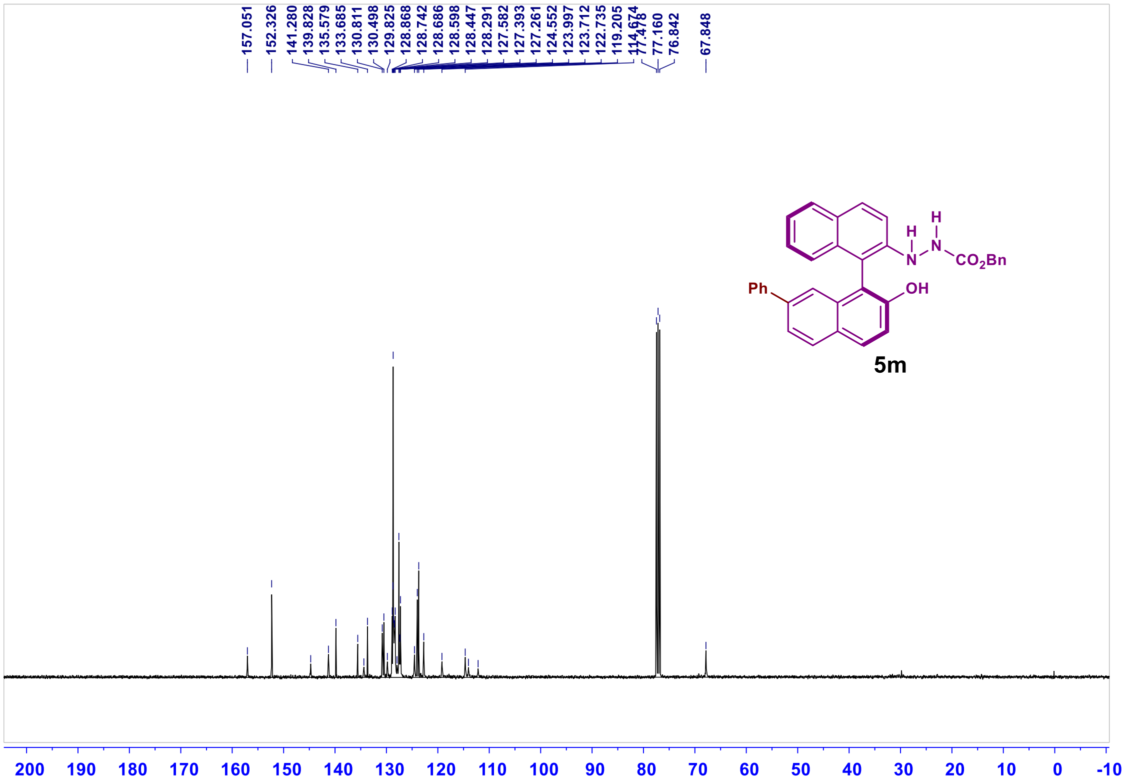

Supplementary Figure 114. ^{1}H and ^{13}C NMR spectra of 5m

Supplementary Figure 115. HPLC spectra of (*S*)-benzyl 2-(2'-hydroxy-7'-phenyl-[1,1'-binaphthalen]-2-yl)hydrazine-1-carboxylate (5m). Diacel Chiralpak AD-H, *n*-Hexane:*i*-PrOH = 80:20, flow = 1.0 mL/min, 25 °C, λ = 242 nm, t_R(major) = 11.7 min, t_R(minor) = 10.3 min, e.r. = 5:95

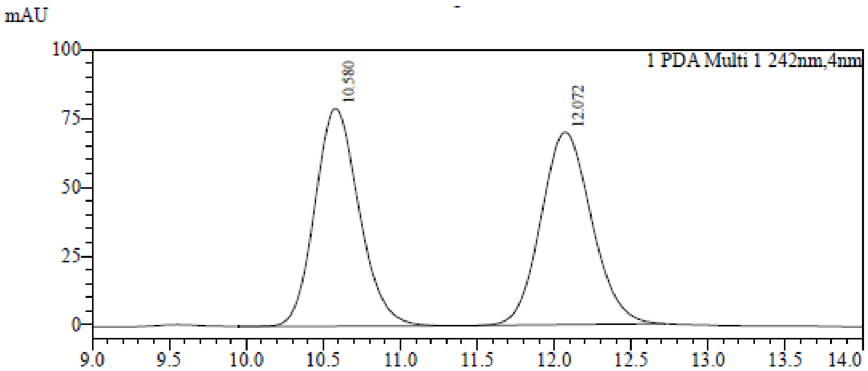

Peak Table

PDA Ch1 242nm

Peak#	Ret. Time	Peak End	Height	Area	Area%
1	10.580	11.445	79196	1588236	49.722
2	12.072	12.715	69962	1605974	50.278
Total			149158	3194210	100.000

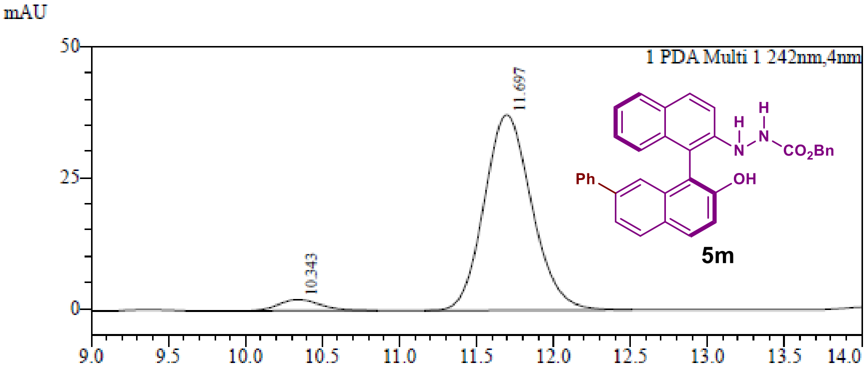

Peak Table

PDA Ch1 242nm

Peak#	Ret. Time	Peak End	Height	Area	Area%
1	10.343	10.859	2141	40166	4.765
2	11.697	12.512	37258	802779	95.235
Total			39399	842945	100.000

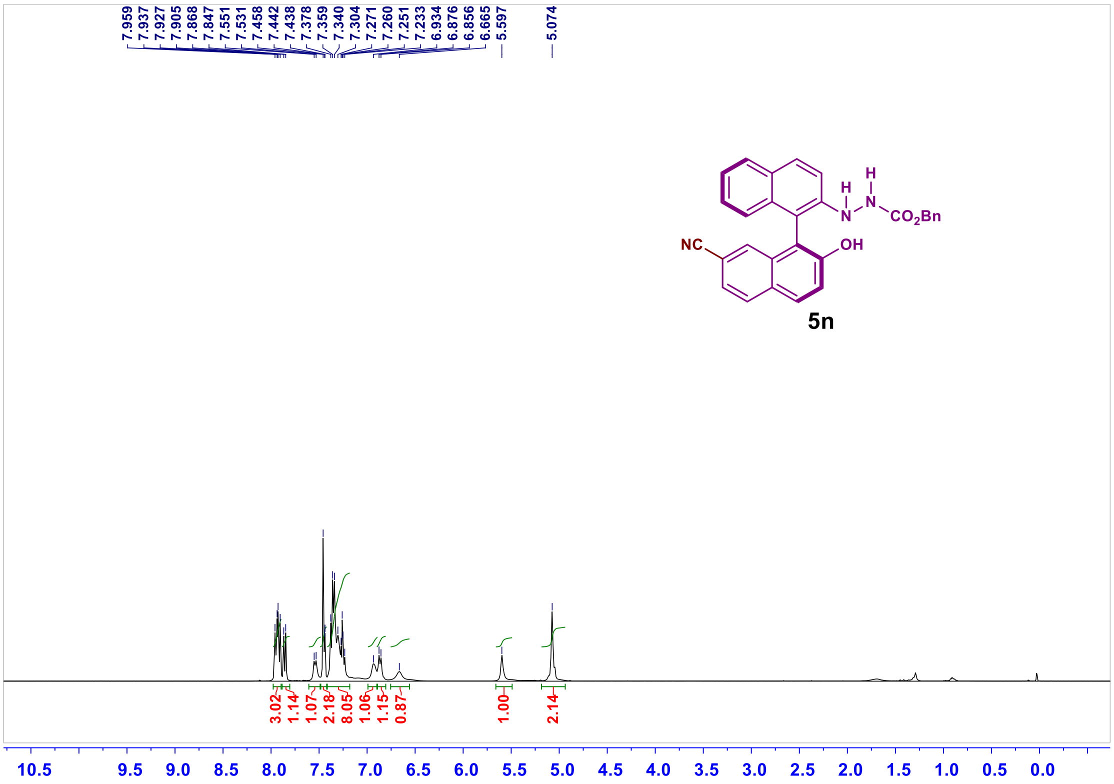

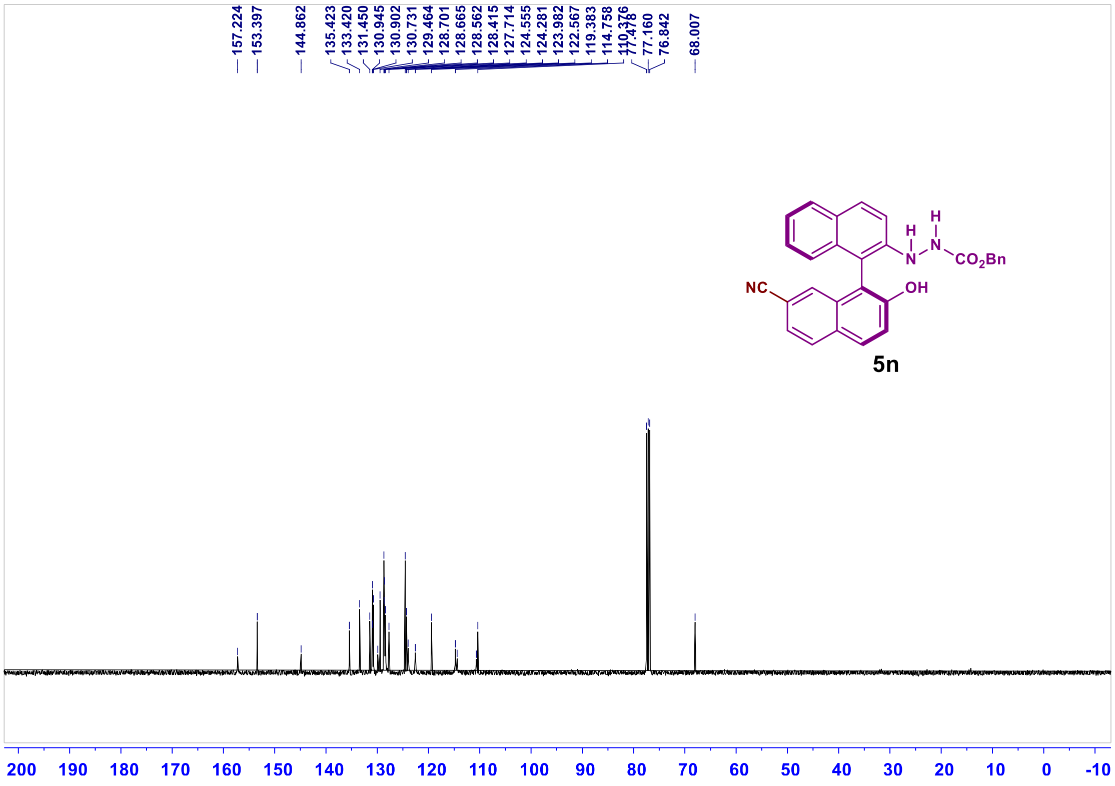

Supplementary Figure 116. ^{1}H and ^{13}C NMR spectra of 5n

Supplementary Figure 117. HPLC spectra of (*S*)-benzyl 2-(7'-cyano-2'-hydroxy-[1,1'-binaphthalen]-2-yl)hydrazine-1-carboxylate (5n). Diacel Chiralpak AD-H, *n*-Hexane:*i*-PrOH = 90:10, flow = 1.0 mL/min, 25 °C, λ = 241 nm, t_R(major) = 34.5 min, t_R(minor) = 37.5 min, e.r. = 7:93

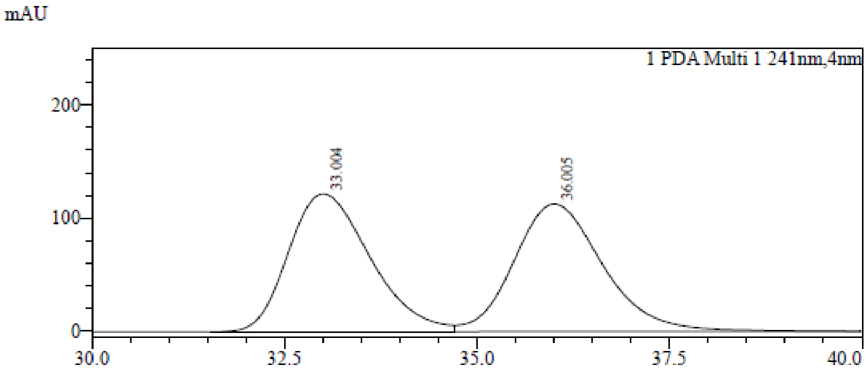

Peak Table

PDA Ch1 241nm

Peak#	Ret. Time	Peak End	Height	Area	Area%
1	33.004	34.709	121716	9007002	49.783
2	36.005	39.979	112635	9085351	50.217
Total			234350	18092353	100.000

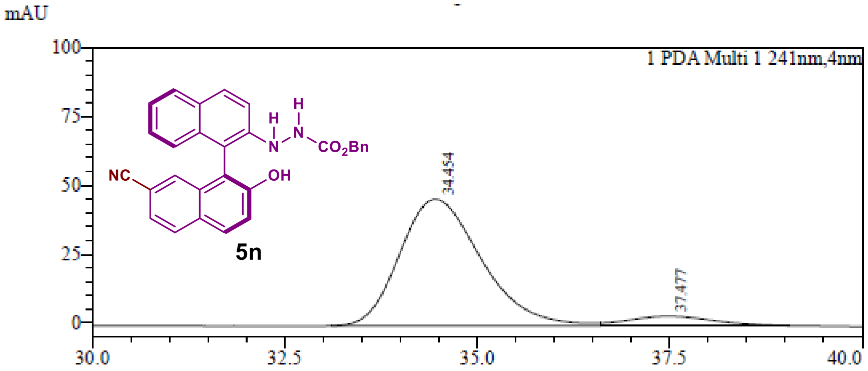

Peak Table

PDA Ch1 241nm

Peak#	Ret. Time	Peak End	Height	Area	Area%
1	34.454	36.608	46089	3403327	92.823
2	37.477	39.051	3472	263135	7.177
Total			49561	3666462	100.000

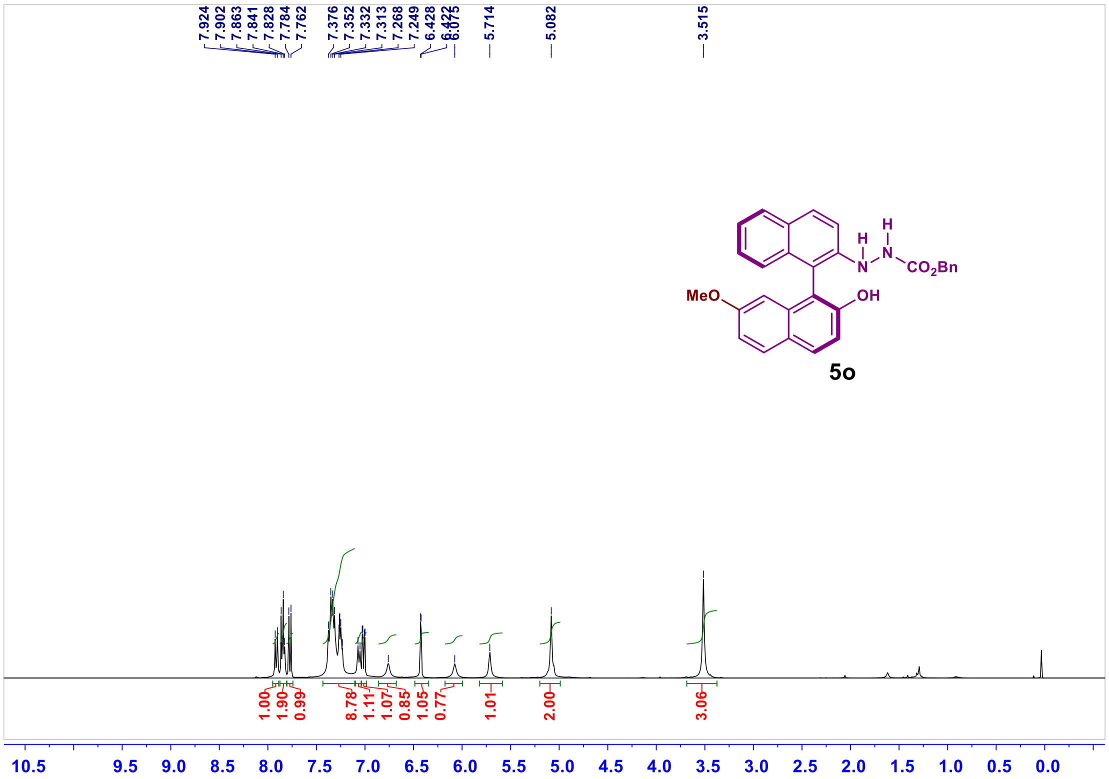

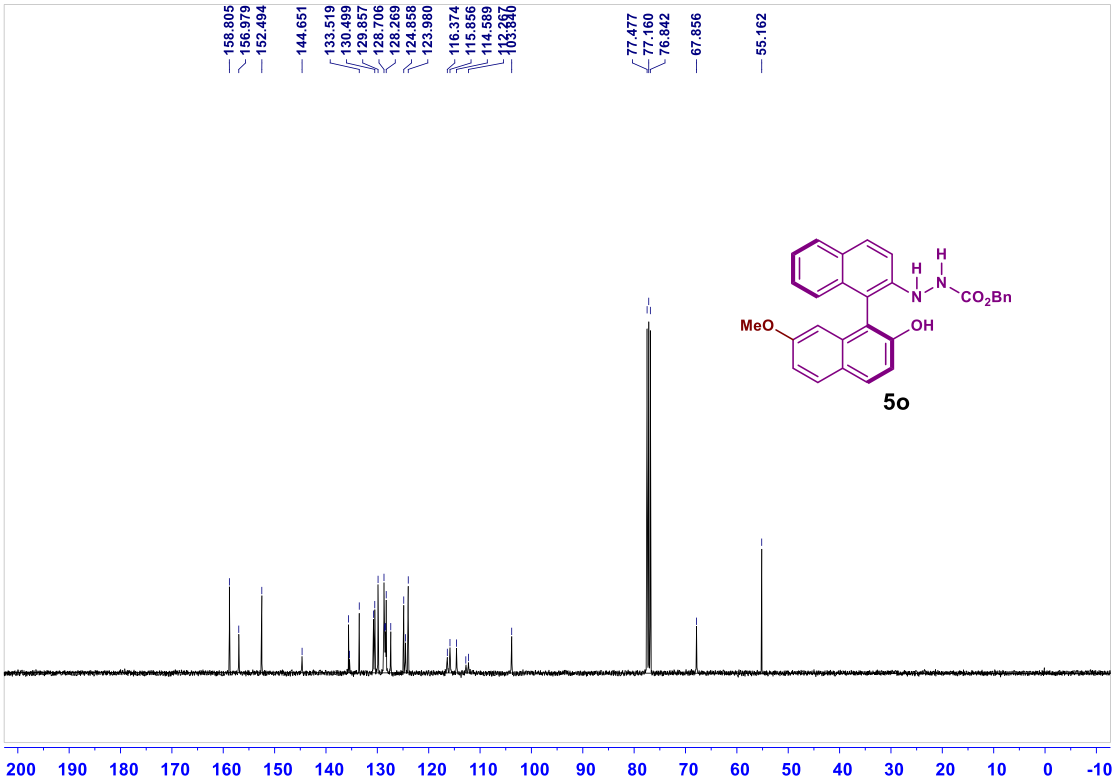

Supplementary Figure 118. ^{1}H and ^{13}C NMR spectra of 5o

Supplementary Figure 119. HPLC spectra of (*S*)-benzyl 2-(2'-hydroxy-7'-methoxy-[1,1'-binaphthalen]-2-yl)hydrazine-1-carboxylate (5o). Diacel Chiralpak AD-H, *n*-Hexane:*i*-PrOH = 80:20, flow = 1.0 mL/min, 25 °C, λ = 237 nm, t_R(major) = 13.3 min, t_R(minor) = 16.3 min, e.r. = 5:95

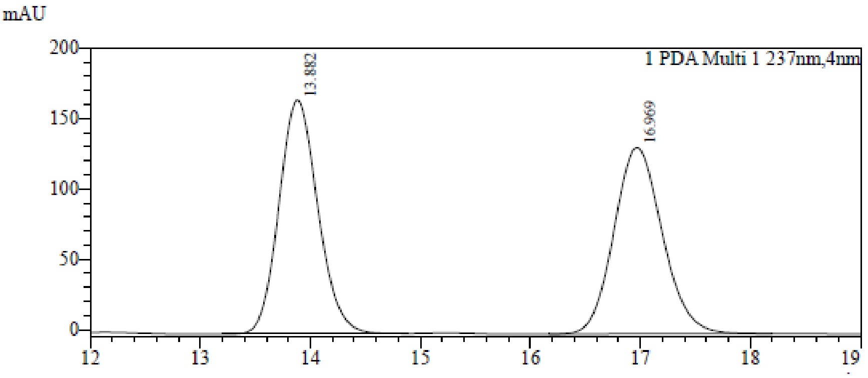

Peak Table

PDA Ch1 237nm

Peak#	Ret. Time	Peak End	Height	Area	Area%
1	13.882	14.891	166419	4117761	50.322
2	16.969	18.197	132511	4065098	49.678
Total			298930	8182860	100.000

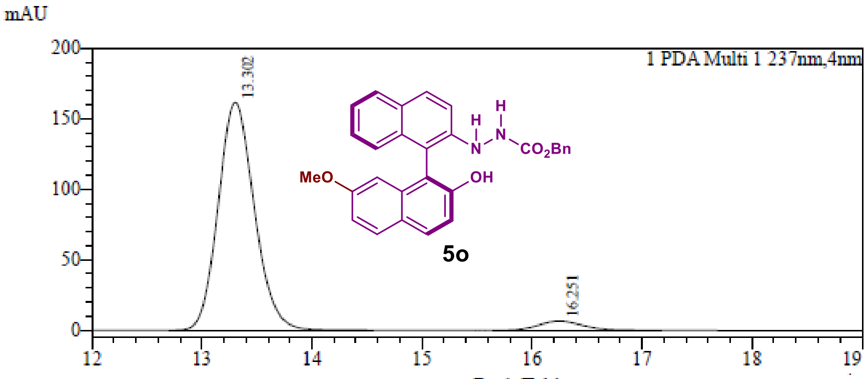

Peak Table

PDA Ch1 237nm

Peak#	Ret. Time	Peak End	Height	Area	Area%
1	13.302	14.549	161517	3703205	95.091
2	16.251	17.173	6647	191158	4.909
Total			168164	3894363	100.000

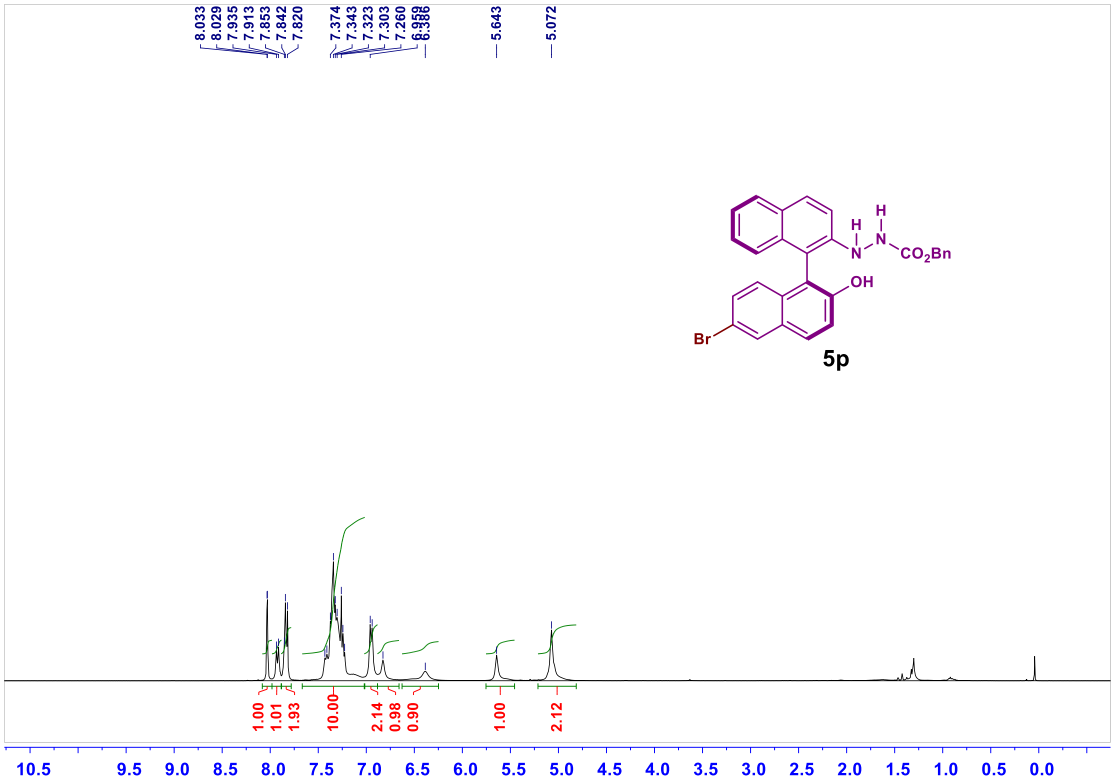

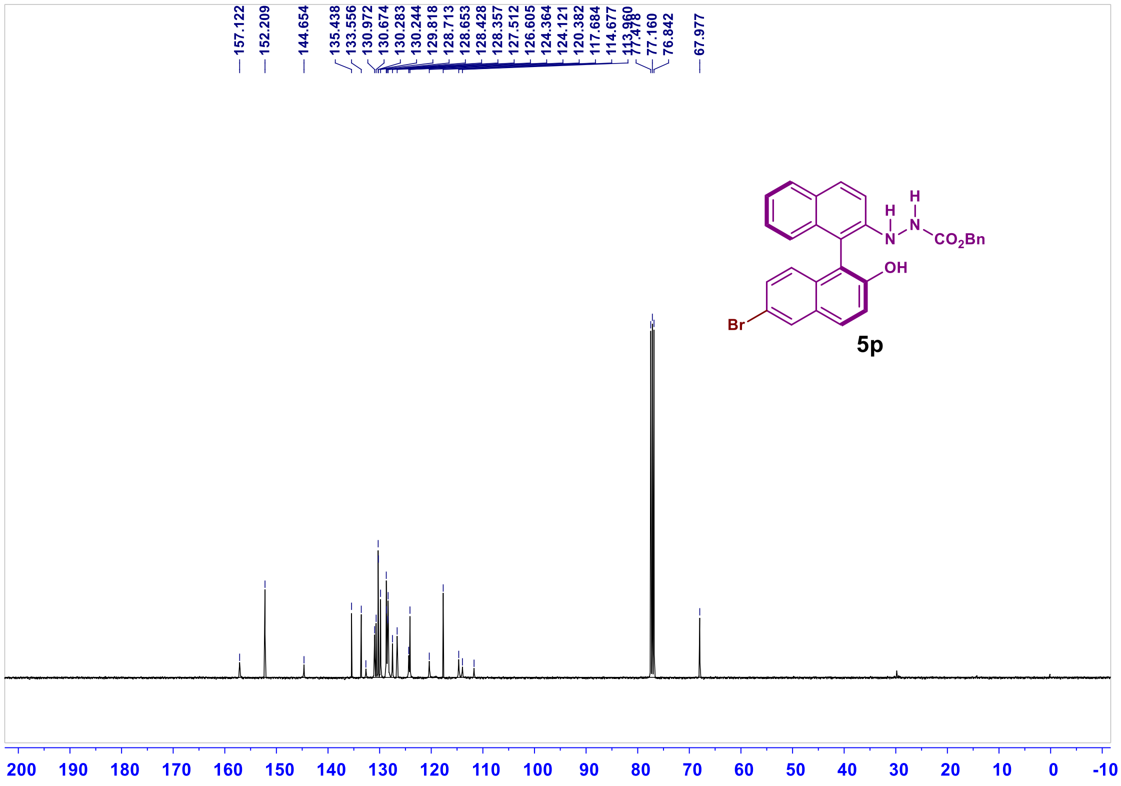

Supplementary Figure 120. ^{1}H and ^{13}C NMR spectra of 5p

Supplementary Figure 121. HPLC spectra of (*S*)-benzyl 2-(6'-bromo-2'-hydroxy-[1,1'-binaphthalen]-2-yl)hydrazine-1-carboxylate (5p). Diacel Chiralpak AD-H, *n*-Hexane:*i*-PrOH = 80:20, flow = 1.0 mL/min, 25 °C, λ = 236 nm, t_R(major) = 15.2 min, t_R(minor) = 12.8 min, e.r. = 5:95

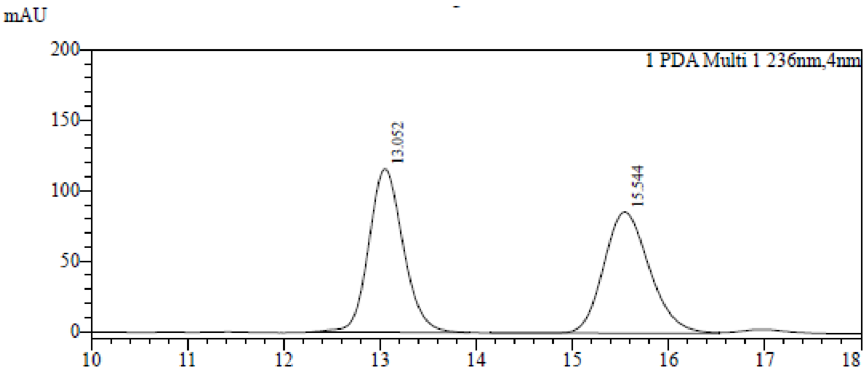

Peak Table

PDA Ch1 236nm

Peak#	Ret. Time	Peak End	Height	Area	Area%
1	13.052	13.931	116030	2896361	50.357
2	15.544	16.533	85985	2855280	49.643
Total			202015	5751642	100.000

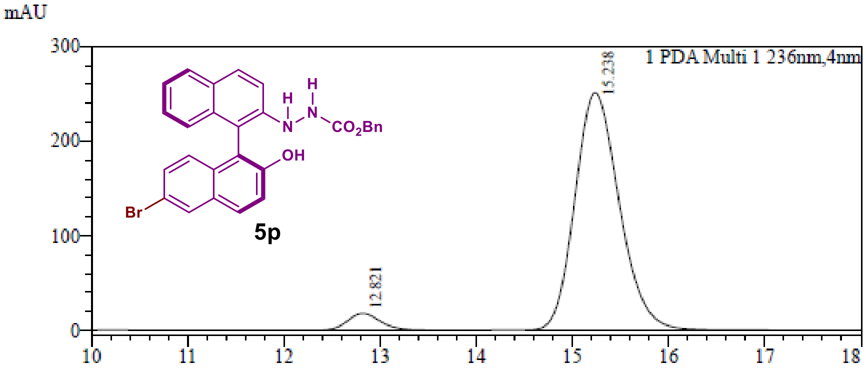

Peak Table

PDA Ch1 236nm

Peak#	Ret. Time	Peak End	Height	Area	Area%
1	12.821	13.696	17856	425302	5.011
2	15.238	17.099	250892	8061963	94.989
Total			268748	8487266	100.000

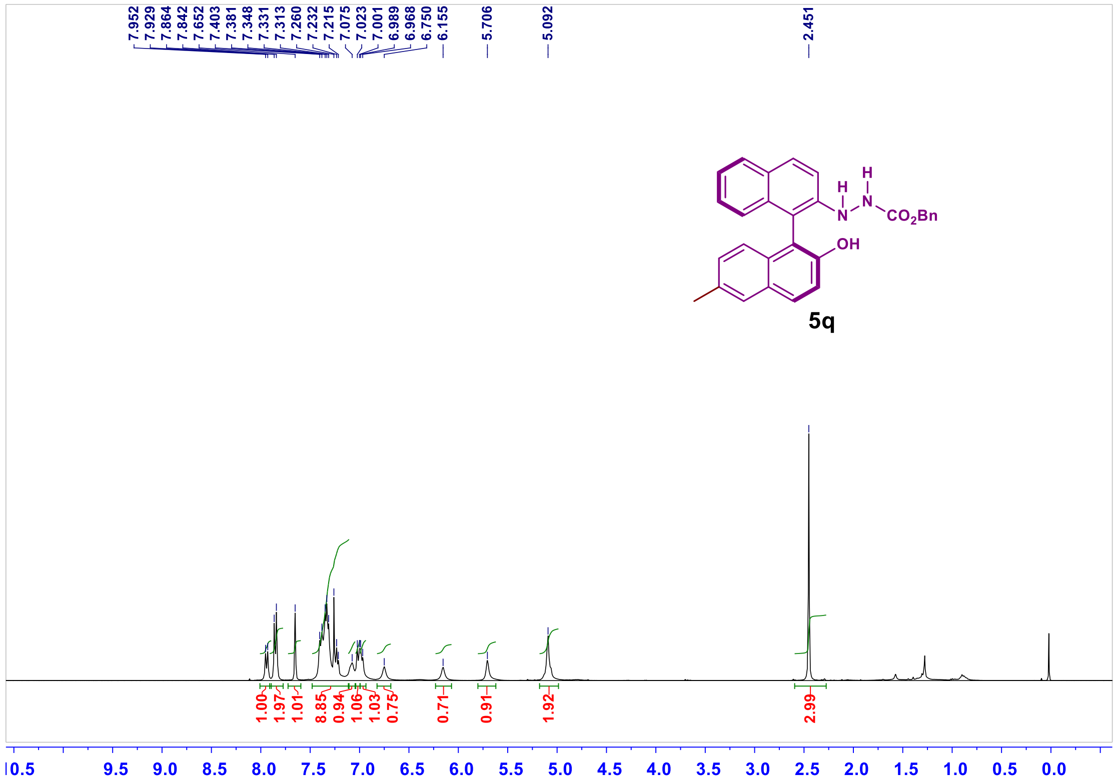

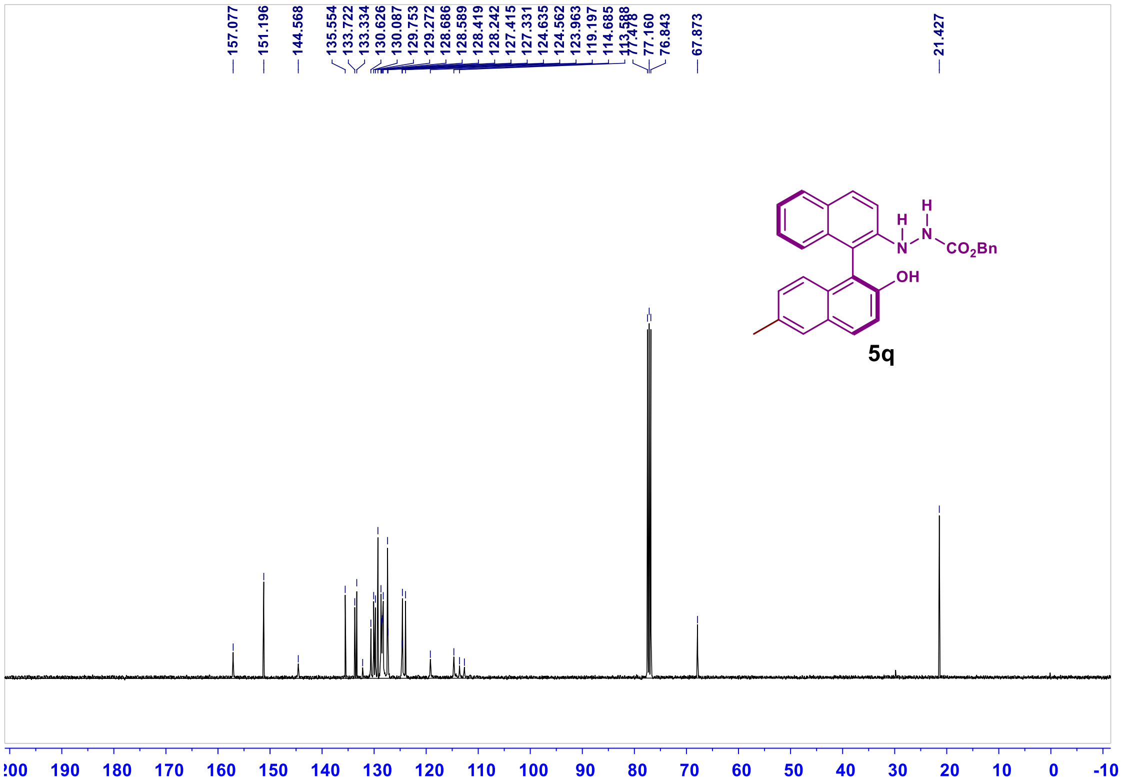

Supplementary Figure 122. ^{1}H and ^{13}C NMR spectra of 5q

Supplementary Figure 123. HPLC spectra of (*S*)-benzyl 2-(2'-hydroxy-6'-methyl-[1,1'-binaphthalen]-2-yl)hydrazine-1-carboxylate (5q). Diacel Chiralpak AD-H, *n*-Hexane:*i*-PrOH = 80:20, flow = 1.0 mL/min, 25 °C, λ = 231 nm, t_R(major) = 15.2 min, t_R(minor) = 13.5 min, e.r. = 5:95

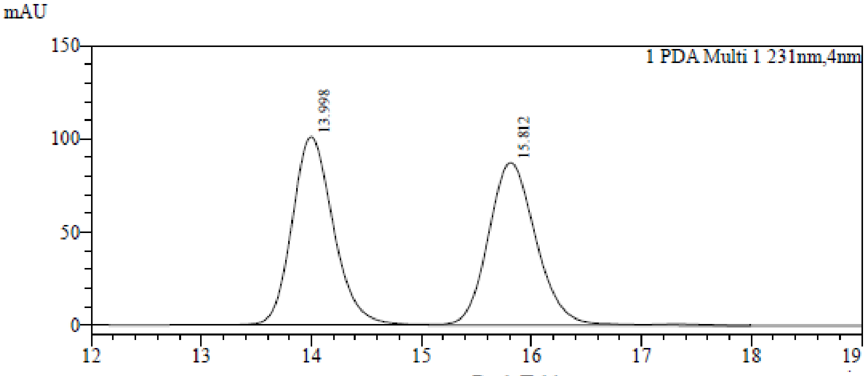

Peak Table

PDA Ch1 231nm

Peak#	Ret. Time	Peak End	Height	Area	Area%
1	13.998	15.061	101335	2660024	49.964
2	15.812	17.995	87472	2663844	50.036
Total			188807	5323868	100.000

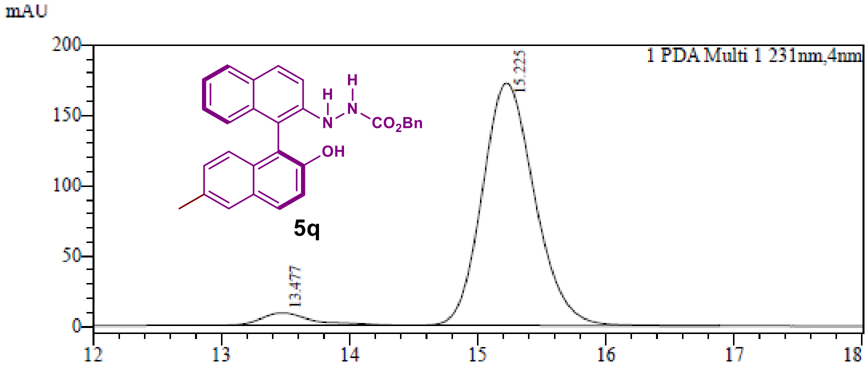

Peak Table

PDA Ch1 231nm

Peak#	Ret. Time	Peak End	Height	Area	Area%
1	13.477	14.464	9052	250560	4.864
2	15.225	16.885	172314	4900394	95.136
Total			181366	5150953	100.000

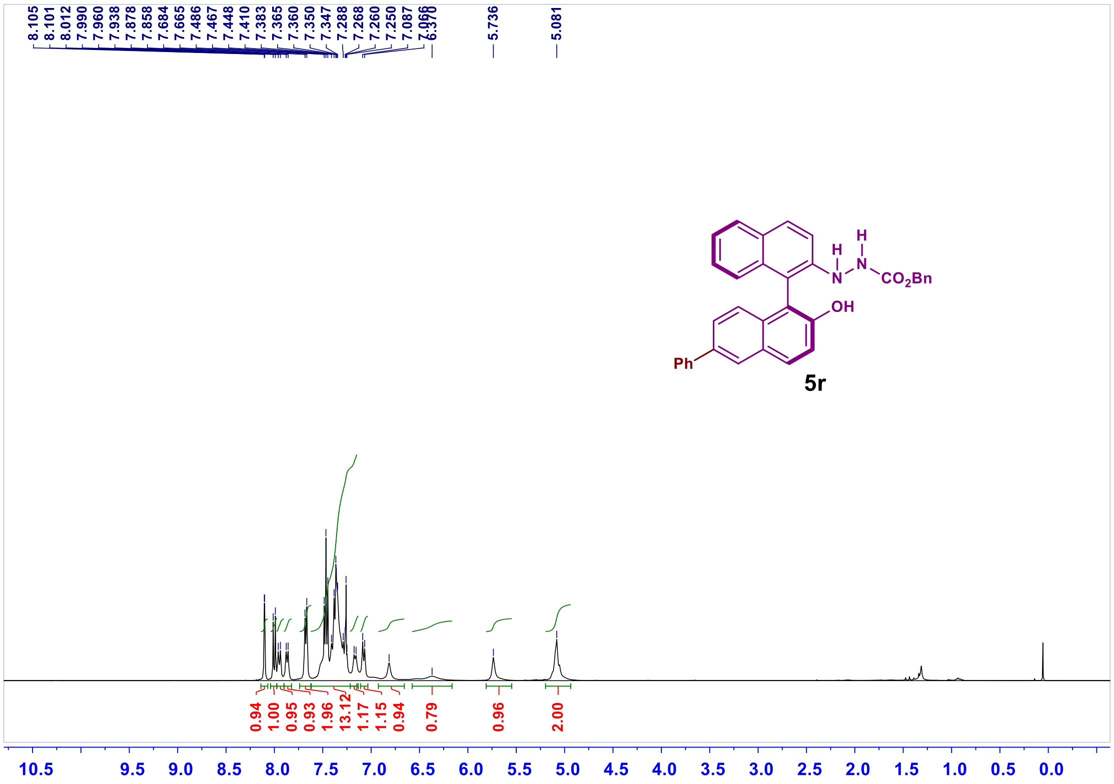

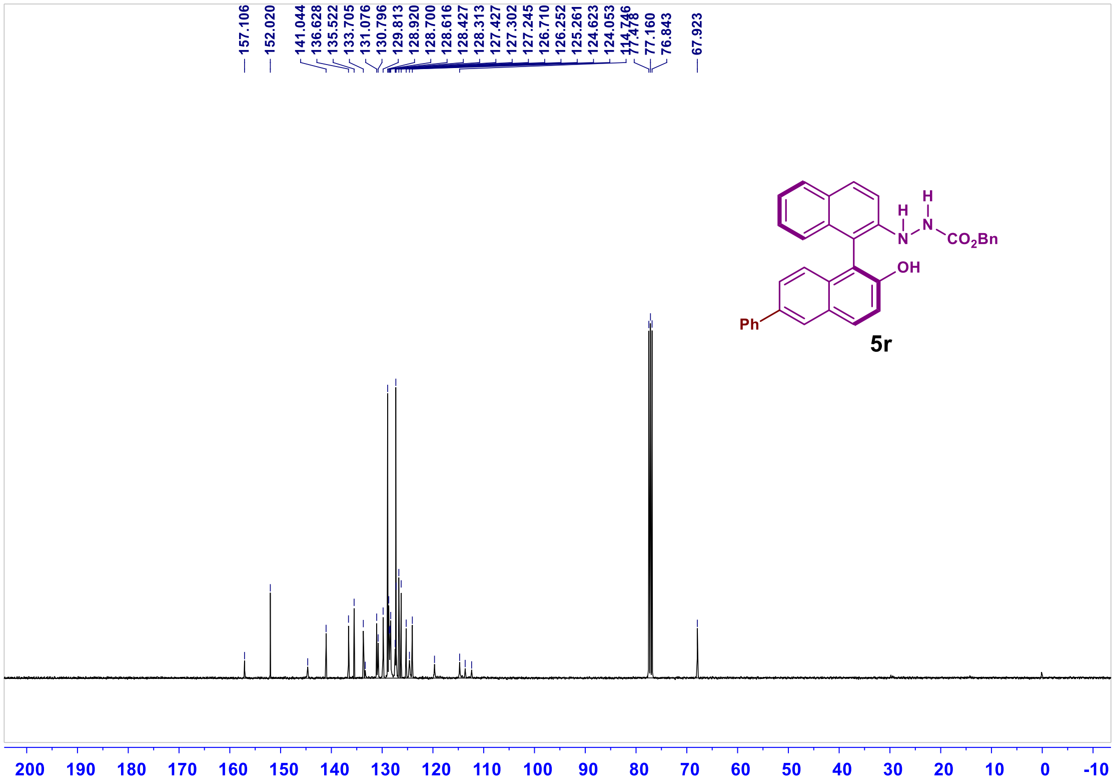

Supplementary Figure 124. ^{1}H **and** ^{13}C **NMR spectra of 5r**

Supplementary Figure 125. HPLC spectra of (*S*)-benzyl 2-(2'-hydroxy-6'-phenyl-[1,1'-binaphthalen]-2-yl)hydrazine-1-carboxylate (5r). Diacel Chiralpak AD-H, *n*-Hexane:*i*-PrOH = 80:20, flow = 1.0 mL/min, 25 °C, λ = 266 nm, t_R(major) = 18.2 min, t_R(minor) = 16.7 min, e.r. = 4:96

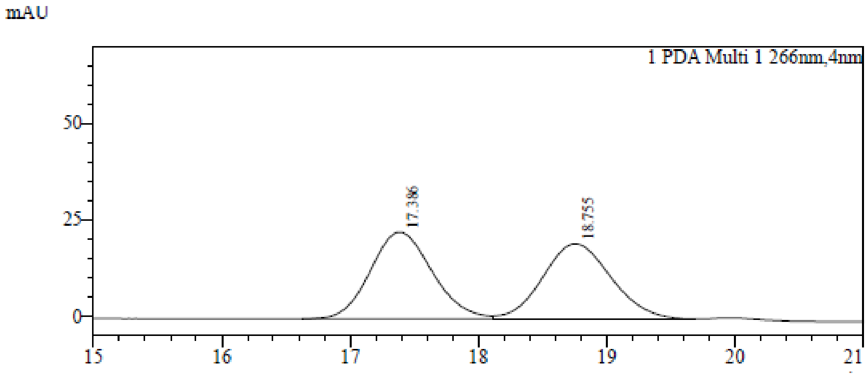

Peak Table

PDA Ch1 266nm

Peak#	Ret. Time	Peak End	Height	Area	Area%
1	17.386	18.112	22543	756575	51.040
2	18.755	19.691	19540	725751	48.960
Total			42083	1482326	100.000

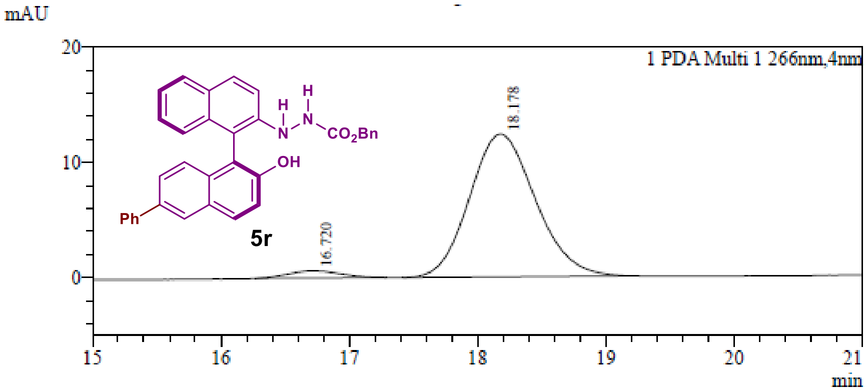

Peak Table

PDA Ch1 266nm

Peak#	Ret. Time	Peak End	Height	Area	Area%
1	16.720	17.291	611	17574	3.814
2	18.178	19.232	12373	443151	96.186
Total			12984	460725	100.000

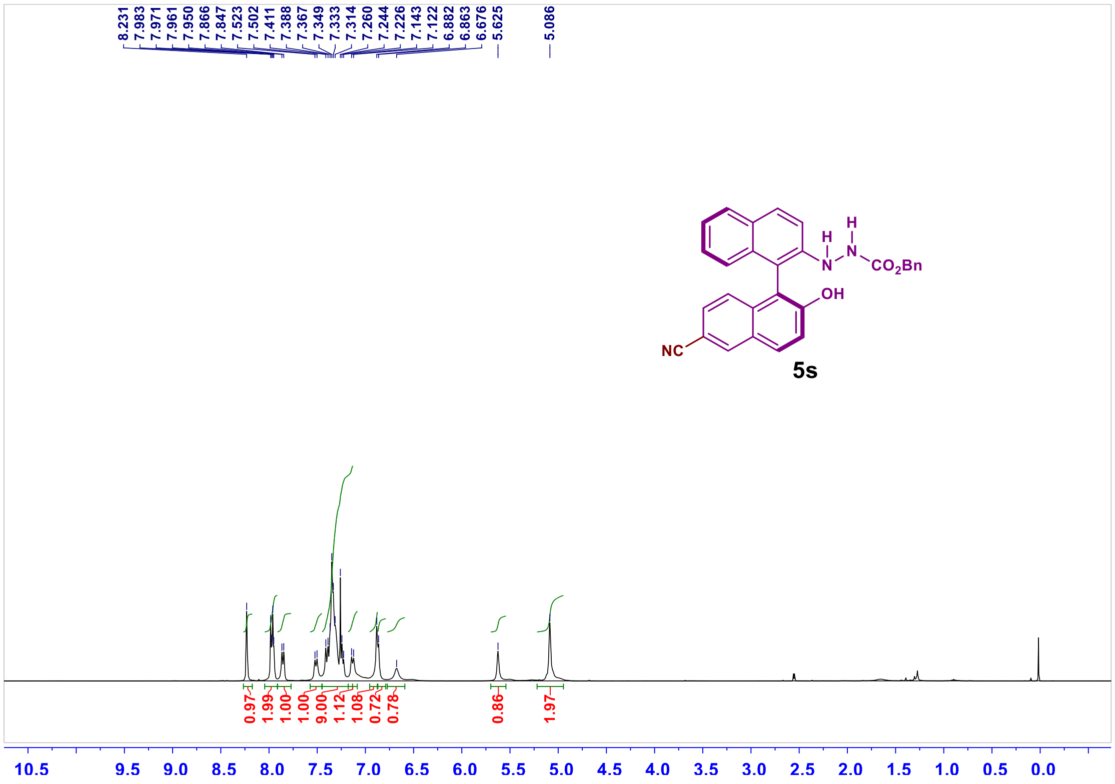

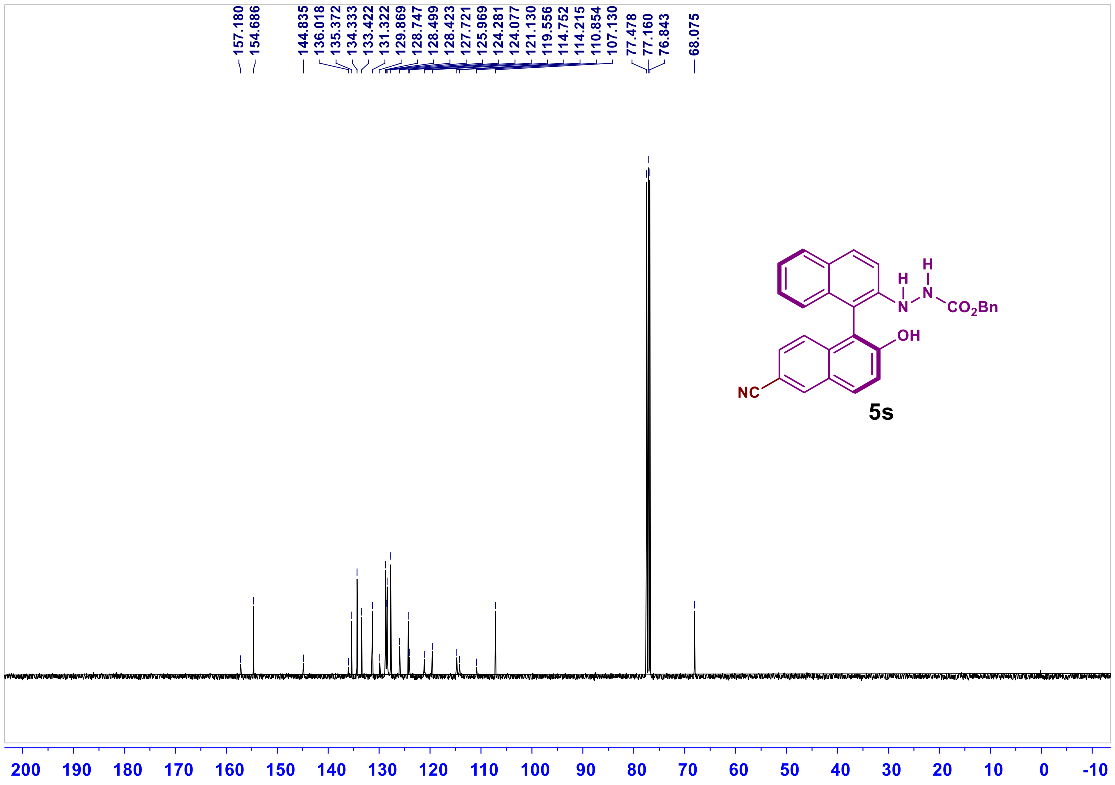

Supplementary Figure 126. ^{1}H and ^{13}C NMR spectra of 5s

Supplementary Figure 127. HPLC spectra of (*S*)-benzyl 2-(6'-cyano-2'-hydroxy-[1,1'-binaphthalen]-2-yl)hydrazine-1-carboxylate (5s). Diacel Chiralcel OD-H, *n*-Hexane:*i*-PrOH = 80:20, flow = 1.0 mL/min, 25 °C, λ = 239 nm, t_R(major) = 16.5 min, t_R(minor) = 21.0 min, e.r. = 5:95

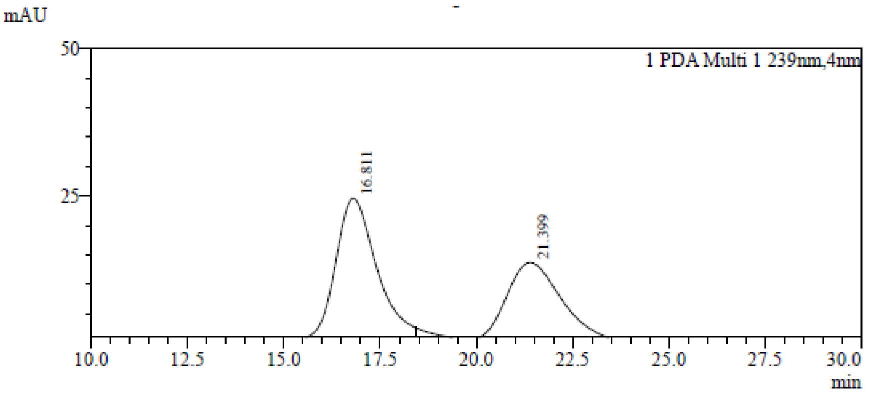

Peak Table

PDA Ch1 239nm

Peak#	Ret. Time	Peak End	Height	Area	Area%
1	16.811	18.421	25614	2060510	49.952
2	21.399	30.048	14941	2064489	50.048
Total			40554	4124999	100.000

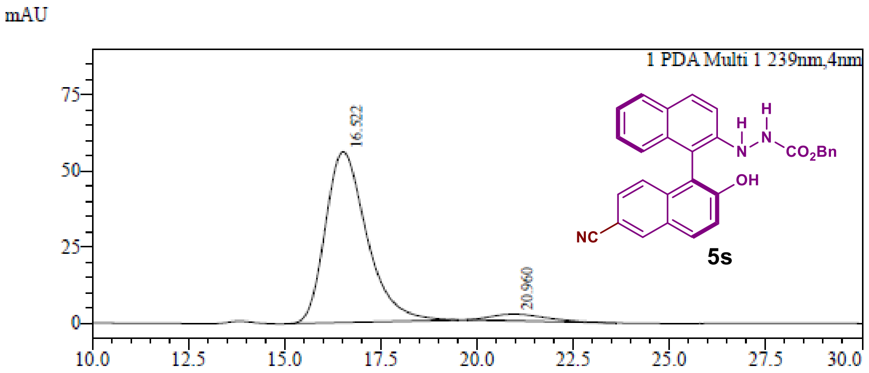

Peak Table

PDA Ch1 239nm

Peak#	Ret. Time	Peak End	Height	Area	Area%
1	16.522	19.509	56128	4226499	95.132
2	20.960	23.616	2230	216294	4.868
Total			58359	4442793	100.000

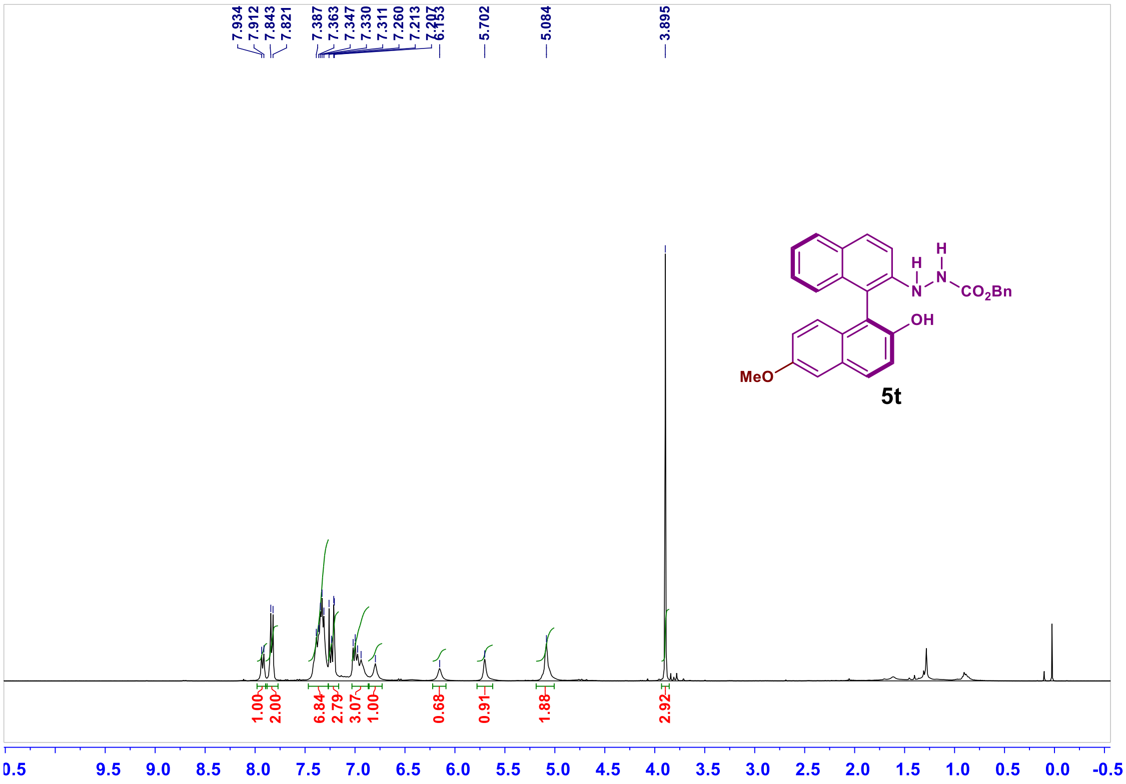

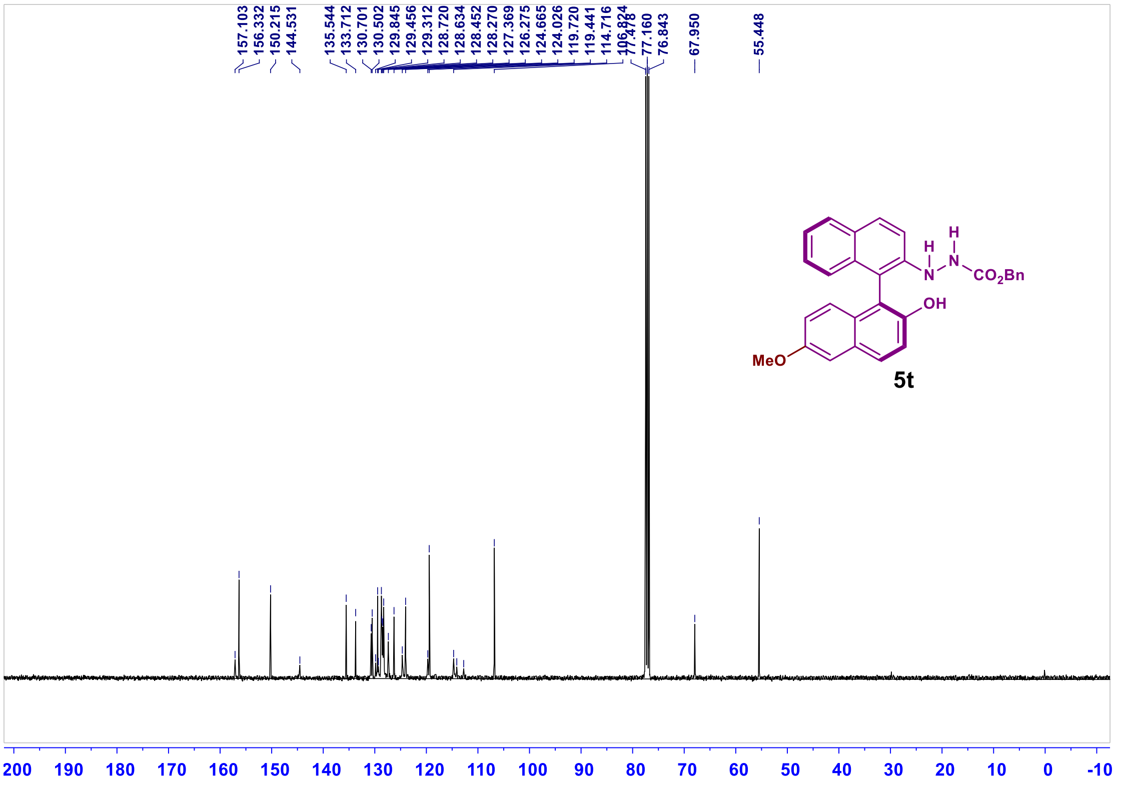

Supplementary Figure 128. ^{1}H and ^{13}C NMR spectra of 5t

Supplementary Figure 129. HPLC spectra of (*S*)-benzyl 2-(2'-hydroxy-6'-methoxy-[1,1'-binaphthalen]-2-yl)hydrazine-1-carboxylate (5t). Diacel Chiralpak AD-H, *n*-Hexane:*i*-PrOH = 70:30, flow = 1.0 mL/min, 25 °C, λ = 231 nm, t_R(major) = 16.3 min, t_R(minor) = 12.9 min, e.r. = 3.5:96.5

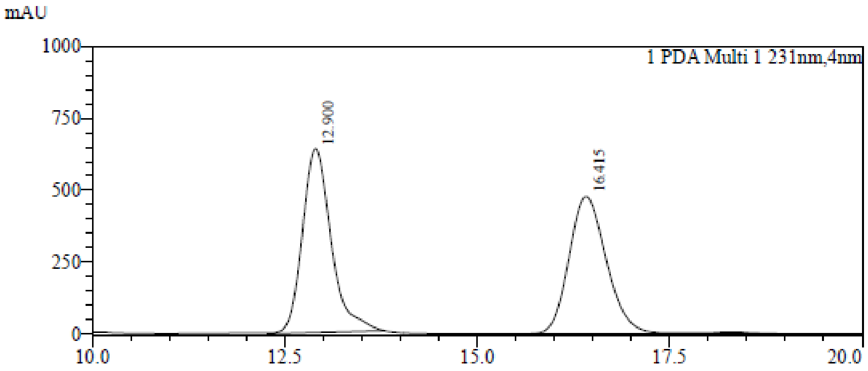

Peak Table

PDA Ch1 231nm

Peak#	Ret. Time	Peak End	Height	Area	Area%
1	12.900	13.792	640066	16687383	50.376
2	16.415	19.189	478001	16438119	49.624
Total			1118066	33125502	100.000

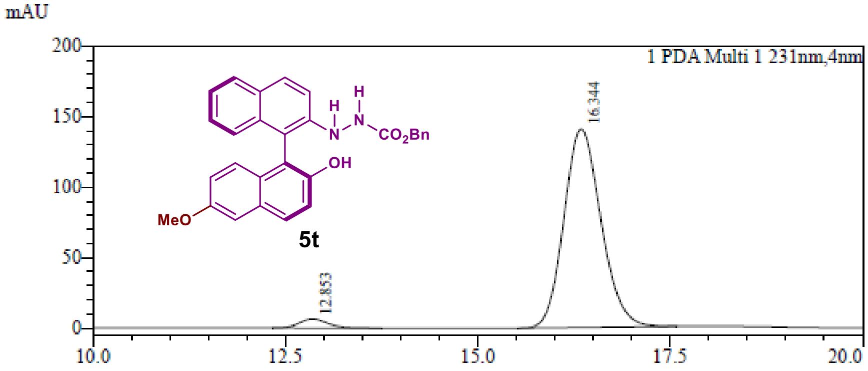

Peak Table

PDA Ch1 231nm

Peak#	Ret. Time	Peak End	Height	Area	Area%
1	12.853	13.749	6560	167073	3.412
2	16.344	17.589	140915	4730029	96.588
Total			147475	4897102	100.000

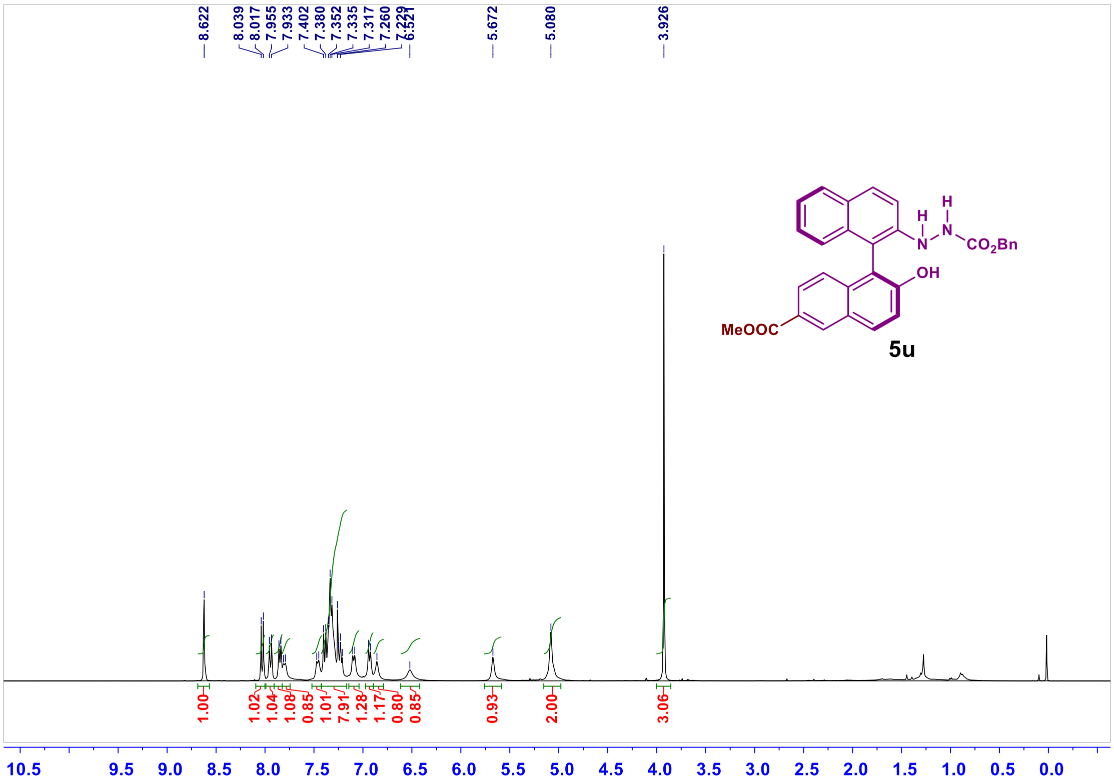

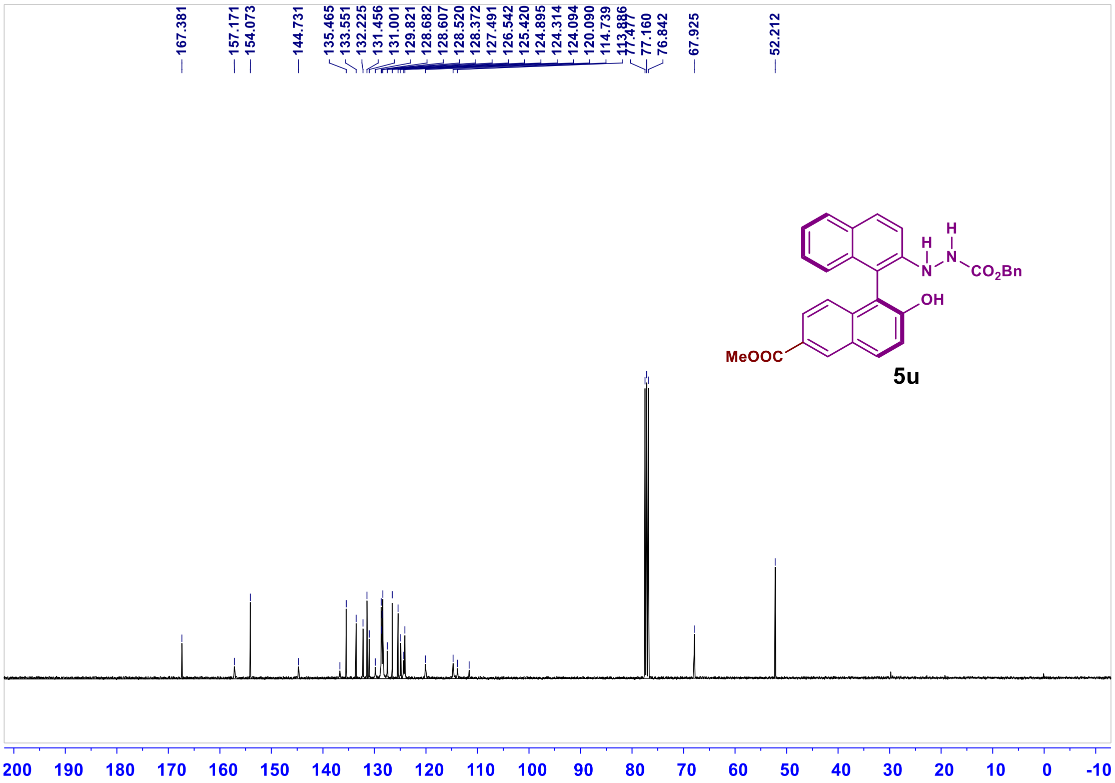

Supplementary Figure 130. ^{1}H and ^{13}C NMR spectra of 5u

Supplementary Figure 131. HPLC spectra of (*S*)-benzyl 2-(2'-hydroxy-6'-(methoxycarbonyl)-[1,1'-binaphthalen]-2-yl)hydrazine-1-carbo xylate (5u). Diacel Chiralpak AD-H, *n*-Hexane:*i*-PrOH = 80:20, flow = 1.0 mL/min, 25 °C, λ = 240 nm, t_R(major) = 21.3 min, t_R(minor) = 28.0 min, e.r. = 3.5:96.5

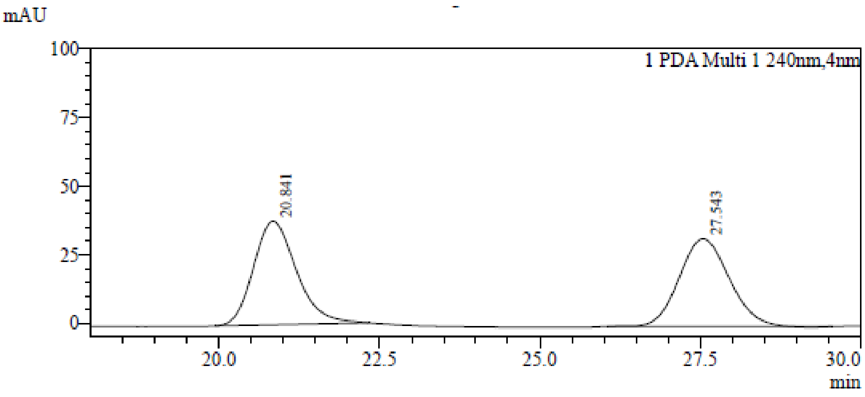

Peak Table

PDA Ch1 240nm

Peak#	Ret. Time	Peak End	Height	Area	Area%
1	20.841	22.336	37678	1760219	50.363
2	27.543	29.557	31963	1734861	49.637
Total			69642	3495080	100.000

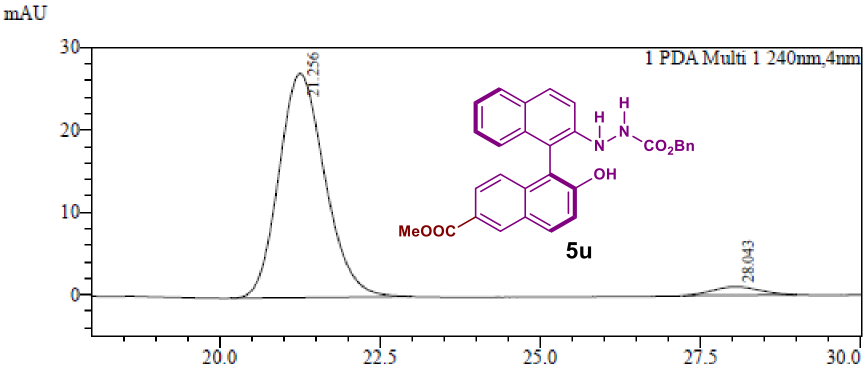

Peak Table

PDA Ch1 240nm

Peak#	Ret. Time	Peak End	Height	Area	Area%
1	21.256	22.987	27162	1358011	96.256
2	28.043	28.981	1035	52825	3.744
Total			28197	1410837	100.000

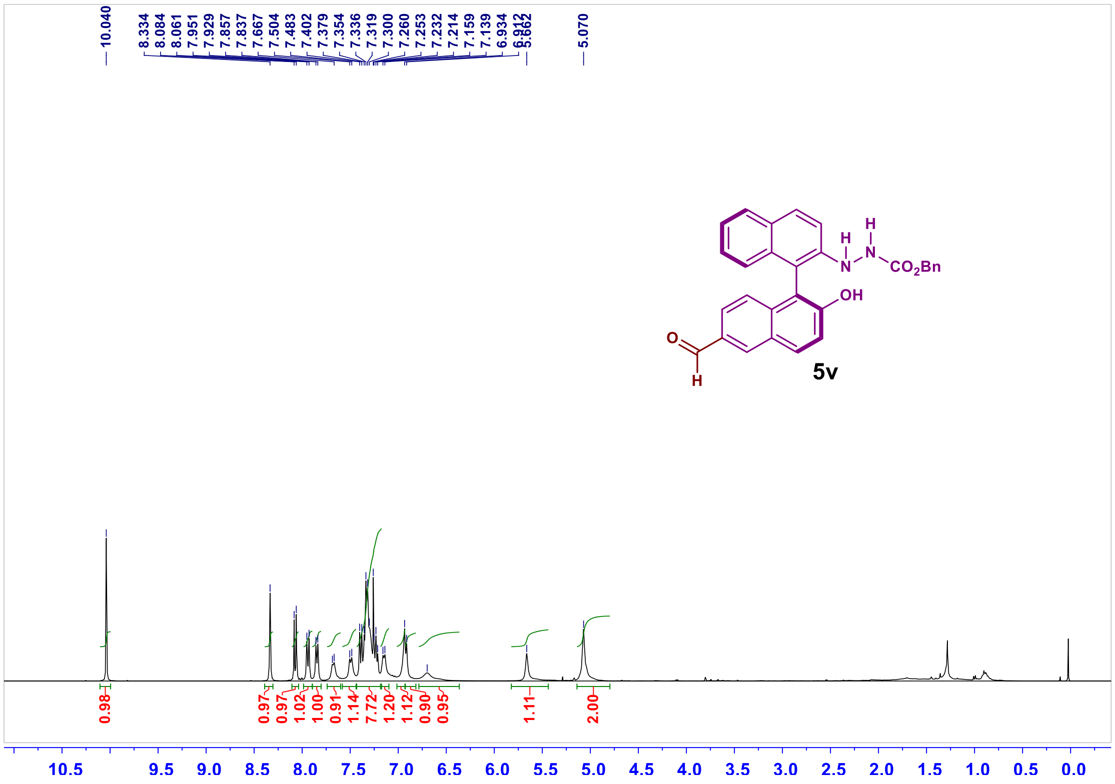

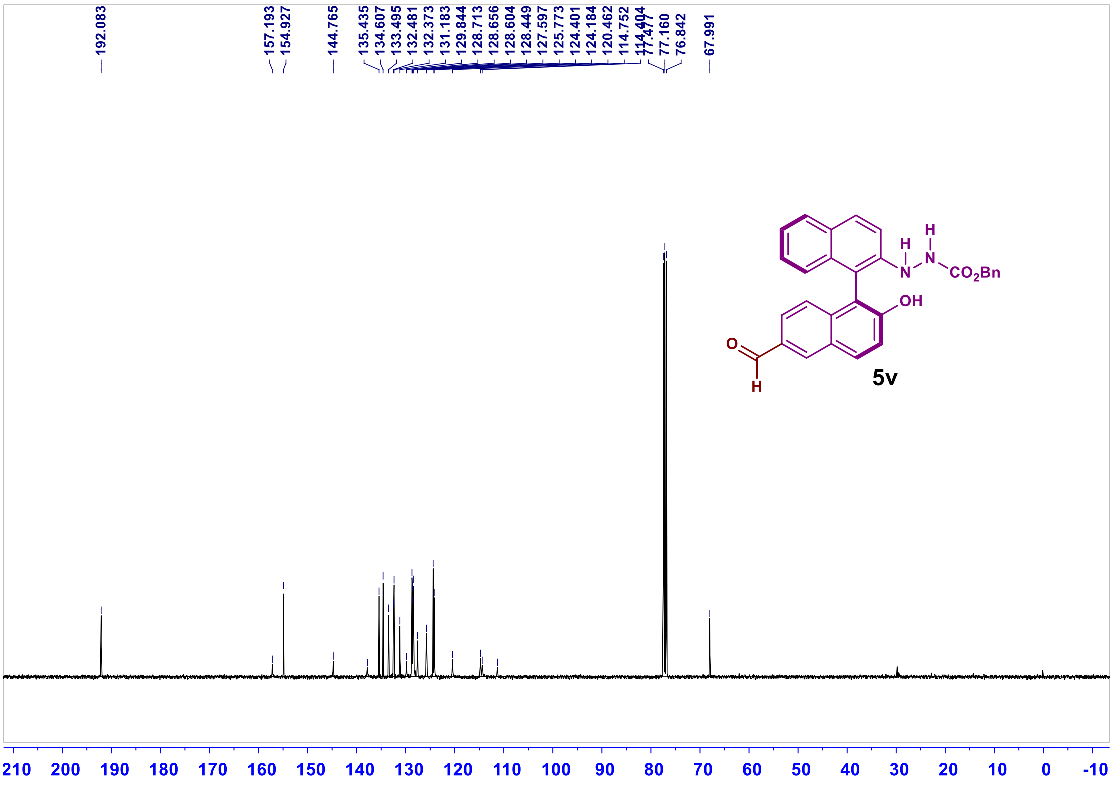

Supplementary Figure 132. ^{1}H and ^{13}C NMR spectra of 5v

Supplementary Figure 133. HPLC spectra of (*S*)-benzyl 2-(6'-formyl-2'-hydroxy-[1,1'-binaphthalen]-2-yl)hydrazine-1-carboxylate (5v). Diacel Chiralpak AD-H, *n*-Hexane:*i*-PrOH = 80:20, flow = 1.0 mL/min, 25 °C, λ = 241 nm, t_R(major) = 23.0 min, t_R(minor) = 20.8 min, e.r. = 4:96

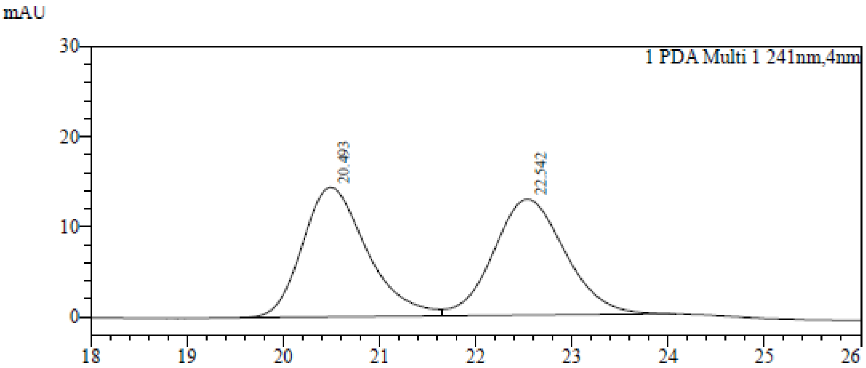

Peak Table

PDA Ch1 241nm

Peak#	Ret. Time	Peak End	Height	Area	Area%
1	20.493	21.643	14352	669527	50.072
2	22.542	24.075	12859	667600	49.928
Total			27211	1337127	100.000

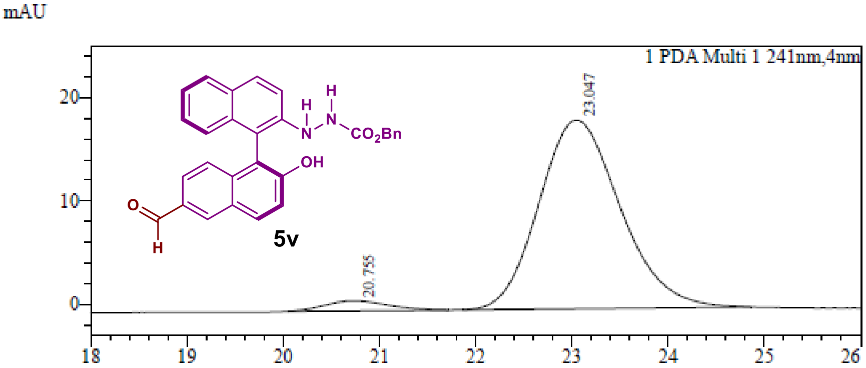

Peak Table

PDA Ch1 241nm

Peak#	Ret. Time	Peak End	Height	Area	Area%
1	20.755	21.717	971	44031	3.930
2	23.047	24.853	18293	1076283	96.070
Total			19264	1120314	100.000

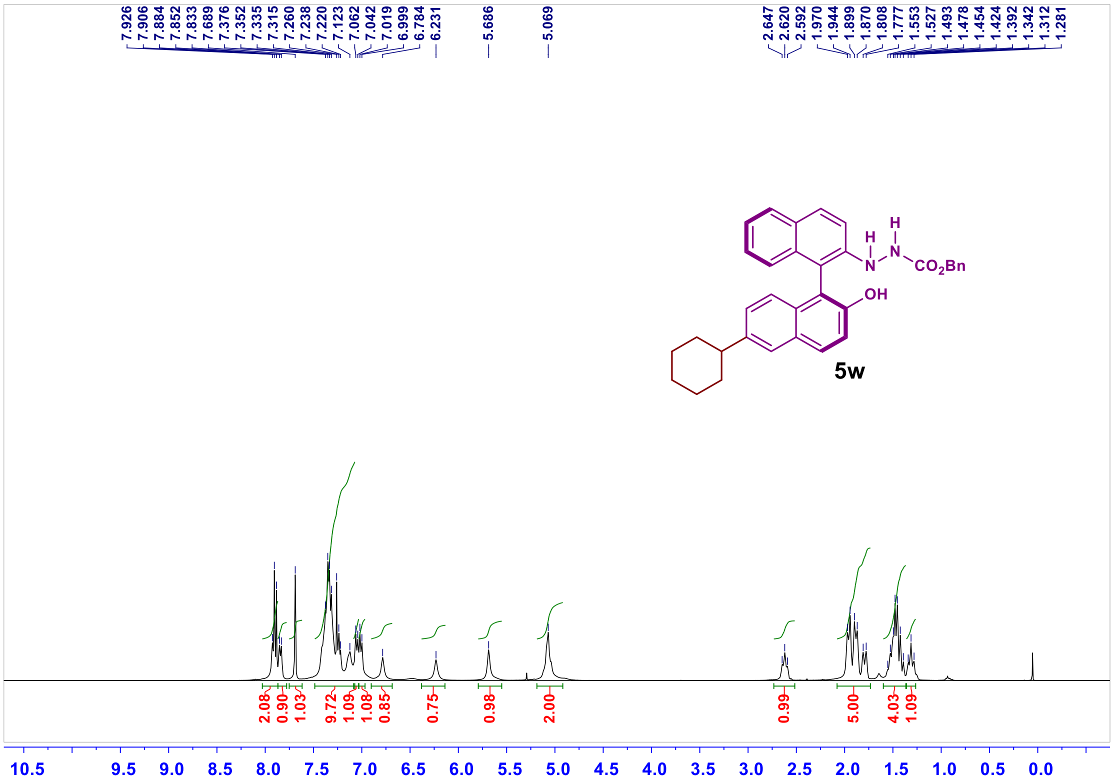

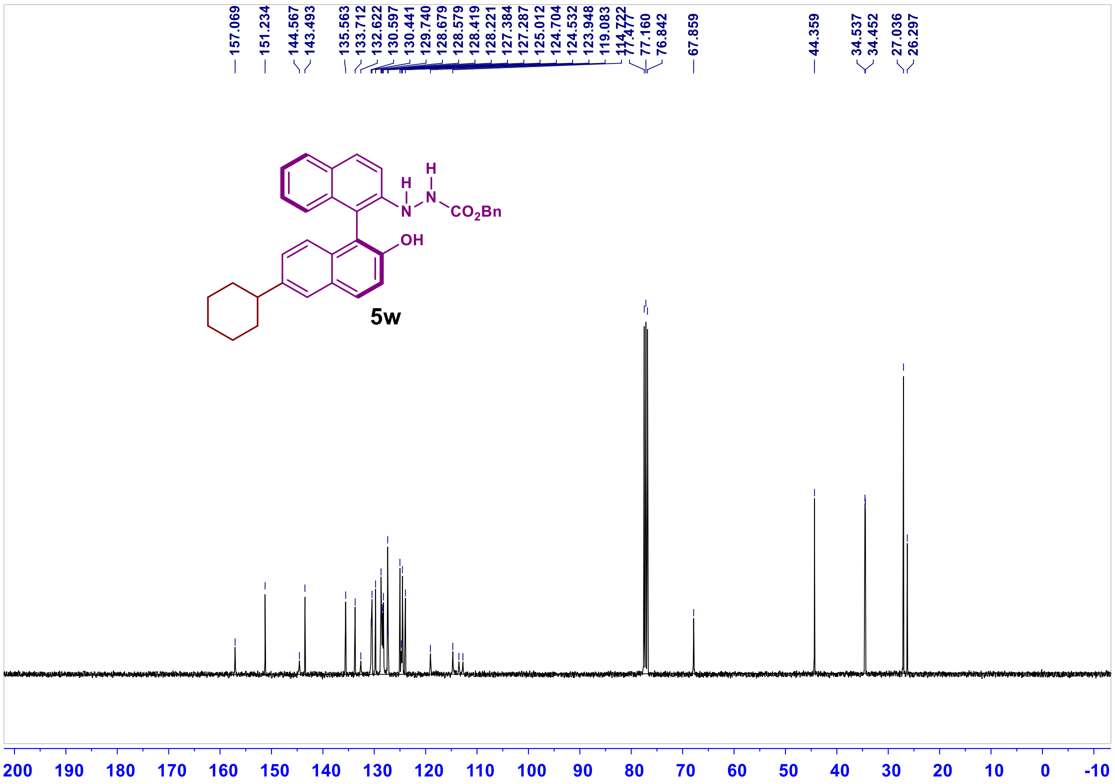

Supplementary Figure 134. ^{1}H and ^{13}C NMR spectra of 5w

Supplementary Figure 135. HPLC spectra of (*S*)-benzyl 2-(6'-cyclohexyl-2'-hydroxy-[1,1'-binaphthalen]-2-yl)hydrazine-1-carboxylate (5w). Diacel Chiralpak AD-H, *n*-Hexane:*i*-PrOH = 80:20, flow = 1.0 mL/min, 25 °C, λ = 232 nm, t_R(major) = 14.1 min, t_R(minor) = 11.9 min, e.r. = 3.5:96.5

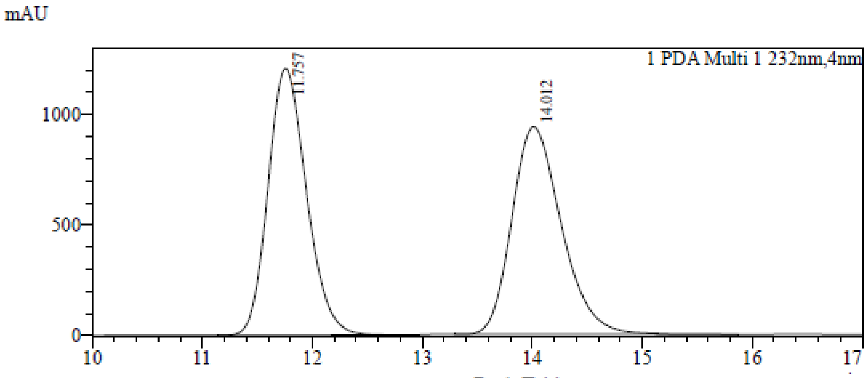

Peak Table

PDA Ch1 232nm

Peak#	Ret. Time	Peak End	Height	Area	Area%
1	11.757	12.832	1207042	29428210	49.985
2	14.012	15.872	938239	29446326	50.015
Total			2145281	58874537	100.000

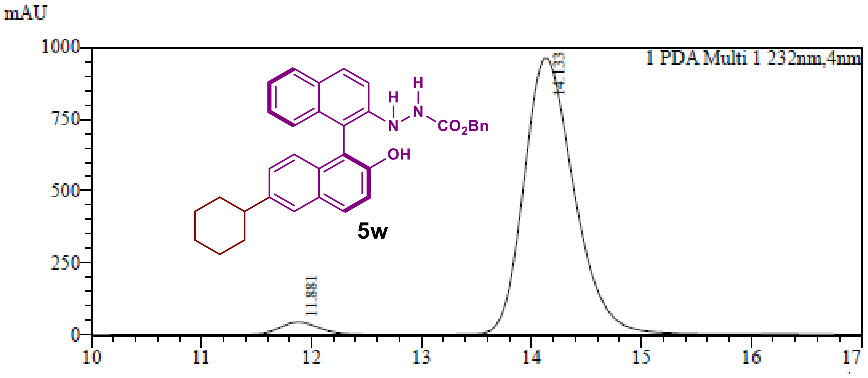

Peak Table

PDA Ch1 232nm

Peak#	Ret. Time	Peak End	Height	Area	Area%
1	11.881	12.672	43046	1058077	3.352
2	14.133	16.427	962799	30510823	96.648
Total			1005845	31568900	100.000

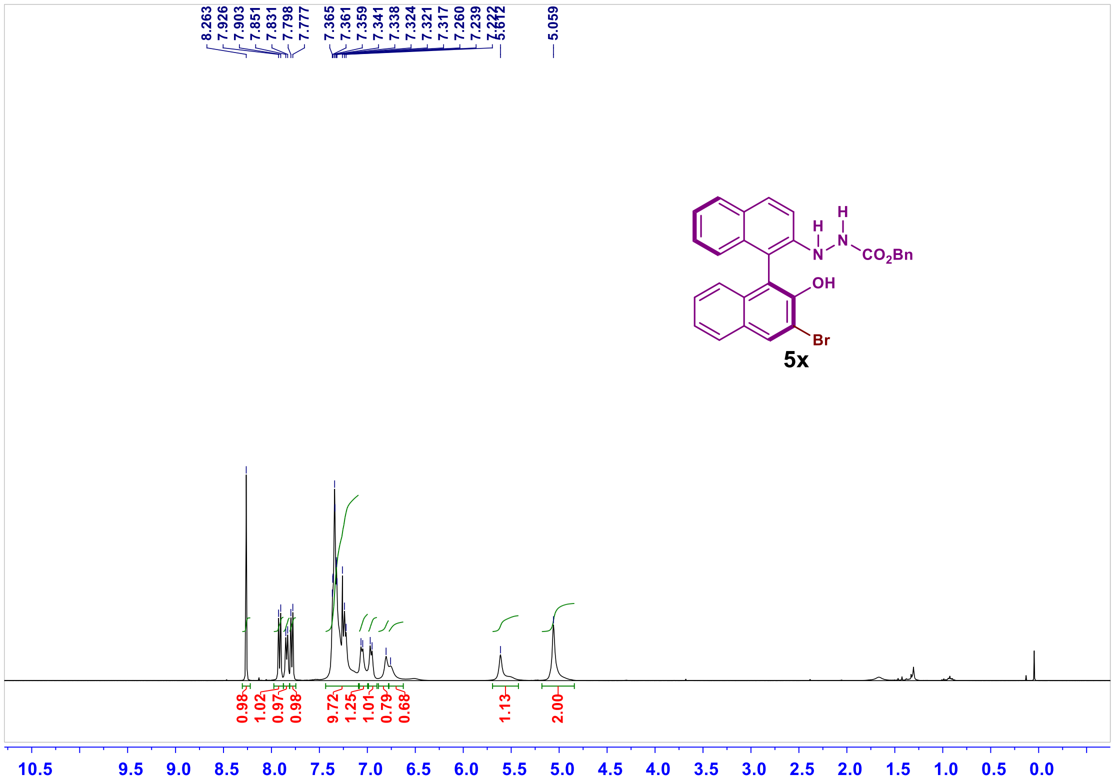

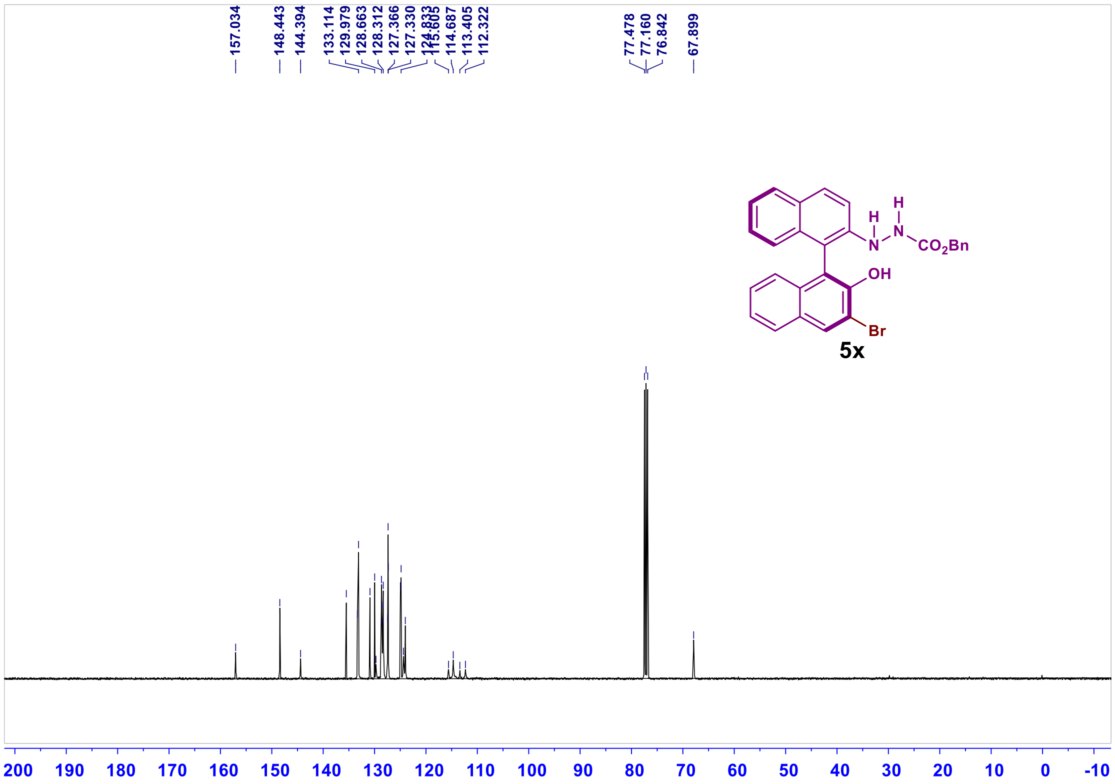

Supplementary Figure 136. ¹H and ¹³C NMR spectra of 5x

Supplementary Figure 137. HPLC spectra of (*S*)-benzyl 2-(3'-bromo-2'-hydroxy-[1,1'-binaphthalen]-2-yl)hydrazine-1-carboxylate (5x). Diacel Chiralpak AD-H, *n*-Hexane:*i*-PrOH = 80:20, flow = 1.0 mL/min, 25 °C, λ = 238 nm, t_R(major) = 8.2 min, t_R(minor) = 14.2 min, e.r. = 5:95

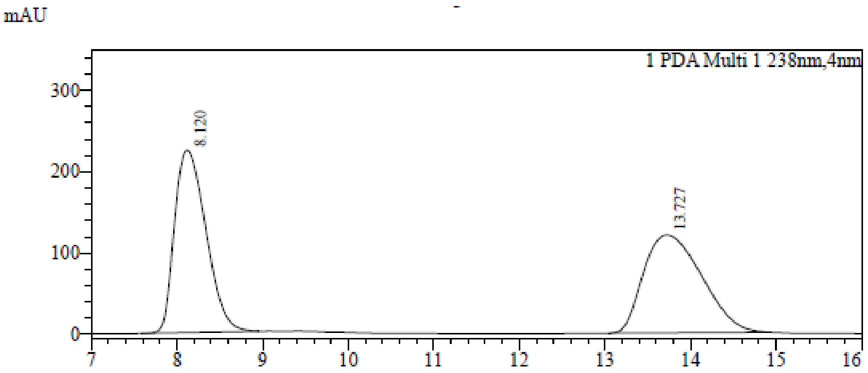

Peak Table

PDA Ch1 238nm

Peak#	Ret. Time	Peak End	Height	Area	Area%
1	8.120	8.960	224157	5737883	50.225
2	13.727	14.912	120259	5686484	49.775
Total			344416	11424368	100.000

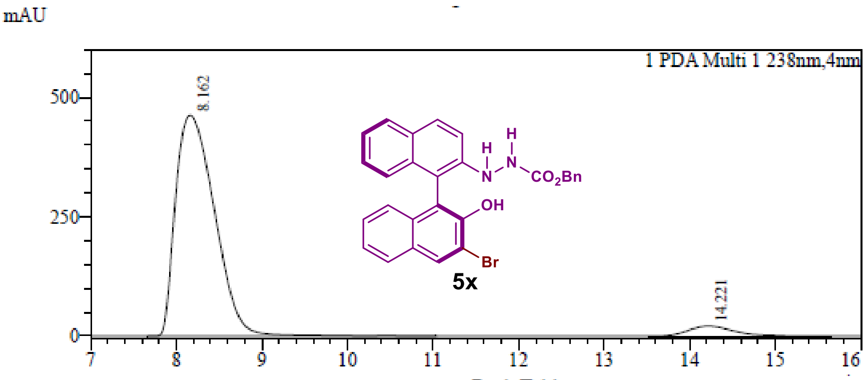

Peak Table

PDA Ch1 238nm

Peak#	Ret. Time	Peak End	Height	Area	Area%
1	8.162	11.040	462740	14695381	94.939
2	14.221	15.648	21034	783458	5.061
Total			483774	15478839	100.000

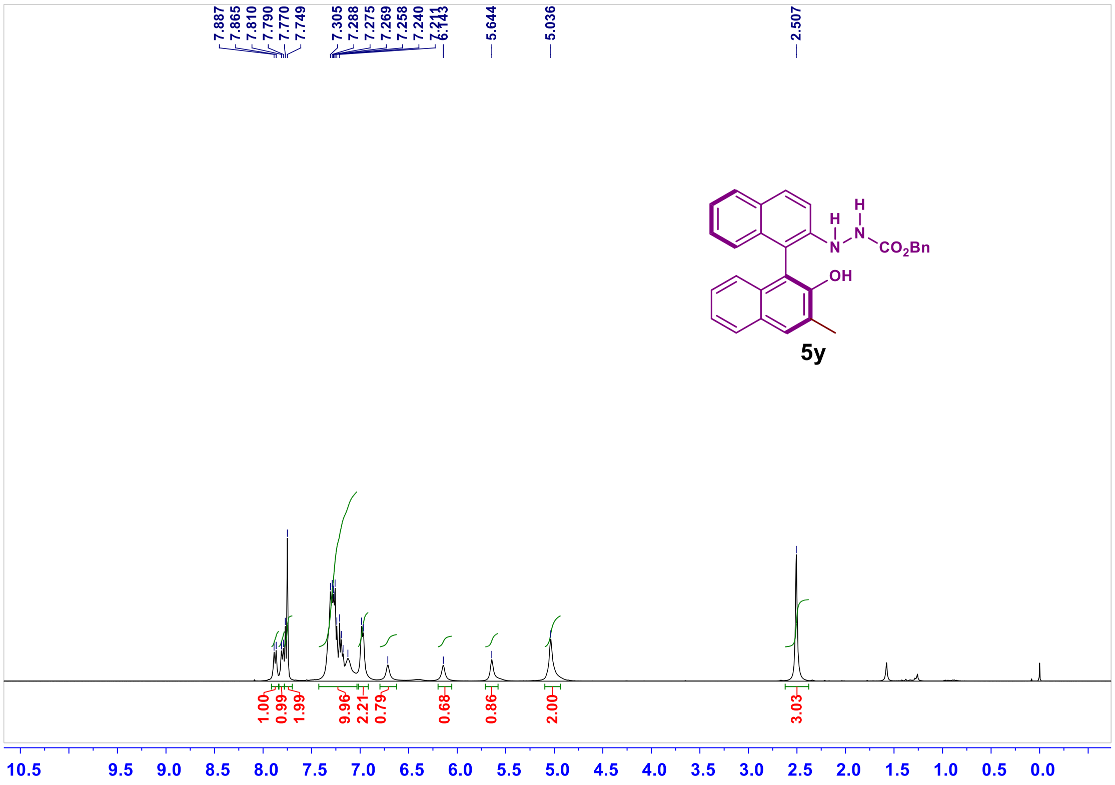

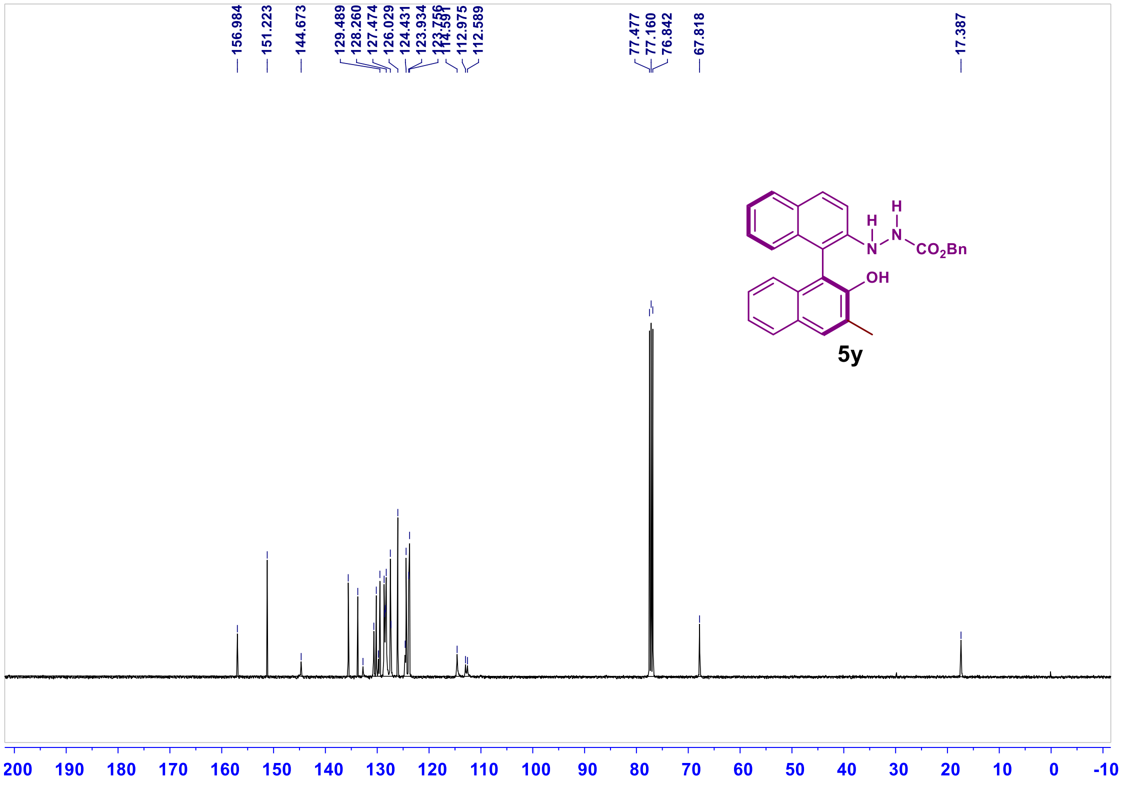

Supplementary Figure 138. ^{1}H and ^{13}C NMR spectra of 5y

Supplementary Figure 139. HPLC spectra of (*S*)-benzyl 2-(2'-hydroxy-3'-methyl-[1,1'-binaphthalen]-2-yl)hydrazine-1-carboxylate (5y). Diacel Chiralpak AD-H, *n*-Hexane:*i*-PrOH = 80:20, flow = 1.0 mL/min, 25 °C, λ = 232 nm, t_R(major) = 8.0 min, t_R(minor) = 10.2 min, e.r. = 3.5:96.5

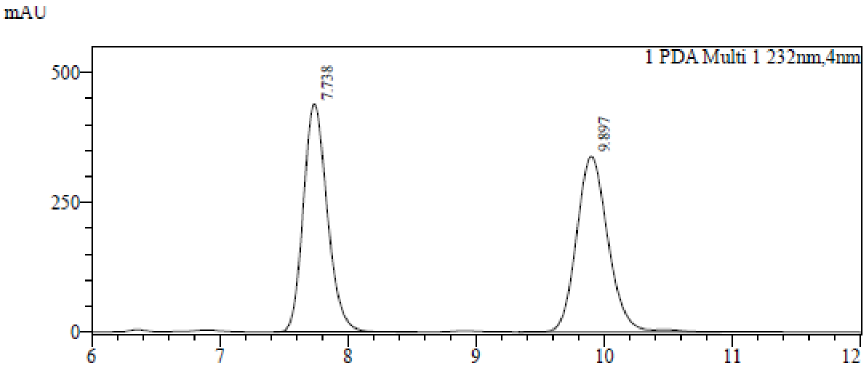

Peak Table

PDA Ch1 232nm

Peak#	Ret. Time	Peak End	Height	Area	Area%
1	7.738	8.533	438345	5716095	49.708
2	9.897	10.987	337914	5783252	50.292
Total			776259	11499347	100.000

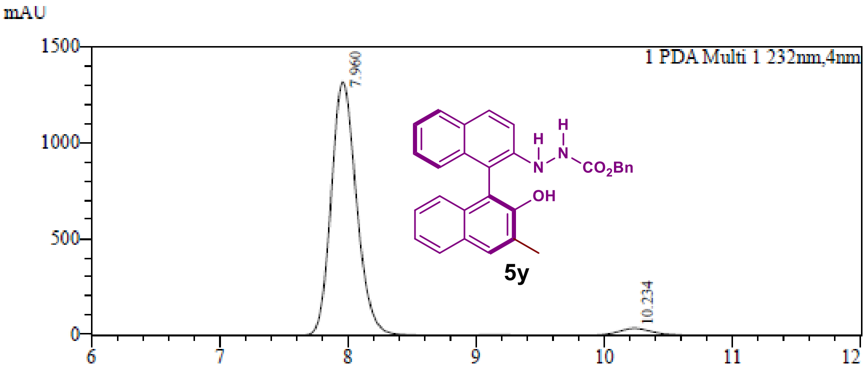

Peak Table

PDA Ch1 232nm

Peak#	Ret. Time	Peak End	Height	Area	Area%
1	7.960	8.736	1321695	18089910	96.549
2	10.234	10.709	36487	646612	3.451
Total			1358181	18736521	100.000

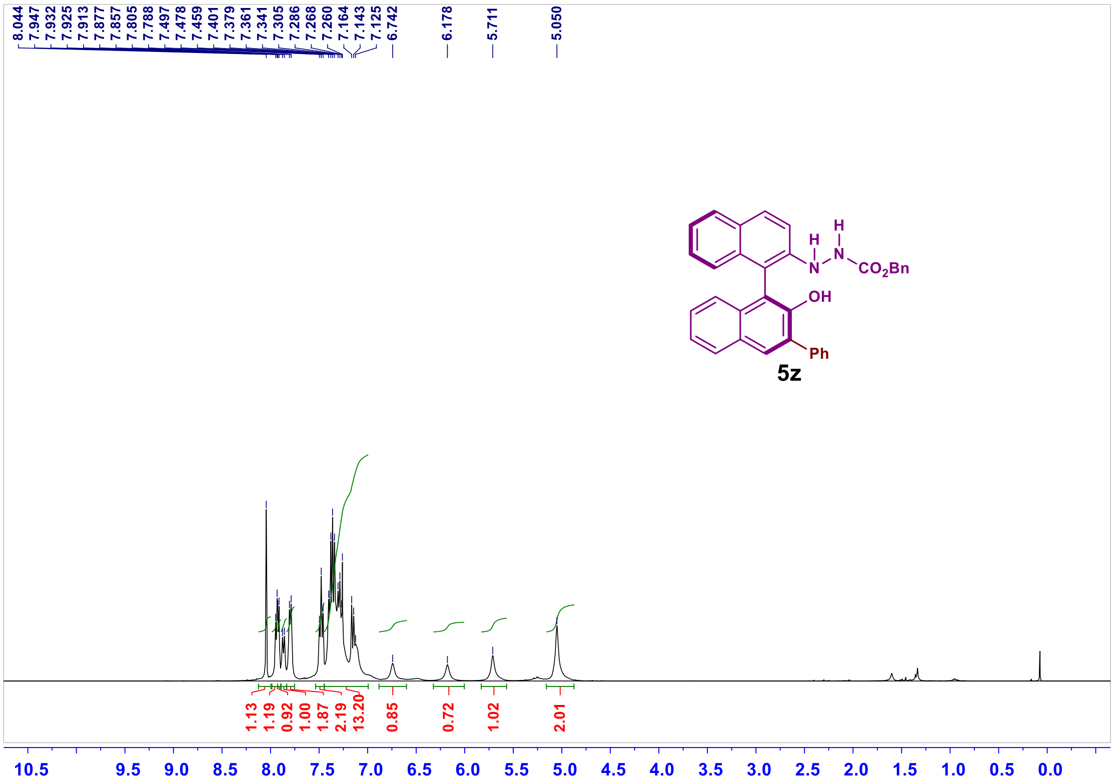

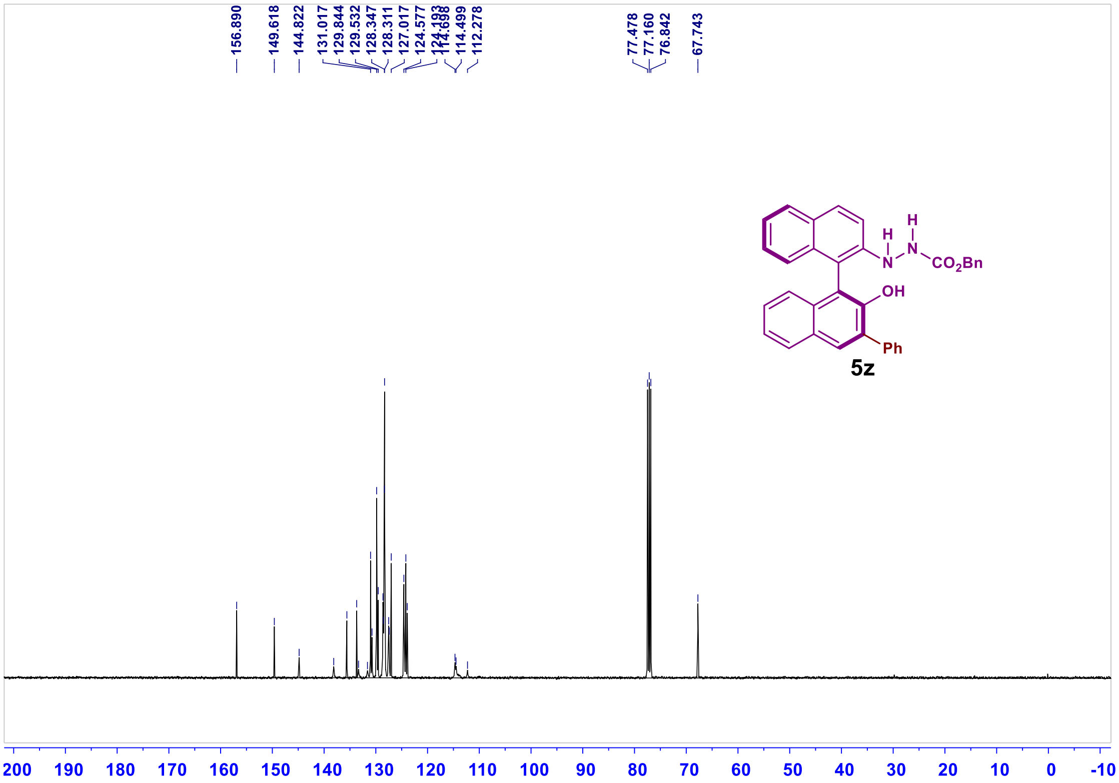

Supplementary Figure 140. ^{1}H **and** ^{13}C **NMR spectra of 5z**

Supplementary Figure 141. HPLC spectra of (*S*)-benzyl 2-(2'-hydroxy-3'-phenyl-[1,1'-binaphthalen]-2-yl)hydrazine-1-carboxylate (5z). Diacel Chiralpak AD-H, *n*-Hexane:*i*-PrOH = 80:20, flow = 1.0 mL/min, 25 °C, λ = 242 nm, t_R(major) = 8.2 min, t_R(minor) = 10.6 min, e.r. = 6.5:93.5

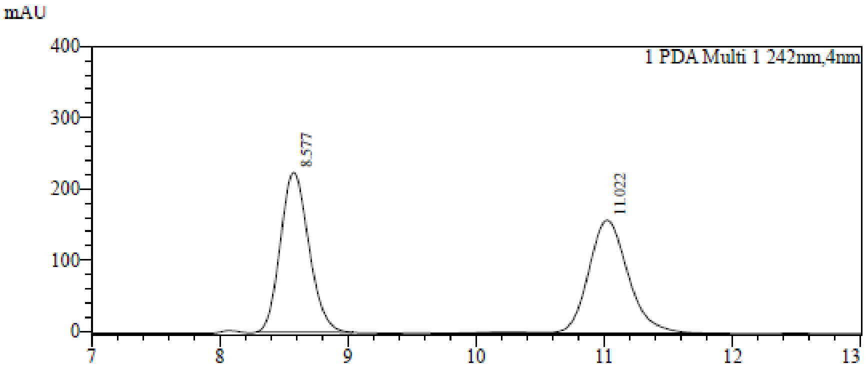

Peak Table

PDA Ch1 242nm

Peak#	Ret. Time	Peak End	Height	Area	Area%
1	8.577	9.035	224572	3483941	50.192
2	11.022	11.861	158912	3457276	49.808
Total			383484	6941218	100.000

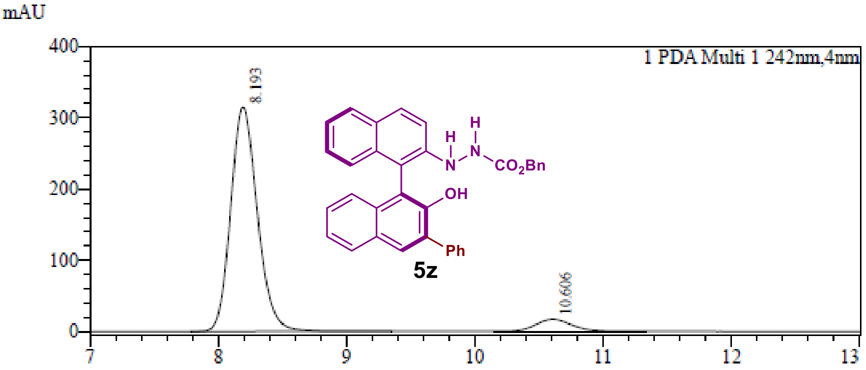

Peak Table

PDA Ch1 242nm

Peak#	Ret. Time	Peak End	Height	Area	Area%
1	8.193	9.355	314840	4555939	93.331
2	10.606	11.328	16867	325535	6.669
Total			331706	4881474	100.000

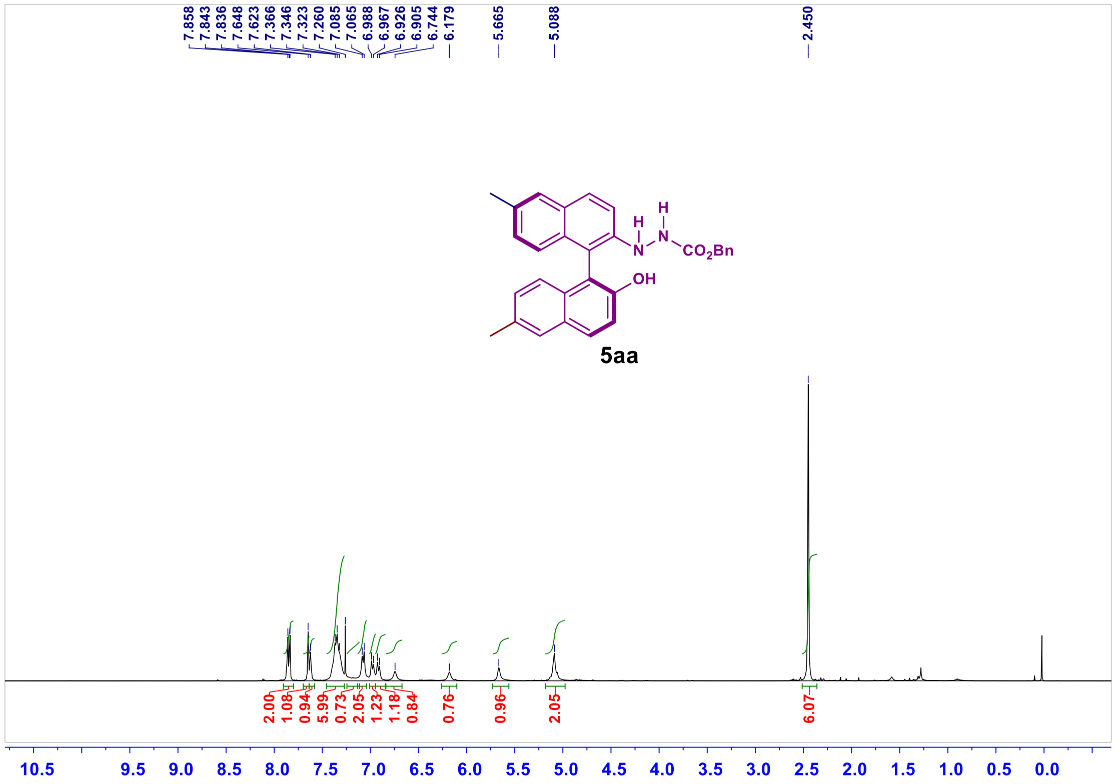

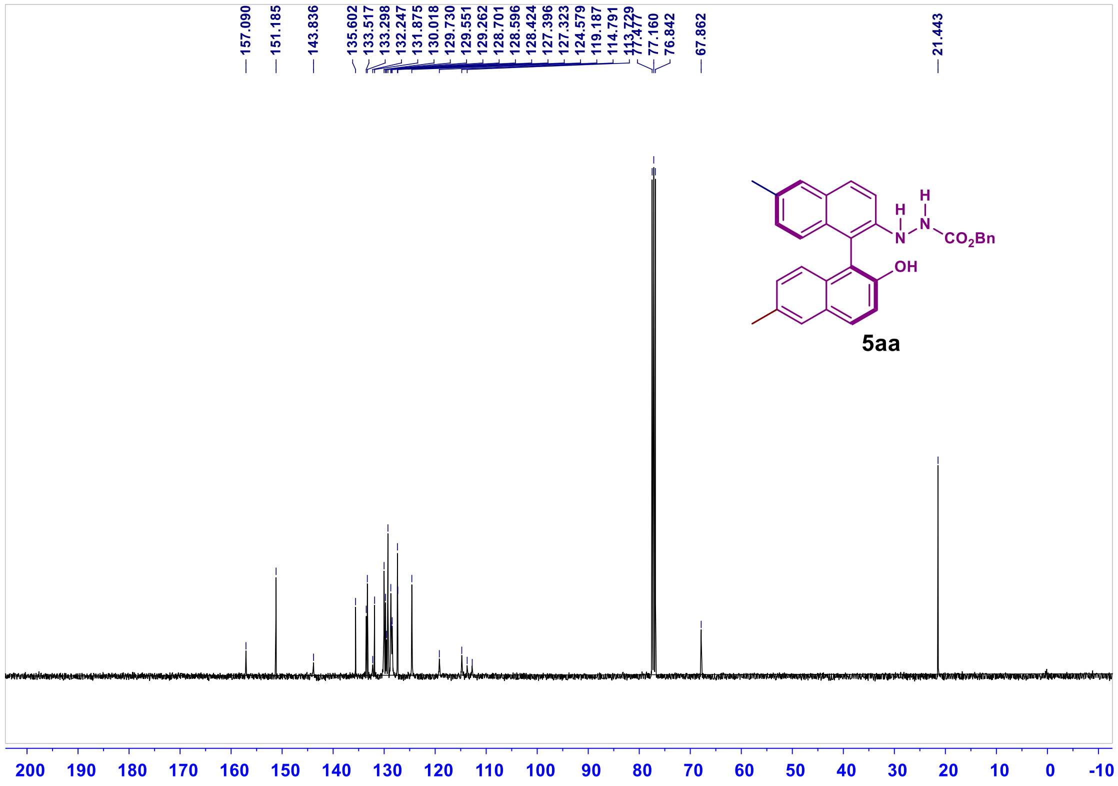

Supplementary Figure 142. ^{1}H and ^{13}C NMR spectra of 5aa

Supplementary Figure 143. HPLC spectra of (*S*)-benzyl 2-(2'-hydroxy-6,6'-dimethyl-[1,1'-binaphthalen]-2-yl)hydrazine-1-carboxylate (5aa). Diacel Chiralcel OD-H, *n*-Hexane:*i*-PrOH = 80:20, flow = 1.0 mL/min, 25 °C, λ = 231 nm, t_R(major) = 15.6 min, t_R(minor) = 13.1 min, e.r. = 6:94

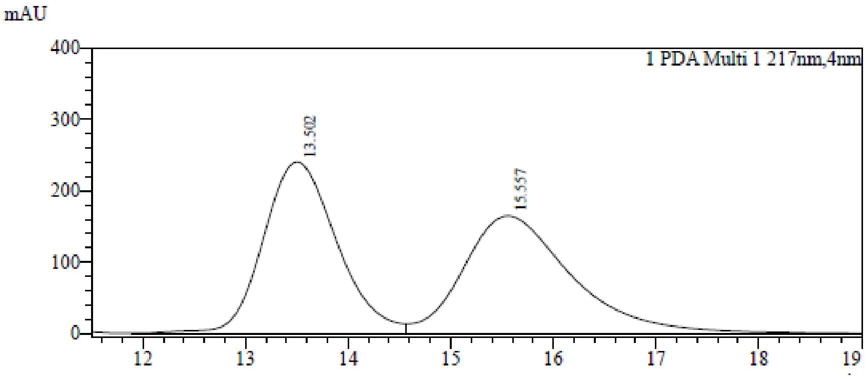

Peak Table

PDA Ch1 217nm

Peak#	Ret. Time	Peak End	Height	Area	Area%
1	13.502	14.571	241078	12094591	49.731
2	15.557	19.680	165270	12225364	50.269
Total			406348	24319955	100.000

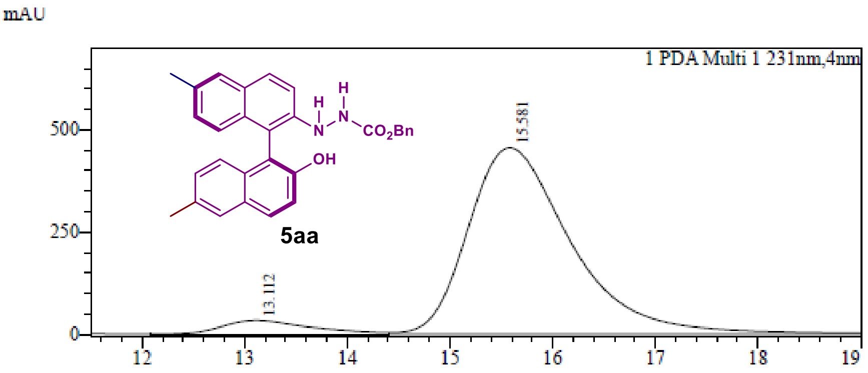

Peak Table

PDA Ch1 231nm

Peak#	Ret. Time	Peak End	Height	Area	Area%
1	13.112	14.411	34013	2028881	5.971
2	15.581	21.088	455539	31952261	94.029
Total			489552	33981142	100.000

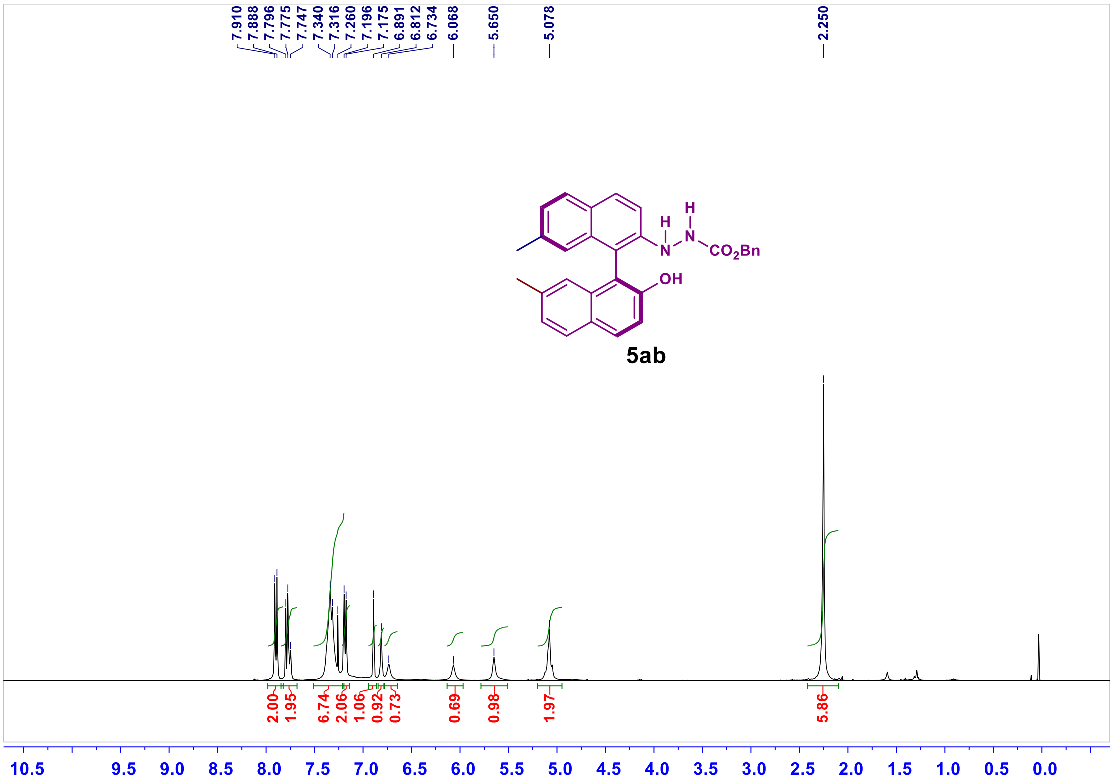

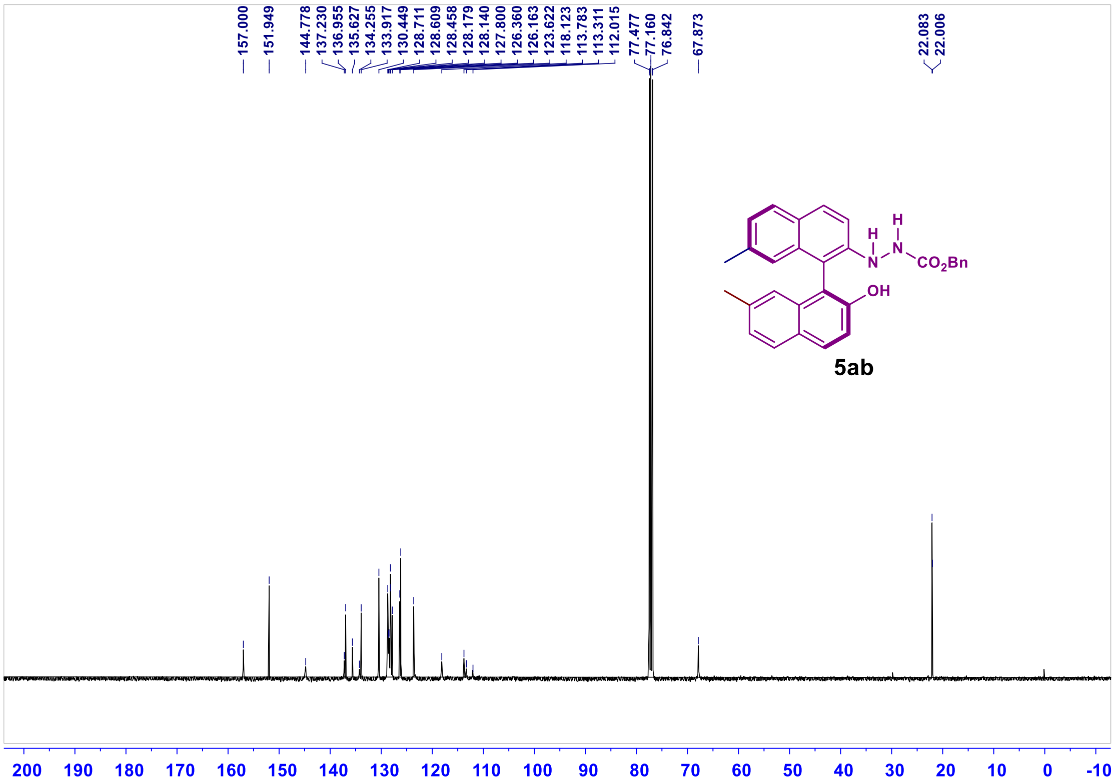

Supplementary Figure 144. ^{1}H and ^{13}C NMR spectra of 5ab

Supplementary Figure 145. HPLC spectra of (*S*)-benzyl 2-(2'-hydroxy-7,7'-dimethyl-[1,1'-binaphthalen]-2-yl)hydrazine-1-carboxylate (5ab). Diacel Chiralpak AD-H, *n*-Hexane:*i*-PrOH = 80:20, flow = 1.0 mL/min, 25 °C, λ = 234 nm, t_R(major) = 10.2 min, t_R(minor) = 7.5 min, e.r. = 4.5:95.5

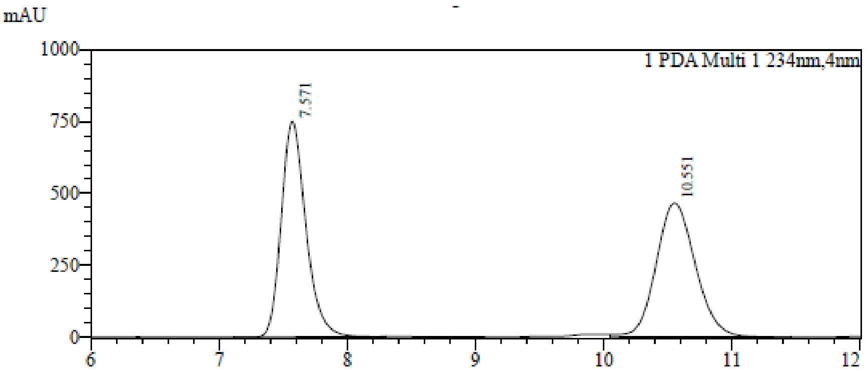

Peak Table

PDA Ch1 234nm

Peak#	Ret. Time	Peak End	Height	Area	Area%
1	7.571	8.299	750526	10057330	50.346
2	10.551	11.456	466409	9919068	49.654
Total			1216935	19976398	100.000

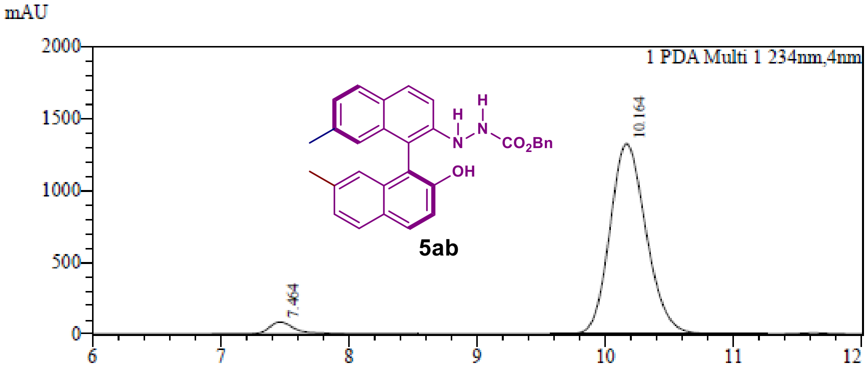

Peak Table

PDA Ch1 234nm

Peak#	Ret. Time	Peak End	Height	Area	Area%
1	7.464	8.533	85366	1223068	4.472
2	10.164	11.264	1324641	26123782	95.528
Total			1410007	27346851	100.000

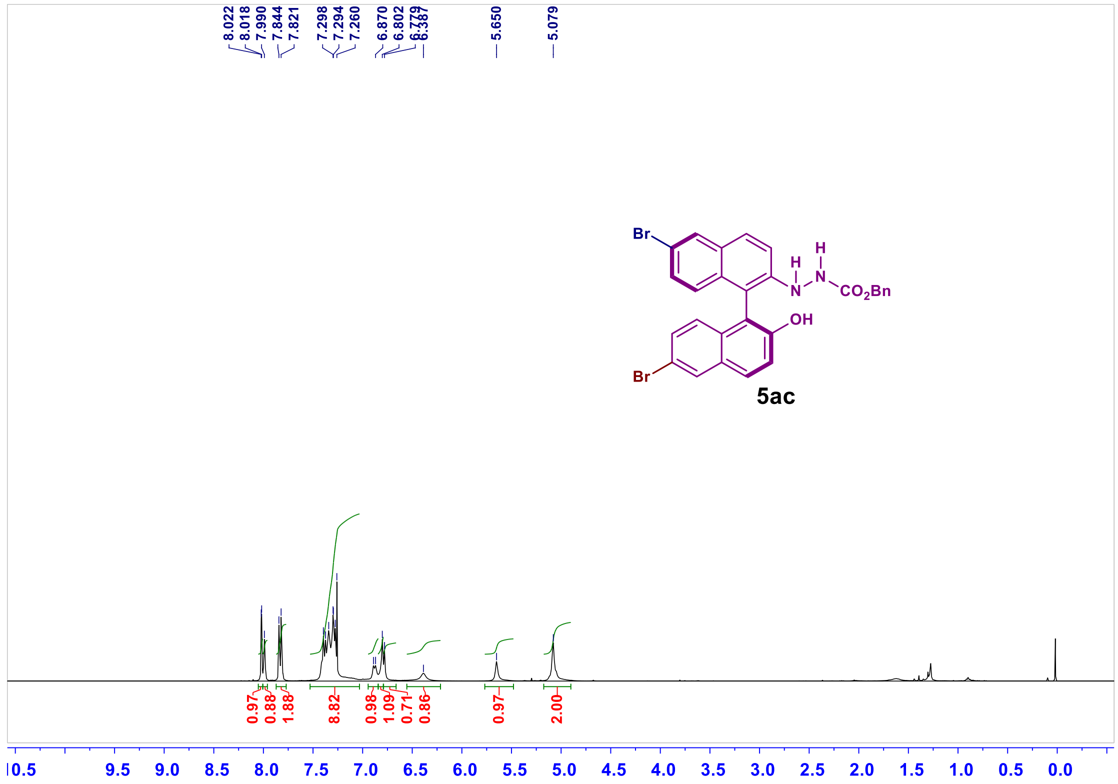

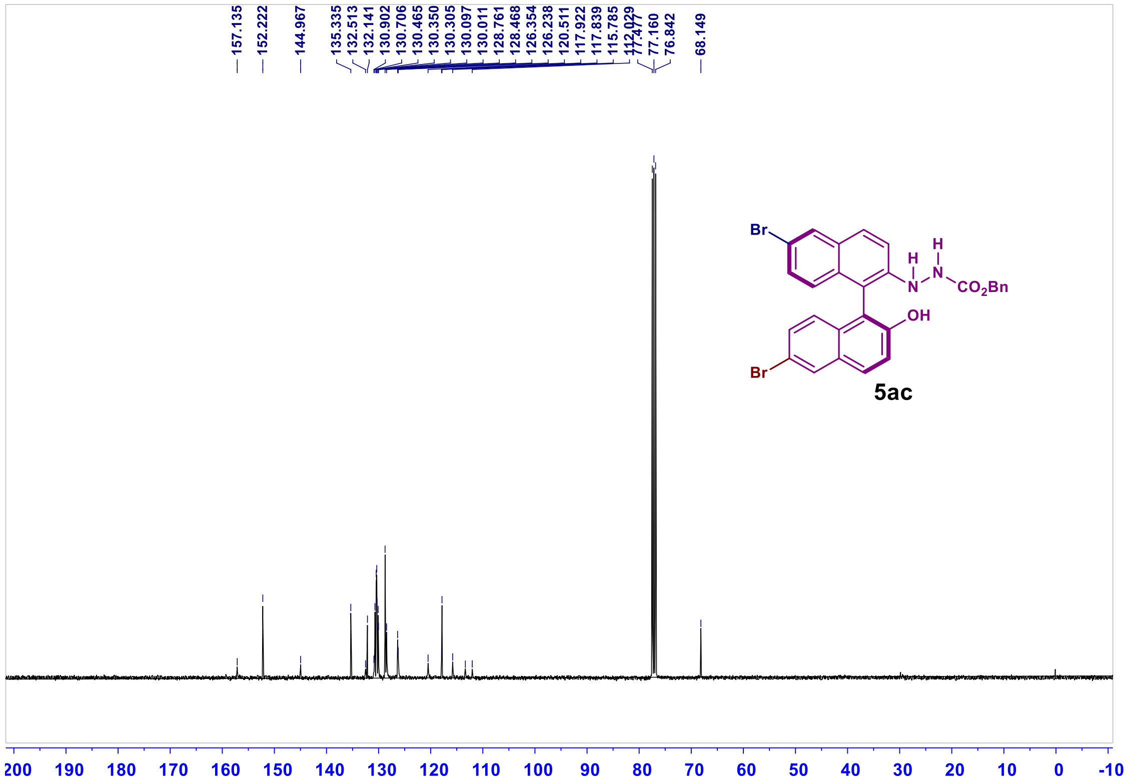

Supplementary Figure 146. ^{1}H and ^{13}C NMR spectra of 5ac

Supplementary Figure 147. HPLC spectra of (*S*)-benzyl 2-(6,6'-dibromo-2'-hydroxy-[1,1'-binaphthalen]-2-yl)hydrazine-1-carboxylate (5ac). Diacel Chiralpak AD-H, *n*-Hexane:*i*-PrOH = 80:20, flow = 1.0 mL/min, 25 °C, λ = 236 nm, t_R(major) = 14.0 min, t_R(minor) = 11.7 min, e.r. = 6:94

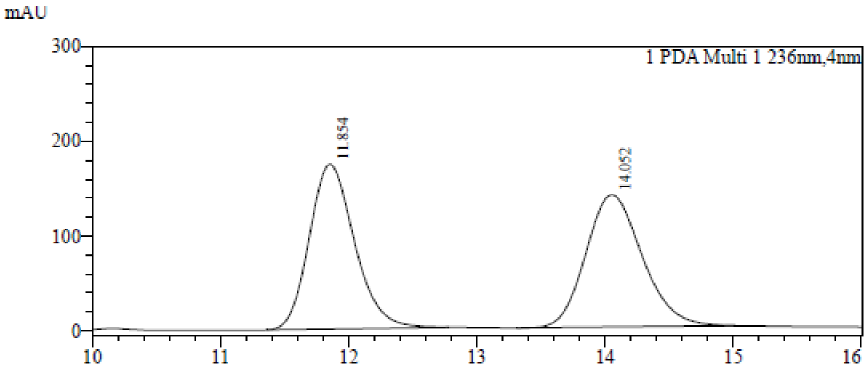

Peak Table

PDA Ch1 236nm

Peak#	Ret. Time	Peak End	Height	Area	Area%
1	11.854	12.779	173770	4265949	50.013
2	14.052	15.008	139422	4263660	49.987
Total			313192	8529609	100.000

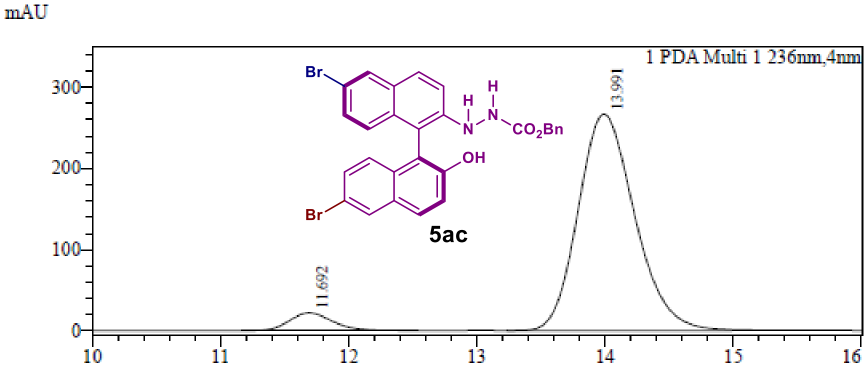

Peak Table

PDA Ch1 236nm

Peak#	Ret. Time	Peak End	Height	Area	Area%
1	11.692	12.533	21899	518125	5.938
2	13.991	15.776	266446	8207314	94.062
Total			288345	8725439	100.000

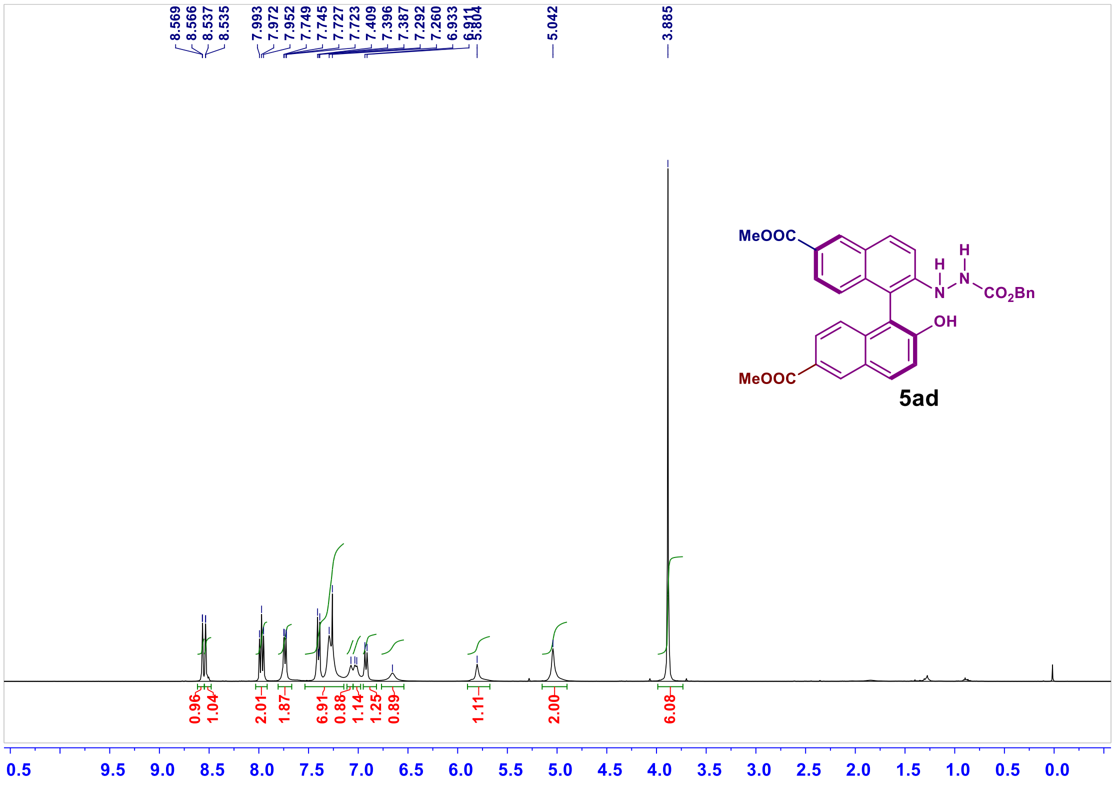

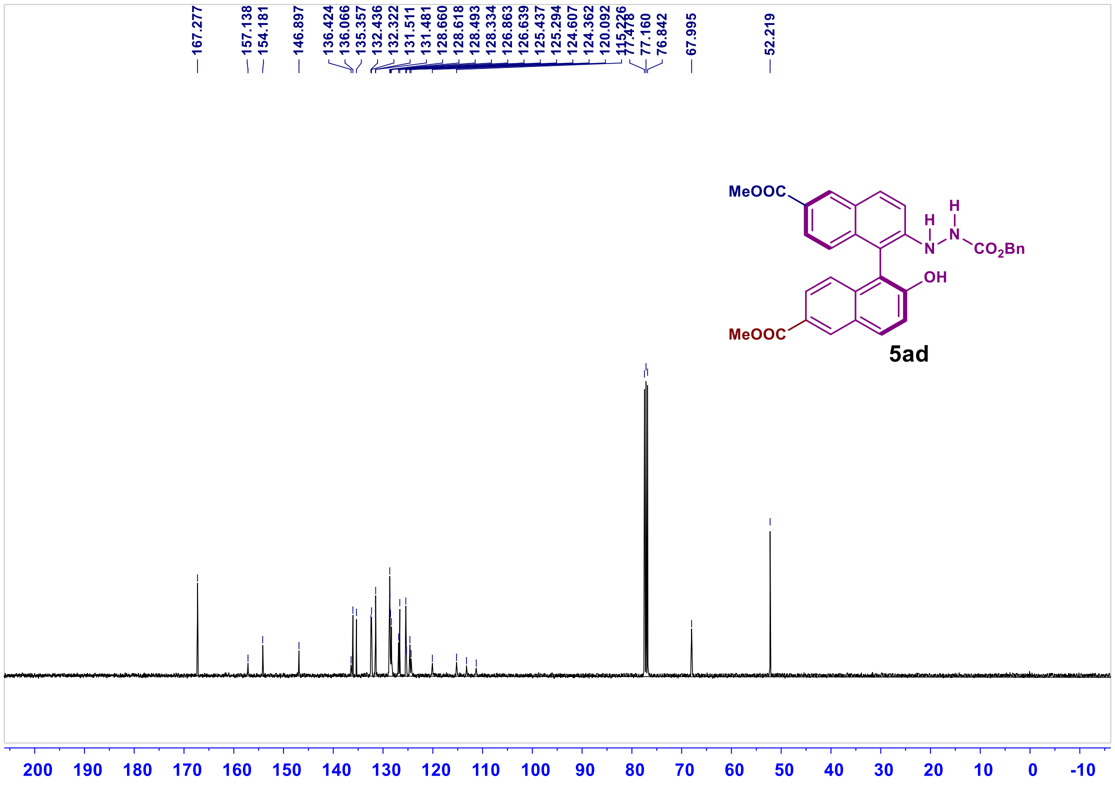

Supplementary Figure 148. ^{1}H and ^{13}C NMR spectra of 5ad

Supplementary Figure 149. HPLC spectra of (*S*)-dimethyl 2-(2-((benzyloxy)carbonyl)hydrazinyl)-2'-hydroxy-[1,1'-binaphthalene]-6,6'-dicarboxylate (5ad). Diacel Chiralpak AD-H, *n*-Hexane:*i*-PrOH = 70:30, flow = 1.0 mL/min, 25 °C, λ = 246 nm, t_R(major) = 34.0 min, t_R(minor) = 14.0 min, e.r. = 6:94

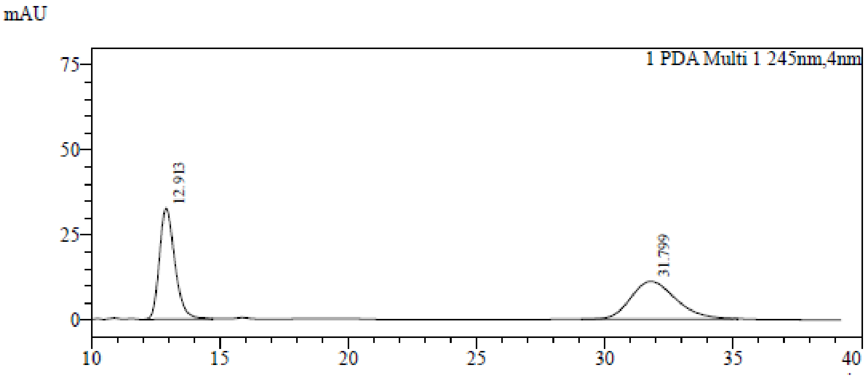

Peak Table

PDA Ch1 245nm

Peak#	Ret. Time	Peak End	Height	Area	Area%
1	12.913	14.731	32622	1360327	50.303
2	31.799	35.179	11063	1343916	49.697
Total			43685	2704243	100.000

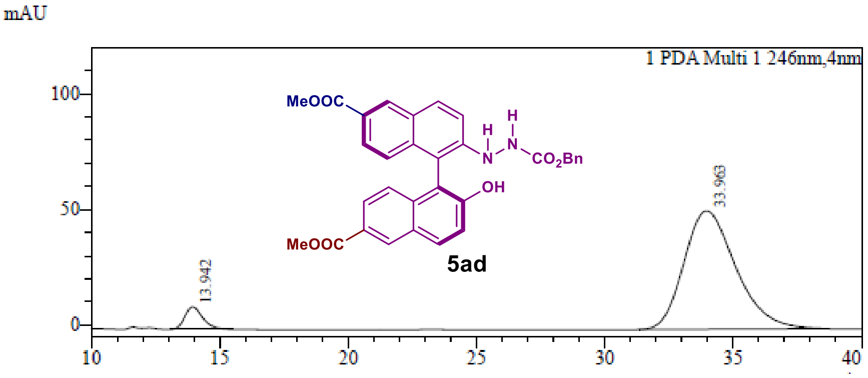

Peak Table

PDA Ch1 246nm

Peak#	Ret. Time	Peak End	Height	Area	Area%
1	13.942	15.541	9701	472225	6.150
2	33.963	38.613	51185	7206176	93.850
Total			60887	7678401	100.000

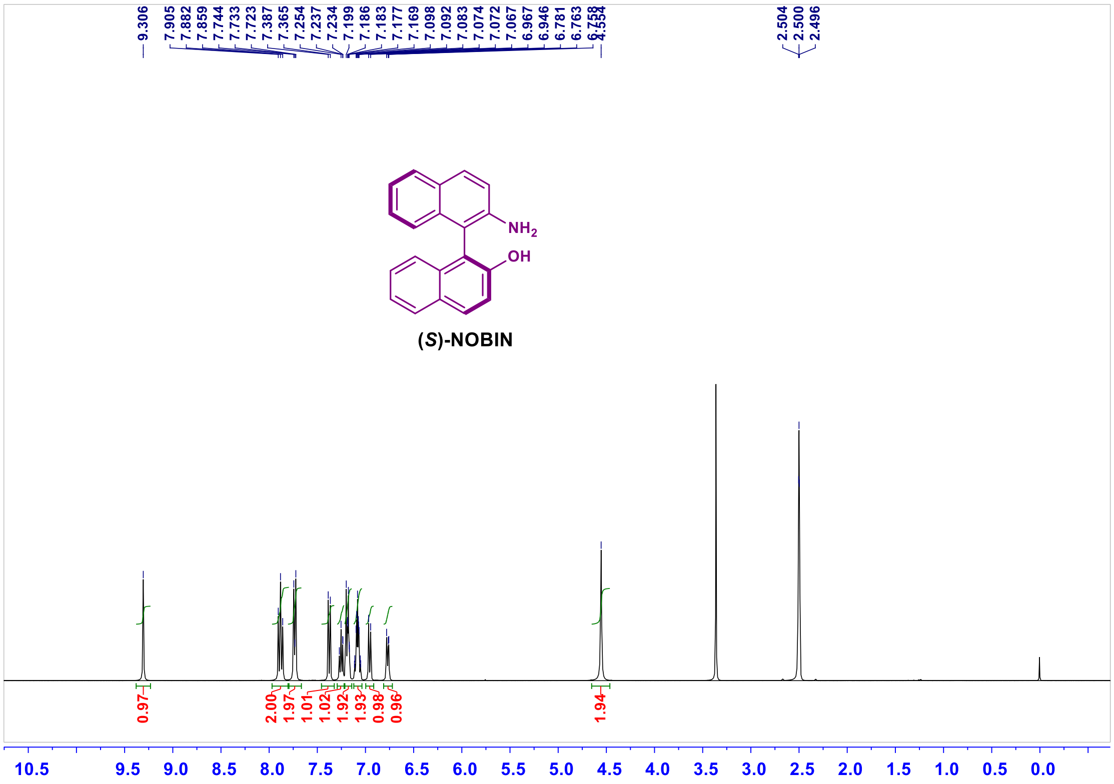

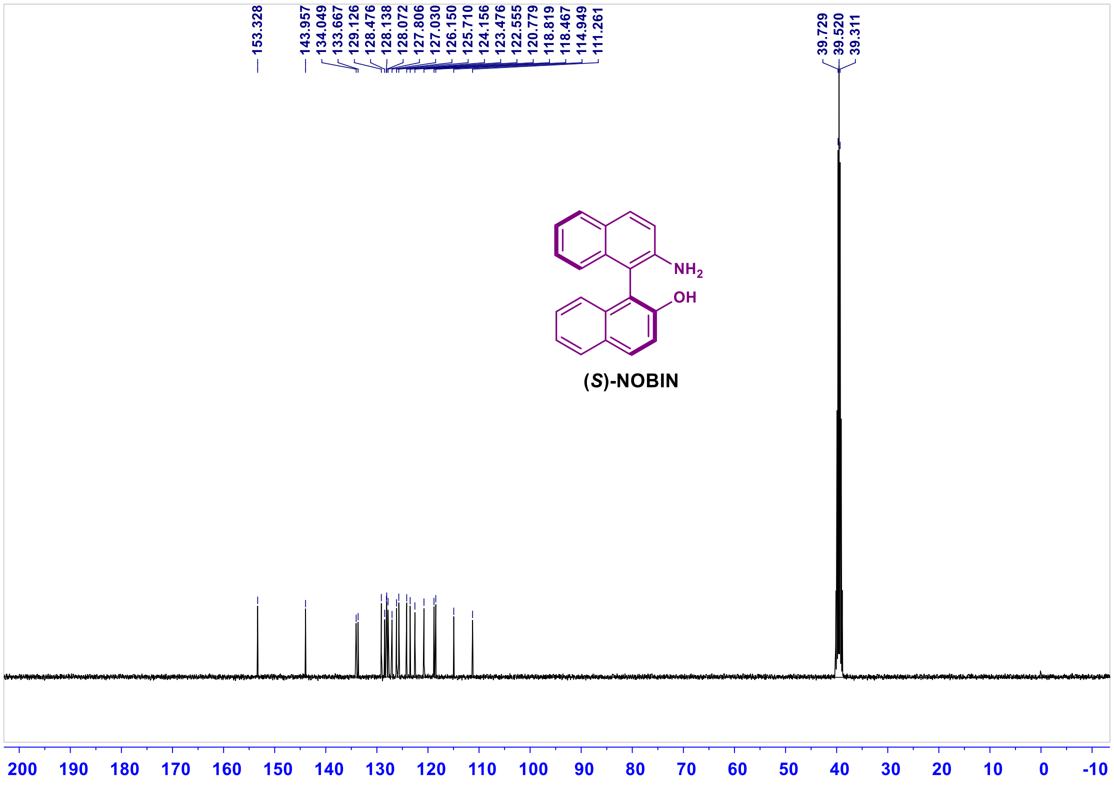

Supplementary Figure 150. ^{1}H and ^{13}C NMR spectra of (*S*)-NOBIN

Supplementary Figure 151. HPLC spectra of (*S*)-NOBIN. Diacel Chiralpak AD-H, *n*-Hexane:*i*-PrOH = 70:30, flow = 1.0 mL/min, 25 °C, λ = 254 nm, t_R(major) = 14.8 min, t_R(minor) = 7.9 min, e.r. = 6:94

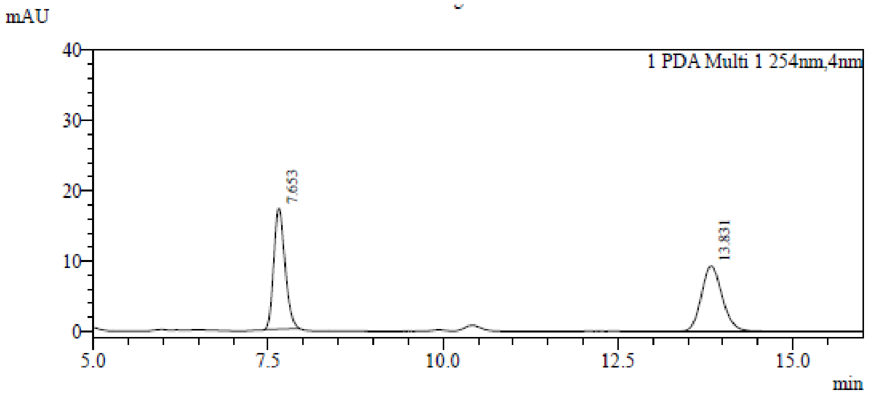

Peak Table

PDA Ch1 254nm

Peak#	Ret. Time	Peak End	Height	Area	Area%
1	7.653	7.947	17155	192114	49.913
2	13.831	15.808	9270	192782	50.087
Total			26425	384896	100.000

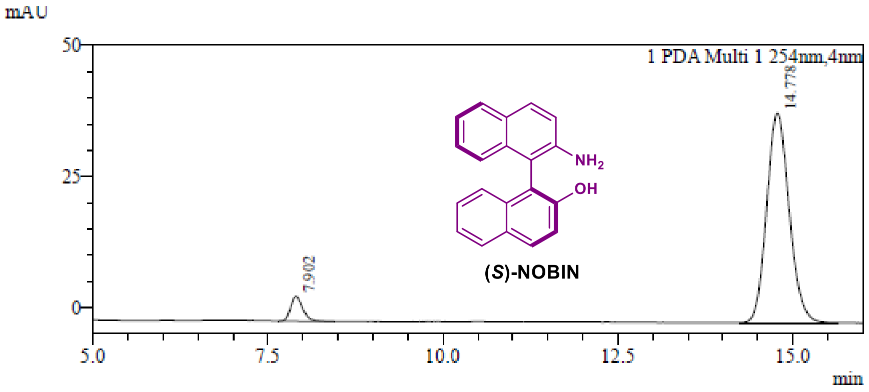

Peak Table

PDA Ch1 254nm

Peak#	Ret. Time	Peak End	Height	Area	Area%
1	7.902	8.448	4736	57349	6.012
2	14.778	15.637	39960	896601	93.988
Total			44696	953950	100.000

6. X-Ray Crystallographic Data

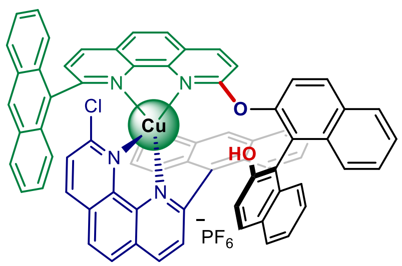

(A)

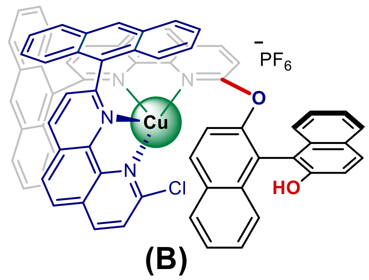

(B)

Cu-1

(CCDC 2096699)

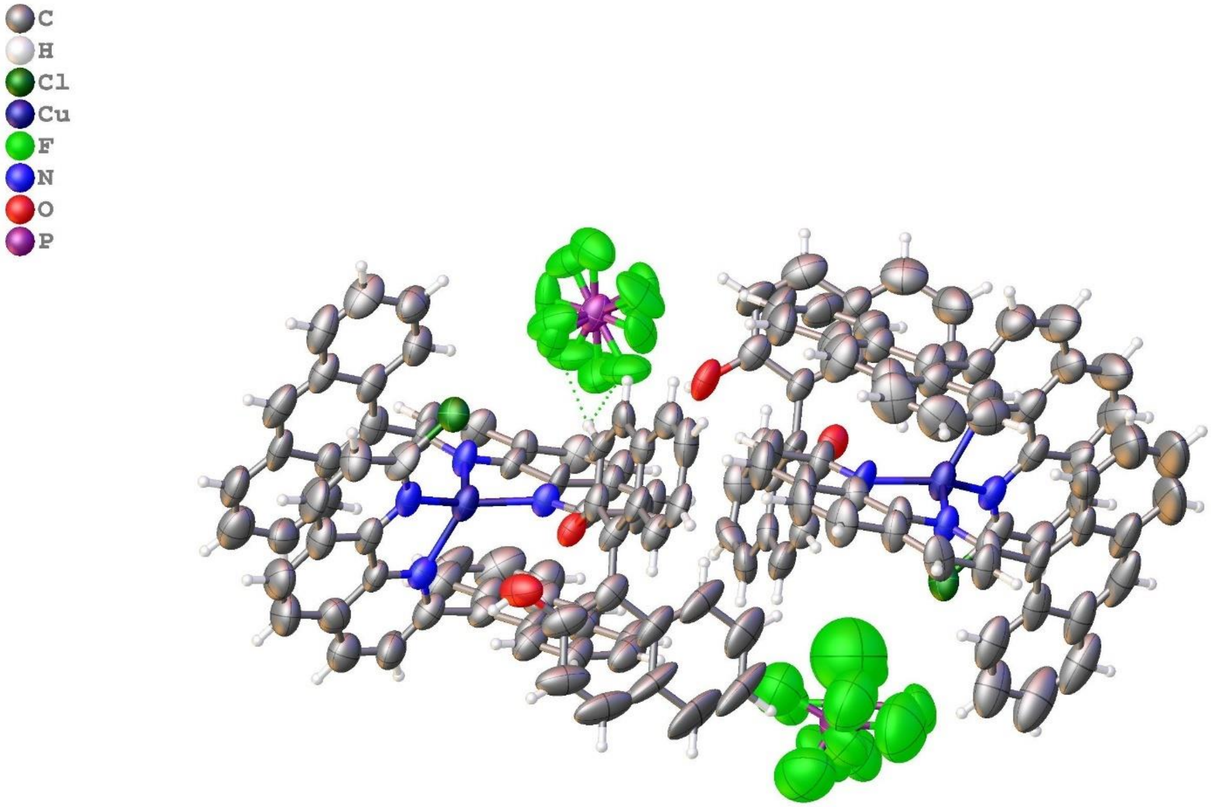

(thermal ellipsoids at 30% probability)

Supplementary Table 9. Crystal data and structure refinement for mo_d8v21018_0m.

Identification code	mo_d8v21018_0m	
Empirical formula	$C_{72} H_{43} Cl Cu F_6 N_4 O_2 P$	
Formula weight	1240.06	
Temperature	193(2) K	
Wavelength	0.71073 Å	
Crystal system	Triclinic	
Space group	P 1	
Unit cell dimensions	a = 14.3327(15) Å	α= 64.323(3)°.
	b = 14.7844(17) Å	β= 75.896(3)°.
	c = 16.615(2) Å	γ = 81.934(3)°.
Volume	3074.9(6) $Å^3$	
Z	2	
Density (calculated)	1.339 Mg/m^3	
Absorption coefficient	0.493 mm^{-1}	
F(000)	1268	
Crystal size	0.180 x 0.130 x 0.080 mm^3	
Theta range for data collection	1.582 to 24.999°.	
Index ranges	-17<=h<=16, -17<=k<=16, -19<=l<=19	
Reflections collected	41553	
Independent reflections	18679 [R(int) = 0.0580]	
Completeness to theta = 25.242°	97.1 %	
Absorption correction	Semi-empirical from equivalents	
Max. and min. transmission	0.7456 and 0.6116	
Refinement method	Full-matrix least-squares on F^2	
Data / restraints / parameters	18679 / 115 / 1650	
Goodness-of-fit on F^2	0.943	
Final R indices [I>2sigma(I)]	R1 = 0.0630, wR2 = 0.1450	
R indices (all data)	R1 = 0.1135, wR2 = 0.1693	
Absolute structure parameter	0.106(11)	
Extinction coefficient	n/a	
Largest diff. peak and hole	0.361 and -0.425 e.$Å^{-3}$	

Explanations for the A- or B-level alerts in the CheckCIF report.

Complex Cu-1 (CCDC 2096699) mo_d8v21018_0m

Alert level B

PLAT234_ALERT_4_B Large Hirshfeld Difference C45A --C46A . 0.28 Ang.

Explanation: The constraint was performed on these atoms during the refinement.

PLAT234_ALERT_4_B Large Hirshfeld Difference P1 --F6′ . 0.26 Ang.

Explanation: The constraint was performed on these atoms during the refinement.

PLAT341_ALERT_3_B Low Bond Precision on C-C Bonds .... 0.01948 Ang.

Explanation: The low bond precision is due to the quality of the crystal and the obtained data.

PLAT420_ALERT_2_B D-H Bond Without Acceptor O2A --H2AA . Please Check

PLAT420_ALERT_2_B D-H Bond Without Acceptor O2 --H2 . Please Check

Explanation: The hydrogen bonding acceptor comes from the disordered solvent molecules (dichloromethane, methanol or petroleum ether) which cannot be determined.

PLAT987_ALERT_1_B The Flack x is >> 0 - Do a BASF/TWIN Refinement Please Check

Explanation: The relative high Flack parameter is due to the insufficient reflection point at high angle. The reflection cycle is cut above dmin = 0.84 A.

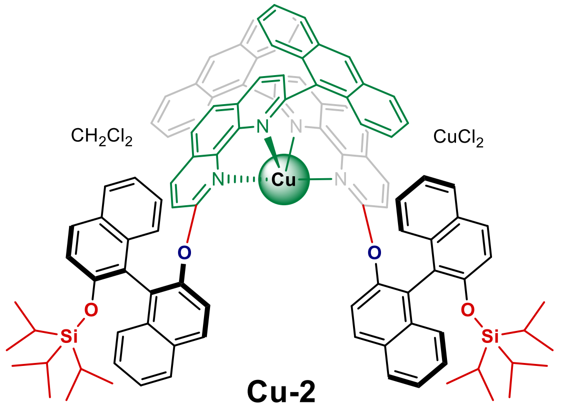

(CCDC 2096715)

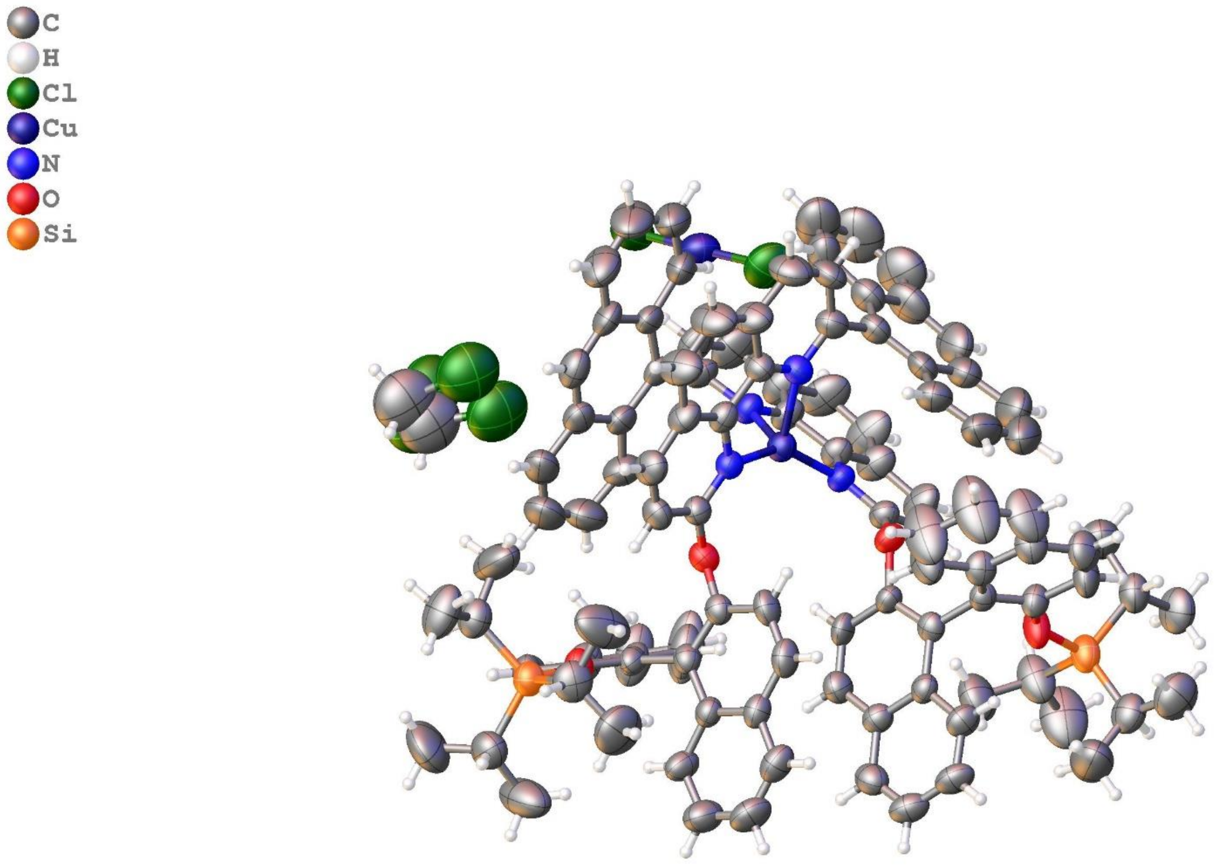

(thermal ellipsoids at 30% probability)

Supplementary Table 10. Crystal data and structure refinement for mo_d8v21187_0m.

Identification code	mo_d8v21187_0m	
Empirical formula	$C_{111} H_{98} Cl_4 Cu_2 N_4 O_4 Si_2$	
Formula weight	1876.99	
Temperature	293(2) K	
Wavelength	0.71073 Å	
Crystal system	Orthorhombic	
Space group	P 21 21 21	
Unit cell dimensions	a = 11.0082(6) Å	α= 90°.
	b = 21.9952(10) Å	β= 90°.
	c = 39.706(2) Å	γ = 90°.
Volume	9614.0(9) $Å^3$	
Z	4	
Density (calculated)	1.297 Mg/m^3	
Absorption coefficient	0.634 mm^{-1}	
F(000)	3912	
Crystal size	0.180 x 0.150 x 0.130 mm^3	
Theta range for data collection	2.309 to 25.500°.	
Index ranges	-12<=h<=13, -24<=k<=26, -43<=l<=48	
Reflections collected	46700	
Independent reflections	17826 [R(int) = 0.0485]	
Completeness to theta = 25.242°	99.8 %	
Absorption correction	Semi-empirical from equivalents	
Max. and min. transmission	0.7456 and 0.6481	
Refinement method	Full-matrix least-squares on F^2	
Data / restraints / parameters	17826 / 64 / 1184	
Goodness-of-fit on F^2	1.030	
Final R indices [I>2sigma(I)]	R1 = 0.0578, wR2 = 0.1348	
R indices (all data)	R1 = 0.1094, wR2 = 0.1659	
Absolute structure parameter	0.002(6)	
Extinction coefficient	0.0022(3)	
Largest diff. peak and hole	0.791 and -0.409 e.$Å^{-3}$	

Explanations for the A- or B-level alerts in the CheckCIF report.

Complex Cu-3 (CCDC 2096715) mo_d8v21187_0m

Alert level B

PLAT910_ALERT_3_B Missing # of FCF Reflection(s) Below Theta(Min). 13 Note

Explanation: This alert is probably due to the obstruction of the reflection points by the beam stop.

Supplementary References

1. Ma, G., Deng, J. & Sibi, M. P. Fluxionally chiral DMAP catalysts: kinetic resolution of axially chiral biaryl compounds. *Angew. Chem. Int. Ed.* **53**, 11818–11821 (2014).
2. Krapcho, A. P. & Lanza, J. B. Improved synthesis of 2-chloro- and 2, 9-dichloro-1, 10-phenanthrolines, *Org. Prep. Proced. Int.*, **39**, 603–608 (2007).
3. Fan, M., Zhou, W., Jiang, Y. & Ma, D. CuI/oxalamide catalyzed couplings of (hetero)aryl chlorides and phenols for diaryl ether formation. *Angew. Chem. Int. Ed*. **55**, 6211–6215 (2016).
4. L.-W. Qi, S. Li, S.-H. Xiang, J. Wang, B. Tan, Asymmetric construction of atropisomeric biaryls via a redox neutral cross-coupling strategy. *Nat. Catal.* **2**, 314–323 (2019).
